# Supplementary material for: Sulfonamide-directed site-selective functionalization of unactivated C(sp3)−H enabled by photocatalytic sequential electron/proton transfer
Source: Nat Commun. 2024 Jun 14;15:5087. doi: 10.1038/s41467-024-49337-3 (PMC11178871; doi:10.1038/s41467-024-49337-3)
Supplement: Supplementary file 1 — Supplementary Information [file 41467_2024_49337_MOESM1_ESM.pdf]

Supplementary Information

Sulfonamide-Directed Site-Selective Functionalization of Unactivated C(sp^3)−H Enabled by Photocatalytic Sequential Electron/Proton Transfer

Chaodong Wang,[1] Zhi Chen,[1] Jie Sun,[1] Luwei Tong,[1] Wenjian Wang,[1] Shengjie Song,[1] and Jianjun Li[1,2]*

[1]Key Laboratory for Green Pharmaceutical Technologies and Related Equipment of Ministry of Education, College of Pharmaceutical Sciences, Zhejiang University of Technology, Hangzhou 310014, P. R. of China

[2]Taizhou Key Laboratory of Advanced Manufacturing Technology, Taizhou Institute, Zhejiang University of Technology, Taizhou 318014, P. R. of China

*Corresponding author. Email: lijianjun@zjut.edu.cn

Table of content

1. General information 3

2. General procedure 4

2.1 General procedure A: Preparation of *N*-protected amines substrates from free amines and sulfonyl chloride. 4

2.2 General procedure B: Preparation of *N*-protected amines substrates from 4-methoxybenzenesulfonamide and alkyl bromide. 4

2.3 General procedure C: Preparation of *N*-protected amines substrates from free alcohol intermediate and carboxylic acids 5

2.4 General procedure D: Preparation of *N*-protected amines substrates from free carboxylic acid intermediate and alcohols 5

2.5 General procedure E: Remote $C(sp^3)$–H heteroarylation of sulfonamides 6

2.6 General procedure F: Dehydrogenative $C(sp^3)$-H heteroarylation of alkanes 7

2.7 General procedure G: Remote $C(sp^3)$–H functionalization of sulfonamides 7

2.8 Procedures for preparation of specific compounds 7

3. Unsuccessful substrates 11

4. Gram-scale experiments 11

4.1 Gram-scale experiments in batch 11

4.2 Gram-scale experiments in continuous-flow 12

5. Synthetic application 14

6. The mechanistic studies 15

6.1 Radical quenching experiments 15

6.2 Electron paramagnetic resonance (EPR) texts 16

6.3 Control experiment 18

6.4 Radical clock experiments 18

6.5 $K_2S_2O_8$-promoted remote heteroarylation 20

6.6 Cyclovoltammetric experiments 21

6.7 Stern-Volmer fluorescence quenching studies 27

6.8 Hydrogen evolution detection 29

6.9 Light on/off experiments 30

6.10 Density functional theory calculations 30

7. Characterization data for synthesized compounds 32

7.1 Characterization data for substrates 32

7.2 Characterization data for products 43

8. NMR spectra for synthesized compounds 86

8.1 NMR spectra for substrates 86

8.2 NMR spectra for products 123

9. Supplementary References 231

1. General information

The reagents and solvents were purchased from commercial suppliers and used without further purification unless noted. Chromatographic purification of products was performed by flash column chromatography on silica gel (200−300 meshes). Thin-layer chromatography (TLC) was carried out on silica plates (TLC Silica GF254). Visualization of the compounds was accomplished by projecting UV light onto the developed plates. The experiments under 450-460 nm light irradiation were performed using two 25 W JG LED lamps from Xuzhou Ai Jia Electronic Technology Co., Ltd. The distance from the light source to the irradiation vessel was approximate 2-3 cm, and no filter was used in our study. A fan was employed to ensure reactions remained at or near room temperature when using LED. ^{1}H (400 MHz or 600 MHz) NMR, ^{13}C (101 MHz or 151 MHz) NMR and ^{19}F (376 MHz) NMR spectra were recorded on a Varian spectrometer in $CDCl_3$ or DMSO-d_6 using tetramethylsilane (TMS) as internal standards. Data are reported as follows: Chemical shift (number of protons, multiplicity, coupling constants). Coupling constants were quoted to the nearest 0.1 Hz and multiplicity reported according to the following convention: s = singlet, d = doublet, t = triplet, q = quartet, hept = heptet, m = multiplet, dd = doublet of doublets, dt = doublet of triplets, td = triplet of doublets, tt = triplet of triplet, ddd = doublet of doublet of doublets, br s = broad singlet. HRMS spectra were recorded on a Bruker Impact II UHR-QTOF spectrometer using ESI on a TOF mass analyze. The EPR data were recorded with Bruker Emxplus. Fluorescence quenching experiments were performed on Hitachi F7000 FL Spectrophotometer. The cyclic voltammetry measurements were detected by using a CHI 600E electrochemical workstation. Hydrogen gas was analyzed by gas chromatography-thermal conductivity detector (GC-TCD, Agilent 7890B, nitrogen as the carrier gas).

2. General procedure

2.1 General procedure A: Preparation of *N*-protected amines substrates from free amines and sulfonyl chloride.

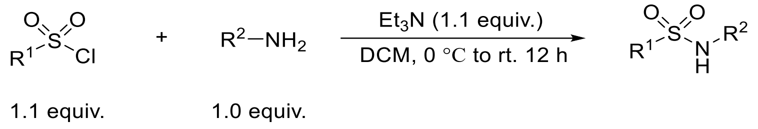

Free amine/ amine hydrochloride (5.0 mmol, 1.0 equiv.), and triethyl-amine (5.5 mmol, 1.1 equiv.) were dissolved in DCM (25 mL) at 0 °C. A DCM solution (5 mL) of corresponding sulfuryl chloride (5.5 mmol,1.1 equiv.) was added slowly over 5 minutes. After the addition was completed, the mixture was allowed to warm to room temperature and the solution was allowed to stir for 24 hours. The reaction mixture was quenched with water (5 mL) and 1.0 M HCl (11 mL). The aqueous phase was extracted with 10 mL DCM for three times. Combined organic phases were washed with brine (20 mL) and then dried with anhydrous Na_2SO_4, concentrated under reduced pressure. The desired product was afforded after purification by flash column chromatography on silica gel (PE/EtOAc).

2.2 General procedure B: Preparation of *N*-protected amines substrates from 4-methoxybenzenesulfonamide and alkyl bromide.

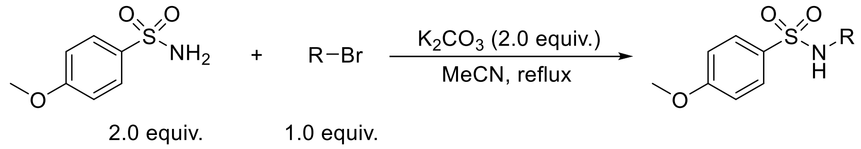

To a solution of alkyl bromide (5.0 mmol, 1.0 equiv.) in MeCN (20 mL) were added K_2CO_3 (10 mmol, 2 equiv.) and 4-methoxybenzenesulfonamide (10 mmol, 2.0 equiv.), and the reaction was heated to reflux for 8 h. After the reaction was completed, the reaction mixture was filtrated, washed with EtOAc (20 mL), and concentrated under reduced pressure. The desired product was afforded after purification by flash column chromatography on silica gel (PE/EtOAc).

2.3 General procedure C: Preparation of *N*-protected amines substrates from free alcohol intermediate and carboxylic acids

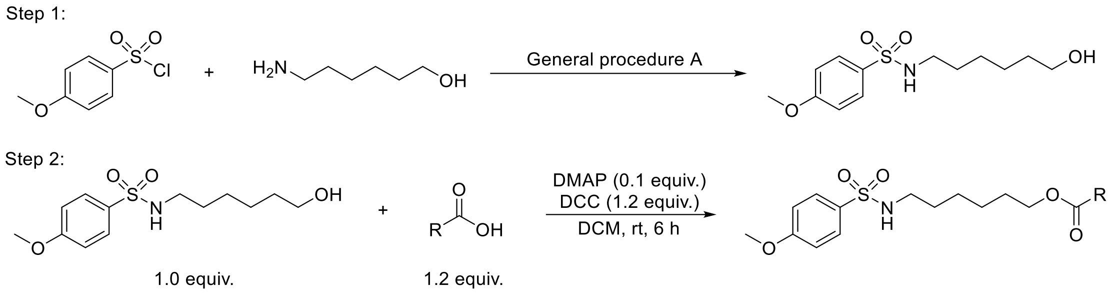

STEP 1 is consistent with the general procedure A.

STEP 2: A flame-dried round-bottomed flask was charged with *N*-(6-hydroxyhexyl)-4-methoxybenzenesulfonamide (1.0 mmol, 1.0 equiv.), carboxylic acid (1.2 mmol, 1.2 equiv.), DMAP (0.1 mmol, 0.1 equiv.), DCC (1.2 mmol, 1.0 equiv.) and dry DCM (5 mL). The reaction mixture was stirred at room temperature for 6 hours. The reaction mixture was then filtered, washed with DCM (20 mL) and concentrated under reduced pressure. The desired product was afforded after purification by flash column chromatography on silica gel (PE/EtOAc).

2.4 General procedure D: Preparation of *N*-protected amines substrates from free carboxylic acid intermediate and alcohols

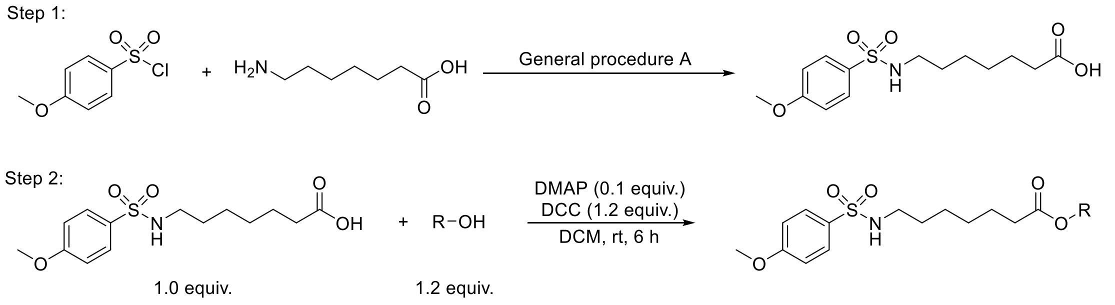

STEP 1 is consistent with the general procedure A.

STEP 2: A flame-dried round-bottomed flask was charged with 7-((4-methoxyphenyl)sulfonamido)heptanoic acid (1.0 mmol, 1.0 equiv.), alcohol (1.2 mmol, 1.2 equiv.), DMAP (0.1 mmol, 0.1 equiv.), DCC (1.2 mmol, 1.0 equiv.) and dry DCM (5 mL). The reaction mixture was stirred at room temperature for 6 hours. The reaction mixture was then filtered, washed with DCM (20 mL) and concentrated under reduced pressure. The desired product was afforded after purification by flash column chromatography on silica gel (PE/EtOAc).

2.5 General procedure E: Remote C(sp^3)–H heteroarylation of sulfonamides

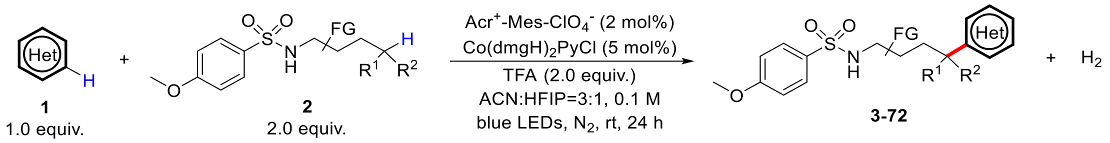

Photochemical Reaction Apparatus

Photochemical reaction was carried out under visible light irradiation by two 25 W blue lamps at room temperature.

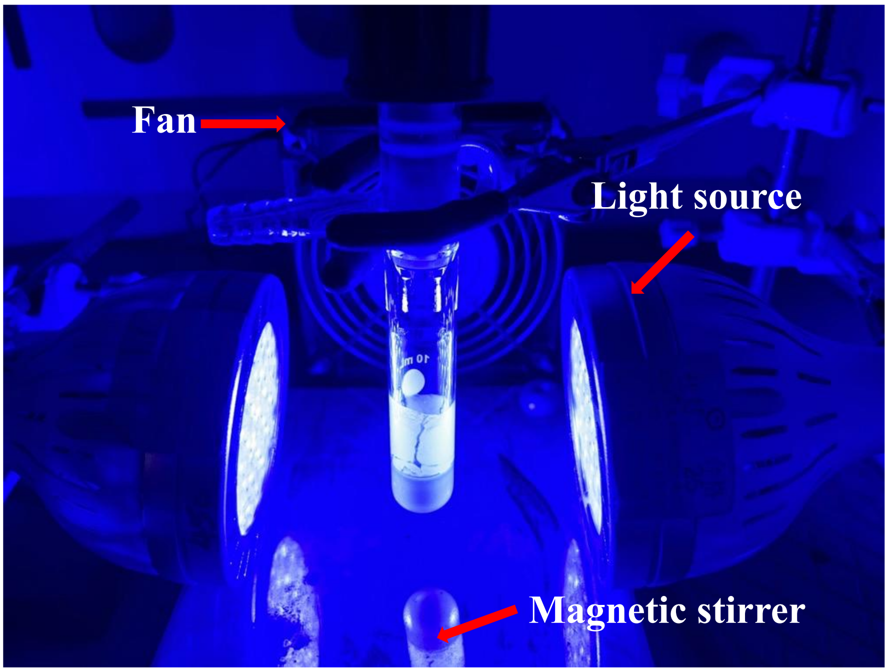

Supplementary Figure 1. Reaction set-up for the remote Minisci reaction

To a 10 mL Schlenk tube equipped with a magnetic stirring bar was added heteroarene **1** (0.2 mmol), *N*-protected amines substrates **2** (0.4 mmol), Acr^+-Mes-ClO_4^- (2 mol%) and Co(dmgH)$_2$PyCl (5 mol%). After three cycles of evacuation and backfilling of the reaction flask with nitrogen, TFA (2.0 equiv.), ACN (1.5 mL) and HFIP (0.5 mL) were added to the tube under nitrogen. The mixture was then irradiated by two 25 W blue lamps for 24 h. The reaction mixture was quenched by adding 4 mL saturated $NaHCO_3$ solution and 15 mL water and then extracted with ethyl acetate (3 × 20 mL). The combined organic extracts were washed by brine, dried over Na_2SO_4, filtered, concentrated under reduced pressure. The crude product was purified by column chromatography on silica gel to afford the desired product **3-72**.

2.6 General procedure F: Dehydrogenative C(sp^3)-H heteroarylation of alkanes

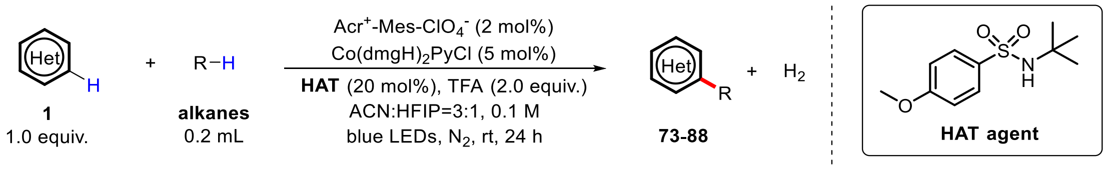

To a 10 mL Schlenk tube equipped with a magnetic stirring bar was added heteroarene **1** (0.2 mmol), Acr^+-Mes-ClO_4^- (2 mol%), Co(dmgH)$_2$PyCl (5 mol%) and *N*-(tert-butyl)-4-methoxybenzenesulfonamide (20 mol%). After three cycles of evacuation and backfilling of the reaction flask with nitrogen, TFA (2.0 equiv.), alkanes (0.2 mL), ACN (1.5 mL) and HFIP (0.5 mL) were added to the tube under nitrogen. The mixture was then irradiated by two 25 W blue lamps for 24 h. The reaction mixture was quenched by adding 4 mL saturated $NaHCO_3$ solution and 15 mL water and then extracted with ethyl acetate (3 × 20 mL). The combined organic extracts were washed by brine, dried over Na_2SO_4, filtered, concentrated under reduced pressure. The crude product was purified by column chromatography on silica gel to afford the desired product **73-88**.

2.7 General procedure G: Remote C(sp^3)–H functionalization of sulfonamides

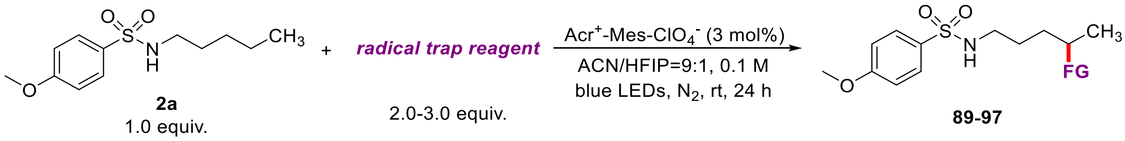

To a 10 mL Schlenk tube equipped with a magnetic stirring bar was added **2a** (0.2 mmol, 1.0 equiv.), radical trap reagent (0.4-0.6 mmol, 2.0-3.0 equiv.) and Acr^+-Mes-ClO_4^- (3 mol%). After three cycles of evacuation and backfilling of the reaction flask with nitrogen, ACN (1.8 mL) and HFIP (0.2 mL) was added to the tube under nitrogen. The mixture was then irradiated by two 25 W blue lamps for 24 h. The reaction mixture was quenched by adding 15 mL water and then extracted with ethyl acetate (3 × 20 mL). The combined organic extracts were washed by brine, dried over Na_2SO_4, filtered, concentrated under reduced pressure. The crude product was purified by column chromatography on silica gel to afford the desired product **89-97**.

2.8 Procedures for preparation of specific compounds

methyl (*S*)-2-((4-methoxyphenyl)sulfonamido)hexanoate

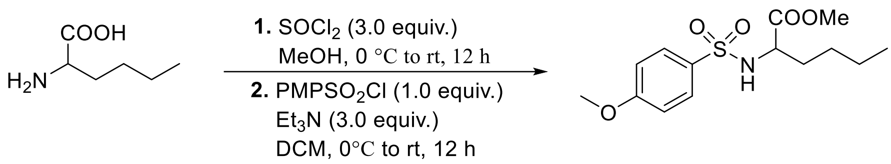

STEP 1: 2-Aminohexanoic acid (10 mmol, 1.0 equiv.) was suspended in methanol (100 mL) and cooled at 0 °C. Thionyl chloride (30 mmol, 3.0 equiv.) was added, the mixture was allowed to warm to room temperature and the solution was allowed to stir for 12 h. The solution was concentrated under reduced pressure to obtain crude amine hydrochloride.

SETP 2: The crude amine hydrochloride and Et_3N (30 mmol, 3.0 equiv.) were dissolved in DCM (50 mL) at 0 °C. A DCM solution (20 mL) of sulfuryl chloride (10 mmol, 1.0 equiv.) was added slowly over 5 minutes. After the addition was completed, the mixture was allowed to warm to room temperature and the solution was allowed to stir for 12 h. The reaction mixture was quenched with water (10 mL) and 1.0 M HCl (10 mL). The aqueous phase was extracted with 20 mL DCM for three times. Combined organic phases were dried with anhydrous Na_2SO_4, concentrated under reduced pressure. The crude product was purified by flash chromatography on silica gel (PE/ EtOAc =2.5/1).

***N*-(6-chlorohexyl)-4-methoxybenzenesulfonamide**

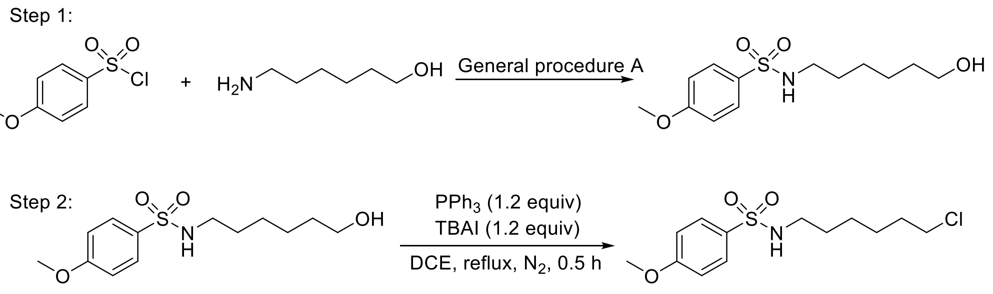

STEP 1 is consistent with the general procedure A.

STEP 2: In a 25 mL Schlenk flask, *N*-(6-hydroxyhexyl)-4-methoxybenzenesulfonamide (2.0 mmol, 1.0 equiv.), triphenylphosphine (2.4 mmol, 1.2 equiv.), tetrabutylammonium iodide (2.4 mmol, 1.2 equiv.), and dry 1,2-dichloroethane (20 mL) were added under a N_2 atmosphere. The mixture was heated to reflux for 0.5 hours. After the mixture was cooled to room temperature, the solvent was removed by concentration under reduced pressure. The residue was purified by flash chromatography on silica gel (PE/EtOAc = 3/1) to give the desired product (90% yield).

***N*-(6-(1,1-dioxido-3-oxobenzo[*d*]isothiazol-2(3*H*)-yl)hexyl)-4-methoxybenzenesulfonamide**

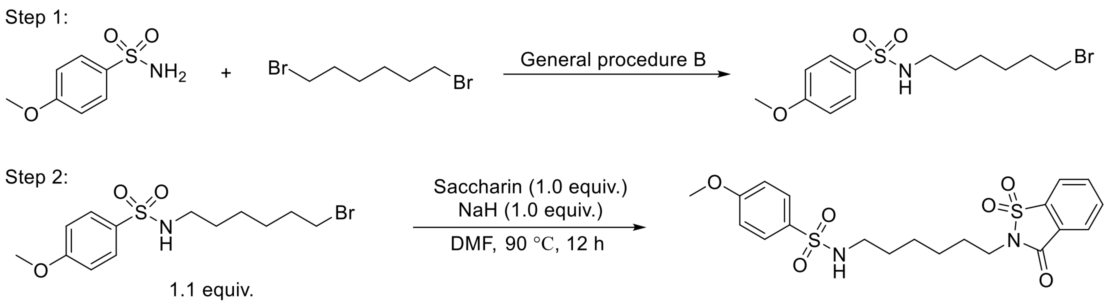

STEP 1 is consistent with the general procedure B.

STEP 2: To a solution of Saccharin (1.0 eq. 5 mmol) in dry DMF was added sodium hydride (1.0 equiv., 60% mineral oil dispersion) at 0 °C. The mixture was allowed to warm to room temperature and stirred for 30 minutes and then cooled to 0 °C before *N*-(6-bromohexyl)-4-methoxybenzenesulfonamide (2.2 mmol, 1.1 equiv.) was added. The mixture was allowed to come to room temperature and then heated at 90 °C for 12 hours. The reaction mixture was quenched by adding saturated aqueous ammonium chloride solution and then extracted with EtOAc for three times. Combined organic phases were washed with brine, dried with anhydrous Na_2SO_4 and concentrated under reduced pressure. The crude product was purified by flash chromatography on silica gel (PE/EtOAc = 3/1) to give the desired product.

<u>*N*-(6-((4-methoxyphenyl)sulfonamido)hexyl)-*N*-methyl-4-(5-(*p*-tolyl)-3-(trifluoromethyl)-1*H*-pyrazol-1-yl)benzenesulfonamide</u>

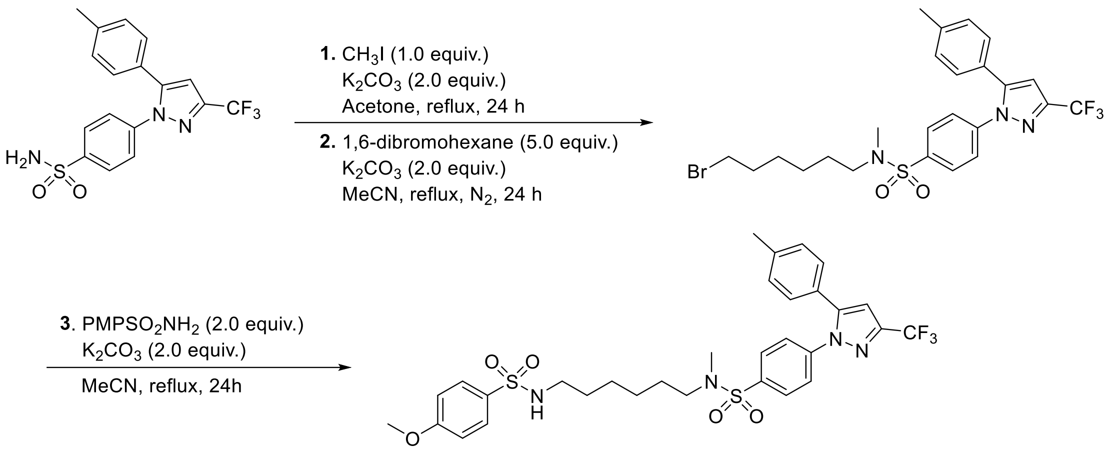

STEP1: Under N_2 atmosphere, to a solution of Celecoxib (5.0 mmol, 1.0 equiv.) and CH_3I (7.5 mmol, 1.5 equiv.)) in acetone (25.0 mL) was added K_2CO_3 (10 mmol. 2.0 equiv.) at room temperature. Then the reaction mixture was heated to reflux for 24 hours. After cooling to room temperature, the reaction mixture was filtered through a pad of Celite and concentrated under

reduced pressure. The residue was purified by flash chromatography on silica gel (PE/EtOAc = 5/1) to afford *N*-methyl-Celecoxib as a white solid (90% yield).

STEP2: Under N_2 atmosphere, to a solution of *N*-methyl-Celecoxib (4.5 mmol, 1.0 equiv.) and 1,6-dibromohexane (22.5 mmol, 5.0 equiv.) in anhydrous CH_3CN (20 mL) was added K_2CO_3 (13.5 mmol 3.0 equiv.) at room temperature. Then the reaction mixture was heated for 24 hours. After cooling to room temperature, the reaction mixture was filtered through a pad of Celite and concentrated under reduced pressure. The residue was purified by flash chromatography on silica gel (PE/EtOAc = 10/1) to afford the alkyl bromide intermediate as a white solid (76% yield).

STEP3: Alkyl bromide intermediate (2.0 mmol, 1.0 equiv.), 4-methoxybenzenesulfonamide (4.0 mmol, 2.0 equiv.), K_2CO_3 (4.0 mmol, 2.0 equiv.) and MeCN (10 mL) were added into a round-bottom flask. Then the reaction mixture was heated to reflux for 24 hours. After being cooled to room temperature, the reaction mixture was concentrated under reduced pressure, 5 mL EA was added to the residue and insoluble was filtered off. The solution was concentrated under reduced pressure. The crude product was purified by flash chromatography on silica gel (PE/EtOAc = 3/1) to give the desired product (46% yield).

<u>*N*-(4-(3-chloroquinoxalin-2-yl)pentyl)-4-methoxybenzenesulfonamide</u>

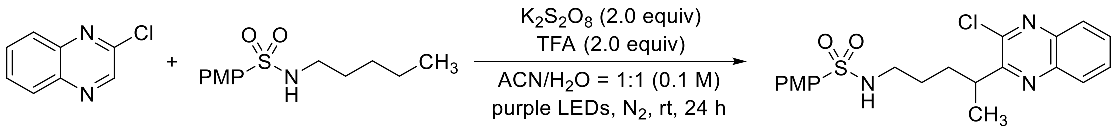

37

To a 10 mL Schlenk tube equipped with a magnetic stirring bar was added 2-chloroquinoxaline (0.2 mmol), 4-methoxy-*N*-pentylbenzenesulfonamide **2a** (0.4 mmol), $K_2S_2O_8$ (2.0 equiv). After three cycles of evacuation and backfilling of the reaction flask with nitrogen, TFA (2.0 equiv.), ACN (1.0 mL) and H_2O (1.0 mL) were added to the tube under nitrogen. The mixture was then irradiated by two 25 W purple lamps (λ =390−400 nm) for 24 h. The reaction mixture was quenched by adding 6 mL saturated $NaHCO_3$ solution and 15 mL water and then extracted with ethyl acetate (3 × 20 mL). The combined organic extracts were washed by brine, dried over Na_2SO_4, filtered, concentrated under reduced pressure. The crude product was purified by column chromatography on silica gel to afford the desired product **37**.

<u>4-methoxy-*N*-(pentyl-4-*d*)benzenesulfonamide</u>

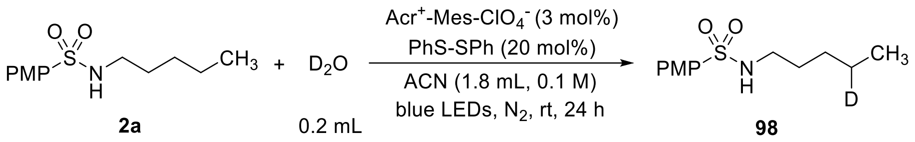

To a 10 mL Schlenk tube equipped with a magnetic stirring bar was added 4-methoxy-*N*-pentylbenzenesulfonamide **2a** (0.2 mmol), Acr^{+}-Mes-ClO_4^{-} (3 mol%) and PhS-SPh (20 mol%). After three cycles of evacuation and backfilling of the reaction flask with nitrogen, D_2O (0.2 mL) and ACN (1.8 mL) were added to the tube under nitrogen. The mixture was then irradiated by two 25 W blue lamps for 24 h. The reaction mixture was concentrated under reduced pressure. The crude product was purified by column chromatography on silica gel to afford the desired product **98**.

3. Unsuccessful substrates

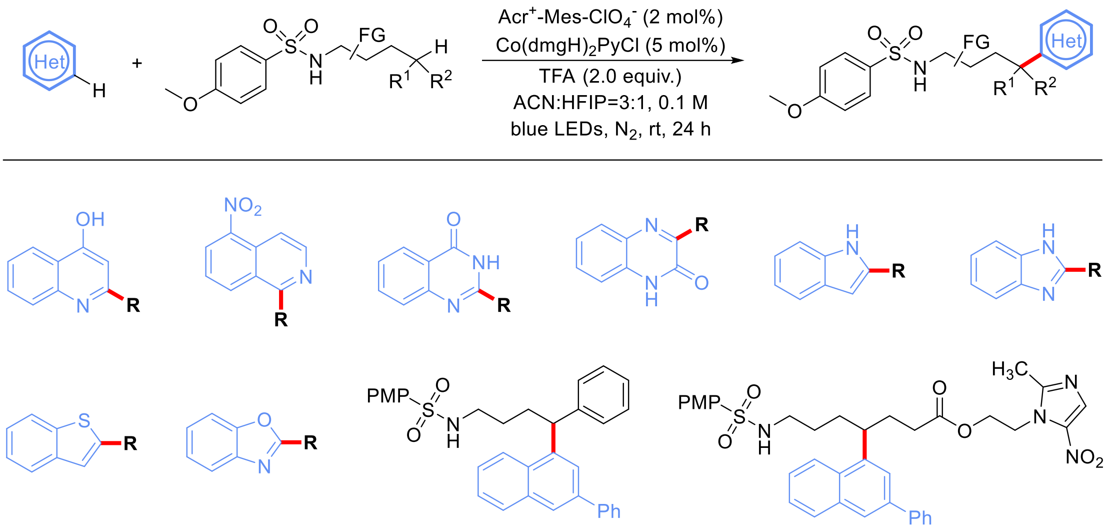

Supplementary Figure 2. Unsuccessful substrates. The listed substrates were suffered from decomposition or low reactivity under optimal conditions, which did not undergo further optimizations.

4. Gram-scale experiments

4.1 Gram-scale experiments in batch

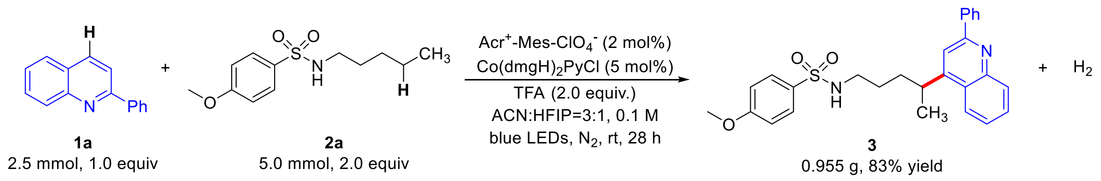

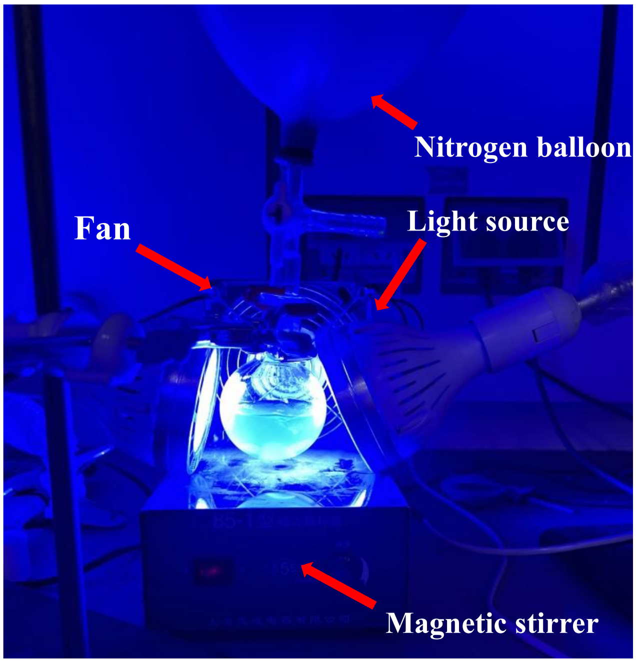

Supplementary Figure 3. Reaction set-up for the gram-scale experiment in batch

A flame-dried round-bottomed flask was charged with 2-phenylquinoline **1a** (2.5 mmol, 1.0 equiv.), 4-methoxy-*N*-pentylbenzenesulfonamide **2a** (5.0 mmol, 2.0 equiv.), Acr^{+}-Mes-ClO_4^{-} (0.05 mmol, 2 mol%) and Co(dmgH)$_2$PyCl (0.125 mmol, 5 mol%). After three cycles of evacuation and backfilling of the reaction flask with nitrogen, TFA (5.0 mmol, 2.0 equiv.), ACN (19.0 mL) and HFIP (6.0 mL) were added to the flask under nitrogen. The mixture was then irradiated by two 25 W blue lamps for 28 h. The reaction mixture was quenched by adding 20 mL saturated $NaHCO_3$ solution and 50 mL water and then extracted with ethyl acetate (3 × 50 mL). The combined organic extracts were washed by brine, dried over Na_2SO_4, filtered, concentrated under reduced pressure. The crude product was purified by column chromatography on silica gel to afford the desired product **3** in 83% yield.

4.2 Gram-scale experiments in continuous-flow

Nitrogen was pumped into the pipeline to remove the air. Under nitrogen atmosphere, a two-neck round bottom flask was equipped with a rubber septum and magnetic stir bar and charged with heteroarene (2.5 mmol, 1.0 equiv.), 4-methoxy-*N*-pentylbenzenesulfonamide **2a** (5.0 mmol, 2.0 equiv.), Acr^{+}-Mes-ClO_4^{-} (0.05 mmol, 2 mol%) and Co(dmgH)$_2$PyCl (0.125 mmol, 5 mol%). The resulting mixture was sealed and degassed via vacuum evacuation and back-filled with nitrogen gas three times. The TFA (5.0 mmol, 2.0 equiv.), ACN (19.0 mL) and HFIP (6.0 mL) were then added

to the mixture. The round bottom flask was warpped with tin foil to avoid light. The microtubing reactor (PFA, O.D. = 1/16", I.D. = 1 mm", 10.5 m, volume = 8.3 mL) was placed under 4×25 W and 1×40 W blue LEDs at 40-45 °C for 103 min. After the reaction was completed, 10.0 mL acetonitrile was pumped to wash the microtubing reactor. About 35.0 mL of solution was pumped out from the microtubing reactor. The reaction mixture was quenched by adding 20 mL saturated $NaHCO_3$ solution and 50 mL water and then extracted with ethyl acetate (3 × 50 mL). The combined organic extracts were washed by brine, dried over Na_2SO_4, filtered, concentrated under reduced pressure. The crude product was purified by column chromatography on silica gel to afford the desired product **3** in 78% yield.

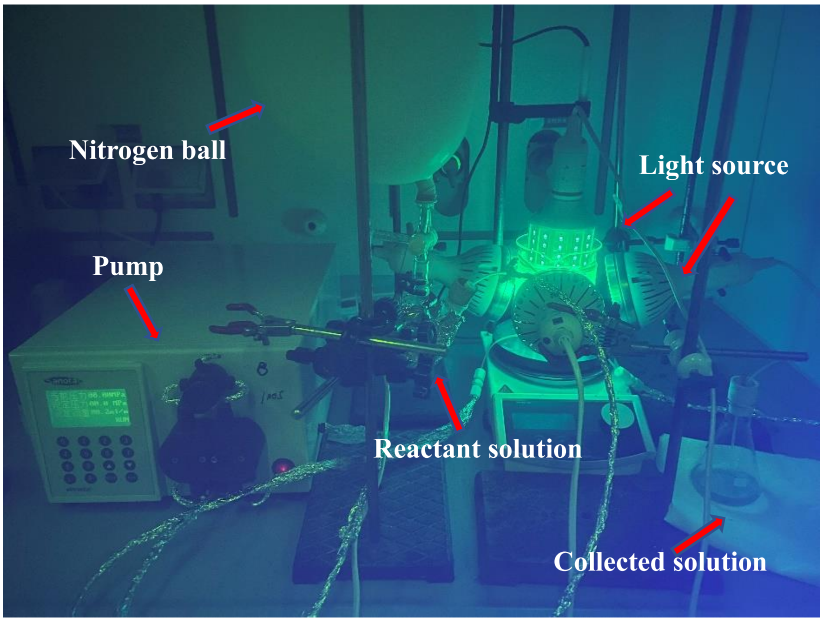

Supplementary Figure 4. Reaction set-up for the gram-scale experiment in continuous-flow

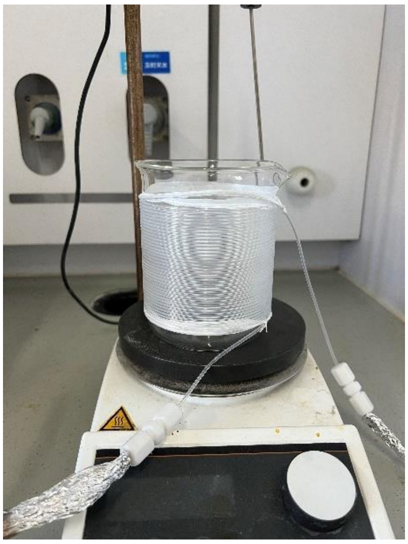

Supplementary Figure 5. Microtubing reactor setup

5. Synthetic application

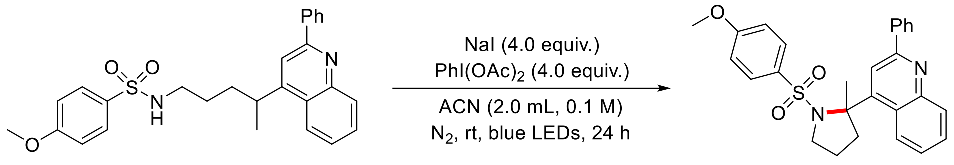

To a 10 mL Schlenk tube equipped with a magnetic stirring bar was added compound **3** (0.2 mmol, 1.0 equiv.), NaI (0.8 mmol, 4.0 equiv.) and PhI(OAc)$_2$ (0.8 mmol, 4.0 equiv.). After three cycles of evacuation and backfilling of the reaction flask with nitrogen, ACN (2.0 mL) was added to the tube under nitrogen. The mixture was then irradiated by two 25 W blue lamps for 24 h. The reaction mixture was quenched by adding 10 mL saturated sodium thiosulfate solution and then extracted with ethyl acetate (3 × 10 mL). The combined organic extracts were washed by brine, dried over Na_2SO_4, filtered, concentrated under reduced pressure. The crude product was purified by column chromatography on silica gel to afford the desired product **99** in 29% yield.

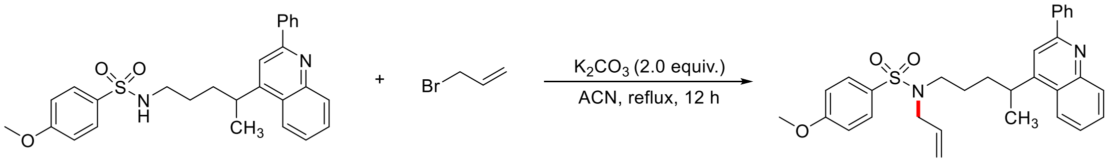

To a solution of 3-bromoprop-1-ene (0.4 mmol, 2.0 equiv.) in MeCN (2.0 mL) was added compound **3** (0.2 mmol, 1.0 equiv.) and K_2CO_3 (0.4 mmol, 2.0 equiv.). The reaction mixture was heated to reflux for 12 hours. After cooling to room temperature, the reaction mixture was filtered through a pad of Celite and concentrated under reduced pressure. The residue was purified by flash chromatography on silica gel to afford the desired product **100** in 83% yield.

6. The mechanistic studies

6.1 Radical quenching experiments

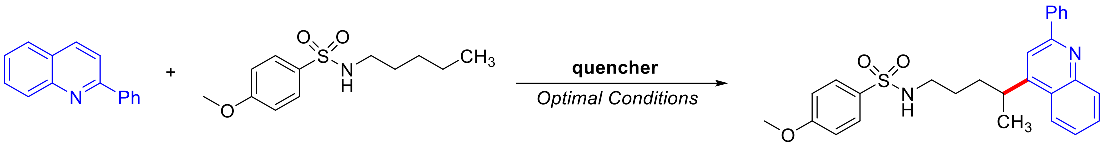

1a, 0.2 mmol | **2a**, 0.4 mmol | **quencher** (2.0 equiv) | **3**

quencher (2.0 equiv)	3
TEMPO	N.R.
BHT	41%

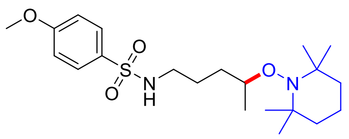

101, detected by HRMS
[M+H]$^+$: $C_{21}H_{37}N_2O_4S^+$
Calcd for: 413.2469
found 413.2488

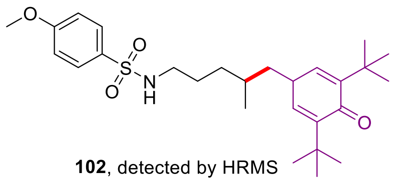

102, detected by HRMS
[M+Na]$^+$: $C_{27}H_{41}NNaO_4S^+$
Calcd for: 498.2649
found 498.2654

To a 10 mL Schlenk tube equipped with a magnetic stirring bar was added 2-phenylquinoline **1a** (0.2 mmol, 1.0 equiv.), 4-methoxy-*N*-pentylbenzenesulfonamide **2a** (0.4 mmol, 2.0 equiv.), Acr$^+$-Mes-ClO_4^- (2 mol%), Co(dmgH)$_2$PyCl (5 mol%) and radical quencher (TEMPO or BHT, 0.4 mmol, 2.0 equiv.). After three cycles of evacuation and backfilling of the reaction flask with nitrogen, TFA (2.0 equiv.), ACN (1.5 mL) and HFIP (0.5 mL) were added to the tube under nitrogen. The mixture was then irradiated by two 25 W blue lamps for 24 h. The reaction was completely inhibited in the presence of TEMPO, and the desired product **3** was isolated in the yield of 41% in the presence of BHT. In addition, radical adducts **101** and **102** were detected by ESI-HRMS in the case of TEMPO and BHT, respectively, which suggested that the reaction proceed through a radical involved pathway.

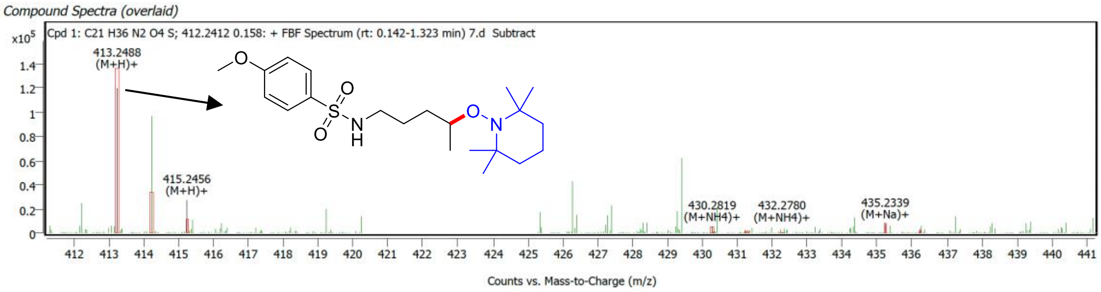

Supplementary Figure 6. The HRMS analysis of radical quenching experiment in the presence of TEMPO

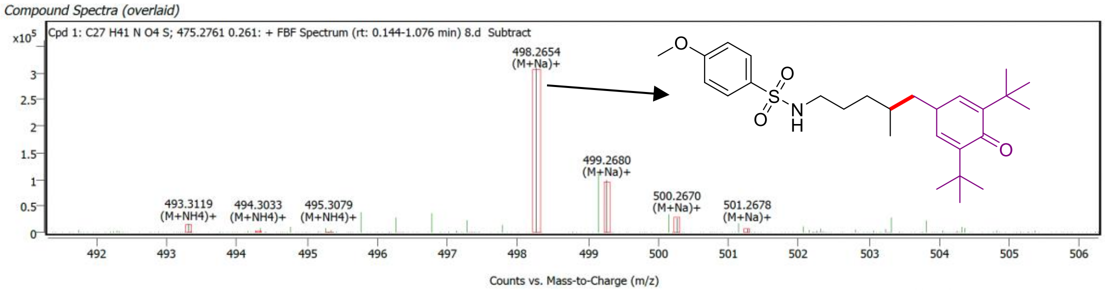

Supplementary Figure 7. The HRMS analysis of radical quenching experiment in the presence of BHT

6.2 Electron paramagnetic resonance (EPR) texts

The electron paramagnetic resonance (EPR) experiments were carried out to determination of remote C-centred radical. EPR spectra was recorded at room temperature on Bruker Emxplus spectrometer. Typical spectrometer parameters were shown as follows, center field: 3505.00 G, frequency mon: 9.843483 GHz, sweep width: 100.0 G, power: 3.170 mW, power atten: 18.0 dB, modulation amplitude: 1.000 G, modulation frequency: 100.00 kHz, sweep time: 60.00 s, time constant: 5.12 ms.

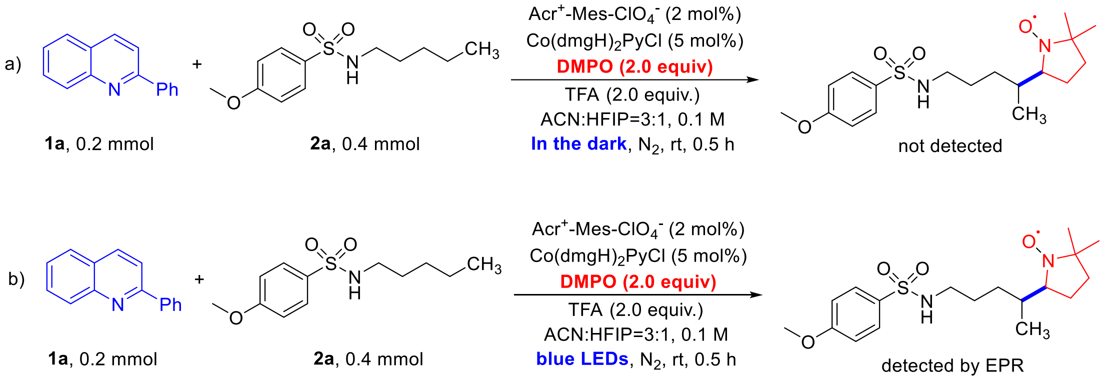

a): To a 10 mL Schlenk tube equipped with a magnetic stirring bar was added 2-phenylquinoline **1a** (0.2 mmol, 1.0 equiv.), 4-methoxy-*N*-pentylbenzenesulfonamide **2a** (0.4 mmol, 2.0 equiv.), Acr^{+}-Mes-ClO_4^{-} (2 mol%) and $Co(dmgH)_2PyCl$ (5 mol%). After three cycles of evacuation and backfilling of the reaction flask with nitrogen, TFA (2.0 equiv.), ACN (1.5 mL) and HFIP (0.5 mL) were added to the tube under nitrogen. The mixture was stirred in the dark for 0.5 h. Then the 5,5-dimethyl-1-pyrroline-*N*-oxide (DMPO, 0.4 mmol, 2.0 equiv.) was added into the tube and reacted for 1 min. The mixture was put into a simple melting point tube for EPR test. As shown in **Figure**

S8, any useful information could not be detected by EPR spin-trapping experiment with DMPO.

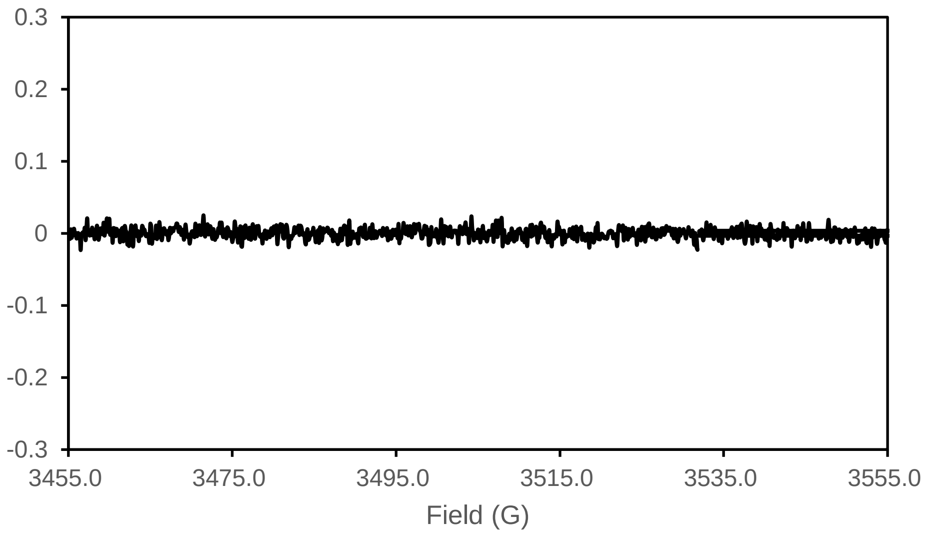

Supplementary Figure 8. EPR spin-trapping results of experiment **a**

b): To a 10 mL Schlenk tube equipped with a magnetic stirring bar was added 2-phenylquinoline **1a** (0.2 mmol, 1.0 equiv.), 4-methoxy-*N*-pentylbenzenesulfonamide **2a** (0.4 mmol, 2.0 equiv.), Acr^+-Mes-ClO_4^- (2 mol%) and $Co(dmgH)_2PyCl$ (5 mol%). After three cycles of evacuation and backfilling of the reaction flask with nitrogen, TFA (2.0 equiv.), ACN (1.5 mL) and HFIP (0.5 mL) were added to the tube under nitrogen. The mixture was then irradiated by 450-460 nm blue lamps for 0.5 h. Then the 5,5-dimethyl-1-pyrroline-*N*-oxide (DMPO, 0.4 mmol, 2.0 equiv.) was added into the tube and reacted for 1 min. The mixture was put into a simple melting point tube for EPR test. As shown in **Figure S9**, a C-centred radical trapped by DMPO was detected.

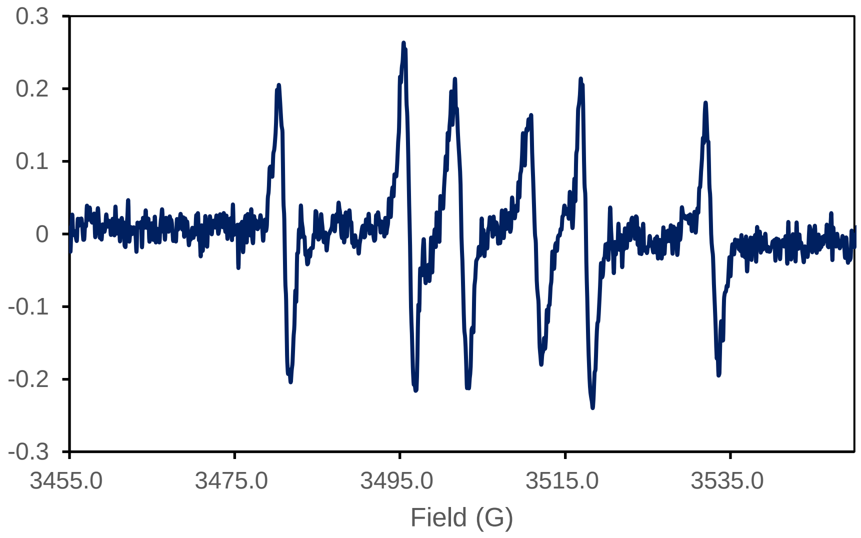

Supplementary Figure 9. The hyperfine structure with DMPO

6.3 Control experiment

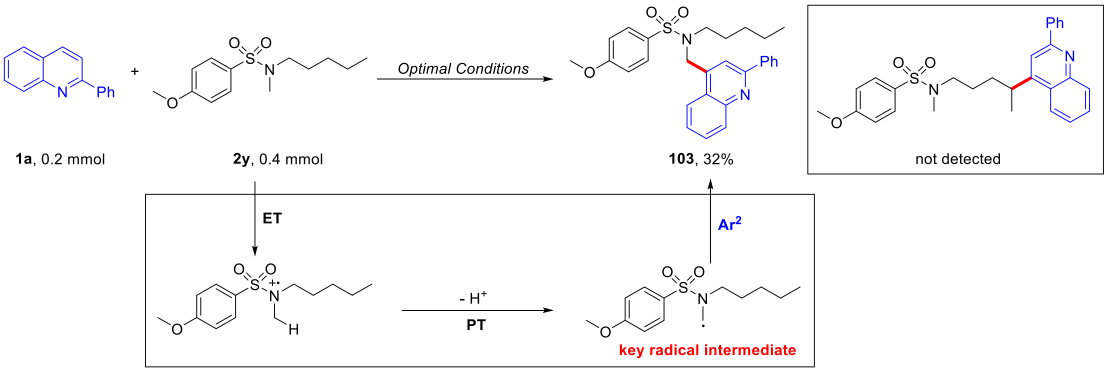

To a 10 mL Schlenk tube equipped with a magnetic stirring bar was added 2-phenylquinoline **1a** (0.2 mmol, 1.0 equiv.), 4-methoxy-*N*-methyl-*N*-pentylbenzenesulfonamide **2y** (0.4 mmol, 2.0 equiv.), Acr^{+}-Mes-ClO_4^{-} (2 mol%) and $Co(dmgH)_2PyCl$ (5 mol%). After three cycles of evacuation and backfilling of the reaction flask with nitrogen, TFA (2.0 equiv.), ACN (1.5 mL) and HFIP (0.5 mL) were added to the tube under nitrogen. The mixture was then irradiated by 450-460 nm blue lamps for 24 h. The reaction mixture was quenched by adding 4 mL saturated $NaHCO_3$ solution and 15 mL water and then extracted with ethyl acetate (3 × 20 mL). The combined organic extracts were washed by brine, dried over Na_2SO_4, filtered, concentrated under reduced pressure. The crude product was purified by column chromatography on silica gel to afford the desired product **103** in a yield of 32%. However, the remote heteroarylation product was not observed.

6.4 Radical clock experiments

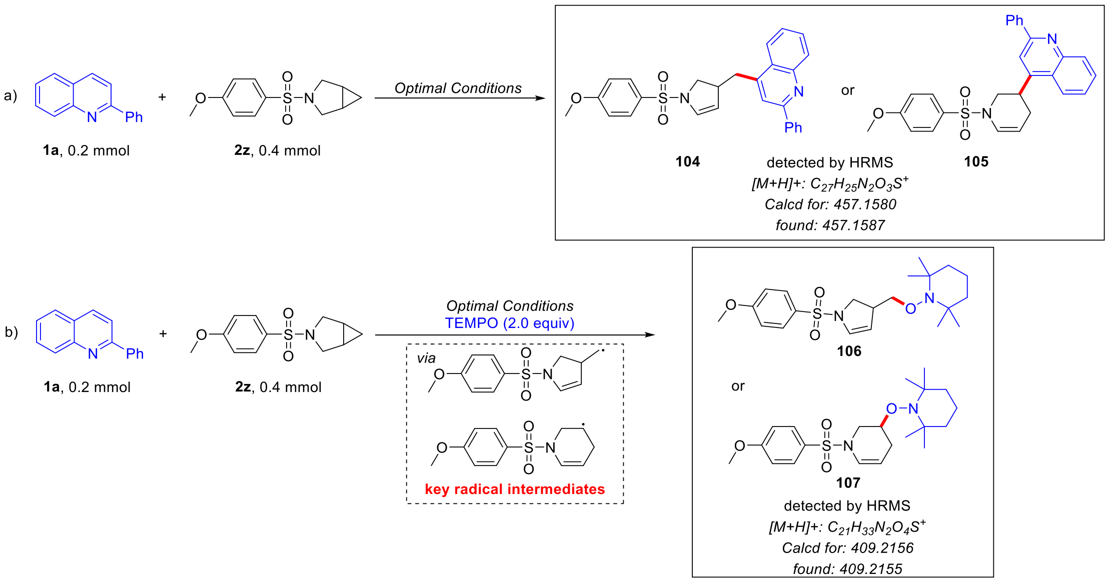

a) To a 10 mL Schlenk tube equipped with a magnetic stirring bar was added 2-phenylquinoline **1a**

(0.2 mmol, 1.0 equiv.), 3-((4-methoxyphenyl)sulfonyl)-3-azabicyclo[3.1.0]hexane **2z** (0.4 mmol, 2.0 equiv.), Acr$^+$-Mes-ClO$_4^-$ (2 mol%) and Co(dmgH)$_2$PyCl (5 mol%). After three cycles of evacuation and backfilling of the reaction flask with nitrogen, TFA (2.0 equiv.), ACN (1.5 mL) and HFIP (0.5 mL) were added to the tube under nitrogen. The mixture was then irradiated by two 25 W blue lamps for 24 h. After the reaction was completed, the mixture was put into a simple melting point tube for HRMS test. As expected, the molecular peak of the desired products **104** or **105** was detected by high-resolution mass spectrometry (**Figure S10**).

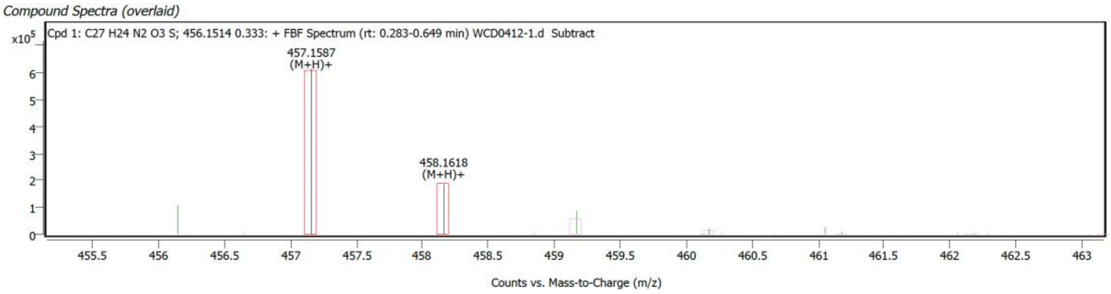

Supplementary Figure 10. The HRMS analysis of the product **104** or **105** in radical clock experiment **a**

b) To a 10 mL Schlenk tube equipped with a magnetic stirring bar was added 2-phenylquinoline **1a** (0.2 mmol, 1.0 equiv.), 3-((4-methoxyphenyl)sulfonyl)-3-azabicyclo[3.1.0]hexane **2z** (0.4 mmol, 2.0 equiv.), Acr$^+$-Mes-ClO$_4^-$ (2 mol%), Co(dmgH)$_2$PyCl (5 mol%) and TEMPO (0.4 mmol, 2.0 equiv.). After three cycles of evacuation and backfilling of the reaction flask with nitrogen, TFA (2.0 equiv.), ACN (1.5 mL) and HFIP (0.5 mL) were added to the tube under nitrogen. The mixture was then irradiated by two 25 W blue lamps for 24 h. After the reaction was completed, the mixture was put into a simple melting point tube for HRMS test. As expected, not only the molecular peak of the desired product **104** or **105**, but also the molecular peak of the radical adducts **106** or **107** were detected by high-resolution mass spectrometry (**Figure S11** and **S12**).

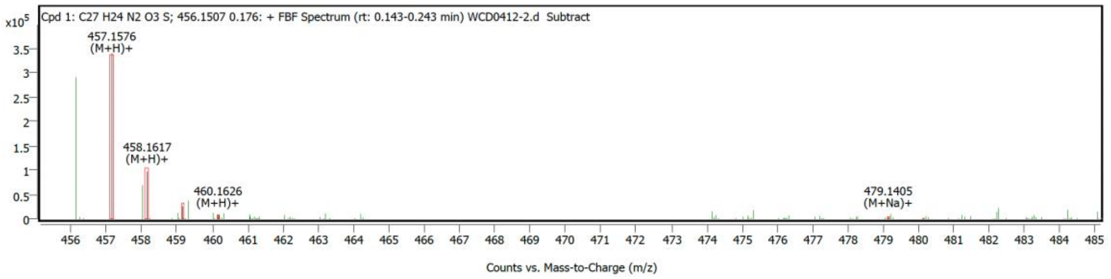

Supplementary Figure 11. The HRMS analysis of the product **104** or **105** in radical clock experiment **b**

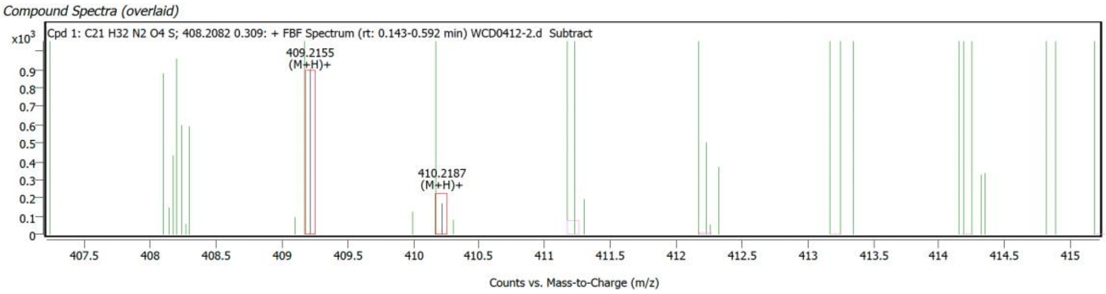

Supplementary Figure 12. The HRMS analysis of the radical adducts **106** or **107** in radical clock experiment **b**

6.5 $K_2S_2O_8$-promoted remote heteroarylation

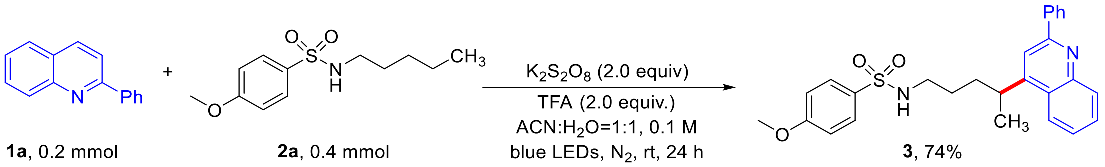

To a 10 mL Schlenk tube equipped with a magnetic stirring bar was added 2-phenylquinoline **1a** (0.2 mmol, 1.0 equiv.), 4-methoxy-*N*-pentylbenzenesulfonamide **2a** (0.4 mmol, 2.0 equiv.) and $K_2S_2O_8$ (2.0 equiv). After three cycles of evacuation and backfilling of the reaction flask with nitrogen, TFA (2.0 equiv.), ACN (1.0 mL) and H_2O (1.0 mL) were added to the tube under nitrogen. The mixture was then irradiated by 450-460 nm blue lamps for 24 h. The reaction mixture was quenched by adding 4 mL saturated $NaHCO_3$ solution and 15 mL water and then extracted with ethyl acetate (3 × 20 mL). The combined organic extracts were washed by brine, dried over Na_2SO_4, filtered, concentrated under reduced pressure. The crude product was purified by column chromatography on silica gel to afford the desired product **3** in 74% yield.

The mechanism was proposed as followed. Initially, $SO_4^{\bullet-}$ ($E_{p/2}$ = +2.5-3.0 V) was generated through homolytic cleavage of $S_2O_8^{2-}$ by means of light irradiation, which could remove an electron from neutral *N*-alkylsulfonamide **2a** to afford the sulfonamide radical cations **I**. The so-formed sulfonamide radical cations **I** then underwent deprotonation to afford *N*-centred radicals **II**, which could trigger intramolecular remote HAT through a cyclic transition state to afford the distal C-centred radicals **III**. The intermediate **III** then undergo addition on the protonated heteroarene **1** in a Minisci-type pathway to afford the amine radical cation **IV**, which then was oxidized by $SO_4^{\bullet-}$ to give the desired product **3-H$^+$**.

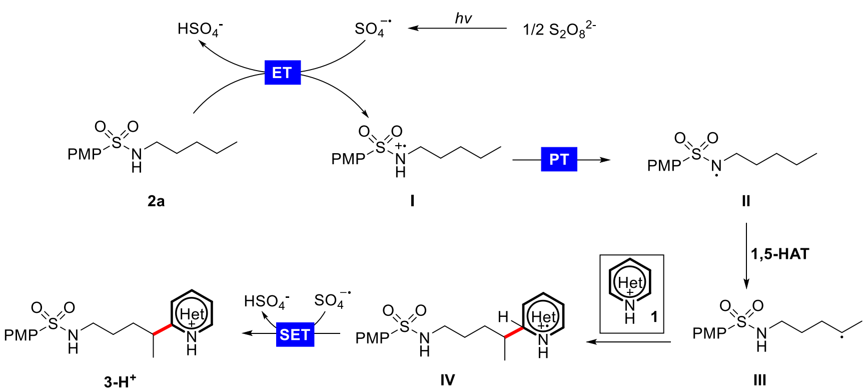

Supplementary Figure 13. The plausible mechanism of $K_2S_2O_8$-promoted remote heteroarylation

6.6 Cyclovoltammetric experiments

The cyclic voltammetry measurements were detected by using a CHI 600E electrochemical workstation. Electrochemical measurements (cyclic voltammetry) were performed in a three-electrode cell (10 mL) and acetonitrile was used as solvent. As for the electrolyte, it was used in a 0.1 M concentration, while the tested compounds were used in a 10 mM concentration. Electrodes: a 6 mm-diameter glassy carbon as the working electrode, Pt wire as the counter electrode and Ag/AgCl (sat'd KCl) as the reference electrode. Scan speed was 100 mV/s.

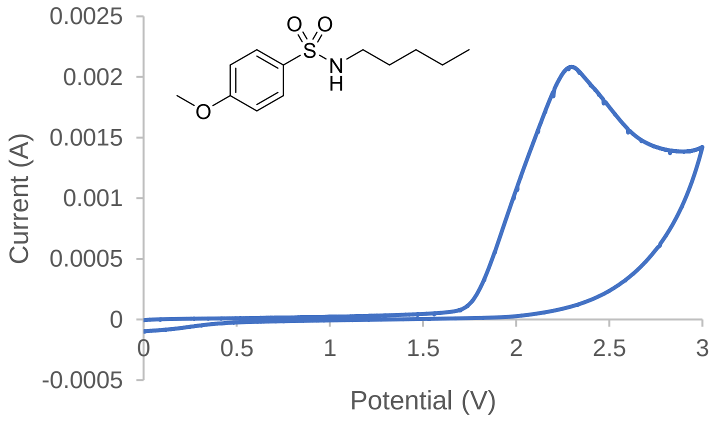

Supplementary Figure 14. The CV data of 4-methoxy-*N*-pentylbenzenesulfonamide in ACN. Conditions: a 6 mm-diameter glassy carbon as working electrode, Pt wire as the counter electrode and Ag/AgCl (sat'd KCl) as the reference electrode. Scan speed was 100 mV/s, room temperature. It was found that the half-peak potential of 4-methoxy-*N*-pentylbenzenesulfonamide in ACN was observed at 1.95 V (vs. SCE in ACN).

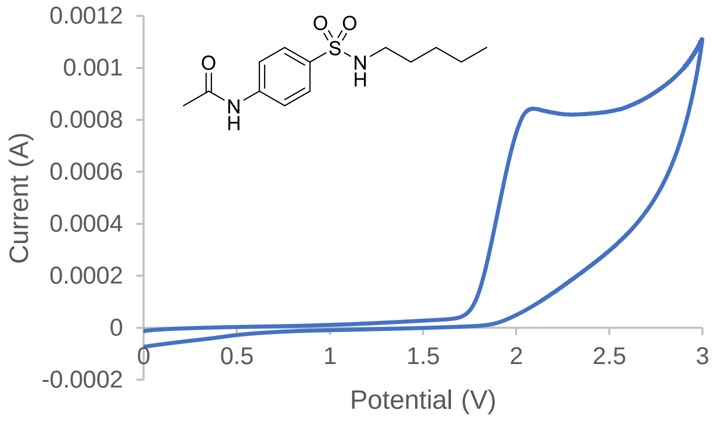

Supplementary Figure 15. The CV data of *N*-(4-(*N*-pentylsulfamoyl)phenyl)acetamide in ACN. Conditions: a 6 mm-diameter glassy carbon as working electrode, Pt wire as the counter electrode and Ag/AgCl (sat'd KCl) as the reference electrode. Scan speed was 100 mV/s, room temperature. It was found that the half-peak potential of 4-methoxy-*N*-pentylbenzenesulfonamide in ACN was observed at 1.89 V (vs. SCE in ACN).

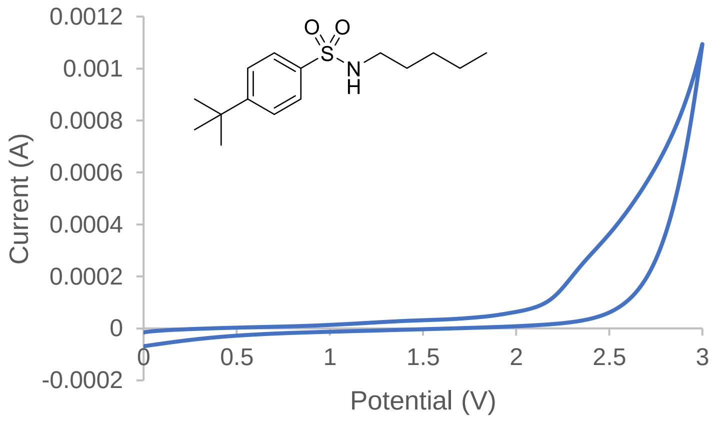

Supplementary Figure 16. The CV data of 4-(*tert*-butyl)-*N*-pentylbenzenesulfonamide in ACN. Conditions: a 6 mm-diameter glassy carbon as working electrode, Pt wire as the counter electrode and Ag/AgCl (sat'd KCl) as the reference electrode. Scan speed was 100 mV/s, room temperature. The results showed that no redox features were displayed between 0 and 3.0 V.

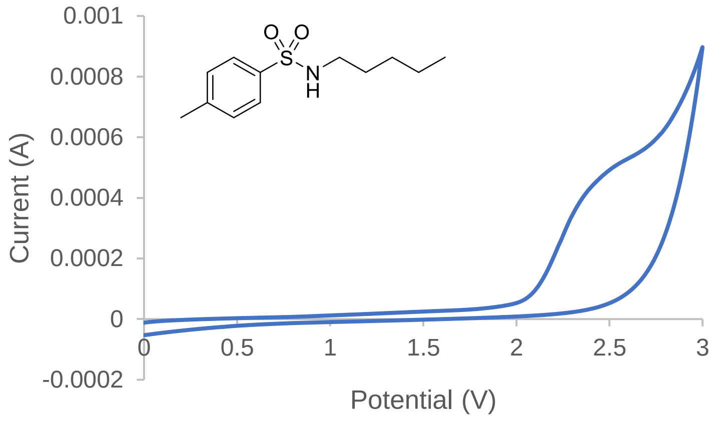

Supplementary Figure 17. The CV data of 4-methyl-*N*-pentylbenzenesulfonamide in ACN. Conditions: a 6 mm-diameter glassy carbon as working electrode, Pt wire as the counter electrode and Ag/AgCl (sat'd KCl) as the reference electrode. Scan speed was 100 mV/s, room temperature. It was found that the half-peak potential of 4-methyl-*N*-pentylbenzenesulfonamide in ACN was observed at 2.23 V (vs. SCE in ACN).

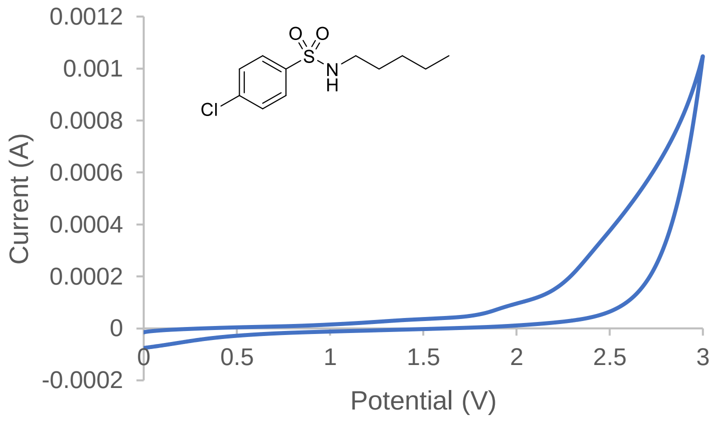

Supplementary Figure 18. The CV data of 4-chloro-*N*-pentylbenzenesulfonamide in ACN. Conditions: a 6 mm-diameter glassy carbon as working electrode, Pt wire as the counter electrode and Ag/AgCl (sat'd KCl) as the reference electrode. Scan speed was 100 mV/s, room temperature. The results showed that no redox features were displayed between 0 and 3.0 V.

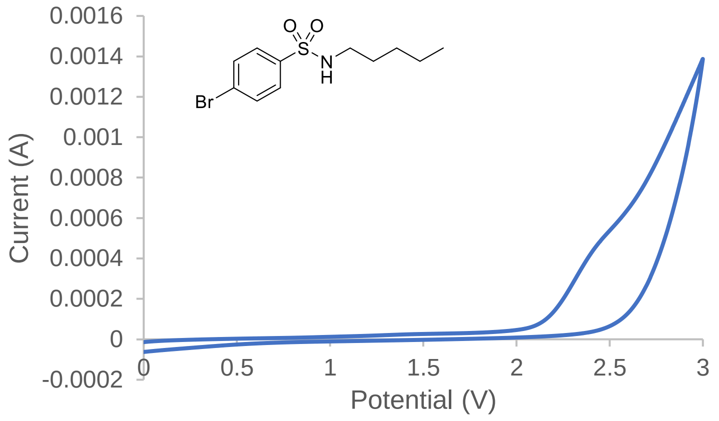

Supplementary Figure 19. The CV data of 4-bromo-*N*-pentylbenzenesulfonamide in ACN. Conditions: a 6 mm-diameter glassy carbon as working electrode, Pt wire as the counter electrode and Ag/AgCl (sat'd KCl) as the reference electrode. Scan speed was 100 mV/s, room temperature. The results showed that no redox features were displayed between 0 and 3.0 V.

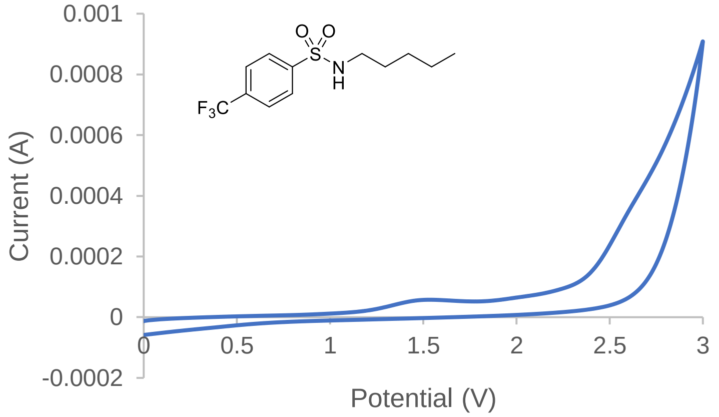

Supplementary Figure 20. The CV data of *N*-pentyl-4-(trifluoromethyl)benzenesulfonamide in ACN. Conditions: a 6 mm-diameter glassy carbon as working electrode, Pt wire as the counter electrode and Ag/AgCl (sat'd KCl) as the reference electrode. Scan speed was 100 mV/s, room temperature. The results showed that no redox features were displayed between 0 and 3.0 V.

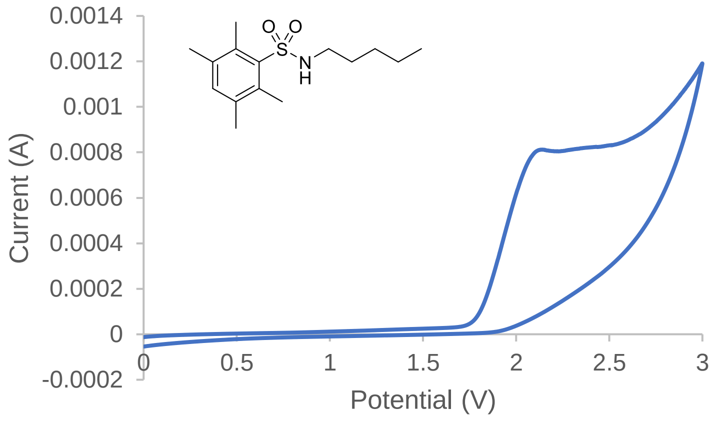

Supplementary Figure 21. The CV data of 2,3,5,6-tetramethyl-*N*-pentylbenzenesulfonamide in ACN. Conditions: a 6 mm-diameter glassy carbon as working electrode, Pt wire as the counter electrode and Ag/AgCl (sat'd KCl) as the reference electrode. Scan speed was 100 mV/s, room temperature. It was found that the half-peak potential of 2,3,5,6-tetramethyl-*N*-pentylbenzenesulfonamide in ACN was observed at 1.93 V (vs. SCE in ACN).

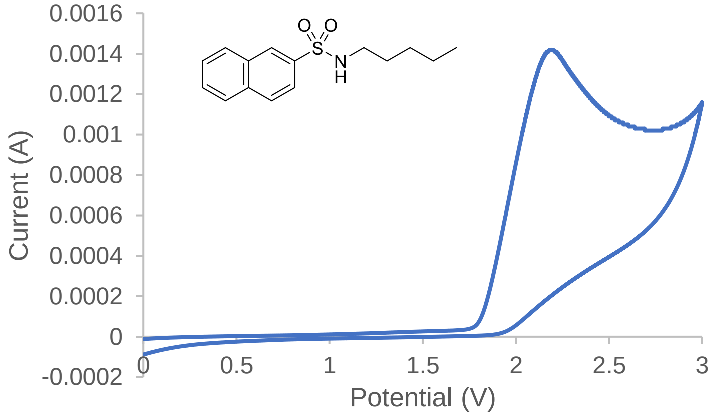

Supplementary Figure 22. The CV data of *N*-pentylnaphthalene-2-sulfonamide in ACN. Conditions: a 6 mm-diameter glassy carbon as working electrode, Pt wire as the counter electrode and Ag/AgCl (sat'd KCl) as the reference electrode. Scan speed was 100 mV/s, room temperature. It was found that the half-peak potential of *N*-pentylnaphthalene-2-sulfonamide in ACN was observed at 1.97 V (vs. SCE in ACN).

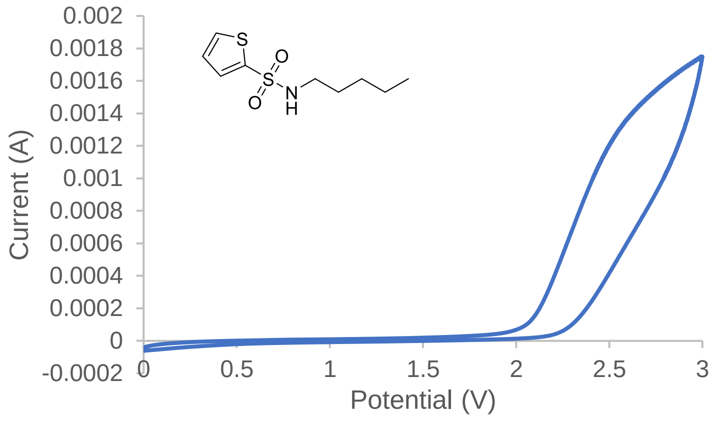

Supplementary Figure 23. The CV data of *N*-pentylthiophene-2-sulfonamide in ACN. Conditions: a 6 mm-diameter glassy carbon as working electrode, Pt wire as the counter electrode and Ag/AgCl (sat'd KCl) as the reference electrode. Scan speed was 100 mV/s, room temperature. The results showed that no redox features were displayed between 0 and 3.0 V.

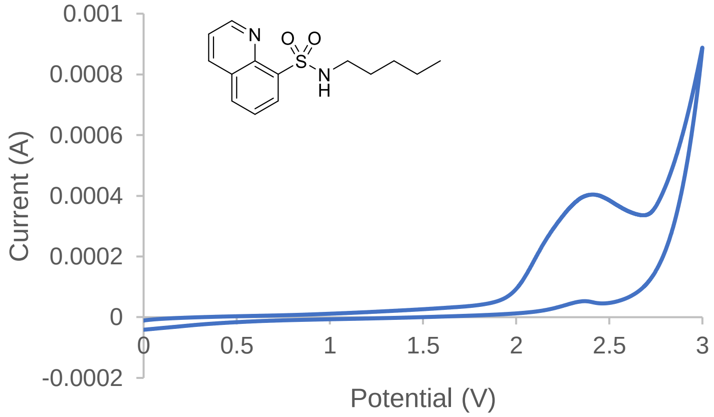

Supplementary Figure 24. The CV data of *N*-pentylquinoline-8-sulfonamide in ACN. Conditions: a 6 mm-diameter glassy carbon as working electrode, Pt wire as the counter electrode and Ag/AgCl (sat'd KCl) as the reference electrode. Scan speed was 100 mV/s, room temperature. It was found that the half-peak potential of *N*-pentylquinoline-8-sulfonamide in ACN was observed at 2.11 V (vs. SCE in ACN).

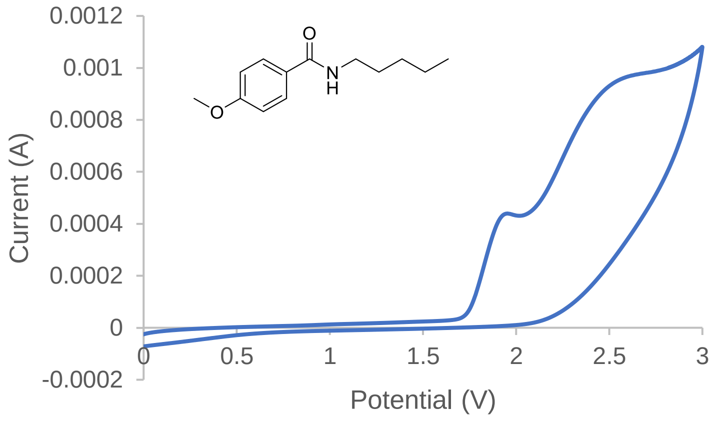

Supplementary Figure 25. The CV data of 4-methoxy-*N*-pentylbenzamide in ACN. Conditions: a 6 mm-diameter glassy carbon as working electrode, Pt wire as the counter electrode and Ag/AgCl (sat'd KCl) as the reference electrode. Scan speed was 100 mV/s, room temperature. It was found that the half-peak potential of 4-methoxy-*N*-pentylbenzamide in ACN was observed at 1.82 V (vs. SCE in ACN).

6.7 Stern-Volmer fluorescence quenching studies

Stern-Volmer luminescence quenching experiments of PC with 2-phenylquinoline **1a** were carried out with freshly prepared solutions of PC (5×10^{-5} M) in ACN at room temperature. All PC solutions were irradiated at 380 nm approximately and the emission intensity from 450 nm to 600 nm was recorded by F-7000 FL Spectrophotometer. It was shown that no significant fluorescence quenching was observed between excited PC and 2-phenylquinoline **1a** (**Supplementary Figure 26**).

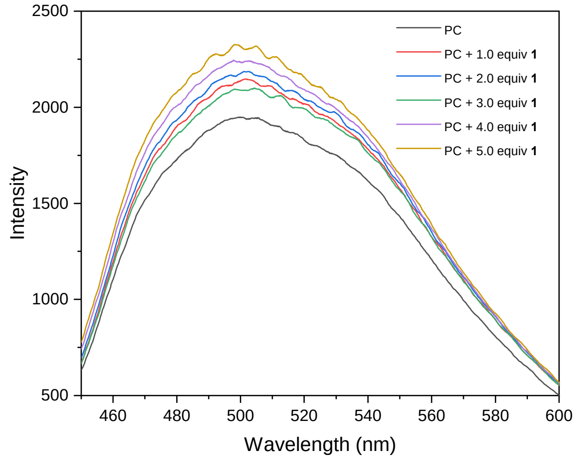

Supplementary Figure 26. Emission intensity of 5×10^{-5} M PC in ACN, with varied amount of **1a**

Stern-Volmer luminescence quenching experiments of PC with Co(dmgH)$_2$PyCl were carried out with freshly prepared solutions of PC (5 × 10^{-5} M) in ACN at room temperature. All PC solutions were irradiated at 380 nm approximately and the emission intensity from 450 nm to 600 nm was recorded by F-7000 FL Spectrophotometer. The resulting fluorescence emission spectra was shown in **Supplementary Figure 27**.

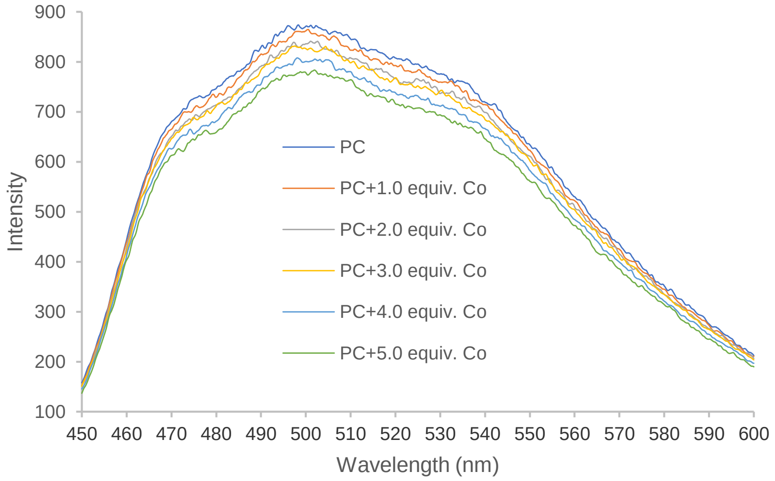

Supplementary Figure 27. Emission intensity of 5 × 10^{-5} M PC in ACN, with varied amount of Co(dmgH)$_2$PyCl

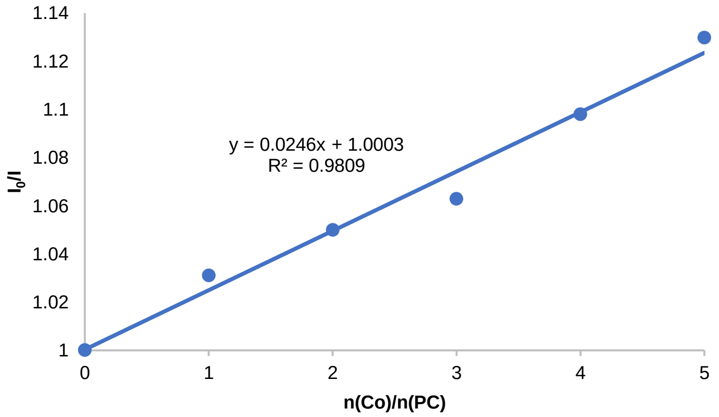

Supplementary Figure 28. Stern-Volmer plot of PC and Co(dmgH)$_2$PyCl

Stern-Volmer luminescence quenching experiments of PC with 4-methoxy-*N*-pentylbenzenesulfonamide **2a** were carried out with freshly prepared solutions of PC (5 × 10^{-4} M) in ACN at room temperature. All PC solutions were irradiated at 380 nm approximately and the emission intensity from 450 nm to 600 nm was recorded by F-7000 FL Spectrophotometer. The resulting fluorescence emission spectra was shown in **Supplementary Figure 29**.

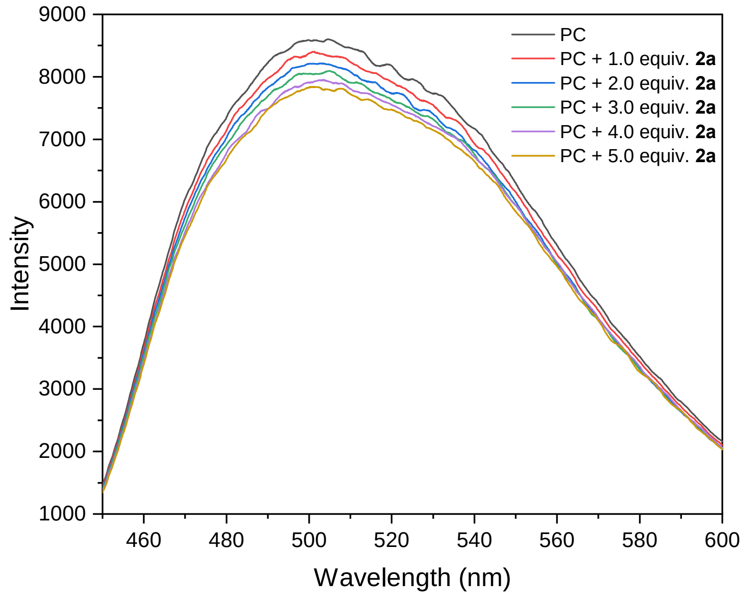

Supplementary Figure 29. Emission intensity of 5 × 10^{-4} M PC in ACN, with varied amount of **2a**

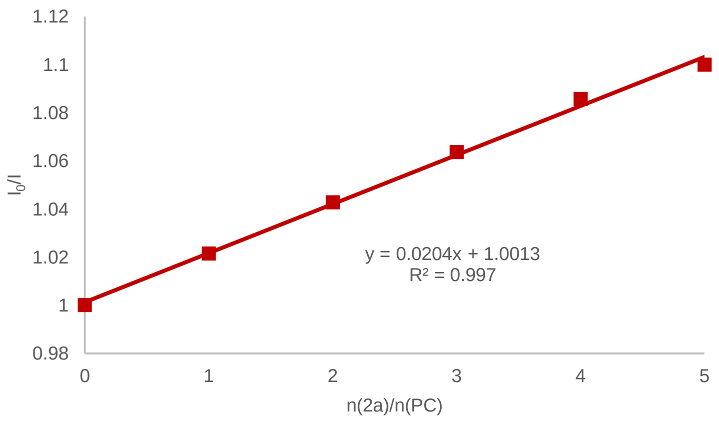

Supplementary Figure 30. Stern-Volmer plot of PC and **2a**

6.8 Hydrogen evolution detection

To a 50 mL Schlenk tube equipped with a magnetic stirring bar was added 2-phenylquinoline **1a** (1.0 mmol), 4-methoxy-*N*-pentylbenzenesulfonamide **2a** (2.0 mmol), Acr^+-Mes-ClO_4^- (2 mol%) and Co(dmgH)$_2$PyCl (5 mol%). After three cycles of evacuation and backfilling of the reaction flask with nitrogen, TFA (2.0 equiv.), ACN (7.5 mL) and HFIP (2.5 mL) were added to the tube under nitrogen. The mixture was then irradiated by two 25 W blue lamps for 24 h. After the reaction was

completed, the upper 20 mL gas content of the tube was taken by a gas-tight syringe and analyzed by GC-TCD. The results indicated the formation of hydrogen during the reaction (Figure S31).

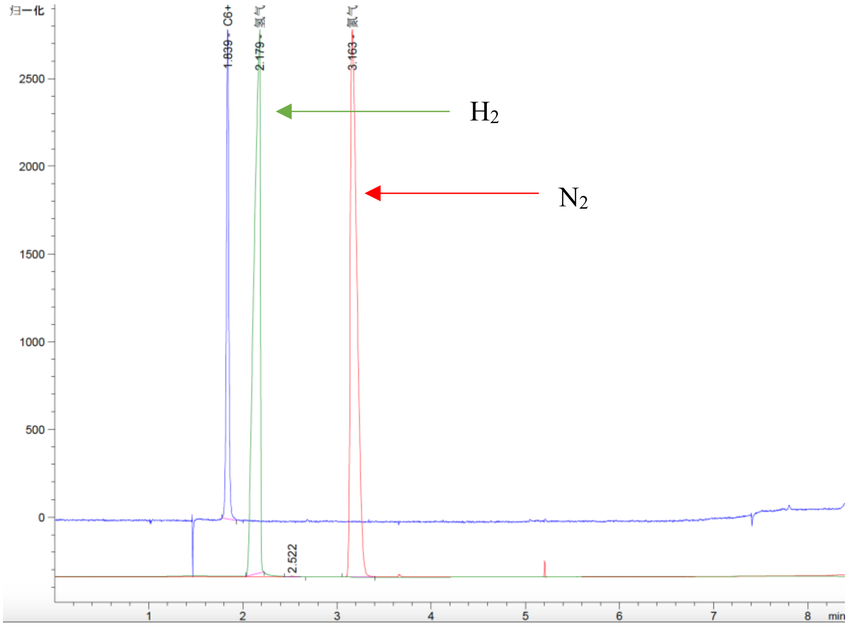

Supplementary Figure 31. Hydrogen evolution detected by GC-TCD

6.9 Light on/off experiments

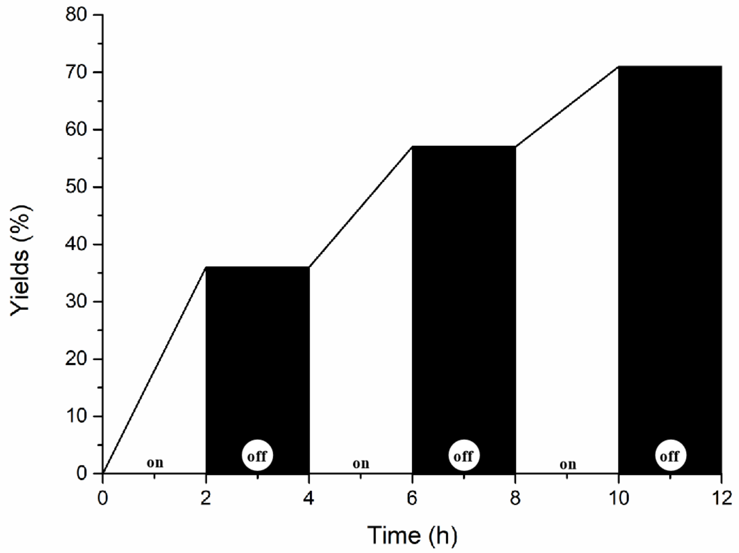

Supplementary Figure 32. On/off light experiments for the model reaction

6.10 Density functional theory calculations

Method

All the calculations were carried out by the Gaussian16 package.[1] The PBE0 hybrid functional[2] was applied for all calculations in combination with the D3BJ[3] dispersion correction. For geometry optimization, the def2-SV(P)[4] basis set and IEFPCM[5] solvent model for Acetonitrile were used. The frequencies were computed analytically at the same level of theory as the geometry optimizations to identify the nature of all stationary points being either minimum (no imaginary frequency) or transition state (only one imaginary frequency) and also to obtain the Gibbs free energy correction at 298.15 K. The final and solvation energies for the fully optimized structures in the acetonitrile were calculated by employing the SMD continuum solvation model[6] with the larger def2-TZVP basis set.

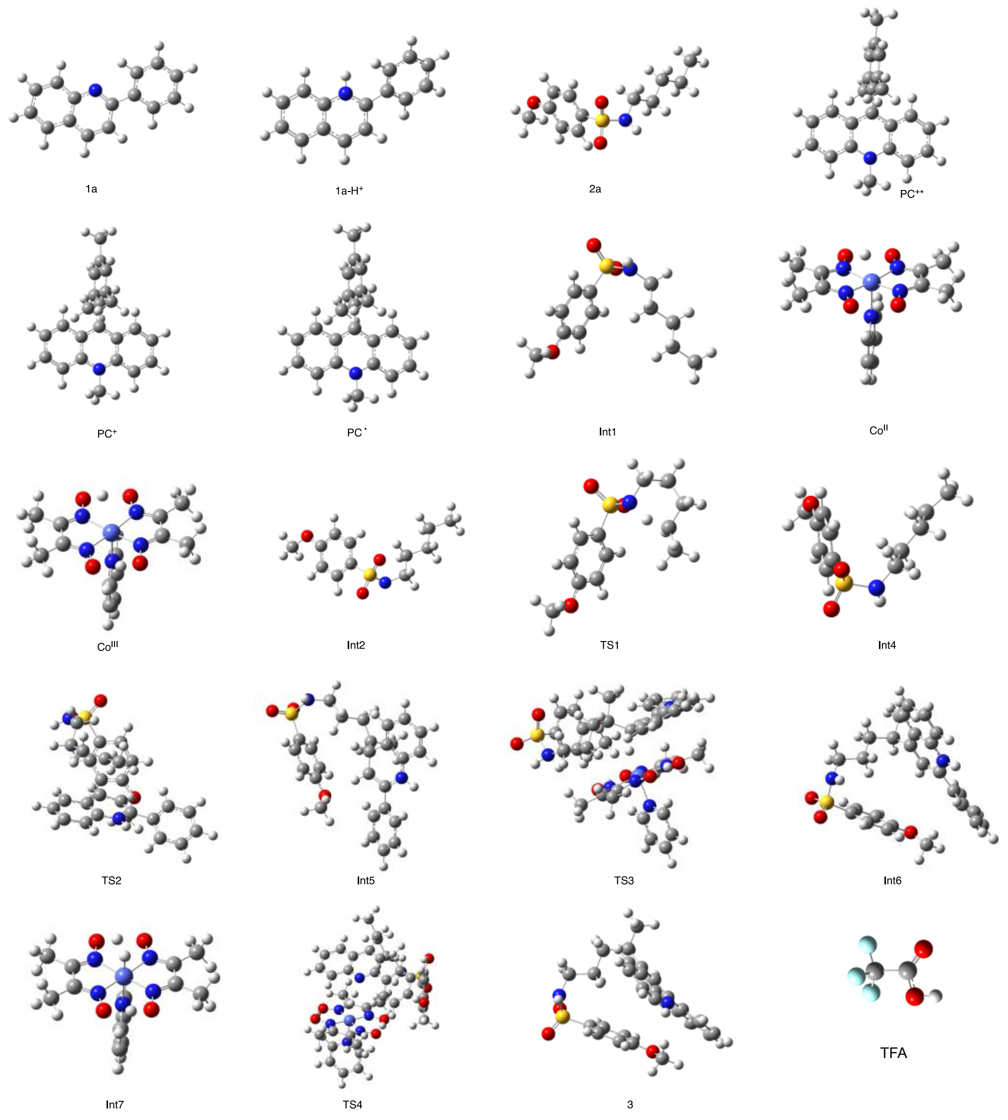

Supplementary Figure 33. The 3D structures of involved substrates, catalysts, intermediates and transition states

7. Characterization data for synthesized compounds

7.1 Characterization data for substrates

4-methoxy-*N*-pentylbenzenesulfonamide (2a)

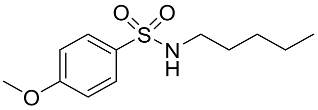

The substrate **2a** was prepared following **General procedure A** on a 10 mmol scale, which was obtained as colorless oil in 95% yield.

^{1}H NMR (400 MHz, $CDCl_3$) δ 7.90 – 7.73 (m, 2H), 7.05 – 6.88 (m, 2H), 4.54 (br, 1H), 3.86 (s, 3H), 2.90 (t, *J* = 7.1 Hz, 2H), 1.44 (p, *J* = 6.8 Hz, 2H), 1.30 – 1.16 (m, 4H), 0.83 (t, *J* = 6.3 Hz, 3H).

^{13}C NMR (101 MHz, $CDCl_3$) δ 162.82, 131.61, 129.21, 114.21, 55.61, 43.18, 29.21, 28.67, 22.14, 13.87.

Analytical data matched that reported in literature.[7]

***N*-hexyl-4-methoxybenzenesulfonamide (2b)**

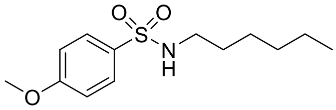

The substrate **2b** was prepared following **General procedure A** on a 10 mmol scale, which was obtained as white solid in 96% yield.

^{1}H NMR (400 MHz, $CDCl_3$) δ 7.83 – 7.77 (m, 2H), 7.00 – 6.94 (m, 2H), 4.43 (br, 1H), 3.86 (s, 3H), 2.90 (t, *J* = 7.1 Hz, 2H), 1.49 – 1.38 (m, 2H), 1.31 – 1.13 (m, 6H), 0.83 (t, *J* = 7.0 Hz, 3H).

^{13}C NMR (101 MHz, $CDCl_3$) δ 162.81, 131.66, 129.20, 114.20, 55.59, 43.19, 31.23, 29.49, 26.19, 22.44, 13.91.

Analytical data matched that reported in literature.[7]

***N*-heptyl-4-methoxybenzenesulfonamide (2c)**

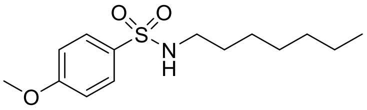

The substrate **2c** was prepared following **General procedure A** on a 10 mmol scale, which was obtained as white solid in 93% yield.

^{1}H NMR (400 MHz, $CDCl_3$) δ 7.80 (d, *J* = 8.6 Hz, 2H), 6.97 (d, *J* = 8.6 Hz, 2H), 4.65 – 4.44 (br, 1H), 3.86 (s, 3H), 2.91 (q, *J* = 7.0, 6.5 Hz, 2H), 1.50 – 1.36 (m, 2H), 1.32 – 1.12 (m, 8H), 0.85 (t, *J* = 6.6 Hz, 3H).

^{13}C NMR (101 MHz, $CDCl_3$) δ 162.82, 131.57, 129.21, 114.20, 55.61, 43.19, 31.63, 29.51, 28.73, 26.49, 22.52, 14.04.

Analytical data matched that reported in literature.[7]

4-methoxy-*N*-octylbenzenesulfonamide (2d)

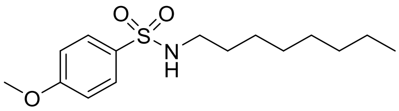

The substrate **2d** was prepared following **General procedure A** on a 10 mmol scale, which was obtained as white solid in 95% yield.

^{1}H NMR (400 MHz, $CDCl_3$) δ 7.83 – 7.77 (m, 2H), 7.00 – 6.94 (m, 2H), 4.67 (br, 1H), 3.86 (s, 3H), 2.90 (t, J = 7.2 Hz, 2H), 1.49 – 1.35 (m, 2H), 1.30 – 1.13 (m, 10H), 0.85 (t, J = 6.1 Hz, 3H).

^{13}C NMR (101 MHz, $CDCl_3$) δ 162.81, 131.67, 129.20, 114.20, 55.58, 43.18, 31.70, 29.51, 29.07, 29.01, 26.52, 22.58, 14.03.

Analytical data matched that reported in literature.[7]

_N_-(2,2-dimethylhexyl)-4-methoxybenzenesulfonamide (2e)

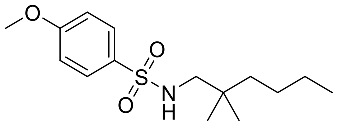

The substrate **2e** was prepared following **General procedure A** on a 10 mmol scale, which was obtained as white solid in 94% yield.

^{1}H NMR (400 MHz, $CDCl_3$) δ 7.82 – 7.77 (m, 2H), 7.00 – 6.94 (m, 2H), 4.49 (t, J = 6.7 Hz, 1H), 3.87 (s, 3H), 2.66 (d, J = 6.8 Hz, 2H), 1.26 – 1.06 (m, 6H), 0.85 (t, J = 7.2 Hz, 3H), 0.82 (s, 6H).

^{13}C NMR (101 MHz, $CDCl_3$) δ 162.77, 131.72, 129.19, 114.18, 55.57, 52.98, 39.27, 33.63, 25.87, 24.92, 23.39, 14.01.

HRMS (ESI+) m/z Calcd for $C_{15}H_{26}NO_3S^+$: 300.1628 $[M+H]^+$; found: 300.1634.

_N_-(2-ethylhexyl)-4-methoxybenzenesulfonamide (2f)

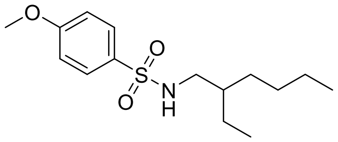

The substrate **2i** was prepared following **General procedure A** on a 10 mmol scale, which was obtained as yellow oil in 90% yield.

^{1}H NMR (400 MHz, $CDCl_3$) δ 7.83 – 7.77 (m, 2H), 7.01 – 6.93 (m, 2H), 4.51 (t, J = 6.5 Hz, 1H), 3.86 (s, 3H), 2.83 (t, J = 5.7 Hz, 2H), 1.44 – 1.07 (m, 9H), 0.84 (t, J = 7.0 Hz, 3H), 0.78 (t, J = 7.3 Hz, 3H).

^{13}C NMR (101 MHz, $CDCl_3$) δ 162.79, 131.63, 129.21, 114.18, 55.59, 45.74, 39.16, 30.66, 28.68, 23.90, 22.88, 13.98, 10.68.

Analytical data matched that reported in literature.[8]

4-methoxy-_N_-(5-methylhexyl)benzenesulfonamide (2g)

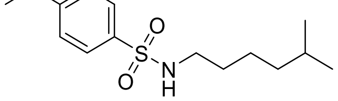

The substrate **2k** was prepared following **General procedure A** on a 10 mmol scale, which was obtained as colorless oil in 86% yield.

^{1}H NMR (400 MHz, $CDCl_3$) δ 7.84 – 7.77 (m, 2H), 7.01 – 6.93 (m, 2H), 4.52 (br, 1H), 3.86 (s, 3H), 2.91 (t, J = 7.1 Hz, 2H), 1.53 – 1.37 (m, 3H), 1.31 – 1.16 (m, 2H), 1.14 – 1.01 (m, 2H), 0.81 (d, J = 6.6 Hz, 6H).

^{13}C NMR (101 MHz, $CDCl_3$) δ 162.82, 131.68, 129.20, 114.21, 55.59, 43.21, 38.35, 29.80, 27.80, 24.33, 22.47.

Analytical data matched that reported in literature.[9]

4-methoxy-*N*-(1-phenylpentyl)benzenesulfonamide (2h)

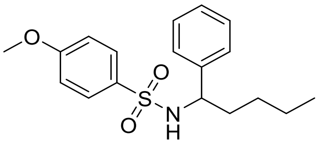

The substrate **2f** was prepared following **General procedure A** on a 10 mmol scale, which was obtained as white solid in 90% yield.

^{1}H NMR (400 MHz, $CDCl_3$) δ 7.60 – 7.54 (m, 2H), 7.17 – 7.10 (m, 3H), 7.05 – 6.97 (m, 2H), 6.80 – 6.71 (m, 2H), 5.11 (br, 1H), 4.24 (q, J = 7.3 Hz, 1H), 3.80 (s, 3H), 1.83 – 1.60 (m, 2H), 1.30 – 1.02 (m, 4H), 0.80 (t, J = 7.1 Hz, 3H).

^{13}C NMR (101 MHz, $CDCl_3$) δ 162.52, 141.15, 132.50, 129.12, 128.36, 127.23, 126.52, 113.80, 58.34, 55.52, 37.37, 27.98, 22.19, 13.79.

HRMS (ESI+) *m/z* Calcd for $C_{18}H_{24}NO_3S^+$: 334.1471 [M+H]$^+$; found: 334.1469.

methyl (*S*)-2-((4-methoxyphenyl)sulfonamido)hexanoate (2i)

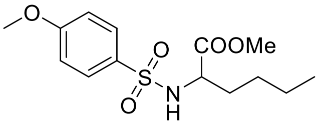

The substrate **2j** was prepared following the **Procedures for preparation of specific compounds**, which was obtained as white solid in 37% yield.

^{1}H NMR (400 MHz, $CDCl_3$) δ 7.79 – 7.73 (m, 2H), 6.97 – 6.92 (m, 2H), 5.10 (d, J = 9.0 Hz, 1H), 3.92 – 3.86 (m, 1H), 3.85 (s, 3H), 3.50 (s, 3H), 1.77 – 1.51 (m, 2H), 1.36 – 1.18 (m, 4H), 0.85 (t, J = 7.0 Hz, 3H).

^{13}C NMR (101 MHz, $CDCl_3$) δ 172.41, 163.00, 131.36, 129.42, 114.12, 55.65, 55.62, 52.38, 33.07, 26.99, 22.03, 13.74.

Analytical data matched that reported in literature.[7]

4-methoxy-*N*-(2-methylpentyl)benzenesulfonamide (2j)

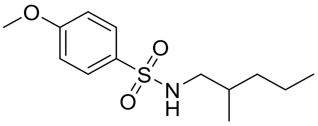

The substrate **2g** was prepared following **General procedure A** on a 5 mmol scale, which was

obtained as yellow oil in 94% yield.

^{1}H NMR (400 MHz, $CDCl_3$) δ 7.87 – 7.75 (m, 2H), 7.03 – 6.91 (m, 2H), 4.61 (br, 1H), 3.86 (s, 3H), 2.90 – 2.61 (m, 2H), 1.64 – 1.47 (m, 1H), 1.31 – 0.99 (m, 4H), 0.86 – 0.80 (m, 6H).

^{13}C NMR (101 MHz, $CDCl_3$) δ 162.79, 131.73, 129.18, 114.19, 55.59, 49.05, 36.22, 32.87, 19.79, 17.42, 14.12.

Analytical data matched that reported in literature.[7]

4-methoxy-*N*-(3-methylpentyl)benzenesulfonamide (2k)

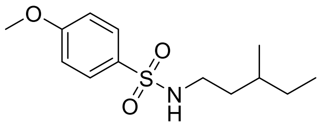

The substrate **2h** was prepared following **General procedure A** on a 5 mmol scale, which was obtained as yellow oil in 87% yield.

^{1}H NMR (600 MHz, $CDCl_3$) δ 7.82 – 7.78 (m, 2H), 7.01 – 6.94 (m, 2H), 4.59 (br, 1H), 3.86 (s, 3H), 2.98 – 2.86 (m, 2H), 1.49 – 1.40 (m, 1H), 1.37 – 1.29 (m, 1H), 1.29 – 1.18 (m, 2H), 1.13 – 1.04 (m, 1H), 0.83 – 0.75 (m, 6H).

^{13}C NMR (151 MHz, $CDCl_3$) δ 162.81, 131.58, 129.21, 114.21, 55.61, 41.30, 36.15, 31.75, 29.17, 18.79, 11.14.

HRMS (ESI+) *m/z* Calcd for $C_{13}H_{22}NO_3S^+$: 272.1315 [M+H]$^+$; found: 272.1320.

***N*-(2-ethoxyethyl)-4-methoxybenzenesulfonamide (2l)**

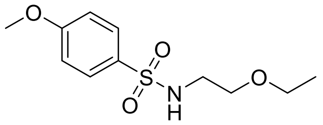

The substrate **2l** was prepared following **General procedure B** on a 10 mmol scale, which was obtained as colorless oil in 82% yield.

^{1}H NMR (400 MHz, $CDCl_3$) δ 7.82 – 7.75 (m, 2H), 7.01 – 6.92 (m, 2H), 4.88 (br, 1H), 3.86 (s, 3H), 3.47 – 3.35 (m, 4H), 3.09 (t, J = 5.1 Hz, 2H), 1.12 (t, J = 7.0 Hz, 3H).

^{13}C NMR (101 MHz, $CDCl_3$) δ 162.88, 131.63, 129.18, 114.23, 68.37, 66.47, 55.60, 42.95, 14.95.

HRMS (ESI+) *m/z* Calcd for $C_{11}H_{18}NO_4S^+$: 260.0951 [M+H]$^+$; found: 260.0958.

***N*-(6-chlorohexyl)-4-methoxybenzenesulfonamide (2m)**

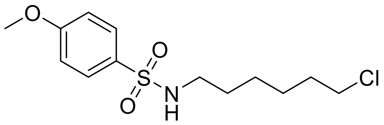

The substrate **2m** was prepared following the **Procedures for preparation of specific compounds**, which was obtained as yellow oil in 90% yield.

^{1}H NMR (400 MHz, $CDCl_3$) δ 7.82 – 7.77 (m, 2H), 7.00 – 6.95 (m, 2H), 4.55 (br, 1H), 3.87 (s, 3H), 3.48 (t, J = 6.6 Hz, 2H), 2.92 (t, J = 6.7 Hz, 2H), 1.70 (p, J = 6.7 Hz, 2H), 1.47 (p, J = 7.2 Hz, 2H), 1.41 – 1.24 (m, 4H).

^{13}C NMR (101 MHz, $CDCl_3$) δ 162.87, 131.62, 129.19, 114.25, 55.61, 44.82, 42.99, 32.32, 29.38,

26.29, 25.77.
Analytical data matched that reported in literature.[7]

ethyl 8-((4-methoxyphenyl)sulfonamido)octanoate (2n)

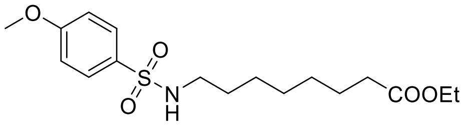

The substrate **2n** was prepared following **General procedure B** on a 5 mmol scale, which was obtained as white solid in 63% yield.

^{1}H NMR (400 MHz, $CDCl_3$) δ 7.79 (d, *J* = 8.2 Hz, 2H), 6.97 (d, *J* = 8.2 Hz, 2H), 4.55 (br, 1H), 4.10 (q, *J* = 6.6 Hz, 2H), 3.86 (s, 3H), 2.89 (t, *J* = 6.1 Hz, 2H), 2.25 (t, *J* = 7.1 Hz, 2H), 1.63 – 1.49 (m, 2H), 1.50 – 1.34 (m, 2H), 1.33 – 1.11 (m, 9H).

^{13}C NMR (101 MHz, $CDCl_3$) δ 173.81, 162.81, 131.54, 129.20, 114.21, 60.22, 55.61, 43.12, 34.25, 29.44, 28.88, 28.68, 26.31, 24.77, 14.26.

Analytical data matched that reported in literature.[7]

6-((4-methoxyphenyl)sulfonamido)hexyl 4-methylbenzoate (2o)

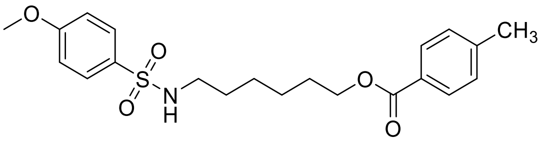

The substrate **2o** was prepared following **General procedure C** on a 1 mmol scale, which was obtained as white solid in 82% yield.

^{1}H NMR (400 MHz, $CDCl_3$) δ 7.91 (d, *J* = 5.8 Hz, 2H), 7.83 – 7.75 (m, 2H), 7.23 (d, *J* = 6.6 Hz, 2H), 6.97 (d, *J* = 7.7 Hz, 2H), 4.63 (br, 1H), 4.30 – 4.17 (m, 2H), 3.86 (s, 3H), 2.99 – 2.85 (m, 2H), 2.41 (s, 3H), 1.77 – 1.60 (m, 2H), 1.55 – 1.43 (m, 2H), 1.41 – 1.27 (m, 4H).

^{13}C NMR (101 MHz, $CDCl_3$) δ 166.73, 162.84, 143.50, 131.68, 129.54, 129.19, 129.05, 127.68, 114.23, 64.57, 55.58, 43.03, 29.46, 28.57, 26.16, 25.51, 21.61.

Analytical data matched that reported in literature.[7]

***N*-(cyclohexylmethyl)-4-methoxybenzenesulfonamide (2p)**

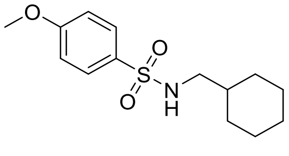

The substrate **2p** was prepared following **General procedure A** on a 5 mmol scale, which was obtained as white solid in 78% yield.

^{1}H NMR (400 MHz, $CDCl_3$) δ 7.82 – 7.75 (m, 2H), 7.01 – 6.93 (m, 2H), 4.52 (br, 1H), 3.87 (s, 3H), 2.74 (t, *J* = 5.2 Hz, 2H), 1.73 – 1.58 (m, 5H), 1.45 – 1.32 (m, 1H), 1.27 – 1.03 (m, 3H), 0.84 (td, *J* = 13.2, 9.9 Hz, 2H).

^{13}C NMR (101 MHz, $CDCl_3$) δ 162.77, 131.76, 129.17, 114.18, 55.59, 49.35, 37.73, 30.57, 26.26, 25.65.

Analytical data matched that reported in literature.[9]

***N*-(2-cyclopentylethyl)-4-methoxybenzenesulfonamide (2q)**

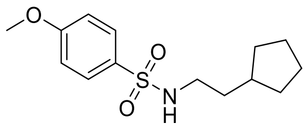

The substrate **2q** was prepared following **General procedure A** on a 5 mmol scale, which was obtained as white solid in 84% yield.

^{1}H NMR (600 MHz, $CDCl_3$) δ 7.82 – 7.77 (m, 2H), 6.99 – 6.95 (m, 2H), 4.56 (t, *J* = 6.2 Hz, 1H), 3.86 (s, 3H), 2.92 (td, *J* = 7.4, 5.7 Hz, 2H), 1.76 – 1.64 (m, 3H), 1.59 – 1.51 (m, 2H), 1.50 – 1.42 (m, 4H), 1.04 – 0.95 (m, 2H).

^{13}C NMR (151 MHz, $CDCl_3$) δ 162.80, 131.58, 129.21, 114.20, 55.61, 42.60, 37.30, 35.78, 32.41, 24.99.

HRMS (ESI+) *m*/*z* Calcd for $C_{14}H_{22}NO_3S^+$: 284.1315 [*M*+H]$^+$; found: 284.1312.

***N*-(2-cyclohexylethyl)-4-methoxybenzenesulfonamide (2r)**

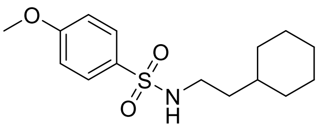

The substrate **2r** was prepared following **General procedure A** on a 5 mmol scale, which was obtained as white solid in 76% yield.

^{1}H NMR (400 MHz, $CDCl_3$) δ 7.83 – 7.76 (m, 2H), 7.00 – 6.94 (m, 2H), 4.47 (br, 1H), 3.87 (s, 3H), 2.93 (t, *J* = 7.2 Hz, 2H), 1.72 – 1.47 (m, 5H), 1.37 – 1.28 (m, 2H), 1.29 – 1.03 (m, 4H), 0.87 – 0.75 (m, 2H).

^{13}C NMR (101 MHz, $CDCl_3$) δ 162.81, 131.48, 129.23, 114.21, 55.63, 40.93, 36.92, 34.83, 32.96, 26.40, 26.11.

Analytical data matched that reported in literature.[9]

***N*-(3-cyclopentylpropyl)-4-methoxybenzenesulfonamide (2s)**

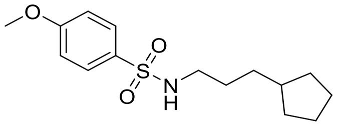

The substrate **2t** was prepared following **General procedure A** on a 5 mmol scale, which was obtained as white solid in 69% yield.

^{1}H NMR (600 MHz, $CDCl_3$) δ 7.81 – 7.78 (m, 2H), 7.00 – 6.95 (m, 2H), 4.62 (br, 1H), 3.86 (dd, *J* = 2.2, 1.1 Hz, 3H), 2.90 (t, *J* = 6.5 Hz, 2H), 1.72 – 1.60 (m, 3H), 1.58 – 1.50 (m, 2H), 1.50 – 1.41 (m, 4H), 1.28 – 1.19 (m, 2H), 1.04 – 0.92 (m, 2H).

^{13}C NMR (151 MHz, $CDCl_3$) δ 162.80, 131.64, 129.21, 114.20, 55.61, 43.44, 39.58, 32.95, 32.55, 28.73, 25.09.

HRMS (ESI+) *m*/*z* Calcd for $C_{15}H_{24}NO_3S^+$: 298.1471 [*M*+H]$^+$; found: 298.1481.

***N*-(3-cyclohexylpropyl)-4-methoxybenzenesulfonamide (2t)**

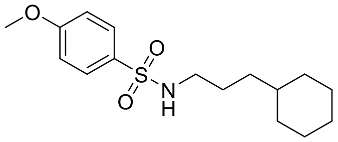

The substrate **2u** was prepared following **General procedure A** on a 5 mmol scale, which was obtained as white solid in 77% yield.

^{1}H NMR (600 MHz, $CDCl_3$) δ 7.82 – 7.78 (m, 2H), 7.00 – 6.95 (m, 2H), 4.50 (br, 1H), 3.86 (s, 3H), 2.89 (td, *J* = 7.3, 3.5 Hz, 2H), 1.73 – 1.53 (m, 5H), 1.44 (p, *J* = 6.9 Hz, 2H), 1.21 – 1.05 (m, 6H), 0.86 – 0.73 (m, 2H).

^{13}C NMR (151 MHz, $CDCl_3$) δ 162.81, 131.64, 129.22, 114.21, 55.60, 43.51, 37.14, 34.19, 33.19, 26.88, 26.56, 26.25.

Analytical data matched that reported in literature.[8]

4-methoxy-*N*-(4-methylpentyl)benzenesulfonamide (2u)

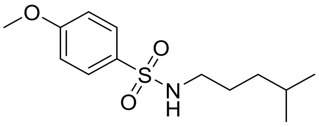

The substrate **2v** was prepared following **General procedure A** on a 5 mmol scale, which was obtained as colorless oil in 91% yield.

^{1}H NMR (600 MHz, $CDCl_3$) δ 7.81 – 7.78 (m, 2H), 6.99 – 6.95 (m, 2H), 4.58 (br, 1H), 3.86 (s, 3H), 2.95 – 2.84 (m, 2H), 1.50 – 1.39 (m, 3H), 1.15 – 1.07 (m, 2H), 0.81 (d, *J* = 6.7 Hz, 6H).

^{13}C NMR (151 MHz, $CDCl_3$) δ 162.81, 131.61, 129.21, 114.21, 55.61, 43.48, 35.67, 27.58, 27.41, 22.42.

Analytical data matched that reported in literature.[9]

***N*-butyl-4-methoxybenzenesulfonamide (2v)**

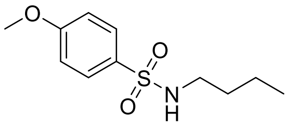

The substrate **2w** was prepared following **General procedure A** on a 10 mmol scale, which was obtained as colorless oil in 94% yield.

^{1}H NMR (400 MHz, $CDCl_3$) δ 7.83 – 7.76 (m, 2H), 6.99 – 6.93 (m, 2H), 4.73 (br, 1H), 3.86 (s, 3H), 2.90 (t, *J* = 7.1 Hz, 2H), 1.42 (p, *J* = 7.2 Hz, 2H), 1.27 (h, *J* = 7.2 Hz, 2H), 0.83 (t, *J* = 7.3 Hz, 3H).

^{13}C NMR (101 MHz, $CDCl_3$) δ 162.79, 131.52, 129.21, 114.21, 55.62, 42.88, 31.51, 19.71, 13.55.

Analytical data matched that reported in literature.[8]

***N*-isopentyl-4-methoxybenzenesulfonamide (2w)**

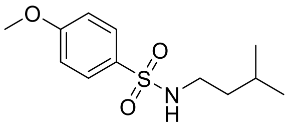

The substrate **2x** was prepared following **General procedure B** on a 5 mmol scale, which was obtained as white solid in 86% yield.

^{1}H NMR (400 MHz, $CDCl_3$) δ 7.83 – 7.78 (m, 2H), 7.00 – 6.94 (m, 2H), 4.60 (br, 1H), 3.86 (s, 3H), 2.92 (t, J = 7.4 Hz, 2H), 1.57 (dp, J = 13.4, 6.7 Hz, 1H), 1.32 (q, J = 7.2 Hz, 2H), 0.81 (d, J = 6.8 Hz, 6H).

^{13}C NMR (101 MHz, $CDCl_3$) δ 162.82, 131.49, 129.24, 114.23, 55.63, 41.45, 38.30, 25.42, 22.26.

Analytical data matched that reported in literature.[8]

4-methoxy-*N*-(4-phenylbutyl)benzenesulfonamide (2x)

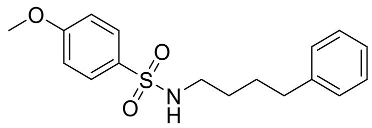

The substrate **2s** was prepared following **General procedure A** on a 5 mmol scale, which was obtained as white solid in 87% yield.

^{1}H NMR (400 MHz, $CDCl_3$) δ 7.81 – 7.75 (m, 2H), 7.29 – 7.04 (m, 5H), 6.99 – 6.93 (m, 2H), 4.60 (t, J = 6.3 Hz, 1H), 3.85 (s, 3H), 2.93 (q, J = 6.5 Hz, 2H), 2.54 (t, J = 7.5 Hz, 2H), 1.63 – 1.53 (m, 2H), 1.52 – 1.43 (m, 2H).

^{13}C NMR (101 MHz, $CDCl_3$) δ 162.84, 141.78, 131.63, 129.19, 128.33, 125.84, 114.24, 55.60, 43.02, 35.24, 29.09, 28.21.

Analytical data matched that reported in literature.[7]

4-methoxy-*N*-methyl-*N*-pentylbenzenesulfonamide (2y)

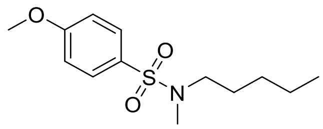

The substrate **2y** was prepared following **General procedure A** on a 5 mmol scale, which was obtained as colorless oil in 91% yield.

^{1}H NMR (400 MHz, $CDCl_3$) δ 7.74 – 7.67 (m, 2H), 7.00 – 6.95 (m, 2H), 3.86 (s, 3H), 2.99 – 2.92 (m, 2H), 2.68 (s, 3H), 1.51 (p, J = 7.3 Hz, 2H), 1.38 – 1.23 (m, 4H), 0.88 (t, J = 6.8 Hz, 3H).

^{13}C NMR (101 MHz, $CDCl_3$) δ 162.74, 129.48, 129.42, 114.13, 55.55, 50.09, 34.53, 28.67, 27.27, 22.24, 13.91.

HRMS (ESI+) *m/z* Calcd for $C_{13}H_{22}NO_3S^+$: 272.1315 [M+H]$^+$; found: 272.1319.

3-((4-methoxyphenyl)sulfonyl)-3-azabicyclo[3.1.0]hexane (2z)

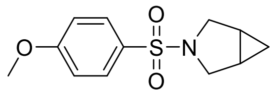

The substrate **2z** was prepared following **General procedure A** on a 2 mmol scale, which was obtained as white solid in 88% yield.

^{1}H NMR (400 MHz, $CDCl_3$) δ 7.76 – 7.68 (m, 2H), 7.02 – 6.95 (m, 2H), 3.87 (s, 3H), 3.49 (d, J = 9.1 Hz, 2H), 3.04 (dt, J = 9.2, 1.8 Hz, 2H), 1.42 – 1.37 (m, 2H), 0.54 (td, J = 7.8, 5.1 Hz, 1H), 0.37 (q, J = 4.3 Hz, 1H).

^{13}**C NMR** (101 MHz, $CDCl_3$) δ 162.87, 129.60, 128.29, 114.09, 55.60, 49.80, 15.60, 7.58.
HRMS (ESI+) *m/z* Calcd for $C_{12}H_{16}NO_3S^+$: 254.0845 [*M*+H]$^+$; found: 254.0844.

(2*R*)-1,7,7-trimethylbicyclo[2.2.1]heptan-2-yl 7-((4-methoxyphenyl)sulfonamido)heptanoate (2aa)

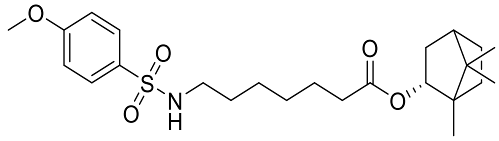

The substrate **2aa** was prepared following **General procedure D** on a 2 mmol scale, which was obtained as colorless oil in 65% yield.
^{1}**H NMR** (400 MHz, $CDCl_3$) δ 7.83 – 7.73 (m, 2H), 7.00 – 6.94 (m, 2H), 4.86 (ddd, *J* = 10.0, 3.5, 2.1 Hz, 1H), 4.60 (t, *J* = 6.3 Hz, 1H), 3.86 (s, 3H), 2.90 (q, *J* = 6.6 Hz, 2H), 2.37 – 2.29 (m, 1H), 2.26 (t, *J* = 7.4 Hz, 2H), 1.90 (ddd, *J* = 12.4, 9.3, 4.5 Hz, 1H), 1.78 – 1.64 (m, 2H), 1.56 (p, *J* = 7.3 Hz, 2H), 1.49 – 1.41 (m, 2H), 1.37 – 1.15 (m, 7H), 0.89 (s, 3H), 0.86 (s, 3H), 0.80 (s, 3H).
^{13}**C NMR** (101 MHz, $CDCl_3$) δ 173.99, 162.81, 131.54, 129.20, 114.21, 79.68, 55.62, 48.73, 47.78, 44.88, 43.09, 36.84, 34.48, 29.38, 28.56, 28.06, 27.12, 26.22, 24.88, 19.71, 18.85, 13.53.
HRMS (ESI+) *m/z* Calcd for $C_{24}H_{38}NO_5S^+$: 452.2465 [*M*+H]$^+$; found: 452.2478.

(2*R*,5*S*)-2-isopropyl-5-methylcyclohexyl 7-((4-methoxyphenyl)sulfonamido)heptanoate (2ab)

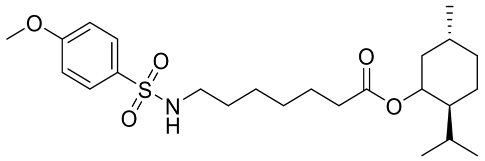

The substrate **2ab** was prepared following **General procedure D** on a 2 mmol scale, which was obtained as colorless oil in 67% yield.
^{1}**H NMR** (400 MHz, $CDCl_3$) δ 7.79 (d, *J* = 8.9 Hz, 2H), 6.97 (d, *J* = 8.9 Hz, 2H), 4.65 (td, *J* = 10.9, 4.2 Hz, 1H), 4.51 (br, 1H), 3.86 (s, 3H), 2.90 (t, *J* = 7.2 Hz, 2H), 2.23 (t, *J* = 7.4 Hz, 2H), 1.99 – 1.90 (m, 1H), 1.89 – 1.77 (m, 1H), 1.72 – 1.61 (m, 2H), 1.61 – 1.50 (m, 2H), 1.50 – 1.39 (m, 3H), 1.39 – 1.29 (m, 1H), 1.29 – 1.21 (m, 4H), 1.10 – 0.80 (m, 9H), 0.73 (d, *J* = 6.8 Hz, 3H).
^{13}**C NMR** (101 MHz, $CDCl_3$) δ 173.25, 162.81, 131.53, 129.20, 114.22, 73.99, 55.61, 46.99, 43.08, 40.94, 34.51, 34.25, 31.38, 29.38, 28.53, 26.25, 26.19, 24.85, 23.40, 22.04, 20.77, 16.29.
HRMS (ESI+) *m/z* Calcd for $C_{24}H_{40}NO_5S^+$: 454.2622 [*M*+H]$^+$; found: 454.2615.

((3a*S*,5a*R*,8a*R*,8b*S*)-2,2,7,7-tetramethyltetrahydro-3a*H*-bis([1,3]dioxolo)[4,5-*b*:4',5'-*d*]pyran-3a-yl)methyl 7-((4-methoxyphenyl)sulfonamido)heptanoate (2ac)

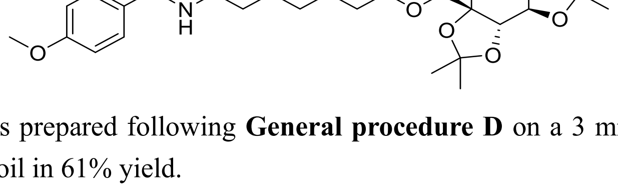

The substrate **2ac** was prepared following **General procedure D** on a 3 mmol scale, which was obtained as colorless oil in 61% yield.
^{1}**H NMR** (600 MHz, $CDCl_3$) δ 7.76 – 7.70 (m, 2H), 6.90 (td, J = 6.1, 2.4 Hz, 2H), 5.16 – 4.96 (m, 1H), 4.53 (td, J = 6.4, 4.9, 2.5 Hz, 1H), 4.31 (dd, J = 11.9, 4.5 Hz, 1H), 4.23 (dd, J = 4.6, 2.3 Hz,

1H), 4.17 (dt, J = 6.3, 3.1 Hz, 1H), 3.94 (dd, J = 12.5, 5.5 Hz, 1H), 3.83 (dt, J = 13.2, 2.7 Hz, 1H), 3.79 (d, J = 4.2 Hz, 3H), 3.67 (dd, J = 13.2, 5.5 Hz, 1H), 2.81 (p, J = 6.7 Hz, 2H), 2.24 (q, J = 6.6, 5.8 Hz, 2H), 1.53 – 1.44 (m, 5H), 1.41 – 1.34 (m, 5H), 1.32 (d, J = 4.9 Hz, 3H), 1.26 (d, J = 5.2 Hz, 3H), 1.18 (tt, J = 7.0, 3.8 Hz, 4H).

^{13}C NMR (151 MHz, $CDCl_3$) δ 172.77, 162.69, 131.64, 129.08, 114.14, 108.98, 108.63, 101.46, 70.69, 70.44, 69.97, 64.98, 61.14, 55.54, 42.94, 33.81, 29.20, 28.42, 26.41, 26.09, 25.82, 25.18, 24.45, 24.01.

HRMS (ESI+) *m*/*z* Calcd for $C_{26}H_{40}NO_{10}S^+$: 558.2367 [*M*+H]$^+$; found: 558.2374.

<u>*N*-(6-(1,1-dioxido-3-oxobenzo[*d*]isothiazol-2(3*H*)-yl)hexyl)-4-methoxybenzenesulfonamide (2ad)</u>

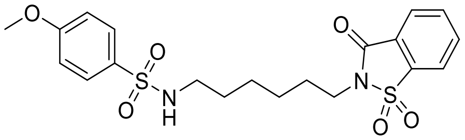

The substrate **2ac** was prepared following the **procedures for preparation of specific compounds** on a 5 mmol scale, which was obtained as white solid in 52% yield.

^{1}H NMR (400 MHz, $CDCl_3$) δ 8.06 – 8.02 (m, 1H), 7.93 – 7.77 (m, 5H), 6.98 – 6.94 (m, 2H), 4.62 (t, *J* = 6.2 Hz, 1H), 3.85 (s, 3H), 3.72 (t, *J* = 7.4 Hz, 2H), 2.91 (q, *J* = 6.7 Hz, 2H), 1.83 – 1.73 (m, 2H), 1.51 – 1.41 (m, 2H), 1.36 – 1.29 (m, 4H).

^{13}C NMR (101 MHz, $CDCl_3$) δ 162.80, 159.02, 137.61, 134.77, 134.37, 131.55, 129.21, 127.35, 125.16, 120.92, 114.23, 55.62, 42.95, 39.12, 29.28, 28.15, 26.07, 25.85.

Analytical data matched that reported in literature.[7]

<u>6-((4-methoxyphenyl)sulfonamido)hexyl 2-(4-isobutylphenyl)propanoate (2ae)</u>

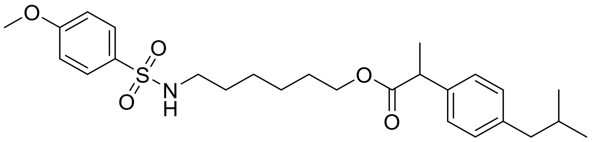

The substrate **2ad** was prepared following **General procedure C** on a 2 mmol scale, which was obtained as colorless oil in 80% yield.

^{1}H NMR (400 MHz, $CDCl_3$) δ 7.82 – 7.77 (m, 2H), 7.20 – 7.15 (m, 2H), 7.09 – 7.05 (m, 2H), 6.99 – 6.95 (m, 2H), 4.55 (br, 1H), 4.00 (t, *J* = 6.6 Hz, 2H), 3.86 (s, 3H), 3.66 (q, *J* = 7.1 Hz, 1H), 2.95 – 2.79 (m, 2H), 2.43 (d, *J* = 7.2 Hz, 2H), 1.83 (dp, *J* = 13.6, 6.8 Hz, 1H), 1.54 – 1.44 (m, 5H), 1.39 (p, *J* = 7.2 Hz, 2H), 1.27 – 1.12 (m, 4H), 0.88 (d, *J* = 6.6 Hz, 6H).

^{13}C NMR (101 MHz, $CDCl_3$) δ 174.75, 162.85, 140.44, 137.86, 131.73, 129.24, 129.18, 127.12, 114.22, 64.36, 55.58, 45.18, 45.01, 42.99, 30.13, 29.41, 28.33, 26.00, 25.22, 22.35, 18.41.

HRMS (ESI+) *m*/*z* Calcd for $C_{26}H_{38}NO_5S^+$: 476.2465 [*M*+H]$^+$; found: 476.2477.

<u>6-((4-methoxyphenyl)sulfonamido)hexyl 4-([1,1'-biphenyl]-4-yl)-4-oxobutanoate (2af)</u>

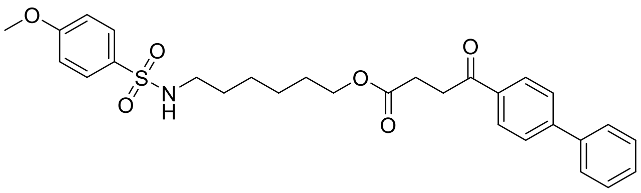

The substrate **2ae** was prepared following **General procedure C** on a 2 mmol scale, which was obtained as colorless oil in 71% yield.

^{1}H NMR (400 MHz, $CDCl_3$) δ 8.09 – 8.01 (m, 2H), 7.82 – 7.76 (m, 2H), 7.71 – 7.66 (m, 2H), 7.64 – 7.60 (m, 2H), 7.50 – 7.36 (m, 3H), 6.99 – 6.93 (m, 2H), 4.82 (t, *J* = 6.2 Hz, 1H), 4.06 (t, *J* = 6.6 Hz, 2H), 3.84 (s, 3H), 3.33 (t, *J* = 6.5 Hz, 2H), 2.90 (q, *J* = 6.5 Hz, 2H), 2.76 (t, *J* = 6.5 Hz, 2H), 1.61 – 1.52 (m, 2H), 1.44 (t, *J* = 7.0 Hz, 2H), 1.33 – 1.23 (m, *J* = 3.9, 3.0 Hz, 4H).

^{13}C NMR (101 MHz, $CDCl_3$) δ 197.88, 173.01, 162.78, 145.88, 139.79, 135.24, 131.62, 129.19, 128.99, 128.67, 128.28, 127.26, 114.22, 64.55, 55.62, 43.03, 33.42, 29.38, 28.39, 28.32, 26.09, 25.40.

HRMS (ESI+) *m/z* Calcd for $C_{29}H_{34}NO_6S^+$: 524.2101 [M+H]$^+$; found: 524.2104.

2-oxo-1-phenyl-2-((3,3,5-trimethylcyclohexyl)oxy)ethyl 7-((4-methoxyphenyl)sulfonamido)heptanoate (2ag)

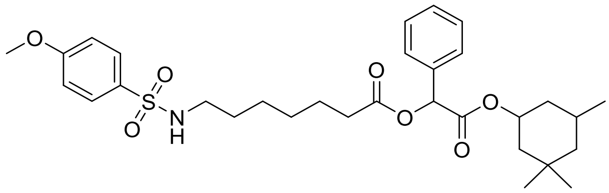

The substrate **2ag** was prepared following **General procedure D** on a 2 mmol scale, which was obtained as colorless oil in 89% yield.

^{1}H NMR (400 MHz, $CDCl_3$) δ 7.83 – 7.75 (m, 2H), 7.48 – 7.41 (m, 2H), 7.41 – 7.34 (m, 3H), 7.01 – 6.92 (m, 2H), 5.84 (d, *J* = 1.6 Hz, 1H), 4.91 (tt, *J* = 11.6, 4.4 Hz, 1H), 4.55 (br, 1H), 3.85 (s, 3H), 2.91 (q, *J* = 6.5 Hz, 2H), 2.50 – 2.31 (m, 2H), 1.89 (dd, *J* = 84.0, 12.1 Hz, 1H), 1.74 – 1.40 (m, 6H), 1.34 – 1.24 (m, 5H), 1.04 (dt, *J* = 61.7, 12.1 Hz, 1H), 0.93 – 0.83 (m, 9H), 0.81 – 0.63 (m, 2H).

^{13}C NMR (101 MHz, $CDCl_3$) δ 173.01, 168.48, 168.46, 162.81, 134.05, 134.04, 131.61, 129.21, 129.09, 128.73, 127.53, 127.51, 114.23, 74.64, 74.62, 72.81, 55.61, 47.42, 43.63, 43.34, 43.03, 40.09, 39.76, 33.77, 32.96, 32.93, 32.29, 32.24, 29.30, 28.40, 27.04, 26.97, 26.10, 25.45, 25.42, 24.55, 22.23, 22.20.

Analytical data matched that reported in literature.[7]

6-((4-methoxyphenyl)sulfonamido)hexyl 2-(4-(4-chlorobenzoyl)phenoxy)-2-methylpropanoate (2ah)

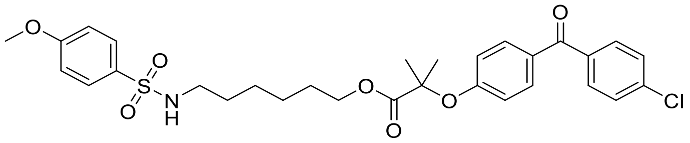

The substrate **2af** was prepared following **General procedure C** on a 1 mmol scale, which was obtained as colorless oil in 82% yield.

^{1}H NMR (400 MHz, $CDCl_3$) δ 7.83 – 7.77 (m, 2H), 7.73 – 7.66 (m, 4H), 7.47 – 7.42 (m, 2H), 6.99

– 6.94 (m, 2H), 6.86 – 6.81 (m, 2H), 4.71 (t, *J* = 5.0 Hz, 1H), 4.11 (t, *J* = 6.4 Hz, 2H), 3.86 (s, 3H), 2.83 (q, *J* = 6.7 Hz, 2H), 1.67 (s, 6H), 1.57 – 1.44 (m, 2H), 1.34 – 1.27 (m, 2H), 1.19 – 1.13 (m, 2H), 1.11 – 1.02 (m, 2H)

^{13}C NMR (101 MHz, $CDCl_3$) δ 194.51, 173.72, 162.77, 159.78, 138.59, 136.18, 132.01, 131.68, 131.27, 130.26, 129.20, 128.60, 116.95, 114.19, 79.43, 65.51, 55.61, 43.06, 29.48, 28.22, 26.05, 25.46, 25.33.

Analytical data matched that reported in literature.[7]

***N*-(6-((4-methoxyphenyl)sulfonamido)hexyl)-*N*-methyl-4-(5-(*p*-tolyl)-3-(trifluoromethyl)-1*H*-pyrazol-1-yl)benzenesulfonamide (2ai)**

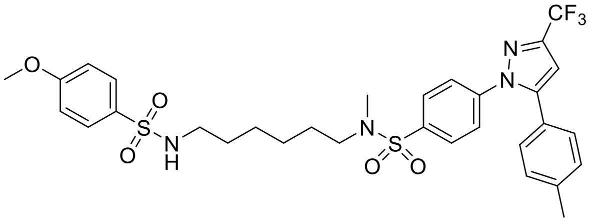

The substrate **2ah** was prepared following the **procedures for preparation of specific compounds** on a 2 mmol scale, which was obtained as colorless oil in 45% yield.

^{1}H NMR (400 MHz, $CDCl_3$) δ 7.83 – 7.72 (m, 4H), 7.51 – 7.43 (m, 2H), 7.16 (d, *J* = 7.9 Hz, 2H), 7.09 (d, *J* = 8.2 Hz, 2H), 7.02 – 6.92 (m, 2H), 6.74 (s, 1H), 4.75 – 4.67 (m, 1H), 3.85 (s, 3H), 2.98 – 2.85 (m, 4H), 2.68 (s, 3H), 2.36 (s, 3H), 1.52 – 1.39 (m, 4H), 1.33 – 1.21 (m, 4H).

^{13}C NMR (101 MHz, $CDCl_3$) δ 162.81, 145.31, 144.08 (q, $J_{F\text{-}C}$ = 38.5 Hz), 142.43, 139.82, 137.15, 131.62, 129.74, 129.18, 128.70, 128.29, 125.61, 121.06 (q, $J_{F\text{-}C}$ = 269.4 Hz), 114.24, 106.21, 55.62, 49.79, 42.88, 34.51, 29.36, 27.20, 25.81, 25.61, 21.31.

^{19}F NMR (376 MHz, $CDCl_3$) δ -62.40.

Analytical data matched that reported in literature.[7]

2-(2-methyl-5-nitro-1*H*-imidazol-1-yl)ethyl 7-((4-methoxyphenyl)sulfonamido)heptanoate (2aj)

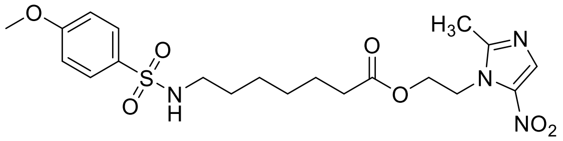

The substrate **2ai** was prepared following **General procedure D** on a 1 mmol scale, which was obtained as yellow solid in 58% yield.

^{1}H NMR (400 MHz, $CDCl_3$) δ 7.89 (s, 1H), 7.78 – 7.73 (m, 2H), 6.97 – 6.91 (m, 2H), 5.12 (t, *J* = 6.1 Hz, 1H), 4.56 (t, *J* = 5.2 Hz, 2H), 4.37 (t, *J* = 5.2 Hz, 2H), 3.84 (s, 3H), 2.85 (q, *J* = 6.7 Hz, 2H), 2.49 (s, 3H), 2.19 (t, *J* = 7.4 Hz, 2H), 1.50 – 1.36 (m, 4H), 1.26 – 1.13 (m, 4H).

^{13}C NMR (101 MHz, $CDCl_3$) δ 172.89, 162.75, 150.98, 138.52, 132.95, 131.57, 129.12, 114.20, 62.30, 55.63, 45.07, 42.95, 33.61, 29.18, 28.30, 26.02, 24.36, 14.37.

HRMS (ESI+) *m*/*z* Calcd for $C_{20}H_{29}N_4O_7S^+$: 469.1751 [*M*+H]$^+$; found: 469.1746.

7.2 Characterization data for products

4-methoxy-*N*-(4-(2-phenylquinolin-4-yl)pentyl)benzenesulfonamide (3)

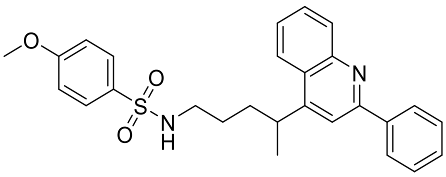

Prepared from 2-phenylquinoline (41.0 mg, 0.2 mmol, 1.0 equiv) and 4-methoxy-*N*-pentylbenzenesulfonamide **2a** (102.8 mg, 0.4 mmol, 2.0 equiv) following **general procedure E**. The product was obtained as colorless oil (79 mg, 86% yield) after silica gel column chromatography using petroleum ether/EtOAc (4:1 to 3:1).

^{1}H NMR (400 MHz, $CDCl_3$) δ 8.21 (d, *J* = 8.3 Hz, 1H), 8.11 (d, *J* = 7.5 Hz, 2H), 8.01 (d, *J* = 8.4 Hz, 1H), 7.73 – 7.66 (m, 4H), 7.55 – 7.42 (m, 4H), 6.86 (d, *J* = 8.4 Hz, 2H), 4.83 (t, *J* = 5.7 Hz, 1H), 3.79 (s, 3H), 3.56 (h, *J* = 7.4 Hz, 1H), 2.89 (q, *J* = 6.9 Hz, 2H), 1.87 – 1.67 (m, 2H), 1.57 – 1.40 (m, 2H), 1.36 (d, *J* = 6.6 Hz, 3H).

^{13}C NMR (101 MHz, $CDCl_3$) δ 162.77, 157.14, 153.74, 148.41, 139.81, 131.39, 130.54, 129.35, 129.33, 129.11, 128.84, 127.66, 126.22, 126.04, 122.75, 115.58, 114.18, 55.58, 43.11, 34.03, 33.21, 27.53, 21.29.

HRMS (ESI+) *m*/*z* Calcd for $C_{27}H_{29}N_2O_3S^+$: 461.1893 [*M*+H]$^+$; found: 461.1896.

4-methoxy-*N*-(4-(2-(*p*-tolyl)quinolin-4-yl)pentyl)benzenesulfonamide (4)

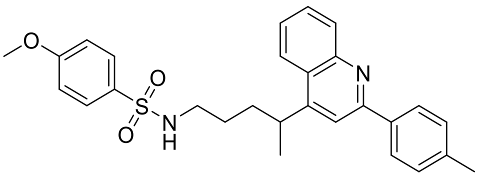

Prepared from 2-(*p*-tolyl)quinoline (43.8 mg, 0.2 mmol, 1.0 equiv) and 4-methoxy-*N*-pentylbenzenesulfonamide **2a** (102.8 mg, 0.4 mmol, 2.0 equiv) following **general procedure E**. The product was obtained as yellow oil (90 mg, 95% yield) after silica gel column chromatography using petroleum ether/EtOAc (4:1 to 3:1).

^{1}H NMR (400 MHz, $CDCl_3$) δ 8.19 (dd, *J* = 8.5, 1.3 Hz, 1H), 8.04 – 8.01 (m, 2H), 7.99 (d, *J* = 8.4 Hz, 1H), 7.74 – 7.64 (m, 4H), 7.49 (ddd, *J* = 8.3, 6.8, 1.3 Hz, 1H), 7.31 (d, *J* = 7.9 Hz, 2H), 6.87 – 6.82 (m, 2H), 5.03 (t, *J* = 6.2 Hz, 1H), 3.77 (s, 3H), 3.52 (h, *J* = 6.9 Hz, 1H), 2.87 (q, *J* = 6.7 Hz, 2H), 2.42 (s, 3H), 1.85 – 1.65 (m, 2H), 1.54 – 1.38 (m, 2H), 1.34 (d, *J* = 6.9 Hz, 3H).

^{13}C NMR (101 MHz, $CDCl_3$) δ 162.76, 157.13, 153.37, 148.60, 139.30, 137.15, 131.43, 130.59, 129.56, 129.16, 129.11, 127.48, 125.96, 122.73, 115.35, 114.18, 55.58, 43.13, 34.02, 33.18, 27.53, 21.38, 21.31.

HRMS (ESI+) *m*/*z* Calcd for $C_{28}H_{31}N_2O_3S^+$: 475.2050 [*M*+H]$^+$; found: 475.2059.

4-methoxy-*N*-(4-(2-(4-methoxyphenyl)quinolin-4-yl)pentyl)benzenesulfonamide (5)

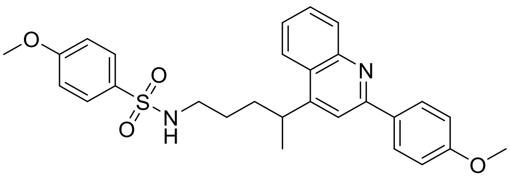

Prepared from 2-(4-methoxyphenyl)quinoline (47.0 mg, 0.2 mmol, 1.0 equiv) and 4-methoxy-*N*-

pentylbenzenesulfonamide **2a** (102.8 mg, 0.4 mmol, 2.0 equiv) following **general procedure E**. The product was obtained as yellow oil (77 mg, 79% yield) after silica gel column chromatography using petroleum ether/EtOAc (4:1 to 2:1).

^{1}H NMR (400 MHz, $CDCl_3$) δ 8.16 (d, *J* = 8.3 Hz, 1H), 8.12 – 8.06 (m, 2H), 7.97 (d, *J* = 8.4 Hz, 1H), 7.73 – 7.64 (m, 3H), 7.62 (s, 1H), 7.47 (ddd, *J* = 8.4, 6.9, 1.3 Hz, 1H), 7.04 – 6.99 (m, 2H), 6.87 – 6.82 (m, 2H), 4.99 (t, *J* = 6.2 Hz, 1H), 3.86 (s, 3H), 3.77 (s, 3H), 3.51 (h, *J* = 7.0 Hz, 1H), 2.87 (q, *J* = 6.7 Hz, 2H), 1.86 – 1.64 (m, 2H), 1.52 – 1.37 (m, 2H), 1.33 (d, *J* = 6.9 Hz, 3H).

^{13}C NMR (101 MHz, $CDCl_3$) δ 162.76, 160.74, 156.70, 153.32, 148.56, 132.48, 131.41, 130.42, 129.16, 129.10, 128.92, 125.79, 125.78, 122.71, 115.04, 114.20, 114.18, 55.57, 55.42, 43.14, 34.01, 33.18, 27.54, 21.31.

HRMS (ESI+) *m/z* Calcd for $C_{28}H_{31}N_2O_4S^+$: 491.1999 [*M*+H]$^+$; found: 491.1996.

***N*-(4-(2-(4-fluorophenyl)quinolin-4-yl)pentyl)-4-methoxybenzenesulfonamide (6)**

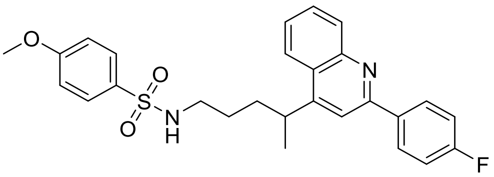

Prepared from 2-(4-fluorophenyl)quinoline (44.6 mg, 0.2 mmol, 1.0 equiv) and 4-methoxy-*N*-pentylbenzenesulfonamide **2a** (102.8 mg, 0.4 mmol, 2.0 equiv) following **general procedure E**. The product was obtained as yellow oil (80 mg, 84% yield) after silica gel column chromatography using petroleum ether/EtOAc (4:1 to 3:1).

^{1}H NMR (400 MHz, $CDCl_3$) δ 8.16 (d, *J* = 8.4 Hz, 1H), 8.12 (dd, *J* = 8.1, 5.6 Hz, 2H), 8.00 (d, *J* = 8.5 Hz, 1H), 6.73 – 6.66 (m, 3H), 7.62 (s, 1H), 7.54 – 7.47 (m, 1H), 7.20 – 7.14 (m, 2H), 6.88 – 6.82 (m, 2H), 5.07 (t, *J* = 6.2 Hz, 1H), 3.78 (s, 3H), 3.53 (h, *J* = 7.0 Hz, 1H), 2.88 (q, *J* = 6.6 Hz, 2H), 1.86 – 1.67 (m, 2H), 1.55 – 1.38 (m, 2H), 1.34 (d, *J* = 6.8 Hz, 3H).

^{19}F NMR (376 MHz, $CDCl_3$) δ -112.54.

^{13}C NMR (101 MHz, $CDCl_3$) δ 163.72 (d, J_{F-C} = 248.9 Hz), 162.79, 156.03, 153.77, 148.48, 136.05 (d, J_{F-C} = 3.3 Hz), 131.37, 130.54, 129.47 (d, J_{F-C} = 8.4 Hz), 129.35, 129.09, 126.21, 125.93, 122.75, 115.71 (d, J_{F-C} = 21.3 Hz), 115.13, 114.19, 55.57, 43.12, 34.00, 33.24, 27.55, 21.30.

HRMS (ESI+) *m/z* Calcd for $C_{27}H_{28}FN_2O_3S^+$: 479.1799 [*M*+H]$^+$; found: 479.1791.

***N*-(4-(2-(4-chlorophenyl)quinolin-4-yl)pentyl)-4-methoxybenzenesulfonamide (7)**

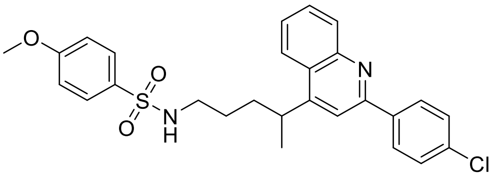

Prepared from 2-(4-chlorophenyl)quinoline (47.8 mg, 0.2 mmol, 1.0 equiv) and 4-methoxy-*N*-pentylbenzenesulfonamide **2a** (102.8 mg, 0.4 mmol, 2.0 equiv) following **general procedure E**. The product was obtained as white solid (75 mg, 76% yield) after silica gel column chromatography using petroleum ether/EtOAc (4:1 to 3:1).

^{1}H NMR (400 MHz, $CDCl_3$) δ 8.17 (dd, *J* = 8.4, 1.3 Hz, 1H), 8.09 – 8.05 (m, 2H), 8.00 (dd, *J* = 8.6, 1.3 Hz, 1H), 7.73 – 7.66 (m, 3H), 7.63 (s, 1H), 7.51 (ddd, *J* = 8.3, 6.8, 1.3 Hz, 1H), 7.47 – 7.43 (m,

2H), 6.87 – 6.82 (m, 2H), 5.10 (t, *J* = 6.2 Hz, 1H), 3.78 (s, 3H), 3.53 (h, *J* = 6.9 Hz, 1H), 2.88 (q, *J* = 6.7 Hz, 2H), 1.88 – 1.66 (m, 2H), 1.56 – 1.36 (m, 2H), 1.34 (d, *J* = 6.8 Hz, 3H).
^{13}C NMR (101 MHz, $CDCl_3$) δ 162.78, 155.79, 153.88, 148.48, 138.29, 135.39, 131.36, 130.60, 129.41, 129.09, 128.95, 128.90, 126.36, 126.08, 122.78, 115.07, 114.19, 55.58, 43.11, 33.98, 33.27, 27.55, 21.31.
HRMS (ESI+) *m*/*z* Calcd for $C_{27}H_{28}ClN_2O_3S^+$: 495.1504 [*M*+H]$^+$; found: 495.1516.

***N*-(4-(2-([1,1'-biphenyl]-4-yl)quinolin-4-yl)pentyl)-4-methoxybenzenesulfonamide (8)**

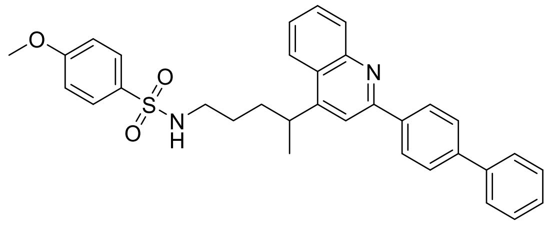

Prepared from 2-([1,1'-biphenyl]-4-yl)quinoline (56.2 mg, 0.2 mmol, 1.0 equiv) and 4-methoxy-*N*-pentylbenzenesulfonamide **2a** (102.8 mg, 0.4 mmol, 2.0 equiv) following **general procedure E**. The product was obtained as colorless oil (41 mg, 38% yield) after silica gel column chromatography using petroleum ether/EtOAc (4:1 to 3:1).
^{1}H NMR (400 MHz, $CDCl_3$) δ 8.26 – 8.18 (m, 3H), 8.02 (dd, *J* = 8.6, 1.3 Hz, 1H), 7.77 – 7.65 (m, 8H), 7.56 – 7.45 (m, 3H), 7.41 – 7.36 (m, 1H), 6.89 – 6.84 (m, 2H), 4.78 (t, *J* = 6.2 Hz, 1H), 3.79 (s, 3H), 3.57 (h, *J* = 6.8 Hz, 1H), 2.91 (q, *J* = 6.7 Hz, 2H), 1.90 – 1.70 (m, 2H), 1.58 – 1.42 (m, 2H), 1.39 (d, *J* = 6.8 Hz, 3H).
^{13}C NMR (101 MHz, $CDCl_3$) δ 162.80, 156.68, 153.57, 148.59, 142.00, 140.57, 138.75, 131.40, 130.65, 129.33, 129.13, 128.89, 128.03, 127.63, 127.53, 127.14, 126.20, 126.08, 122.73, 115.40, 114.20, 55.58, 43.16, 34.07, 33.24, 27.60, 21.37.
HRMS (ESI+) *m*/*z* Calcd for $C_{33}H_{33}N_2O_3S^+$: 537.2206 [*M*+H]$^+$; found: 537.2191.

4-methoxy-*N*-(4-(4-methylquinolin-2-yl)pentyl)benzenesulfonamide (9)

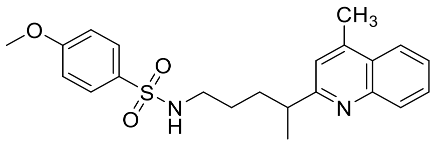

Prepared from 4-methylquinoline (28.6 mg, 0.2 mmol, 1.0 equiv) and 4-methoxy-*N*-pentylbenzenesulfonamide **2a** (102.8 mg, 0.4 mmol, 2.0 equiv) following **general procedure E**. The product was obtained as yellow oil (61 mg, 77% yield) after silica gel column chromatography using petroleum ether/EtOAc (4:1 to 2:1).
^{1}H NMR (600 MHz, $CDCl_3$) δ 8.08 (dd, *J* = 8.4, 1.2 Hz, 1H), 7.93 (dd, *J* = 8.3, 1.4 Hz, 1H), 7.74 – 7.70 (m, 2H), 7.67 (ddd, *J* = 8.3, 6.8, 1.4 Hz, 1H), 7.50 (ddd, *J* = 8.2, 6.8, 1.2 Hz, 1H), 7.07 (s, 1H), 6.88 – 6.84 (m, 2H), 5.62 (t, *J* = 4.7 Hz, 1H), 3.80 (s, 3H), 2.95 (ddt, *J* = 18.9, 12.9, 6.7 Hz, 2H), 2.86 (dq, *J* = 12.3, 6.5 Hz, 1H), 2.65 (s, 3H), 1.81 (dtd, *J* = 14.1, 9.1, 5.2 Hz, 1H), 1.63 (ddt, *J* = 13.7, 9.3, 6.3 Hz, 1H), 1.44 (ddq, *J* = 13.5, 9.0, 6.7 Hz, 1H), 1.40 – 1.32 (m, 1H), 1.27 (d, *J* = 7.0 Hz, 3H).
^{13}C NMR (151 MHz, $CDCl_3$) δ 165.61, 162.61, 147.21, 144.92, 131.69, 129.33, 129.25, 129.14, 127.02, 125.71, 123.61, 120.27, 114.08, 55.56, 43.11, 41.71, 33.74, 27.11, 21.08, 18.86.

HRMS (ESI+) *m/z* Calcd for $C_{22}H_{27}N_2O_3S^+$: 399.1737 $[M+H]^+$; found: 399.1738.

***N*-(4-(4-chloroquinolin-2-yl)pentyl)-4-methoxybenzenesulfonamide (10)**

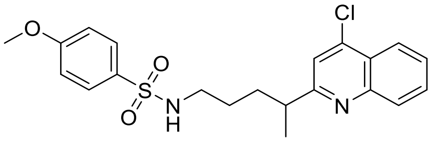

Prepared from 4-chloroquinoline (32.6 mg, 0.2 mmol, 1.0 equiv) and 4-methoxy-*N*-pentylbenzenesulfonamide **2a** (102.8 mg, 0.4 mmol, 2.0 equiv) following **general procedure E**. The product was obtained as yellow oil (61 mg, 73% yield) after silica gel column chromatography using petroleum ether/EtOAc (5:1 to 3:1).

^{1}H NMR (400 MHz, $CDCl_3$) δ 8.17 (d, *J* = 8.3 Hz, 1H), 8.09 (d, *J* = 8.4 Hz, 1H), 7.78 – 7.70 (m, 3H), 7.59 (t, *J* = 7.5 Hz, 1H), 7.33 (s, 1H), 6.88 (d, *J* = 8.3 Hz, 2H), 5.28 – 5.23 (m, 1H), 3.81 (s, 3H), 3.02 – 2.83 (m, 3H), 1.87 – 1.76 (m, 1H), 1.72 – 1.58 (m, 1H), 1.54 – 1.32 (m, 2H), 1.29 (d, *J* = 6.8 Hz, 3H).

^{13}C NMR (101 MHz, $CDCl_3$) δ 165.98, 162.69, 148.36, 143.05, 131.56, 130.51, 129.24, 129.14, 126.95, 125.14, 123.93, 119.84, 114.12, 55.58, 43.05, 41.81, 33.53, 27.16, 20.92.

HRMS (ESI+) *m/z* Calcd for $C_{21}H_{24}ClN_2O_3S^+$: 419.1191 $[M+H]^+$; found: 419.1195.

***N*-(4-(4-bromoquinolin-2-yl)pentyl)-4-methoxybenzenesulfonamide (11)**

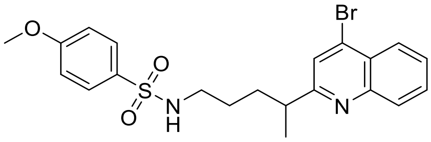

Prepared from 4-bromoquinoline (41.4 mg, 0.2 mmol, 1.0 equiv) and 4-methoxy-*N*-pentylbenzenesulfonamide **2a** (102.8 mg, 0.4 mmol, 2.0 equiv) following **general procedure E**. The product was obtained as yellow oil (60 mg, 65% yield) after silica gel column chromatography using petroleum ether/EtOAc (5:1 to 2:1).

^{1}H NMR (400 MHz, $CDCl_3$) δ 8.11 (d, *J* = 8.3 Hz, 1H), 8.05 (d, *J* = 8.4 Hz, 1H), 7.77 – 7.66 (m, 3H), 7.57 (t, *J* = 7.4 Hz, 1H), 7.52 (s, 1H), 6.90 – 6.83 (m, 2H), 5.37 (t, *J* = 6.0 Hz, 1H), 3.79 (s, 3H), 3.02 – 2.81 (m, 3H), 1.86 – 1.75 (m, 1H), 1.69 – 1.56 (m, 1H), 1.53 – 1.31 (m, 2H), 1.28 (d, *J* = 6.8 Hz, 3H).

^{13}C NMR (101 MHz, $CDCl_3$) δ 165.97, 162.67, 148.09, 134.65, 131.55, 130.50, 129.26, 129.14, 127.20, 126.57, 126.48, 123.73, 114.12, 55.58, 43.04, 41.67, 33.51, 27.20, 20.88.

HRMS (ESI+) *m/z* Calcd for $C_{21}H_{24}BrN_2O_3S^+$: 463.0686 $[M+H]^+$; found: 463.0689.

4-methoxy-*N*-(4-(2-methylquinolin-4-yl)pentyl)benzenesulfonamide (12)

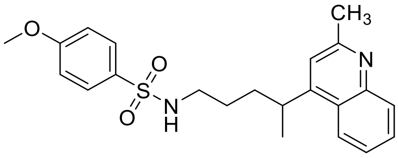

Prepared from 2-methylquinoline (28.6 mg, 0.2 mmol, 1.0 equiv) and 4-methoxy-*N*-pentylbenzenesulfonamide **2a** (102.8 mg, 0.4 mmol, 2.0 equiv) following **general procedure E**. The product was obtained as yellow oil (59 mg, 74% yield) after silica gel column chromatography

using petroleum ether/EtOAc (3:1 to 2:1).

^{1}H NMR (400 MHz, $CDCl_3$) δ 7.99 (d, *J* = 8.3 Hz, 1H), 7.94 (d, *J* = 8.4 Hz, 1H), 7.74 – 7.68 (m, 2H), 7.61 (ddd, *J* = 8.4, 6.8, 1.5 Hz, 1H), 7.44 (ddd, *J* = 8.3, 6.8, 1.4 Hz, 1H), 7.07 (s, 1H), 6.89 – 6.84 (m, 2H), 5.17 (t, *J* = 6.5 Hz, 1H), 3.80 (s, 3H), 3.45 (h, *J* = 6.6 Hz, 1H), 2.88 (q, *J* = 6.7 Hz, 2H), 2.66 (s, 3H), 1.81 – 1.58 (m, 2H), 1.52 – 1.34 (m, 2H), 1.27 (d, *J* = 6.8 Hz, 3H).

^{13}C NMR (101 MHz, $CDCl_3$) δ 162.76, 158.68, 153.06, 147.90, 131.42, 129.30, 129.10, 129.04, 125.56, 125.31, 122.70, 118.44, 114.16, 55.60, 43.10, 33.97, 32.86, 27.50, 25.33, 21.20.

HRMS (ESI+) *m/z* Calcd for $C_{22}H_{27}N_2O_3S^+$: 399.1737 [*M*+H]$^+$; found: 399.1743.

<u>*N*-(4-(4,7-dichloroquinolin-2-yl)pentyl)-4-methoxybenzenesulfonamide (13)</u>

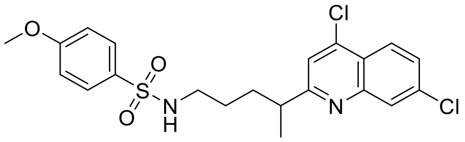

Prepared from 4,7-dichloroquinoline (39.4 mg, 0.2 mmol, 1.0 equiv) and 4-methoxy-*N*-pentylbenzenesulfonamide **2a** (102.8 mg, 0.4 mmol, 2.0 equiv) following **general procedure E**. The product was obtained as yellow oil (39 mg, 43% yield) after silica gel column chromatography using petroleum ether/EtOAc (4:1 to 2:1).

^{1}H NMR (400 MHz, $CDCl_3$) δ 8.10 (d, *J* = 8.9 Hz, 1H), 8.07 (s, 1H), 7.77 – 7.71 (m, 2H), 7.53 (dd, *J* = 8.9, 2.1 Hz, 1H), 7.32 (s, 1H), 6.94 – 6.87 (m, 2H), 4.95 (t, *J* = 6.0 Hz, 1H), 3.83 (s, 3H), 3.02 – 2.85 (m, 3H), 1.86 – 1.75 (m, 1H), 1.70 – 1.58 (m, 1H), 1.51 – 1.33 (m, 2H), 1.29 (d, *J* = 6.7 Hz, 3H).

^{13}C NMR (101 MHz, $CDCl_3$) δ 167.38, 162.75, 148.77, 142.97, 136.53, 131.52, 129.14, 128.23, 127.88, 125.37, 123.65, 120.18, 114.15, 55.59, 43.05, 41.90, 33.42, 27.30, 20.75.

HRMS (ESI+) *m/z* Calcd for $C_{21}H_{23}Cl_2N_2O_3S^+$: 453.0801 [*M*+H]$^+$; found: 453.0793.

<u>*N*-(4-(6-fluorobenzo[*d*]thiazol-2-yl)pentyl)-4-methoxybenzenesulfonamide (14)</u>

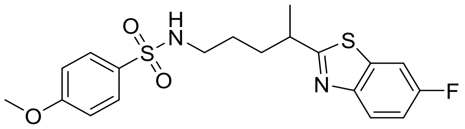

Prepared from 6-fluorobenzo[*d*]thiazole (30.6 mg, 0.2 mmol, 1.0 equiv) and 4-methoxy-*N*-pentylbenzenesulfonamide **2a** (102.8 mg, 0.4 mmol, 2.0 equiv) following **general procedure E**. The product was obtained as yellow oil (42 mg, 52% yield) after silica gel column chromatography using petroleum ether/EtOAc (4:1 to 3:1).

^{1}H NMR (400 MHz, $CDCl_3$) δ 7.91 (dd, *J* = 9.0, 4.7 Hz, 1H), 7.75 (d, *J* = 8.7 Hz, 2H), 7.51 (d, *J* = 7.9 Hz, 1H), 7.18 (t, *J* = 8.7 Hz, 1H), 6.91 (d, *J* = 8.5 Hz, 2H), 4.98 (br, 1H), 3.83 (s, 3H), 3.20 (q, *J* = 7.0 Hz, 1H), 2.93 (t, *J* = 6.8 Hz, 2H), 1.92 – 1.65 (m, 2H), 1.61 – 1.42 (m, 2H), 1.39 (d, *J* = 6.5 Hz, 3H).

^{19}F NMR (376 MHz, $CDCl_3$) δ -116.51.

^{13}C NMR (101 MHz, $CDCl_3$) δ 176.86, 162.79, 160.24 (d, $J_{F\text{-}C}$ = 245.1 Hz), 149.27 (d, $J_{F\text{-}C}$ = 2.0 Hz), 135.46 (d, $J_{F\text{-}C}$ = 1.3 Hz), 131.47, 129.17, 123.48 (d, $J_{F\text{-}C}$ = 9.4 Hz), 114.61 (d, $J_{F\text{-}C}$ = 24.6 Hz), 114.20, 107.84 (d, $J_{F\text{-}C}$ = 26.7 Hz), 55.60, 42.91, 38.82, 33.91, 27.05, 21.26.

HRMS (ESI+) *m/z* Calcd for $C_{19}H_{22}FN_2O_3S_2^+$: 409.1050 [*M*+H]$^+$; found: 409.1055.

***N*-(4-(6-chlorobenzo[*d*]thiazol-2-yl)pentyl)-4-methoxybenzenesulfonamide (15)**

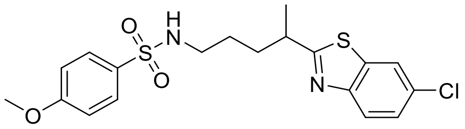

Prepared from 6-chlorobenzo[*d*]thiazole (33.8 mg, 0.2 mmol, 1.0 equiv) and 4-methoxy-*N*-pentylbenzenesulfonamide **2a** (102.8 mg, 0.4 mmol, 2.0 equiv) following **general procedure E**. The product was obtained as yellow oil (54 mg, 64% yield) after silica gel column chromatography using petroleum ether/EtOAc (4:1 to 3:1).

^{1}H NMR (400 MHz, $CDCl_3$) δ 7.83 (d, *J* = 8.7 Hz, 1H), 7.77 (d, *J* = 2.1 Hz, 1H), 7.76 – 7.72 (m, 2H), 7.38 (dd, *J* = 8.7, 2.1 Hz, 1H), 6.91 – 6.87 (m, 2H), 5.15 (br, 1H), 3.81 (s, 3H), 3.18 (h, *J* = 6.9 Hz, 1H), 2.97 – 2.86 (m, 2H), 1.88 – 1.65 (m, 2H), 1.60 – 1.40 (m, 2H), 1.36 (d, *J* = 6.9 Hz, 3H).

^{13}C NMR (101 MHz, $CDCl_3$) δ 177.74, 162.77, 151.34, 135.77, 131.44, 130.66, 129.16, 126.74, 123.33, 121.22, 114.20, 55.61, 42.90, 38.85, 33.97, 27.06, 21.20.

HRMS (ESI+) *m*/*z* Calcd for $C_{19}H_{22}ClN_2O_3S_2^+$: 425.0755 [*M*+H]$^+$; found: 425.0764.

***N*-(4-(6-bromobenzo[*d*]thiazol-2-yl)pentyl)-4-methoxybenzenesulfonamide (16)**

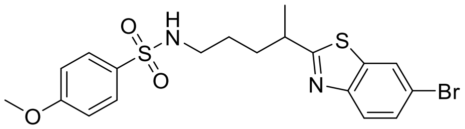

Prepared from 6-bromobenzo[*d*]thiazole (42.6 mg, 0.2 mmol, 1.0 equiv) and 4-methoxy-*N*-pentylbenzenesulfonamide **2a** (102.8 mg, 0.4 mmol, 2.0 equiv) following **general procedure E**. The product was obtained as yellow oil (57 mg, 61% yield) after silica gel column chromatography using petroleum ether/EtOAc (4:1 to 3:1).

^{1}H NMR (400 MHz, $CDCl_3$) δ 7.95 (s, 1H), 7.79 (d, *J* = 8.6 Hz, 1H), 7.74 (d, *J* = 8.5 Hz, 2H), 7.53 (dt, *J* = 8.7, 1.8 Hz, 1H), 6.90 (d, *J* = 8.5 Hz, 2H), 5.06 (br, 1H), 3.82 (s, 3H), 3.20 (h, *J* = 7.1 Hz, 1H), 2.92 (t, *J* = 6.8 Hz, 2H), 1.90 – 1.66 (m, 2H), 1.61 – 1.41 (m, 2H), 1.38 (d, *J* = 6.8 Hz, 3H).

^{13}C NMR (101 MHz, $CDCl_3$) δ 177.82, 162.78, 151.54, 136.19, 131.45, 129.51, 129.16, 124.16, 123.70, 118.39, 114.20, 55.62, 42.88, 38.81, 33.94, 27.07, 21.21.

HRMS (ESI+) *m*/*z* Calcd for $C_{19}H_{22}BrN_2O_3S_2^+$: 469.0250 [*M*+H]$^+$; found: 469.0237.

4-methoxy-*N*-(4-(6-(trifluoromethyl)benzo[*d*]thiazol-2-yl)pentyl)benzenesulfonamide (17)

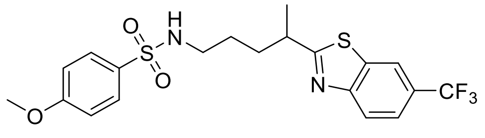

Prepared from 6-(trifluoromethyl)benzo[*d*]thiazole (40.6 mg, 0.2 mmol, 1.0 equiv) and 4-methoxy-*N*-pentylbenzenesulfonamide **2a** (102.8 mg, 0.4 mmol, 2.0 equiv) following **general procedure E**. The product was obtained as yellow oil (50 mg, 55% yield) after silica gel column chromatography using petroleum ether/EtOAc (4:1 to 3:1).

^{1}H NMR (400 MHz, $CDCl_3$) δ 8.12 (s, 1H), 8.03 (d, *J* = 8.5 Hz, 1H), 7.75 (d, *J* = 8.1 Hz, 2H), 7.68 (d, *J* = 8.5 Hz, 1H), 6.91 (d, *J* = 8.2 Hz, 2H), 5.07 (br, 1H), 3.82 (s, 3H), 3.25 (h, *J* = 7.1 Hz, 1H), 2.93 (t, *J* = 6.9 Hz, 2H), 1.92 – 1.69 (m, 2H), 1.61 – 1.45 (m, 2H), 1.40 (d, *J* = 6.8 Hz, 3H).

^{19}**F NMR** (376 MHz, $CDCl_3$) δ -61.35.
^{13}**C NMR** (101 MHz, $CDCl_3$) δ 180.54, 162.81, 154.76, 134.68, 131.42, 129.16, 127.02 (q, J_{F-C} = 32.5 Hz), 124.18 (q, J_{F-C} = 272.5 Hz), 123.02, (q, J_{F-C} = 3.4 Hz), 122.96, 119.29 (q, J_{F-C} = 4.1 Hz), 114.20, 55.58, 42.87, 39.04, 33.97, 27.09, 21.18.
HRMS (ESI+) *m/z* Calcd for $C_{20}H_{22}F_3N_2O_3S_2^+$: 459.1018 [*M*+H]$^+$; found:459.1007.

4-methoxy-*N*-(4-(6-(trifluoromethoxy)benzo[*d*]thiazol-2-yl)pentyl)benzenesulfonamide (18)

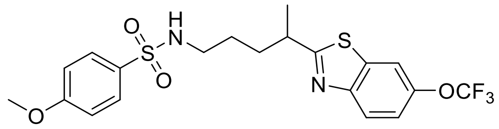

Prepared from 6-(trifluoromethoxy)benzo[*d*]thiazole (43.8 mg, 0.2 mmol, 1.0 equiv) and 4-methoxy-*N*-pentylbenzenesulfonamide **2a** (102.8 mg, 0.4 mmol, 2.0 equiv) following **general procedure E**. The product was obtained as yellow oil (57 mg, 60% yield) after silica gel column chromatography using petroleum ether/EtOAc (4:1 to 3:1).
^{1}**H NMR** (400 MHz, $CDCl_3$) δ 7.95 (d, *J* = 8.8 Hz, 1H), 7.76 (d, *J* = 7.6 Hz, 2H), 7.69 (s, 1H), 7.31 (d, *J* = 8.8 Hz, 1H), 6.91 (d, *J* = 7.6 Hz, 2H), 5.04 (br, 1H), 3.82 (s, 3H), 3.27 – 3.16 (m, 1H), 2.98 – 2.88 (m, 2H), 1.91 – 1.67 (m, 2H), 1.60 – 1.42 (m, 2H), 1.39 (d, *J* = 6.6 Hz, 3H).
^{19}**F NMR** (376 MHz, $CDCl_3$) δ -58.04.
^{13}**C NMR** (101 MHz, $CDCl_3$) δ 178.48, 162.80, 151.23, 146.19, 135.36, 131.45, 129.16, 123.39, 120.52 (q, *J* = 257.4 Hz), 119.97, 114.27, 114.20, 55.58, 42.88, 38.90, 33.94, 27.06, 21.23.
HRMS (ESI+) *m/z* Calcd for $C_{20}H_{22}F_3N_2O_4S_2^+$: 475.0968 [*M*+H]$^+$; found: 475.0974.

ethyl 2-(5-((4-methoxyphenyl)sulfonamido)pentan-2-yl)benzo[*d*]thiazole-6-carboxylate (19)

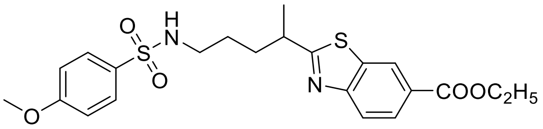

Prepared from ethyl benzo[*d*]thiazole-6-carboxylate (41.4 mg, 0.2 mmol, 1.0 equiv) and 4-methoxy-*N*-pentylbenzenesulfonamide **2a** (102.8 mg, 0.4 mmol, 2.0 equiv) following **general procedure E**. The product was obtained as yellow oil (49 mg, 53% yield) after silica gel column chromatography using petroleum ether/EtOAc (3:1 to 2:1).
^{1}**H NMR** (400 MHz, $CDCl_3$) δ 8.55 (d, *J* = 1.5 Hz, 1H), 8.12 (dd, *J* = 8.5, 1.7 Hz, 1H), 7.97 (d, *J* = 8.5 Hz, 1H), 7.77 – 7.72 (m, 2H), 6.93 – 6.88 (m, 2H), 4.99 (br, 1H), 4.41 (q, *J* = 7.1 Hz, 2H), 3.82 (s, 3H), 3.25 (h, *J* = 7.0 Hz, 1H), 2.94 (t, *J* = 6.8 Hz, 2H), 1.92 – 1.70 (m, 2H), 1.62 – 1.45 (m, 2H), 1.45 – 1.36 (m, 6H).
^{13}**C NMR** (101 MHz, $CDCl_3$) δ 180.91, 166.17, 162.79, 155.57, 134.43, 131.46, 129.15, 127.28, 127.04, 123.78, 122.24, 114.20, 61.31, 55.60, 42.90, 39.08, 33.98, 27.11, 21.18, 14.37.
HRMS (ESI+) *m/z* Calcd for $C_{22}H_{27}N_2O_5S_2^+$: 463.1356 [*M*+H]$^+$; found: 463.1371.

***N*-(4-(5-chlorobenzo[*d*]thiazol-2-yl)pentyl)-4-methoxybenzenesulfonamide (20)**

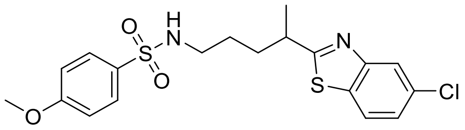

Prepared from 5-chlorobenzo[*d*]thiazole (33.8 mg, 0.2 mmol, 1.0 equiv) and 4-methoxy-*N*-pentylbenzenesulfonamide **2a** (102.8 mg, 0.4 mmol, 2.0 equiv) following **general procedure E**. The product was obtained as colorless oil (53 mg, 63% yield) after silica gel column chromatography using petroleum ether/EtOAc (4:1 to 3:1).

^{1}H NMR (400 MHz, $CDCl_3$) δ 7.90 (s, 1H), 7.77 – 7.70 (m, 3H), 7.31 (d, *J* = 8.5 Hz, 1H), 6.90 (d, *J* = 8.4 Hz, 2H), 5.03 (br, 1H), 3.83 (s, 3H), 3.20 (h, *J* = 7.0 Hz, 1H), 2.95 – 2.89 (m, 2H), 1.88 – 1.67 (m, 2H), 1.60 – 1.41 (m, 2H), 1.38 (d, *J* = 6.8 Hz, 3H).

^{13}C NMR (101 MHz, $CDCl_3$) δ 179.24, 162.79, 153.62, 132.80, 131.99, 131.45, 129.15, 125.29, 122.45, 122.34, 114.20, 55.61, 42.90, 38.93, 34.05, 27.12, 21.23.

HRMS (ESI+) *m/z* Calcd for $C_{19}H_{22}ClN_2O_3S_2^+$: 425.0755 [*M*+H]$^+$; found: 425.0769.

<u>*N*-(4-(5-bromobenzo[*d*]thiazol-2-yl)pentyl)-4-methoxybenzenesulfonamide (21)</u>

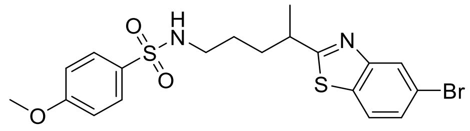

Prepared from 5-bromobenzo[*d*]thiazole (42.6 mg, 0.2 mmol, 1.0 equiv) and 4-methoxy-*N*-pentylbenzenesulfonamide **2a** (102.8 mg, 0.4 mmol, 2.0 equiv) following **general procedure E**. The product was obtained as colorless oil (69 mg, 74% yield) after silica gel column chromatography using petroleum ether/EtOAc (4:1 to 3:1).

^{1}H NMR (400 MHz, $CDCl_3$) δ 8.05 (s, 1H), 7.74 (d, *J* = 8.4 Hz, 2H), 7.67 (d, *J* = 8.5 Hz, 1H), 7.43 (d, *J* = 8.4 Hz, 1H), 6.90 (d, *J* = 8.4 Hz, 2H), 5.09 (br, 1H), 3.82 (s, 3H), 3.20 (h, *J* = 7.0 Hz, 1H), 2.95 – 2.88 (m, 2H), 1.87 – 1.66 (m, 2H), 1.59 – 1.40 (m, 2H), 1.37 (d, *J* = 6.9 Hz, 3H).

^{13}C NMR (101 MHz, $CDCl_3$) δ 179.07, 162.79, 153.93, 133.35, 131.44, 129.14, 127.90, 125.47, 122.70, 119.56, 114.21, 55.62, 42.90, 38.90, 34.06, 27.11, 21.22.

HRMS (ESI+) *m/z* Calcd for $C_{19}H_{22}BrN_2O_3S_2^+$: 469.0250 [*M*+H]$^+$; found: 469.0252.

<u>*N*-(4-(5,6-dimethylbenzo[*d*]thiazol-2-yl)pentyl)-4-methoxybenzenesulfonamide (22)</u>

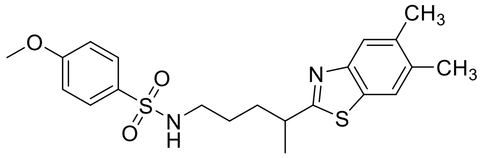

Prepared from 5,6-dimethylbenzo[*d*]thiazole (32.6 mg, 0.2 mmol, 1.0 equiv) and 4-methoxy-*N*-pentylbenzenesulfonamide **2a** (102.8 mg, 0.4 mmol, 2.0 equiv) following **general procedure E**. The product was obtained as yellow oil (40 mg, 48% yield) after silica gel column chromatography using petroleum ether/EtOAc (4:1 to 3:1).

^{1}H NMR (400 MHz, $CDCl_3$) δ 7.76 (s, 1H), 7.73 (d, *J* = 5.9 Hz, 2H), 7.57 (s, 1H), 6.90 (d, *J* = 8.5 Hz, 2H), 4.98 (t, *J* = 6.1 Hz, 1H), 3.83 (s, 3H), 3.18 (h, *J* = 6.9 Hz, 1H), 2.97 – 2.88 (m, 2H), 2.36 (s, 6H), 1.87 – 1.66 (m, 2H), 1.58 – 1.40 (m, 2H), 1.37 (d, *J* = 6.8 Hz, 3H).

^{13}C NMR (101 MHz, $CDCl_3$) δ 175.91, 162.74, 151.49, 135.19, 134.21, 131.86, 131.55, 129.17, 122.80, 121.52, 114.16, 55.57, 42.95, 38.72, 34.06, 27.03, 21.43, 20.19, 20.11.

HRMS (ESI+) *m/z* Calcd for $C_{21}H_{27}N_2O_3S_2^+$: 419.1458 [*M*+H]$^+$; found: 419.1469.

<u>*N*-(4-(benzo[*d*]thiazol-2-yl)pentyl)-4-methoxybenzenesulfonamide (23)</u>

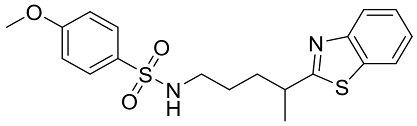

Prepared from benzo[*d*]thiazole (27.0 mg, 0.2 mmol, 1.0 equiv) and 4-methoxy-*N*-pentylbenzenesulfonamide **2a** (102.8 mg, 0.4 mmol, 2.0 equiv) following **general procedure E**. The product was obtained as colorless oil (44 mg, 57% yield) after silica gel column chromatography using petroleum ether/EtOAc (4:1 to 3:1).

^{1}H NMR (400 MHz, $CDCl_3$) δ 7.95 (d, *J* = 8.1 Hz, 1H), 7.82 (d, *J* = 8.0 Hz, 1H), 7.75 (d, *J* = 8.1 Hz, 2H), 7.44 (t, *J* = 7.6 Hz, 1H), 7.34 (t, *J* = 7.6 Hz, 1H), 6.89 (d, *J* = 8.2 Hz, 2H), 5.15 (t, *J* = 5.8 Hz, 1H), 3.81 (s, 3H), 3.21 (h, *J* = 7.1 Hz, 1H), 2.92 (q, *J* = 6.6 Hz, 2H), 1.89 – 1.66 (m, 2H), 1.60 – 1.42 (m, 2H), 1.38 (d, *J* = 6.8 Hz, 3H).

^{13}C NMR (101 MHz, $CDCl_3$) δ 177.20, 162.75, 152.69, 134.50, 131.50, 129.16, 126.02, 124.83, 122.58, 121.63, 114.19, 55.60, 42.93, 38.83, 34.04, 27.07, 21.37.

HRMS (ESI+) *m*/*z* Calcd for $C_{19}H_{23}N_2O_3S_2^+$: 391.1145 [*M*+H]$^+$; found: 391.1148.

***N*-(4-(isoquinolin-1-yl)pentyl)-4-methoxybenzenesulfonamide (24)**

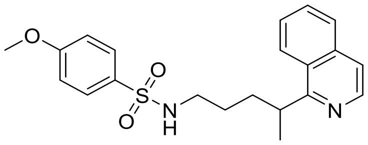

Prepared from isoquinoline (25.8 mg, 0.2 mmol, 1.0 equiv) and 4-methoxy-*N*-pentylbenzenesulfonamide **2a** (102.8 mg, 0.4 mmol, 2.0 equiv) following **general procedure E**. The product was obtained as yellow oil (40 mg, 52% yield) after silica gel column chromatography using petroleum ether/EtOAc (3:1 to 2:1).

^{1}H NMR (400 MHz, $CDCl_3$) δ 8.46 (d, *J* = 5.5 Hz, 1H), 8.14 (d, *J* = 8.4 Hz, 1H), 7.81 (d, *J* = 8.1 Hz, 1H), 7.73 (d, *J* = 8.6 Hz, 2H), 7.62 (dt, *J* = 32.6, 7.2 Hz, 2H), 7.49 (d, *J* = 5.4 Hz, 1H), 6.89 (d, *J* = 8.6 Hz, 2H), 5.09 (t, *J* = 5.9 Hz, 1H), 3.83 (s, 3H), 3.72 (h, *J* = 6.9 Hz, 1H), 2.88 (q, *J* = 6.3 Hz, 2H), 2.11 – 1.95 (m, 1H), 1.75 – 1.64 (m, 1H), 1.57 – 1.44 (m, 1H), 1.38 – 1.27 (m, 4H).

^{13}C NMR (101 MHz, $CDCl_3$) δ 164.77, 162.68, 141.62, 136.43, 131.57, 129.86, 129.17, 127.62, 127.18, 126.74, 124.59, 119.28, 114.11, 55.58, 43.19, 35.75, 32.46, 27.44, 21.40.

HRMS (ESI+) *m*/*z* Calcd for $C_{21}H_{25}N_2O_3S^+$: 385.1580 [*M*+H]$^+$; found: 385.1592.

***N*-(4-(4-bromoisoquinolin-1-yl)pentyl)-4-methoxybenzenesulfonamide (25)**

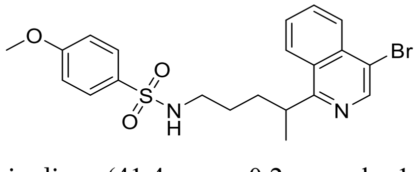

Prepared from 4-bromoisoquinoline (41.4 mg, 0.2 mmol, 1.0 equiv) and 4-methoxy-*N*-pentylbenzenesulfonamide **2a** (102.8 mg, 0.4 mmol, 2.0 equiv) following **general procedure E**. The product was obtained as yellow oil (41 mg, 44% yield) after silica gel column chromatography using petroleum ether/EtOAc (3:1 to 2:1).

^{1}H NMR (400 MHz, $CDCl_3$) δ 8.61 (s, 1H), 8.15 (dd, *J* = 12.8, 8.6 Hz, 2H), 7.80 – 7.70 (m, 3H), 7.64 (ddd, *J* = 8.4, 7.0, 1.3 Hz, 1H), 6.91 – 6.87 (m, 2H), 5.03 – 4.92 (m, 1H), 3.82 (s, 3H), 3.73

– 3.63 (m, 1H), 2.87 (q, *J* = 6.8 Hz, 2H), 2.05 – 1.94 (m, 1H), 1.67 (ddt, *J* = 13.3, 11.5, 5.7 Hz, 1H), 1.54 – 1.41 (m, 1H), 1.39 – 1.26 (m, 4H).
^{13}C NMR (101 MHz, $CDCl_3$) δ 164.56, 162.72, 143.42, 134.89, 131.45, 131.07, 129.14, 128.09, 128.01, 126.87, 124.96, 117.75, 114.15, 55.61, 43.15, 35.67, 32.53, 27.50, 21.19.
HRMS (ESI+) *m/z* Calcd for $C_{21}H_{24}BrN_2O_3S^+$: 463.0686 [*M*+H]$^+$; found: 463.0700.

4-methoxy-*N*-(4-(quinazolin-4-yl)pentyl)benzenesulfonamide (26)

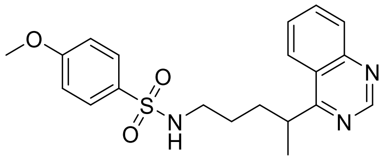

Prepared from quinazoline (26.0 mg, 0.2 mmol, 1.0 equiv) and 4-methoxy-*N*-pentylbenzenesulfonamide **2a** (102.8 mg, 0.4 mmol, 2.0 equiv) following **general procedure E**. The product was obtained as yellow oil (24 mg, 31% yield) after silica gel column chromatography using petroleum ether/EtOAc (3:1 to 1:1).
^{1}H NMR (400 MHz, $CDCl_3$) δ 9.20 (s, 1H), 8.11 (d, *J* = 8.4 Hz, 1H), 8.03 (d, *J* = 8.4 Hz, 1H), 7.87 (ddd, *J* = 8.3, 7.0, 1.2 Hz, 1H), 7.74 – 7.70 (m, 2H), 7.62 (ddd, *J* = 8.2, 7.0, 1.1 Hz, 1H), 6.91 – 6.87 (m, 2H), 4.95 (t, *J* = 6.0 Hz, 1H), 3.83 (s, 3H), 3.72 (h, *J* = 6.8 Hz, 1H), 2.89 (q, *J* = 6.7 Hz, 2H), 2.06 – 1.95 (m, 1H), 1.75 – 1.65 (m, 1H), 1.56 – 1.42 (m, 1H), 1.40 – 1.30 (m, 4H).
^{13}C NMR (101 MHz, $CDCl_3$) δ 174.66, 162.79, 154.53, 150.07, 133.59, 131.47, 129.33, 129.13, 127.68, 124.03, 123.64, 114.18, 55.60, 43.08, 35.58, 32.28, 27.54, 20.72.
HRMS (ESI+) *m/z* Calcd for $C_{20}H_{24}N_3O_3S^+$: 386.1533 [*M*+H]$^+$; found: 386.1541.

4-methoxy-*N*-(4-(phthalazin-1-yl)pentyl)benzenesulfonamide (27)

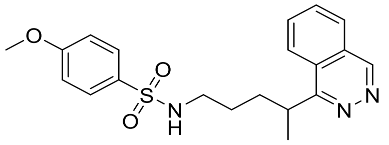

Prepared from phthalazine (26.0 mg, 0.2 mmol, 1.0 equiv) and 4-methoxy-*N*-pentylbenzenesulfonamide **2a** (102.8 mg, 0.4 mmol, 2.0 equiv) following **general procedure E**. The product was obtained as yellow oil (41 mg, 53% yield) after silica gel column chromatography using DCM/MeOH (40:1 to 20:1).
^{1}H NMR (400 MHz, $CDCl_3$) δ 9.38 (s, 1H), 8.14 (d, *J* = 8.0 Hz, 1H), 8.00 – 7.86 (m, 3H), 7.79 – 7.75 (m, 2H), 6.96 – 6.87 (m, 2H), 5.22 (t, *J* = 5.9 Hz, 1H), 3.85 (s, 3H), 3.78 – 3.65 (m, 1H), 2.93 (q, *J* = 6.7 Hz, 2H), 2.25 – 2.11 (m, 1H), 1.87 – 1.74 (m, 1H), 1.63 – 1.45 (m, 2H), 1.42 (d, *J* = 6.9 Hz, 3H).
^{13}C NMR (101 MHz, $CDCl_3$) δ 163.09, 162.67, 150.21, 132.65, 131.95, 131.61, 129.16, 127.27, 126.58, 125.34, 123.39, 114.13, 55.58, 43.18, 35.11, 32.45, 27.54, 20.85.
HRMS (ESI+) *m/z* Calcd for $C_{20}H_{24}N_3O_3S^+$: 386.1533 [*M*+H]$^+$; found: 386.1529.

4-methoxy-*N*-(4-(4-phenylpyridin-2-yl)pentyl)benzenesulfonamide (28)

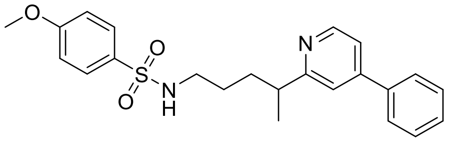

Prepared from 4-phenylpyridine (31.0 mg, 0.2 mmol, 1.0 equiv) and 4-methoxy-*N*-pentylbenzenesulfonamide **2a** (102.8 mg, 0.4 mmol, 2.0 equiv) following **general procedure E**. The product was obtained as colorless oil (47 mg, 57% yield) after silica gel column chromatography using petroleum ether/EtOAc (4:1 to 2:1).

^{1}H NMR (400 MHz, $CDCl_3$) δ 8.54 (d, *J* = 4.5 Hz, 1H), 7.75 (d, *J* = 8.4 Hz, 2H), 7.61 (d, *J* = 7.1 Hz, 2H), 7.50 – 7.40 (m, 3H), 7.32 (d, *J* = 4.5 Hz, 1H), 7.30 (s, 1H), 6.92 (d, *J* = 8.5 Hz, 2H), 5.07 (t, *J* = 6.1 Hz, 1H), 3.82 (s, 3H), 2.95 – 2.84 (m, 3H), 1.83 – 1.71 (m, 1H), 1.65 – 1.55 (m, 1H), 1.53 – 1.42 (m, 1H), 1.41 – 1.30 (m, 1H), 1.27 (d, *J* = 6.8 Hz, 3H).

^{13}C NMR (101 MHz, $CDCl_3$) δ 166.03, 162.71, 149.36, 149.16, 138.35, 131.65, 129.16, 129.10, 129.05, 127.06, 119.72, 119.51, 114.15, 55.57, 43.15, 41.29, 33.78, 27.45, 21.04.

HRMS (ESI+) *m/z* Calcd for $C_{23}H_{27}N_2O_3S^+$: 411.1737 $[M+H]^+$; found: 411.1733.

<u>*N*-(4-(4-cyanopyridin-2-yl)pentyl)-4-methoxybenzenesulfonamide (29)</u>

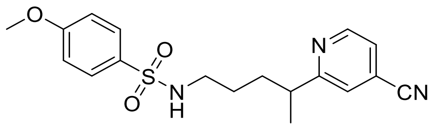

Prepared from isonicotinonitrile (20.8 mg, 0.2 mmol, 1.0 equiv) and 4-methoxy-*N*-pentylbenzenesulfonamide **2a** (102.8 mg, 0.4 mmol, 2.0 equiv) following **general procedure E**. The product was obtained as yellow oil (32 mg, 45% yield) after silica gel column chromatography using petroleum ether/EtOAc (4:1 to 2:1).

^{1}H NMR (400 MHz, $CDCl_3$) δ 8.69 (d, *J* = 4.9 Hz, 1H), 7.78 – 7.74 (m, 2H), 7.33 (dd, *J* = 5.0, 1.3 Hz, 1H), 7.30 (s, 1H), 6.98 – 6.93 (m, 2H), 4.68 (t, *J* = 6.1 Hz, 1H), 3.86 (s, 3H), 2.93 – 2.82 (m, 3H), 1.75 – 1.67 (m, 1H), 1.64 – 1.53 (m, 1H), 1.49 – 1.37 (m, 1H), 1.35 – 1.27 (m, 1H), 1.24 (d, *J* = 6.9 Hz, 3H).

^{13}C NMR (101 MHz, $CDCl_3$) δ 167.39, 162.84, 150.24, 131.46, 129.18, 123.41, 122.73, 120.73, 116.71, 114.22, 55.64, 43.02, 41.35, 33.41, 27.43, 20.59.

HRMS (ESI+) *m/z* Calcd for $C_{18}H_{22}N_3O_3S^+$: 360.1376 $[M+H]^+$; found: 360.1389.

<u>ethyl 2-(5-((4-methoxyphenyl)sulfonamido)pentan-2-yl)-4-methylthiazole-5-carboxylate (30)</u>

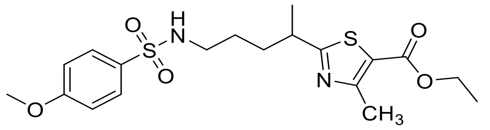

Prepared from ethyl 4-methylthiazole-5-carboxylate (34.2 mg, 0.2 mmol, 1.0 equiv) and 4-methoxy-*N*-pentylbenzenesulfonamide **2a** (102.8 mg, 0.4 mmol, 2.0 equiv) following **general procedure E**. The product was obtained as yellow oil (53 mg, 62% yield) after silica gel column chromatography using petroleum ether/EtOAc (4:1 to 2:1).

^{1}H NMR (400 MHz, $CDCl_3$) δ 7.76 (d, *J* = 8.2 Hz, 2H), 6.94 (d, *J* = 8.2 Hz, 2H), 5.01 (t, *J* = 6.1 Hz, 1H), 4.29 (q, *J* = 7.2 Hz, 2H), 3.84 (s, 3H), 3.04 (h, *J* = 7.2 Hz, 1H), 2.90 (q, *J* = 7.0 Hz, 2H), 2.65 (s, 3H), 1.76 – 1.57 (m, 2H), 1.54 – 1.39 (m, 2H), 1.34 (t, *J* = 7.2 Hz, 3H), 1.29 (d, *J* = 7.0 Hz, 3H).

^{13}C NMR (101 MHz, $CDCl_3$) δ 179.26, 162.80, 162.22, 159.55, 131.47, 129.15, 121.01, 114.21, 61.17, 55.61, 42.88, 38.22, 34.27, 27.03, 21.20, 17.30, 14.33.

HRMS (ESI+) *m/z* Calcd for $C_{19}H_{27}N_2O_5S_2^+$: 427.1356 [*M*+H]$^+$; found: 427.1359.

***N*-(4-(5-formylthiazol-2-yl)pentyl)-4-methoxybenzenesulfonamide (31)**

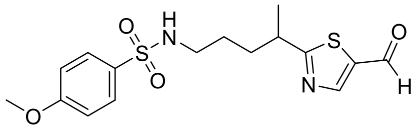

Prepared from thiazole-5-carbaldehyde (22.6 mg, 0.2 mmol, 1.0 equiv) and 4-methoxy-*N*-pentylbenzenesulfonamide **2a** (102.8 mg, 0.4 mmol, 2.0 equiv) following **general procedure E**. The product was obtained as yellow oil (37 mg, 50% yield) after silica gel column chromatography using petroleum ether/EtOAc (3:1 to 2:1).
^{1}H NMR (400 MHz, $CDCl_3$) δ 9.98 (s, 1H), 8.26 (s, 1H), 7.79 – 7.75 (m, 2H), 6.98 – 6.93 (m, 2H), 4.69 (br, 1H), 3.86 (s, 3H), 3.18 (h, *J* = 7.0 Hz, 1H), 2.93 (t, *J* = 7.0 Hz, 2H), 1.83 – 1.64 (m, 2H), 1.57 – 1.41 (m, 2H), 1.36 (d, *J* = 6.8 Hz, 3H).
^{13}C NMR (101 MHz, $CDCl_3$) δ 184.99, 182.07, 162.87, 151.14, 138.57, 131.45, 129.18, 114.26, 55.64, 42.86, 38.81, 34.16, 27.10, 21.10.
HRMS (ESI+) *m/z* Calcd for $C_{16}H_{21}N_2O_4S_2^+$: 369.0937 [*M*+H]$^+$; found: 369.0940.

4-methoxy-*N*-(4-(3,5,6-trimethylpyrazin-2-yl)pentyl)benzenesulfonamide (32)

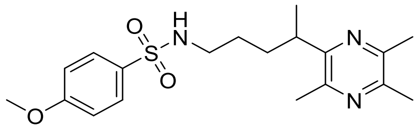

Prepared from 2,3,5-trimethylpyrazine (24.4 mg, 0.2 mmol, 1.0 equiv) and 4-methoxy-*N*-pentylbenzenesulfonamide **2a** (102.8 mg, 0.4 mmol, 2.0 equiv) following **general procedure E**. The product was obtained as colorless oil (35 mg, 46% yield) after silica gel column chromatography using petroleum ether/EtOAc (4:1 to 2:1).
^{1}H NMR (400 MHz, $CDCl_3$) δ 7.73 (d, *J* = 8.4 Hz, 2H), 6.92 (d, *J* = 8.3 Hz, 2H), 4.87 (t, *J* = 6.1 Hz, 1H), 3.83 (s, 3H), 2.94 – 2.86 (m, 1H), 2.83 (q, *J* = 6.7 Hz, 2H), 2.44 – 2.39 (m, 9H), 1.82 – 1.72 (m, 1H), 1.55 – 1.34 (m, 2H), 1.27 – 1.17 (m, 1H), 1.10 (d, *J* = 6.8 Hz, 3H).
^{13}C NMR (101 MHz, $CDCl_3$) δ 162.75, 154.57, 148.81, 147.89, 147.08, 131.55, 129.14, 114.15, 55.60, 43.17, 35.73, 32.22, 27.47, 21.57, 21.35, 20.76, 20.30.
HRMS (ESI+) *m/z* Calcd for $C_{19}H_{28}N_3O_3S^+$: 378.1846 [*M*+H]$^+$; found: 378.1852.

***N*-(4-(5-acetyl-6-methylpyrazin-2-yl)pentyl)-4-methoxybenzenesulfonamide (33)**

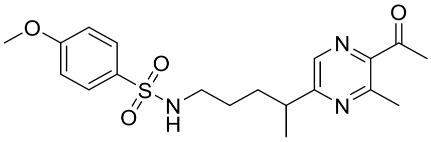

Prepared from 1-(3-methylpyrazin-2-yl)ethan-1-one (27.2 mg, 0.2 mmol, 1.0 equiv) and 4-methoxy-*N*-pentylbenzenesulfonamide **2a** (102.8 mg, 0.4 mmol, 2.0 equiv) following **general procedure E**. The product was obtained as yellow oil (32 mg, 41% yield) after silica gel column chromatography using petroleum ether/EtOAc (4:1 to 2:1).
^{1}H NMR (400 MHz, $CDCl_3$) δ 8.25 (s, 1H), 7.76 (d, *J* = 8.3 Hz, 2H), 6.94 (d, *J* = 8.3 Hz, 2H), 4.77 (br,

1H), 3.85 (s, 3H), 2.94 – 2.85 (m, 3H), 2.77 (s, 3H), 2.67 (s, 3H), 1.81 – 1.70 (m, 1H), 1.66 – 1.56 (m, 1H), 1.52 – 1.40 (m, 1H), 1.38 – 1.28 (m, 1H), 1.25 (d, *J* = 6.8 Hz, 3H).
^{13}C NMR (101 MHz, $CDCl_3$) δ 201.24, 162.83, 162.47, 153.47, 144.65, 139.43, 131.44, 129.15, 114.22, 55.63, 43.00, 39.00, 33.12, 27.88, 27.45, 23.65, 20.28.
HRMS (ESI+) *m*/*z* Calcd for $C_{19}H_{26}N_3O_4S^+$: 392.1639 [*M*+H]$^+$; found: 392.1628.

***N*-(4-(4,6-dimethylpyrimidin-2-yl)pentyl)-4-methoxybenzenesulfonamide (34)**

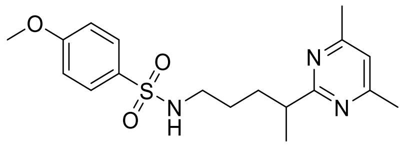

Prepared from 4,6-dimethylpyrimidine (21.6 mg, 0.2 mmol, 1.0 equiv) and 4-methoxy-*N*-pentylbenzenesulfonamide **2a** (102.8 mg, 0.4 mmol, 2.0 equiv) following **general procedure E**. The product was obtained as yellow oil (57 mg, 78% yield) after silica gel column chromatography using petroleum ether/EtOAc (3:1 to 2:1).
^{1}H NMR (400 MHz, $CDCl_3$) δ 7.76 (d, *J* = 8.2 Hz, 2H), 6.94 (d, *J* = 8.3 Hz, 2H), 6.83 (s, 1H), 5.21 (t, *J* = 5.9 Hz, 1H), 3.85 (s, 3H), 2.94 – 2.83 (m, 3H), 2.43 (s, 6H), 1.84 – 1.73 (m, 1H), 1.57 – 1.40 (m, 2H), 1.37 – 1.27 (m, 1H), 1.21 (d, *J* = 6.7 Hz, 3H).
^{13}C NMR (101 MHz, $CDCl_3$) δ 173.28, 166.61, 162.70, 131.71, 129.17, 117.64, 114.14, 55.59, 43.09, 42.17, 32.86, 27.09, 23.95, 20.34.
HRMS (ESI+) *m*/*z* Calcd for $C_{18}H_{26}N_3O_3S^+$: 364.1689 [*M*+H]$^+$; found: 364.1670.

4-methoxy-*N*-(4-(pyridazin-3-yl)pentyl)benzenesulfonamide (35)

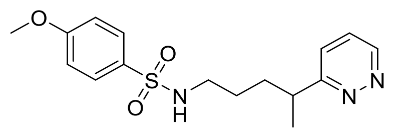

Prepared from pyridazine (16.0 mg, 0.2 mmol, 1.0 equiv) and 4-methoxy-*N*-pentylbenzenesulfonamide **2a** (102.8 mg, 0.4 mmol, 2.0 equiv) following **general procedure E**. The product was obtained as yellow oil (42 mg, 63% yield) after silica gel column chromatography using DCM/MeOH (35:1 to 25:1).
^{1}H NMR (400 MHz, $CDCl_3$) δ 9.04 (d, *J* = 5.3 Hz, 1H), 8.99 (s, 1H), 7.80 – 7.74 (m, 2H), 7.25 (dd, *J* = 5.3, 2.3 Hz, 1H), 6.98 – 6.93 (m, 2H), 5.35 (t, *J* = 6.1 Hz, 1H), 3.87 (s, 3H), 2.92 (q, *J* = 6.6 Hz, 2H), 2.69 (h, *J* = 7.0 Hz, 1H), 1.63 (q, *J* = 7.5 Hz, 2H), 1.52 – 1.41 (m, 1H), 1.39 – 1.30 (m, 1H), 1.25 (d, *J* = 7.0 Hz, 3H).
^{13}C NMR (101 MHz, $CDCl_3$) δ 162.83, 151.85, 151.13, 146.12, 131.49, 129.11, 124.62, 114.24, 55.65, 42.84, 36.76, 33.88, 27.35, 20.82.
HRMS (ESI+) *m*/*z* Calcd for $C_{16}H_{22}N_3O_3S^+$: 336.1376 [*M*+H]$^+$; found: 336.1384.

***N*-(4-(acridin-9-yl)pentyl)-4-methoxybenzenesulfonamide (36)**

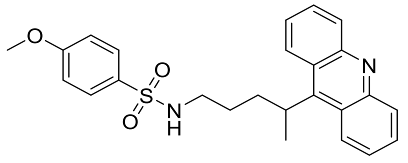

Prepared from acridine (35.8 mg, 0.2 mmol, 1.0 equiv) and 4-methoxy-*N*-pentylbenzenesulfonamide **2a** (102.8 mg, 0.4 mmol, 2.0 equiv) following **general procedure E**. The product was obtained as yellow oil (66 mg, 76% yield) after silica gel column chromatography using petroleum ether/EtOAc (2:1 to 1:1).

^{1}H NMR (400 MHz, $CDCl_3$) δ 8.33 (d, *J* = 28.8 Hz, 2H), 8.21 (d, *J* = 8.7 Hz, 2H), 7.72 (t, *J* = 7.7 Hz, 2H), 7.67 – 7.63 (m, 2H), 7.54 – 7.43 (m, 2H), 6.87 – 6.80 (m, 2H), 4.65 (t, *J* = 6.3 Hz, 1H), 4.25 (h, *J* = 7.2 Hz, 1H), 3.81 (s, 3H), 2.83 (q, *J* = 6.7 Hz, 2H), 2.26 – 2.09 (m, 2H), 1.67 (d, *J* = 7.3 Hz, 3H), 1.54 – 1.42 (m, 1H), 1.22 – 1.10 (m, 1H).

^{13}C NMR (101 MHz, $CDCl_3$) δ 162.75, 150.19, 148.88, 148.53, 131.36, 130.88, 130.45, 129.67, 129.47, 129.04, 125.98, 125.82, 125.70, 125.37, 124.73, 123.51, 114.14, 55.60, 43.00, 34.07, 33.71, 28.76, 21.27.

HRMS (ESI+) *m*/*z* Calcd for $C_{25}H_{27}N_2O_3S^+$: 435.1737 [*M*+H]$^+$; found: 435.1743.

<u>*N*-(4-(3-chloroquinoxalin-2-yl)pentyl)-4-methoxybenzenesulfonamide (37)</u>

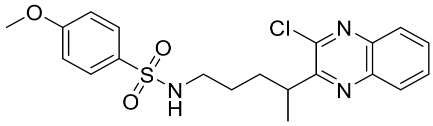

Prepared from 2-chloroquinoxaline (32.8 mg, 0.2 mmol, 1.0 equiv) and 4-methoxy-*N*-pentylbenzenesulfonamide **2a** (102.8 mg, 0.4 mmol, 2.0 equiv) following the **Procedures for preparation of specific compounds**. The product was obtained as colorless oil (63 mg, 75% yield) after silica gel column chromatography using petroleum ether/EtOAc (3:1 to 1:1).

^{1}H NMR (400 MHz, $CDCl_3$) δ 8.03 (dd, *J* = 7.0, 2.9 Hz, 1H), 7.95 (dt, *J* = 9.2, 2.0 Hz, 1H), 7.77 – 7.67 (m, 4H), 6.88 (d, *J* = 7.9 Hz, 2H), 4.95 (br, 1H), 3.80 (s, 3H), 3.47 (h, *J* = 7.2 Hz, 1H), 2.96 – 2.88 (m, 2H), 2.00 – 1.91 (m, 1H), 1.68 – 1.57 (m, 1H), 1.56 – 1.46 (m, 1H), 1.43 – 1.32 (m, 1H), 1.28 (d, *J* = 6.5 Hz, 3H).

^{13}C NMR (101 MHz, $CDCl_3$) δ 162.75, 158.54, 147.44, 140.96, 140.65, 131.45, 130.18, 130.10, 129.15, 128.75, 128.03, 114.17, 55.59, 43.13, 37.25, 31.90, 27.36, 19.67.

HRMS (ESI+) *m*/*z* Calcd for $C_{20}H_{23}ClN_3O_3S^+$: 420.1143 [*M*+H]$^+$; found: 420.1152.

<u>4-methoxy-*N*-(4-(4-oxochroman-2-yl)pentyl)benzenesulfonamide (38)</u>

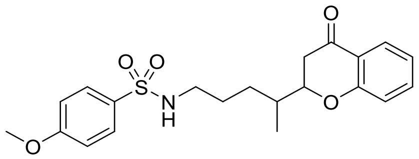

Prepared from 4*H*-chromen-4-one (29.2 mg, 0.2 mmol, 1.0 equiv) and 4-methoxy-*N*-pentylbenzenesulfonamide **2a** (102.8 mg, 0.4 mmol, 2.0 equiv) following **general procedure E**. The product was obtained as colorless oil (38 mg, 47% yield) after silica gel column chromatography using petroleum ether/EtOAc (4:1).

^{1}H NMR (600 MHz, $CDCl_3$) δ 7.84 (dd, *J* = 7.8, 1.7 Hz, 1H), 7.79 (dd, *J* = 8.8, 1.7 Hz, 2H), 7.49 – 7.41 (m, 1H), 6.98 (t, *J* = 7.5 Hz, 1H), 6.96 – 6.92 (m, 3H), 4.87 (t, *J* = 6.1 Hz, 1H), 4.23 (ddd, *J* = 13.8, 4.1, 2.5 Hz, 0.5H), 4.16 (ddd, *J* = 13.0, 6.1, 2.9 Hz, 0.5H), 3.83 (s, 3H), 2.95 (hept, *J* = 6.3 Hz, 2H), 2.74 – 2.46 (m, 2H), 1.89 – 1.73 (m, 1H), 1.63 – 1.43 (m, 3H), 1.24 (qt, *J* = 13.0, 7.6 Hz, 1H), 0.98 (d, *J* = 6.9 Hz, 1.5H), 0.95 (d, *J* = 6.8 Hz, 1.5H).

^{13}C NMR (151 MHz, $CDCl_3$) δ 192.96, 192.82, 162.85, 161.82, 161.72, 136.02, 135.97, 131.58, 129.19, 126.91, 126.89, 121.22, 121.18, 120.91, 120.88, 117.91, 117.89, 114.25, 81.35, 80.93, 55.60, 43.28, 39.93, 39.71, 36.60, 36.57, 29.11, 28.95, 27.23, 27.00, 14.75, 14.36.
HRMS (ESI+) *m/z* Calcd for $C_{21}H_{26}NO_5S^+$: 404.1526 [*M*+H]$^+$; found: 404.1530.

***N*-(4-(6-chlorobenzo[*d*]thiazol-2-yl)hexyl)-4-methoxybenzenesulfonamide (39)**

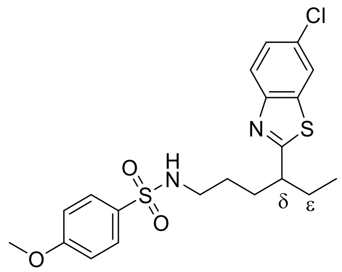

Prepared from 6-chlorobenzo[*d*]thiazole (33.8 mg, 0.2 mmol, 1.0 equiv) and *N*-hexyl-4-methoxybenzenesulfonamide **2b** (108.4 mg, 0.4 mmol, 2.0 equiv) following **general procedure E**. The inseparable mixture of *δ*- and *ε*-arylated products were obtained as yellow oil (*δ:ε* = 3.3:1, 42 mg, 48% yield) after silica gel column chromatography using petroleum ether/EtOAc (4:1 to 3:1).
^{1}H NMR (400 MHz, $CDCl_3$) δ 7.87 (d, *J* = 8.7 Hz, 0.3H), 7.84 (d, *J* = 8.7 Hz, 1H), 7.80 – 7.76 (m, 1H+0.6H), 7.75 – 7.70 (m, 2H+0.3H), 7.38 (dd, *J* = 8.7, 2.1 Hz, 1H+0.3H), 6.95 – 6.86 (m, 2H+0.6H), 5.09 (br, 1H), 4.98 (br, 0.3H), 3.83 (s, 0.9H), 3.82 (s, 3H), 3.19 (h, *J* = 7.0 Hz, 0.3H), 3.01 – 2.93 (m, 1H), 2.89 (t, *J* = 6.8 Hz, 2H+0.6H), 1.82 – 1.68 (m, 4H+0.9H), 1.54 – 1.27 (m, 2H+1.8H), 0.85 (t, *J* = 7.4 Hz, 3H).
^{13}C NMR (101 MHz, $CDCl_3$) δ 178.19, 176.81, 162.76, 151.32, 151.22, 135.77, 135.72, 131.49, 131.45, 130.72, 130.65, 129.17, 129.14, 126.74, 126.72, 123.32, 121.23, 121.21, 114.20, 114.18, 55.60, 46.27, 42.90, 42.80, 39.16, 36.51, 32.34, 29.33, 29.15, 27.16, 24.07, 21.17, 11.74.
HRMS (ESI+) *m/z* Calcd for $C_{20}H_{24}ClN_2O_3S_2^+$: 439.0911 [*M*+H]$^+$; found: 439.0916.

***N*-(4-(6-chlorobenzo[*d*]thiazol-2-yl)heptyl)-4-methoxybenzenesulfonamide (40)**

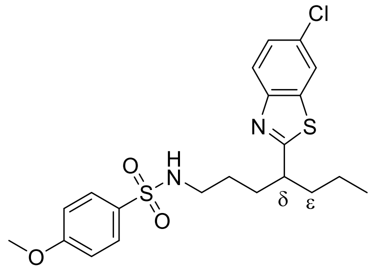

Prepared from 6-chlorobenzo[*d*]thiazole (33.8 mg, 0.2 mmol, 1.0 equiv) and *N*-heptyl-4-methoxybenzenesulfonamide **2c** (114.1 mg, 0.4 mmol, 2.0 equiv) following **general procedure E**. The inseparable mixture of *δ*- and *ε*-arylated products were obtained as yellow oil (*δ:ε* = 6.2:1, 52 mg, 57% yield) after silica gel column chromatography using petroleum ether/EtOAc (4:1 to 3:1).
^{1}H NMR (600 MHz, $CDCl_3$) δ 7.86 (d, *J* = 8.7 Hz, 0.16H), 7.82 (d, *J* = 8.6 Hz, 1H), 7.79 (s, 0.16H), 7.77 (s, 1H), 7.75 – 7.70 (m, 2H+0.32H), 7.38 (d, *J* = 8.7 Hz, 1H+0.16H), 6.94 – 6.91 (m, 0.32H), 6.90 – 6.87 (m, 2H), 5.08 (br, 1H), 4.94 (br, 0.16H), 3.83 (s, 0.48H), 3.81 (s, 3H), 3.08 – 3.02 (m, 1H), 2.99 – 2.94 (m, 0.16H), 2.93 – 2.84 (m, 2H+0.32H), 1.80 – 1.61 (m, 4H+0.64H), 1.53 – 1.33 (m, 2H+0.80H), 1.31 – 1.17 (m, 2H+0.32H), 0.85 (t, *J* = 7.5 Hz, 3H).

^{13}C NMR (151 MHz, $CDCl_3$) δ 177.07, 176.90, 162.77, 151.47, 151.36, 135.84, 135.81, 131.62, 131.53, 130.67, 130.62, 129.16, 129.13, 126.66, 126.63, 123.39, 123.36, 121.20, 114.21, 114.17, 55.58, 46.66, 44.48, 42.90, 42.80, 38.45, 34.85, 32.74, 29.27, 29.21, 27.17, 24.16, 20.40, 13.92, 11.79.

HRMS (ESI+) *m/z* Calcd for $C_{21}H_{26}ClN_2O_3S_2^+$: 453.1068 [*M*+H]$^+$; found: 453.1082.

***N*-(4-(6-chlorobenzo[*d*]thiazol-2-yl)octyl)-4-methoxybenzenesulfonamide (41)**

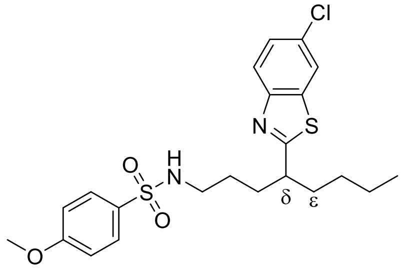

Prepared from 6-chlorobenzo[*d*]thiazole (33.8 mg, 0.2 mmol, 1.0 equiv) and 4-methoxy-*N*-octylbenzenesulfonamide **2d** (119.7 mg, 0.4 mmol, 2.0 equiv) following **general procedure E**. The inseparable mixture of *δ*- and *ε*-arylated products were obtained as yellow oil (*δ:ε* = 8.3:1, 56 mg, 60% yield) after silica gel column chromatography using petroleum ether/EtOAc (4:1 to 3:1).

^{1}H NMR (400 MHz, $CDCl_3$) δ 7.86 (d, *J* = 8.7 Hz, 0.12H), 7.83 (d, *J* = 8.7 Hz, 1H), 7.80 – 7.76 (m, 1H+0.12H), 7.75 – 7.71 (m, 2H+0.24H), 7.41 – 7.33 (m, 1H+0.12H), 6.93 – 6.85 (m, 2H+0.24H), 5.09 (br, 1H), 4.94 (t, *J* = 6.1 Hz, 0.12H), 3.83 (s, 0.36H), 3.81 (s, 3H), 3.09 – 2.98 (m, 1H+0.12H), 2.93 – 2.82 (m, 2H+0.24H), 1.80 – 1.63 (m, 4H+0.48H), 1.53 – 1.32 (m, 2H+0.24H), 1.31 – 1.12 (m, 4H+0.48H), 0.89 – 0.78 (m, 3H+0.36H).

^{13}C NMR (151 MHz, $CDCl_3$) δ 176.98, 162.76, 151.31, 135.80, 131.54, 130.68, 129.13, 126.67, 123.35, 121.22, 114.17, 55.58, 44.71, 42.91, 36.05, 32.78, 29.34, 27.18, 22.54, 13.86.

HRMS (ESI+) *m/z* Calcd for $C_{22}H_{28}ClN_2O_3S_2^+$: 467.1224 [*M*+H]$^+$; found: 467.1243.

***N*-(2,2-dimethyl-4-(2-phenylquinolin-4-yl)hexyl)-4-methoxybenzenesulfonamide (42)**

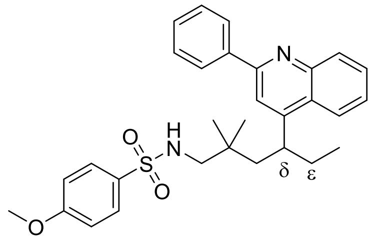

Prepared from 2-phenylquinoline (41.0 mg, 0.2 mmol, 1.0 equiv) and *N*-(2,2-dimethylhexyl)-4-methoxybenzenesulfonamide **2e** (119.7 mg, 0.4 mmol, 2.0 equiv) following **general procedure E**. The inseparable mixture of *δ*- and *ε*-arylated products were obtained as yellow oil (*δ:ε* = 2.5:1, 89 mg, 89% yield) after silica gel column chromatography using petroleum ether/EtOAc (4:1 to 3:1).

^{1}H NMR (600 MHz, $CDCl_3$) δ 8.24 – 8.21 (m, 1H), 8.18 – 8.04 (m, 3H), 7.80 – 7.57 (m, 4H), 7.56 – 7.50 (m, 3H), 7.48 – 7.43 (m, 1H), 6.91 – 6.82 (m, 2H), 5.16 (t, *J* = 6.9 Hz, 0.28H), 4.99 (t, *J* = 7.2 Hz, 0.72H), 3.82 – 3.77 (m, 3H), 3.60 – 3.47 (m, 1H), 2.68 – 2.37 (m, 2H), 2.03 – 1.57 (m, 3.6H), 1.41 – 1.16 (m, 1.4H), 0.87 – 0.66 (m, 8H).

^{13}C NMR (101 MHz, $CDCl_3$) δ 162.74, 162.65, 157.21, 156.82, 154.45, 154.35, 148.57, 140.05, 139.88, 131.50, 131.32, 130.76, 130.60, 129.36, 129.32, 129.24, 129.15, 128.91, 128.86, 127.66,

127.62, 126.74, 126.35, 126.19, 126.16, 122.94, 122.69, 116.24, 115.48, 114.24, 55.58, 53.50, 52.75, 45.63, 37.37, 35.65, 34.91, 34.31, 33.76, 33.01, 31.34, 25.79, 25.64, 25.07, 24.90, 21.37, 12.01.
HRMS (ESI+) *m*/*z* Calcd for $C_{30}H_{35}N_2O_3S^+$: 503.2363 [*M*+H]$^+$; found: 503.2378.

<u>*N*-(4-(6-chlorobenzo[*d*]thiazol-2-yl)-2-ethylhexyl)-4-methoxybenzenesulfonamide (43)</u>

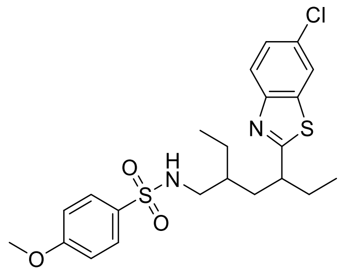

Prepared from 6-chlorobenzo[*d*]thiazole (33.8 mg, 0.2 mmol, 1.0 equiv) and *N*-(2-ethylhexyl)-4-methoxybenzenesulfonamide **2i** (119.7 mg, 0.4 mmol, 2.0 equiv) following **general procedure E**. The product was obtained as yellow oil (61 mg, 65% yield) after silica gel column chromatography using petroleum ether/EtOAc (4:1 to 3:1).
^{1}H NMR (400 MHz, $CDCl_3$) δ 8.03 (d, *J* = 8.8 Hz, 0.55H), 7.89 (d, *J* = 8.8 Hz, 0.45H), 7.82 (d, *J* = 2.2 Hz, 1H), 7.75 (dd, *J* = 19.1, 8.4 Hz, 2H), 7.44 (dd, *J* = 10.8, 8.4 Hz, 1H), 6.90 (dd, *J* = 19.3, 8.4 Hz, 2H), 5.92 (br, 0.55H), 5.33 (br, 0.45H), 3.84 (d, *J* = 6.2 Hz, 3H), 3.08 (dt, *J* = 11.4, 5.9 Hz, 1H), 2.99 – 2.69 (m, 2H), 1.76 (dt, *J* = 14.6, 7.2 Hz, 3H), 1.58 – 1.17 (m, 4H), 0.91 – 0.85 (m, 3H), 0.77 (dt, *J* = 26.0, 7.2 Hz, 3H).
^{13}C NMR (101 MHz, $CDCl_3$) δ 177.25, 177.06, 162.72, 162.60, 151.17, 151.00, 135.83, 135.81, 131.59, 131.46, 130.92, 130.76, 129.13, 129.11, 126.93, 126.74, 123.61, 123.40, 121.27, 121.17, 114.16, 114.05, 55.60, 55.57, 46.27, 45.36, 44.46, 44.05, 37.77, 37.65, 37.00, 36.24, 30.57, 29.98, 25.02, 24.87, 11.84, 11.21, 10.84, 10.81.
HRMS (ESI+) *m*/*z* Calcd for $C_{22}H_{28}ClN_2O_3S_2^+$: 467.1224 [*M*+H]$^+$; found: 467.1233.

<u>4-methoxy-*N*-(5-methyl-4-(2-phenylquinolin-4-yl)hexyl)benzenesulfonamide (44)</u>

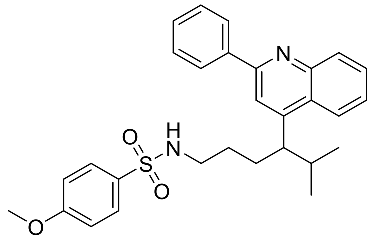

Prepared from 2-phenylquinoline (41.0 mg, 0.2 mmol, 1.0 equiv) and 4-methoxy-*N*-(5-methylhexyl)benzenesulfonamide **2k** (114.1 mg, 0.4 mmol, 2.0 equiv) following **general procedure E**. The product was obtained as yellow solid (65 mg, 67% yield) after silica gel column chromatography using petroleum ether/EtOAc (4:1 to 2:1).
^{1}H NMR (600 MHz, $CDCl_3$) δ 8.25 – 8.22 (m, 1H), 8.17 – 8.14 (m, 2H), 8.05 (d, *J* = 8.5 Hz, 1H), 7.74 – 7.66 (m, 4H), 7.55 – 7.51 (m, 3H), 7.49 – 7.45 (m, 1H), 6.87 – 6.83 (m, 2H), 4.87 (t, *J* = 6.1 Hz, 1H), 3.81 (s, 3H), 3.35 – 3.22 (m, 1H), 2.87 – 2.77 (m, 2H), 2.06 – 1.94 (m, 2H), 1.85 – 1.73 (m, 1H), 1.28 – 1.15 (m, 2H), 1.01 (d, *J* = 6.6 Hz, 3H), 0.80 (d, *J* = 6.6 Hz, 3H).
^{13}C NMR (151 MHz, $CDCl_3$) δ 162.72, 156.79, 151.79, 148.61, 139.92, 131.48, 130.65, 129.28, 129.24, 129.04, 128.84, 127.63, 126.87, 126.10, 123.05, 116.18, 114.16, 55.57, 44.76, 43.16, 33.76,

28.98, 27.42, 21.25, 20.25.

HRMS (ESI+) *m*/*z* Calcd for $C_{29}H_{33}N_2O_3S^+$: 489.2206 [*M*+H]$^+$; found: 489.2193.

<u>*N*-(4-(6-chlorobenzo[*d*]thiazol-2-yl)-1-phenylpentyl)-4-methoxybenzenesulfonamide (45)</u>

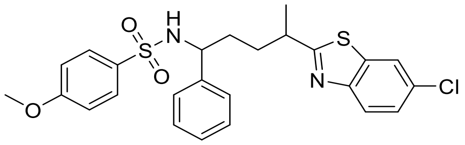

Prepared from 6-chlorobenzo[*d*]thiazole (33.8 mg, 0.2 mmol, 1.0 equiv) and 4-methoxy-*N*-(1-phenylpentyl)benzenesulfonamide **2f** (133.3 mg, 0.4 mmol, 2.0 equiv) following **general procedure E**. The product was obtained as yellow oil (49 mg, 49% yield) after silica gel column chromatography using petroleum ether/EtOAc (4:1 to 3:1).

^{1}H NMR (400 MHz, $CDCl_3$) δ 7.91 (t, *J* = 7.3 Hz, 1H), 7.78 (s, 1H), 7.56 (t, *J* = 8.1 Hz, 2H), 7.41 (dd, *J* = 8.7, 2.2 Hz, 1H), 7.15 – 7.08 (m, 3H), 7.04 – 6.97 (m, 2H), 6.77 – 6.67 (m, 2H), 5.85 (d, *J* = 6.4 Hz, 0.5H), 5.79 (d, *J* = 6.6 Hz, 0.5H), 4.24 (t, *J* = 7.1 Hz, 1H), 3.76 (s, 3H), 3.17 (h, *J* = 7.4 Hz, 1H), 1.87 – 1.58 (m, 4H), 1.33 (d, *J* = 6.3 Hz, 3H).

^{13}C NMR (101 MHz, $CDCl_3$) δ 177.61, 177.57, 162.50, 162.49, 151.32, 151.29, 140.93, 140.77, 135.83, 132.24, 132.22, 130.74, 130.70, 129.13, 129.10, 128.46, 127.39, 127.36, 126.81, 126.77, 126.44, 126.39, 123.46, 121.21, 121.19, 113.81, 58.25, 58.12, 55.55, 55.54, 38.86, 38.82, 35.13, 34.73, 32.98, 32.92, 21.57, 21.08.

HRMS (ESI+) *m*/*z* Calcd for $C_{25}H_{26}ClN_2O_3S_2^+$: 501.1068 [*M*+H]$^+$; found: 501.1073.

<u>methyl 2-((4-methoxyphenyl)sulfonamido)-5-(2-phenylquinolin-4-yl)hexanoate (46)</u>

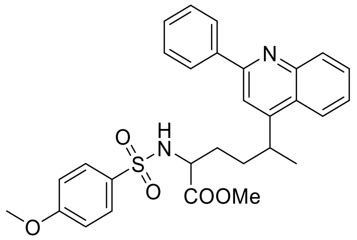

Prepared from 2-phenylquinoline (41.0 mg, 0.2 mmol, 1.0 equiv) and methyl 2-((4-methoxyphenyl)sulfonamido)hexanoate **2j** (126.0 mg, 0.4 mmol, 2.0 equiv) following **general procedure E**. The product was obtained as colorless oil (84 mg, 81% yield) after silica gel column chromatography using petroleum ether/EtOAc (3:1 to 2:1).

^{1}H NMR (600 MHz, $CDCl_3$) δ 8.21 (d, *J* = 8.4 Hz, 1H), 8.16 (d, *J* = 7.8 Hz, 2H), 8.04 (t, *J* = 7.8 Hz, 1H), 7.71 (dd, *J* = 10.0, 6.8 Hz, 4H), 7.52 (t, *J* = 7.5 Hz, 3H), 7.45 (t, *J* = 7.3 Hz, 1H), 6.85 (dd, *J* = 13.3, 8.8 Hz, 2H), 5.47 (d, *J* = 9.3 Hz, 0.45H), 5.44 (d, *J* = 9.0 Hz, 0.55H), 3.89 (dtd, *J* = 12.6, 8.4, 5.1 Hz, 1H), 3.76 (s, 1.65H), 3.74 (s, 1.35H), 3.58 (h, *J* = 6.6 Hz, 1H), 3.40 (s, 1.35H), 3.38 (s, 1.65H), 1.96 – 1.69 (m, 3H), 1.68 – 1.52 (m, 1H), 1.38 (dd, *J* = 6.5, 4.0 Hz, 3H).

^{13}C NMR (151 MHz, $CDCl_3$) δ 171.99, 171.95, 163.01, 162.98, 157.18, 157.15, 153.12, 153.05, 148.63, 148.59, 139.93, 139.88, 131.06, 130.72, 130.67, 129.38, 129.36, 129.32, 129.30, 128.86, 128.85, 127.65, 127.60, 126.24, 126.21, 126.09, 125.99, 122.79, 122.71, 115.51, 115.45, 114.14, 114.11, 55.67, 55.62, 55.60, 52.54, 33.17, 33.13, 32.32, 32.15, 31.16, 21.66, 21.21.

HRMS (ESI+) *m*/*z* Calcd for $C_{29}H_{31}N_2O_5S^+$: 519.1948 [*M*+H]$^+$; found: 519.1953.

4-methoxy-*N*-(2-methyl-4-(2-phenylquinolin-4-yl)pentyl)benzenesulfonamide (47)

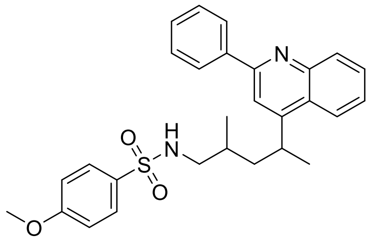

Prepared from 2-phenylquinoline (41.0 mg, 0.2 mmol, 1.0 equiv) and 4-methoxy-*N*-(2-methylpentyl)benzenesulfonamide **2g** (108.4 mg, 0.4 mmol, 2.0 equiv) following **general procedure E**. The product was obtained as white solid (73 mg, 77% yield) after silica gel column chromatography using petroleum ether/EtOAc (4:1 to 3:1).

^{1}H NMR (600 MHz, $CDCl_3$) δ 8.24 – 8.21 (m, 1H), 8.19 – 7.99 (m, 3H), 7.75 – 7.66 (m, 4H), 7.56 – 7.49 (m, 3H), 7.45 (td, *J* = 7.1, 1.5 Hz, 1H), 6.86 – 6.82 (m, 2H), 5.25 (t, *J* = 6.5 Hz, 0.67H), 5.14 (t, *J* = 6.5 Hz, 0.33H), 3.77 – 3.75 (m, 3H), 3.73 – 3.63 (m, 1H), 2.87 – 2.71 (m, 2H), 1.77 – 1.47 (m, 3H), 1.36 – 1.30 (m, 3H), 0.95 (d, *J* = 6.5 Hz, 2H), 0.83 (d, *J* = 6.4 Hz, 1H).

^{13}C NMR (151 MHz, $CDCl_3$) δ 162.74, 162.73, 154.13, 153.70, 148.59, 148.56, 139.95, 139.86, 131.57, 131.53, 130.67, 130.63, 129.30, 129.26, 129.07, 129.06, 128.87, 128.82, 127.69, 127.65, 126.24, 126.22, 126.15, 125.84, 122.74, 122.59, 115.73, 115.66, 114.19, 55.56, 49.14, 49.10, 41.51, 41.22, 31.11, 31.08, 30.79, 22.76, 20.92, 17.95, 17.54.

HRMS (ESI+) *m*/*z* Calcd for $C_{28}H_{31}N_2O_3S^+$: 475.2050 [*M*+H]$^+$; found: 475.2048.

4-methoxy-*N*-(3-methyl-4-(2-phenylquinolin-4-yl)pentyl)benzenesulfonamide (48)

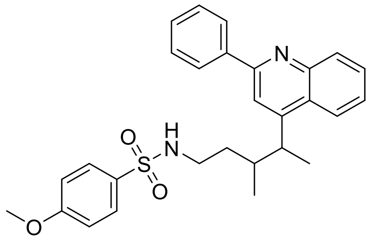

Prepared from 2-phenylquinoline (41.0 mg, 0.2 mmol, 1.0 equiv) and 4-methoxy-*N*-(3-methylpentyl)benzenesulfonamide **2h** (108.4 mg, 0.4 mmol, 2.0 equiv) following **general procedure E**. The product was obtained as colorless oil (69 mg, 73% yield) after silica gel column chromatography using petroleum ether/EtOAc (4:1 to 3:1)

^{1}H NMR (600 MHz, $CDCl_3$) δ 8.21 (ddd, *J* = 8.5, 2.9, 1.3 Hz, 1H), 8.13 (ddt, *J* = 9.4, 8.2, 1.3 Hz, 2H), 8.05 (dd, *J* = 8.6, 1.3 Hz, 0.6H), 8.00 (dd, *J* = 8.6, 1.3 Hz, 0.4H), 7.73 – 7.66 (m, 4H), 7.55 – 7.50 (m, 3H), 7.47 – 7.43 (m, 1H), 6.89 – 6.86 (m, 0.8H), 6.85 – 6.82 (m, 1.2H), 4.75 (t, *J* = 6.2 Hz, 0.4H), 4.70 (t, *J* = 6.2 Hz, 0.6H), 3.79 (d, *J* = 3.3 Hz, 3H), 3.54 – 3.43 (m, 1H), 3.03 – 2.95 (m, 1H), 2.93 – 2.75 (m, 1H), 2.05 – 1.92 (m, 1H), 1.73 – 1.49 (m, 1H), 1.38 – 1.28 (m, 4H), 0.86 – 0.83 (m, 3H).

^{13}C NMR (151 MHz, $CDCl_3$) δ 162.80, 162.77, 156.93, 156.90, 153.07, 152.98, 148.62, 148.58, 139.92, 131.36, 131.30, 130.69, 130.63, 129.28, 129.27, 129.23, 129.13, 129.09, 128.84, 128.83, 127.65, 126.33, 126.26, 126.19, 126.12, 122.98, 122.94, 116.60, 116.56, 114.21, 114.18, 55.57, 41.37, 41.35, 39.15, 38.23, 35.87, 35.21, 35.13, 32.63, 17.90, 16.97, 15.99, 15.34.

HRMS (ESI+) *m*/*z* Calcd for $C_{28}H_{31}N_2O_3S^+$: 475.2050 [*M*+H]$^+$; found: 475.2056.

<u>*N*-(2-(1-(6-chlorobenzo[*d*]thiazol-2-yl)ethoxy)ethyl)-4-methoxybenzenesulfonamide (49)</u>

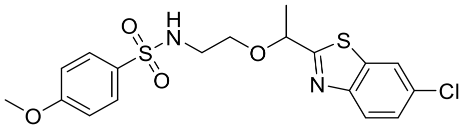

Prepared from 6-chlorobenzo[*d*]thiazole (33.8 mg, 0.2 mmol, 1.0 equiv) and *N*-(2-ethoxyethyl)-4-methoxybenzenesulfonamide **2l** (103.6 mg, 0.4 mmol, 2.0 equiv) following **general procedure E**. The product was obtained as yellow oil (53 mg, 62% yield) after silica gel column chromatography using petroleum ether/EtOAc (3:1 to 2:1).

^{1}H NMR (400 MHz, $CDCl_3$) δ 7.89 (d, *J* = 8.7 Hz, 1H), 7.82 (d, *J* = 2.0 Hz, 1H), 7.79 – 7.75 (m, 2H), 7.41 (dd, *J* = 8.7, 2.1 Hz, 1H), 6.93 – 6.88 (m, 2H), 5.43 (br, 1H), 4.75 (q, *J* = 6.6 Hz, 1H), 3.82 (s, 3H), 3.62 (ddd, *J* = 9.8, 5.9, 4.0 Hz, 1H), 3.53 (ddd, *J* = 9.7, 6.2, 4.2 Hz, 1H), 3.22 – 3.10 (m, 2H), 1.56 (d, *J* = 6.6 Hz, 3H).

^{13}C NMR (101 MHz, $CDCl_3$) δ 175.28, 162.86, 151.36, 135.94, 131.49, 131.23, 129.18, 127.01, 123.86, 121.50, 114.23, 76.37, 68.13, 55.62, 43.05, 22.19.

HRMS (ESI+) *m*/*z* Calcd for $C_{18}H_{20}ClN_2O_4S_2^+$: 427.0548 [*M*+H]$^+$; found: 427.0552.

<u>*N*-(6-chloro-4-(6-chlorobenzo[*d*]thiazol-2-yl)hexyl)-4-methoxybenzenesulfonamide (50)</u>

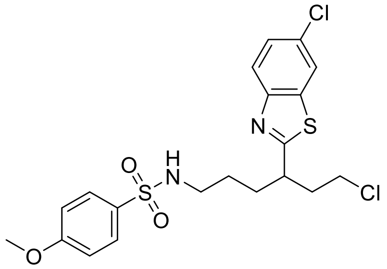

Prepared from 6-chlorobenzo[*d*]thiazole (33.8 mg, 0.2 mmol, 1.0 equiv) and *N*-(6-chlorohexyl)-4-methoxybenzenesulfonamide **2m** (122.0 mg, 0.4 mmol, 2.0 equiv) following **general procedure E**. The product was obtained as yellow oil (36 mg, 38% yield) after silica gel column chromatography using petroleum ether/EtOAc (4:1 to 3:1).

^{1}H NMR (400 MHz, $CDCl_3$) δ 7.86 (d, *J* = 8.7 Hz, 1H), 7.81 (s, 1H), 7.73 (d, *J* = 8.2 Hz, 2H), 7.42 (d, *J* = 8.7 Hz, 1H), 6.91 (d, *J* = 8.3 Hz, 2H), 4.89 (br, 1H), 3.83 (s, 3H), 3.53 (dt, *J* = 10.7, 5.4 Hz, 1H), 3.44 – 3.31 (m, 2H), 2.90 (t, *J* = 7.0 Hz, 2H), 2.34 – 2.06 (m, 2H), 1.90 – 1.74 (m, 2H), 1.55 – 1.36 (m, 2H).

^{13}C NMR (101 MHz, $CDCl_3$) δ 174.41, 162.83, 151.46, 135.70, 131.40, 131.06, 129.14, 126.97, 123.54, 121.28, 114.23, 55.62, 42.76, 42.24, 41.50, 38.23, 32.37, 27.08.

HRMS (ESI+) *m*/*z* Calcd for $C_{20}H_{23}Cl_2N_2O_3S_2^+$: 473.0522 [*M*+H]$^+$; found: 473.0529.

<u>ethyl 5-(6-chlorobenzo[*d*]thiazol-2-yl)-8-((4-methoxyphenyl)sulfonamido)octanoate (51)</u>

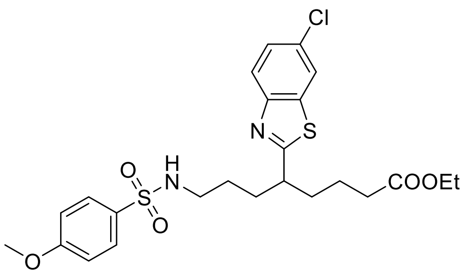

Prepared from 6-chlorobenzo[*d*]thiazole (33.8 mg, 0.2 mmol, 1.0 equiv) and ethyl 8-((4-methoxyphenyl)sulfonamido)octanoate **2n** (142.9 mg, 0.4 mmol, 2.0 equiv) following **general procedure E**. The product was obtained as yellow oil (47 mg, 45% yield) after silica gel column chromatography using petroleum ether/EtOAc (3:1 to 2:1).

^{1}H NMR (400 MHz, $CDCl_3$) δ 7.84 (d, *J* = 8.7 Hz, 1H), 7.80 – 7.77 (m, 1H), 7.72 (d, *J* = 8.7 Hz, 2H), 7.42 – 7.36 (m, 1H), 6.89 (d, *J* = 8.8 Hz, 2H), 4.97 (br, 1H), 4.07 (q, *J* = 7.1 Hz, 2H), 3.82 (s, 3H), 3.07 (p, *J* = 7.7 Hz, 1H), 2.88 (t, *J* = 6.8 Hz, 2H), 2.26 (t, *J* = 7.4 Hz, 2H), 1.82 – 1.70 (m, 4H), 1.65 – 1.33 (m, 4H), 1.20 (t, *J* = 7.1 Hz, 3H).

^{13}C NMR (101 MHz, $CDCl_3$) δ 176.20, 173.20, 162.77, 151.22, 135.74, 131.44, 130.85, 129.14, 126.81, 123.40, 121.26, 114.19, 60.39, 55.60, 44.41, 42.83, 35.49, 33.91, 32.64, 27.08, 22.53, 14.23.

HRMS (ESI+) *m*/*z* Calcd for $C_{24}H_{30}ClN_2O_5S_2^+$: 525.1279 [*M*+H]$^+$; found: 525.1289.

<u>6-((4-methoxyphenyl)sulfonamido)-3-(2-phenylquinolin-4-yl)hexyl 4-methylbenzoate (52)</u>

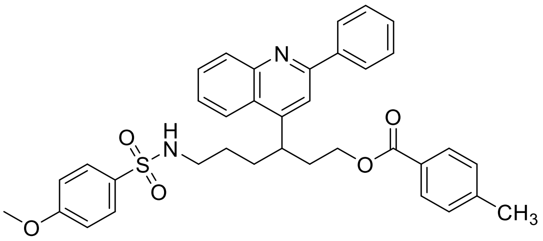

Prepared from 2-phenylquinoline (41.0 mg, 0.2 mmol, 1.0 equiv) and 6-((4-methoxyphenyl)sulfonamido)hexyl 4-methylbenzoate **2o** (162.1 mg, 0.4 mmol, 2.0 equiv) following **general procedure E**. The product was obtained as colorless oil (91 mg, 75% yield) after silica gel column chromatography using petroleum ether/EtOAc (3:1 to 2:1).

^{1}H NMR (400 MHz, $CDCl_3$) δ 8.20 (d, *J* = 8.4 Hz, 1H), 8.11 (d, *J* = 7.3 Hz, 2H), 8.04 (d, *J* = 8.5 Hz, 1H), 7.77 (d, *J* = 8.0 Hz, 2H), 7.74 – 7.63 (m, 4H), 7.54 – 7.49 (m, 2H), 7.49 – 7.42 (m, 2H), 7.19 (d, *J* = 8.0 Hz, 2H), 6.90 – 6.82 (m, 2H), 4.60 (t, *J* = 6.3 Hz, 1H), 4.29 (dt, *J* = 11.9, 6.2 Hz, 1H), 4.16 – 4.06 (m, 1H), 3.79 (s, 3H), 3.78 – 3.66 (m, 1H), 2.85 (q, *J* = 6.7 Hz, 2H), 2.40 (s, 3H), 2.31 – 2.19 (m, 2H), 2.01 – 1.83 (m, 2H), 1.46 – 1.23 (m, 2H).

^{13}C NMR (101 MHz, $CDCl_3$) δ 166.48, 162.78, 157.08, 151.26, 148.56, 143.71, 139.61, 131.36, 130.69, 129.54, 129.49, 129.40, 129.09, 129.07, 128.86, 127.64, 127.20, 126.61, 126.37, 122.53, 118.78, 114.18, 62.79, 55.57, 42.97, 35.30, 34.97, 32.99, 27.37, 21.69.

HRMS (ESI+) *m*/*z* Calcd for $C_{36}H_{37}N_2O_5S^+$: 609.2418 [*M*+H]$^+$; found: 609.2431.

<u>*N*-((3-(6-chlorobenzo[*d*]thiazol-2-yl)cyclohexyl)methyl)-4-methoxybenzenesulfonamide (53)</u>

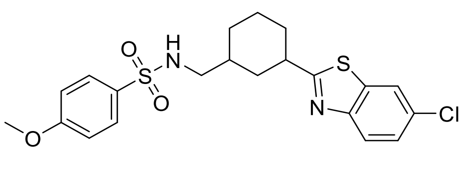

Prepared from 6-chlorobenzo[*d*]thiazole (33.8 mg, 0.2 mmol, 1.0 equiv) and *N*-(cyclohexylmethyl)-4-methoxybenzenesulfonamide **2p** (113.2 mg, 0.4 mmol, 2.0 equiv) following **general procedure E**. The product was obtained as yellow oil (59 mg, 65% yield) after silica gel column chromatography using petroleum ether/EtOAc (4:1 to 3:1).

^{1}H NMR (400 MHz, $CDCl_3$) δ 7.84 – 7.76 (m, 4H), 7.38 (d, *J* = 8.6 Hz, 1H), 6.94 (d, *J* = 8.0 Hz, 2H), 4.93 (t, *J* = 6.6 Hz, 1H), 3.82 (s, 3H), 3.02 (t, *J* = 11.1 Hz, 1H), 2.88 – 2.78 (m, 2H), 2.17 (t, *J* = 10.9 Hz, 2H), 1.90 (d, *J* = 12.5 Hz, 1H), 1.79 (d, *J* = 12.7 Hz, 1H), 1.72 – 1.59 (m, 1H), 1.55 – 1.33 (m, 2H), 1.29 – 1.19 (m, 1H), 1.01 – 0.85 (m, 1H).

^{13}C NMR (101 MHz, $CDCl_3$) δ 177.07, 162.83, 151.50, 135.65, 131.52, 130.56, 129.17, 126.70, 123.29, 121.21, 114.24, 55.59, 49.00, 42.69, 37.73, 36.75, 32.99, 29.69, 25.28.

HRMS (ESI+) *m/z* Calcd for $C_{21}H_{24}ClN_2O_3S_2^+$: 451.0911 [*M*+H]$^+$; found: 451.0918.

***N*-(2-(2-(6-chlorobenzo[*d*]thiazol-2-yl)cyclopentyl)ethyl)-4-methoxybenzenesulfonamide (54)**

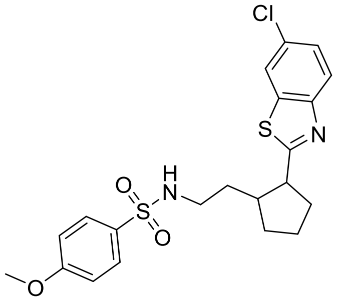

Prepared from 6-chlorobenzo[*d*]thiazole (33.8 mg, 0.2 mmol, 1.0 equiv) and *N*-(2-cyclopentylethyl)-4-methoxybenzenesulfonamide **2q** (113.2 mg, 0.4 mmol, 2.0 equiv) following **general procedure E**. The product was obtained as yellow oil (66 mg, 73% yield) after silica gel column chromatography using petroleum ether/EtOAc (4:1 to 3:1).

^{1}H NMR (400 MHz, $CDCl_3$) δ 7.83 (d, *J* = 8.6 Hz, 1H), 7.74 (s, 1H), 7.65 (d, *J* = 8.2 Hz, 2H), 7.37 (d, *J* = 8.6 Hz, 1H), 6.81 (d, *J* = 8.2 Hz, 2H), 5.76 (t, *J* = 6.2 Hz, 1H), 3.78 (s, 3H), 3.02 (q, *J* = 8.5 Hz, 1H), 2.89 (tq, *J* = 12.5, 6.0 Hz, 2H), 2.35 – 2.13 (m, 2H), 1.99 – 1.90 (m, 1H), 1.89 – 1.78 (m, 1H), 1.78 – 1.54 (m, 4H), 1.33 – 1.19 (m, 1H).

^{13}C NMR (101 MHz, $CDCl_3$) δ 177.00, 162.62, 151.30, 135.86, 131.58, 130.66, 129.04, 126.79, 123.27, 121.17, 114.07, 55.58, 50.20, 44.18, 41.87, 34.94, 34.54, 32.79, 24.50.

HRMS (ESI+) *m/z* Calcd for $C_{21}H_{24}ClN_2O_3S_2^+$: 451.0911 [*M*+H]$^+$; found: 451.0916.

***N*-(2-(2-(6-chlorobenzo[*d*]thiazol-2-yl)cyclohexyl)ethyl)-4-methoxybenzenesulfonamide (55)**

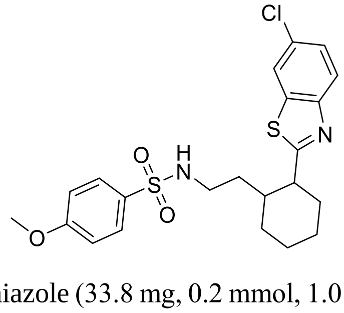

Prepared from 6-chlorobenzo[*d*]thiazole (33.8 mg, 0.2 mmol, 1.0 equiv) and *N*-(2-cyclohexylethyl)-4-methoxybenzenesulfonamide **2r** (118.9 mg, 0.4 mmol, 2.0 equiv) following **general procedure E**. The product was obtained as yellow oil (71 mg, 76% yield) after silica gel column

chromatography using petroleum ether/EtOAc (4:1 to 3:1).

^{1}H NMR (400 MHz, $CDCl_3$) δ 7.90 (d, *J* = 8.6 Hz, 1H), 7.78 (s, 1H), 7.70 (d, *J* = 7.9 Hz, 2H), 7.41 (d, *J* = 8.6 Hz, 1H), 6.86 (d, *J* = 7.9 Hz, 2H), 5.47 (br, 1H), 3.82 (s, 3H), 2.98 – 2.72 (m, 3H), 2.04 (d, *J* = 11.5 Hz, 1H), 1.88 – 1.64 (m, 4H), 1.58 – 1.35 (m, 2H), 1.33 – 1.17 (m, 3H), 1.08 – 0.93 (m, 1H).

^{13}C NMR (101 MHz, $CDCl_3$) δ 177.06, 162.66, 151.04, 135.62, 131.45, 130.85, 129.14, 126.89, 123.42, 121.24, 114.07, 55.60, 48.76, 40.68, 39.36, 35.49, 33.82, 31.99, 25.94, 25.62.

HRMS (ESI+) *m/z* Calcd for $C_{22}H_{26}ClN_2O_3S_2^+$: 465.1068 [*M*+H]$^+$; found: 465.1086.

<u>*N*-(3-(1-(6-chlorobenzo[*d*]thiazol-2-yl)cyclopentyl)propyl)-4-methoxybenzenesulfonamide (56)</u>

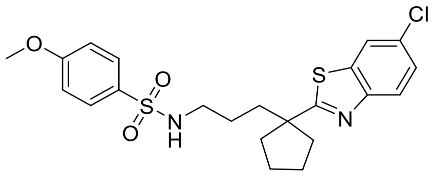

Prepared from 6-chlorobenzo[*d*]thiazole (33.8 mg, 0.2 mmol, 1.0 equiv) and *N*-(3-cyclopentylpropyl)-4-methoxybenzenesulfonamide **2s** (118.9 mg, 0.4 mmol, 2.0 equiv) following **general procedure E**. The product was obtained as yellow oil (64 mg, 69% yield) after silica gel column chromatography using petroleum ether/EtOAc (4:1 to 3:1).

^{1}H NMR (400 MHz, $CDCl_3$) δ 7.87 (dd, *J* = 8.7, 2.4 Hz, 1H), 7.77 (s, 1H), 7.71 (d, *J* = 8.1 Hz, 2H), 7.38 (dt, *J* = 7.3, 1.9 Hz, 1H), 6.92 – 6.82 (m, 2H), 5.10 (t, *J* = 5.9 Hz, 1H), 3.82 (s, 3H), 2.84 (q, *J* = 6.0 Hz, 2H), 2.22 – 2.13 (m, 2H), 1.91 – 1.65 (m, 8H), 1.47 – 1.29 (m, 2H).

^{13}C NMR (101 MHz, $CDCl_3$) δ 180.86, 162.74, 151.42, 136.19, 131.48, 130.58, 129.14, 126.63, 123.45, 121.05, 114.14, 55.60, 53.31, 43.28, 39.53, 38.17, 25.52, 24.38.

HRMS (ESI+) *m/z* Calcd for $C_{22}H_{26}ClN_2O_3S_2^+$: 465.1068 [*M*+H]$^+$; found: 465.1065.

<u>*N*-(3-(1-(6-chlorobenzo[*d*]thiazol-2-yl)cyclohexyl)propyl)-4-methoxybenzenesulfonamide (57)</u>

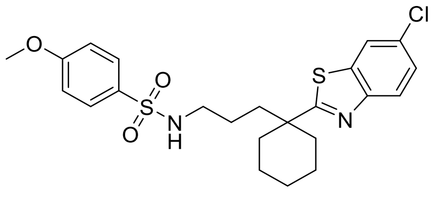

Prepared from 6-chlorobenzo[*d*]thiazole (33.8 mg, 0.2 mmol, 1.0 equiv) and *N*-(3-cyclohexylpropyl)-4-methoxybenzenesulfonamide **2t** (124.5 mg, 0.4 mmol, 2.0 equiv) following **general procedure E**. The product was obtained as yellow oil (71 mg, 74% yield) after silica gel column chromatography using petroleum ether/EtOAc (4:1 to 3:1).

^{1}H NMR (400 MHz, $CDCl_3$) δ 7.86 (d, *J* = 8.6 Hz, 1H), 7.78 (s, 1H), 7.70 (d, *J* = 8.1 Hz, 2H), 7.38 (dt, *J* = 8.8, 2.1 Hz, 1H), 6.88 (d, *J* = 8.1 Hz, 2H), 4.92 (t, *J* = 5.9 Hz, 1H), 3.82 (s, 3H), 2.80 (q, *J* = 6.1 Hz, 2H), 2.15 – 2.05 (m, 2H), 1.72 – 1.64 (m, 2H), 1.65 – 1.51 (m, 4H), 1.51 – 1.35 (m, 4H), 1.31 – 1.22 (m, 2H).

^{13}C NMR (101 MHz, $CDCl_3$) δ 180.28, 162.75, 151.41, 136.12, 131.45, 130.56, 129.13, 126.49, 123.50, 121.10, 114.15, 55.60, 45.11, 43.37, 39.09, 36.94, 25.74, 23.76, 22.31.

HRMS (ESI+) *m/z* Calcd for $C_{23}H_{28}ClN_2O_3S_2^+$: 479.1224 [*M*+H]$^+$; found: 479.1239.

<u>*N*-(4-(6-chlorobenzo[*d*]thiazol-2-yl)-4-methylpentyl)-4-methoxybenzenesulfonamide (58)</u>

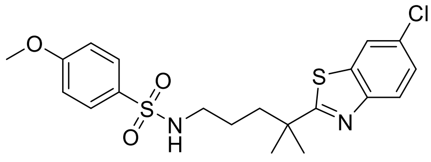

Prepared from 6-chlorobenzo[*d*]thiazole (33.8 mg, 0.2 mmol, 1.0 equiv) and 4-methoxy-*N*-(4-methylpentyl)benzenesulfonamide **2u** (108.4 mg, 0.4 mmol, 2.0 equiv) following **general procedure E**. The product was obtained as yellow oil (72 mg, 82% yield) after silica gel column chromatography using petroleum ether/EtOAc (4:1 to 3:1).

^{1}H NMR (400 MHz, $CDCl_3$) δ 7.89 (d, *J* = 8.7 Hz, 1H), 7.78 (s, 1H), 7.73 (dd, *J* = 8.9, 2.3 Hz, 2H), 7.38 (dt, *J* = 8.8, 2.2 Hz, 1H), 6.88 (dd, *J* = 8.9, 2.4 Hz, 2H), 5.27 (br, 1H), 3.82 (s, 3H), 2.86 (t, *J* = 6.8 Hz, 2H), 1.86 – 1.64 (m, 2H), 1.43 – 1.35 (m, 8H).

^{13}C NMR (101 MHz, $CDCl_3$) δ 181.22, 162.74, 151.38, 136.03, 131.46, 130.62, 129.15, 126.70, 123.51, 121.08, 114.15, 55.60, 43.28, 41.37, 39.83, 28.75, 24.69.

HRMS (ESI+) *m*/*z* Calcd for $C_{20}H_{24}ClN_2O_3S_2^+$: 439.0911 [*M*+H]$^+$; found: 439.0925.

<u>*N*-(4-(6-chlorobenzo[*d*]thiazol-2-yl)butyl)-4-methoxybenzenesulfonamide (59)</u>

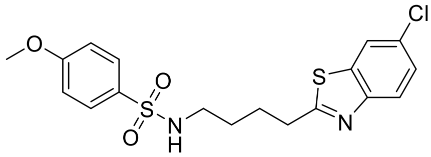

Prepared from 6-chlorobenzo[*d*]thiazole (33.8 mg, 0.2 mmol, 1.0 equiv) and 4-methoxy-*N*-pentylbenzenesulfonamide **2v** (97.2 mg, 0.4 mmol, 2.0 equiv) following **general procedure E**. The product was obtained as yellow oil (18 mg, 22% yield) after silica gel column chromatography using petroleum ether/EtOAc (4:1 to 3:1).

^{1}H NMR (400 MHz, $CDCl_3$) δ 7.87 (d, *J* = 8.6 Hz, 1H), 7.83 – 7.72 (m, 3H), 7.41 (d, *J* = 8.7 Hz, 1H), 6.94 (d, *J* = 7.8 Hz, 2H), 4.78 (br, 1H), 3.84 (s, 3H), 3.05 (t, *J* = 7.2 Hz, 2H), 2.97 (t, *J* = 7.0 Hz, 2H), 1.88 (p, *J* = 7.3 Hz, 2H), 1.60 (p, *J* = 7.3 Hz, 2H).

^{13}C NMR (101 MHz, $CDCl_3$) δ 171.84, 162.84, 151.57, 136.20, 131.45, 130.80, 129.18, 126.84, 123.29, 121.15, 114.23, 55.61, 42.68, 33.39, 28.77, 25.94.

HRMS (ESI+) *m*/*z* Calcd for $C_{18}H_{20}ClN_2O_3S_2^+$: 411.0598 [*M*+H]$^+$; found: 411.0605.

<u>4-methoxy-*N*-(3-methyl-4-(2-phenylquinolin-4-yl)butyl)benzenesulfonamide (60)</u>

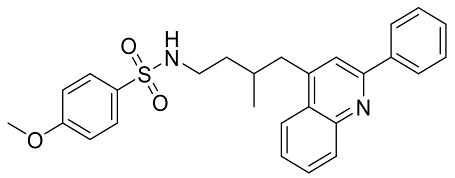

Prepared from 2-phenylquinoline (41.0 mg, 0.2 mmol, 1.0 equiv) and *N*-isopentyl-4-methoxybenzenesulfonamide **2w** (102.8 mg, 0.4 mmol, 2.0 equiv) following **general procedure E**. The product was obtained as yellow oil (26 mg, 28% yield) after silica gel column chromatography using petroleum ether/EtOAc (4:1 to 3:1).

^{1}H NMR (400 MHz, $CDCl_3$) δ 8.21 (d, *J* = 8.5 Hz, 1H), 8.13 (d, *J* = 6.7 Hz, 2H), 7.96 (d, *J* = 8.4 Hz, 1H), 7.79 – 7.67 (m, 3H), 7.61 (s, 1H), 7.56 – 7.43 (m, 4H), 6.89 (dd, *J* = 9.0, 2.3 Hz, 2H), 4.68

(t, *J* = 6.1 Hz, 1H), 3.80 (s, 3H), 3.13 – 3.01 (m, 2H), 2.97 – 2.79 (m, 2H), 2.10 – 1.98 (m, 1H), 1.66 – 1.55 (m, 1H), 1.49 – 1.37 (m, 1H), 0.88 (d, *J* = 6.6 Hz, 3H).

^{13}C NMR (101 MHz, $CDCl_3$) δ 162.82, 156.74, 148.42, 147.37, 139.56, 131.26, 130.38, 129.42, 129.34, 129.14, 128.84, 127.61, 126.67, 126.22, 123.56, 119.92, 114.23, 55.59, 41.13, 40.01, 36.64, 31.45, 19.48.

HRMS (ESI+) *m/z* Calcd for $C_{27}H_{29}N_2O_3S^+$: 461.1893 [*M*+H]$^+$; found: 461.1905.

3-(cyclopropylmethoxy)-*N*-(3,5-dichloro-2-(5-((4-methoxyphenyl)sulfonamido)pentan-2-yl)pyridin-4-yl)-4-(difluoromethoxy)benzamide (61)

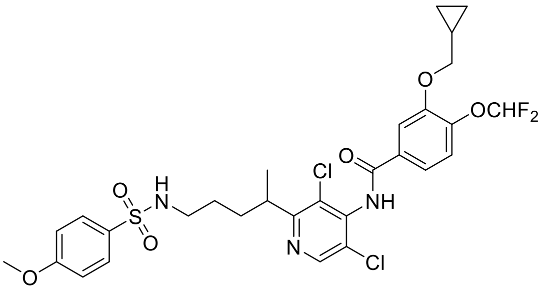

Prepared from 3-(cyclopropylmethoxy)-*N*-(3,5-dichloropyridin-4-yl)-4-(difluoromethoxy)benzamide (80.4 mg, 0.2 mmol, 1.0 equiv) and 4-methoxy-*N*-pentylbenzenesulfonamide **2a** (102.8 mg, 0.4 mmol, 2.0 equiv) following **general procedure E**. The product was obtained as yellow oil (45 mg, 34% yield) after silica gel column chromatography using petroleum DCM/MeOH (40:1 to 30:1).

^{1}H NMR (400 MHz, $CDCl_3$) δ 8.46 (s, 1H), 8.09 (s, 1H), 7.72 (d, *J* = 8.8 Hz, 2H), 7.59 (d, *J* = 1.9 Hz, 1H), 7.50 (dd, *J* = 8.3, 2.0 Hz, 1H), 7.24 (d, *J* = 8.2 Hz, 1H), 6.94 (s, 2H), 6.92 – 6.73 (m, 1H), 4.85 (s, 1H), 3.93 (d, *J* = 7.0 Hz, 2H), 3.84 (s, 3H), 3.42 – 3.29 (m, 1H), 2.88 – 2.77 (m, 2H), 1.82 – 1.69 (m, 1H), 1.59 – 1.46 (m, 1H), 1.44 – 1.32 (m, 1H), 1.35 – 1.23 (m, 2H), 1.18 (d, *J* = 6.8 Hz, 3H), 0.69 – 0.60 (m, 2H), 0.39 – 0.30 (m, 2H).

^{13}C NMR (101 MHz, $CDCl_3$) δ 164.11, 162.79, 161.72, 150.74, 147.11, 143.67 (t, $J_{F\text{-}C}$ = 3.1 Hz), 139.84, 131.31, 130.95, 129.13, 128.37, 127.06, 122.17, 120.18, 115.73 (t, $J_{F\text{-}C}$ = 261.2 Hz), 114.23, 114.15, 74.15, 55.65, 43.12, 36.82, 32.45, 27.26, 19.68, 10.01, 3.28.

^{19}F NMR (376 MHz, $CDCl_3$) δ -81.97.

HRMS (ESI+) *m/z* Calcd for $C_{29}H_{32}Cl_2F_2N_3O_6S^+$: 658.1351 [*M*+H]$^+$; found: 658.1353.

***N*-(4-(4-((2*S*,3*R*)-3-(2,4-difluorophenyl)-3-hydroxy-4-(1*H*-1,2,4-triazol-1-yl)butan-2-yl)-5-fluoropyrimidin-2-yl)pentyl)-4-methoxybenzenesulfonamide (62)**

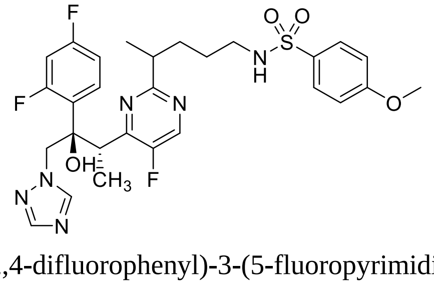

Prepared from (2*R*,3*S*)-2-(2,4-difluorophenyl)-3-(5-fluoropyrimidin-4-yl)-1-(1*H*-1,2,4-triazol-1-yl)butan-2-ol (69.8 mg, 0.2 mmol, 1.0 equiv) and 4-methoxy-*N*-pentylbenzenesulfonamide **2a** (102.8 mg, 0.4 mmol, 2.0 equiv) following **general procedure E**. The product was obtained as colorless oil (71 mg, 59% yield) after silica gel column chromatography using petroleum

DCM/MeOH (30:1 to 20:1).

^{1}H NMR (600 MHz, $CDCl_3$) δ 8.76 (t, *J* = 1.9 Hz, 1H), 7.99 (d, *J* = 3.0 Hz, 1H), 7.73 (dd, *J* = 8.8, 4.1 Hz, 2H), 7.64 – 7.57 (m, 1H), 7.51 – 7.48 (m, 1H), 6.93 (dd, *J* = 9.0, 2.7 Hz, 2H), 6.81 (ddt, *J* = 8.6, 6.0, 2.4 Hz, 2H), 6.71 (d, *J* = 22.1 Hz, 1H), 5.20 (t, *J* = 6.1 Hz, 0.5H), 4.99 (t, *J* = 6.1 Hz, 0.5H), 4.67 (d, *J* = 14.3 Hz, 1H), 4.32 (dd, *J* = 24.9, 14.3 Hz, 1H), 4.08 (dt, *J* = 11.3, 5.7 Hz, 1H), 3.84 (d, *J* = 3.5 Hz, 3H), 3.21 (dh, *J* = 27.7, 6.7 Hz, 1H), 2.89 (dq, *J* = 13.2, 6.7 Hz, 2H), 1.86 – 1.75 (m, 1H), 1.68 – 1.57 (m, 1H), 1.49 (ddp, *J* = 19.9, 12.9, 6.7 Hz, 1H), 1.42 – 1.29 (m, 1H), 1.28 – 1.22 (m, 3H), 1.05 (dd, *J* = 7.1, 2.3 Hz, 3H).

^{13}C NMR (151 MHz, $CDCl_3$) δ 163.60, 163.52, 162.82, 162.79, 162.41, 162.38, 162.32, 162.28, 161.95, 161.87, 159.37, 159.29, 158.19, 158.14, 158.10, 158.04, 152.82, 152.81, 152.76, 152.74, 150.73, 150.71, 144.12, 144.04, 131.46, 130.68, 130.64, 130.62, 130.58, 129.13, 123.85, 123.83, 123.77, 123.74, 123.72, 123.70, 123.65, 123.62, 114.21, 111.64, 111.62, 111.51, 111.49, 104.26, 104.07, 103.90, 77.53, 77.50, 77.46, 57.54, 57.52, 57.48, 55.61, 42.94, 36.48, 36.45, 36.42, 36.39, 34.20, 33.84, 31.85, 31.62, 27.49, 27.25, 19.10, 19.00, 16.38, 16.28.

^{19}F NMR (376 MHz, $CDCl_3$) δ -109.00 (d, *J* = 8.1 Hz, 0.5H), -109.05 (d, *J* = 8.1 Hz, 0.5H), -110.43 (d, *J* = 8.1 Hz, 0.5H), -110.47 (d, *J* = 8.1 Hz, 0.5H), -139.00 (s, 0.5H), -139.15 (s, 0.5H).

HRMS (ESI+) *m/z* Calcd for $C_{28}H_{32}F_3N_6O_4S^+$: 605.2152 [*M*+H]$^+$; found: 605.2150.

2-((2-(4-chlorophenoxy)-2-methylpropanoyl)oxy)ethyl 6-(5-((4-methoxyphenyl)sulfonamido)pentan-2-yl)nicotinate (63)

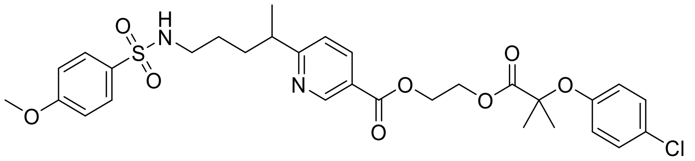

Prepared from 2-((2-(4-chlorophenoxy)-2-methylpropanoyl)oxy)ethyl nicotinate (72.6 mg, 0.2 mmol, 1.0 equiv) and 4-methoxy-*N*-pentylbenzenesulfonamide **2a** (102.8 mg, 0.4 mmol, 2.0 equiv) following **general procedure E**. The product was obtained as colorless oil (49 mg, 40% yield) after silica gel column chromatography using petroleum PE/EtOAc (4:1 to 2:1).

^{1}H NMR (600 MHz, $CDCl_3$) δ 9.00 (d, *J* = 2.2 Hz, 1H), 7.99 (dd, *J* = 8.2, 2.2 Hz, 1H), 7.74 (d, *J* = 8.9 Hz, 2H), 7.13 (d, *J* = 8.1 Hz, 1H), 7.09 – 7.05 (m, 2H), 6.95 – 6.90 (m, 2H), 6.75 – 6.72 (m, 2H), 4.85 (t, *J* = 6.1 Hz, 1H), 4.51 (ddt, *J* = 8.2, 6.4, 2.1 Hz, 4H), 3.83 (s, 3H), 2.87 (qt, *J* = 6.5, 2.9 Hz, 3H), 1.76 – 1.68 (m, 1H), 1.58 (s, 7H), 1.47 – 1.38 (m, 1H), 1.29 (dq, *J* = 17.0, 6.2, 5.7 Hz, 1H), 1.23 (d, *J* = 6.9 Hz, 3H).

^{13}C NMR (151 MHz, $CDCl_3$) δ 173.91, 170.63, 164.94, 162.77, 153.91, 150.43, 137.57, 131.49, 129.15, 129.12, 127.14, 123.23, 121.40, 120.23, 114.19, 79.34, 63.06, 62.60, 55.61, 43.08, 41.51, 33.52, 27.45, 25.31, 20.66.

HRMS (ESI+) *m/z* Calcd for $C_{30}H_{36}ClN_2O_8S^+$: 619.1875 [*M*+H]$^+$; found: 619.1878.

(2*R*)-1,7,7-trimethylbicyclo[2.2.1]heptan-2-yl 7-((4-methoxyphenyl)sulfonamido)-4-(2-phenylquinolin-4-yl)heptanoate (64)

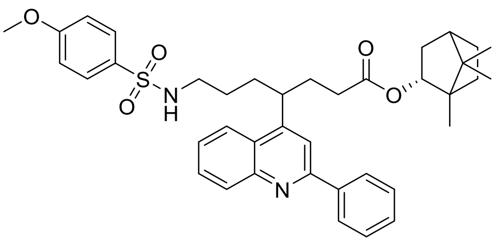

Prepared from 2-phenylquinoline (41.0 mg, 0.2 mmol, 1.0 equiv) and (2*R*)-1,7,7-trimethylbicyclo[2.2.1]heptan-2-yl 7-((4-methoxyphenyl)sulfonamido)heptanoate **2aa** (180.5 mg, 0.4 mmol, 2.0 equiv) following **general procedure E**. The product was obtained as white solid (90 mg, 69% yield) after silica gel column chromatography using petroleum ether/EtOAc (5:1 to 2:1).

^{1}H NMR (400 MHz, $CDCl_3$) δ 8.21 (d, *J* = 8.4 Hz, 1H), 8.14 – 8.09 (m, 2H), 8.01 (d, *J* = 8.5 Hz, 1H), 7.74 – 7.63 (m, 4H), 7.54 – 7.42 (m, 4H), 6.86 – 6.82 (m, 2H), 4.93 (t, *J* = 6.2 Hz, 1H), 4.83 (ddt, *J* = 9.9, 3.7, 2.1 Hz, 1H), 3.79 (s, 3H), 3.69 – 3.46 (m, 1H), 2.82 (q, *J* = 6.7 Hz, 2H), 2.34 – 2.24 (m, 1H), 2.21 – 2.11 (m, 3H), 2.09 – 2.01 (m, 1H), 1.92 – 1.73 (m, 3H), 1.72 – 1.60 (m, 2H), 1.44 – 1.06 (m, 5H), 0.87 (s, 3H), 0.83 (s, 3H), 0.74 (d, *J* = 8.9 Hz, 3H).

^{13}C NMR (101 MHz, $CDCl_3$) δ 173.58, 162.74, 157.12, 151.39, 148.59, 139.68, 131.37, 130.66, 129.51, 129.38, 129.06, 128.86, 127.65, 126.90, 126.41, 122.68, 115.86, 114.17, 80.05, 55.57, 48.69, 47.79, 44.80, 43.02, 36.73, 33.12, 32.11, 31.16, 27.98, 27.96, 27.33, 27.04, 19.70, 18.83, 13.55.

HRMS (ESI+) *m/z* Calcd for $C_{39}H_{47}N_2O_5S^+$: 655.3200 [*M*+H]$^+$; found 655.3203.

(2*S*,5*R*)-2-isopropyl-5-methylcyclohexyl 4-(6-chlorobenzo[*d*]thiazol-2-yl)-7-((4-methoxyphenyl)sulfonamido)heptanoate (65)

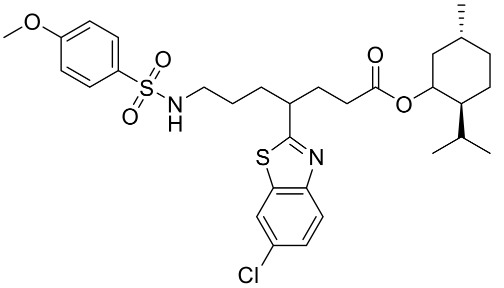

Prepared from 6-chlorobenzo[*d*]thiazole (33.8 mg,_0.2 mmol, 1.0 equiv) and (2*S*,5*R*)-2-isopropyl-5-methylcyclohexyl 7-((4-methoxyphenyl)sulfonamido)heptanoate **2ab** (181.3 mg, 0.4 mmol, 2.0 equiv) following **general procedure E**. The product was obtained as yellow solid (55 mg, 44% yield) after silica gel column chromatography using petroleum ether/EtOAc (4:1 to 3:1).

^{1}H NMR (400 MHz, $CDCl_3$) δ 7.86 (dd, *J* = 8.7, 2.2 Hz, 1H), 7.81 (t, *J* = 2.0 Hz, 1H), 7.76 – 7.70 (m, 2H), 7.42 (ddd, *J* = 8.6, 2.1, 1.0 Hz, 1H), 6.96 – 6.86 (m, 2H), 4.75 (td, *J* = 6.2, 2.3 Hz, 1H), 4.64 (tdd, *J* = 10.9, 4.4, 1.5 Hz, 1H), 3.83 (s, 3H), 3.16 – 3.07 (m, 1H), 2.91 (q, *J* = 6.6 Hz, 2H), 2.25 (t, *J* = 7.3 Hz, 2H), 2.12 – 2.00 (m, 2H), 1.96 – 1.85 (m, 1H), 1.84 – 1.75 (m, 2H), 1.70 – 1.60 (m, 2H), 1.55 – 1.38 (m, 3H), 1.35 – 1.24 (m, 2H), 1.08 – 0.95 (m, 1H), 0.90 – 0.83 (m, 8H), 0.72 (t, *J* = 7.1 Hz, 3H).

^{13}C NMR (101 MHz, $CDCl_3$) δ 175.21, 172.37, 162.80, 151.49, 135.81, 131.44, 130.94, 129.15, 126.86, 123.54, 121.26, 114.19, 74.38, 55.60, 46.92, 43.83, 43.81, 42.82, 42.80, 40.90, 40.87, 34.19, 32.59, 32.52, 32.02, 31.37, 31.36, 31.08, 31.01, 27.18, 26.23, 23.36, 22.03, 22.01, 20.78, 20.76, 16.32, 16.28.

HRMS (ESI+) *m/z* Calcd for $C_{31}H_{42}ClN_2O_5S_2^+$: 621.2218 [*M*+H]$^+$; found 621.2217.

((3a*S*,4a*R*,7a*S*,8a*S*)-2,2,6,6-tetramethyltetrahydro-3a*H*-bis([1,3]dioxolo)[4,5-*b*:4',5'-*e*]pyran-3a-yl)methyl 7-((4-methoxyphenyl)sulfonamido)-4-(2-phenylquinolin-4-yl)heptanoate (66)

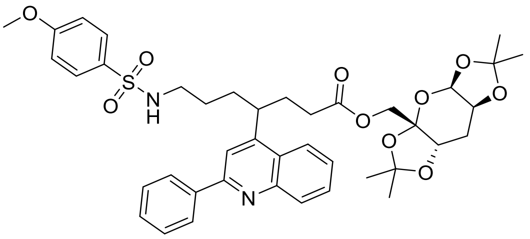

Prepared from 2-phenylquinoline (41.0 mg, 0.2 mmol, 1.0 equiv) and ((3a*S*,4a*R*,7a*S*,8a*S*)-2,2,6,6-tetramethyltetrahydro-3a*H*-bis([1,3]dioxolo)[4,5-*b*:4',5'-*e*]pyran-3a-yl)methyl 7-((4-methoxyphenyl)sulfonamido)heptanoate **2ac** (222.9 mg, 0.4 mmol, 2.0 equiv) following **general procedure E**. The product was obtained as colorless oil (119 mg, 78% yield) after silica gel column chromatography using petroleum ether/EtOAc (4:1 to 3:1).

^{1}H NMR (600 MHz, $CDCl_3$) δ 8.18 (d, *J* = 8.4 Hz, 1H), 8.09 (dd, *J* = 7.7, 2.3 Hz, 2H), 7.98 (d, *J* = 8.5 Hz, 1H), 7.67 (dd, *J* = 20.2, 8.2 Hz, 4H), 7.49 (q, *J* = 7.4, 7.0 Hz, 3H), 7.43 (t, *J* = 7.3 Hz, 1H), 6.83 (d, *J* = 8.8 Hz, 2H), 4.98 (q, *J* = 6.0 Hz, 1H), 4.54 – 4.44 (m, 1H), 4.37 – 4.29 (m, 1H), 4.16 (d, *J* = 7.9 Hz, 1H), 4.12 – 4.07 (m, 1H), 3.93 (dd, *J* = 11.6, 7.5 Hz, 1H), 3.77 (s, 3H), 3.68 (d, *J* = 13.0 Hz, 1H), 2.81 (q, *J* = 6.6 Hz, 2H), 2.23 – 2.11 (m, 3H), 2.08 – 1.98 (m, 1H), 1.89 – 1.70 (m, 2H), 1.53 – 1.31 (m, 9H), 1.26 (t, *J* = 8.9 Hz, 4H), 1.12 (d, *J* = 24.2 Hz, 3H).

^{13}C NMR (151 MHz, $CDCl_3$) δ 172.48, 162.73, 157.04, 157.02, 151.19, 148.60, 139.65, 131.41, 130.70, 129.47, 129.35, 129.04, 128.84, 127.60, 126.84, 126.44, 122.62, 115.61, 114.16, 109.03, 109.01, 108.65, 108.63, 101.36, 70.66, 70.65, 70.49, 69.93, 65.37, 61.17, 61.14, 55.57, 42.96, 36.99, 32.96, 31.64, 30.90, 27.27, 26.36, 25.83, 25.81, 24.98, 24.92, 24.02, 23.97.

HRMS (ESI+) *m/z* Calcd for $C_{41}H_{49}N_2O_{10}S^+$: 761.3102 $[M+H]^+$; found 761.3106.

***N*-(6-(1,1-dioxido-3-oxobenzo[*d*]isothiazol-2(3*H*)-yl)-4-(2-phenylquinolin-4-yl)hexyl)-4-methoxybenzenesulfonamide (67)**

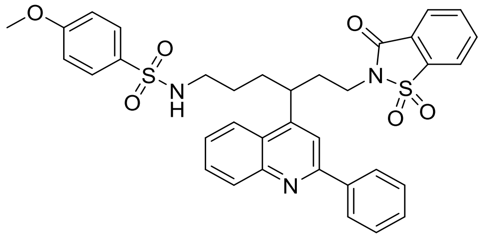

Prepared from 2-phenylquinoline (41.0 mg, 0.2 mmol, 1.0 equiv) and *N*-(6-(1,1-dioxido-3-oxobenzo[*d*]isothiazol-2(3*H*)-yl)hexyl)-4-methoxybenzenesulfonamide **2ad** (180.8 mg, 0.4 mmol, 2.0 equiv) following **general procedure E**. The product was obtained as colorless oil (98 mg, 75% yield) after silica gel column chromatography using petroleum ether/EtOAc (2:1 to 1:1).

^{1}H NMR (400 MHz, $CDCl_3$) δ 8.21 (dd, *J* = 8.5, 1.2 Hz, 1H), 8.17 (dd, *J* = 7.0, 1.5 Hz, 2H), 8.00 (d, *J* = 8.5 Hz, 1H), 7.98 – 7.94 (m, 1H), 7.88 – 7.85 (m, 1H), 7.84 – 7.69 (m, 4H), 7.68 – 7.64 (m, 2H), 7.54 – 7.42 (m, 4H), 6.86 – 6.82 (m, 2H), 4.79 (t, *J* = 6.2 Hz, 1H), 3.77 (s, 3H), 3.75 – 3.57 (m, 3H), 2.82 (q, *J* = 6.7 Hz, 2H), 2.45 – 2.27 (m, 2H), 1.95 – 1.74 (m, 2H), 1.44 – 1.27 (m, 2H).

^{13}C NMR (101 MHz, $CDCl_3$) δ 162.74, 158.79, 157.15, 150.34, 148.63, 139.62, 137.53, 134.85, 134.42, 131.46, 130.72, 129.54, 129.38, 129.08, 128.86, 127.72, 127.18, 126.45, 125.16, 122.62, 120.94, 115.97, 114.18, 55.57, 42.92, 37.56, 35.76, 33.66, 32.89, 27.01.

HRMS (ESI+) *m/z* Calcd for $C_{35}H_{34}N_3O_6S_2^+$: 656.1884 [*M*+H]+; found 656.1891.

6-((4-methoxyphenyl)sulfonamido)-3-(2-phenylquinolin-4-yl)hexyl 2-(4-isobutylphenyl)propanoate (68)

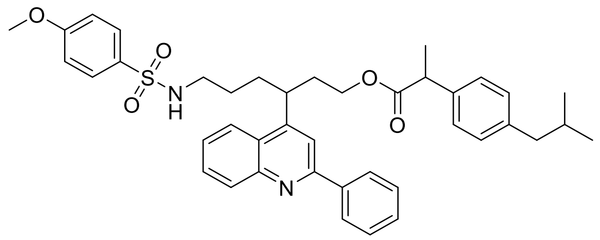

Prepared from 2-phenylquinoline (41.0 mg, 0.2 mmol, 1.0 equiv) and 6-((4-methoxyphenyl)sulfonamido)hexyl 2-(4-isobutylphenyl)propanoate **2ae** (190.1 mg, 0.4 mmol, 2.0 equiv) following **general procedure E**. The product was obtained as yellow oil (72 mg, 53% yield) after silica gel column chromatography using petroleum ether/EtOAc (4:1 to 2:1).

^{1}H NMR (400 MHz, $CDCl_3$) δ 8.21 (d, *J* = 8.4 Hz, 1H), 8.13 – 8.08 (m, 2H), 7.80 (dd, *J* = 25.8, 8.5 Hz, 1H), 7.75 – 7.60 (m, 4H), 7.56 – 7.37 (m, 4H), 7.18 – 7.05 (m, 4H), 6.88 – 6.83 (m, 2H), 4.62 – 4.52 (m, 1H), 4.15 – 4.01 (m, 1H), 3.85 – 3.73 (m, 4H), 3.64 – 3.39 (m, 2H), 2.80 (p, *J* = 7.1 Hz, 2H), 2.43 (d, *J* = 7.2 Hz, 2H), 2.11 – 1.98 (m, 2H), 1.88 – 1.67 (m, 3H), 1.41 (d, *J* = 7.1 Hz, 3H), 1.36 – 1.15 (m, 2H), 0.90 – 0.84 (m, 6H).

^{13}C NMR (101 MHz, $CDCl_3$) δ 174.55, 162.78, 157.01, 151.40, 148.50, 140.67, 140.58, 139.59, 137.94, 137.57, 131.38, 131.37, 130.62, 129.53, 129.42, 129.35, 129.06, 128.88, 127.64, 127.17, 127.14, 126.65, 126.39, 126.33, 122.61, 115.88, 114.19, 62.60, 62.35, 55.58, 45.18, 45.02, 45.00, 43.00, 42.97, 35.21, 35.12, 32.80, 32.77, 30.19, 27.34, 22.41, 22.38, 18.56, 18.23.

HRMS (ESI+) *m/z* Calcd for $C_{41}H_{47}N_2O_5S^+$: 679.3200 [*M*+H]+; found 679.3208.

6-((4-methoxyphenyl)sulfonamido)-3-(2-phenylquinolin-4-yl)hexyl 4-([1,1'-biphenyl]-4-yl)-4-oxobutanoate (69)

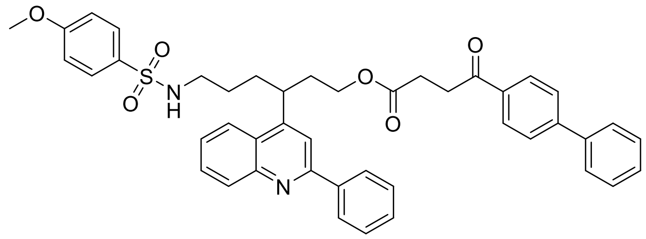

Prepared from 2-phenylquinoline (41.0 mg, 0.2 mmol, 1.0 equiv) and 6-((4-methoxyphenyl)sulfonamido)hexyl 4-([1,1'-biphenyl]-4-yl)-4-oxobutanoate **2af** (209.3 mg, 0.4 mmol, 2.0 equiv) following **general procedure E**. The product was obtained as yellow oil (109 mg, 75% yield) after silica gel column chromatography using petroleum ether/EtOAc (4:1 to 2:1).

^{1}H NMR (400 MHz, $CDCl_3$) δ 8.23 – 8.19 (m, 1H), 8.16 – 8.11 (m, 2H), 8.07 (dd, *J* = 8.7, 1.3 Hz, 1H), 8.03 – 8.00 (m, 2H), 7.73 – 7.65 (m, 6H), 7.64 – 7.60 (m, 2H), 7.54 – 7.44 (m, 6H), 7.43 – 7.38 (m, 1H), 6.87 – 6.82 (m, 2H), 4.90 (t, *J* = 6.2 Hz, 1H), 4.17 – 4.10 (m, 1H), 3.89 (dt, *J* = 11.2, 6.5 Hz, 1H), 3.78 (s, 3H), 3.75 – 3.62 (m, 1H), 3.34 – 3.16 (m, 2H), 2.90 – 2.80 (m, 2H), 2.74 – 2.58 (m, 2H), 2.14 (q, *J* = 6.8 Hz, 2H), 1.98 – 1.77 (m, 2H), 1.44 – 1.27 (m, 2H).

^{13}C NMR (101 MHz, $CDCl_3$) δ 197.93, 172.78, 162.72, 157.03, 151.39, 148.63, 145.94, 139.76, 139.68, 135.13, 131.50, 130.73, 129.51, 129.38, 129.06, 129.00, 128.88, 128.69, 128.32, 127.63,

127.27, 126.68, 126.37, 122.70, 115.90, 114.16, 62.62, 55.57, 42.95, 34.98, 33.37, 32.73, 28.23, 27.24.

HRMS (ESI+) *m/z* Calcd for $C_{44}H_{43}N_2O_6S^+$: 727.2836 [*M*+H]$^+$; found 727.2859.

2-oxo-1-phenyl-2-((3,3,5-trimethylcyclohexyl)oxy)ethyl 7-((4-methoxyphenyl)sulfonamido)-4-(2-phenylquinolin-4-yl)heptanoate (70)

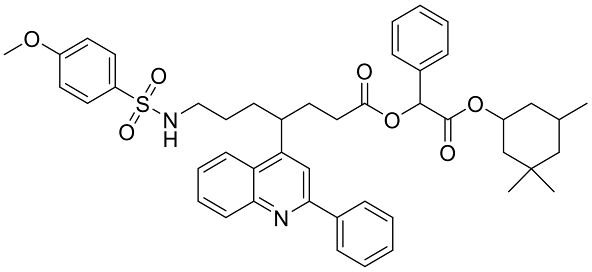

Prepared from 2-phenylquinoline (41.0 mg, 0.2 mmol, 1.0 equiv) and 2-oxo-1-phenyl-2-((3,3,5-trimethylcyclohexyl)oxy)ethyl 7-((4-methoxyphenyl)sulfonamido)heptanoate **2ag** (229.3 mg, 0.4 mmol, 2.0 equiv) following **general procedure E**. The product was obtained as colorless oil (110 mg, 71% yield) after silica gel column chromatography using petroleum ether/EtOAc (4:1 to 2:1).

^{1}H NMR (400 MHz, $CDCl_3$) δ 8.21 (dt, *J* = 8.5, 1.6 Hz, 1H), 8.12 (dt, *J* = 8.1, 1.6 Hz, 2H), 8.03 (dd, *J* = 16.1, 8.5 Hz, 1H), 7.73 – 7.65 (m, 4H), 7.53 – 7.42 (m, 4H), 7.42 – 7.31 (m, 5H), 6.86 – 6.81 (m, 2H), 5.82 (dd, *J* = 10.2, 2.3 Hz, 1H), 5.01 – 4.87 (m, 2H), 3.77 (d, *J* = 2.1 Hz, 3H), 3.71 – 3.41 (m, 1H), 2.87 – 2.79 (m, 2H), 2.43 – 2.09 (m, 4H), 2.05 – 1.95 (m, 0.5H), 1.94 – 1.55 (m, 4H), 1.53 – 1.45 (m, 0.5H), 1.42 – 1.26 (m, 3H), 1.16 – 0.95 (m, 1H), 0.95 – 0.86 (m, 8H), 0.83 (dd, *J* = 6.5, 2.5 Hz, 2H), 0.77 – 0.65 (m, 1H).

^{13}C NMR (101 MHz, $CDCl_3$) δ 172.62, 172.58, 168.46, 168.44, 168.38, 168.37, 162.71, 157.05, 151.29, 148.60, 139.66, 133.79, 133.77, 133.74, 131.52, 131.50, 130.66, 129.51, 129.37, 129.15, 129.07, 128.87, 128.76, 128.74, 127.66, 127.57, 127.54, 127.52, 126.83, 126.41, 122.75, 115.75, 114.16, 74.84, 74.82, 74.80, 74.77, 72.95, 72.92, 55.57, 47.39, 43.65, 43.33, 42.96, 40.10, 39.74, 32.98, 32.94, 32.70, 32.61, 32.30, 32.24, 31.80, 31.64, 31.11, 27.24, 27.06, 26.98, 25.48, 25.46, 25.44, 22.27, 22.21.

HRMS (ESI+) *m/z* Calcd for $C_{46}H_{53}N_2O_7S^+$: 777.3568 [*M*+H]$^+$; found 777.3514.

6-((4-methoxyphenyl)sulfonamido)-3-(2-phenylquinolin-4-yl)hexyl 2-(4-(4-chlorobenzoyl)phenoxy)-2-methylpropanoate (71)

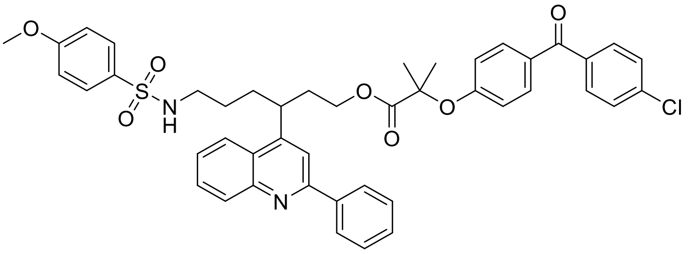

Prepared from 2-phenylquinoline (41.0 mg, 0.2 mmol, 1.0 equiv) and 6-((4-methoxyphenyl)sulfonamido)hexyl 2-(4-(4-chlorobenzoyl)phenoxy)-2-methylpropanoate **2ah** (234.9 mg, 0.4 mmol, 2.0 equiv) following **general procedure E**. The product was obtained as yellow oil (104 mg, 66% yield) after silica gel column chromatography using petroleum

ether/EtOAc (3:1 to 2:1).

^{1}H NMR (400 MHz, $CDCl_3$) δ 8.17 (d, *J* = 8.4 Hz, 1H), 8.11 – 8.07 (m, 2H), 7.83 (d, *J* = 7.2 Hz, 1H), 7.74 – 7.62 (m, 8H), 7.53 – 7.40 (m, 6H), 6.89 – 6.84 (m, 4H), 5.14 (t, *J* = 6.3 Hz, 1H), 4.31 – 4.23 (m, 1H), 3.83 – 3.78 (m, 4H), 3.44 – 3.19 (m, 1H), 2.75 (q, *J* = 6.7 Hz, 2H), 2.07 – 1.98 (m, 2H), 1.83 – 1.69 (m, 2H), 1.66 (s, 3H), 1.60 (s, 3H), 1.12 – 1.03 (m, 2H).

^{13}C NMR (101 MHz, $CDCl_3$) δ 194.67, 173.49, 162.69, 159.79, 157.05, 151.04, 148.54, 139.53, 138.74, 135.99, 132.14, 131.56, 131.36, 130.68, 130.29, 129.52, 129.44, 129.09, 128.88, 128.63, 127.58, 126.63, 126.23, 122.64, 116.80, 115.69, 114.12, 79.35, 63.46, 55.57, 43.09, 35.19, 34.62, 32.85, 27.30, 26.65, 24.07.

HRMS (ESI+) *m/z* Calcd for $C_{45}H_{44}ClN_2O_7S^+$: 791.2552 [*M*+H]$^+$; found 791.2562.

<u>***N*-(6-((4-methoxyphenyl)sulfonamido)-3-(2-phenylquinolin-4-yl)hexyl)-*N*-methyl-4-(5-(*p*-tolyl)-3-(trifluoromethyl)-1*H*-pyrazol-1-yl)benzenesulfonamide (72)**</u>

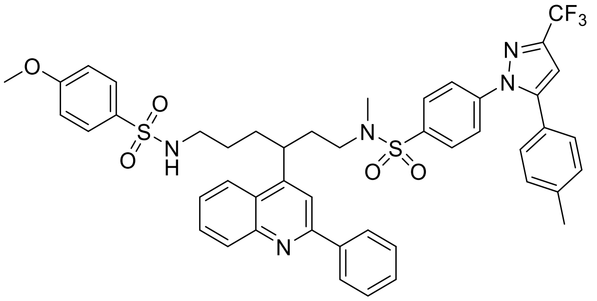

Prepared from 2-phenylquinoline (41.0 mg, 0.2 mmol, 1.0 equiv) and *N*-(6-((4-methoxyphenyl)sulfonamido)hexyl)-*N*-methyl-4-(5-(*p*-tolyl)-3-(trifluoromethyl)-1*H*-pyrazol-1-yl)benzenesulfonamid*e* **2ai** (265.7 mg, 0.4 mmol, 2.0 equiv) following **general procedure E**. The product was obtained as colorless oil (56 mg, 32% yield) after silica gel column chromatography using petroleum ether/EtOAc (3:1 to 2:1).

^{1}H NMR (400 MHz, $CDCl_3$) δ 8.19 (d, *J* = 8.4 Hz, 1H), 8.13 (d, *J* = 7.0 Hz, 2H), 8.01 (d, *J* = 8.5 Hz, 1H), 7.73 – 7.63 (m, 6H), 7.55 – 7.36 (m, 6H), 7.16 – 7.03 (m, 4H), 6.89 – 6.84 (m, 2H), 6.73 (s, 1H), 5.01 (t, *J* = 6.3 Hz, 1H), 3.80 (s, 3H), 3.71 – 3.47 (m, 1H), 3.16 – 3.01 (m, 1H), 2.83 (q, *J* = 6.5 Hz, 3H), 2.64 (s, 3H), 2.34 (s, 3H), 2.08 – 1.90 (m, 3H), 1.88 – 1.74 (m, 1H), 1.39 – 1.28 (m, 2H).

^{19}F NMR (376 MHz, $CDCl_3$) δ -62.37.

^{13}C NMR (101 MHz, $CDCl_3$) δ 162.74, 157.03, 151.15, 148.65, 145.28, 144.04 (q, *J* = 38.5 Hz), 142.50, 139.81, 139.58, 136.66, 131.46, 130.73, 129.74, 129.55, 129.42, 129.09, 128.88, 128.68, 128.26, 127.61, 126.51, 126.41, 125.62, 125.55, 122.51, 121.07 (q, *J* = 269.2 Hz), 115.77, 114.19, 106.25, 55.59, 48.44, 42.71, 35.05, 34.73, 33.90, 31.81, 27.05, 21.33.

HRMS (ESI+) *m/z* Calcd for $C_{46}H_{45}F_3N_5O_5S_2^+$: 868.2809 [*M*+H]$^+$; found 868.2807.

<u>**6-chloro-2-cyclopentylbenzo[*d*]thiazole (73)**</u>

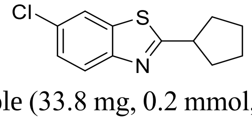

Prepared from 6-chlorobenzo[*d*]thiazole (33.8 mg, 0.2 mmol, 1.0 equiv) and cyclopentane (0.2 mL) following **general procedure F**. The product was obtained as yellow solid (28 mg, 60% yield) after silica gel column chromatography using petroleum ether/EtOAc (50:1 to 40:1).

^{1}H NMR (600 MHz, $CDCl_3$) δ 7.85 (d, *J* = 8.7 Hz, 1H), 7.79 (d, *J* = 2.1 Hz, 1H), 7.39 (dd, *J* = 8.7, 2.1 Hz, 1H), 3.53 (p, *J* = 8.2 Hz, 1H), 2.29 – 2.20 (m, 2H), 1.97 – 1.82 (m, 4H), 1.78 – 1.70 (m, 2H).
^{13}C NMR (151 MHz, $CDCl_3$) δ 177.73, 151.66, 136.00, 130.43, 126.60, 123.19, 121.10, 44.73, 34.00, 25.59.
HRMS (ESI+) *m/z* Calcd for $C_{12}H_{13}ClNS^+$: 238.0452 [*M*+H]$^+$; found 238.0457.

6-chloro-2-cyclooctylbenzo[*d*]thiazole (74)

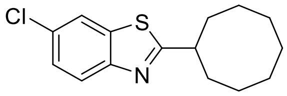

Prepared from 6-chlorobenzo[*d*]thiazole (33.8 mg, 0.2 mmol, 1.0 equiv) and cyclooctane (0.2 mL) following **general procedure F**. The product was obtained as yellow oil (32 mg, 57% yield) after silica gel column chromatography using petroleum ether/EtOAc (50:1 to 40:1).
^{1}H NMR (600 MHz, $CDCl_3$) δ 7.86 (d, *J* = 8.7 Hz, 1H), 7.82 – 7.78 (m, 1H), 7.41 – 7.37 (m, 1H), 3.35 (tt, *J* = 9.2, 3.8 Hz, 1H), 2.18– 2.12 (m, 2H), 1.99 – 1.91 (m, 2H), 1.85 – 1.79 (m, 2H), 1.68 – 1.59 (m, 8H).
^{13}C NMR (151 MHz, $CDCl_3$) δ 179.68, 151.51, 135.92, 130.38, 126.55, 123.25, 121.11, 43.68, 32.79, 26.89, 26.09, 25.38.
HRMS (ESI+) *m/z* Calcd for $C_{15}H_{19}ClNS^+$: 280.0921 [*M*+H]$^+$; found 280.0928.

6-chloro-2-cyclododecylbenzo[*d*]thiazole (75)

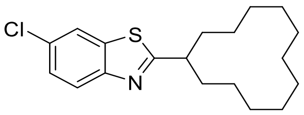

Prepared from 6-chlorobenzo[*d*]thiazole (33.8 mg, 0.2 mmol, 1.0 equiv) and cyclododecane (0.2 mL) following **general procedure F**. The product was obtained as yellow oil (42 mg, 63% yield) after silica gel column chromatography using petroleum ether/EtOAc (55:1 to 45:1).
^{1}H NMR (400 MHz, $CDCl_3$) δ 7.87 (d, *J* = 8.7 Hz, 1H), 7.80 (d, *J* = 2.0 Hz, 1H), 7.38 (dd, *J* = 8.7, 2.0 Hz, 1H), 3.33 (p, *J* = 6.6 Hz, 1H), 1.97 – 1.88 (m, 2H), 1.84 – 1.70 (m, 2H), 1.55 – 1.27 (m, 18H).
^{13}C NMR (101 MHz, $CDCl_3$) δ 178.62, 151.59, 135.95, 130.41, 126.51, 123.29, 121.11, 40.16, 30.90, 23.71, 23.69, 23.62, 23.36, 22.60.
HRMS (ESI+) *m/z* Calcd for $C_{19}H_{27}ClNS^+$: 336.1547 [*M*+H]$^+$; found 336.1555.

2-cyclohexyl-4-methylquinoline (76)

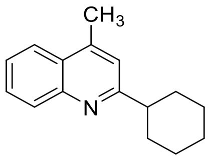

Prepared from 4-methylquinoline (28.6 mg, 0.2 mmol, 1.0 equiv) and cyclohexane (0.2 mL) following **general procedure F**. The product was obtained as colorless oil (20 mg, 44% yield) after silica gel column chromatography using petroleum ether/EtOAc (20:1 to 10:1).
^{1}H NMR (400 MHz, DMSO) δ 8.02 (d, *J* = 8.2 Hz, 1H), 7.92 (dd, *J* = 8.4, 1.2 Hz, 1H), 7.69 (ddd, *J* = 8.3, 6.8, 1.4 Hz, 1H), 7.54 (ddd, *J* = 8.2, 6.8, 1.3 Hz, 1H), 7.31 (s, 1H), 2.79 (tt, *J* = 11.9, 3.4 Hz,

1H), 2.65 (s, 3H), 1.94 – 1.79 (m, 4H), 1.76 – 1.69 (m, 1H), 1.61 (qd, *J* = 12.4, 3.2 Hz, 2H), 1.40 (qt, *J* = 12.4, 3.2 Hz, 2H), 1.27 (qt, *J* = 12.5, 3.2 Hz, 1H).
^{13}C NMR (101 MHz, DMSO) δ 166.19, 147.45, 144.70, 129.48, 129.42, 127.01, 125.95, 124.42, 121.16, 46.77, 32.61, 26.51, 26.13, 18.72.
HRMS (ESI+) *m/z* Calcd for $C_{16}H_{20}N^+$: 226.1590 [*M*+H]$^+$; found: 226.1591.

4-(1,2-dimethoxyethyl)-2-phenylquinoline (77)

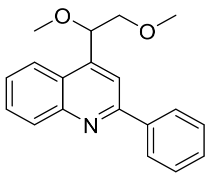

Prepared from 2-phenylquinoline (41.0 mg, 0.2 mmol, 1.0 equiv) and 1,2-dimethoxyethane (0.2 mL) following **general procedure F**. The product was obtained as colorless oil (23 mg, 40% yield) after silica gel column chromatography using petroleum ether/EtOAc (40:1 to 30:1).
^{1}H NMR (600 MHz, $CDCl_3$) δ 8.45 – 8.18 (m 3H), 8.12 (d, *J* = 8.4 Hz, 1H), 8.03 (s, 1H), 7.77 (t, *J* = 7.5 Hz, 1H), 7.57 (dt, *J* = 19.4, 7.6 Hz, 3H), 7.49 (t, *J* = 7.3 Hz, 1H), 5.22 (dd, *J* = 7.7, 3.1 Hz, 1H), 3.78 – 3.66 (m, 2H), 3.45 (s, 3H), 3.44 (s, 3H).
^{13}C NMR (151 MHz, $CDCl_3$) δ 157.16, 148.62, 144.87, 139.50, 130.75, 129.46, 129.43, 128.84, 127.61, 126.47, 125.38, 122.63, 116.67, 80.16, 76.50, 59.41, 57.77.
HRMS (ESI+) *m/z* Calcd for $C_{19}H_{20}NO_2^+$: 294.1489 [*M*+H]$^+$; found: 294.1490.

4-((2-methoxyethoxy)methyl)-2-phenylquinoline (78)

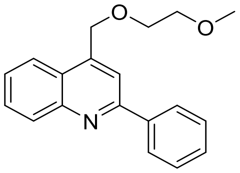

Prepared from 2-phenylquinoline (41.0 mg, 0.2 mmol, 1.0 equiv) and 1,2-dimethoxyethane (0.2 mL) following **general procedure F**. The product was obtained as colorless oil (13 mg, 22% yield) after silica gel column chromatography using petroleum ether/EtOAc (30:1 to 20:1).
^{1}H NMR (600 MHz, $CDCl_3$) δ 8.24 – 8.17 (m, 3H), 8.05 – 7.96 (m, 2H), 7.73 (ddd, *J* = 8.4, 6.9, 1.3 Hz, 1H), 7.54 (q, *J* = 8.3, 7.8 Hz, 3H), 7.49 – 7.43 (m, 1H), 5.12 (s, 2H), 3.80 (dd, *J* = 5.6, 3.6 Hz, 2H), 3.66 (dd, *J* = 5.7, 3.6 Hz, 2H), 3.44 (s, 3H).
^{13}C NMR (151 MHz, $CDCl_3$) δ 157.17, 148.25, 144.11, 139.63, 130.35, 129.42, 129.35, 128.78, 127.62, 126.34, 125.27, 123.02, 117.40, 72.05, 70.31, 70.22, 59.15.
HRMS (ESI+) *m/z* Calcd for $C_{19}H_{20}NO_2^+$: 294.1489 [*M*+H]$^+$; found: 294.1492.

4-(*tert*-butoxymethyl)-2-phenylquinoline (79)

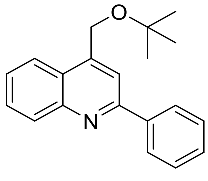

Prepared from 2-phenylquinoline (41.0 mg, 0.2 mmol, 1.0 equiv) and 2-methoxy-2-methylpropane (0.2 mL) following **general procedure F**. The product was obtained as colorless oil (18 mg, 31% yield) after silica gel column chromatography using petroleum ether/EtOAc (40:1 to 30:1).

^{1}H NMR (600 MHz, $CDCl_3$) δ 8.29 – 8.16 (m, 3H), 8.05 (d, *J* = 1.3 Hz, 1H), 7.98 (d, *J* = 8.3 Hz, 1H), 7.76 – 7.68 (m, 1H), 7.58 – 7.51 (m, 3H), 7.50 – 7.44 (m, 1H), 5.00 (s, 2H), 1.41 (s, 9H).

^{13}C NMR (151 MHz, $CDCl_3$) δ 157.28, 147.85, 144.54, 139.66, 130.24, 129.34, 128.78, 127.72, 126.21, 125.26, 122.76, 117.16, 74.27, 60.89, 27.69.

HRMS (ESI+) *m/z* Calcd for $C_{20}H_{22}NO^+$: 292.1696 [*M*+H]$^+$; found: 292.1697.

4-(1,4-dioxan-2-yl)-2-phenylquinoline (80)

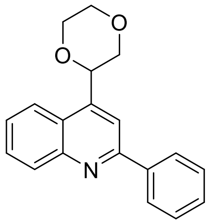

Prepared from 2-phenylquinoline (41.0 mg, 0.2 mmol, 1.0 equiv) and 1,4-dioxane (0.2 mL) following **general procedure F**. The product was obtained as colorless oil (44 mg, 75% yield) after silica gel column chromatography using petroleum ether/EtOAc (15:1 to 10:1).

^{1}H NMR (400 MHz, $CDCl_3$) δ 8.29 – 8.18 (m, 3H), 8.11 (s, 1H), 8.00 (d, *J* = 8.2 Hz, 1H), 7.73 (ddd, *J* = 8.4, 6.8, 1.4 Hz, 1H), 7.58 – 7.51 (m, 3H), 7.50 – 7.44 (m, 1H), 5.42 (dd, *J* = 9.9, 2.3 Hz, 1H), 4.18 (dd, *J* = 11.9, 2.5 Hz, 1H), 4.13 – 4.03 (m, 2H), 3.93 – 3.79 (m, 2H), 3.52 (dd, *J* = 11.8, 9.9 Hz, 1H).

^{13}C NMR (101 MHz, $CDCl_3$) δ 157.33, 148.26, 144.21, 139.58, 130.77, 129.44, 129.40, 128.83, 127.65, 126.53, 124.22, 122.35, 116.18, 74.43, 72.13, 67.38, 66.66.

HRMS (ESI+) *m/z* Calcd for $C_{19}H_{18}NO_2^+$: 292.1332 [*M*+H]$^+$; found 292.1341.

methyl 2-(2-phenylquinolin-4-yl)tetrahydro-2*H*-pyran-4-carboxylate (81)

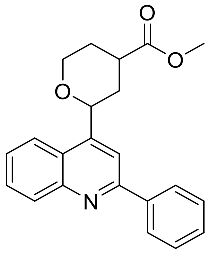

Prepared from 2-phenylquinoline (41.0 mg, 0.2 mmol, 1.0 equiv) and methyl tetrahydro-2*H*-pyran-4-carboxylate (0.2 mL) following **general procedure F**. The product was obtained as colorless oil (29 mg, 42% yield) after silica gel column chromatography using petroleum ether/EtOAc (10:1 to 8:1).

^{1}H NMR (600 MHz, $CDCl_3$) δ 8.24 – 8.19 (m, 3H), 8.15 (d, *J* = 8.2 Hz, 1H), 8.07 (s, 1H), 7.71 (ddd, *J* = 8.3, 6.8, 1.3 Hz, 1H), 7.57 (ddd, *J* = 8.2, 6.8, 1.3 Hz, 1H), 7.52 (dd, *J* = 8.2, 6.8 Hz, 2H), 7.47 – 7.43 (m, 1H), 5.39 (dd, *J* = 11.5, 2.0 Hz, 1H), 4.22 – 4.16 (m, 1H), 3.88 (s, 3H), 3.84 (td, *J* = 12.4, 2.5 Hz, 1H), 3.01 (dp, *J* = 5.3, 2.4 Hz, 1H), 2.61 (dq, *J* = 14.1, 2.2 Hz, 1H), 2.18 (dp, *J* = 13.9, 2.2 Hz, 1H), 2.10 (ddt, *J* = 14.0, 12.7, 5.2 Hz, 1H), 1.80 – 1.72 (m, 1H).

^{13}C NMR (151 MHz, $CDCl_3$) δ 174.95, 157.30, 149.09, 148.19, 139.74, 130.43, 129.27, 129.23, 128.72, 127.67, 126.35, 124.18, 122.93, 114.93, 72.84, 65.85, 52.08, 37.50, 34.56, 27.38.
HRMS (ESI+) *m/z* Calcd for $C_{22}H_{22}NO_3^+$: 348.1594 [*M*+H]$^+$; found 348.1599.

2-(2-phenylquinolin-4-yl)ethan-1-ol (82)

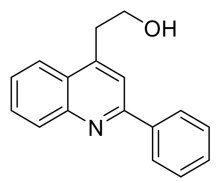

Prepared from 2-phenylquinoline (41.0 mg, 0.2 mmol, 1.0 equiv) and 1,3-dioxolane (0.2 mL) following **general procedure F**. The product was obtained as white solid (18 mg, 36% yield) after silica gel column chromatography using petroleum ether/EtOAc (5:1 to 4:1).
^{1}H NMR (400 MHz, $CDCl_3$) δ 8.20 (d, *J* = 8.4 Hz, 1H), 8.06 – 8.01 (m, 2H), 7.96 (dd, *J* = 8.4, 1.5 Hz, 1H), 7.74 – 7.67 (m, 2H), 7.53 – 7.40 (m, 4H), 4.02 (t, *J* = 6.4 Hz, 2H), 3.35 (t, *J* = 6.4 Hz, 2H), 2.46 (br, 1H).
^{13}C NMR (101 MHz, $CDCl_3$) δ 156.71, 148.04, 145.80, 139.06, 130.09, 129.59, 129.42, 128.80, 127.60, 126.54, 126.38, 123.34, 119.94, 62.21, 35.83.
HRMS (ESI+) *m/z* Calcd for $C_{17}H_{16}NO^+$: 250.1226 [*M*+H]$^+$; found 250.1222.

4-(2-phenylquinolin-4-yl)butan-1-ol (83)

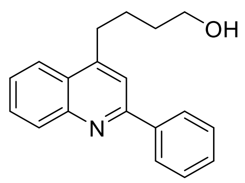

Prepared from 2-phenylquinoline (41.0 mg, 0.2 mmol, 1.0 equiv) and tetrahydrofuran (0.2 mL) following **general procedure F**. The product was obtained as yellow oil (37 mg, 67% yield) after silica gel column chromatography using petroleum ether/EtOAc (5:1 to 4:1).
^{1}H NMR (400 MHz, $CDCl_3$) δ 8.23 (d, *J* = 8.4 Hz, 1H), 8.14 – 8.09 (m, 2H), 8.01 (dd, *J* = 8.4, 1.3 Hz, 1H), 7.71 (ddd, *J* = 8.4, 6.8, 1.4 Hz, 1H), 7.68 (s, 1H), 7.56 – 7.42 (m, 4H), 3.70 (t, *J* = 6.3 Hz, 2H), 3.12 (t, *J* = 7.8 Hz, 2H), 2.67 (br, 1H), 1.91 – 1.81 (m, 2H), 1.75 – 1.65 (m, 2H).
^{13}C NMR (101 MHz, $CDCl_3$) δ 156.98, 149.57, 147.87, 139.31, 129.95, 129.59, 129.45, 128.84, 127.71, 126.47, 126.26, 123.40, 118.90, 62.43, 32.62, 32.25, 26.36.
HRMS (ESI+) *m/z* Calcd for $C_{19}H_{20}NO^+$: 278.1539 [*M*+H]$^+$; found 278.1544.

5-(2-phenylquinolin-4-yl)pentan-2-ol (84)

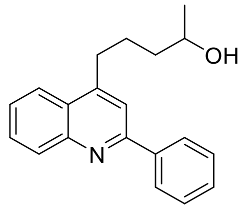

Prepared from 2-phenylquinoline (41.0 mg, 0.2 mmol, 1.0 equiv) and 2-methyltetrahydrofuran (0.2 mL) following **general procedure F**. The product was obtained as white solid (38 mg, 65% yield)

after silica gel column chromatography using petroleum ether/EtOAc (5:1 to 4:1).

^{1}H NMR (400 MHz, $CDCl_3$) δ 8.21 (dd, *J* = 8.5, 1.3 Hz, 1H), 8.16 – 8.11 (m, 2H), 8.01 (dd, *J* = 8.4, 1.4 Hz, 1H), 7.73 – 7.66 (m, 2H), 7.52 (ddd, *J* = 8.0, 6.5, 1.5 Hz, 3H), 7.49 – 7.42 (m, 1H), 3.83 (h, *J* = 6.1 Hz, 1H), 3.17 – 3.05 (m, 2H), 1.99 – 1.79 (m, 2H), 1.74 (br, 1H), 1.66 – 1.53 (m, 2H), 1.19 (d, *J* = 6.2 Hz, 3H).

^{13}C NMR (101 MHz, $CDCl_3$) δ 157.08, 148.99, 148.38, 139.79, 130.40, 129.31, 129.26, 128.81, 127.62, 126.48, 126.09, 123.36, 118.75, 67.76, 39.10, 32.42, 26.33, 23.74.

HRMS (ESI+) *m/z* Calcd for $C_{20}H_{22}NO^+$: 292.1696 [*M*+H]$^+$; found 292.1698.

<u>5-(2-phenylquinolin-4-yl)pentan-1-ol (85)</u>

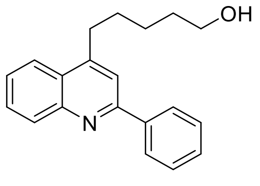

Prepared from 2-phenylquinoline (41.0 mg, 0.2 mmol, 1.0 equiv) and tetrahydro-2*H*-pyran (0.2 mL) following **general procedure F**. The product was obtained as colorless oil (26 mg, 44% yield) after silica gel column chromatography using petroleum ether/EtOAc (5:1 to 4:1).

^{1}H NMR (400 MHz, $CDCl_3$) δ 8.21 (d, *J* = 8.4 Hz, 1H), 8.17 – 8.13 (m, 2H), 8.01 (dd, *J* = 8.4, 1.3 Hz, 1H), 7.73 – 7.68 (m, 2H), 7.56 – 7.50 (m, 3H), 7.49 – 7.43 (m, 1H), 3.63 (t, *J* = 6.4 Hz, 2H), 3.17 – 3.01 (m, 2H), 1.93 – 1.75 (m, 3H), 1.68 – 1.58 (m, 2H), 1.56 – 1.48 (m, 2H).

^{13}C NMR (101 MHz, $CDCl_3$) δ 157.11, 149.20, 148.37, 139.79, 130.37, 129.33, 129.27, 128.82, 127.64, 126.50, 126.08, 123.38, 118.83, 62.64, 32.54, 30.02, 25.94.

HRMS (ESI+) *m/z* Calcd for $C_{20}H_{22}NO^+$: 292.1696 [*M*+H]$^+$; found 292.1701.

<u>1-(2-phenylquinolin-4-yl)ethane-1,2-diol (86)</u>

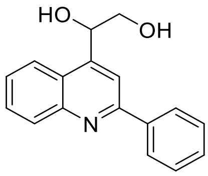

Prepared from 2-phenylquinoline (41.0 mg, 0.2 mmol, 1.0 equiv) and ethane-1,2-diol (0.2 mL) following **general procedure F**. The product was obtained as colorless oil (24 mg, 45% yield) after silica gel column chromatography using DCM/MeOH (40:1 to 30:1).

^{1}H NMR (600 MHz, DMSO-*d*$_6$) δ 8.23 (ddd, *J* = 15.7, 8.5, 1.4 Hz, 3H), 8.18 (s, 1H), 8.10 (dd, *J* = 8.5, 1.2 Hz, 1H), 7.76 (ddd, *J* = 8.3, 6.8, 1.3 Hz, 1H), 7.64 – 7.55 (m, 3H), 7.53 – 7.49 (m, 1H), 5.73 (d, *J* = 4.6 Hz, 1H), 5.42 (dt, *J* = 6.5, 4.5 Hz, 1H), 4.91 (t, *J* = 6.0 Hz, 1H), 3.75 (ddd, *J* = 10.7, 5.9, 4.4 Hz, 1H), 3.64 (dt, *J* = 11.3, 6.2 Hz, 1H).

^{13}C NMR (151 MHz, DMSO-*d*$_6$) δ 156.11, 150.32, 148.18, 139.44, 130.31, 129.96, 129.78, 129.33, 127.56, 126.59, 125.41, 124.25, 116.41, 70.99, 67.17.

HRMS (ESI+) *m/z* Calcd for $C_{17}H_{16}NO_2^+$: 266.1176 [*M*+H]$^+$; found 266.1174.

<u>N-methyl-*N*-((2-phenylquinolin-4-yl)methyl)formamide (87)</u>

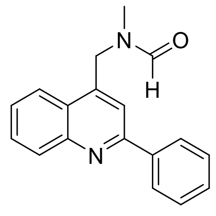

Prepared from 2-phenylquinoline (41.0 mg, 0.2 mmol, 1.0 equiv) and *N*,*N*-dimethylformamide (0.2 mL) following **general procedure F**. The product was obtained as colorless oil (18 mg, 33% yield) after silica gel column chromatography using petroleum ether/EtOAc (2:1 to 1:1).

^{1}H NMR (600 MHz, $CDCl_3$) δ 8.44 – 8.21 (m, 2H), 8.19 – 8.11 (m, 2H), 7.99 (dd, *J* = 103.2, 8.4 Hz, 1H), 7.80 – 7.63 (m, 2H), 7.61 – 7.45 (m, 4H), 5.07 – 4.95 (m, 2H), 2.98 – 2.88 (m, 2H).

^{13}C NMR (151 MHz, $CDCl_3$) δ 163.35, 162.57, 157.24, 156.96, 148.48, 141.92, 139.22, 130.86, 130.44, 129.94, 129.72, 129.58, 128.95, 128.89, 127.60, 127.55, 126.94, 125.60, 125.07, 123.19, 121.79, 118.92, 116.71, 50.11, 45.18, 34.36, 30.52.

HRMS (ESI+) *m/z* Calcd for $C_{18}H_{17}N_2O^+$: 277.1335 [*M*+H]$^+$; found 277.1338.

<u>3-(2-phenylquinolin-4-yl)cyclohexan-1-one (88)</u>

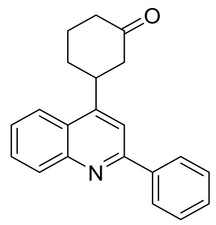

Prepared from 2-phenylquinoline (41.0 mg, 0.2 mmol, 1.0 equiv) and cyclohexanone (0.2 mL) following **general procedure F**. The product was obtained as colorless oil (34 mg, 57% yield) after silica gel column chromatography using petroleum ether/EtOAc (7:1 to 5:1).

^{1}H NMR (600 MHz, $CDCl_3$) δ 8.24 (d, *J* = 8.4 Hz, 1H), 8.17 – 8.11 (m, 2H), 8.02 (dd, *J* = 8.5, 1.3 Hz, 1H), 7.77 (s, 1H), 7.73 (ddd, *J* = 8.3, 6.8, 1.3 Hz, 1H), 7.59 – 7.52 (m, 3H), 7.50 – 7.46 (m, 1H), 3.91 (tt, *J* = 11.5, 3.8 Hz, 1H), 2.81 (ddt, *J* = 14.0, 4.1, 2.0 Hz, 1H), 2.72 (ddd, *J* = 13.8, 12.1, 1.0 Hz, 1H), 2.62 – 2.56 (m, 1H), 2.50 (dddd, *J* = 14.5, 12.3, 6.2, 1.1 Hz, 1H), 2.30 (ddq, *J* = 12.9, 3.7, 1.8 Hz, 1H), 2.24 (ddq, *J* = 13.8, 7.4, 3.6 Hz, 1H), 2.09 (dtd, *J* = 12.9, 11.4, 3.3 Hz, 1H), 1.96 (dtdd, *J* = 13.4, 12.1, 4.7, 3.4 Hz, 1H).

^{13}C NMR (151 MHz, $CDCl_3$) δ 210.00, 157.24, 150.23, 148.60, 139.68, 130.81, 129.50, 129.43, 128.88, 127.59, 126.53, 125.23, 122.36, 115.45, 47.75, 41.33, 39.11, 31.75, 25.40.

HRMS (ESI+) *m/z* Calcd for $C_{21}H_{20}NO^+$: 302.1539 [*M*+H]$^+$; found 302.1541.

<u>*N*-(6,6-dicyano-4-methyl-5-phenylhexyl)-4-methoxybenzenesulfonamide (89)</u>

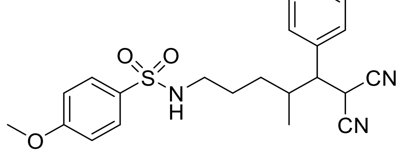

Prepared from 4-methoxy-*N*-pentylbenzenesulfonamide (51.4 mg, 0.2 mmol, 1.0 equiv) and 2-benzylidenemalononitrile (92.4 mg, 0.6 mmol, 3.0 equiv) following **general procedure G**. The product was obtained as yellow oil (37 mg, 45% yield) after silica gel column chromatography using

petroleum ether/EtOAc (4:1).

^{1}H NMR (400 MHz, $CDCl_3$) δ 7.83 – 7.68 (m, 2H), 7.44 – 7.39 (m, 3H), 7.31 – 7.27 (m, 2H), 7.01 – 6.96 (m, 2H), 4.56 (t, *J* = 5.4 Hz, 1H), 4.21 (d, *J* = 5.8 Hz, 1H), 3.89 (s, 3H), 2.90 (dd, *J* = 9.6, 5.8 Hz, 1H), 2.79 (hept, *J* = 6.1 Hz, 2H), 2.24 – 2.13 (m, 1H), 1.58 – 1.44 (m, 1H), 1.40 – 1.21 (m, 2H), 1.08 (d, *J* = 6.6 Hz, 3H), 1.00 – 0.91 (m, 1H).

^{13}C NMR (101 MHz, $CDCl_3$) δ 162.92, 136.22, 131.32, 129.25, 129.16, 128.94, 128.26, 114.31, 112.15, 111.85, 55.66, 52.04, 42.95, 34.50, 30.21, 27.72, 26.58, 17.42.

HRMS (ESI+) *m/z* Calcd for $C_{22}H_{26}N_3O_3S^+$: 412.1689 [*M*+H]$^+$; found 412.1692.

ethyl 2-cyano-7-((4-methoxyphenyl)sulfonamido)-4-methyl-3-phenylheptanoate (90)

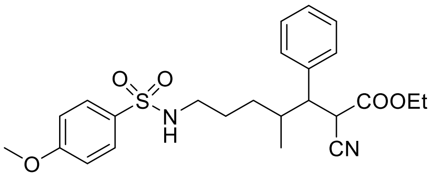

Prepared from 4-methoxy-*N*-pentylbenzenesulfonamide (51.4 mg, 0.2 mmol, 1.0 equiv) and ethyl (*E*)-2-cyano-3-phenylacrylate (120.6 mg, 0.6 mmol, 3.0 equiv) following **general procedure G**. The product was obtained as yellow oil (38 mg, 41% yield) after silica gel column chromatography using petroleum ether/EtOAc (3:1).

^{1}H NMR (400 MHz, $CDCl_3$) δ 7.87 – 7.79 (m, 1H), 7.79 – 7.70 (m, 1H), 7.36 – 7.10 (m, 5H), 7.06 – 6.94 (m, 2H), 4.95 – 4.75 (m, 0.5H), 4.70 – 4.45 (m, 0.5H), 4.17 – 3.80 (m, 6H), 3.30 – 2.87 (m, 2H), 2.87 – 2.65 (m, 1H), 2.24 – 2.02 (m, 1H), 1.71 – 1.43 (m, 2H), 1.41 – 1.11 (m, 2H), 1.10 – 0.66 (m, 6H).

^{13}C NMR (101 MHz, $CDCl_3$) δ 165.58, 165.54, 165.36, 162.89, 162.85, 162.83, 138.61, 137.77, 137.73, 137.01, 131.54, 131.50, 131.44, 129.24, 129.20, 129.17, 129.14, 128.73, 128.68, 128.58, 128.56, 128.47, 128.43, 128.24, 128.10, 127.92, 127.83, 127.79, 116.12, 116.06, 115.75, 115.47, 114.32, 114.28, 114.26, 114.24, 62.80, 62.74, 62.54, 55.64, 55.63, 51.49, 51.27, 51.03, 50.49, 43.25, 43.12, 43.07, 42.43, 42.36, 41.90, 41.75, 35.02, 34.80, 34.75, 31.67, 30.94, 30.80, 29.92, 27.15, 26.89, 26.50, 17.48, 17.27, 17.03, 15.41, 13.74, 13.72, 13.69, 13.67.

HRMS (ESI+) *m/z* Calcd for $C_{24}H_{31}N_2O_5S^+$: 459.1948 [*M*+H]$^+$; found 459.1949.

diethyl 2-(6-((4-methoxyphenyl)sulfonamido)-3-methylhexan-2-yl)malonate (91)

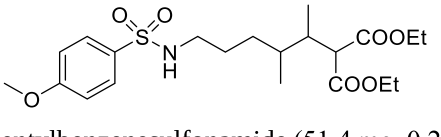

Prepared from 4-methoxy-*N*-pentylbenzenesulfonamide (51.4 mg, 0.2 mmol, 1.0 equiv) and diethyl 2-ethylidenemalonate (111.7 mg, 0.6 mmol, 3.0 equiv) following **general procedure G**. The product was obtained as colorless oil (42 mg, 47% yield) after silica gel column chromatography using petroleum ether/EtOAc (3:1).

^{1}H NMR (400 MHz, $CDCl_3$) δ 7.79 (dq, *J* = 8.2, 3.0 Hz, 2H), 7.01 – 6.93 (m, 2H), 4.72 (t, *J* = 6.2 Hz, 0.52H), 4.60 (t, *J* = 6.3 Hz, 0.48H), 4.20 – 4.12 (m, 4H), 3.86 (s, 3H), 3.26 (dd, *J* = 13.5, 10.1 Hz, 1H), 2.96 – 2.79 (m, 2H), 2.31 – 2.14 (m, 1H), 1.56 – 1.13 (m, 11H), 0.88 – 0.70 (m, 6H).

^{13}C NMR (101 MHz, $CDCl_3$) δ 169.19, 168.89, 168.83, 168.78, 162.80, 162.79, 131.64, 131.58, 129.20, 114.22, 114.20, 61.39, 61.28, 61.22, 56.63, 56.10, 55.61, 43.28, 43.11, 38.47, 36.29, 34.32,

33.77, 32.14, 27.61, 27.49, 27.36, 17.85, 14.10, 13.67, 12.36, 11.03.
HRMS (ESI+) *m/z* Calcd for $C_{21}H_{34}NO_7S^+$: 444.2050 [*M*+H]$^+$; found 444.2056.

di-*tert*-butyl 1-(5-((4-methoxyphenyl)sulfonamido)pentan-2-yl)hydrazine-1,2-dicarboxylate (92)

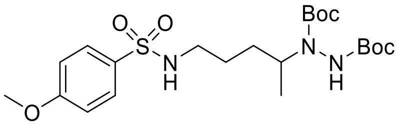

Prepared from 4-methoxy-*N*-pentylbenzenesulfonamide (51.4 mg, 0.2 mmol, 1.0 equiv) and di-*tert*-butyl azodicarboxylate (138.1 mg, 0.6 mmol, 3.0 equiv) following **general procedure G**. The product was obtained as yellow oil (51 mg, 52% yield) after silica gel column chromatography using petroleum ether/EtOAc (3:1).
^{1}H NMR (400 MHz, $CDCl_3$) δ 7.83 – 7.70 (m, 2H), 6.94 (d, *J* = 8.5 Hz, 2H), 6.10 (br, 1H), 5.28 – 4.85 (m, 1H), 4.25 – 2.96 (m, 1H), 3.84 (s, 3H), 2.90 (q, *J* = 5.8 Hz, 2H), 1.68 – 1.36 (m, 22H), 1.04 (d, *J* = 6.7 Hz, 3H).
^{13}C NMR (101 MHz, $CDCl_3$) δ 162.64, 156.53, 155.07, 131.71, 129.18, 114.10, 81.45, 81.09, 55.58, 51.91, 42.77, 31.08, 28.25, 28.23, 25.88, 18.35.
HRMS (ESI+) *m/z* Calcd for $C_{22}H_{38}N_3O_7S^+$: 488.2425 [*M*+H]$^+$; found 488.2428.

***N*-(4-azidopentyl)-4-methoxybenzenesulfonamide (93)**

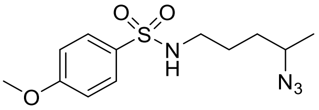

Prepared from 4-methoxy-*N*-pentylbenzenesulfonamide (51.4 mg, 0.2 mmol, 1.0 equiv) and 4-methylbenzenesulfonyl azide (118.2 mg, 0.6 mmol, 3.0 equiv) following **general procedure G**. The product was obtained as colorless oil (18 mg, 30% yield) after silica gel column chromatography using petroleum ether/EtOAc (4:1).
^{1}H NMR (400 MHz, $CDCl_3$) δ 7.85 – 7.78 (m, 2H), 7.04 – 6.97 (m, 2H), 4.73 (t, *J* = 6.2 Hz, 1H), 3.89 (s, 3H), 3.41 (h, *J* = 6.5 Hz, 1H), 2.95 (q, *J* = 6.5 Hz, 2H), 1.65 – 1.40 (m, 4H), 1.24 (d, *J* = 6.5 Hz, 3H).
^{13}C NMR (101 MHz, $CDCl_3$) δ 162.93, 131.49, 129.18, 114.29, 57.36, 55.61, 42.78, 33.08, 26.16, 19.34.
HRMS (ESI+) *m/z* Calcd for $C_{12}H_{19}N_4O_3S^+$: 299.1172 [*M*+H]$^+$; found 299.1165.

***N*-(4-cyanopentyl)-4-methoxybenzenesulfonamide (94)**

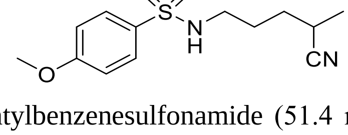

Prepared from 4-methoxy-*N*-pentylbenzenesulfonamide (51.4 mg, 0.2 mmol, 1.0 equiv) and 4-methylbenzenesulfonyl cyanide (108.6 mg, 0.6 mmol, 3.0 equiv) following **general procedure G**. The product was obtained as yellow oil (29 mg, 51% yield) after silica gel column chromatography using petroleum ether/EtOAc (4:1 to 3:1).
^{1}H NMR (400 MHz, $CDCl_3$) δ 7.81 – 7.75 (m, 2H), 7.00 – 6.94 (m, 2H), 4.99 (t, *J* = 6.3 Hz, 1H),

3.86 (s, 3H), 2.93 (q, J = 6.4 Hz, 2H), 2.57 (h, J = 7.0 Hz, 1H), 1.73 – 1.54 (m, 4H), 1.26 (d, J = 7.1 Hz, 3H).

^{13}C NMR (101 MHz, $CDCl_3$) δ 162.96, 131.22, 129.17, 122.65, 114.36, 55.67, 42.37, 30.87, 27.01, 25.10, 17.95.

HRMS (ESI+) *m/z* Calcd for $C_{13}H_{19}N_2O_3S^+$: 283.1111 [M+H]$^+$; found 283.1115.

***N*-(4-bromopentyl)-4-methoxybenzenesulfonamide (95)**

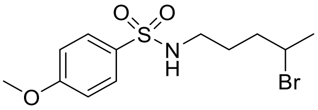

Prepared from 4-methoxy-*N*-pentylbenzenesulfonamide (51.4 mg, 0.2 mmol, 1.0 equiv) and 1-bromopyrrolidine-2,5-dione (70.8 mg, 0.4 mmol, 2.0 equiv) following **general procedure G**. The inseparable mixture of mono- and dibrominated products were obtained as yellow oil (momo:di = 9:1, 36 mg, 53% yield) after silica gel column chromatography using petroleum ether/EtOAc (8:1 to 6:1).

^{1}H NMR (400 MHz, $CDCl_3$) δ 7.82 – 7.77 (m, 2H+0.22H), 7.00 – 6.95 (m, 2H+0.22H), 4.88 (br, 0.11H), 4.80 (br, 1H), 4.09 – 4.00 (m, 1H), 3.86 (s, 3H+0.33H), 3.02 (q, J = 6.3 Hz, 0.22H), 2.94 (t, J = 7.6 Hz, 2H), 2.48 (s, 0.33H), 2.34 – 2.28 (m, 0.22H), 1.92 – 1.83 (m, 0.22H), 1.82 – 1.54 (m, 7H).

^{13}C NMR (101 MHz, $CDCl_3$) δ 162.97, 162.91, 131.32, 131.27, 129.23, 129.21, 114.37, 114.32, 67.42, 55.67, 50.80, 49.61, 42.44, 42.20, 41.41, 37.78, 28.38, 27.81, 26.48.

HRMS (ESI+) *m/z* Calcd for $C_{12}H_{19}BrNO_3S^+$: 336.0264 [M+H]$^+$; found 336.0257.

HRMS (ESI+) *m/z* Calcd for $C_{12}H_{18}Br_2NO_3S^+$: 413.9369 [M+H]$^+$; found 413.9364.

***N*-(4-fluoropentyl)-4-methoxybenzenesulfonamide (96)**

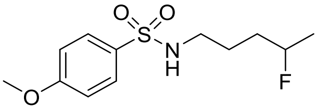

Prepared from 4-methoxy-*N*-pentylbenzenesulfonamide (51.4 mg, 0.2 mmol, 1.0 equiv) and Selectfluor (141.6 mg, 0.4 mmol, 2.0 equiv) following **general procedure G**. The inseparable mixture of mono- and difluorinated products were obtained as colorless oil (momo:di = 1:1, 38 mg, 66% yield) after silica gel column chromatography using petroleum ether/EtOAc (5:1 to 4:1).

^{1}H NMR (400 MHz, $CDCl_3$) δ 7.82 – 7.75 (m, 2H+2H), 7.01 – 6.93 (m, 2H+2H), 5.01 (t, J = 6.4 Hz, 1H), 4.93 (t, J = 6.4 Hz, 1H), 4.69 – 4.46 (m, 1H), 3.85 (s, 3H+3H), 2.98 – 2.89 (m, 2H+2H), 1.91 – 1.76 (m, 2H), 1.70 – 1.47 (m, 7H+2H), 1.25 (dd, J = 24.0, 6.2 Hz, 3H).

^{19}F NMR (376 MHz, $CDCl_3$) δ -91.06, -173.24.

^{13}C NMR (101 MHz, $CDCl_3$) δ 162.91, 162.87, 131.38, 131.32, 129.17, 123.93 (t, $J_{F\text{-}C}$ = 238.1 Hz), 114.31, 114.28, 90.45 (d, $J_{F\text{-}C}$ = 165.1 Hz), 55.64, 42.87, 42.62, 34.86 (t, $J_{F\text{-}C}$ = 25.9 Hz), 33.72 (d, $J_{F\text{-}C}$ = 21.3 Hz), 25.28 (d, $J_{F\text{-}C}$ = 4.0 Hz), 23.41 (t, $J_{F\text{-}C}$ = 27.9 Hz), 22.92 (t, $J_{F\text{-}C}$ = 4.6 Hz), 20.94 (d, $J_{F\text{-}C}$ = 22.4 Hz).

HRMS (ESI+) *m/z* Calcd for $C_{12}H_{19}FNO_3S^+$: 276.1064 [M+H]$^+$; found 276.1069.

HRMS (ESI+) *m/z* Calcd for $C_{12}H_{18}F_2NO_3S^+$: 294.0970 [M+H]$^+$; found 294.0978.

4-methoxy-*N*-(4-((trifluoromethyl)thio)pentyl)benzenesulfonamide (97)

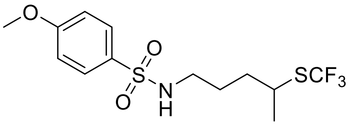

Prepared from 4-methoxy-*N*-pentylbenzenesulfonamide (51.4 mg, 0.2 mmol, 1.0 equiv) and 2-((trifluoromethyl)thio)isoindoline-1,3-dione (148.2 mg, 0.6 mmol, 2.0 equiv) following **general procedure G**. The product was obtained as yellow oil (25 mg, 35% yield) after silica gel column chromatography using petroleum ether/EtOAc (4:1 to 3:1).

^{1}H NMR (400 MHz, $CDCl_3$) δ 7.82 – 7.78 (m, 2H), 7.00 – 6.96 (m, 2H), 4.76 (t, *J* = 6.2 Hz, 1H), 3.86 (s, 3H), 3.22 (dq, *J* = 9.7, 5.2, 3.8 Hz, 1H), 2.94 (dt, *J* = 8.4, 5.1 Hz, 2H), 1.60 (p, *J* = 5.7, 4.9 Hz, 4H), 1.36 (d, *J* = 6.9 Hz, 3H).

^{13}C NMR (101 MHz, $CDCl_3$) δ 162.95, 131.37, 131.02 (q, $J_{F\text{-}C}$ = 306.3 Hz), 129.19, 114.31, 55.62, 42.66, 40.69, 33.68, 26.72, 22.35.

^{19}F NMR (376 MHz, $CDCl_3$) δ -39.13.

HRMS (ESI+) *m*/*z* Calcd for $C_{13}H_{19}F_3NO_3S_2^+$: 358.0753 [*M*+H]$^+$; found 358.0759.

4-methoxy-*N*-(pentyl-4-*d*)benzenesulfonamide (98)

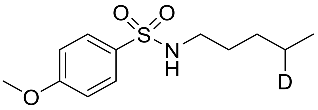

Prepared from 4-methoxy-*N*-pentylbenzenesulfonamide (51.4 mg, 0.2 mmol, 1.0 equiv) and D_2O (0.2 mL) following the **Procedures for preparation of specific compounds**. The product was obtained as colorless oil (41 mg, 80% yield) after silica gel column chromatography using petroleum ether/EtOAc (4:1).

^{1}H NMR (400 MHz, $CDCl_3$) δ 7.81 – 7.77 (m, 2H), 6.98 – 6.92 (m, 2H), 5.00 (t, *J* = 6.2 Hz, 1H), 3.83 (s, 3H), 2.86 (q, *J* = 6.8 Hz, 2H), 1.42 (tt, *J* = 8.0, 6.6 Hz, 2H), 1.24 – 1.14 (m, 3.15H), 0.84 – 0.76 (m, 3H).

^{13}C NMR (101 MHz, $CDCl_3$) δ 162.75, 131.53, 129.19, 114.19, 55.61, 43.16, 29.09 (t, $J_{D\text{-}C}$ = 2.5 Hz), 28.55 (t, $J_{D\text{-}C}$ = 10.6 Hz), 21.73 (t, $J_{D\text{-}C}$ = 19.2 Hz), 13.76 (t, $J_{D\text{-}C}$ = 11.1 Hz).

HRMS (ESI+) *m*/*z* Calcd for $C_{12}H_{19}DNO_3S^+$: 259.1221 [*M*+H]$^+$; found 259.1209.

4**-(1-((4-methoxyphenyl)sulfonyl)-2-methylpyrrolidin-2-yl)-2-phenylquinoline (99)**

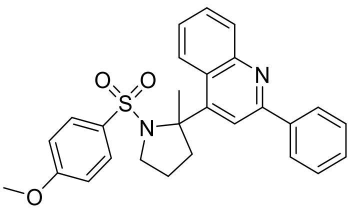

The product was obtained as white solid (27 mg, 29% yield) after silica gel column chromatography using petroleum ether/EtOAc (8:1 to 6:1).

^{1}H NMR (600 MHz, $CDCl_3$) δ 8.29 – 8.19 (m, 4H), 7.89 (d, *J* = 8.6 Hz, 1H), 7.62 (ddd, *J* = 8.2, 6.7, 1.3 Hz, 1H), 7.58 – 7.54 (m, 2H), 7.51 – 7.41 (m, 3H), 7.30 – 7.26 (m, 1H), 6.73 (d, *J* = 8.5 Hz, 2H), 3.84 – 3.72 (m, 5H), 2.84 (dt, *J* = 12.7, 7.4 Hz, 1H), 2.21 (s, 3H), 2.17 – 2.05 (m, 2H), 2.01 – 1.94 (m, 1H).

^{13}C NMR (151 MHz, $CDCl_3$) δ 162.50, 156.80, 150.61, 149.28, 139.50, 132.15, 131.05, 129.47, 129.13, 128.87, 128.52, 127.84, 125.42, 125.01, 124.35, 118.59, 113.66, 70.42, 55.53, 48.50, 42.74, 27.09, 22.96.

HRMS (ESI+) *m/z* Calcd for $C_{27}H_{27}N_2O_3S^+$: 459.1737 [*M*+H]$^+$; found 459.1746.

<u>*N*-allyl-4-methoxy-*N*-(4-(2-phenylquinolin-4-yl)pentyl)benzenesulfonamide (100)</u>

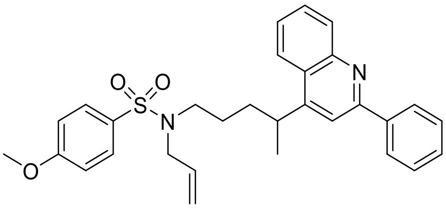

The product was obtained as white solid (83 mg, 83% yield) after silica gel column chromatography using petroleum ether/EtOAc (10:1 to 8:1).

^{1}H NMR (400 MHz, $CDCl_3$) δ 8.23 (dd, *J* = 8.6, 1.3 Hz, 1H), 8.18 – 8.14 (m, 2H), 8.08 (dd, *J* = 8.6, 1.3 Hz, 1H), 7.75 – 7.70 (m, 2H), 7.69 – 7.63 (m, 2H), 7.57 – 7.52 (m, 3H), 7.49 – 7.44 (m, 1H), 6.90 – 6.85 (m, 2H), 5.53 (ddt, *J* = 16.7, 10.1, 6.5 Hz, 1H), 5.04 – 4.95 (m, 2H), 3.80 (s, 3H), 3.71 – 3.59 (m, 3H), 3.10 (td, *J* = 7.1, 2.1 Hz, 2H), 1.93 – 1.72 (m, 2H), 1.53 (ddt, *J* = 16.9, 9.8, 6.9 Hz, 2H), 1.42 (d, *J* = 6.9 Hz, 3H).

^{13}C NMR (101 MHz, $CDCl_3$) δ 162.69, 157.16, 153.74, 148.57, 139.96, 133.18, 131.51, 130.66, 129.30, 129.28, 129.12, 128.86, 127.62, 126.17, 122.81, 118.70, 115.58, 114.17, 55.58, 50.63, 47.26, 34.00, 33.28, 26.02, 21.60.

HRMS (ESI+) *m/z* Calcd for $C_{30}H_{33}N_2O_3S^+$: 501.2206 [*M*+H]$^+$; found 501.2216.

<u>4-methoxy-*N*-pentyl-*N*-((2-phenylquinolin-4-yl)methyl)benzenesulfonamide (103)</u>

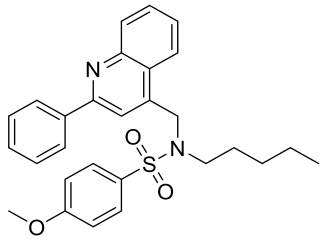

The product was obtained as yellow oil (30 mg, 32% yield) after silica gel column chromatography using petroleum ether/EtOAc (4:1 to 3:1).

^{1}H NMR (400 MHz, $CDCl_3$) δ 8.27 – 8.19 (m, 1H), 8.11 (d, *J* = 1.3 Hz, 1H), 8.10 – 8.05 (m, 2H), 7.86 – 7.81 (m, 2H), 7.80 – 7.76 (m, 1H), 7.74 (dt, *J* = 8.1, 1.4 Hz, 1H), 7.57 (ddd, *J* = 8.3, 6.9, 1.3 Hz, 1H), 7.54 – 7.45 (m, 3H), 7.02 – 6.97 (m, 2H), 4.87 (s, 2H), 3.85 (s, 3H), 3.25 – 3.13 (m, 2H), 1.41 – 1.29 (m, 2H), 1.10 – 1.02 (m, 4H), 0.69 (t, *J* = 6.8 Hz, 3H).

^{13}C NMR (101 MHz, $CDCl_3$) δ 163.02, 156.86, 148.96, 143.99, 139.18, 131.06, 130.44, 129.76, 129.55, 129.39, 128.84, 127.54, 126.72, 125.47, 122.76, 118.21, 114.42, 55.64, 49.42, 49.12, 28.82, 28.03, 22.10, 13.79.

HRMS (ESI+) *m/z* Calcd for $C_{28}H_{31}N_2O_3S^+$: 475.2050 [*M*+H]$^+$; found 475.2051.

8. NMR spectra for synthesized compounds

8.1 NMR spectra for substrates

4-methoxy-*N*-pentylbenzenesulfonamide (2a)

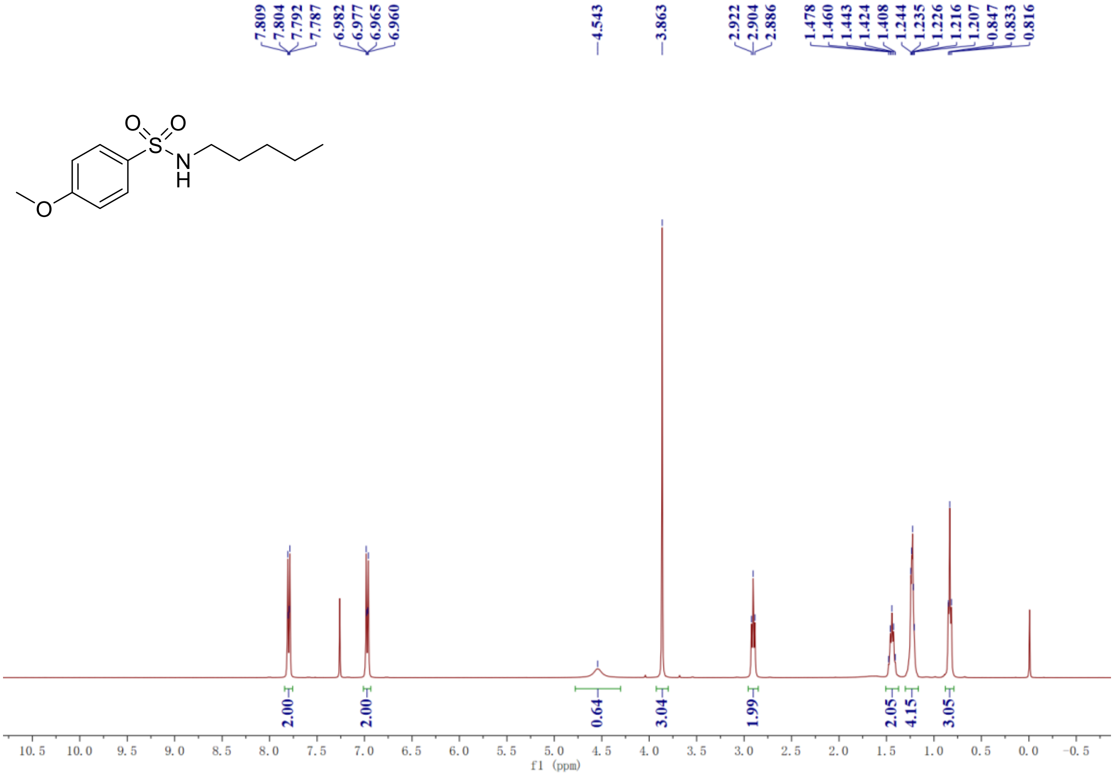

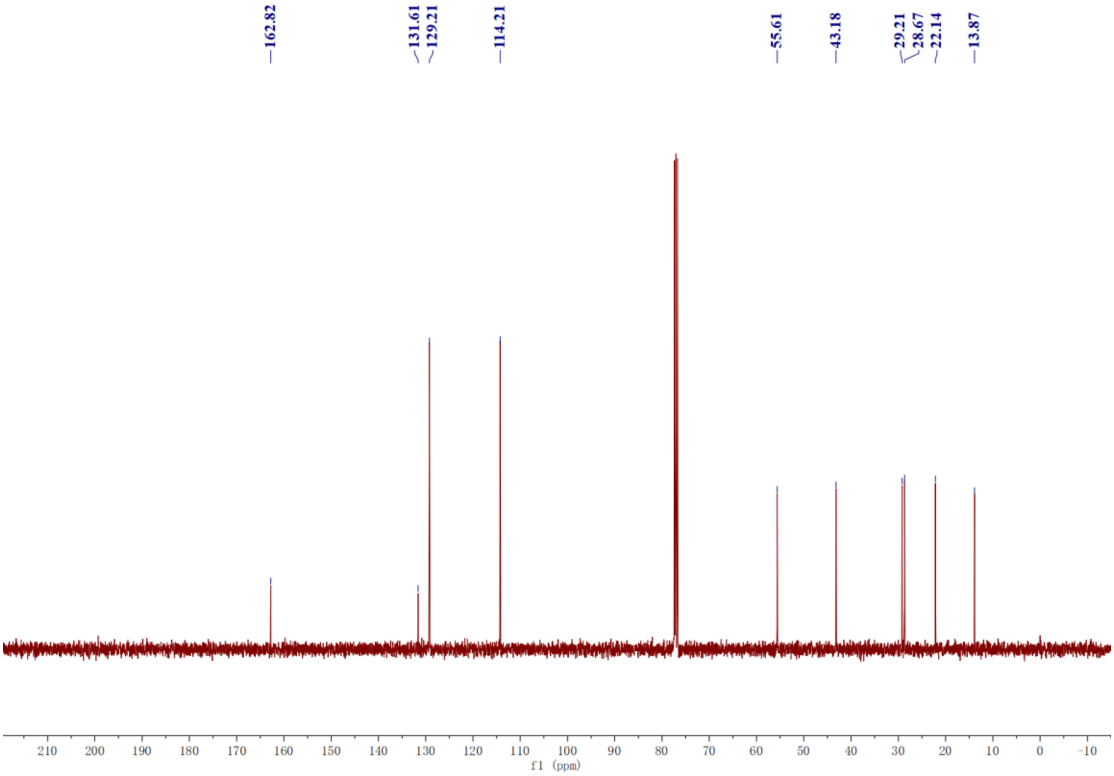

***N*-hexyl-4-methoxybenzenesulfonamide (2b)**

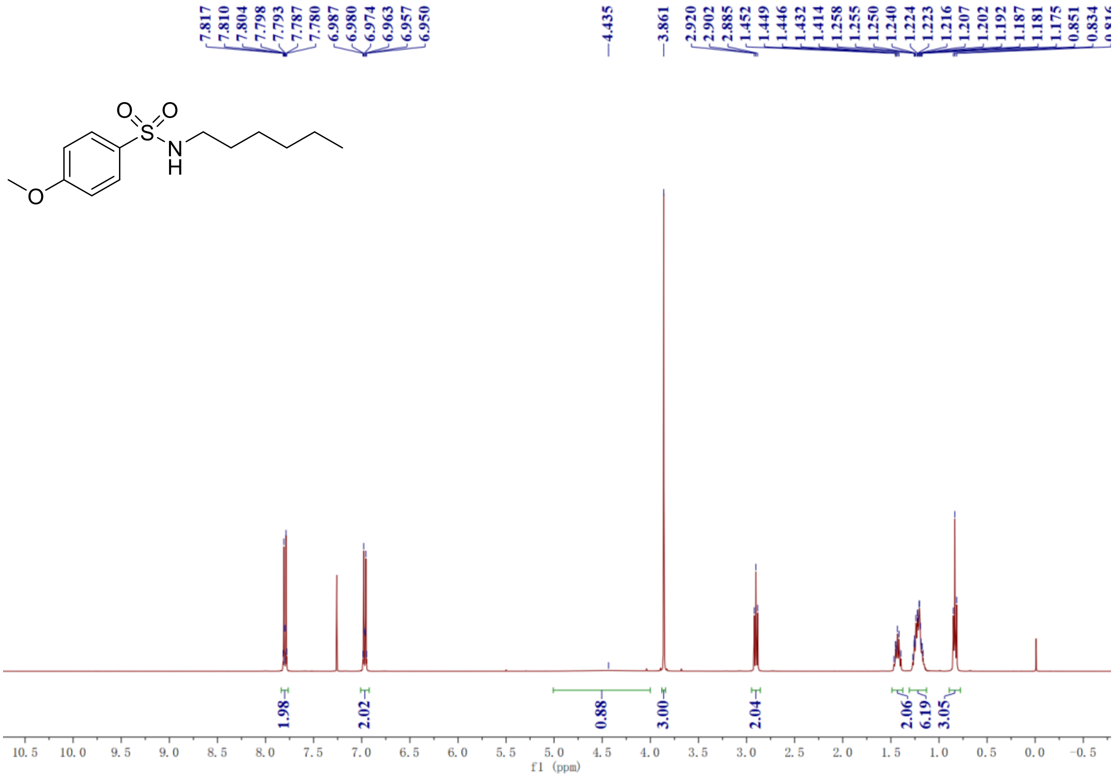

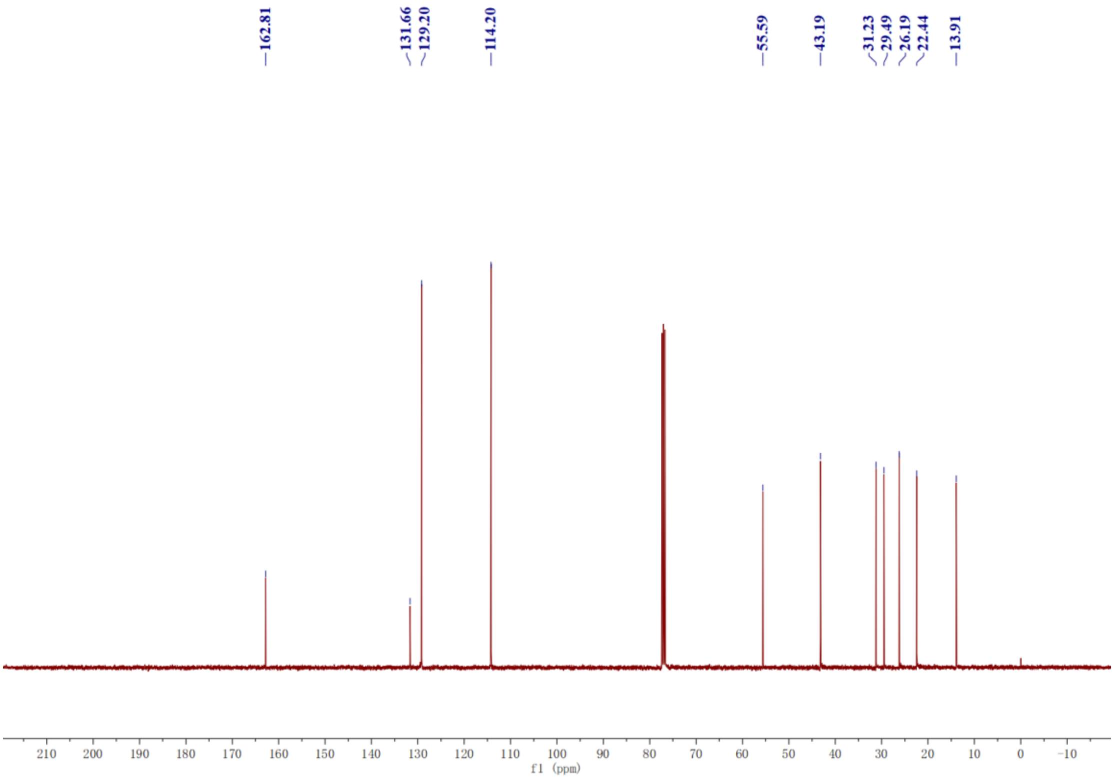

N-heptyl-4-methoxybenzenesulfonamide (2c)

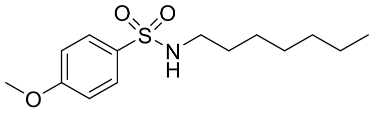

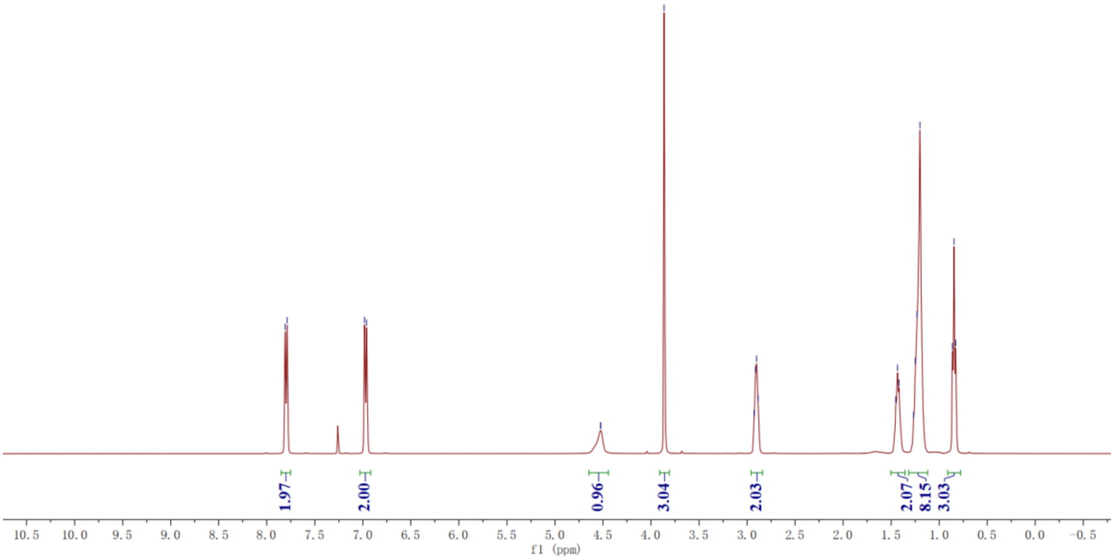

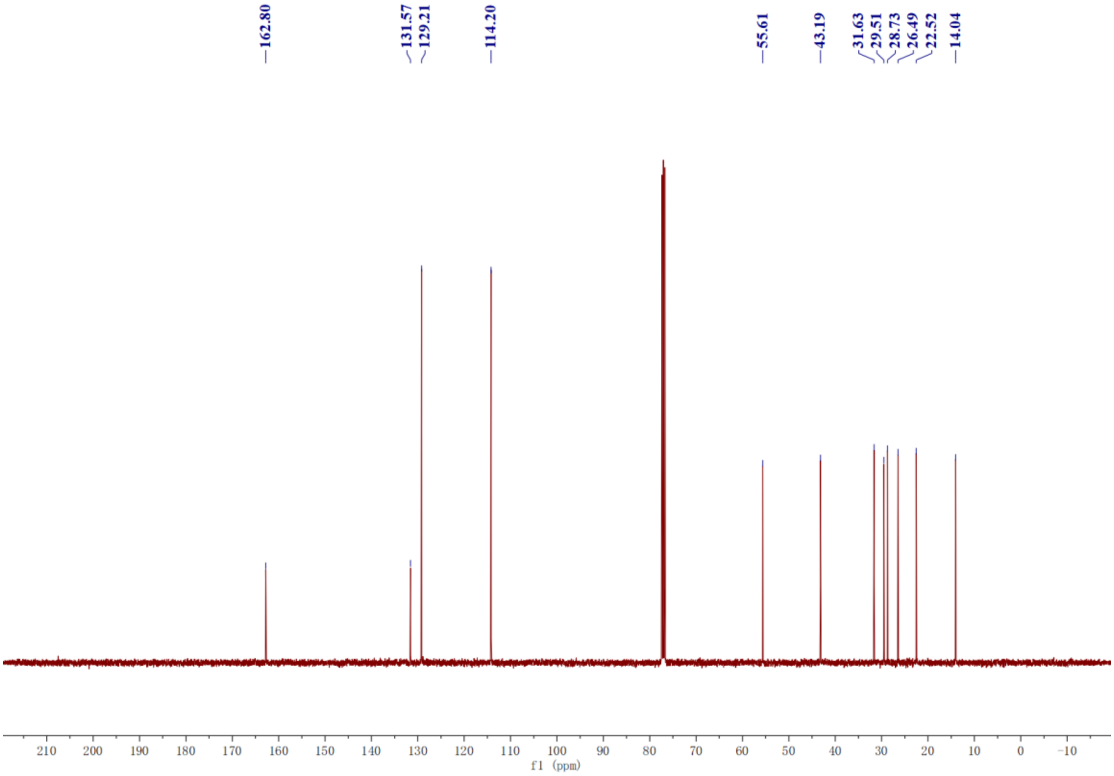

4-methoxy-*N*-octylbenzenesulfonamide (2d)

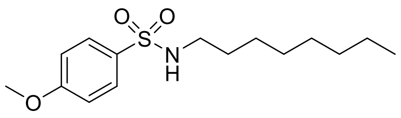

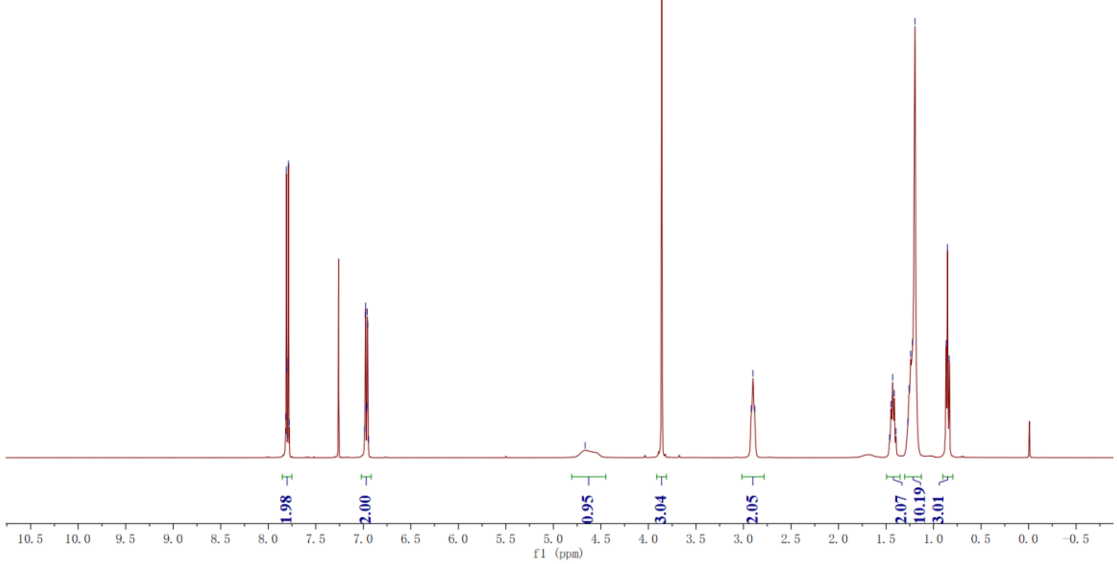

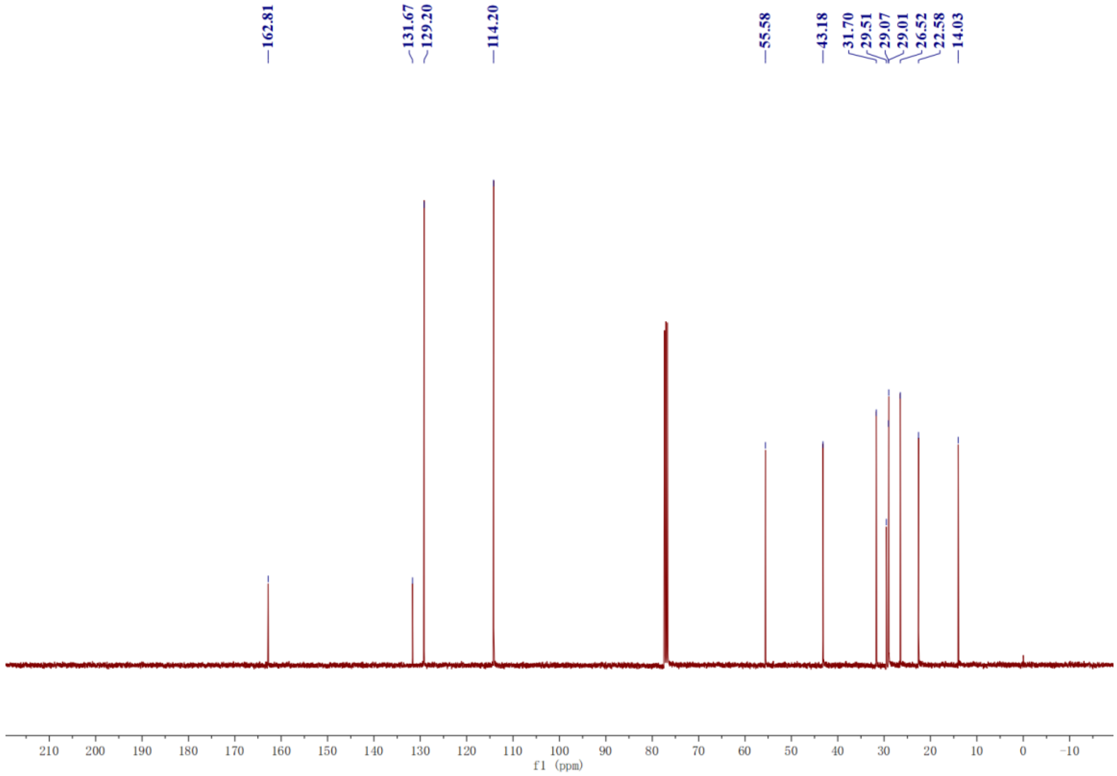

***N*-(2,2-dimethylhexyl)-4-methoxybenzenesulfonamide (2e)**

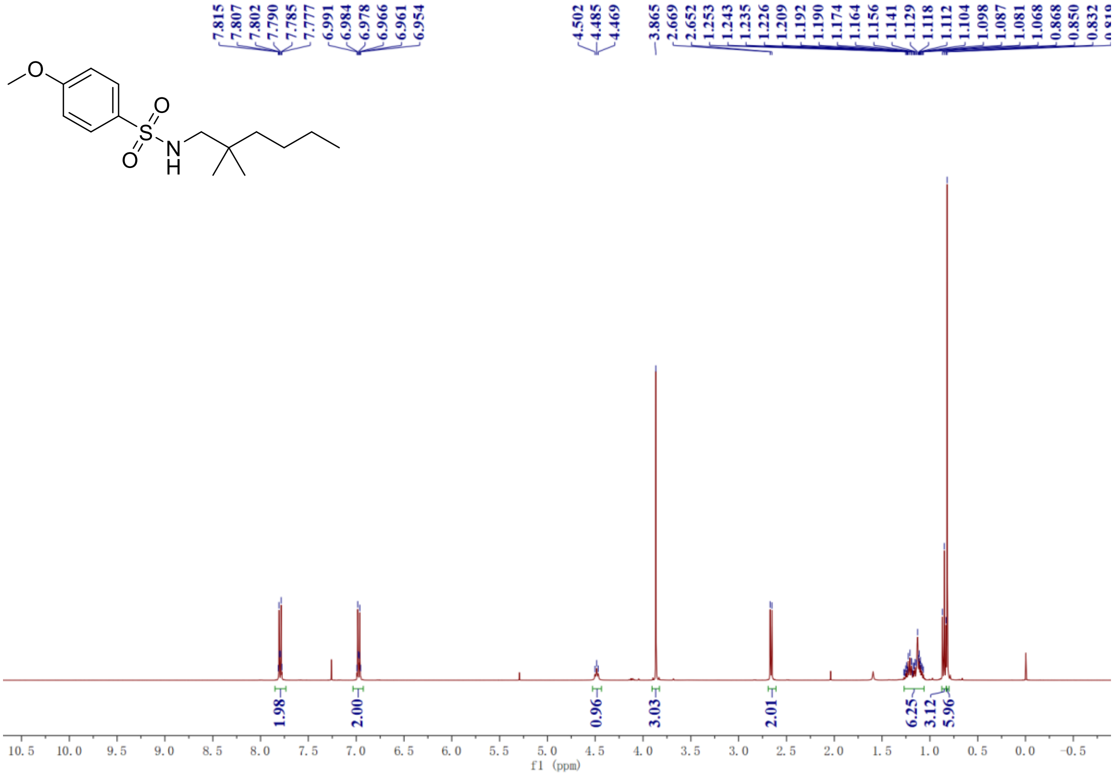

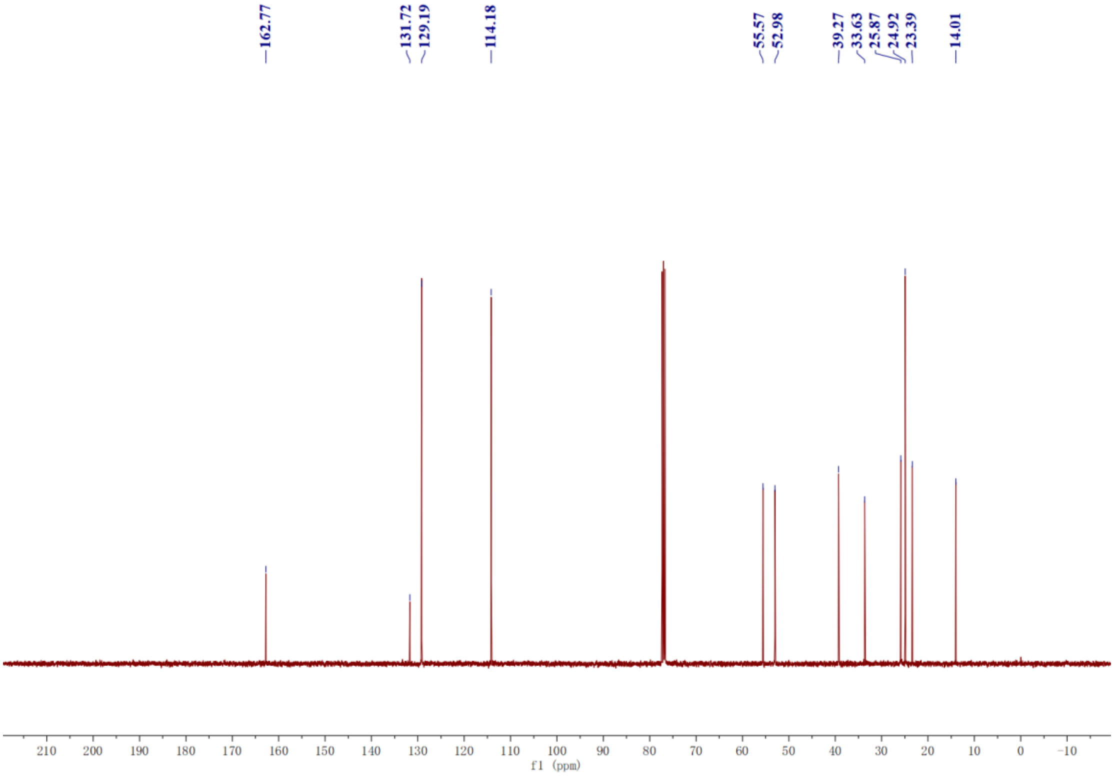

***N*-(2-ethylhexyl)-4-methoxybenzenesulfonamide (2f)**

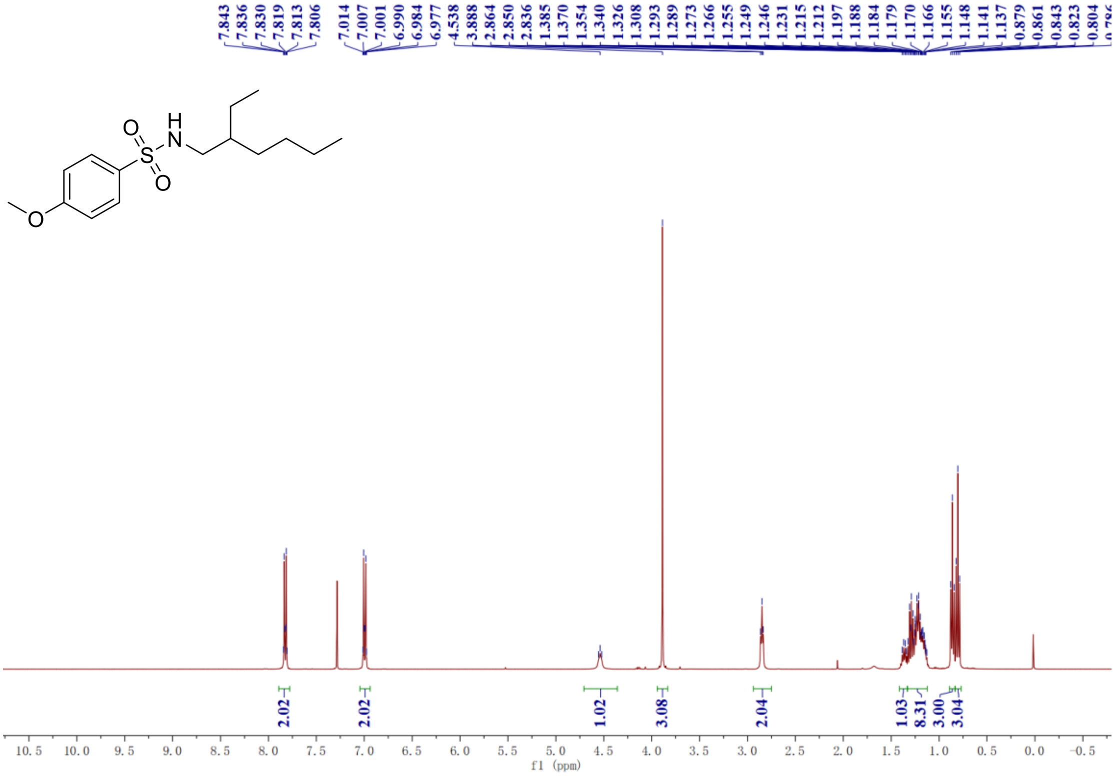

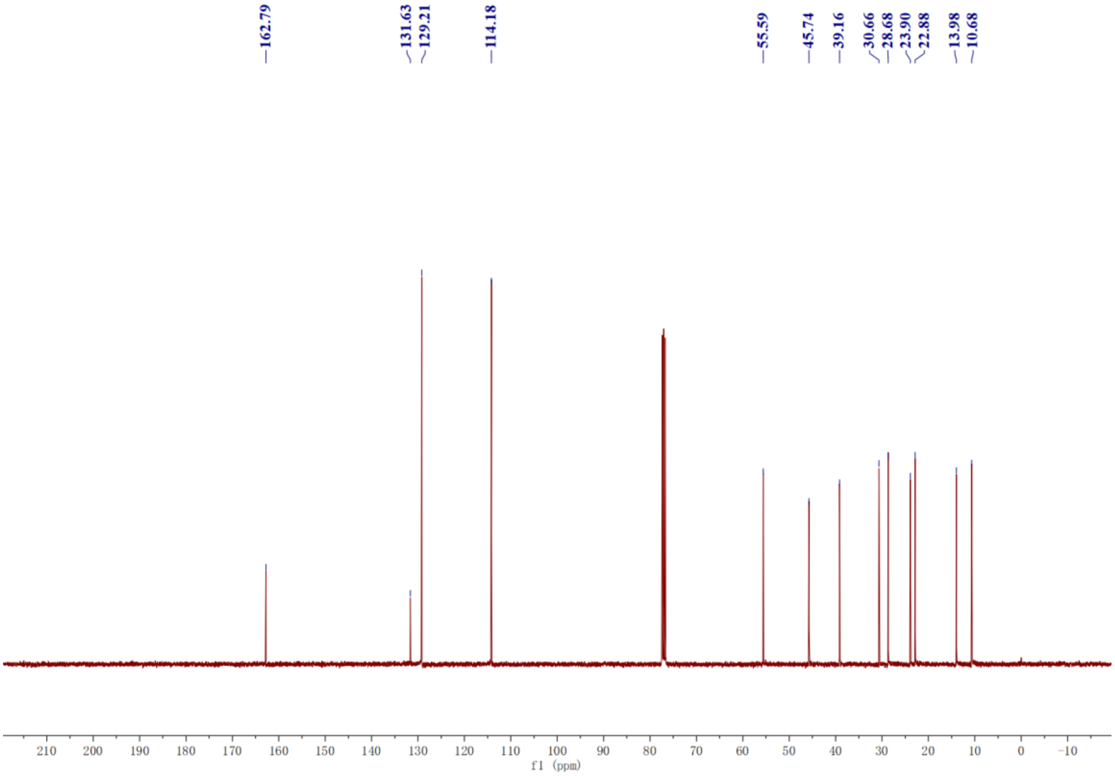

4-methoxy-*N*-(5-methylhexyl)benzenesulfonamide (2g)

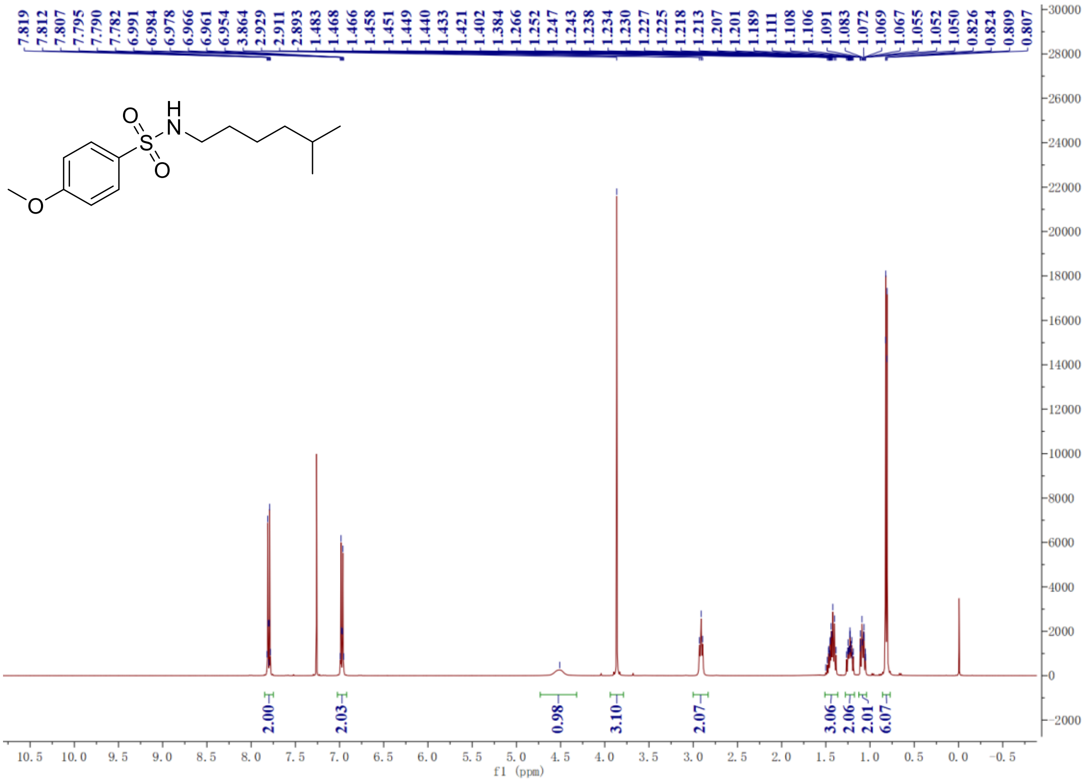

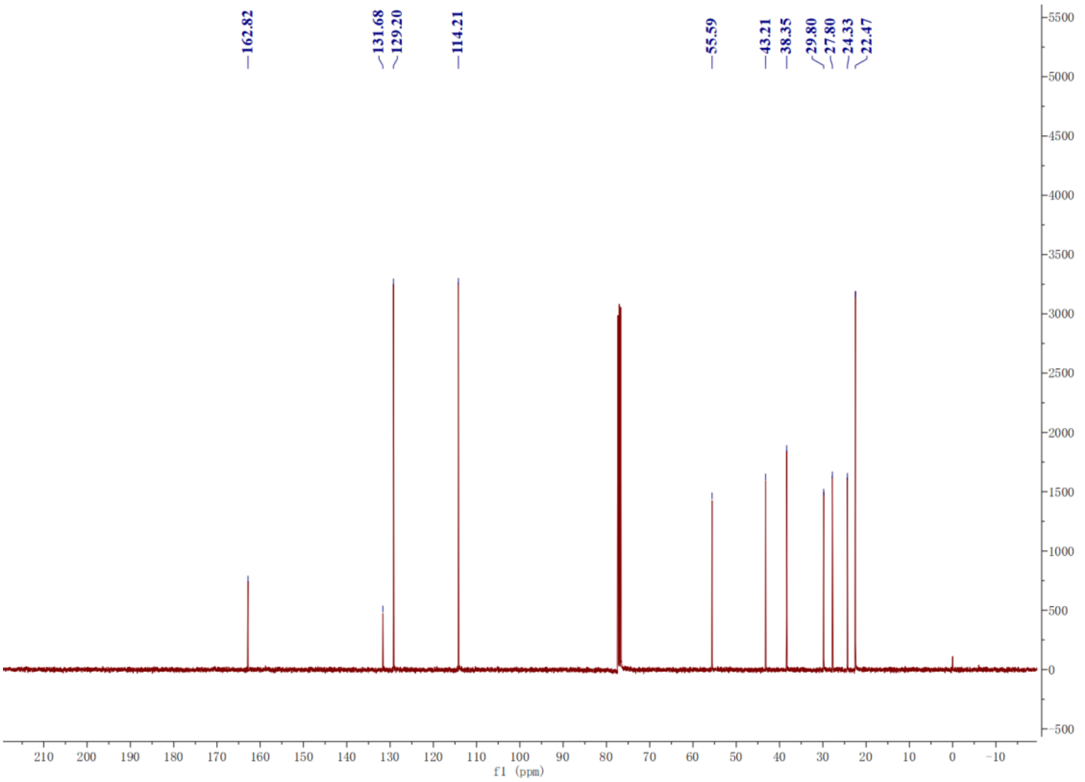

4-methoxy-*N*-(1-phenylpentyl)benzenesulfonamide (2h)

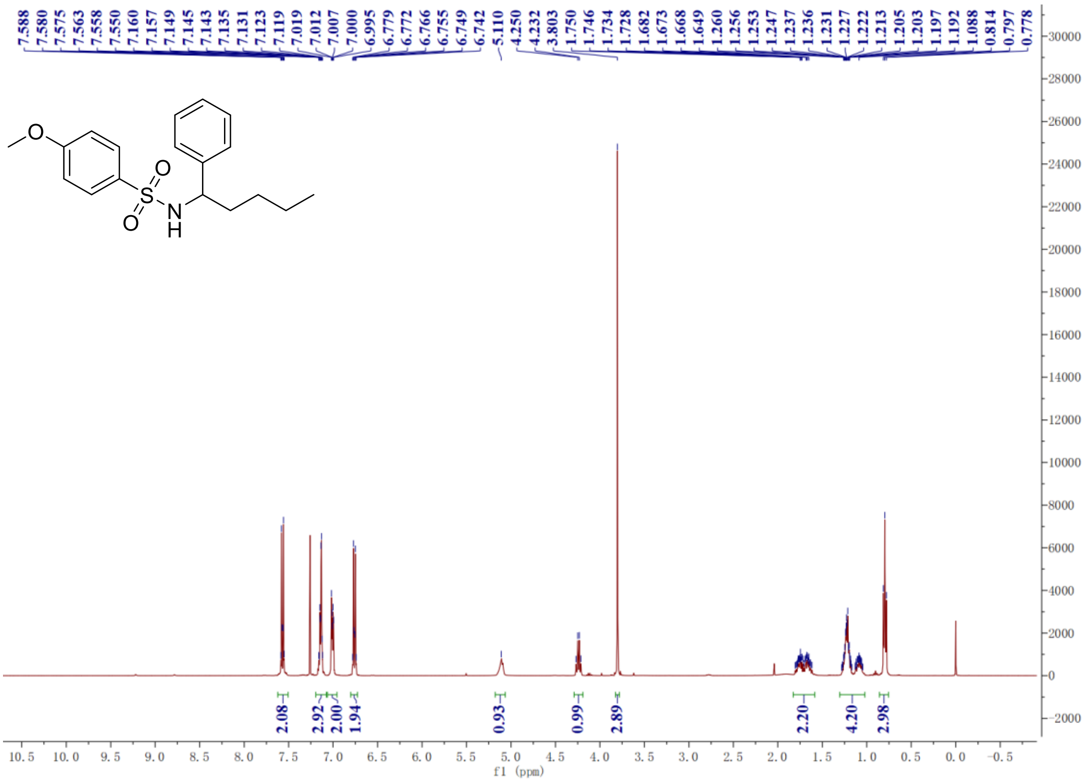

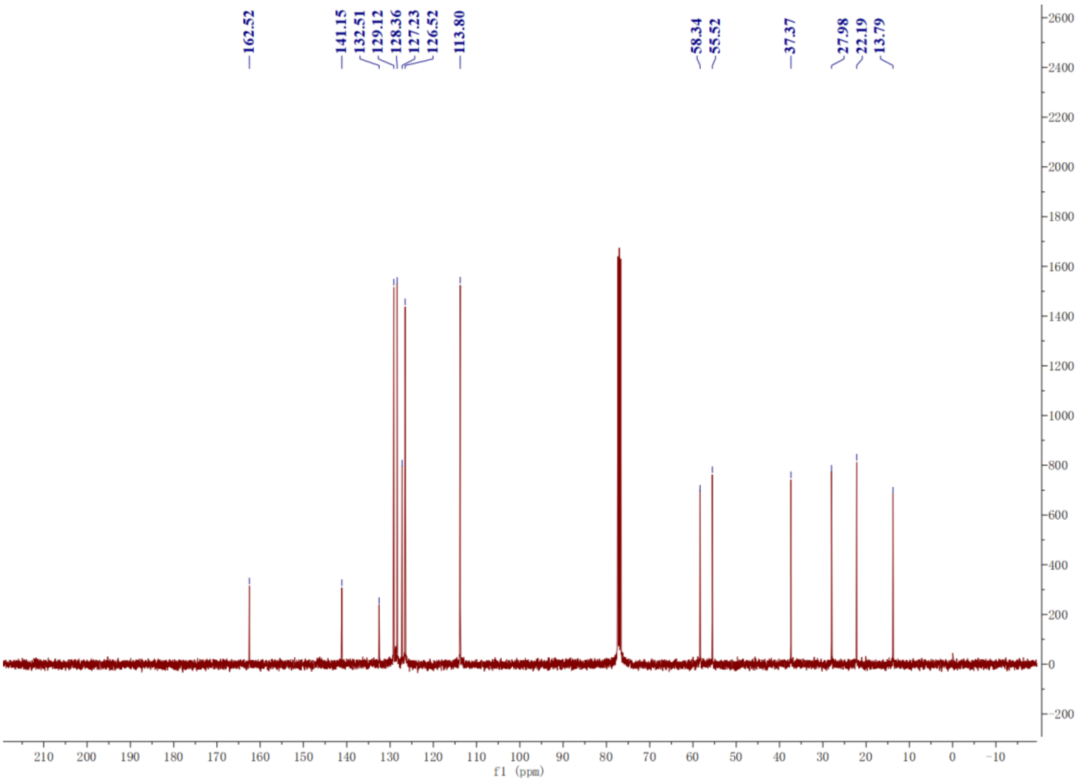

methyl (*S*)-2-((4-methoxyphenyl)sulfonamido)hexanoate (2i)

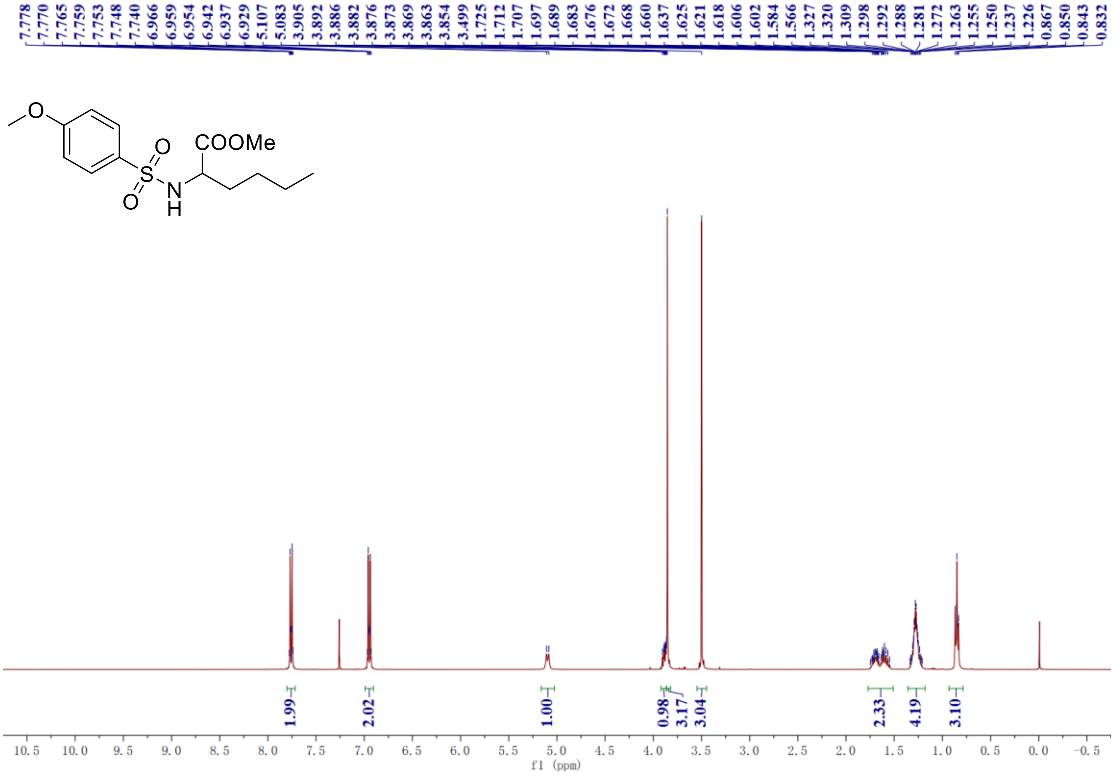

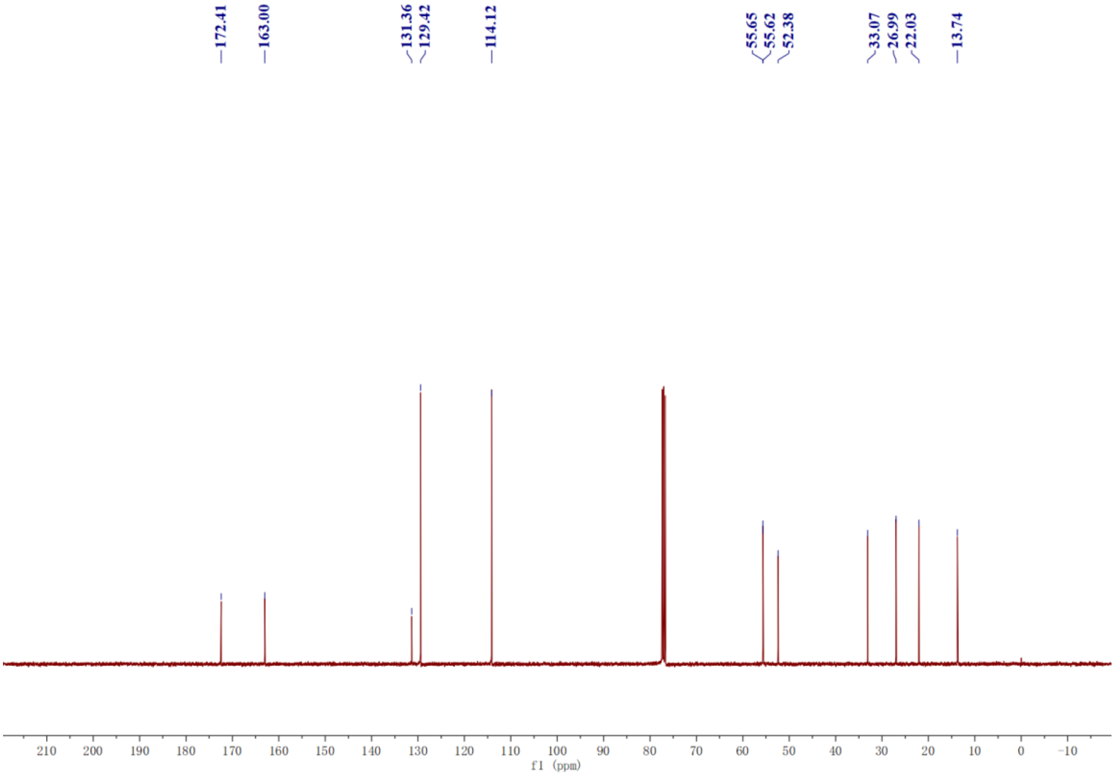

4-methoxy-*N*-(2-methylpentyl)benzenesulfonamide (2j)

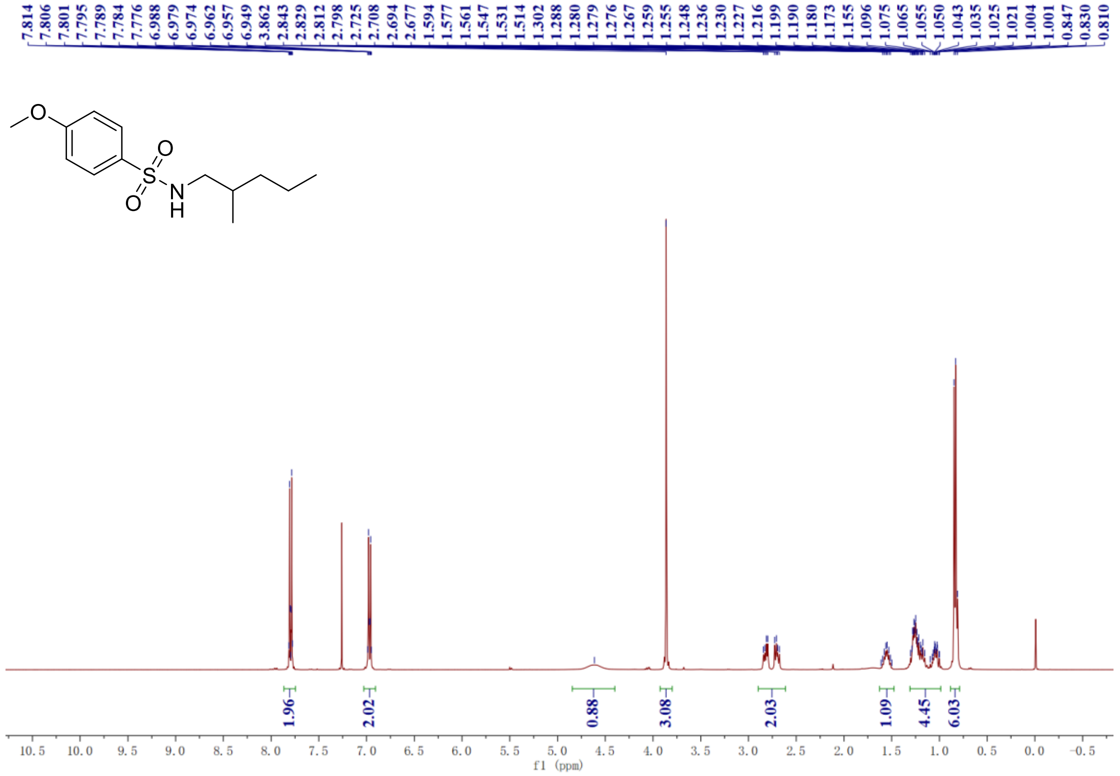

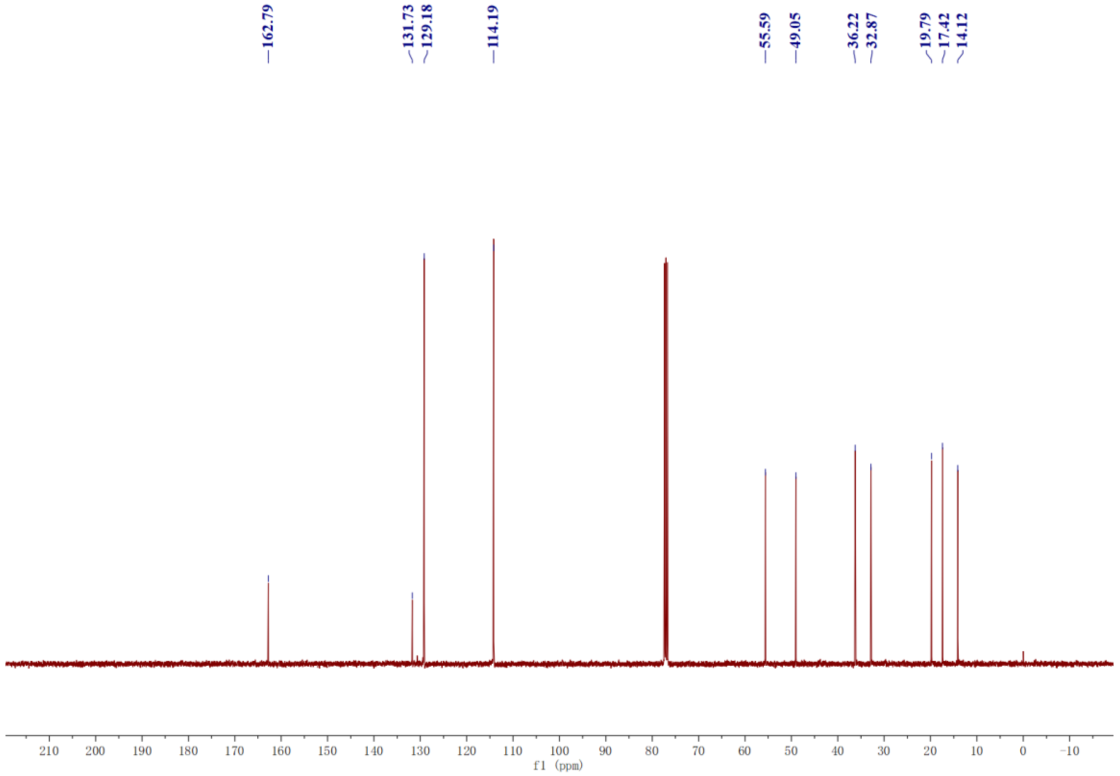

4-methoxy-*N*-(3-methylpentyl)benzenesulfonamide (2k)

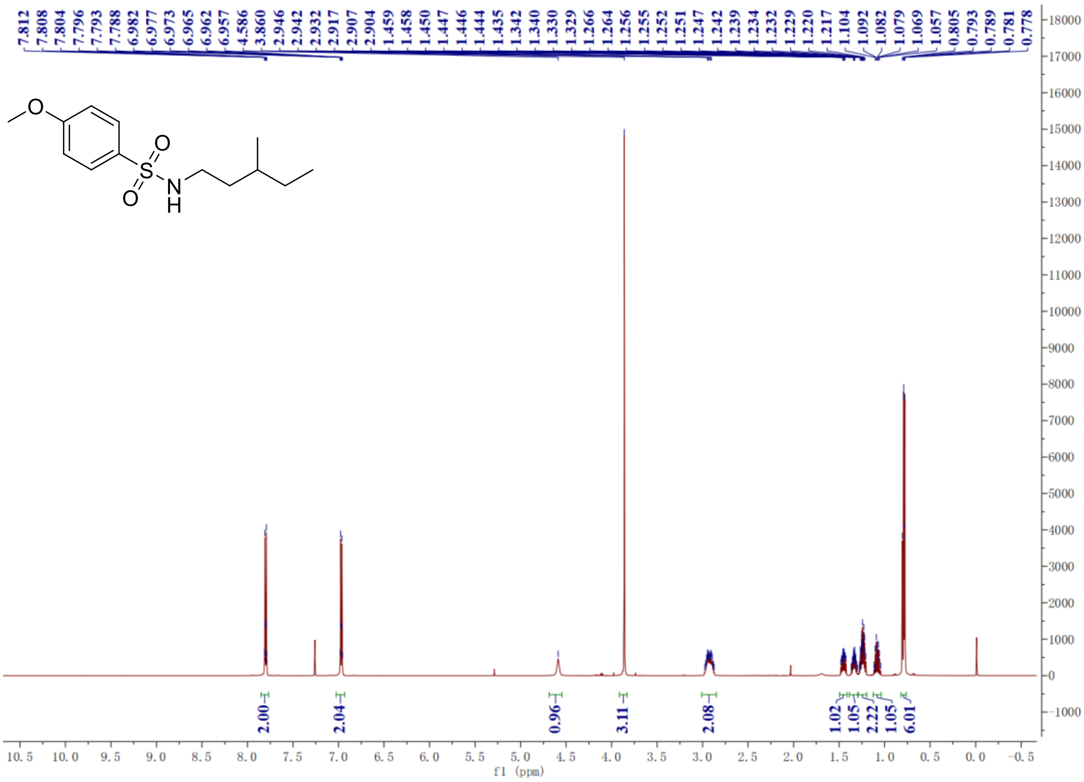

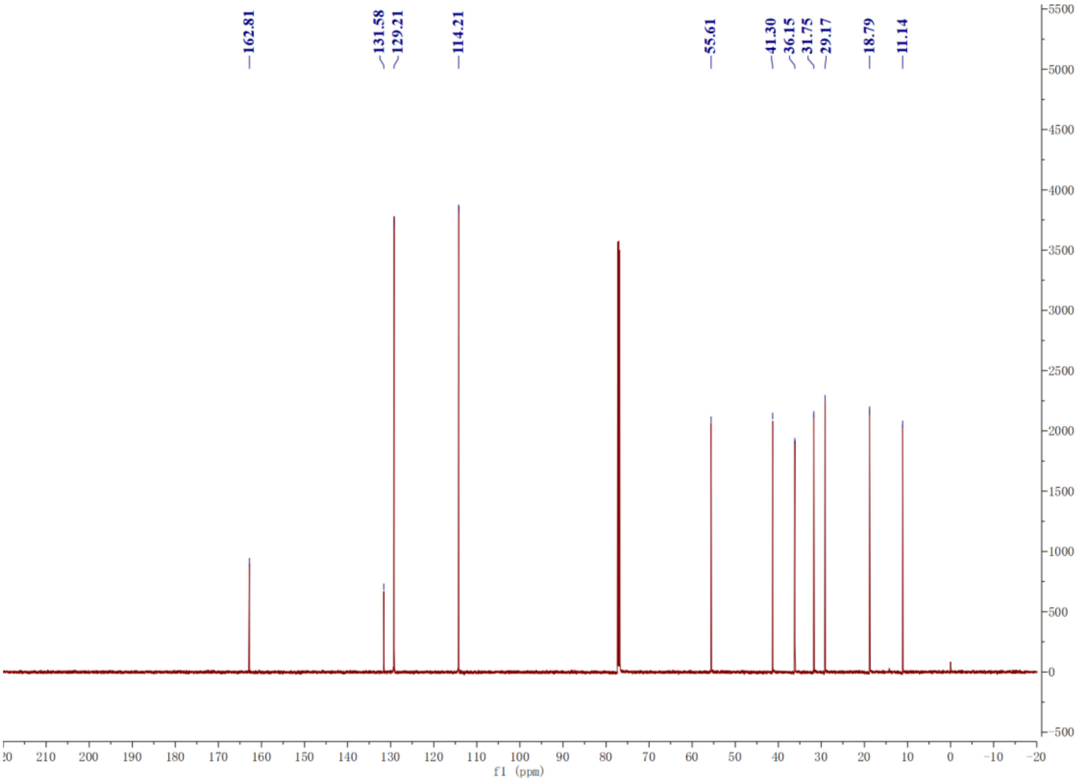

N-(2-ethoxyethyl)-4-methoxybenzenesulfonamide (2l)

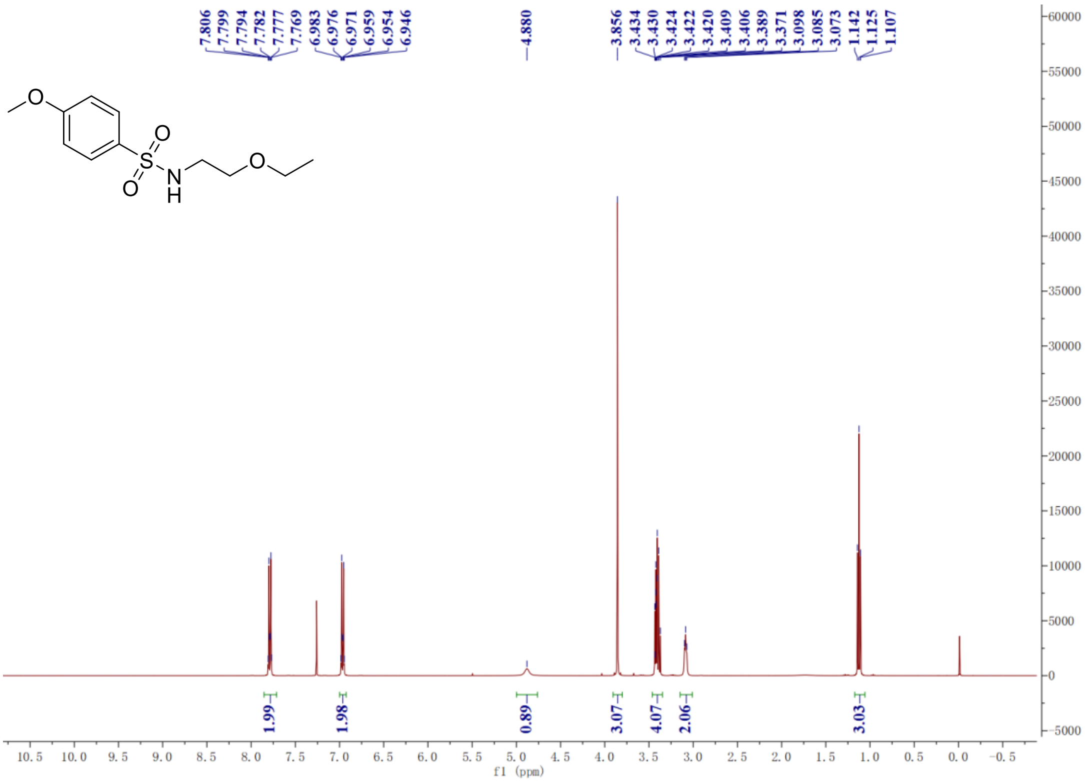

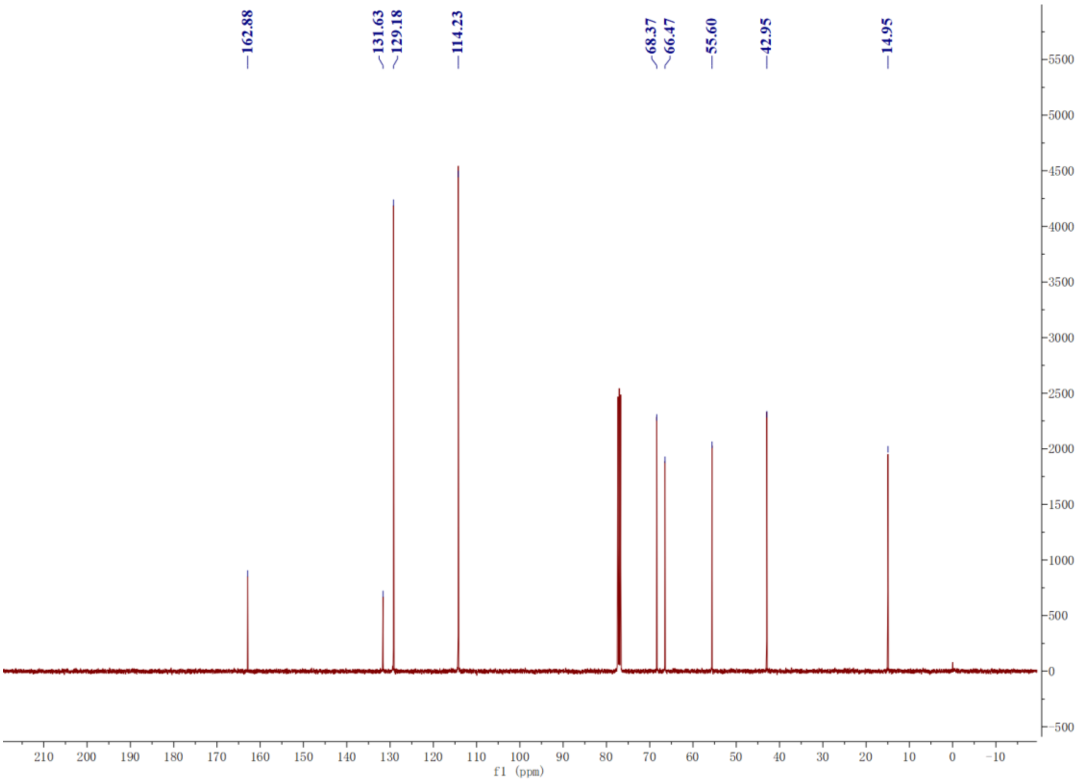

***N*-(6-chlorohexyl)-4-methoxybenzenesulfonamide (2m)**

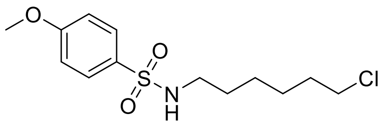

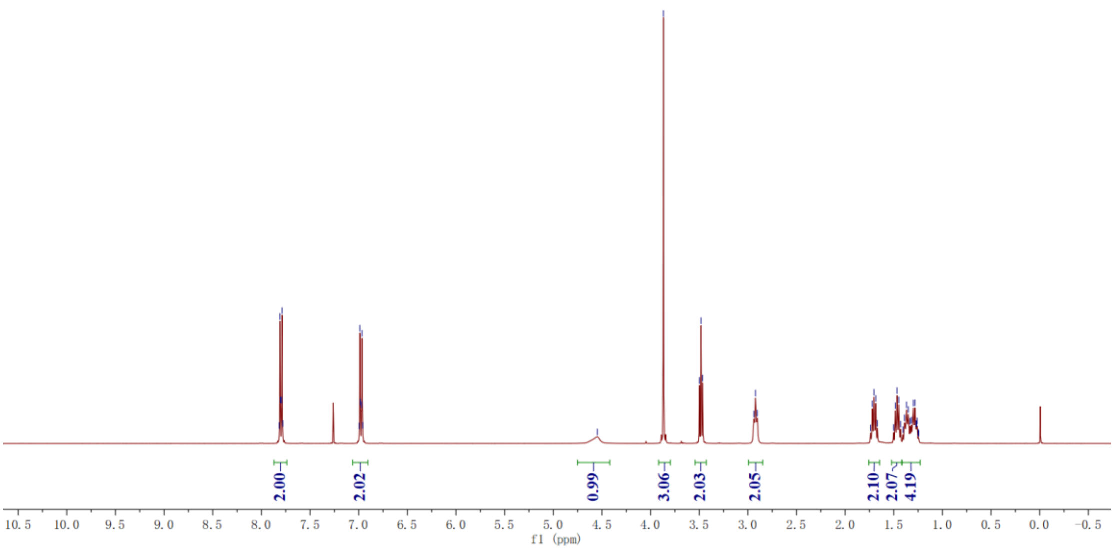

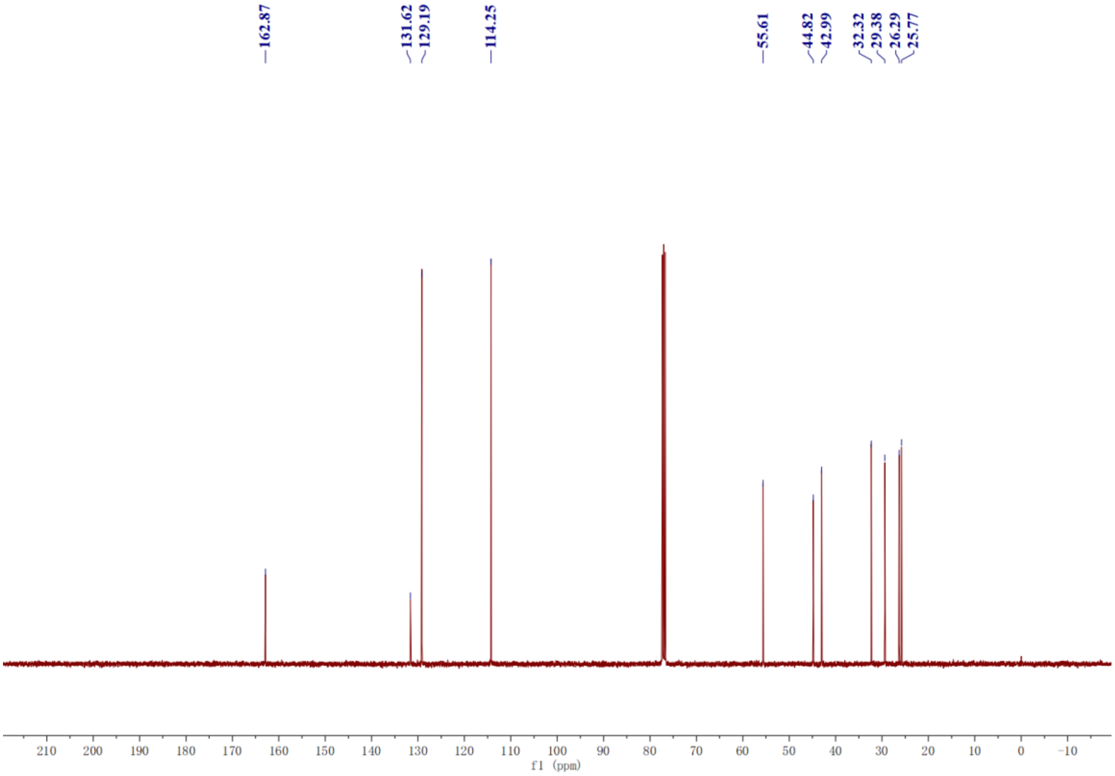

ethyl 8-((4-methoxyphenyl)sulfonamido)octanoate (2n)

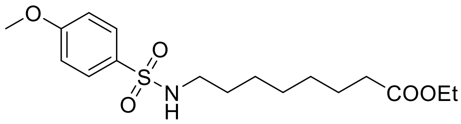

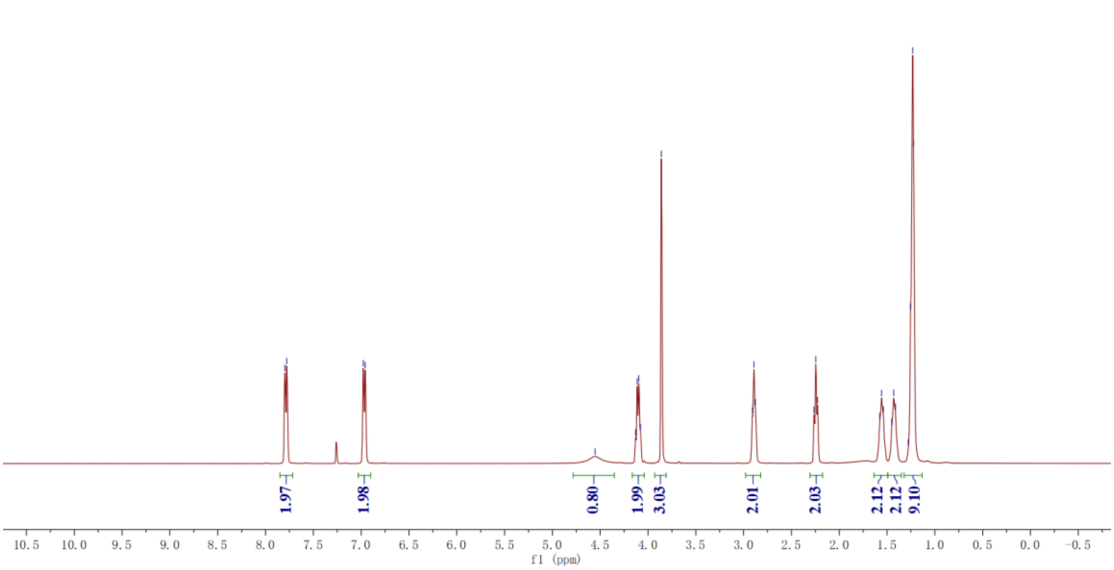

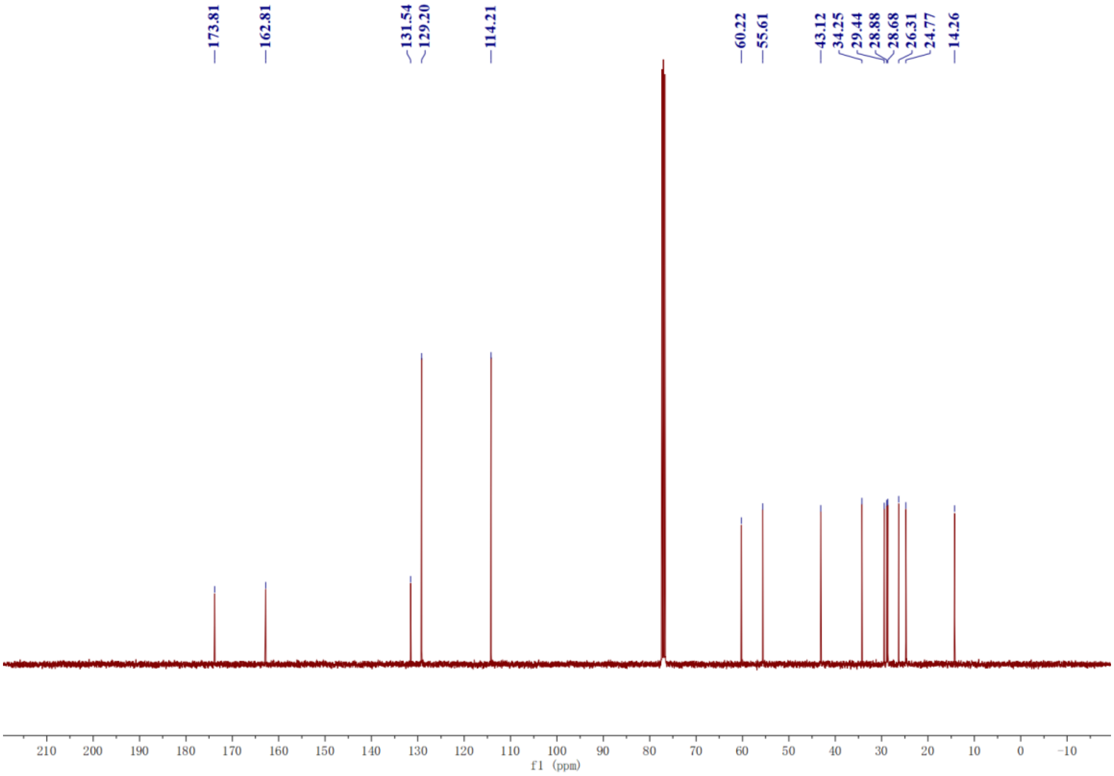

6-((4-methoxyphenyl)sulfonamido)hexyl 4-methylbenzoate (2o)

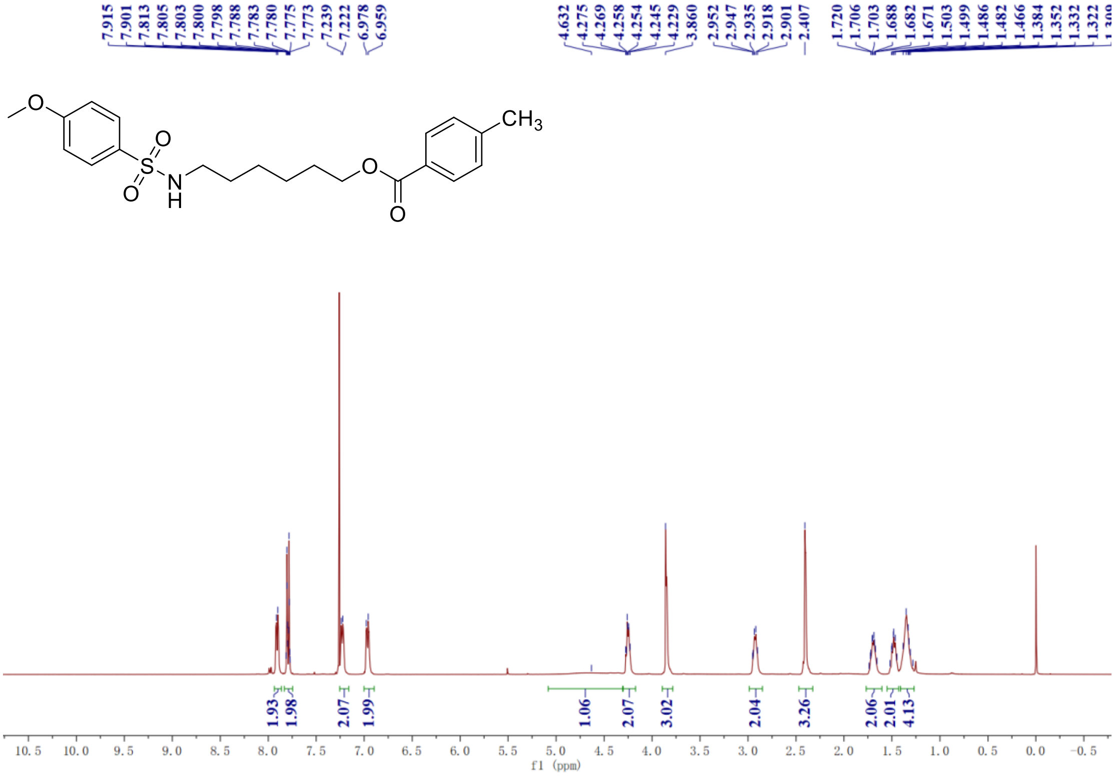

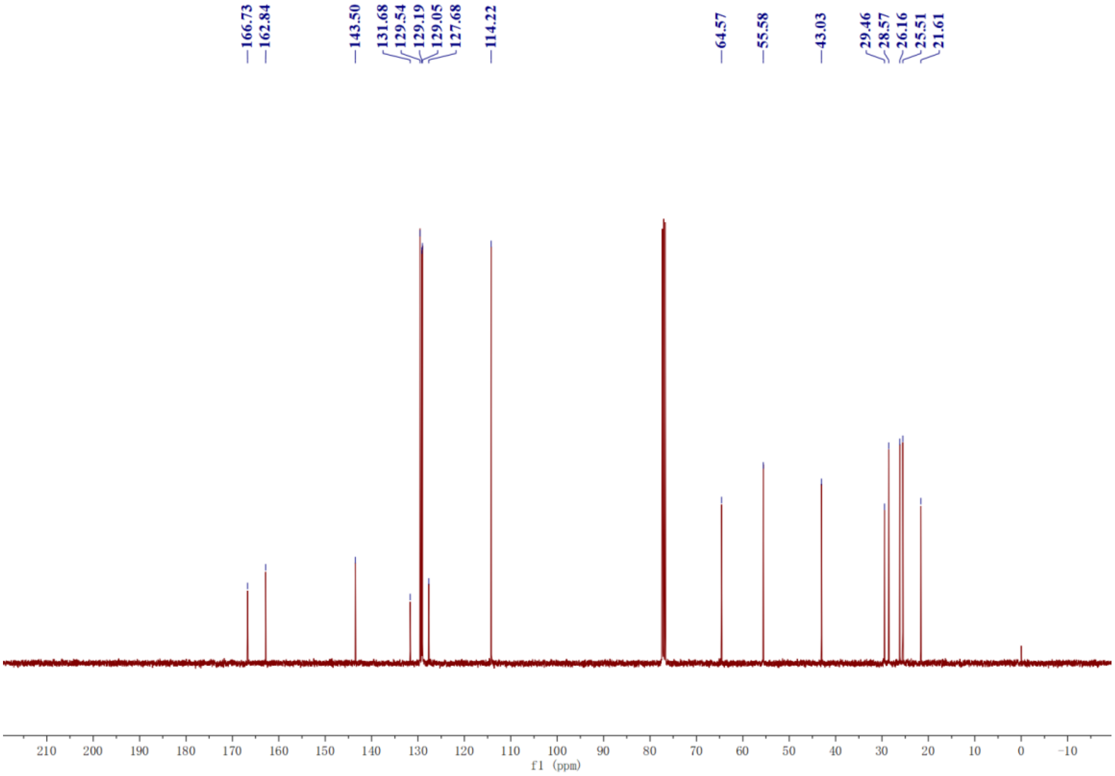

***N*-(cyclohexylmethyl)-4-methoxybenzenesulfonamide (2p)**

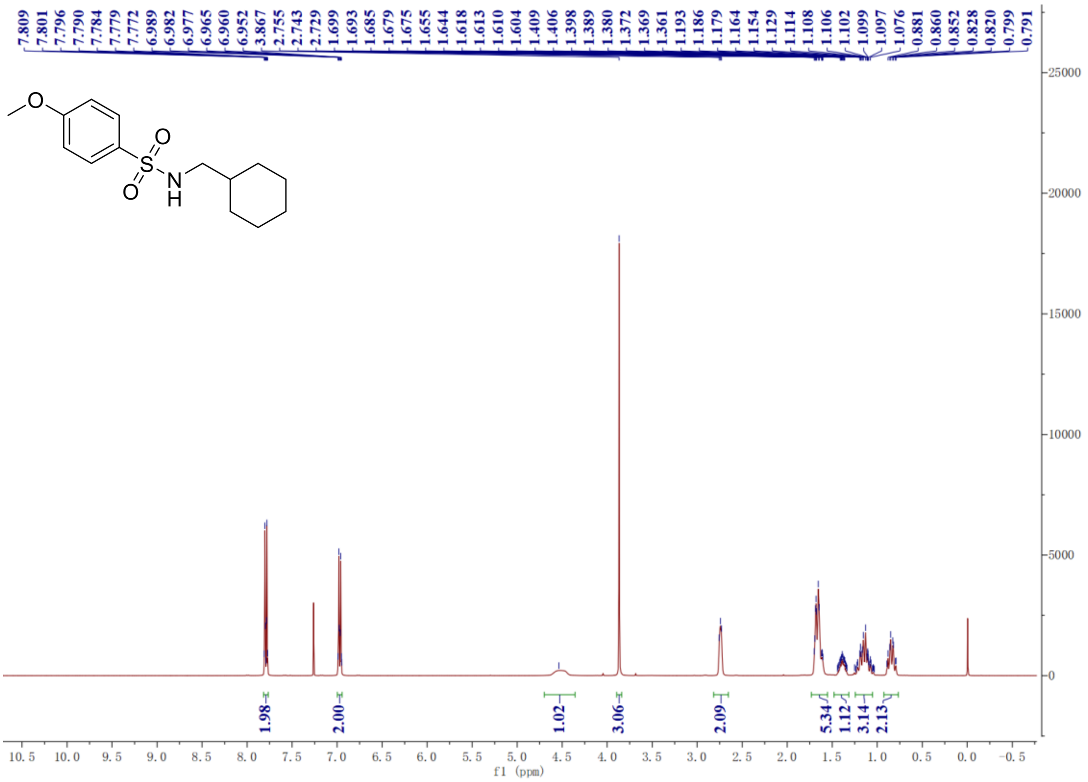

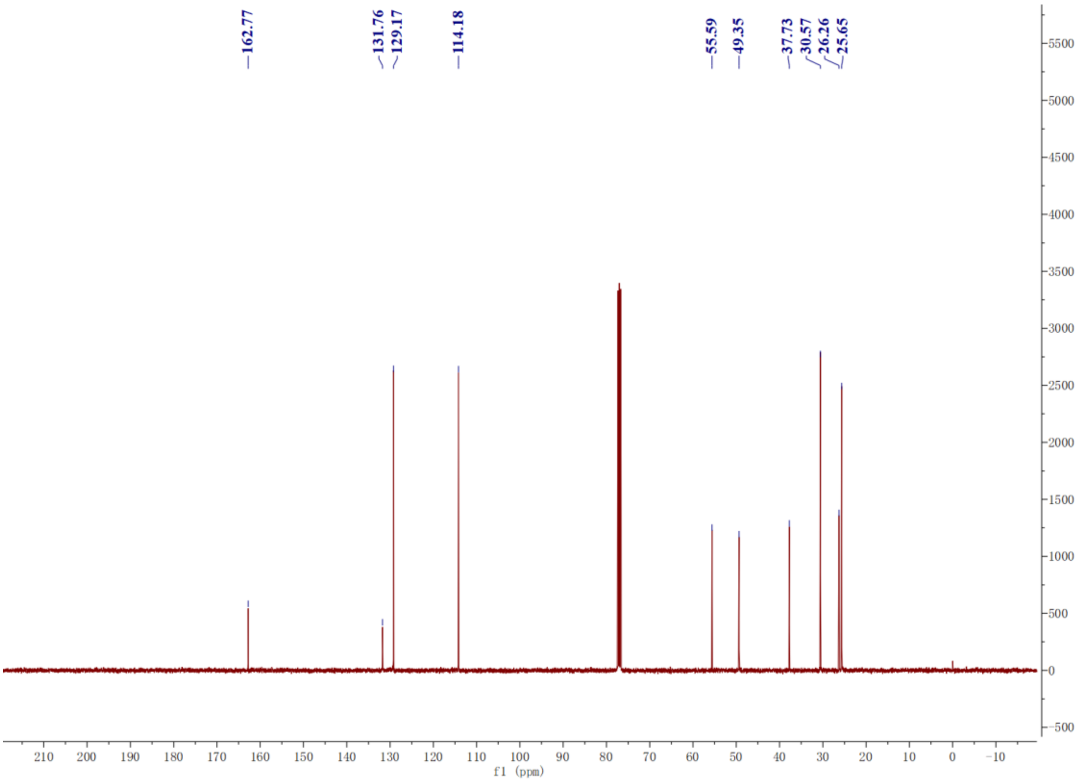

***N*-(2-cyclopentylethyl)-4-methoxybenzenesulfonamide (2q)**

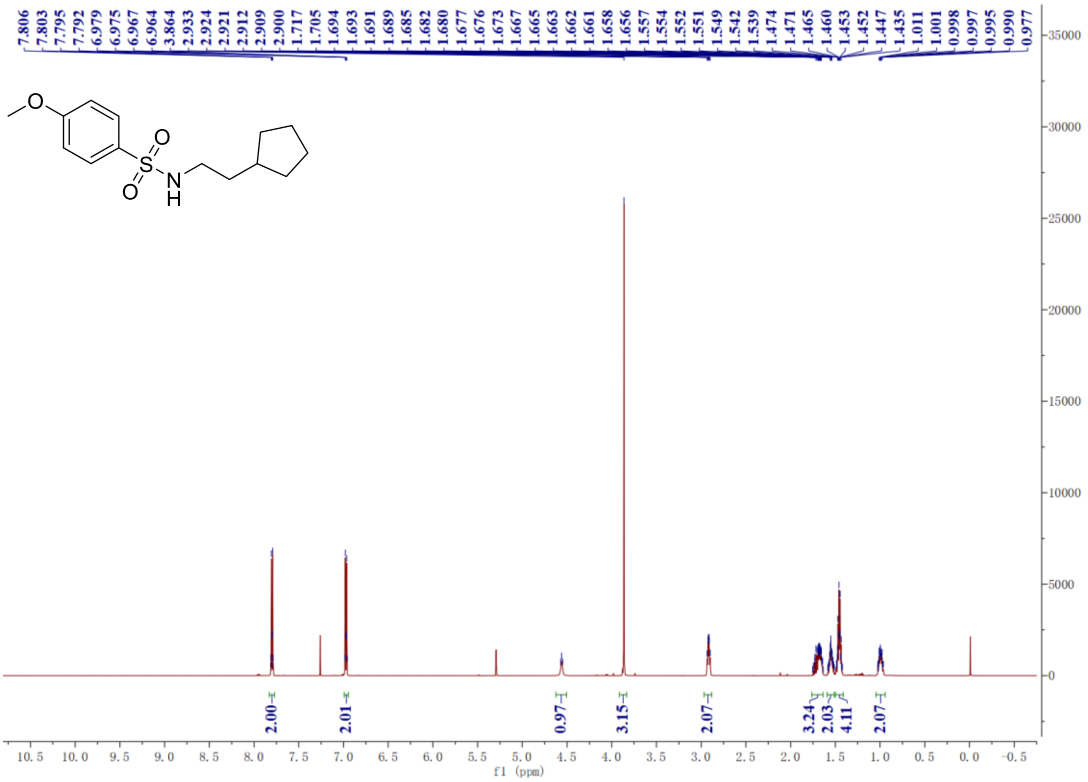

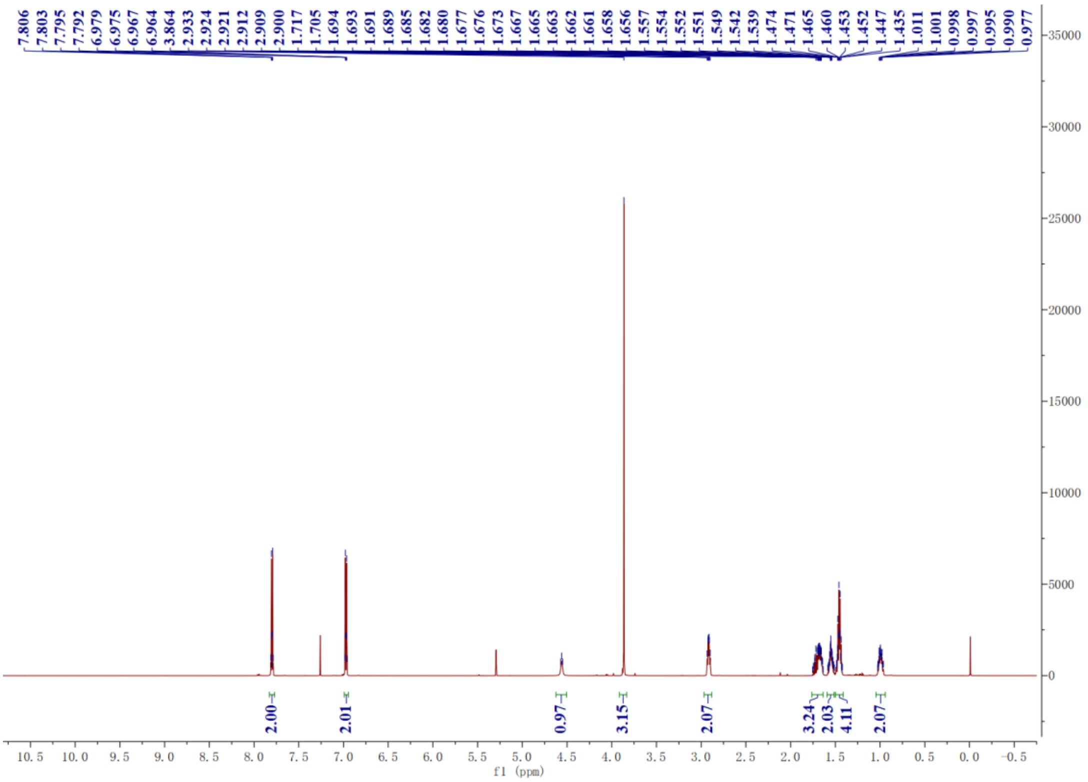

N-(2-cyclohexylethyl)-4-methoxybenzenesulfonamide (2r)

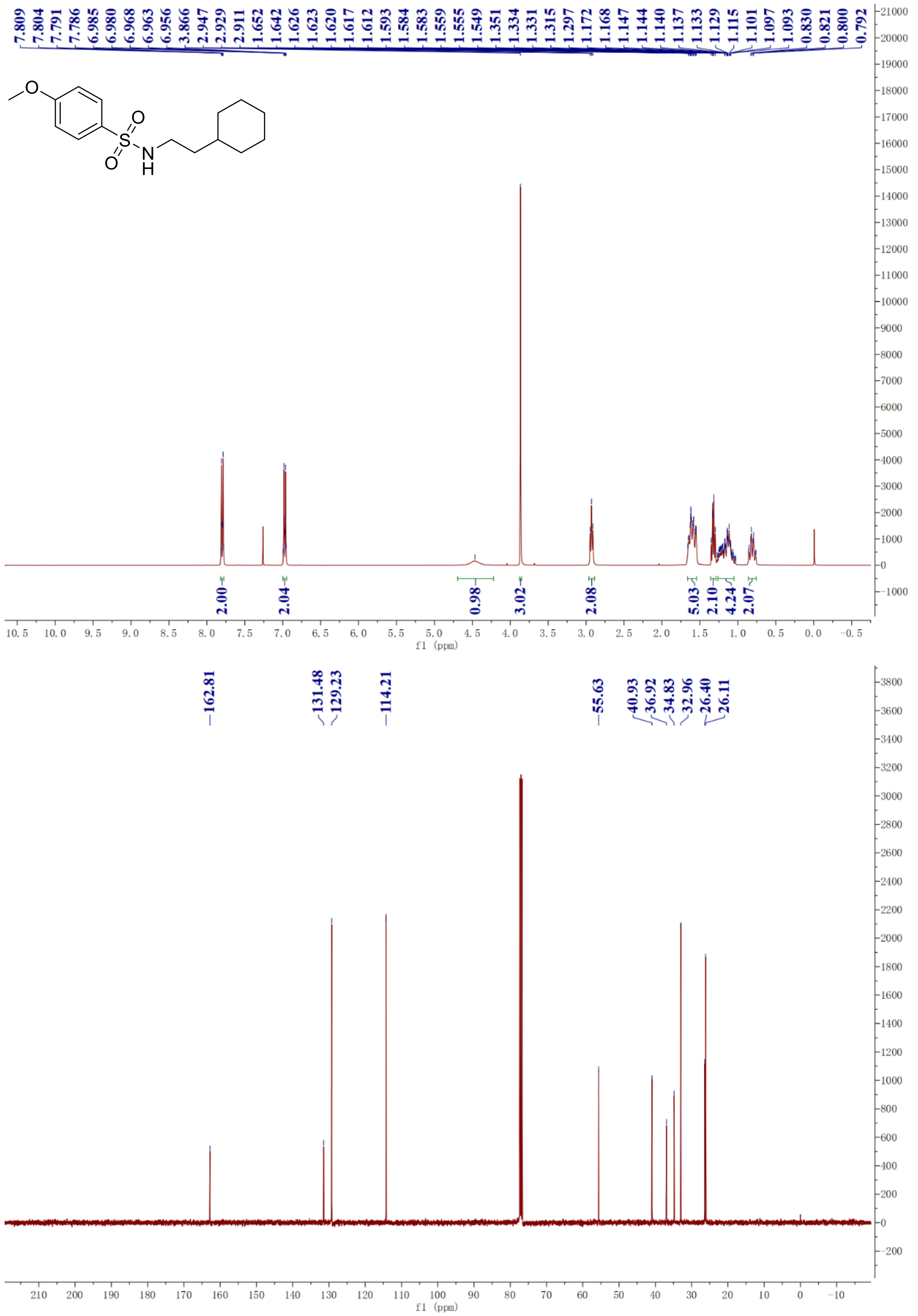

4-methoxy-*N*-(4-phenylbutyl)benzenesulfonamide (2s)

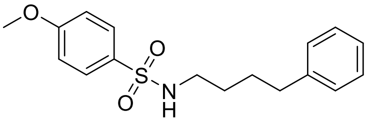

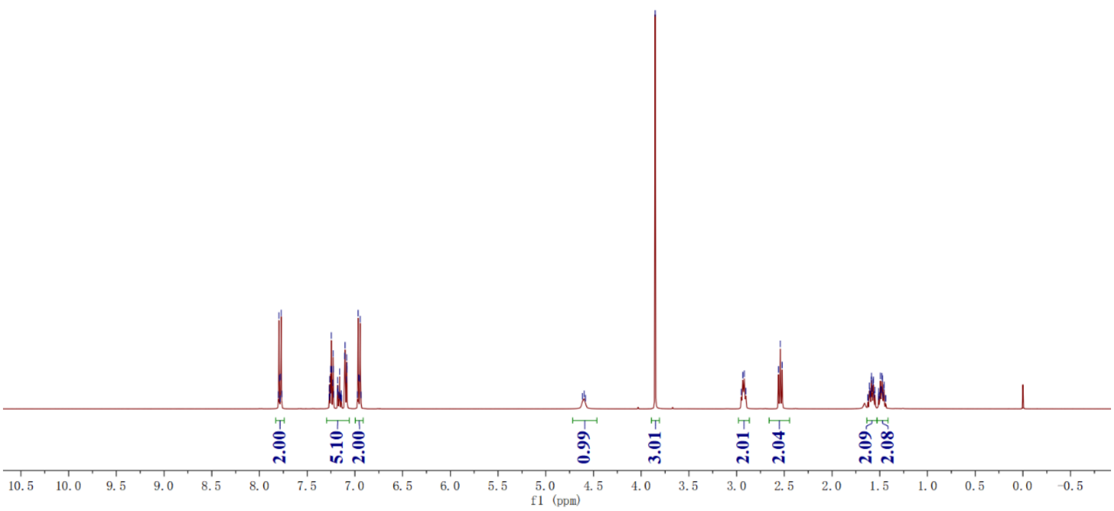

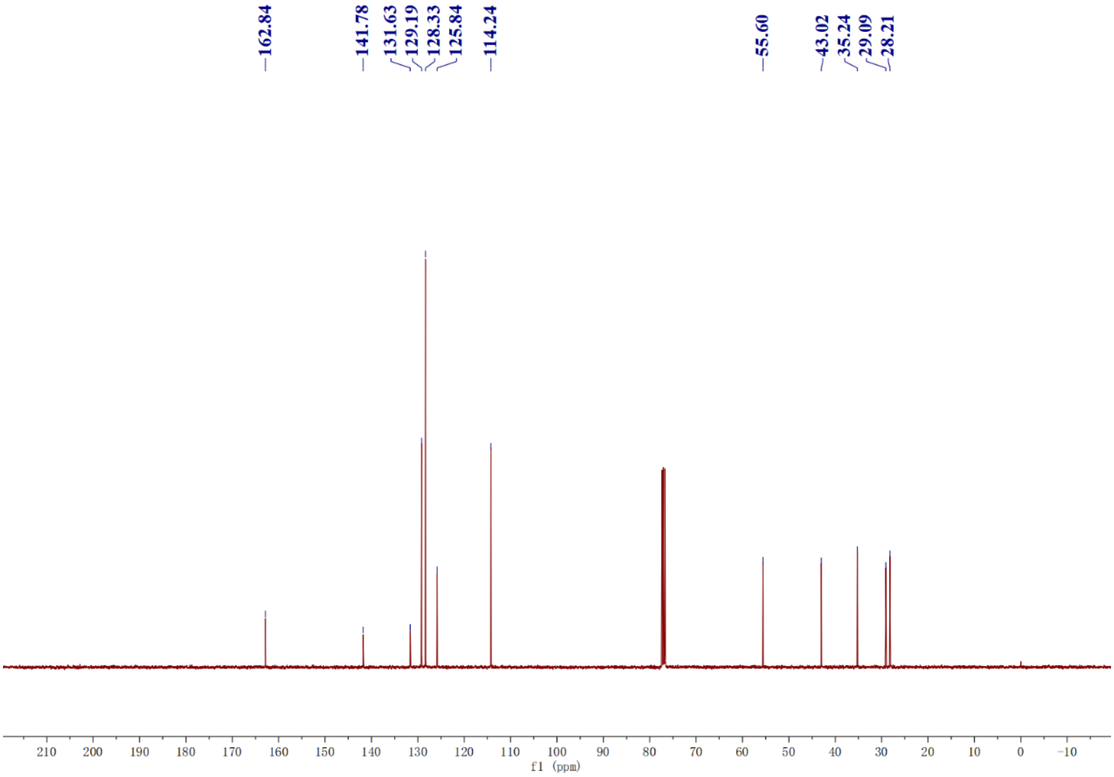

N-(3-cyclopentylpropyl)-4-methoxybenzenesulfonamide (2t)

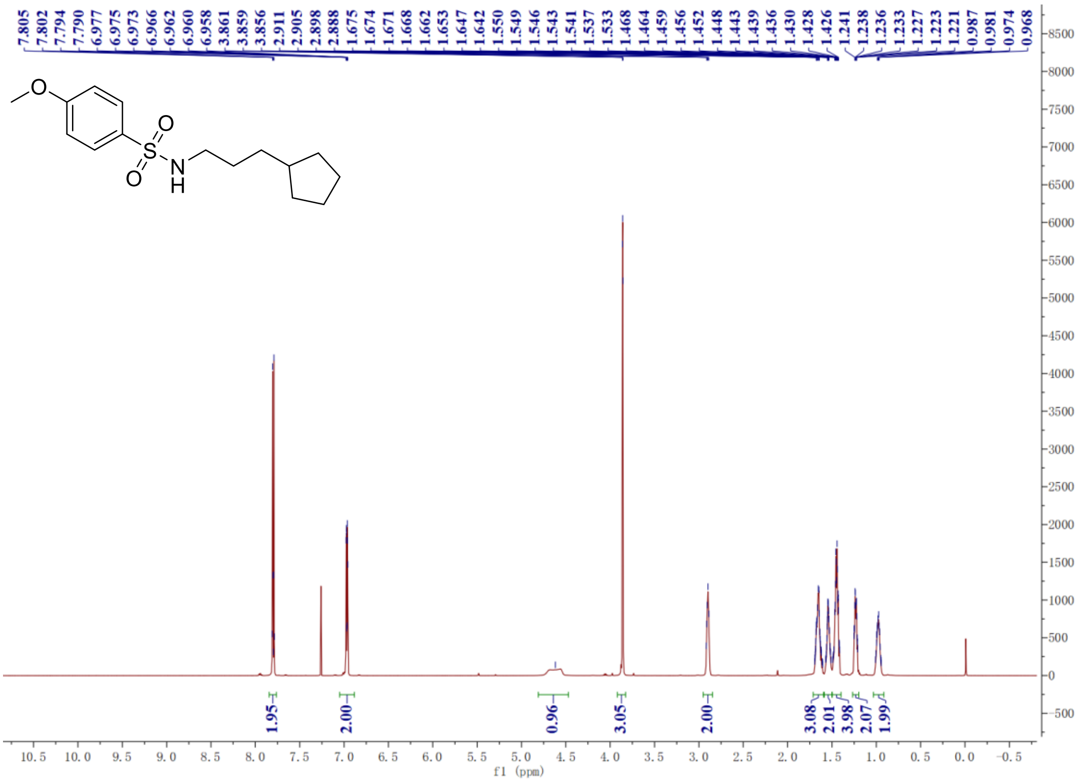

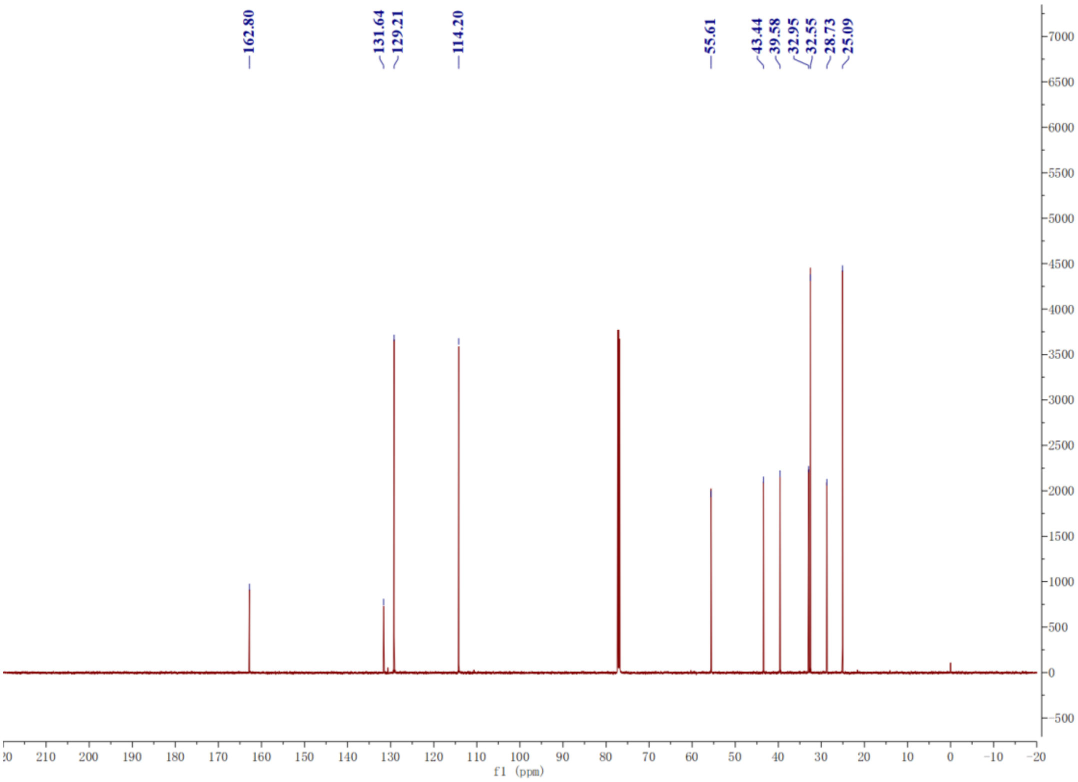

N-(3-cyclohexylpropyl)-4-methoxybenzenesulfonamide (2u)

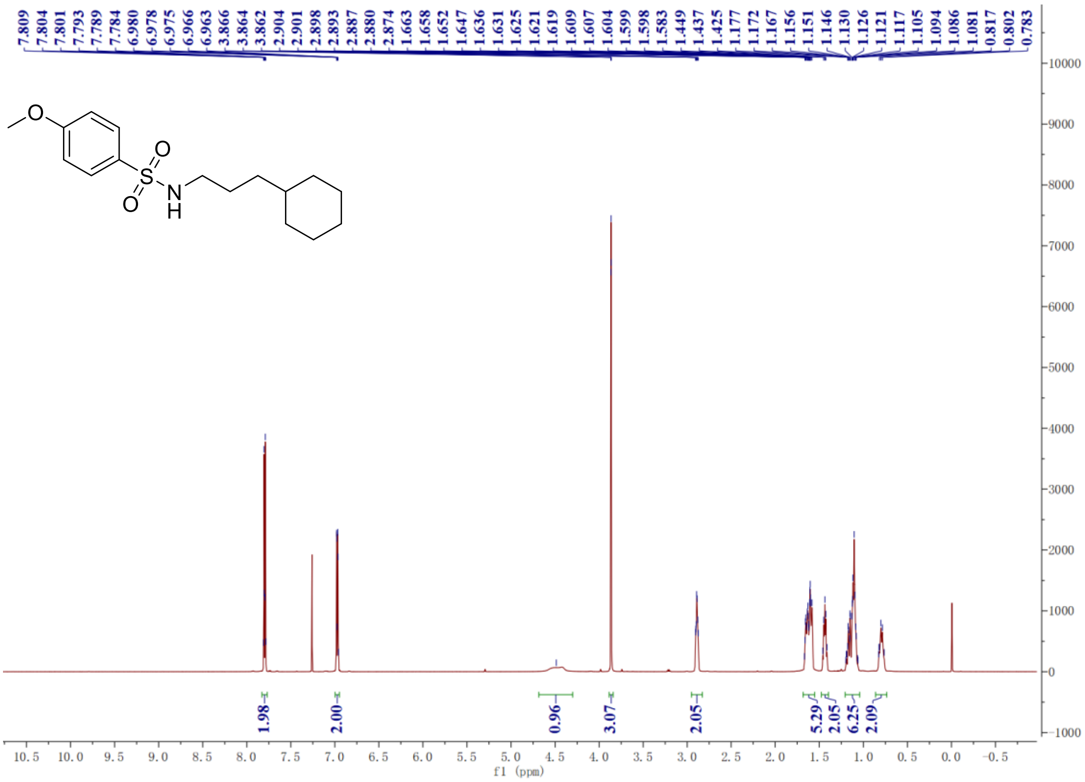

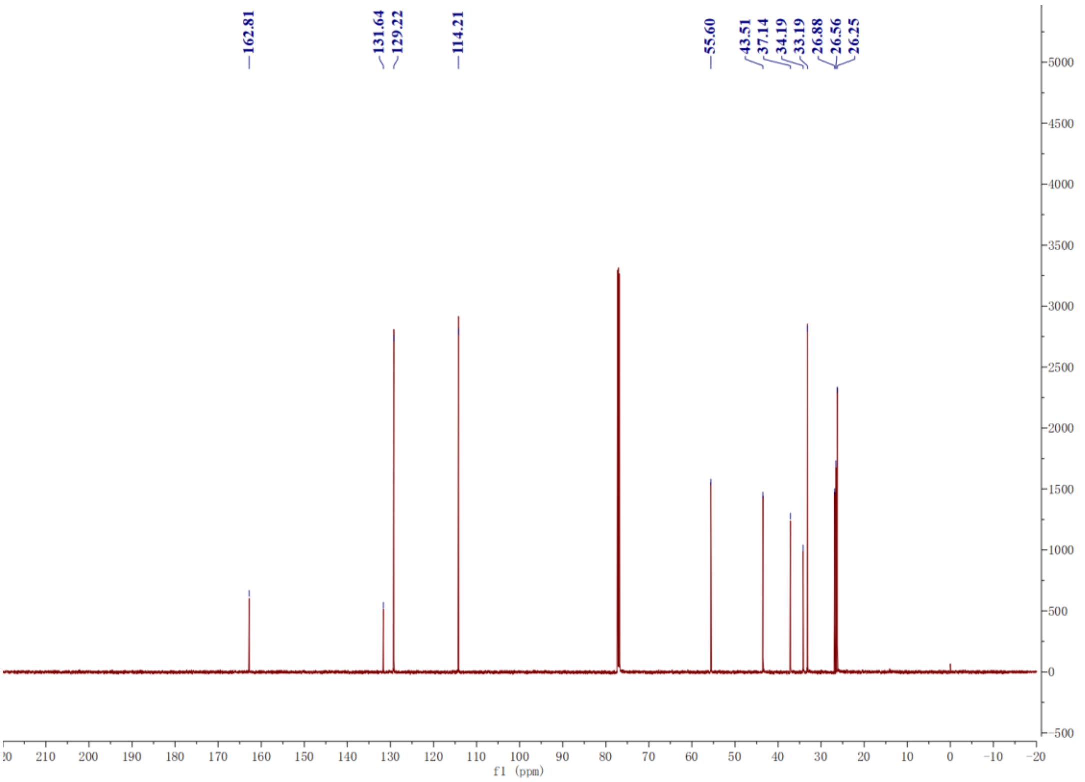

4-methoxy-*N*-(4-methylpentyl)benzenesulfonamide (2v)

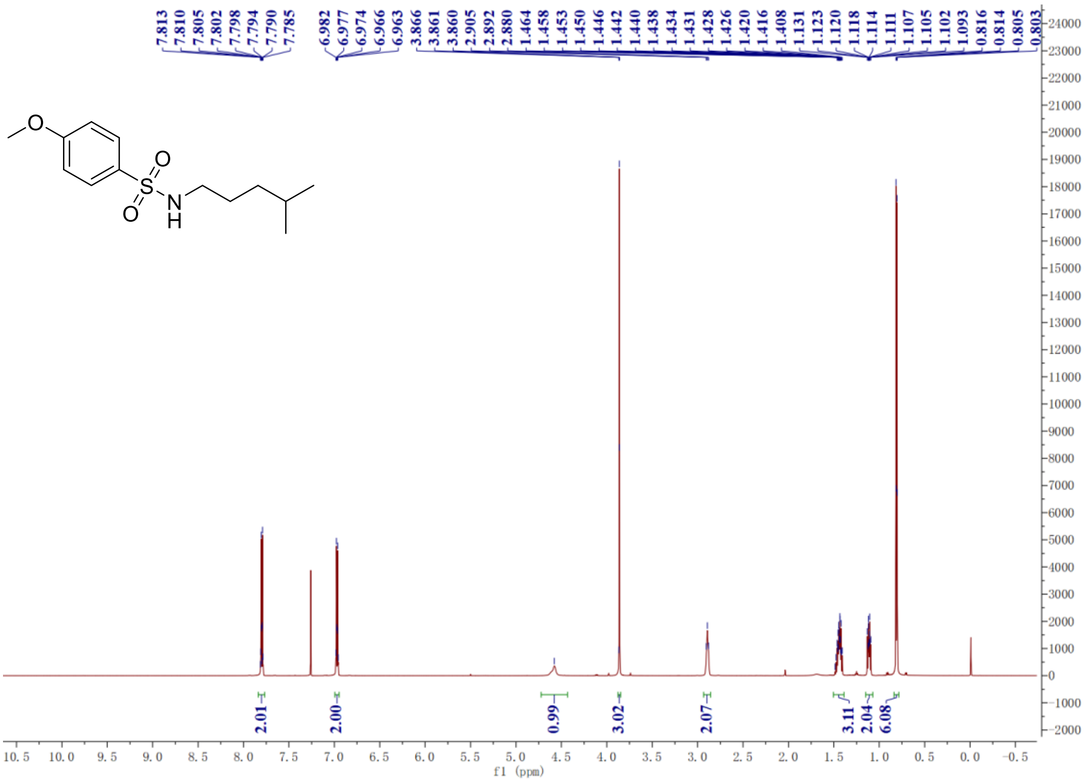

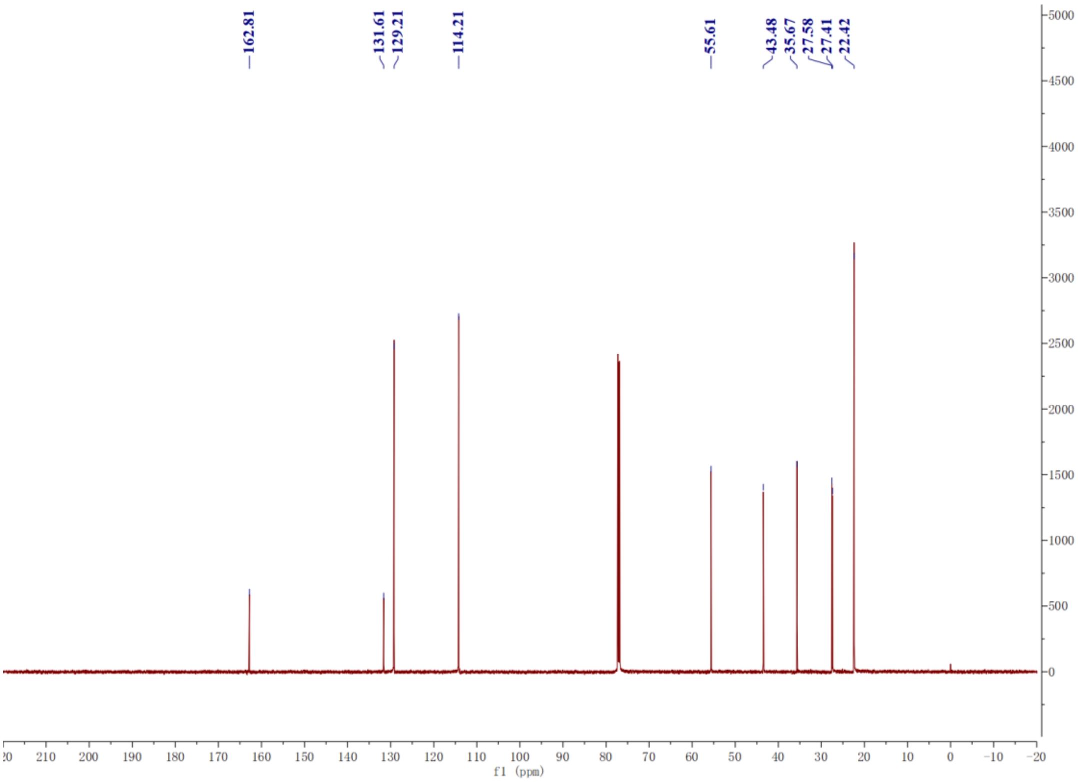

***N*-butyl-4-methoxybenzenesulfonamide (2w)**

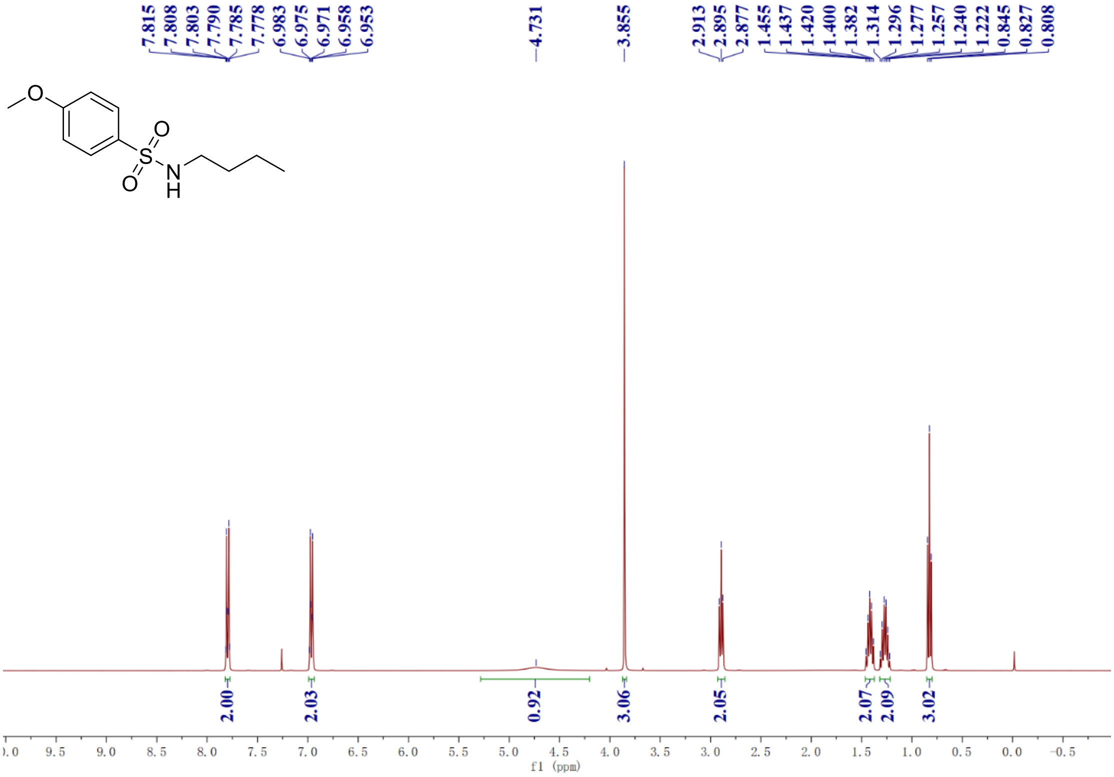

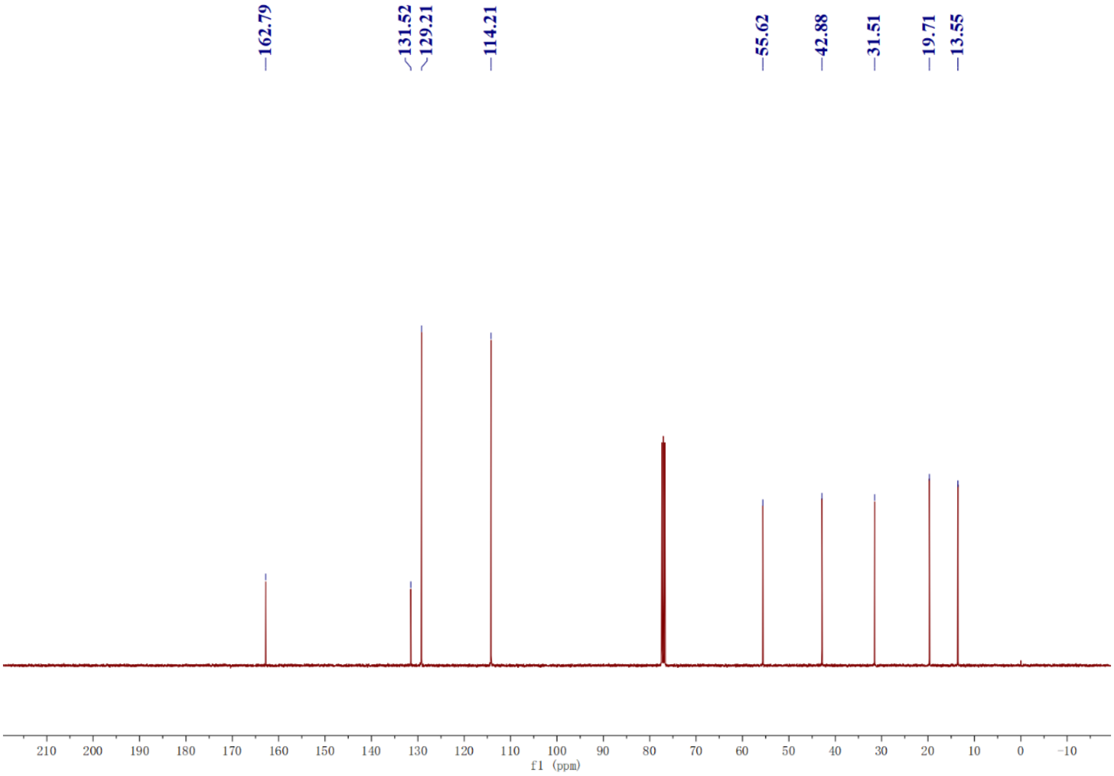

N-isopentyl-4-methoxybenzenesulfonamide (2x)

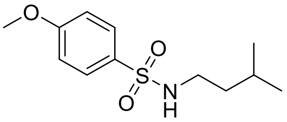

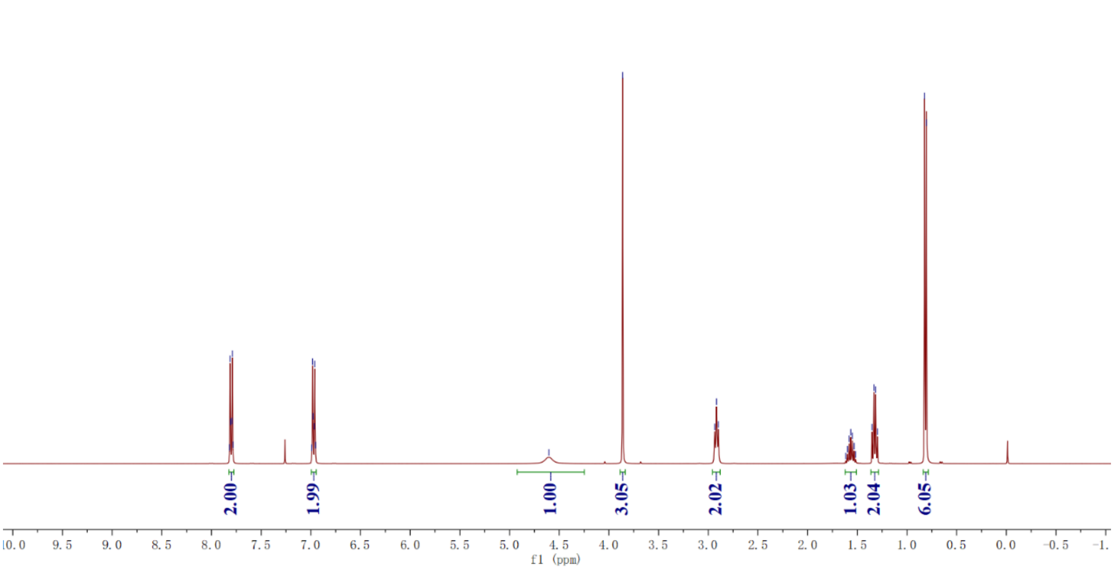

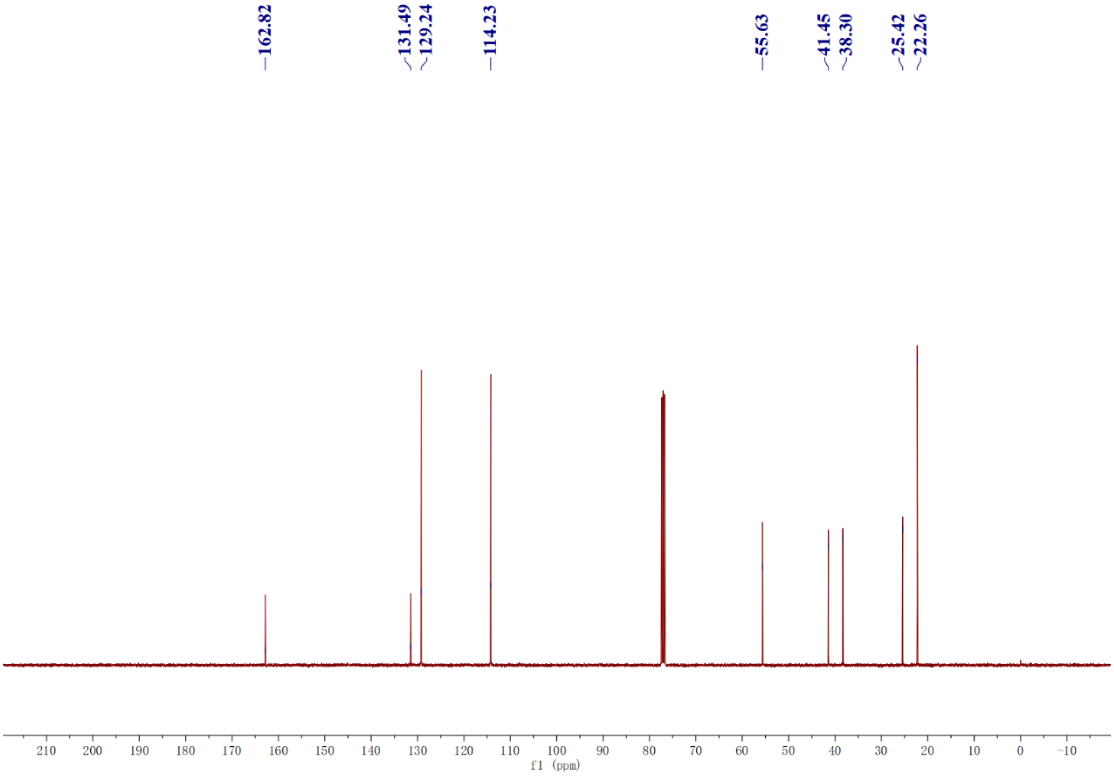

4-methoxy-*N*-methyl-*N*-pentylbenzenesulfonamide (2y)

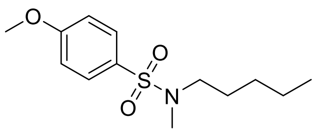

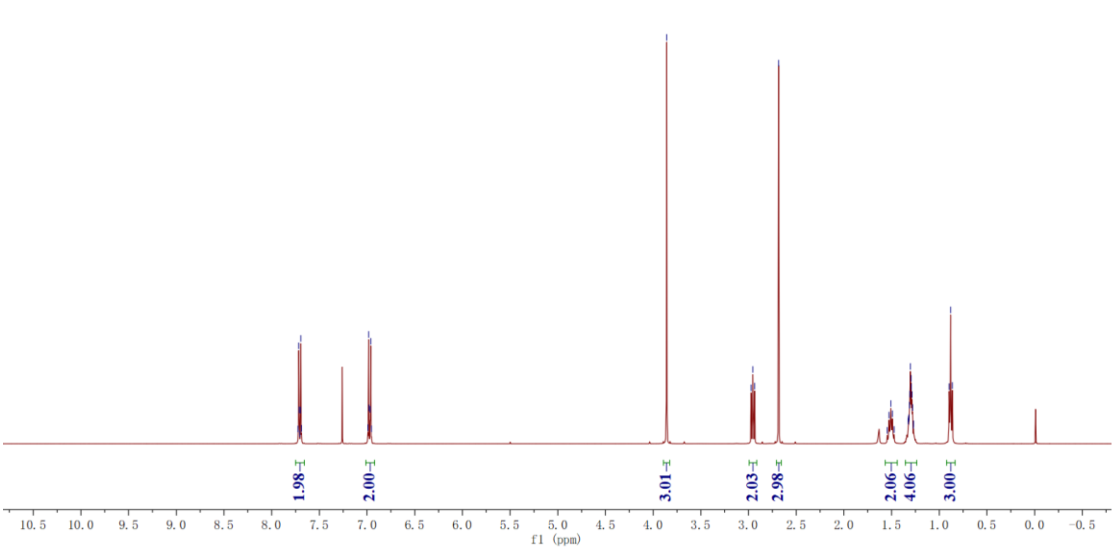

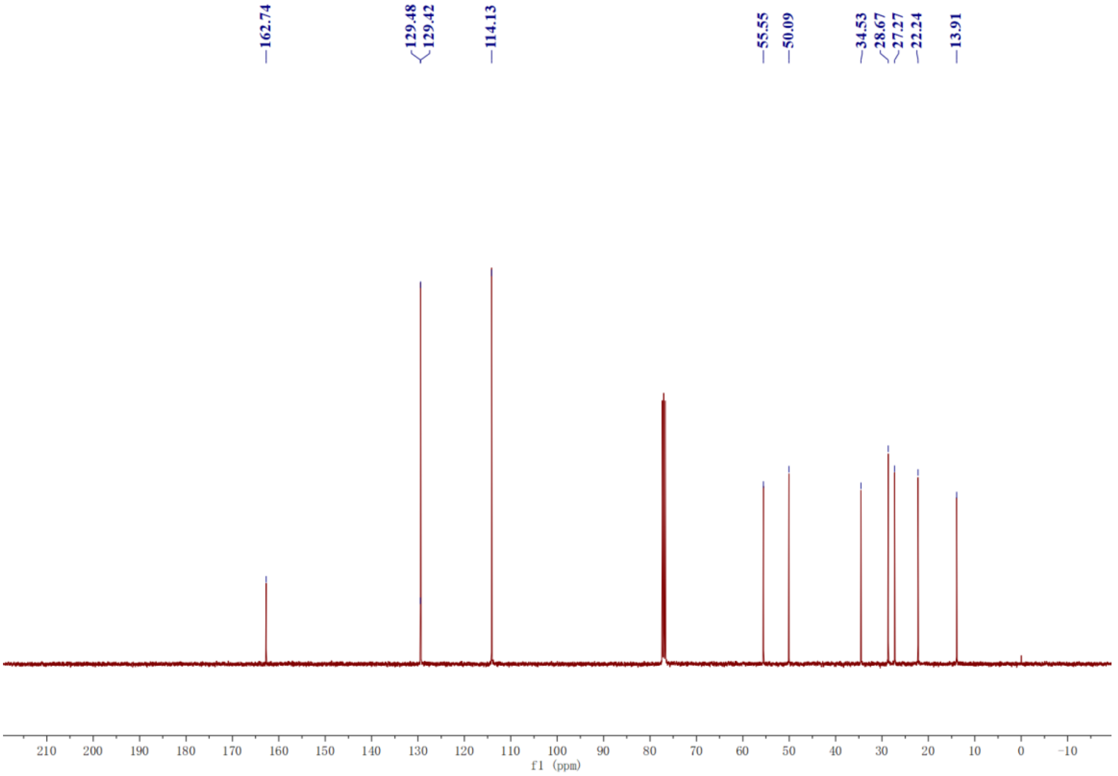

3-((4-methoxyphenyl)sulfonyl)-3-azabicyclo[3.1.0]hexane (2z)

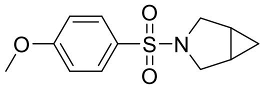

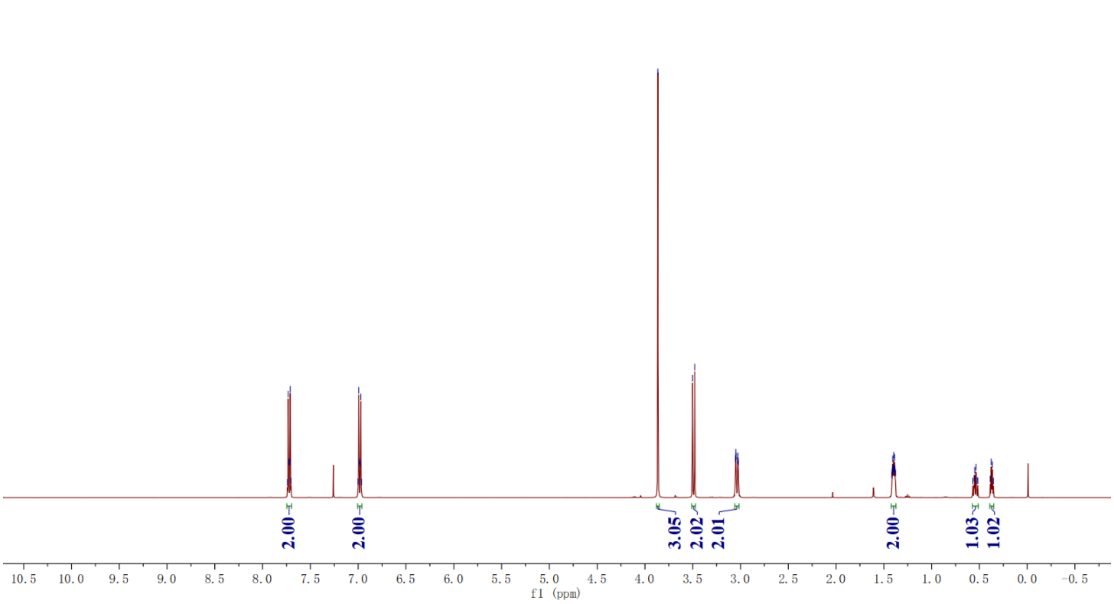

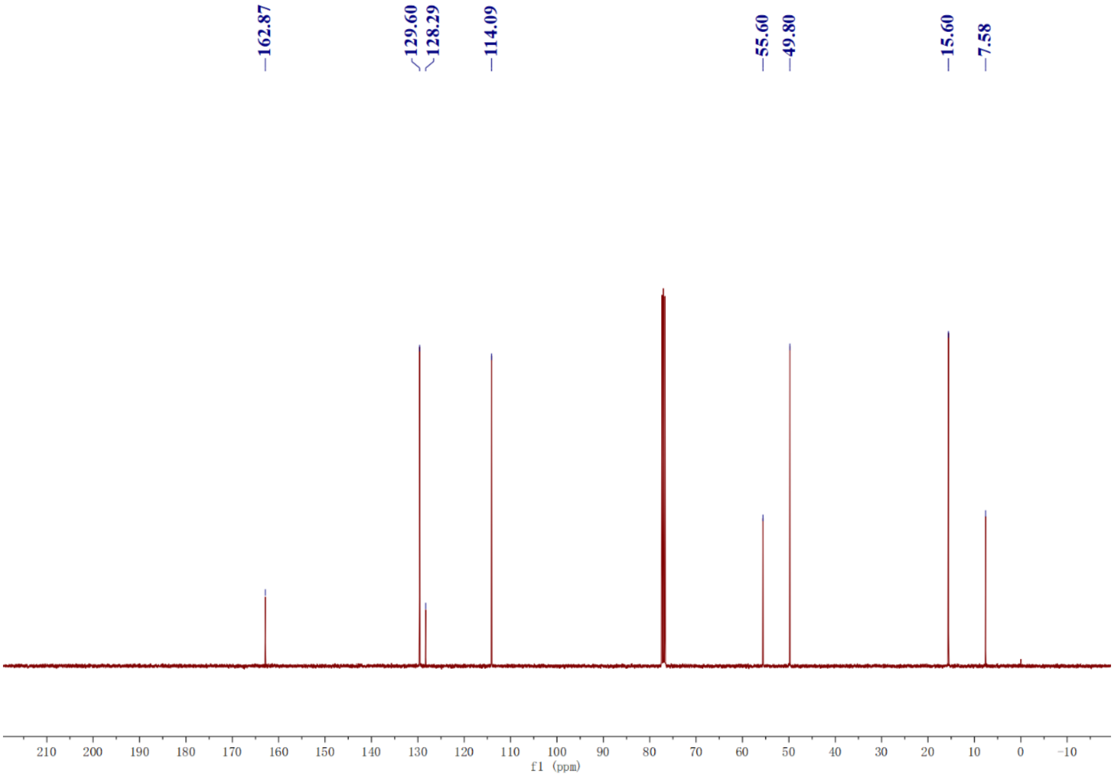

(2*R*)-1,7,7-trimethylbicyclo[2.2.1]heptan-2-yl 7-((4-methoxyphenyl)sulfonamido)heptanoate (2aa)

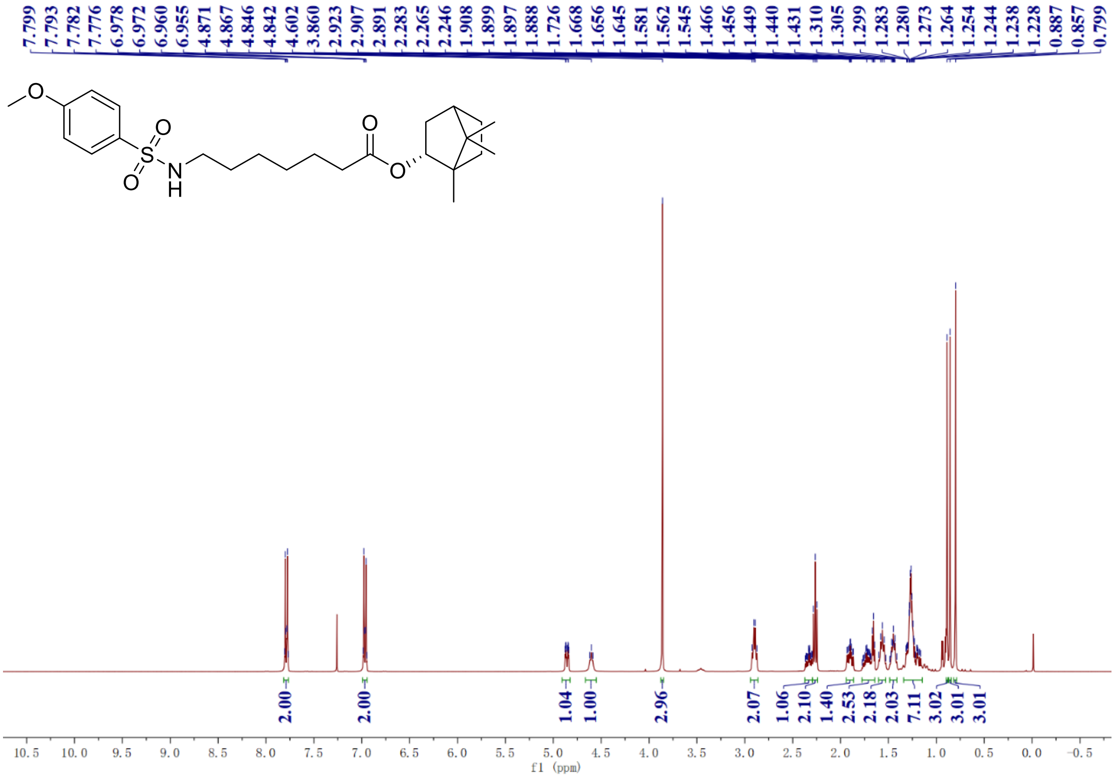

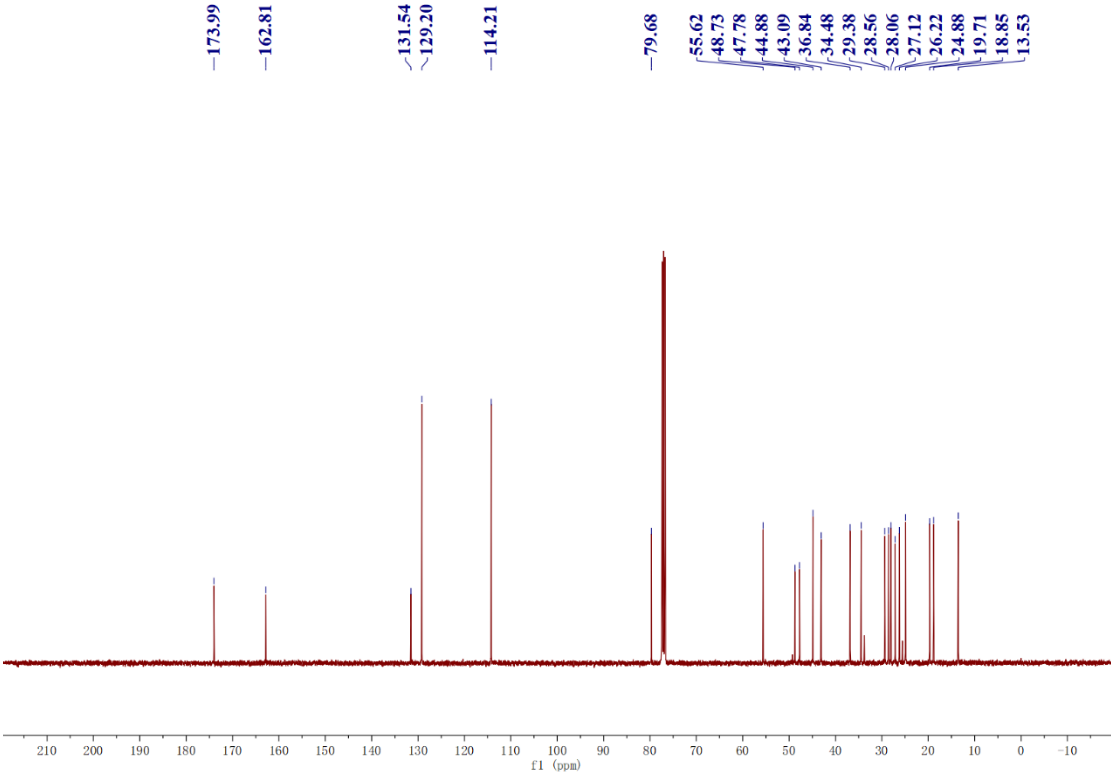

(2*S*,5*R*)-2-isopropyl-5-methylcyclohexyl 7-((4-methoxyphenyl)sulfonamido)heptanoate (2ab)

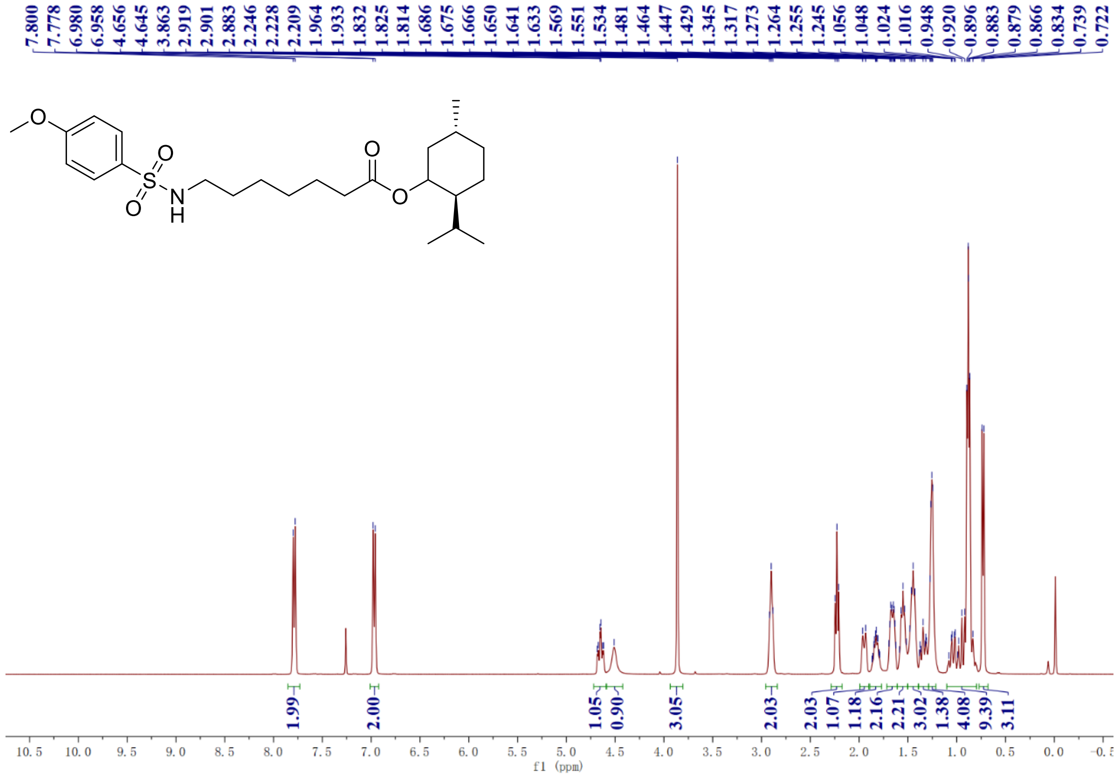

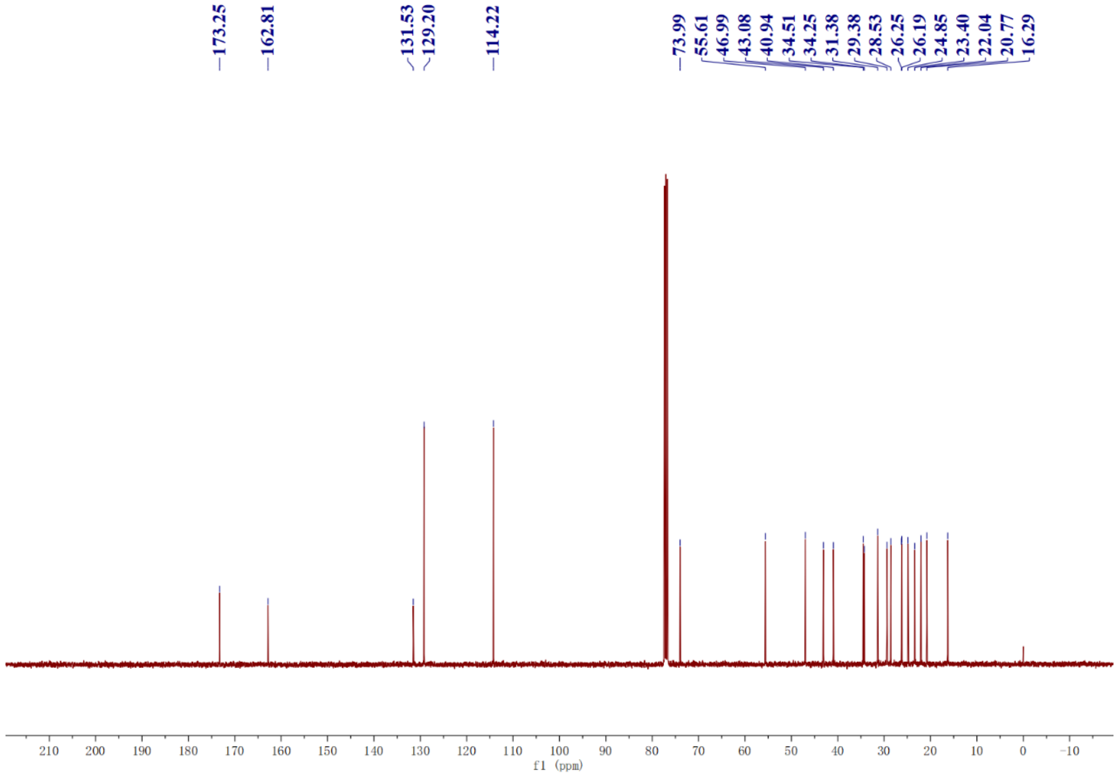

((3a*S*,4a*R*,7a*S*,8a*S*)-2,2,6,6-tetramethyltetrahydro-3a*H*-bis([1,3]dioxolo)[4,5-*b*:4',5'-*e*]pyran-3a-yl)methyl 7-((4-methoxyphenyl)sulfonamido)heptanoate (2ac)

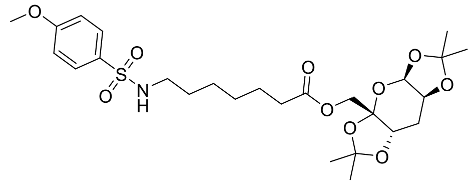

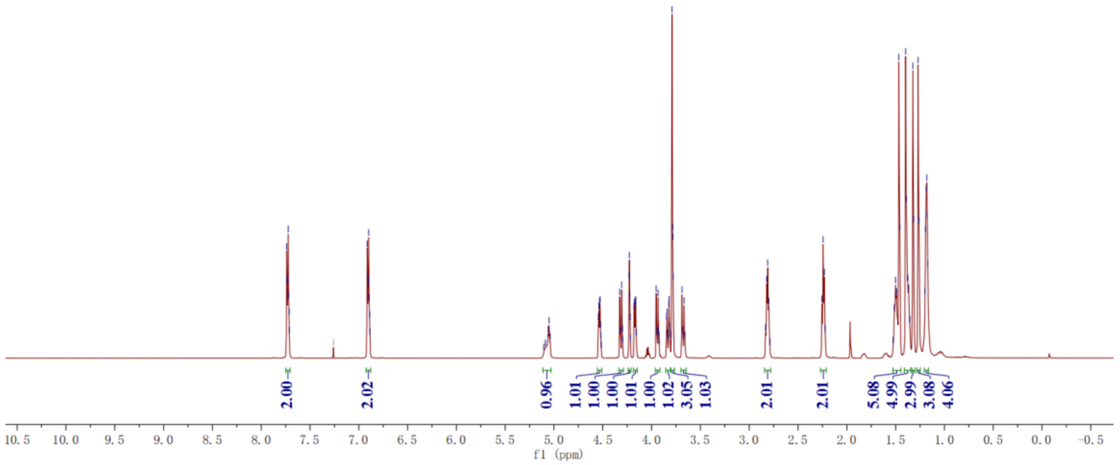

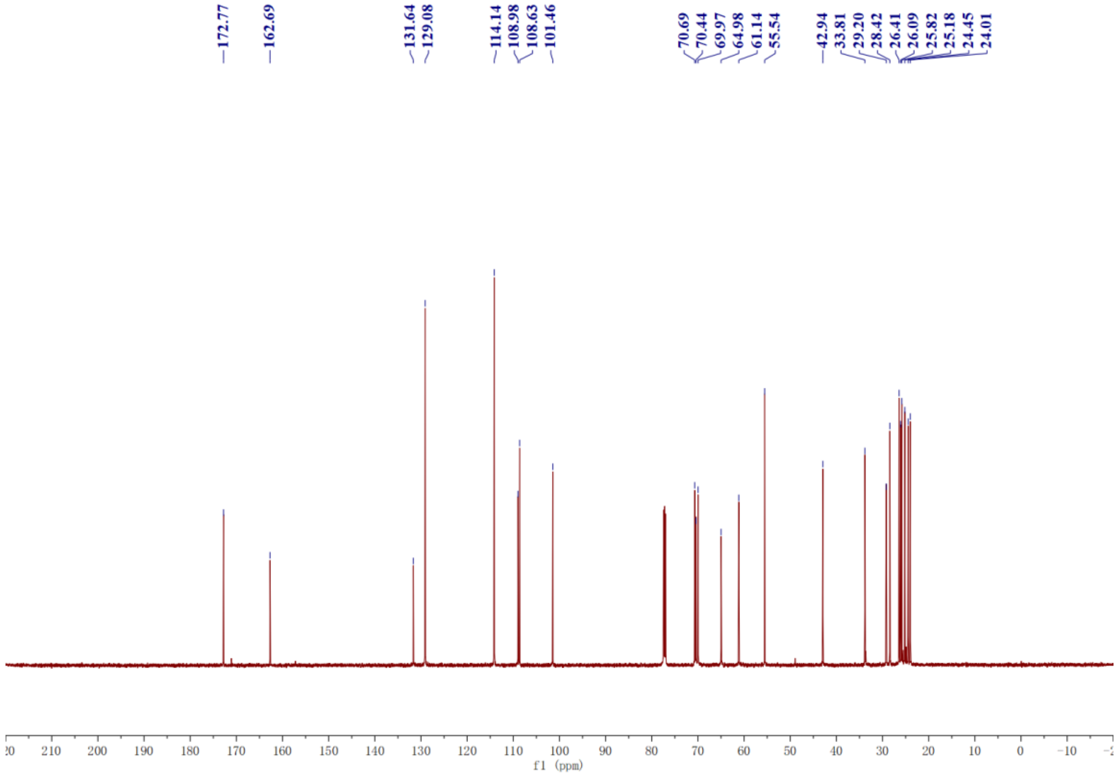

***N*-(6-(1,1-dioxido-3-oxobenzo[*d*]isothiazol-2(3*H*)-yl)hexyl)-4-methoxybenzenesulfonamide (2ad)**

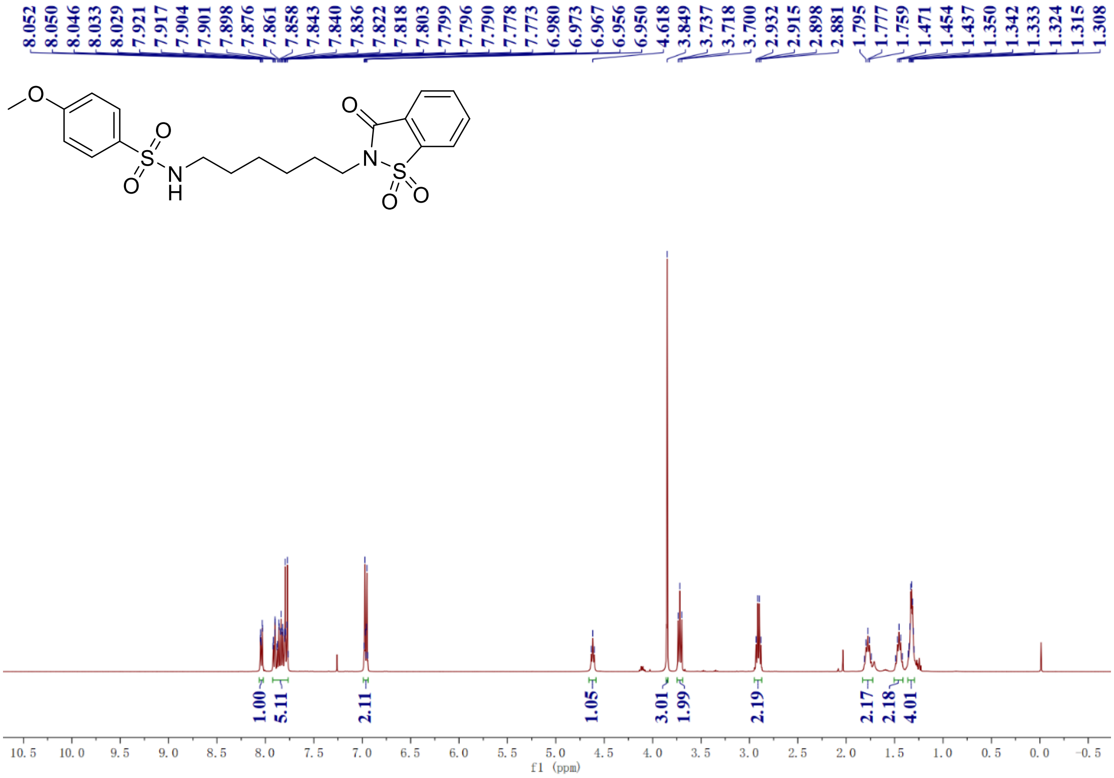

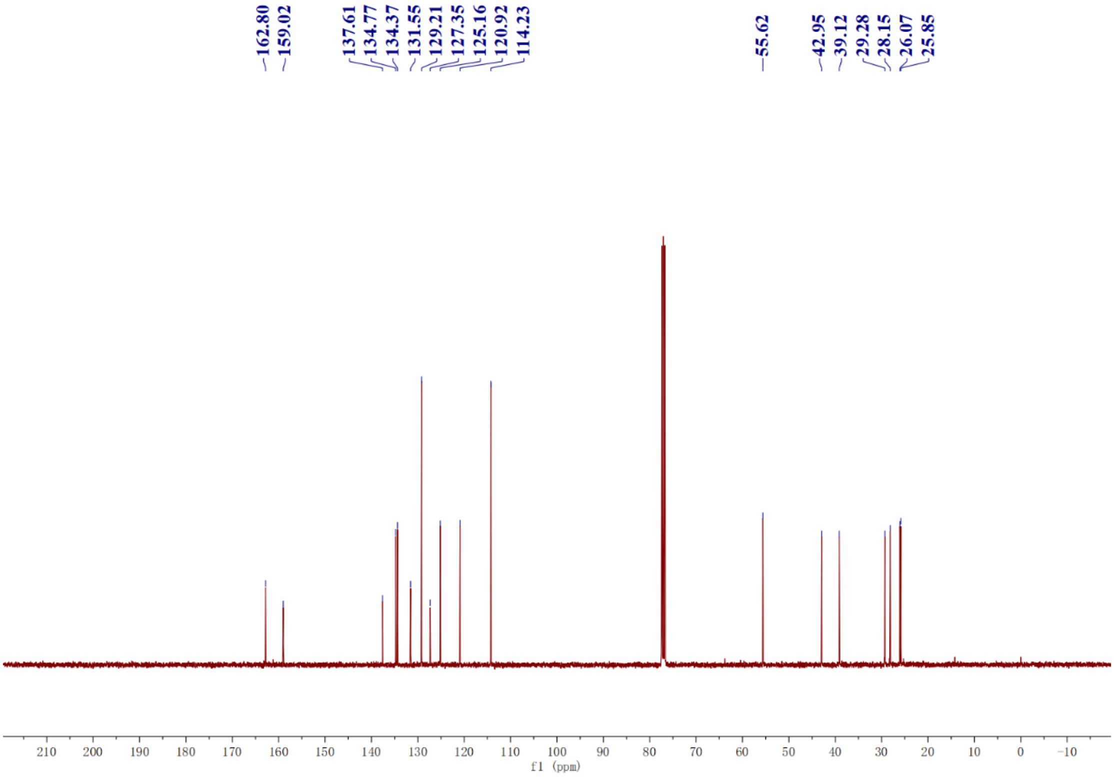

6-((4-methoxyphenyl)sulfonamido)hexyl 2-(4-isobutylphenyl)propanoate (2ae)

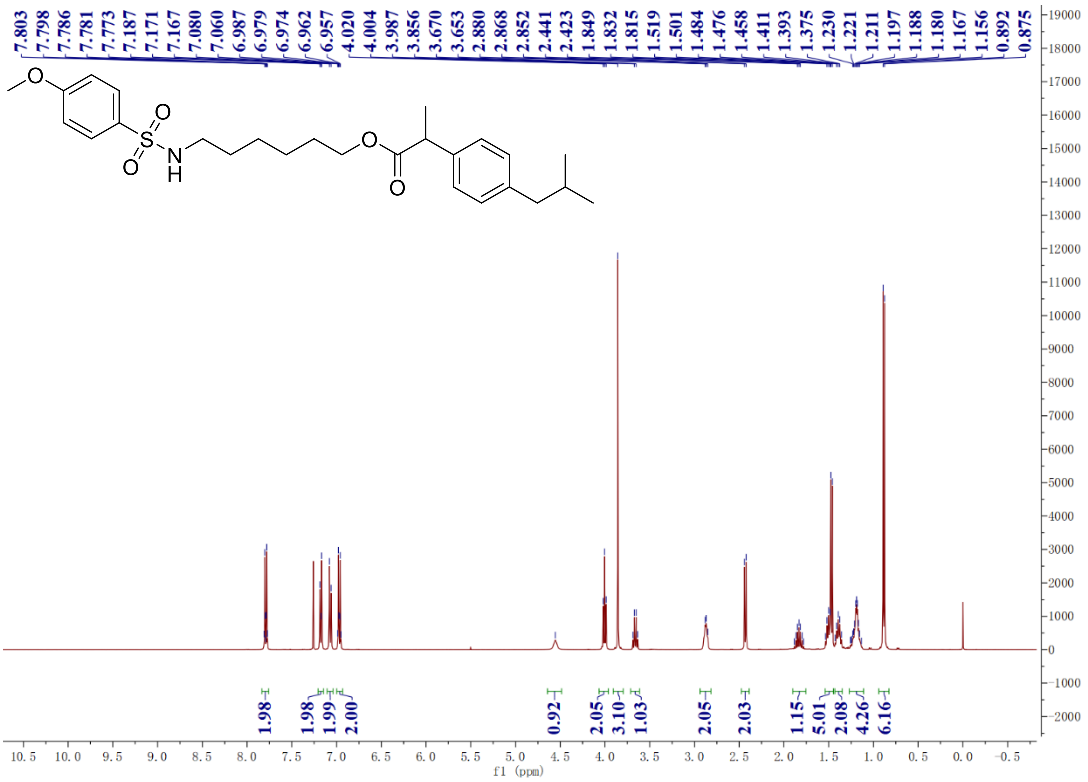

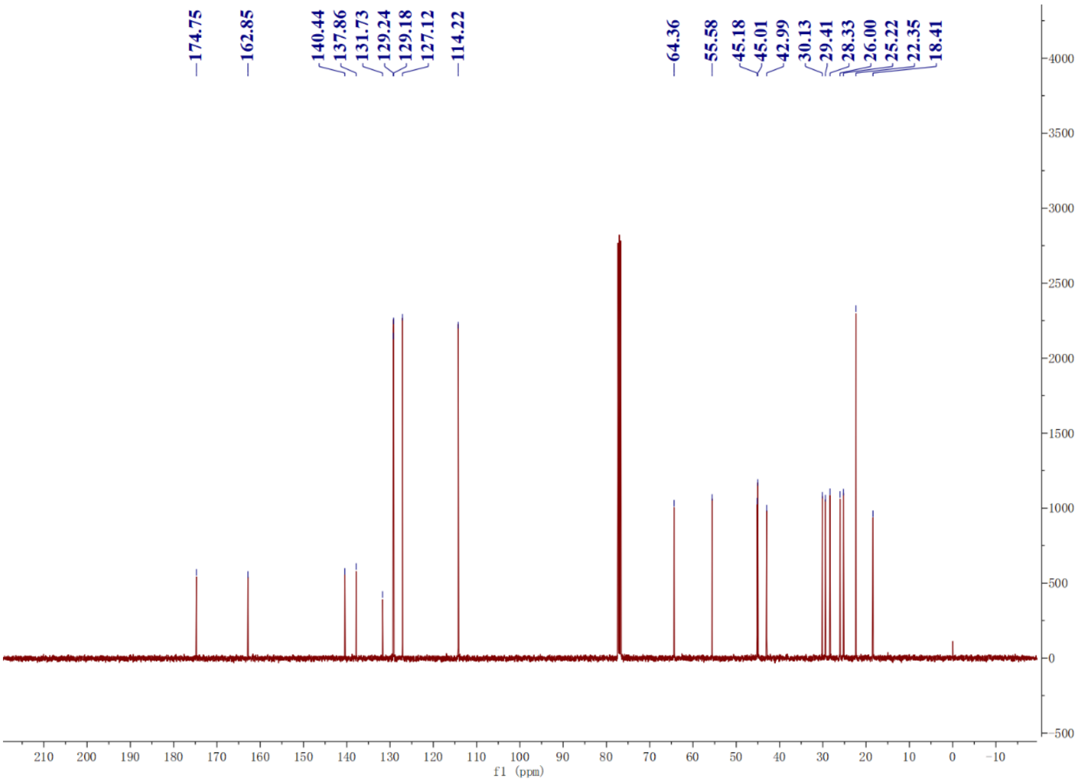

6-((4-methoxyphenyl)sulfonamido)hexyl 4-([1,1'-biphenyl]-4-yl)-4-oxobutanoate (2af)

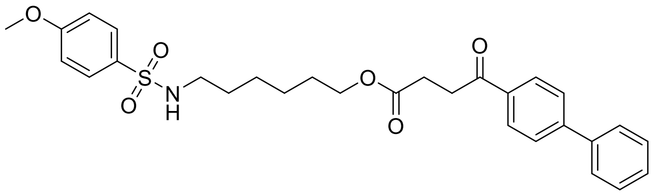

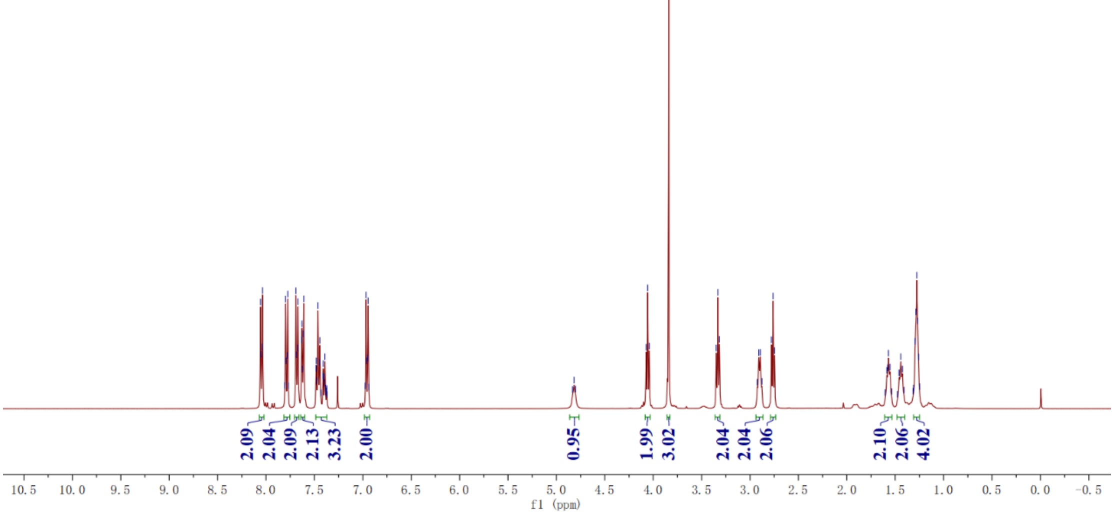

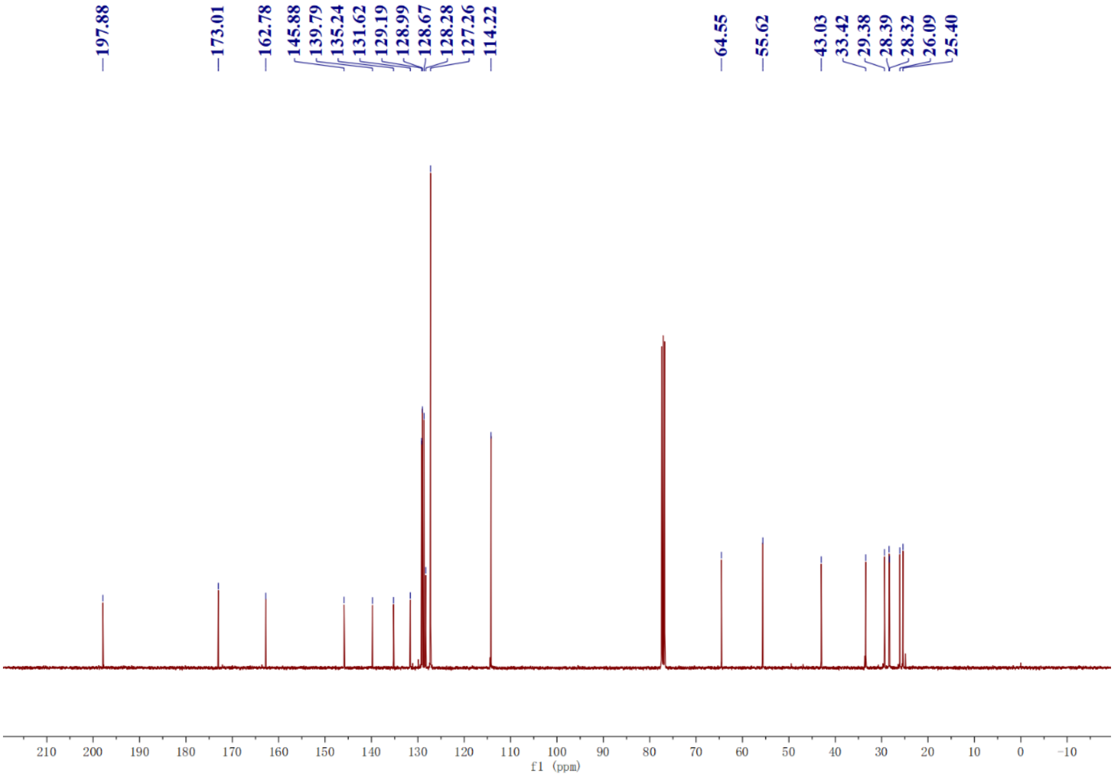

2-oxo-1-phenyl-2-((3,3,5-trimethylcyclohexyl)oxy)ethyl 7-((4-methoxyphenyl)sulfonamido)heptanoate (2ag)

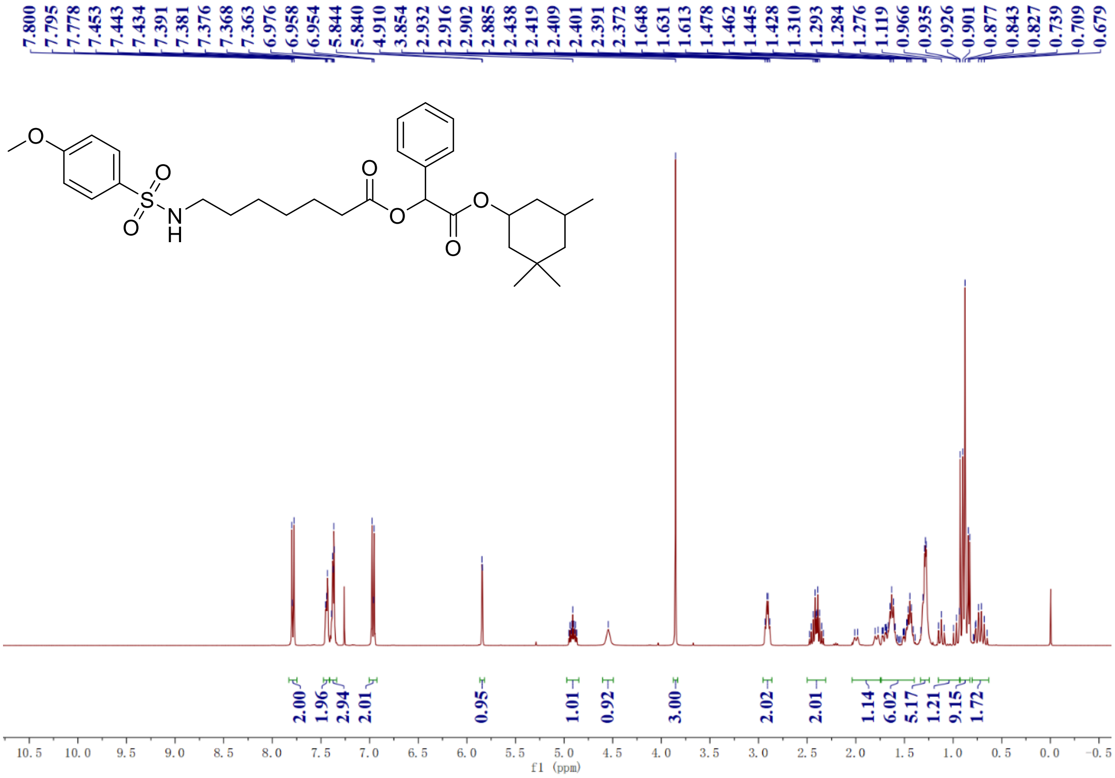

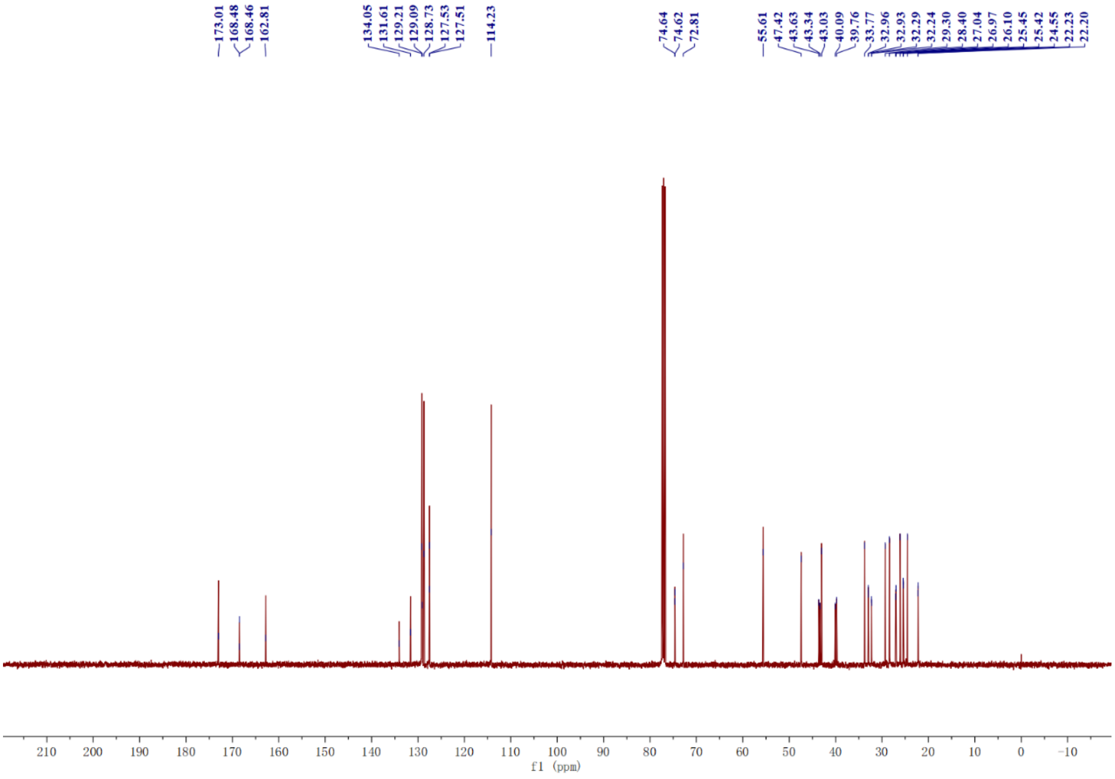

6-((4-methoxyphenyl)sulfonamido)hexyl 2-(4-(4-chlorobenzoyl)phenoxy)-2-methylpropanoate (2ah)

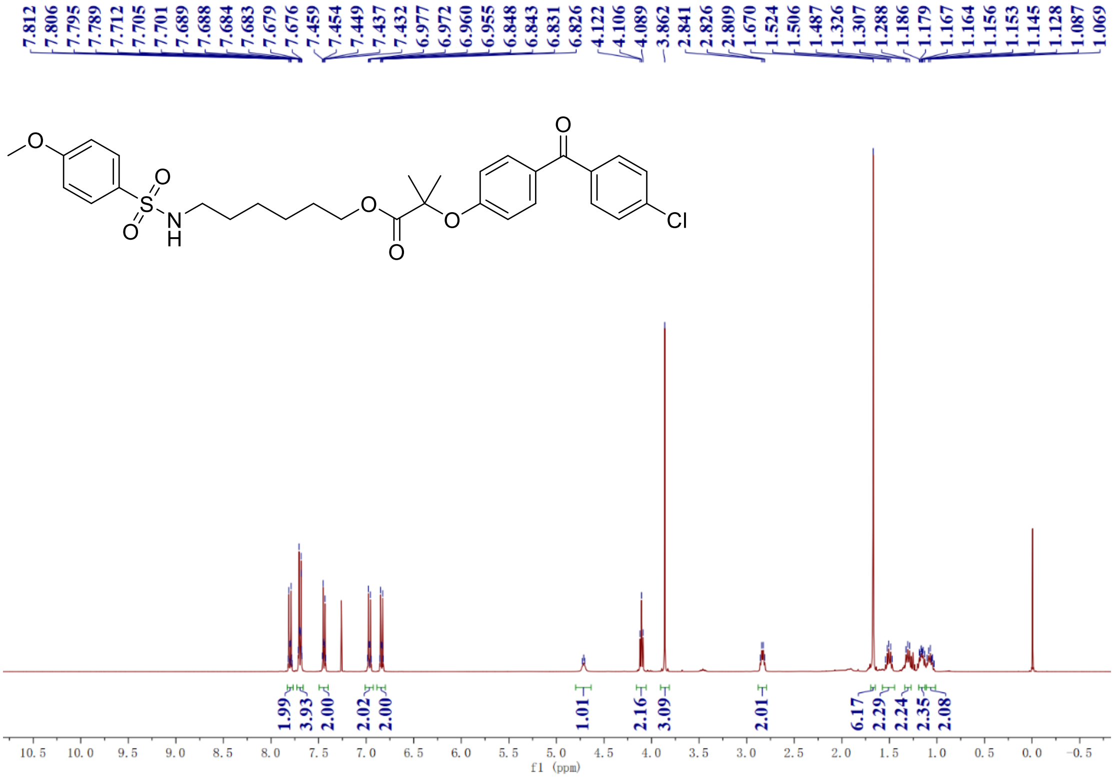

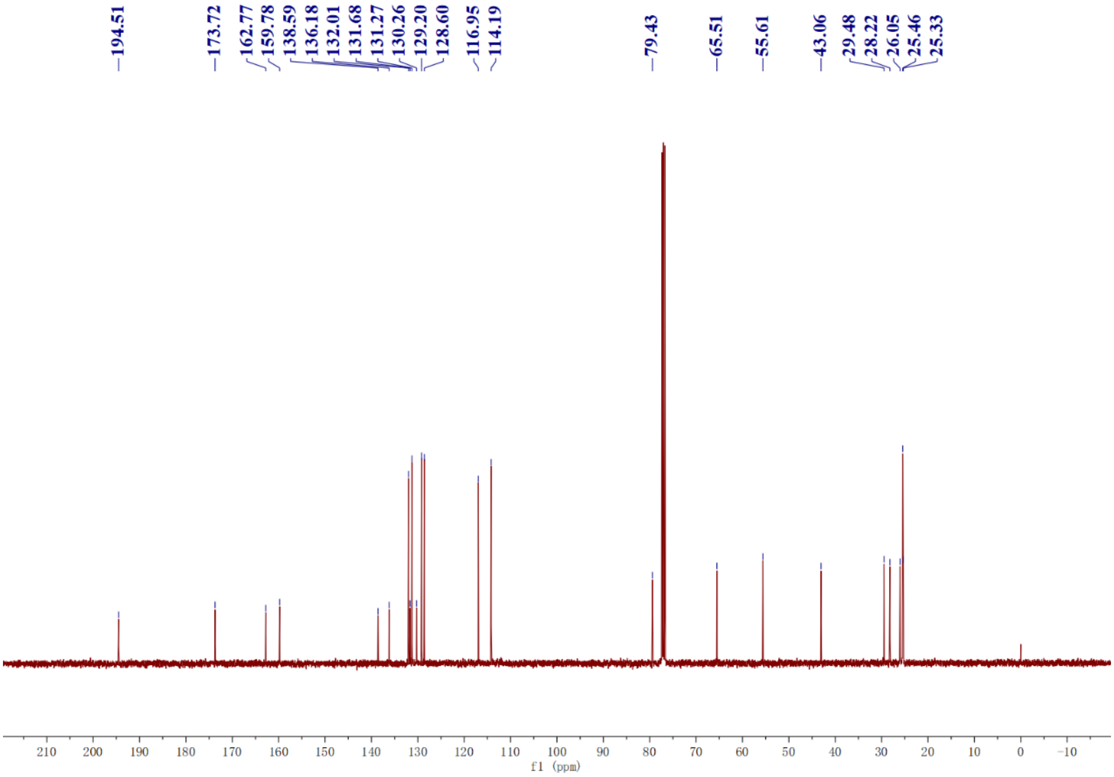

_N_-(6-((4-methoxyphenyl)sulfonamido)hexyl)-_N_-methyl-4-(5-(_p_-tolyl)-3-(trifluoromethyl)-1_H_-pyrazol-1-yl)benzenesulfonamide (2ai)

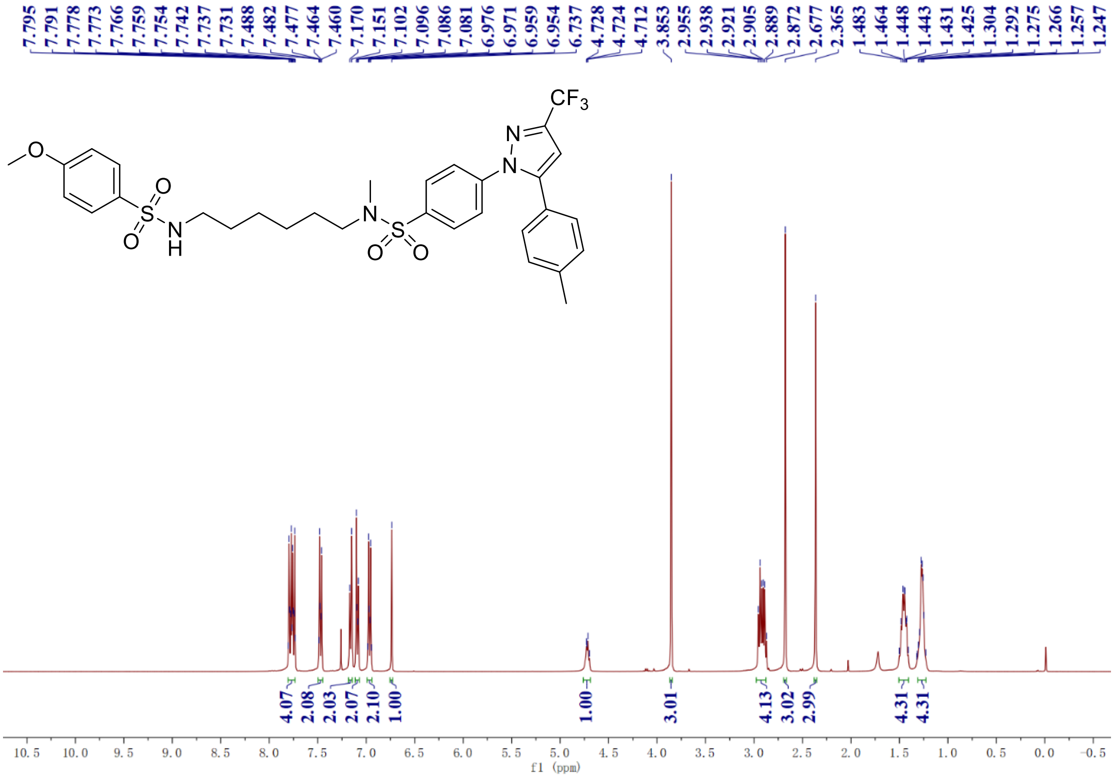

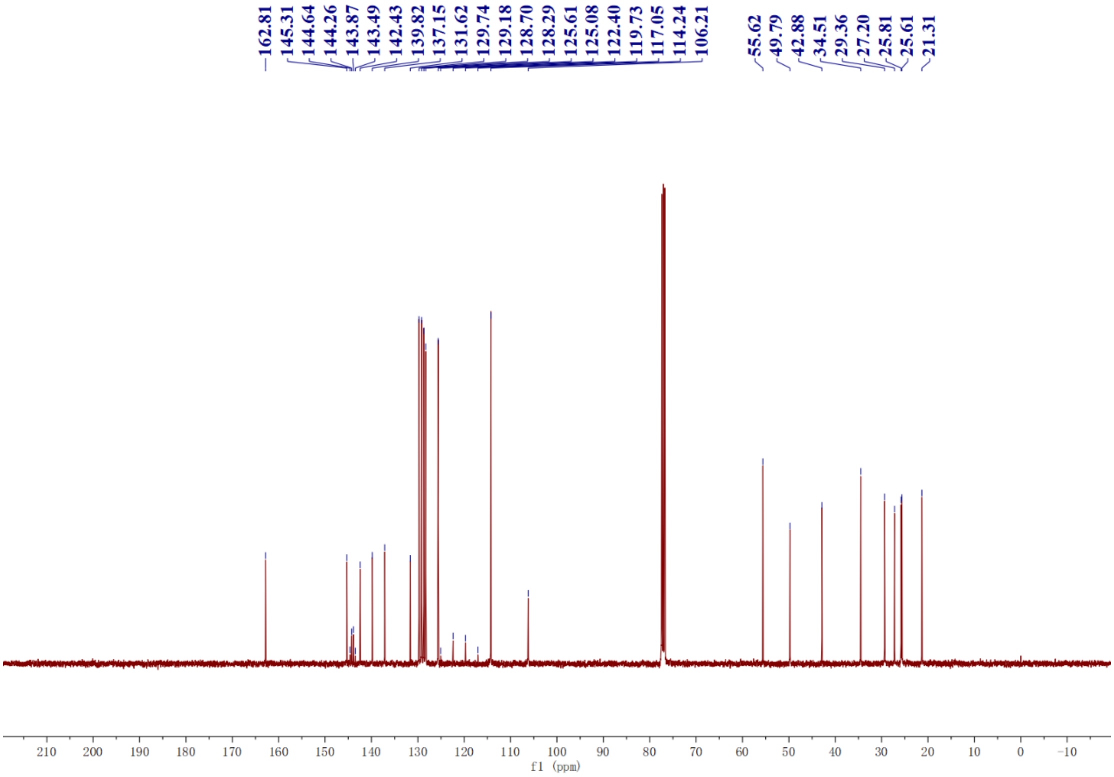

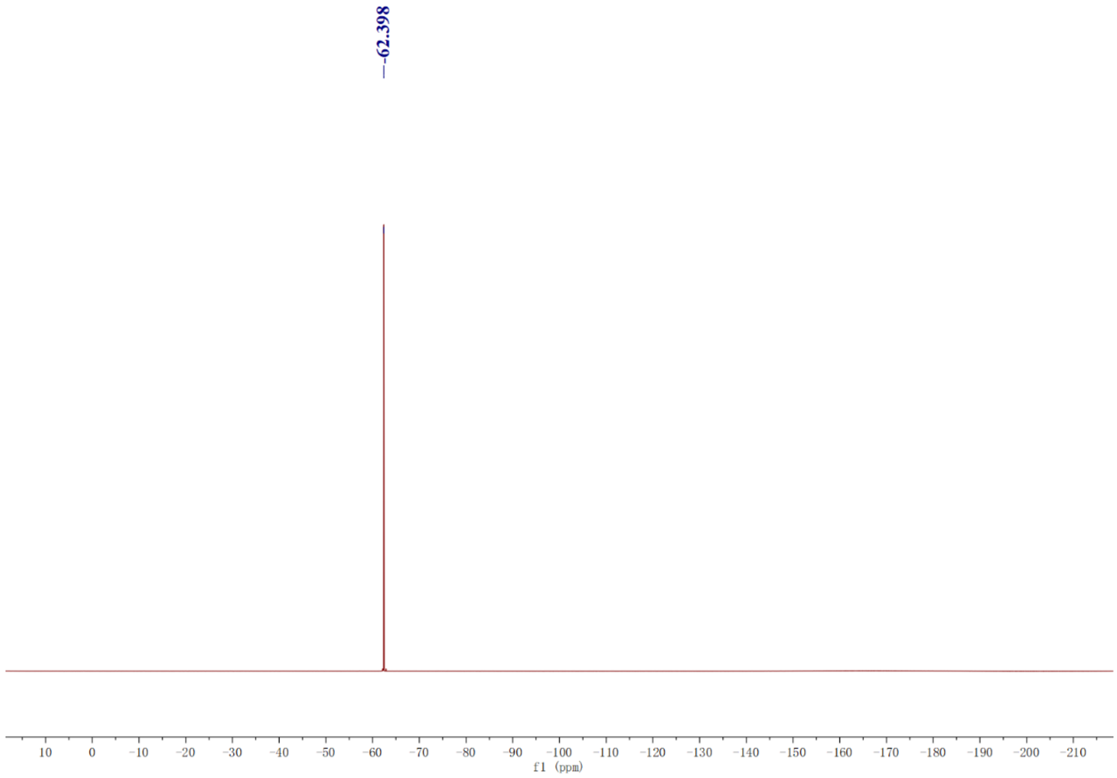
—62.398
10
0
-10
-20
-30
-40
-50
-60
-70
-80
-90
-100
-110
-120
-130
-140
-150
-160
-170
-180
-190
-200
-210
f1 (ppm)

2-(2-methyl-5-nitro-1*H*-imidazol-1-yl)ethyl 7-((4-methoxyphenyl)sulfonamido)heptanoate (2aj)

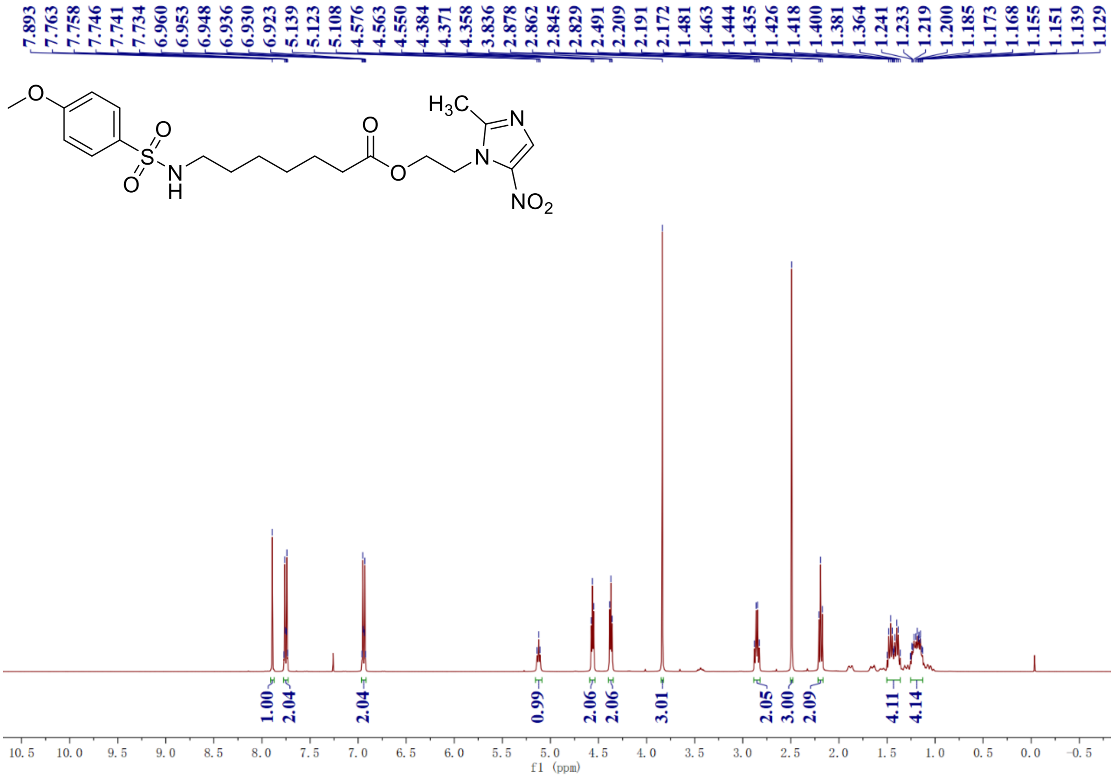

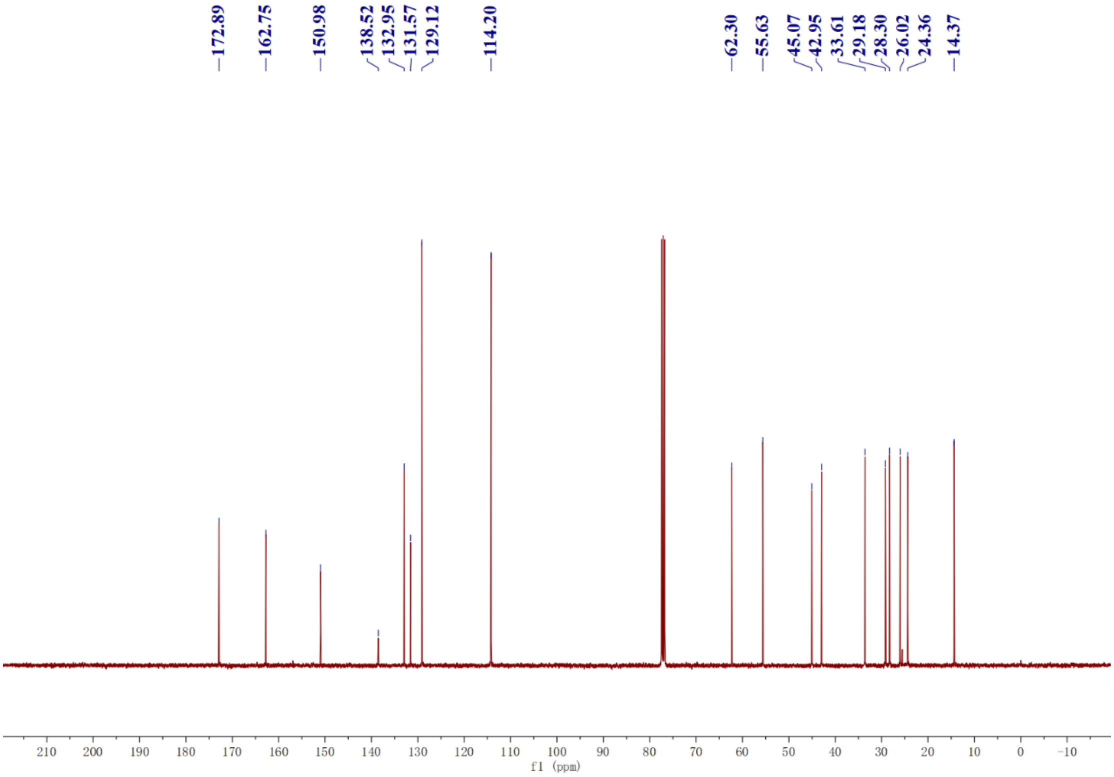

8.2 NMR spectra for products

4-methoxy-*N*-(4-(2-phenylquinolin-4-yl)pentyl)benzenesulfonamide (3)

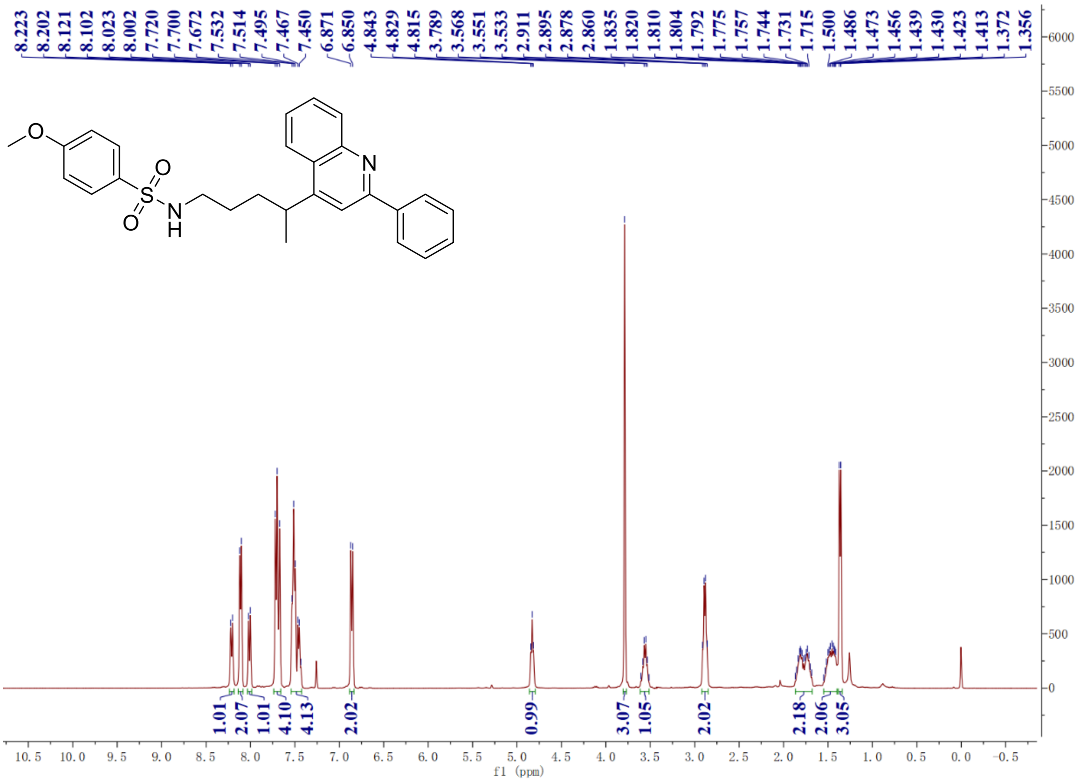

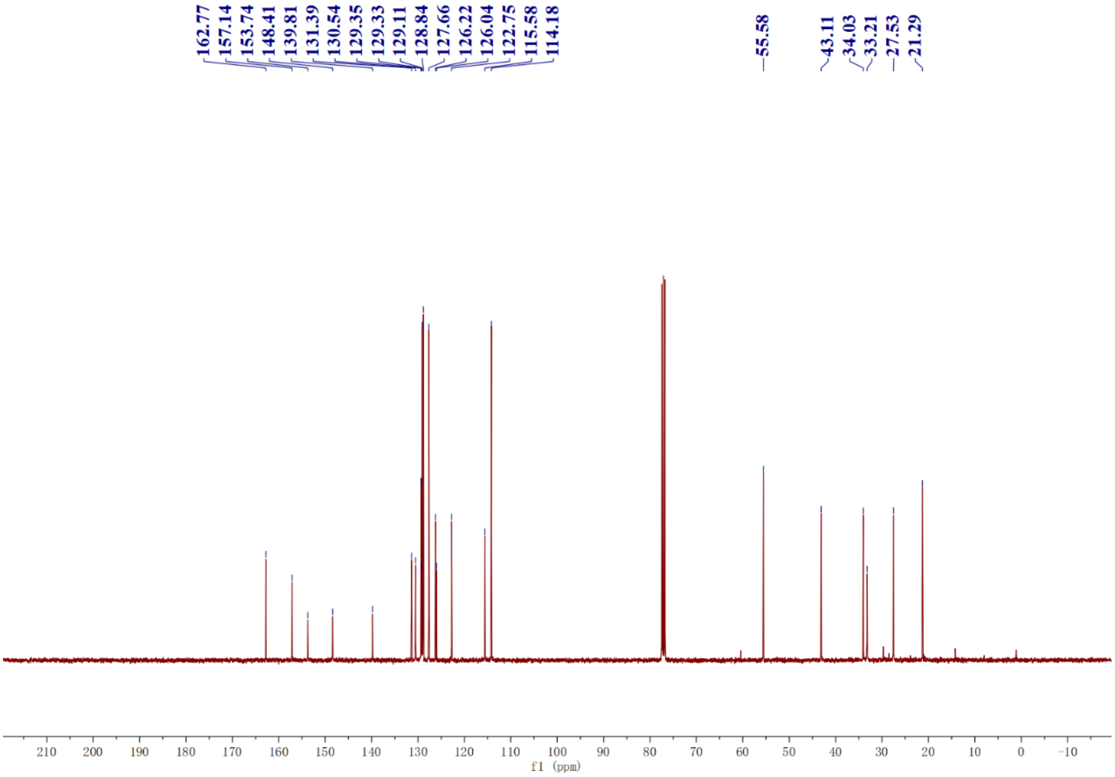

4-methoxy-*N*-(4-(2-(p-tolyl)quinolin-4-yl)pentyl)benzenesulfonamide (4)

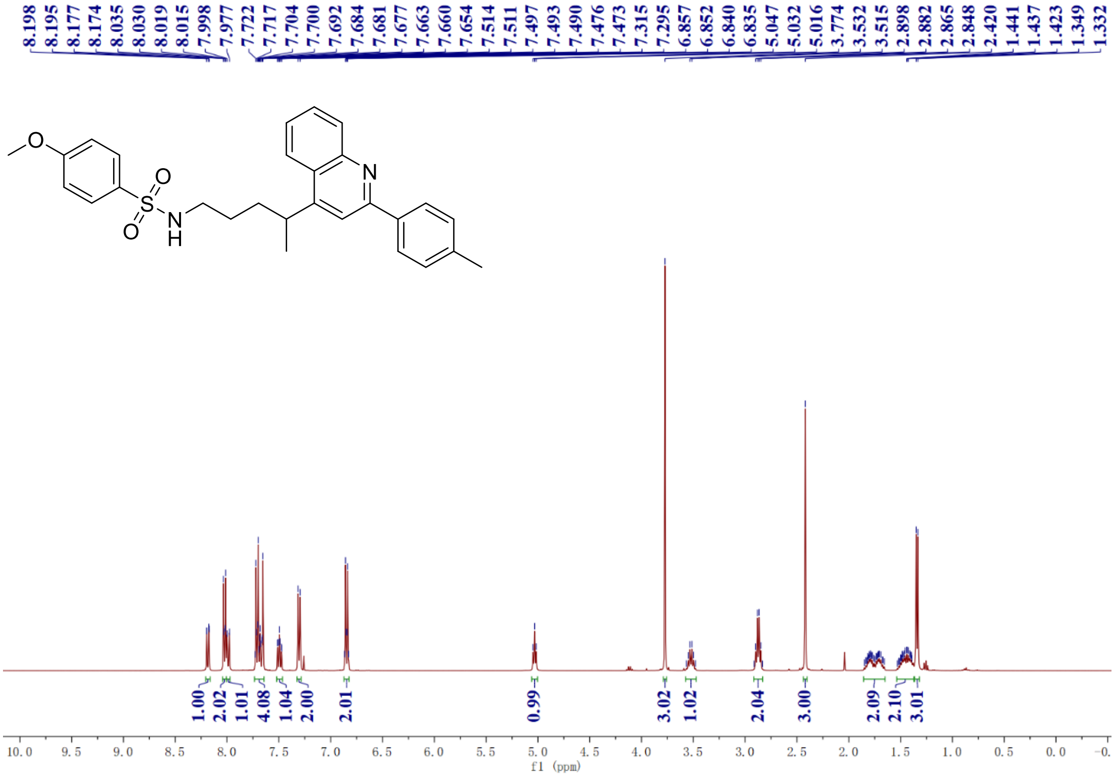

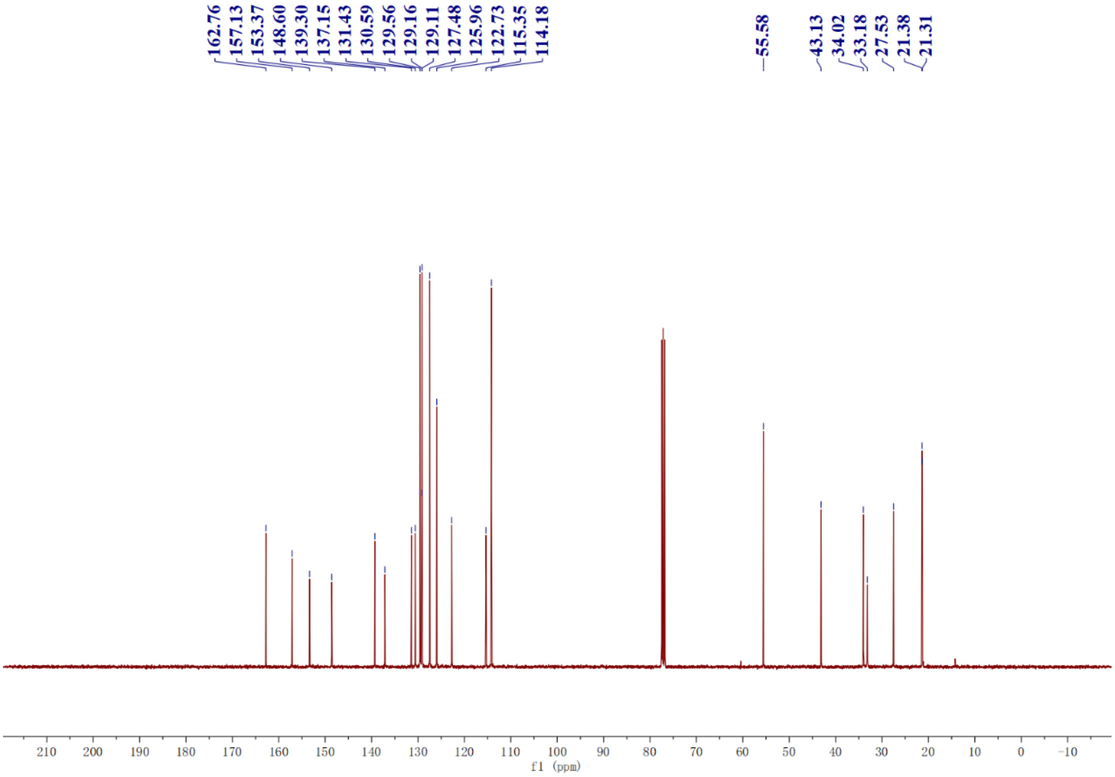

4-methoxy-*N*-(4-(2-(4-methoxyphenyl)quinolin-4-yl)pentyl)benzenesulfonamide (5)

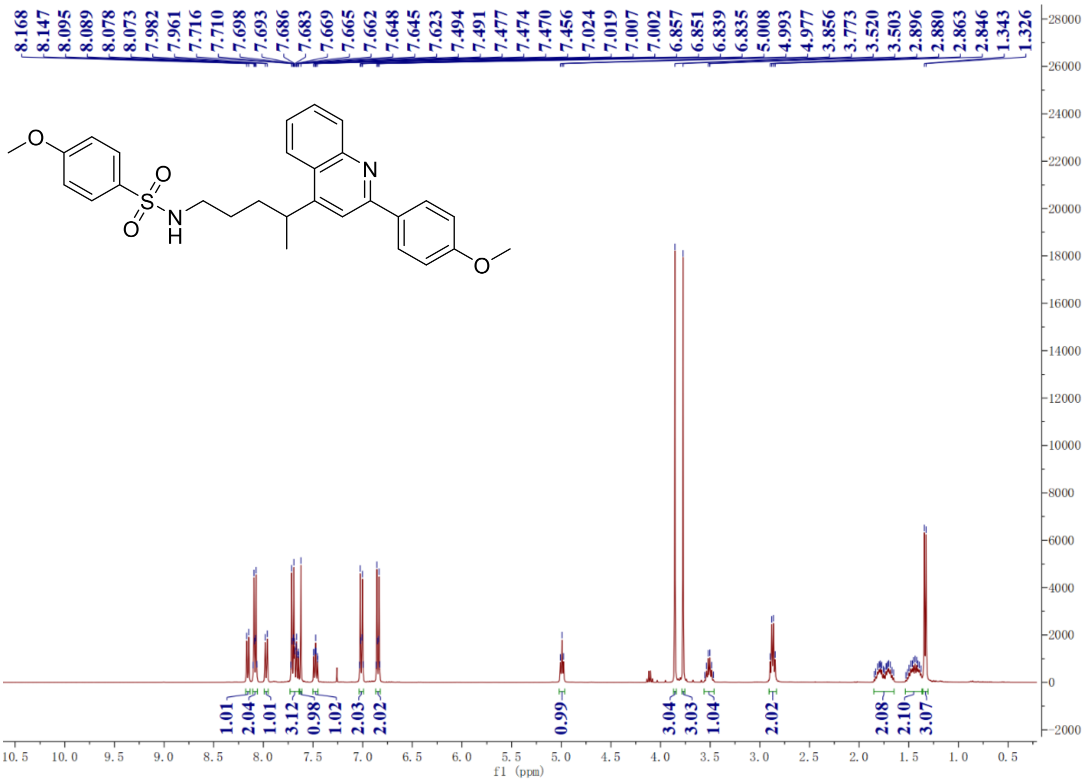

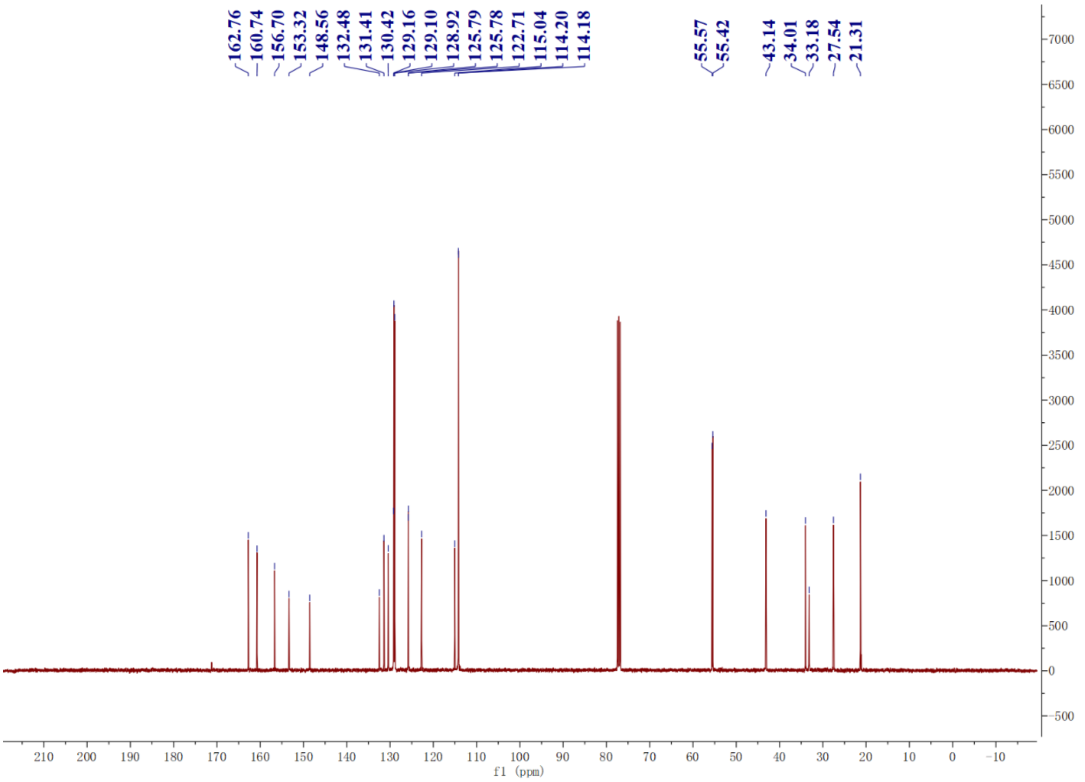

***N*-(4-(2-(4-fluorophenyl)quinolin-4-yl)pentyl)-4-methoxybenzenesulfonamide (6)**

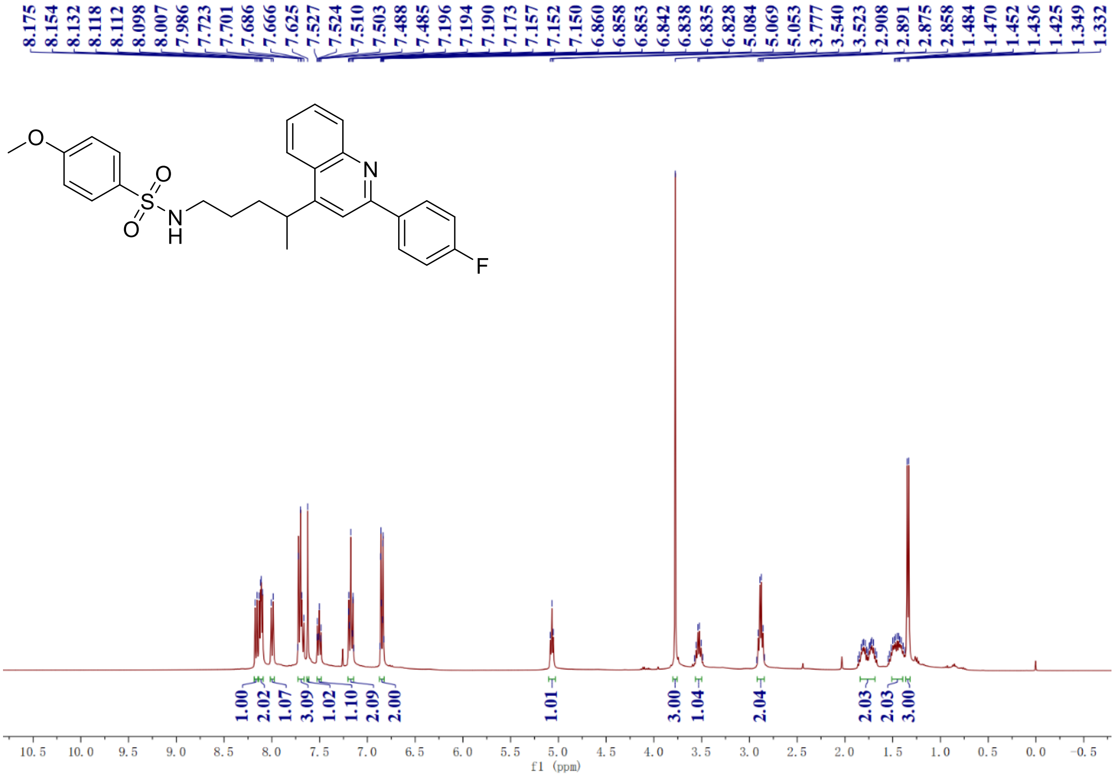

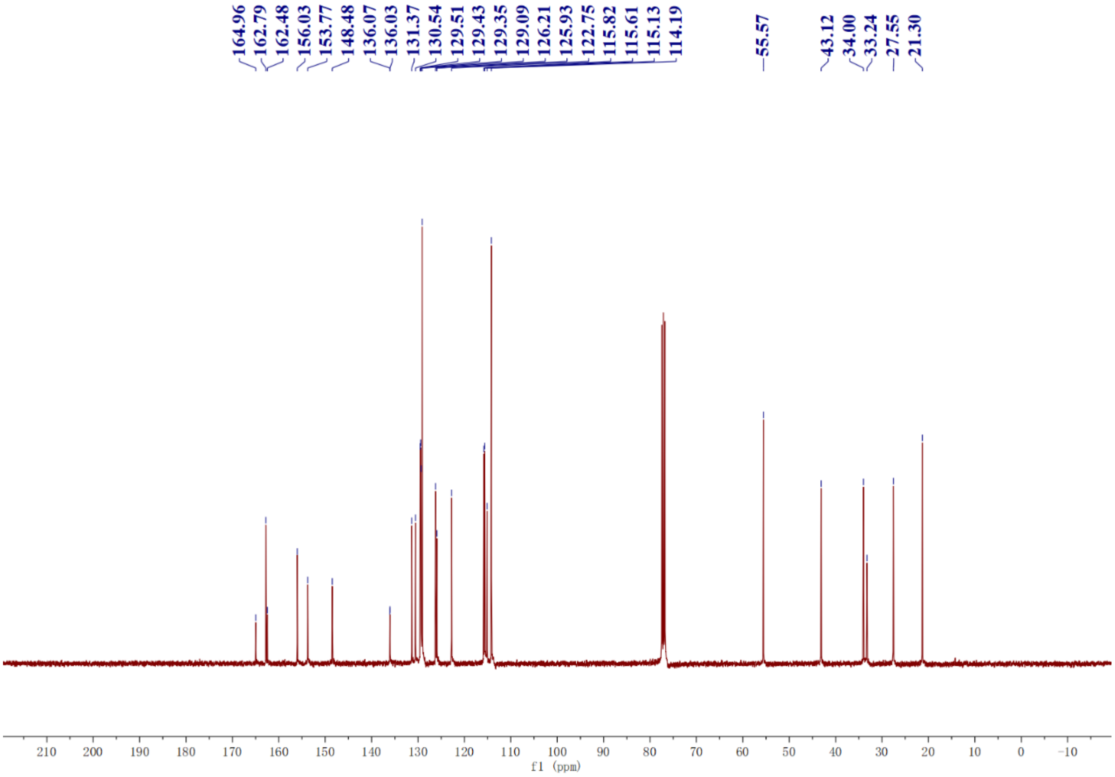

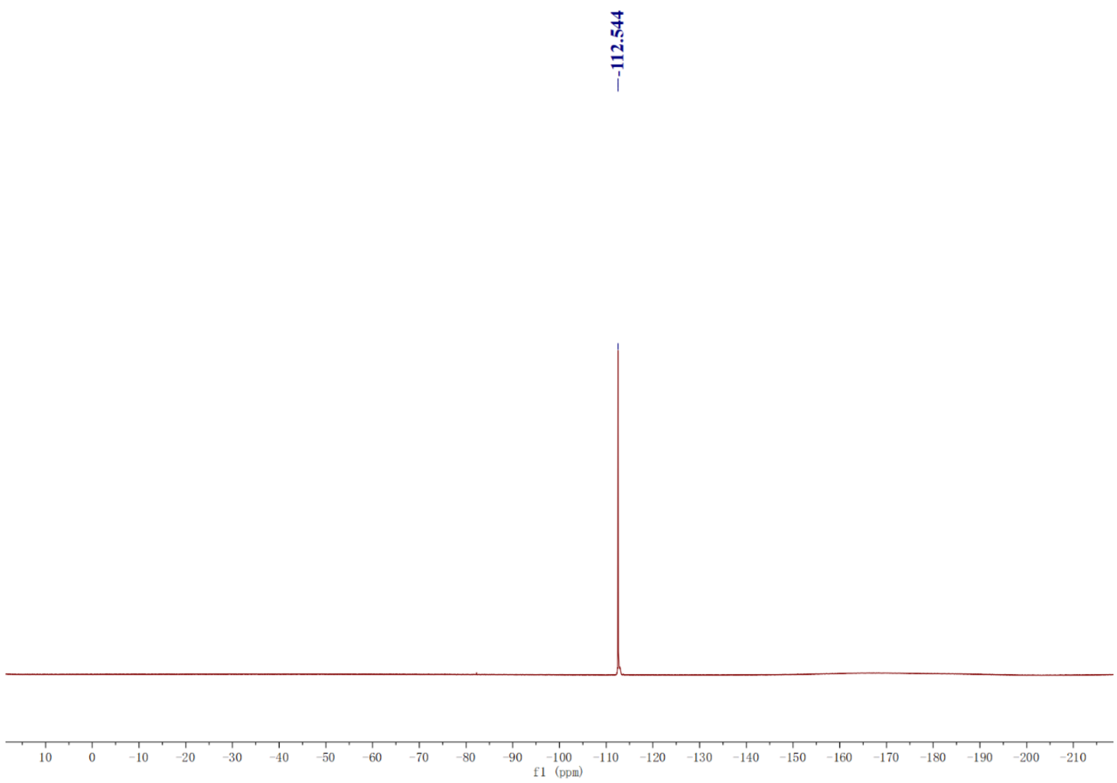

-112.544
10 0 -10 -20 -30 -40 -50 -60 -70 -80 -90 -100 -110 -120 -130 -140 -150 -160 -170 -180 -190 -200 -210
f1 (ppm)

***N*-(4-(2-(4-chlorophenyl)quinolin-4-yl)pentyl)-4-methoxybenzenesulfonamide (7)**

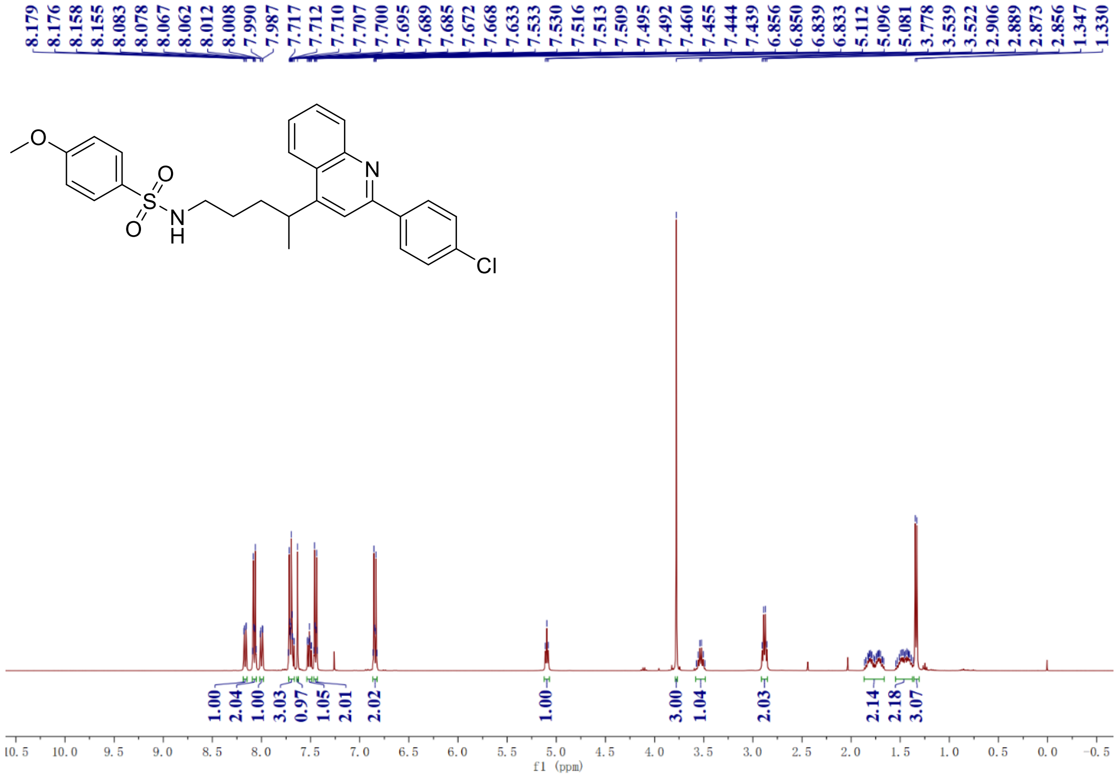

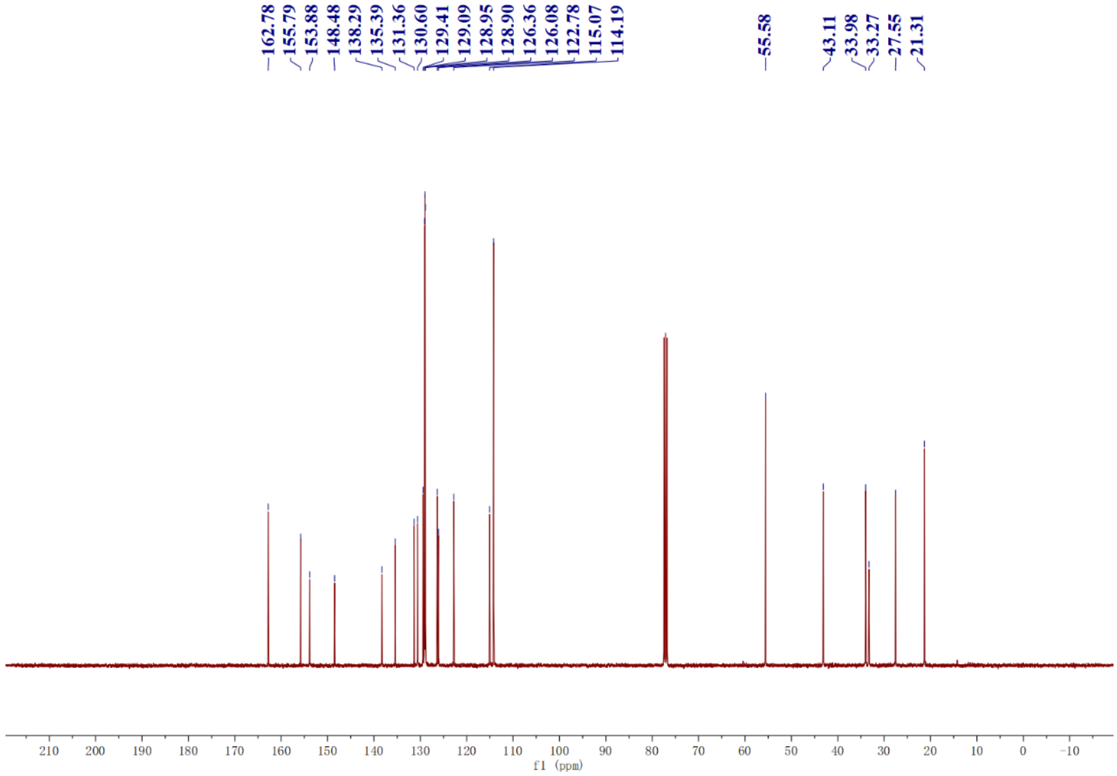

N-(4-(2-([1,1'-biphenyl]-4-yl)quinolin-4-yl)pentyl)-4-methoxybenzenesulfonamide (8)

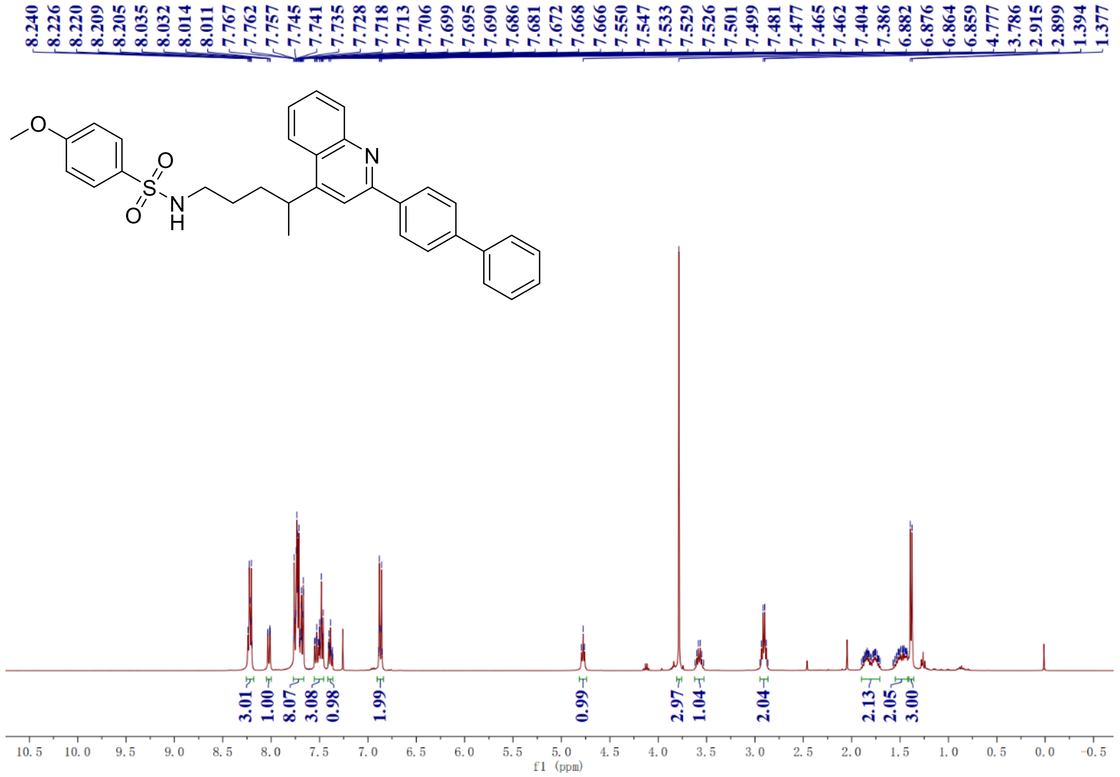

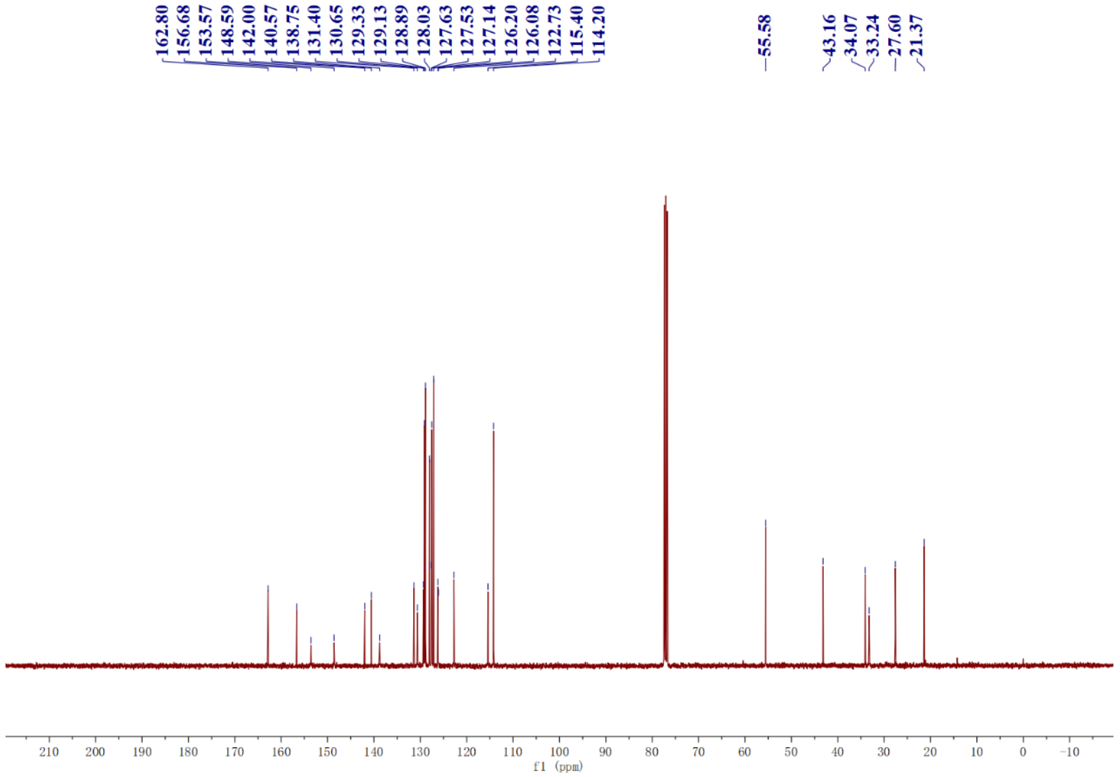

4-methoxy-*N*-(4-(4-methylquinolin-2-yl)pentyl)benzenesulfonamide (9)

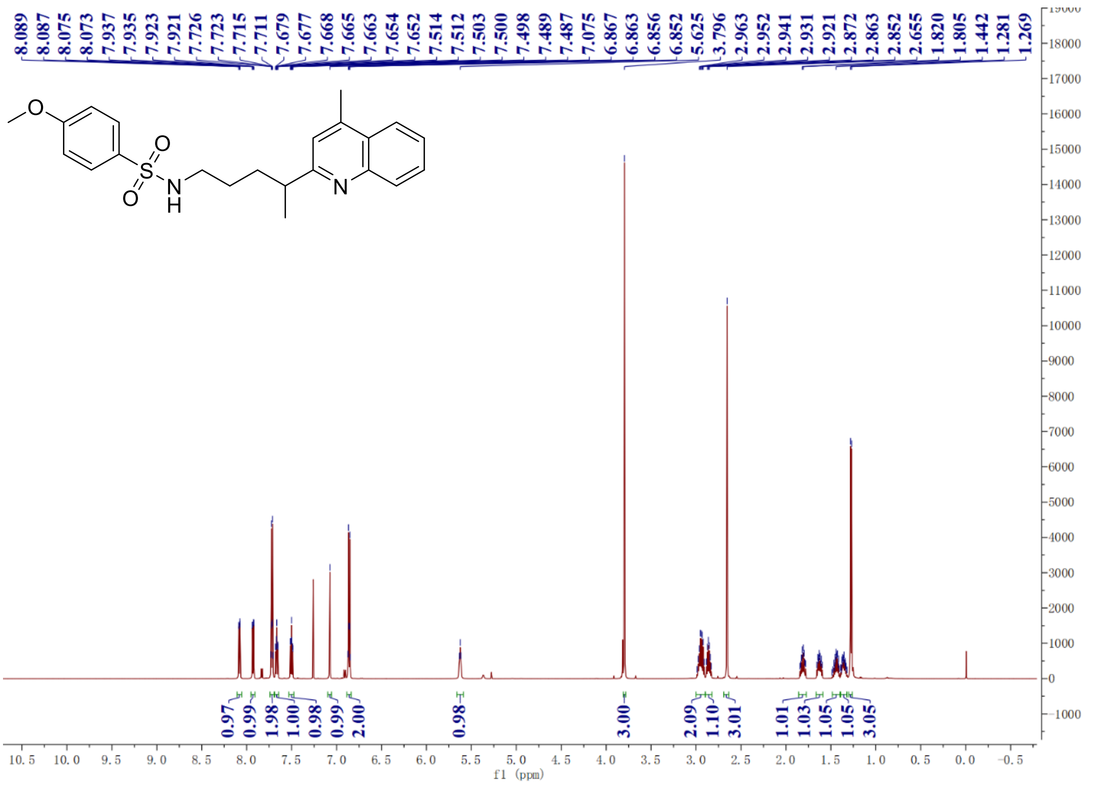

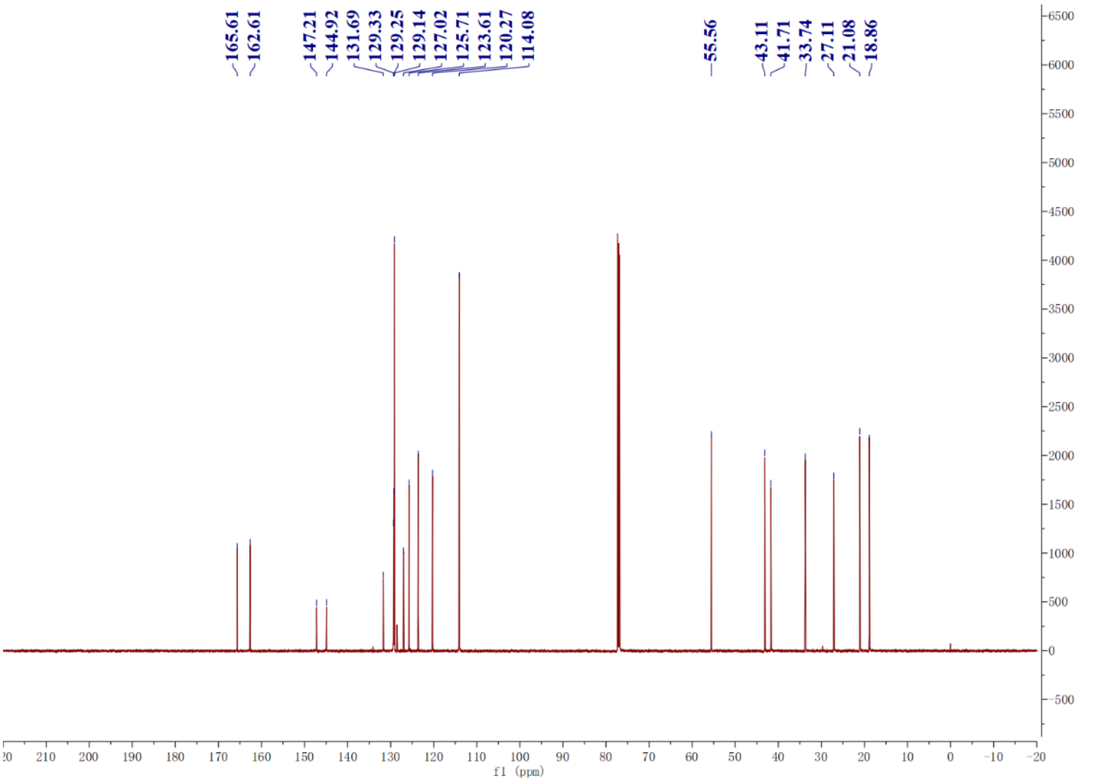

N-(4-(4-chloroquinolin-2-yl)pentyl)-4-methoxybenzenesulfonamide (10)

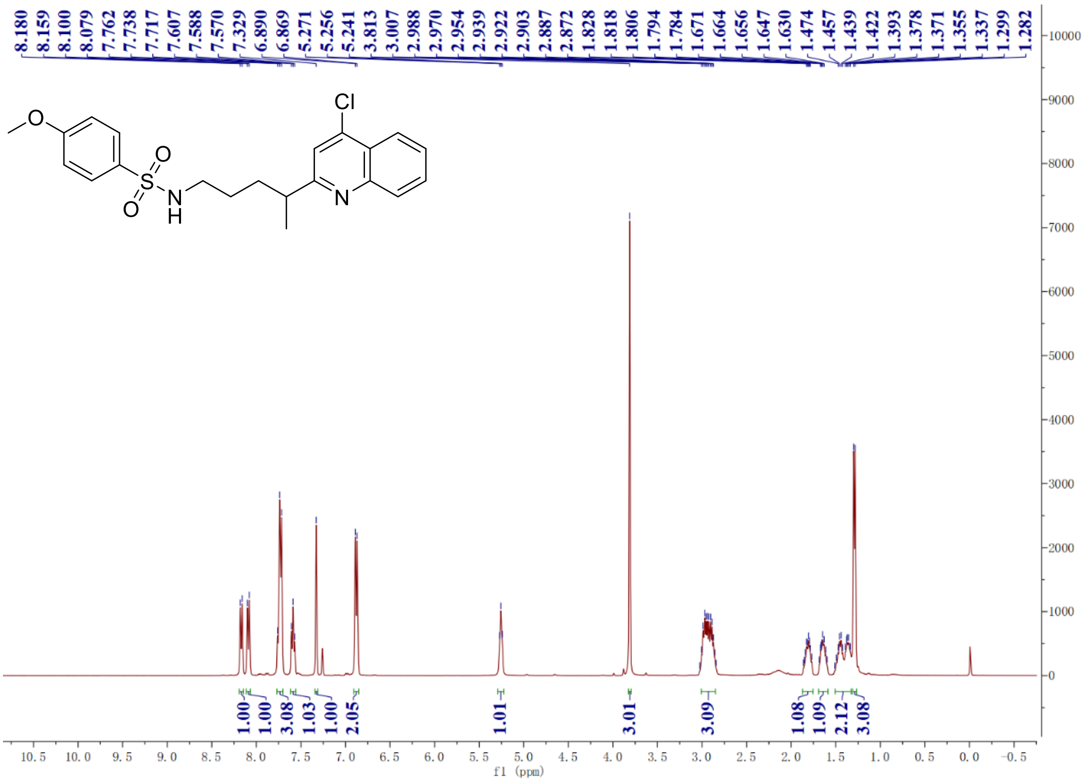

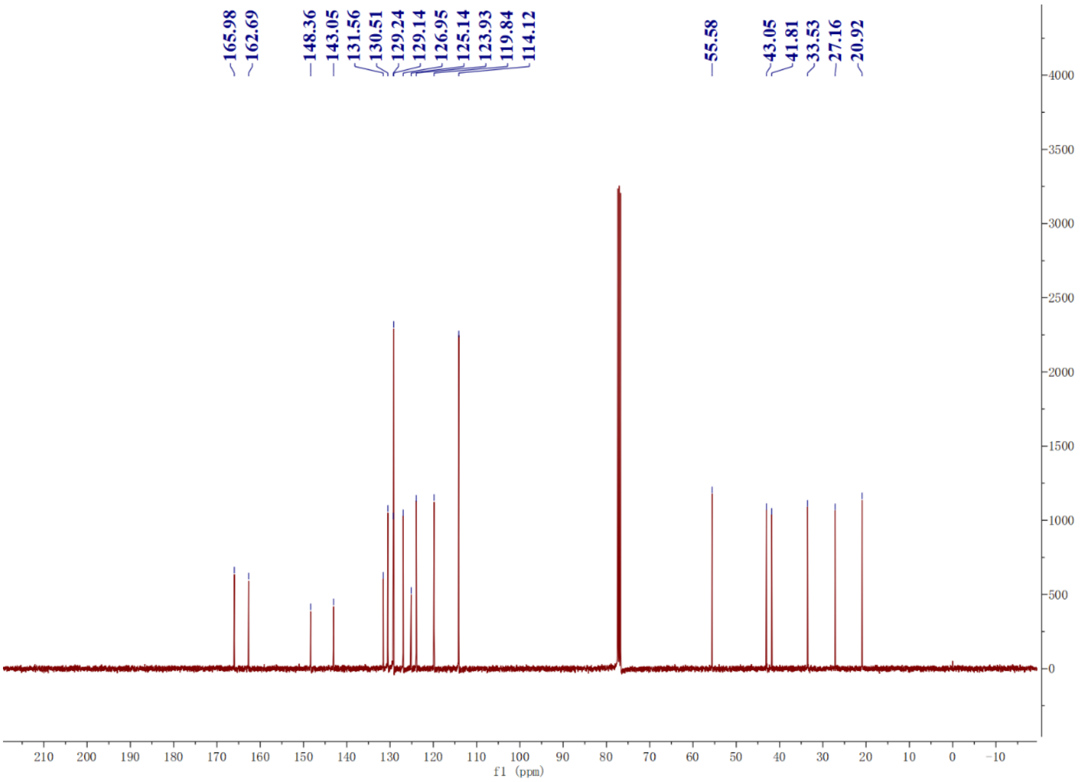

N-(4-(4-bromoquinolin-2-yl)pentyl)-4-methoxybenzenesulfonamide (11)

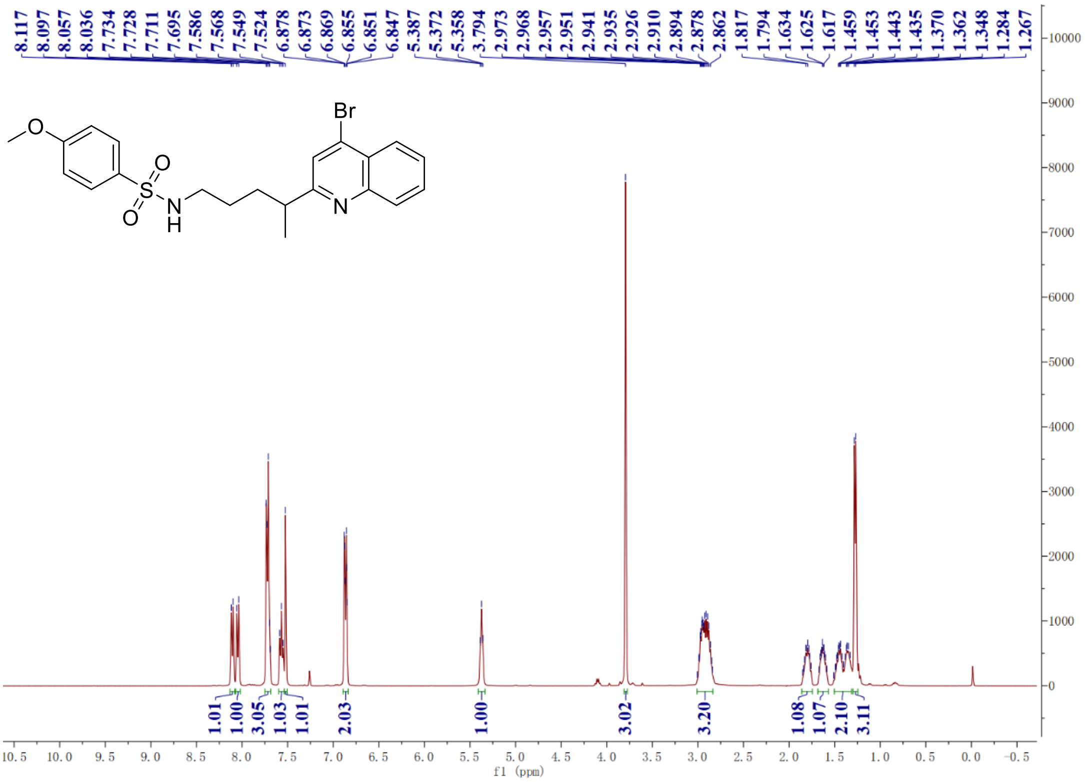

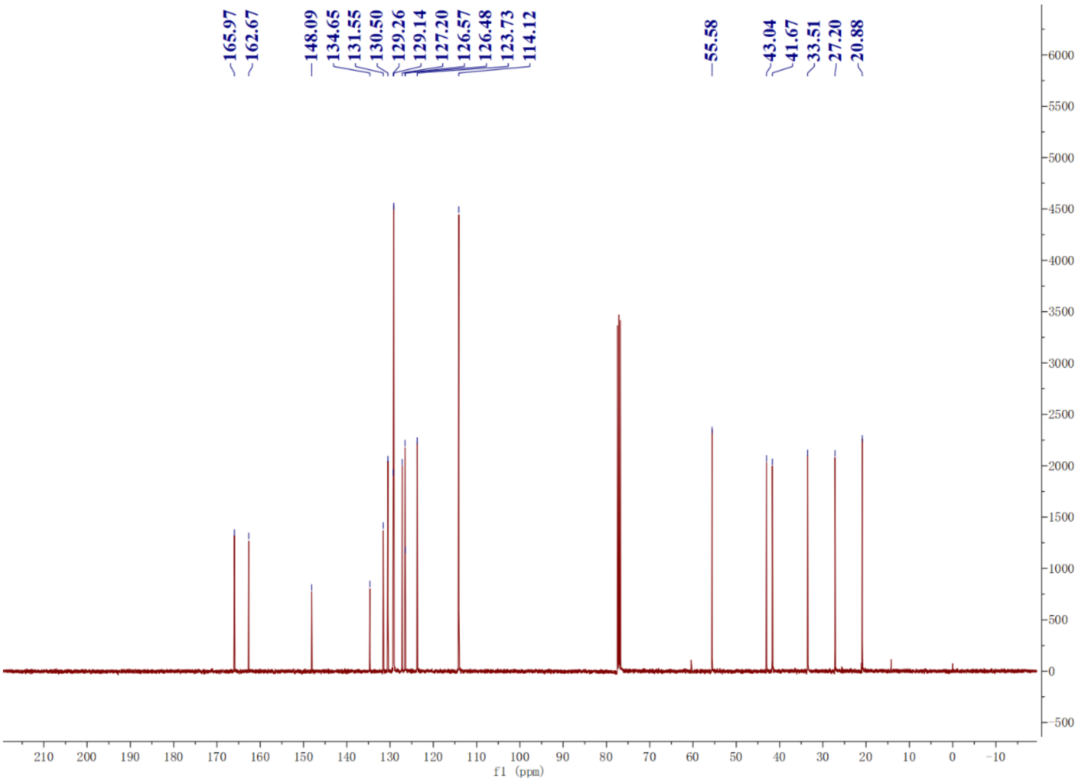

4-methoxy-*N*-(4-(2-methylquinolin-4-yl)pentyl)benzenesulfonamide (12)

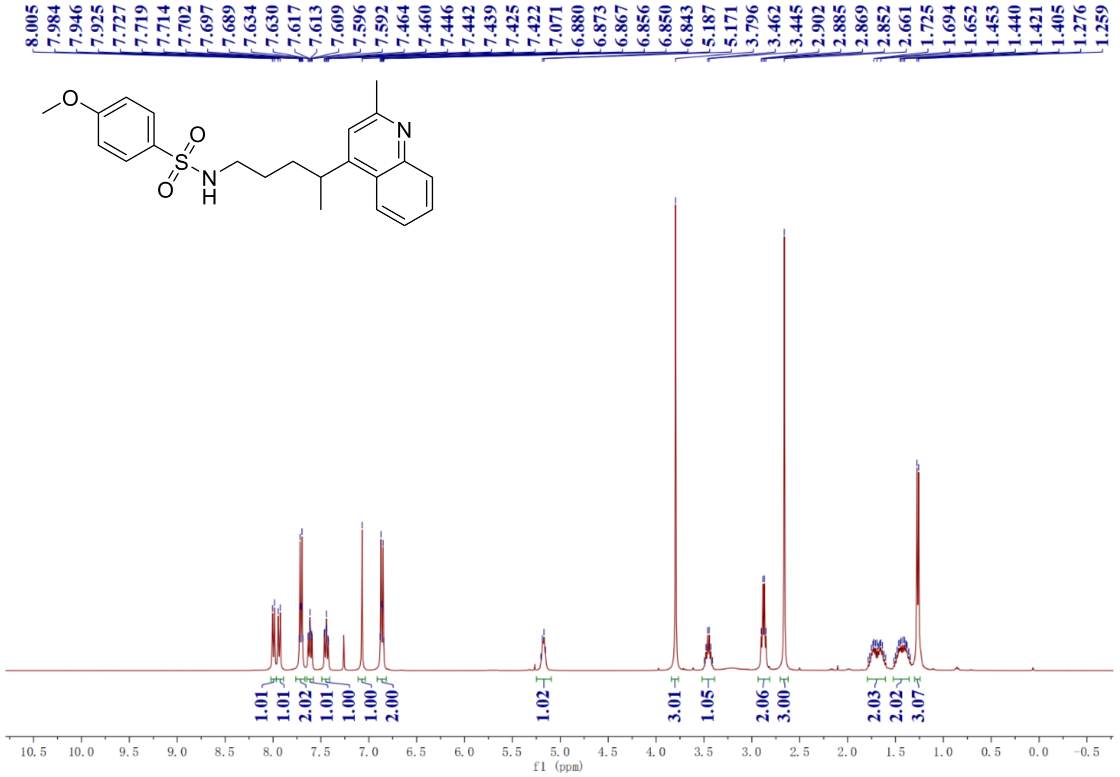

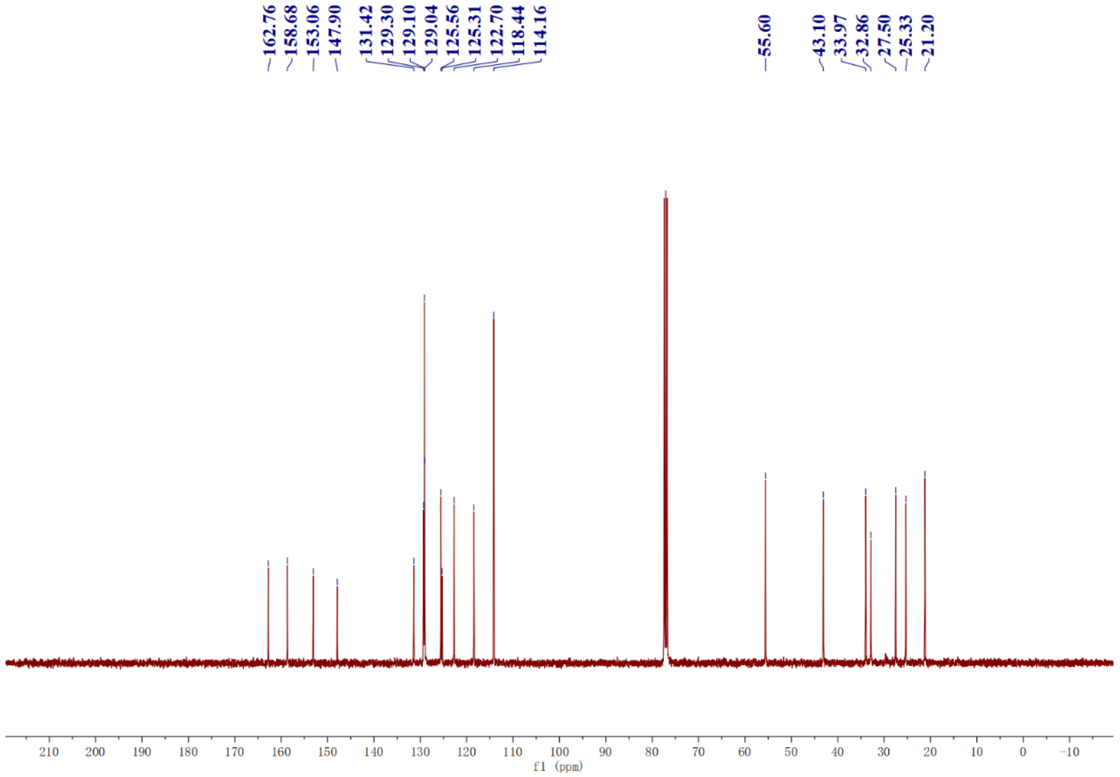

***N*-(4-(4,7-dichloroquinolin-2-yl)pentyl)-4-methoxybenzenesulfonamide (13)**

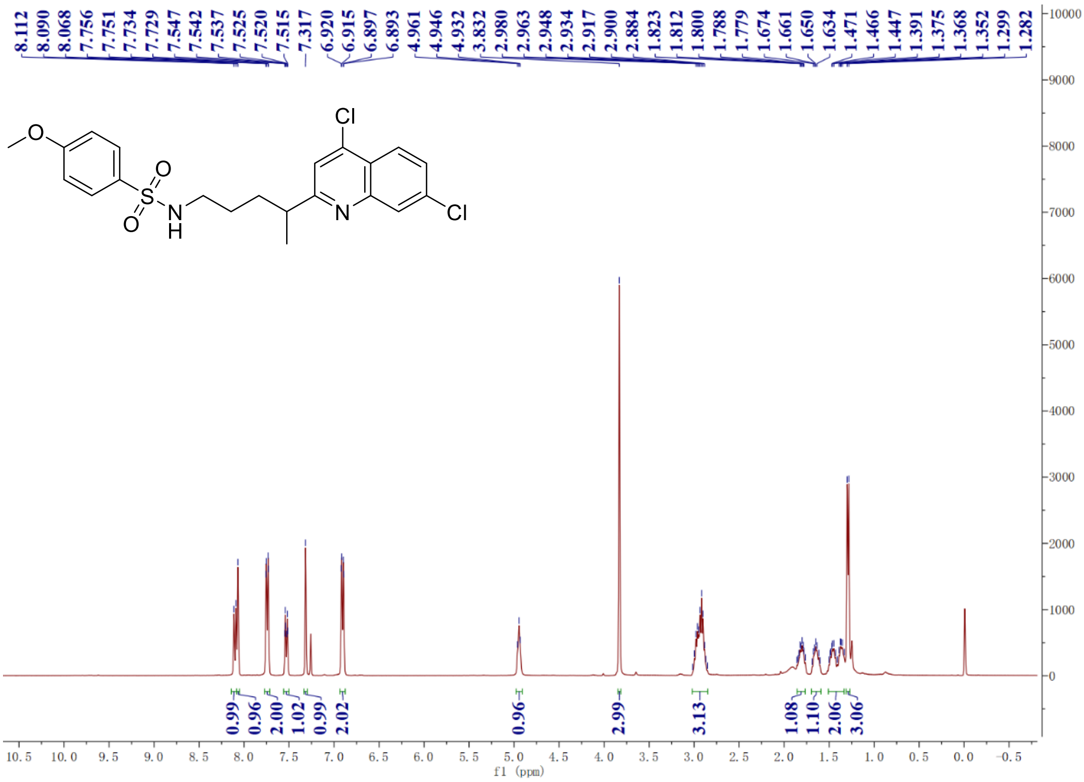

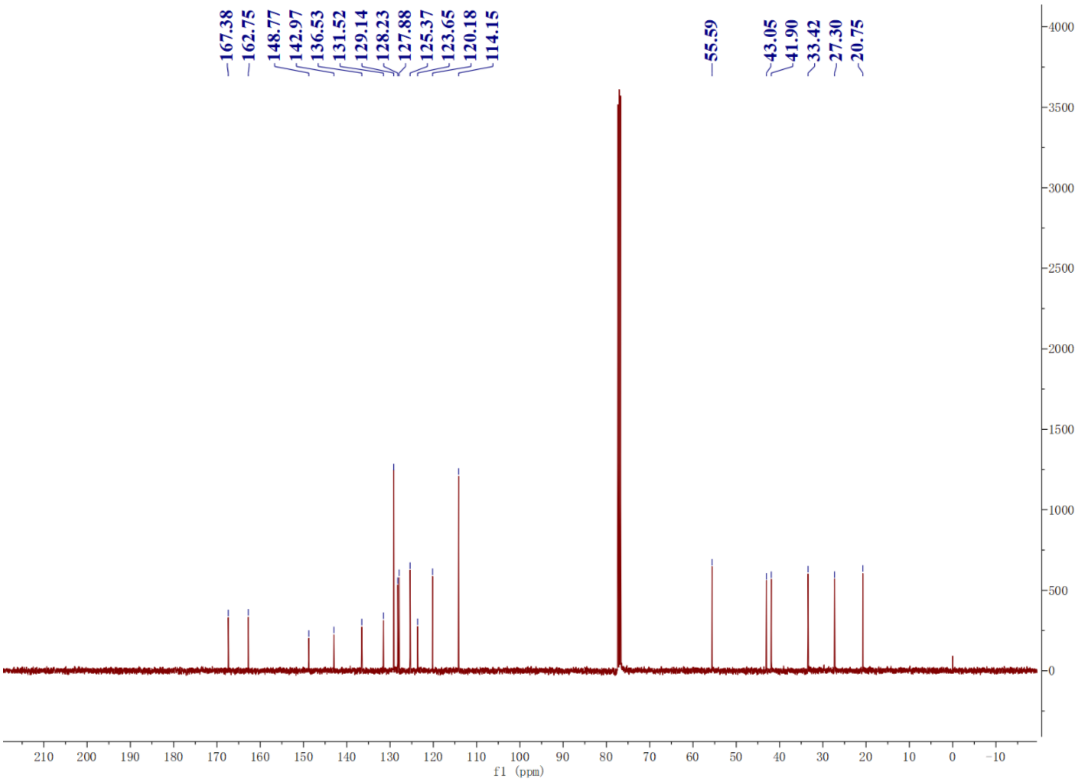

_N_-(4-(6-fluorobenzo[_d_]thiazol-2-yl)pentyl)-4-methoxybenzenesulfonamide (14)

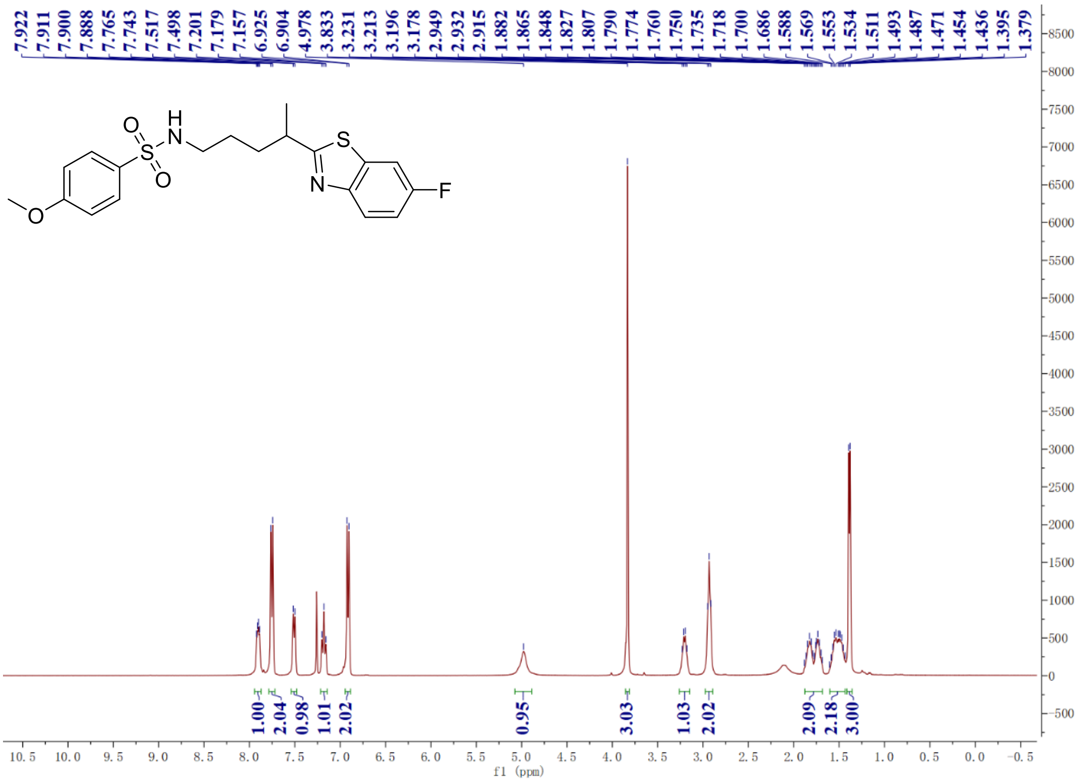

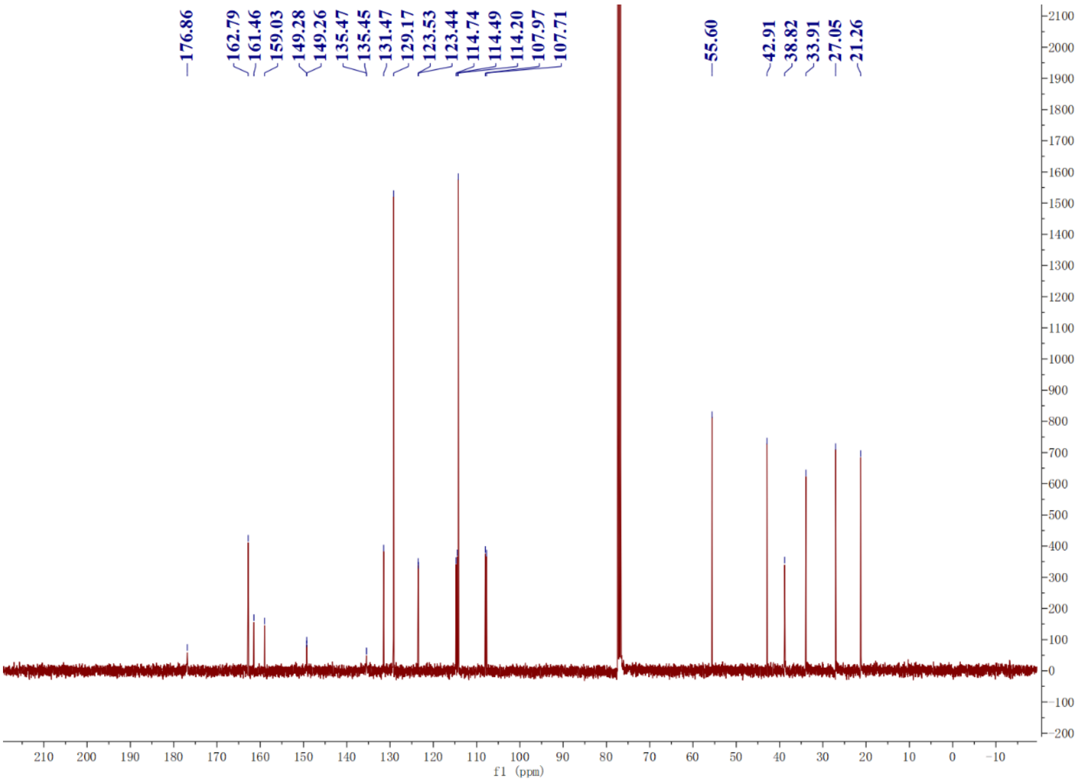

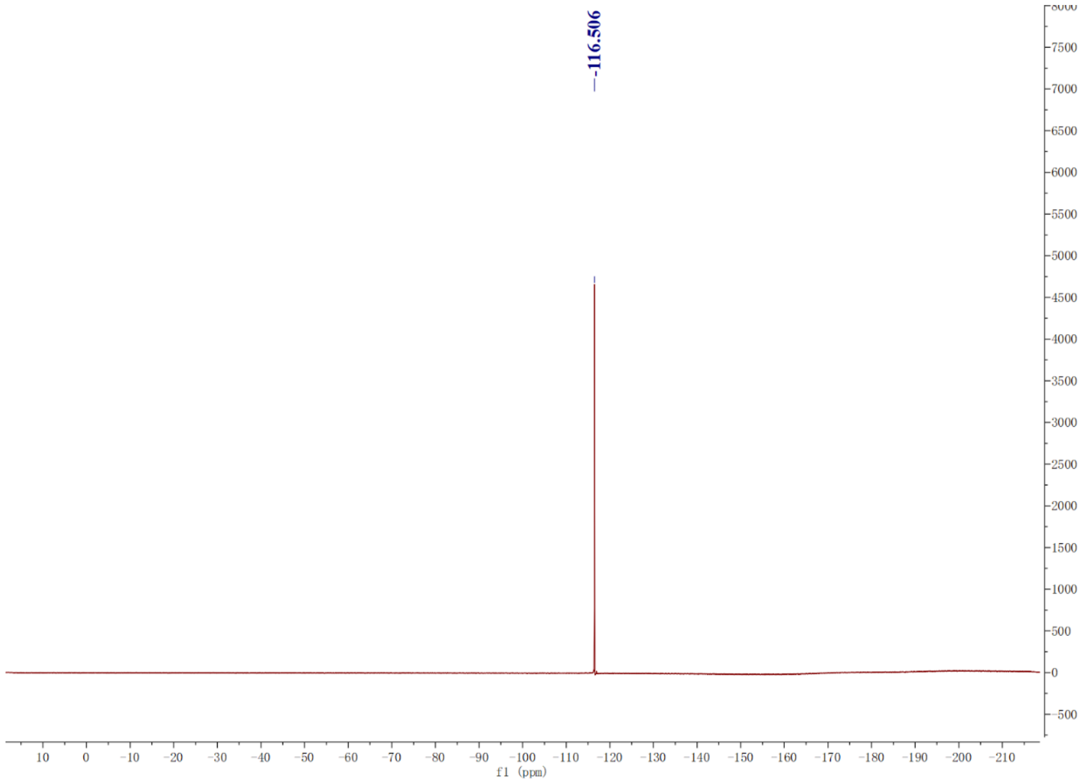

-116.506
f1 (ppm)

***N*-(4-(6-chlorobenzo[*d*]thiazol-2-yl)pentyl)-4-methoxybenzenesulfonamide (15)**

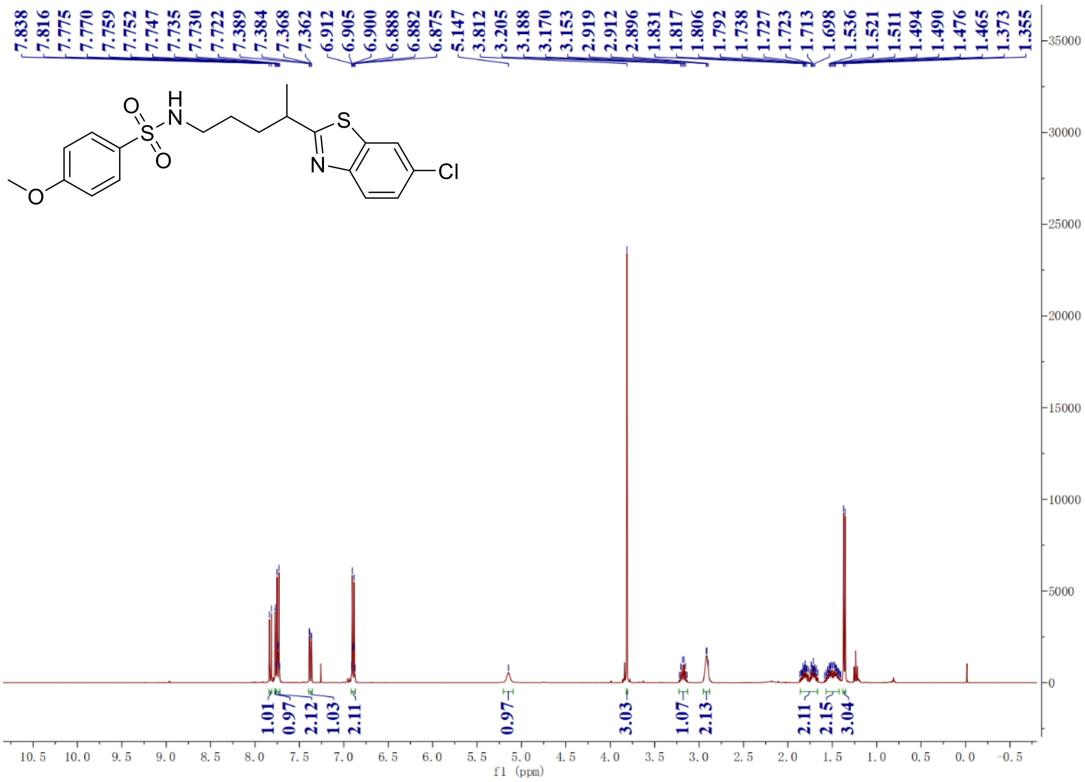

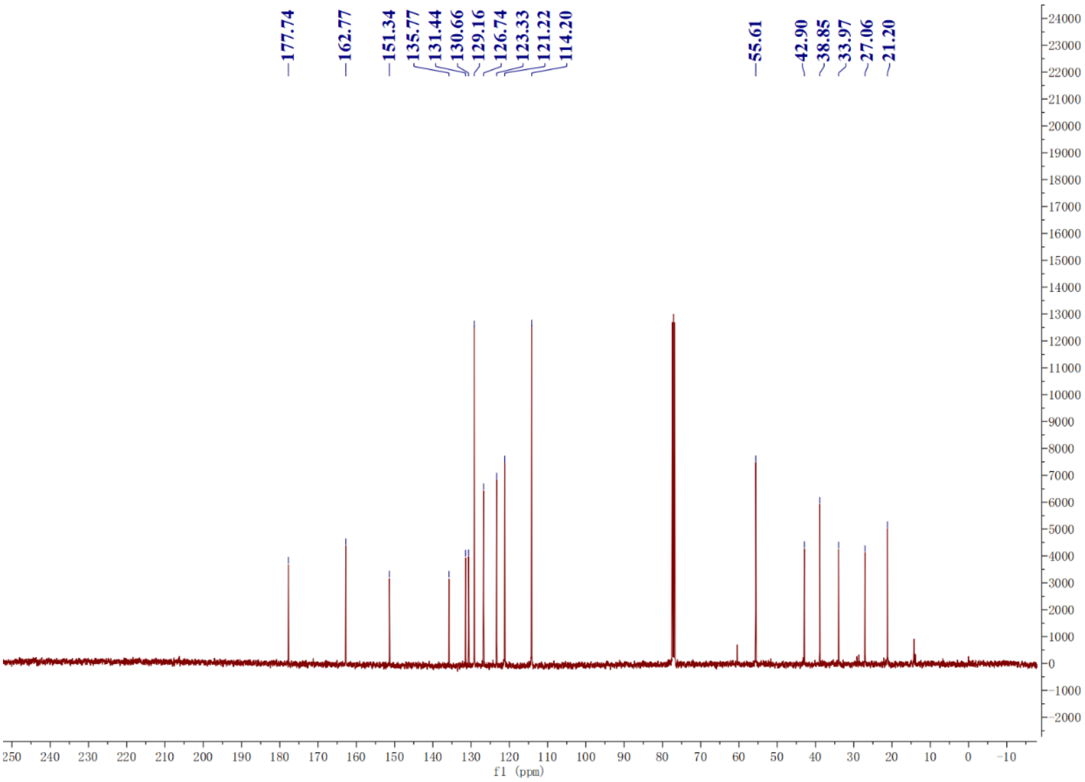

_N_-(4-(6-bromobenzo[_d_]thiazol-2-yl)pentyl)-4-methoxybenzenesulfonamide (16)

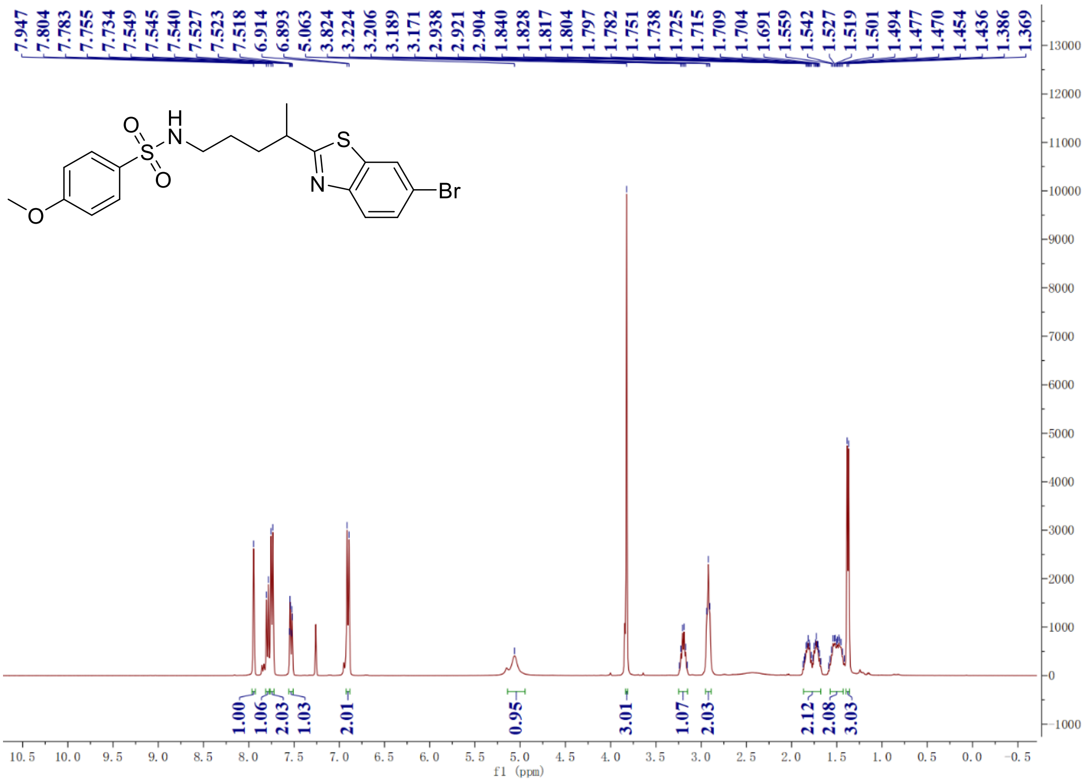

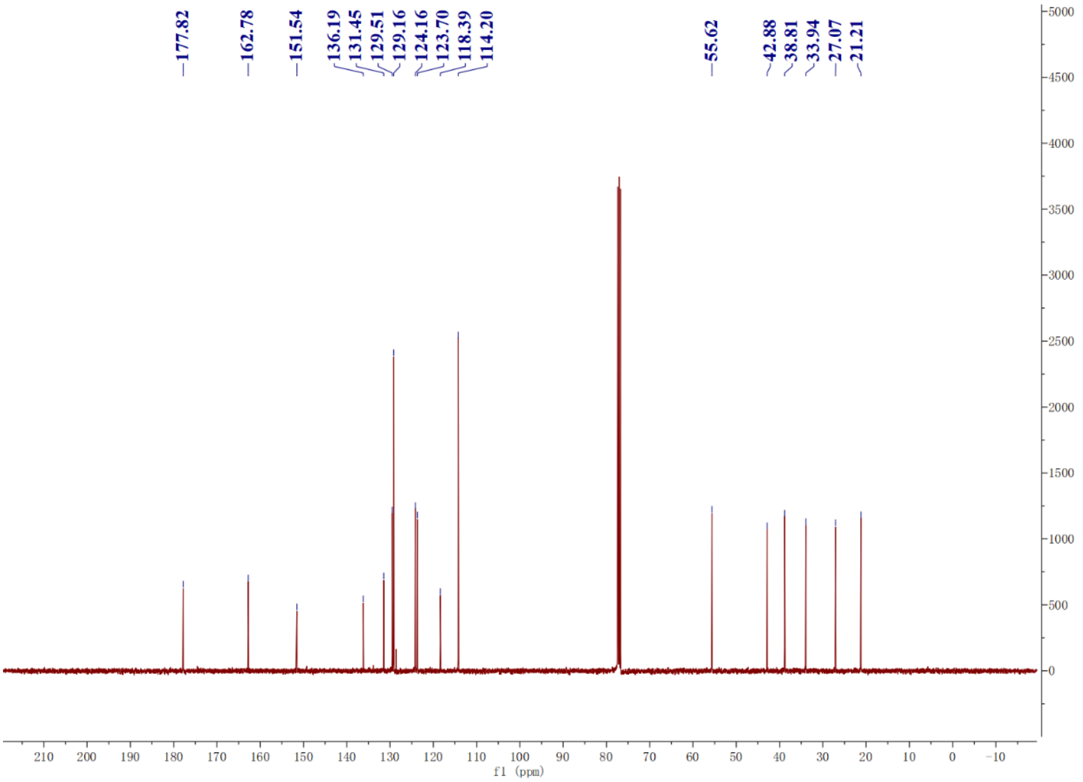

4-methoxy-*N*-(4-(6-(trifluoromethyl)benzo[*d*]thiazol-2-yl)pentyl)benzenesulfonamide (17)

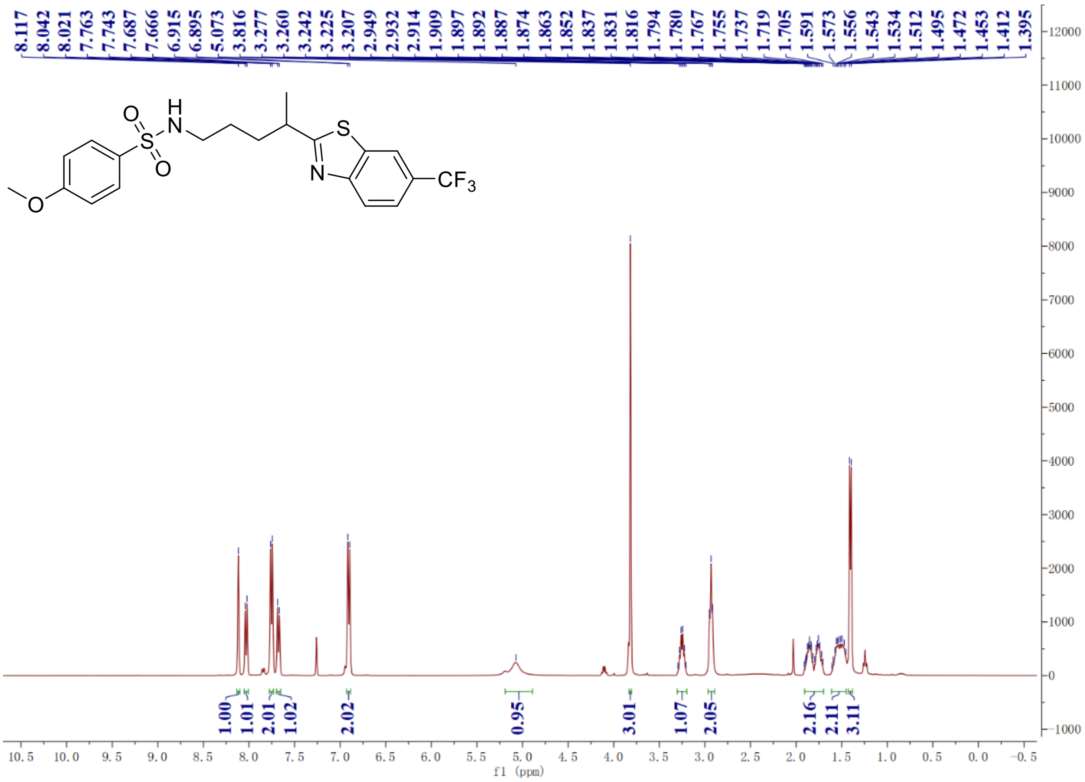

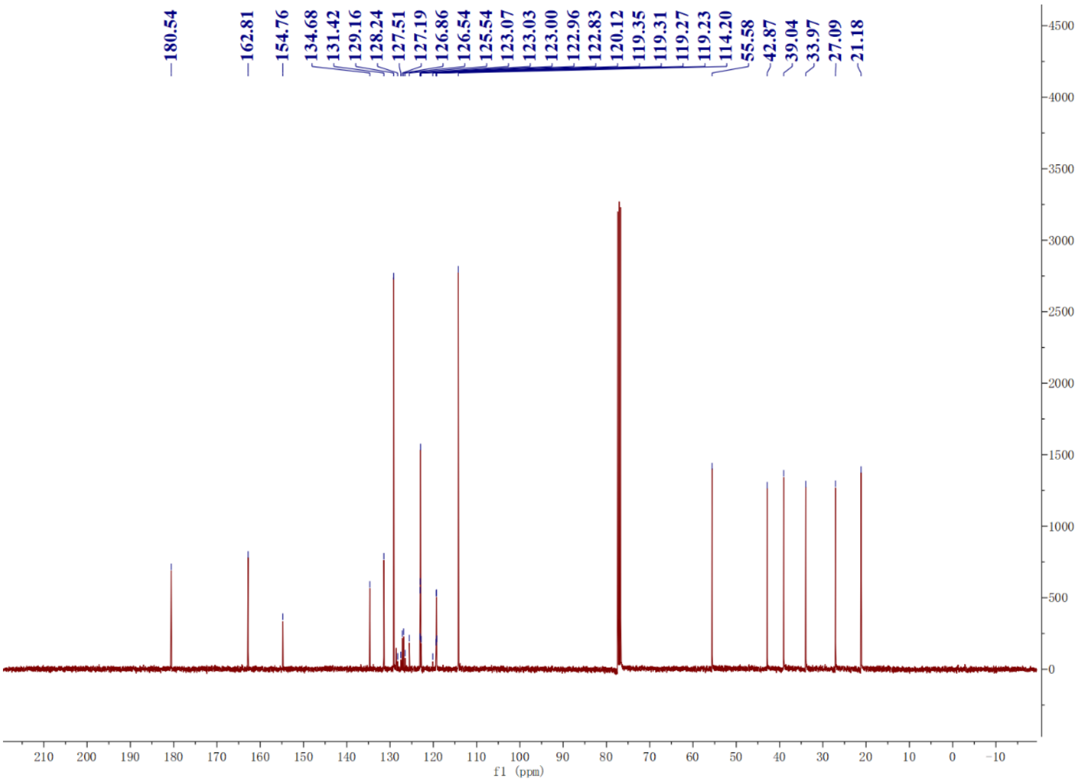

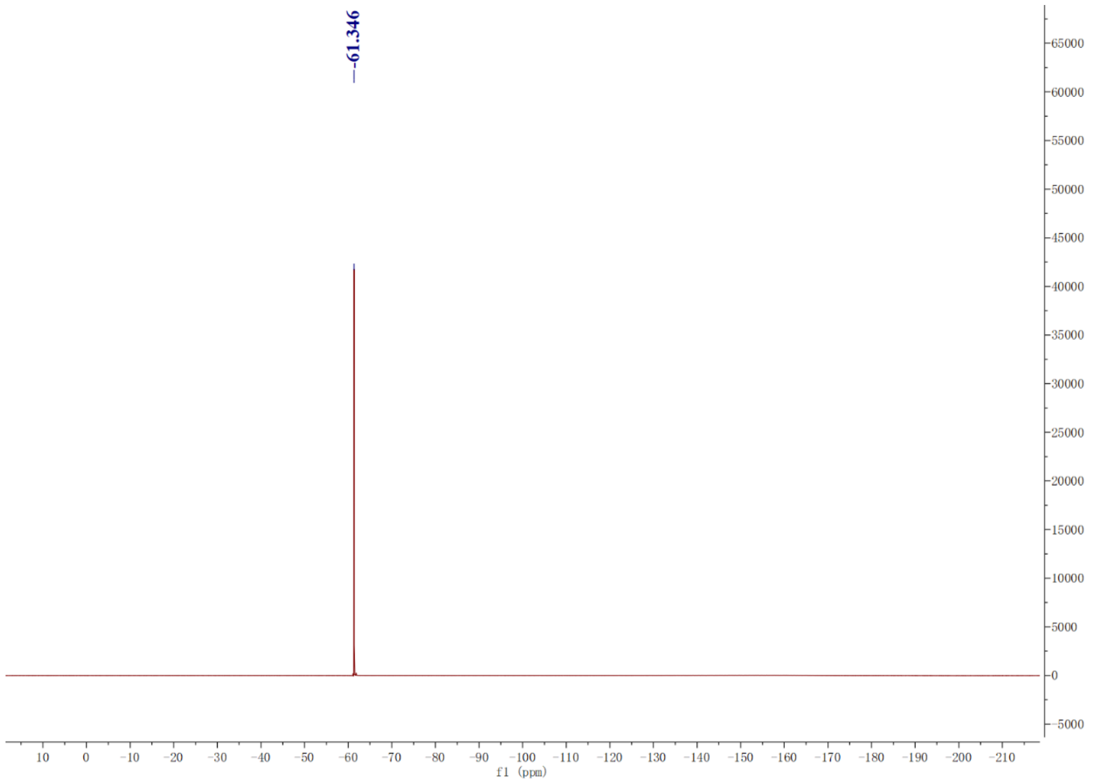

-61.346
65000
60000
55000
50000
45000
40000
35000
30000
25000
20000
15000
10000
5000
0
-5000
10 0 -10 -20 -30 -40 -50 -60 -70 -80 -90 -100 -110 -120 -130 -140 -150 -160 -170 -180 -190 -200 -210
f1 (ppm)

4-methoxy-*N*-(4-(6-(trifluoromethoxy)benzo[*d*]thiazol-2-yl)pentyl)benzenesulfonamide (18)

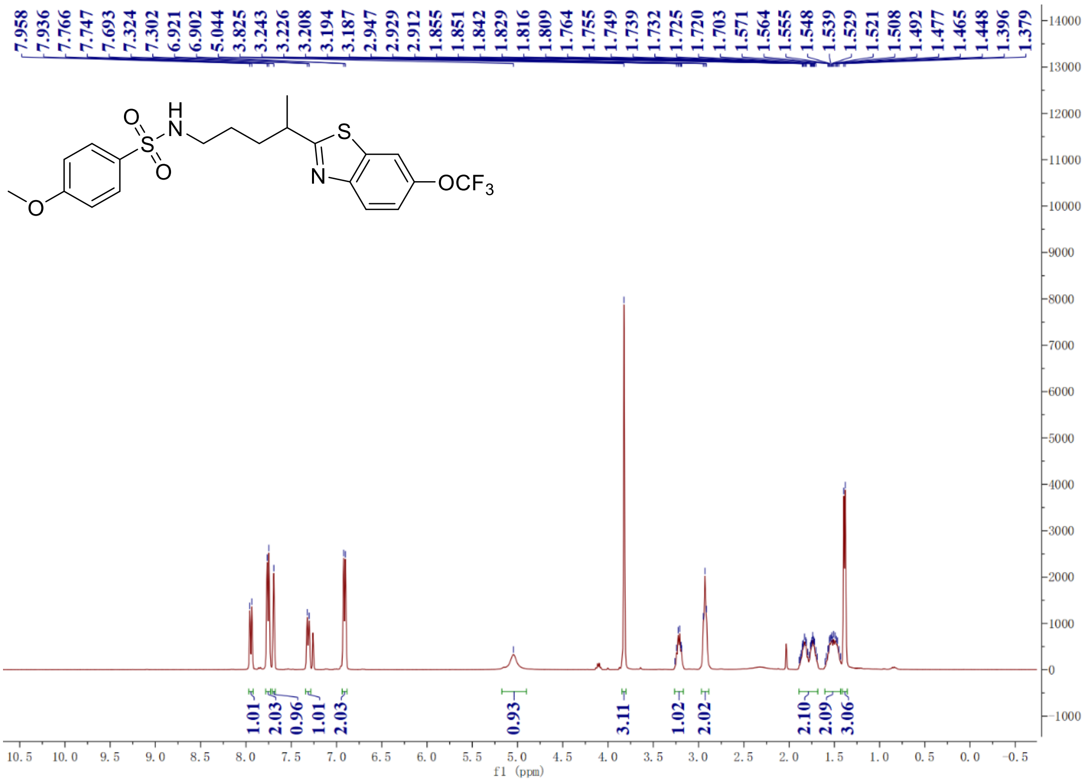

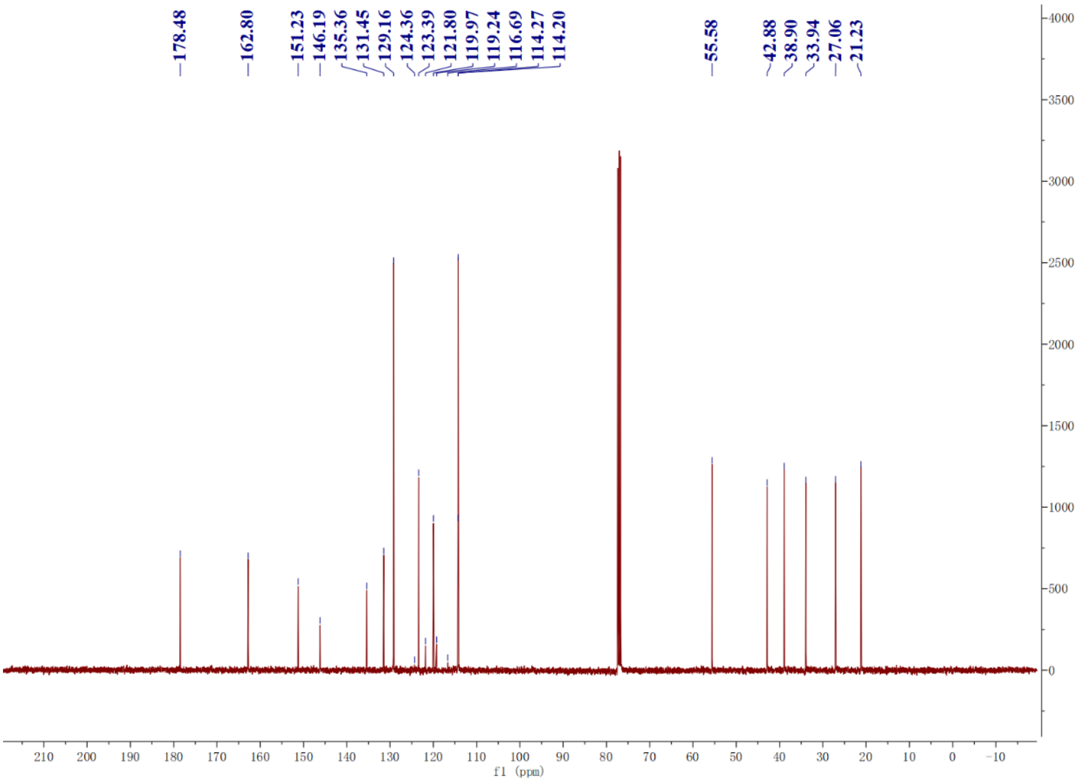

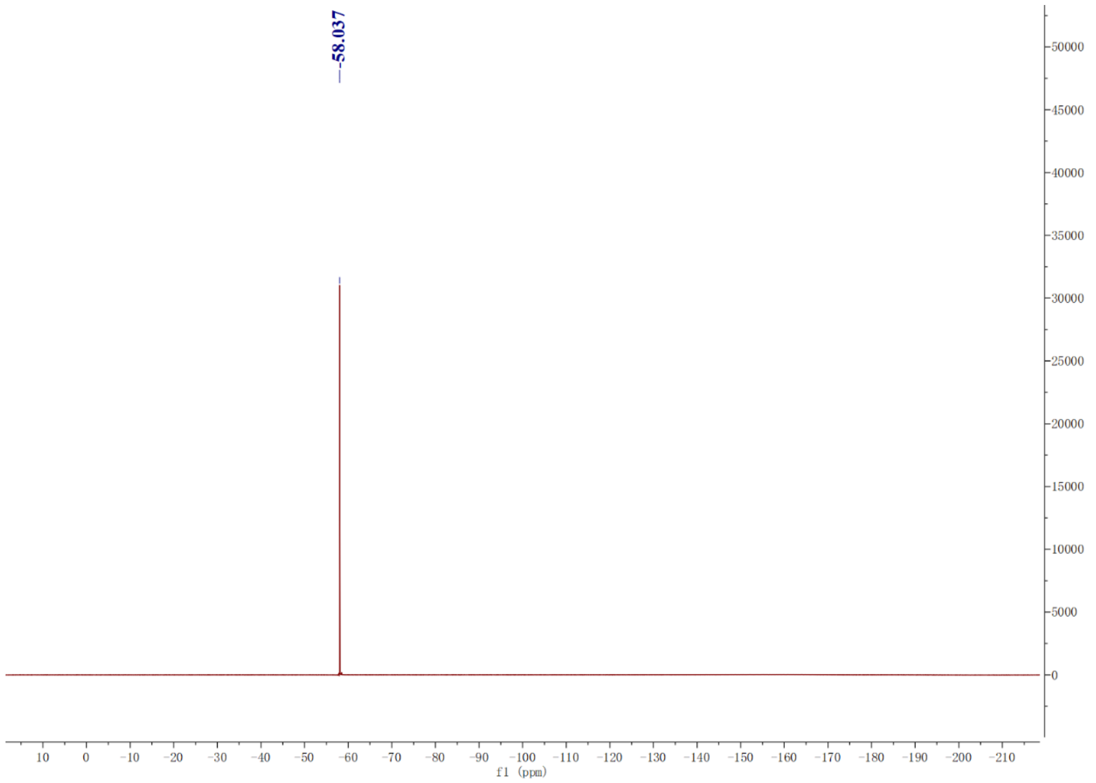

-58.037
50000
45000
40000
35000
30000
25000
20000
15000
10000
5000
0
10 0 -10 -20 -30 -40 -50 -60 -70 -80 -90 -100 -110 -120 -130 -140 -150 -160 -170 -180 -190 -200 -210
f1 (ppm)

ethyl 2-(5-((4-methoxyphenyl)sulfonamido)pentan-2-yl)benzo[*d*]thiazole-6-carboxylate (19)

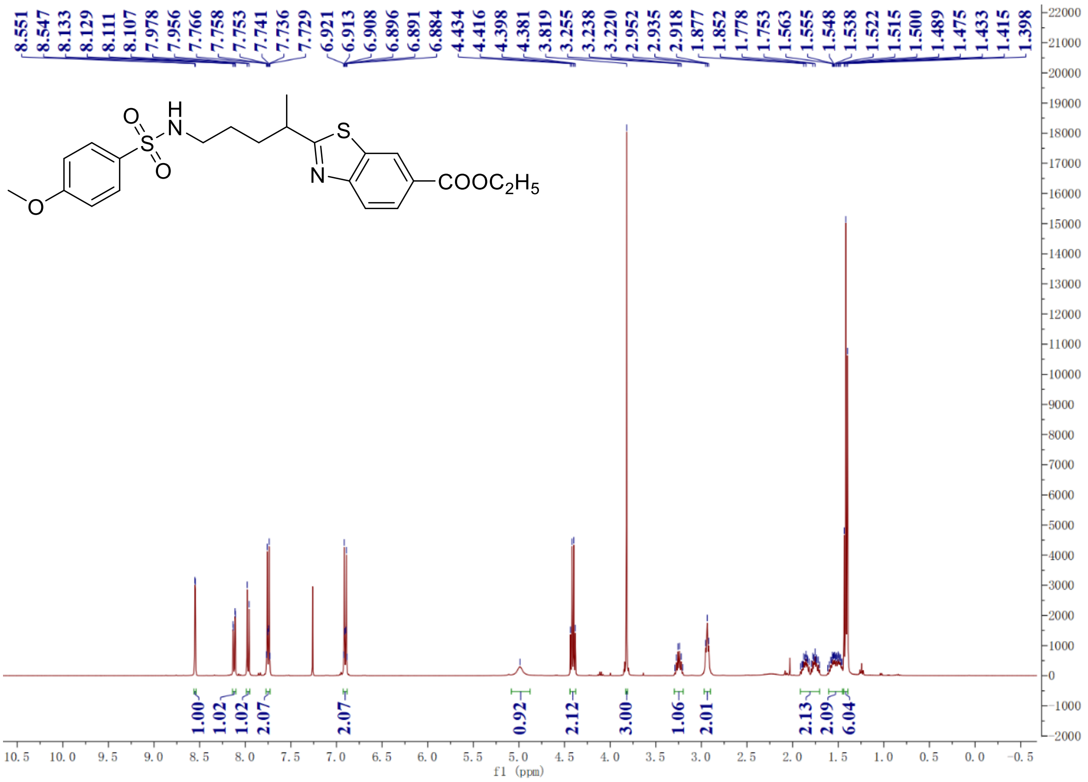

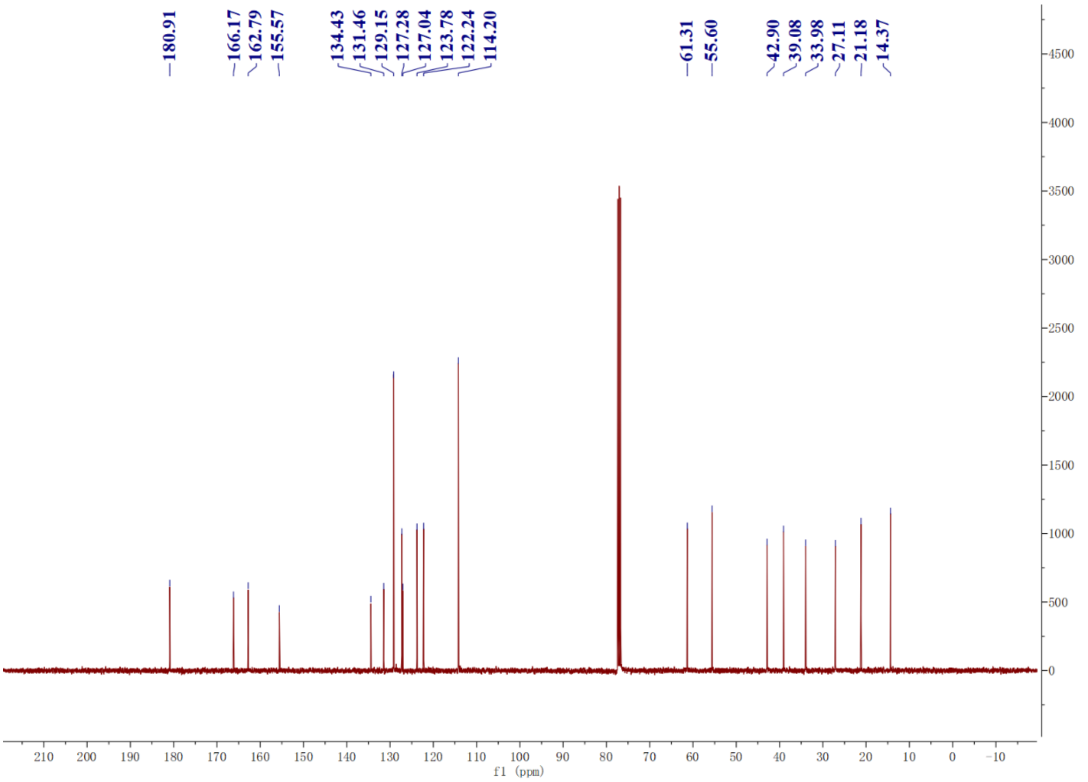

***N*-(4-(5-chlorobenzo[*d*]thiazol-2-yl)pentyl)-4-methoxybenzenesulfonamide (20)**

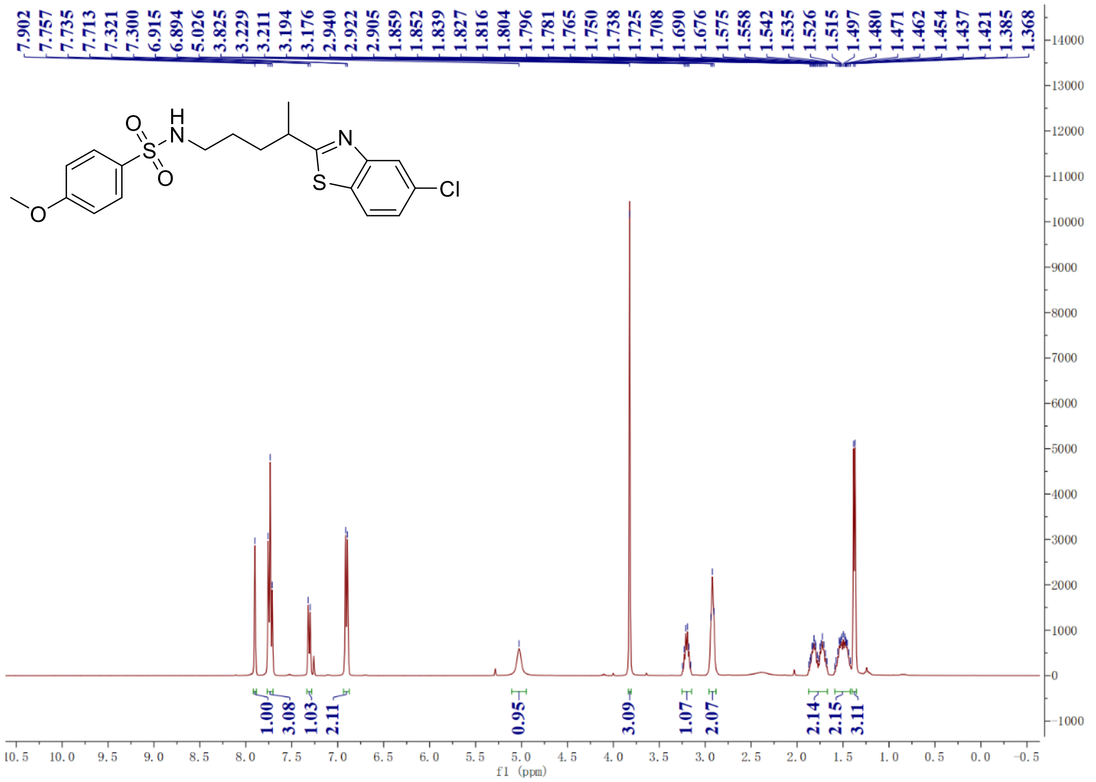

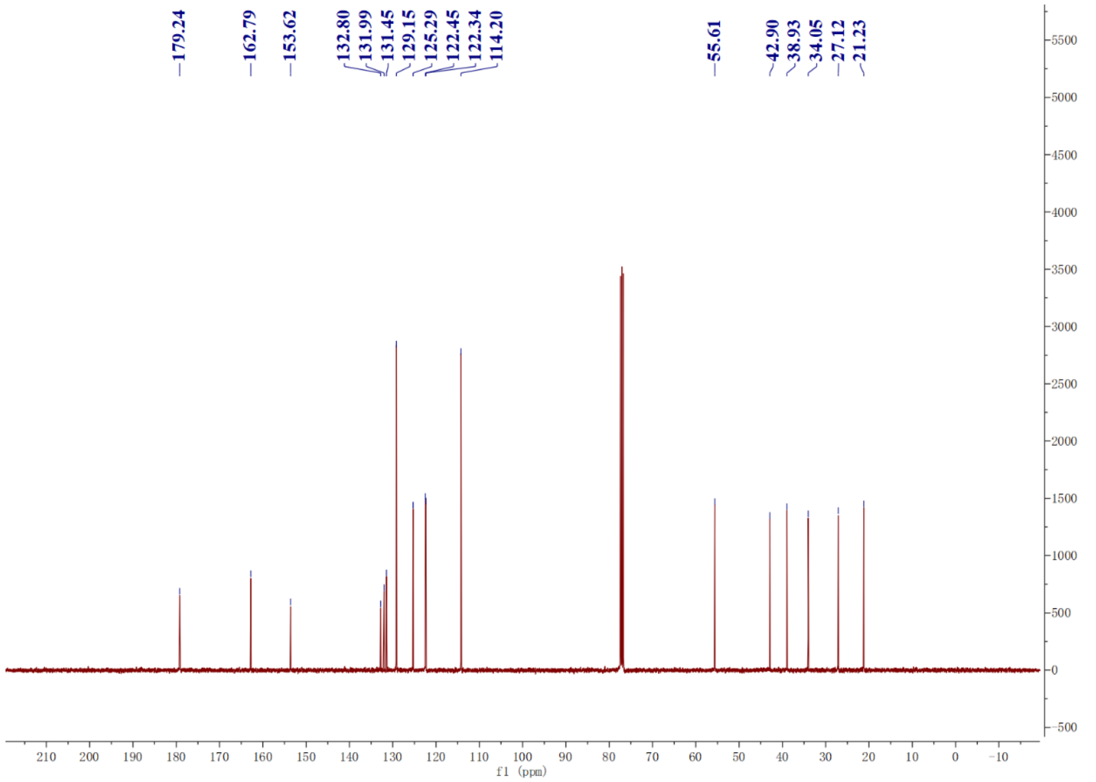

N-(4-(5-bromobenzo[*d*]thiazol-2-yl)pentyl)-4-methoxybenzenesulfonamide (21)

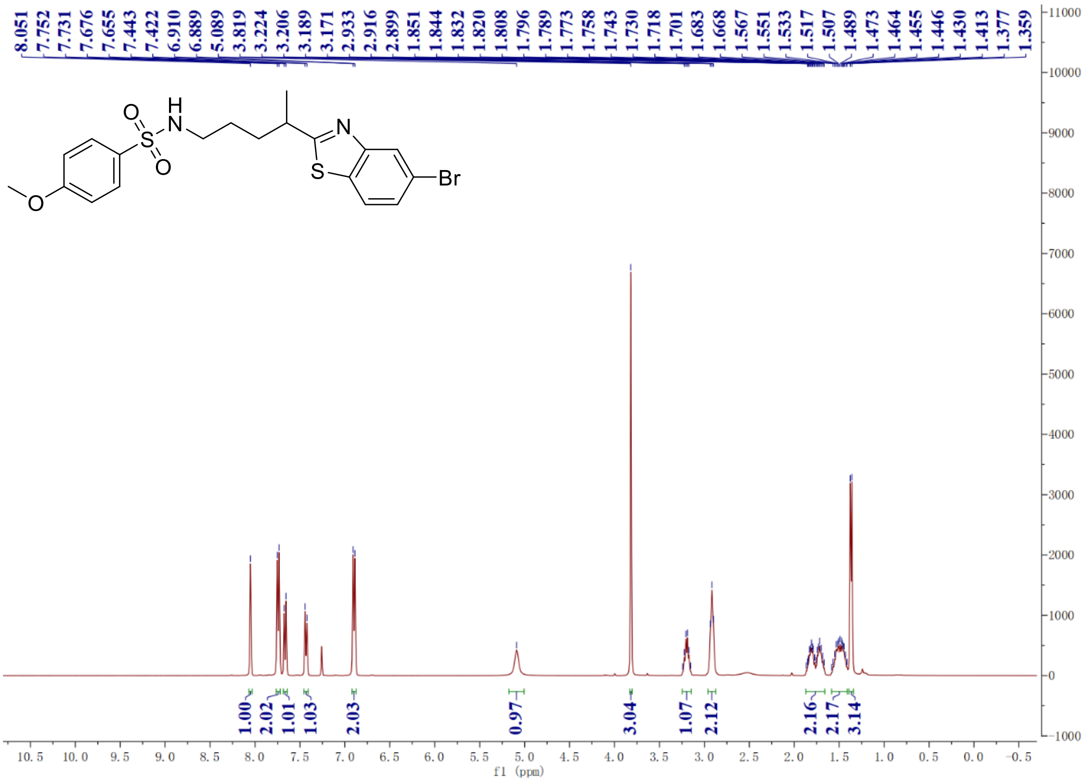

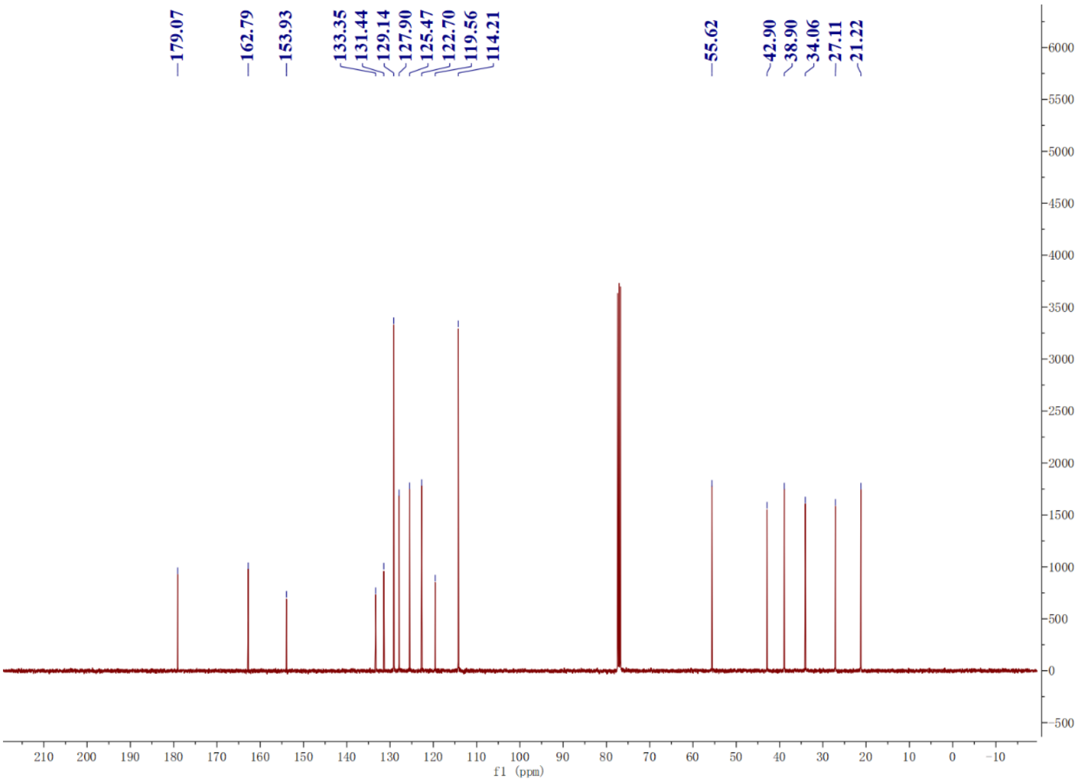

***N*-(4-(5,6-dimethylbenzo[*d*]thiazol-2-yl)pentyl)-4-methoxybenzenesulfonamide (22)**

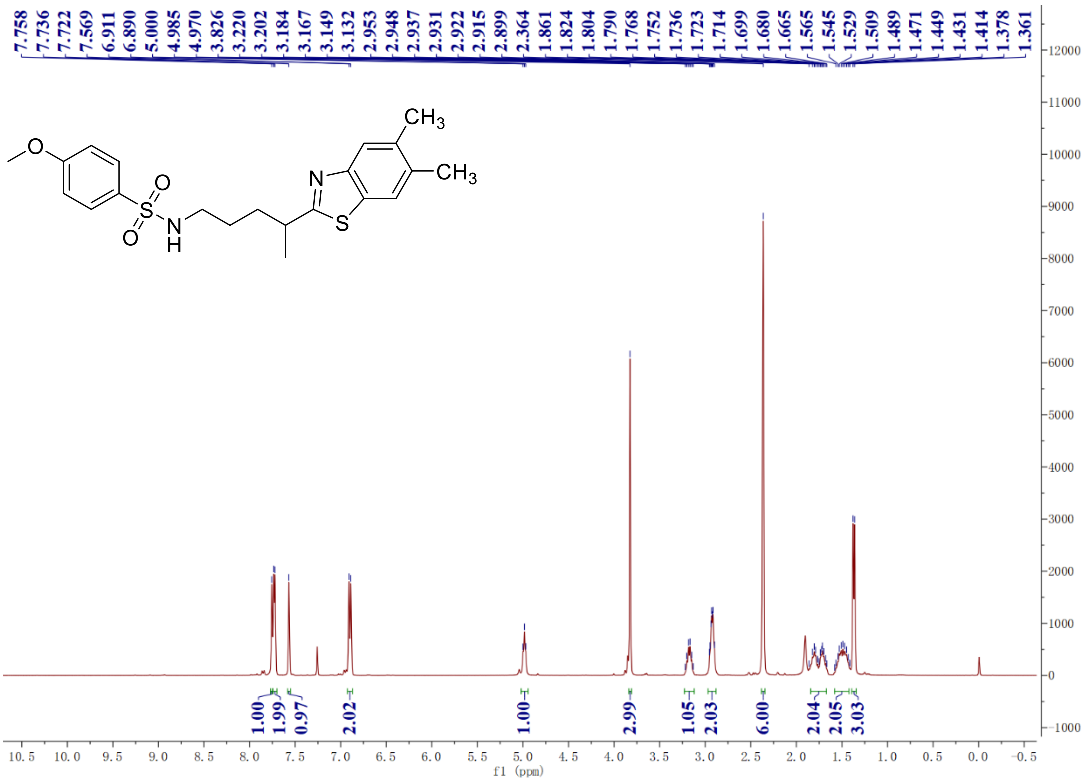

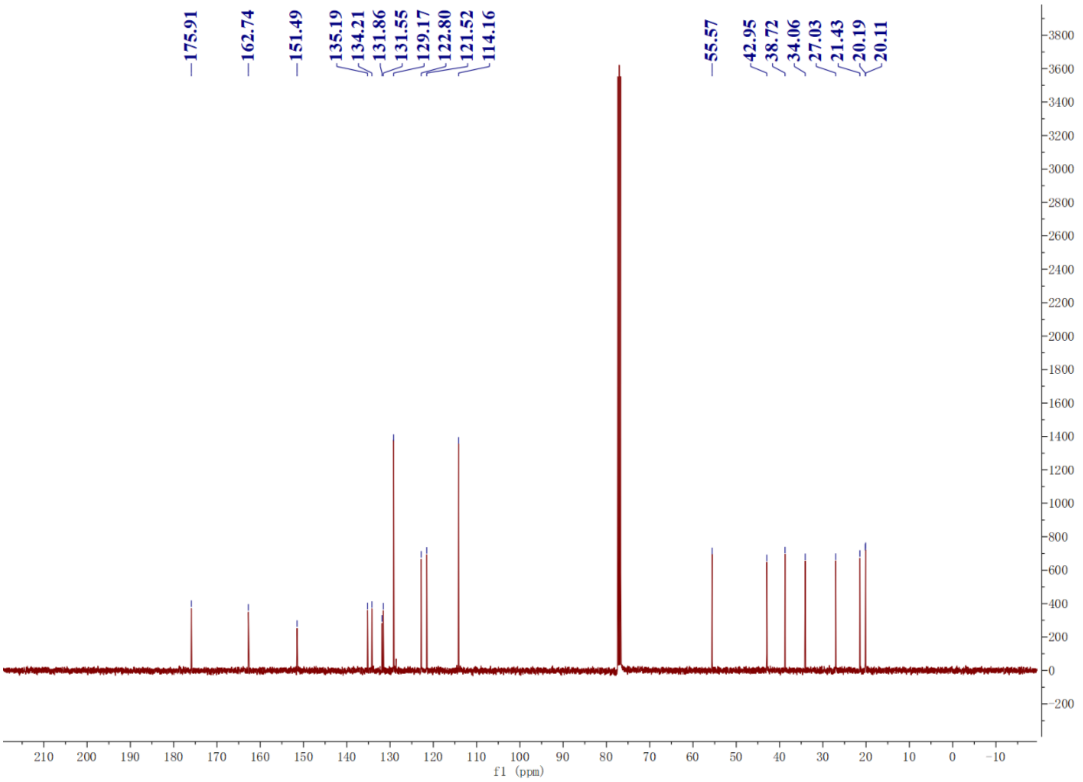

***N*-(4-(benzo[*d*]thiazol-2-yl)pentyl)-4-methoxybenzenesulfonamide (23)**

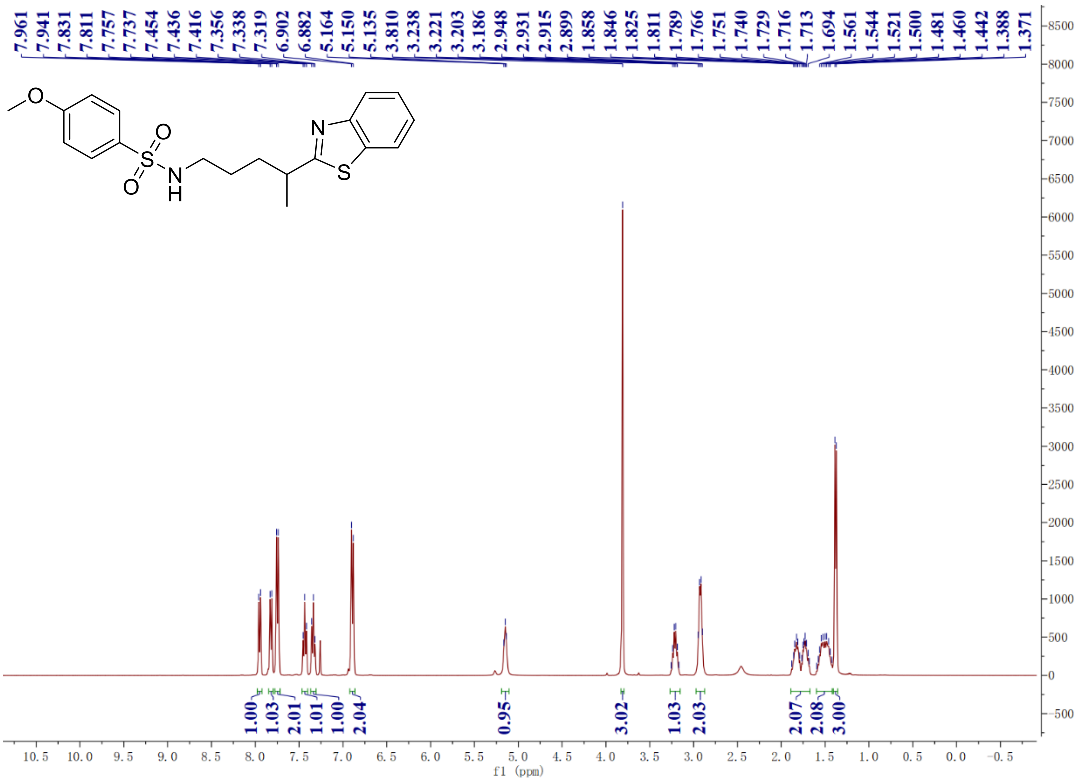

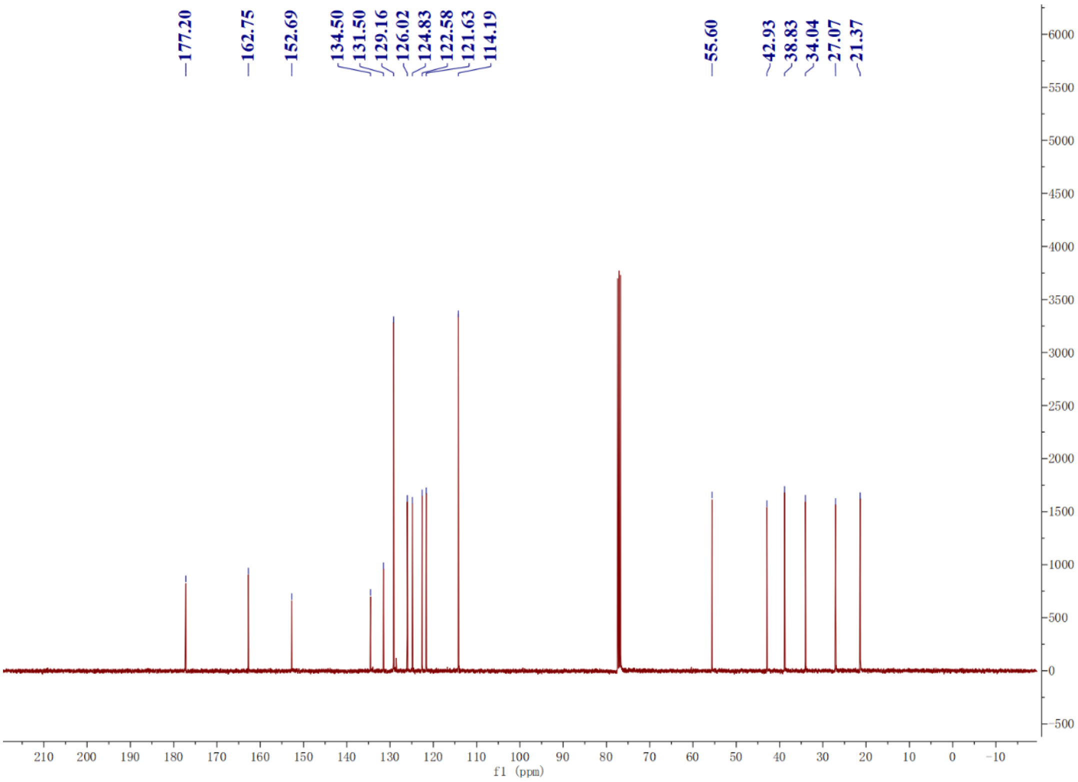

N-(4-(isoquinolin-1-yl)pentyl)-4-methoxybenzenesulfonamide (24)

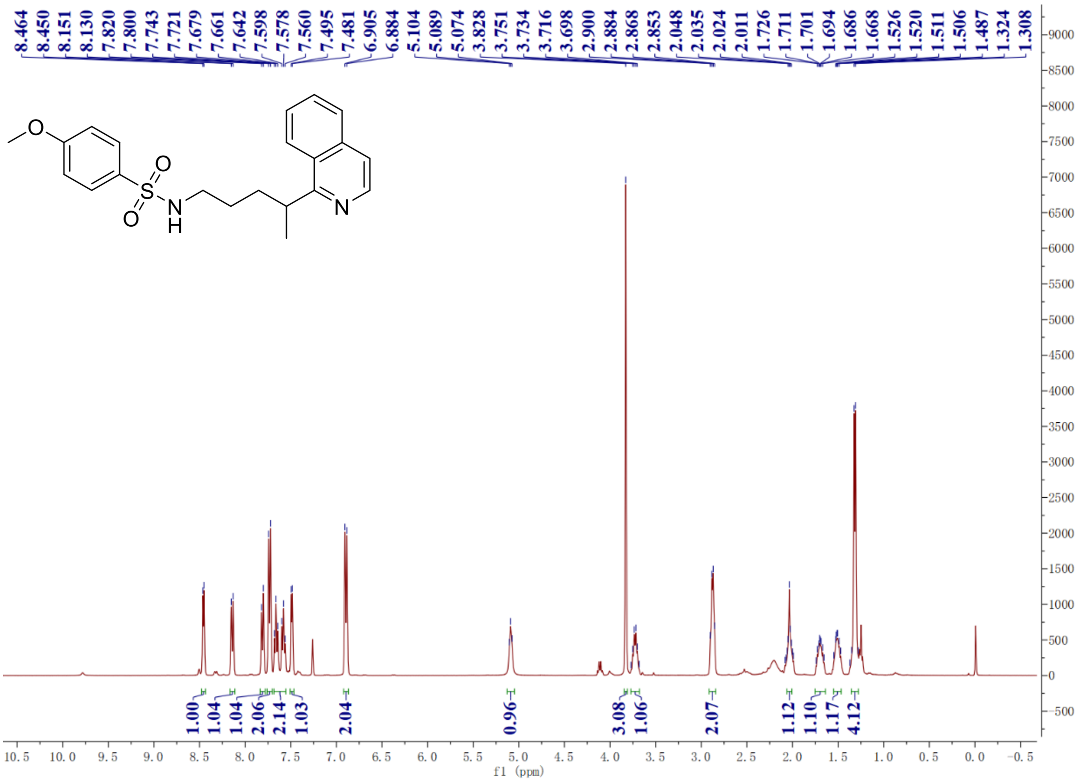

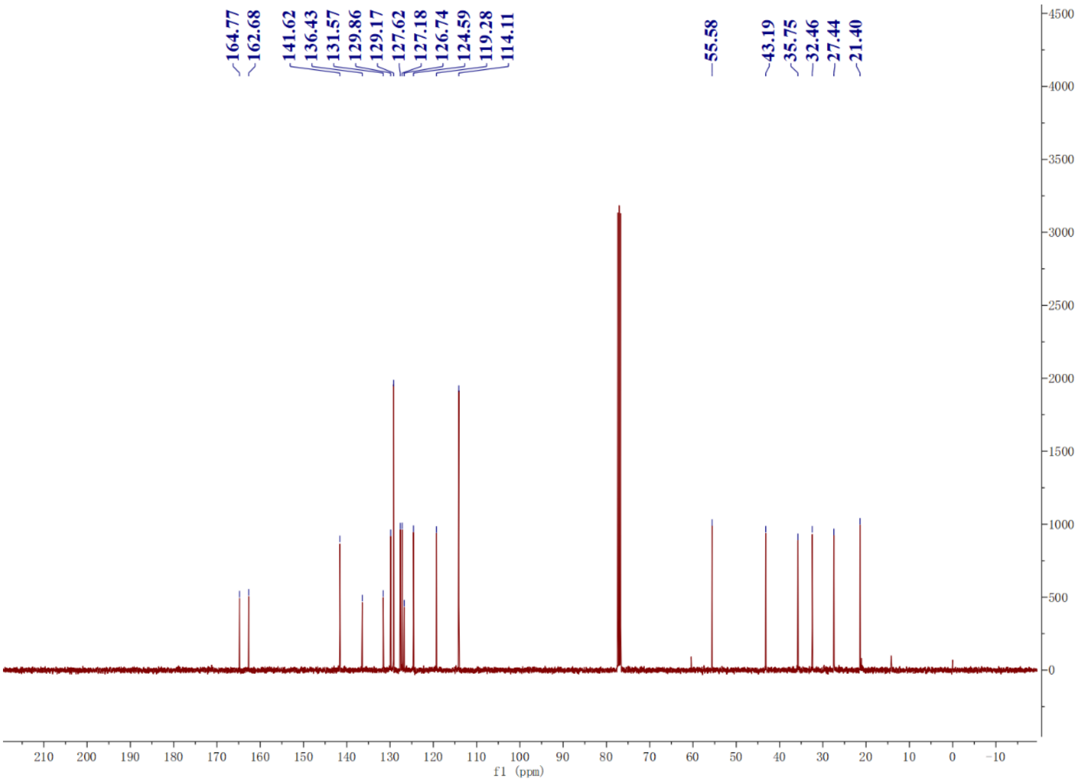

N-(4-(4-bromoisoquinolin-1-yl)pentyl)-4-methoxybenzenesulfonamide (25)

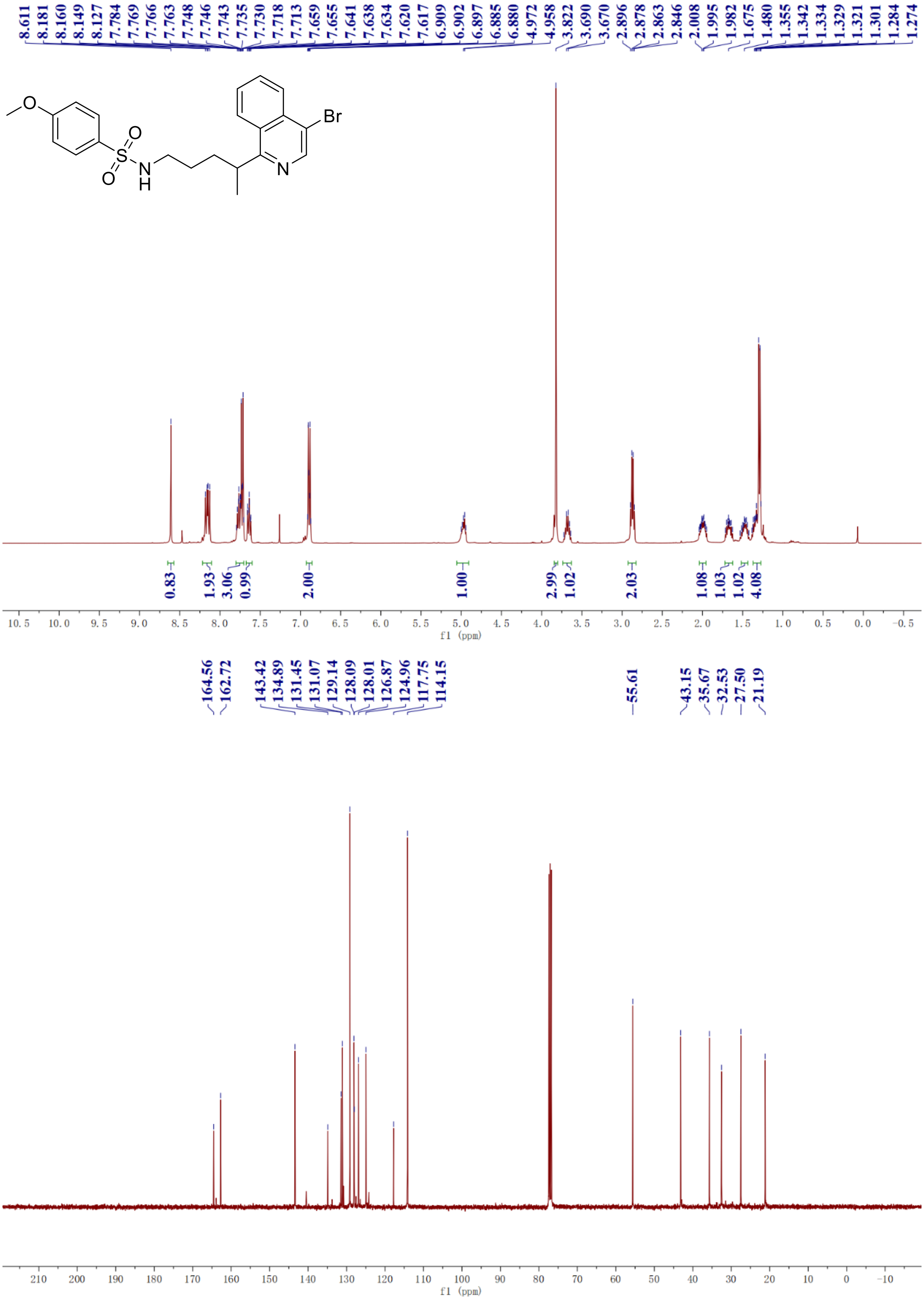

4-methoxy-*N*-(4-(quinazolin-4-yl)pentyl)benzenesulfonamide (26)

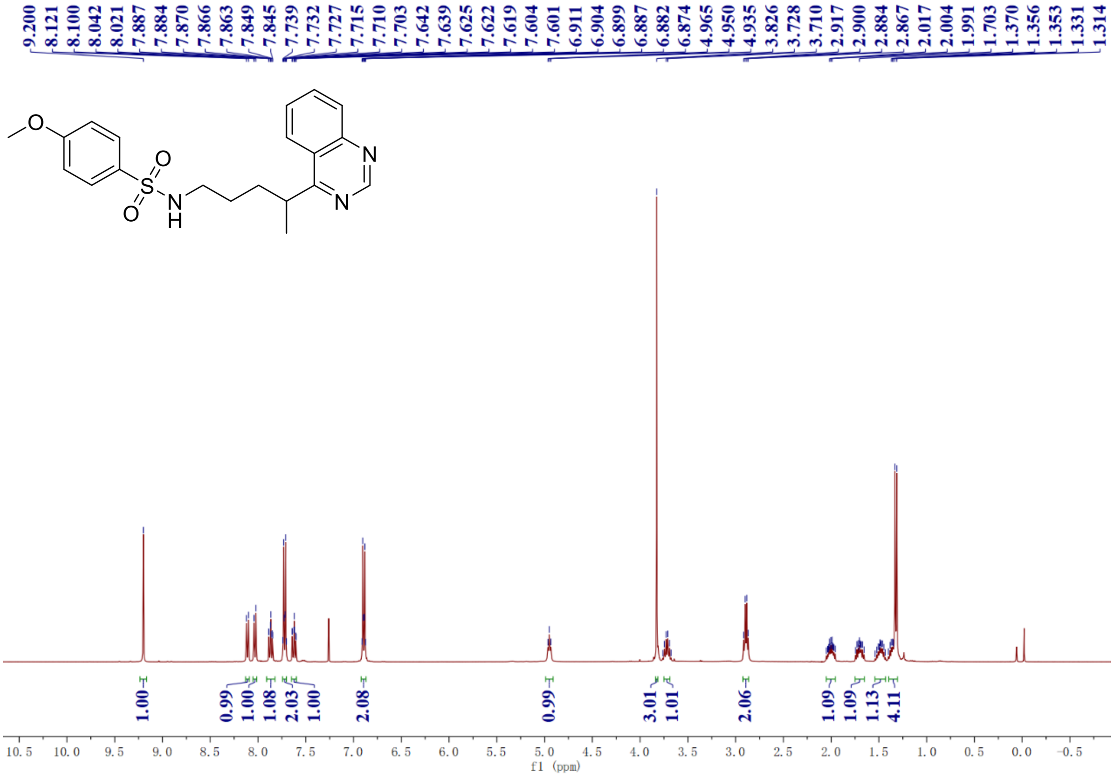

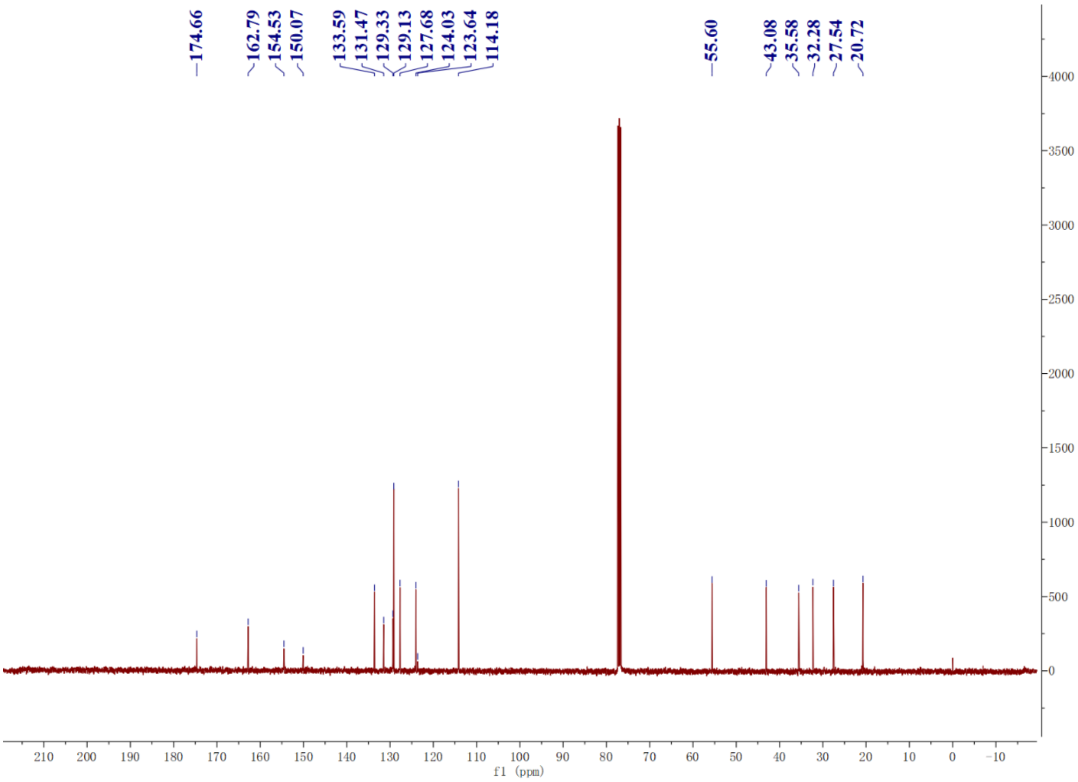

4-methoxy-*N*-(4-(phthalazin-1-yl)pentyl)benzenesulfonamide (27)

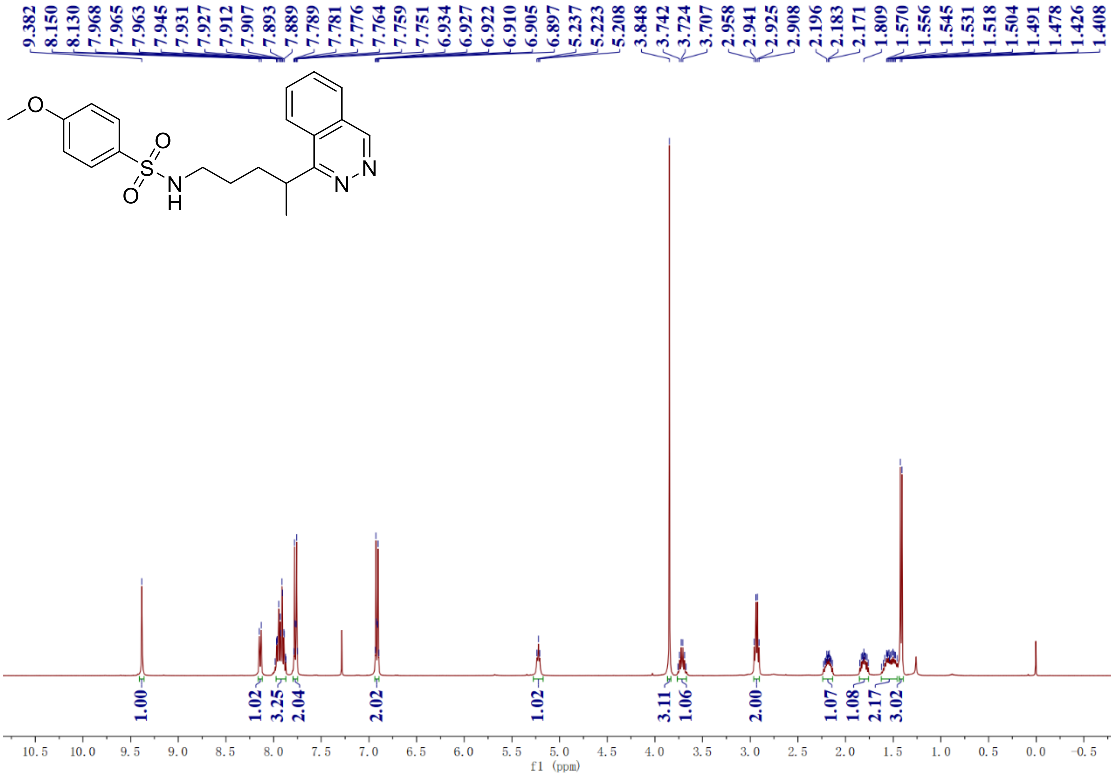

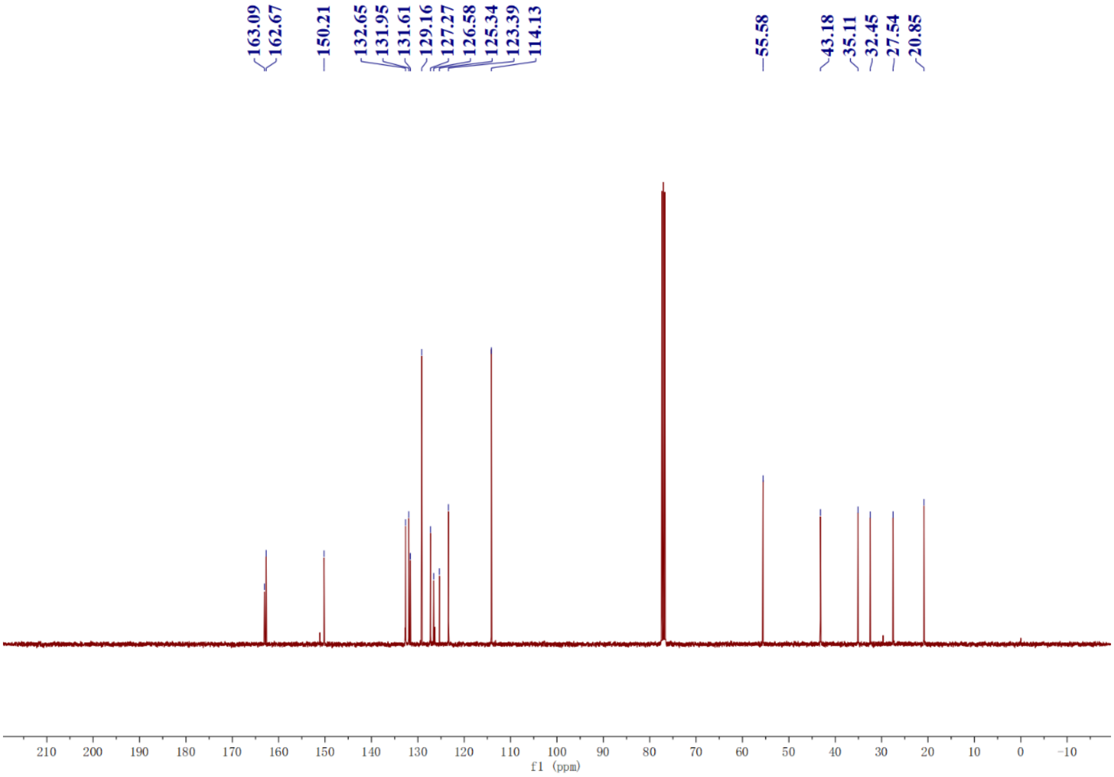

4-methoxy-*N*-(4-(4-phenylpyridin-2-yl)pentyl)benzenesulfonamide (28)

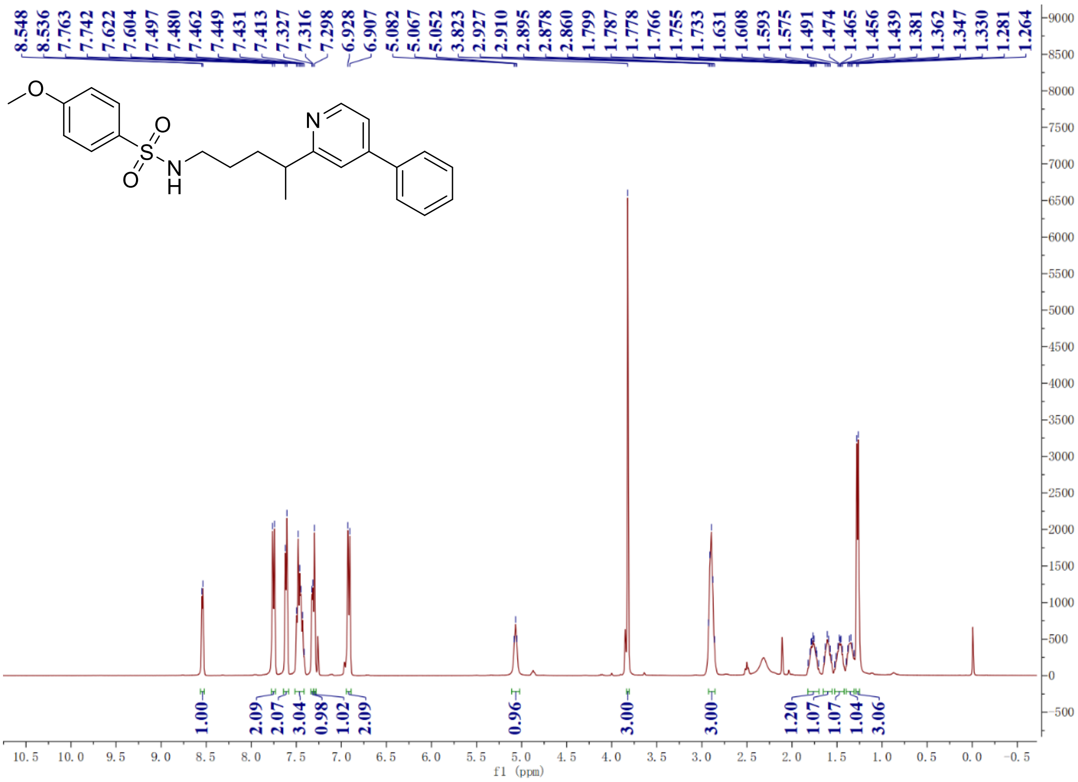

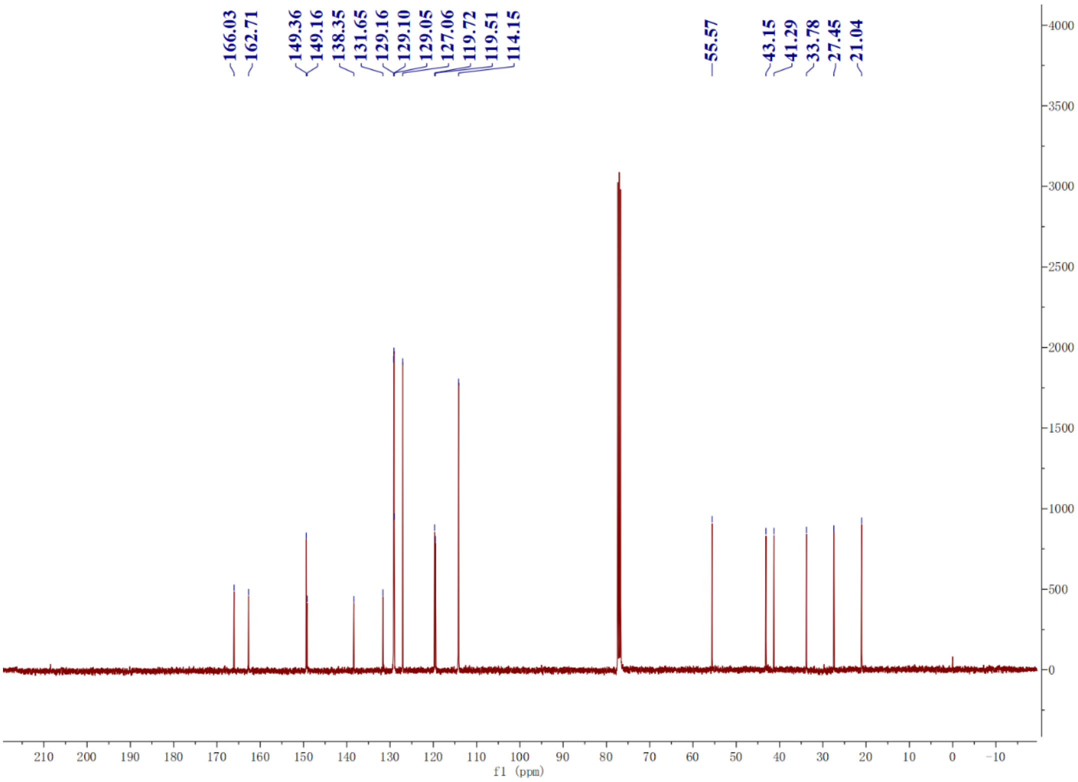

_N_-(4-(4-cyanopyridin-2-yl)pentyl)-4-methoxybenzenesulfonamide (29)

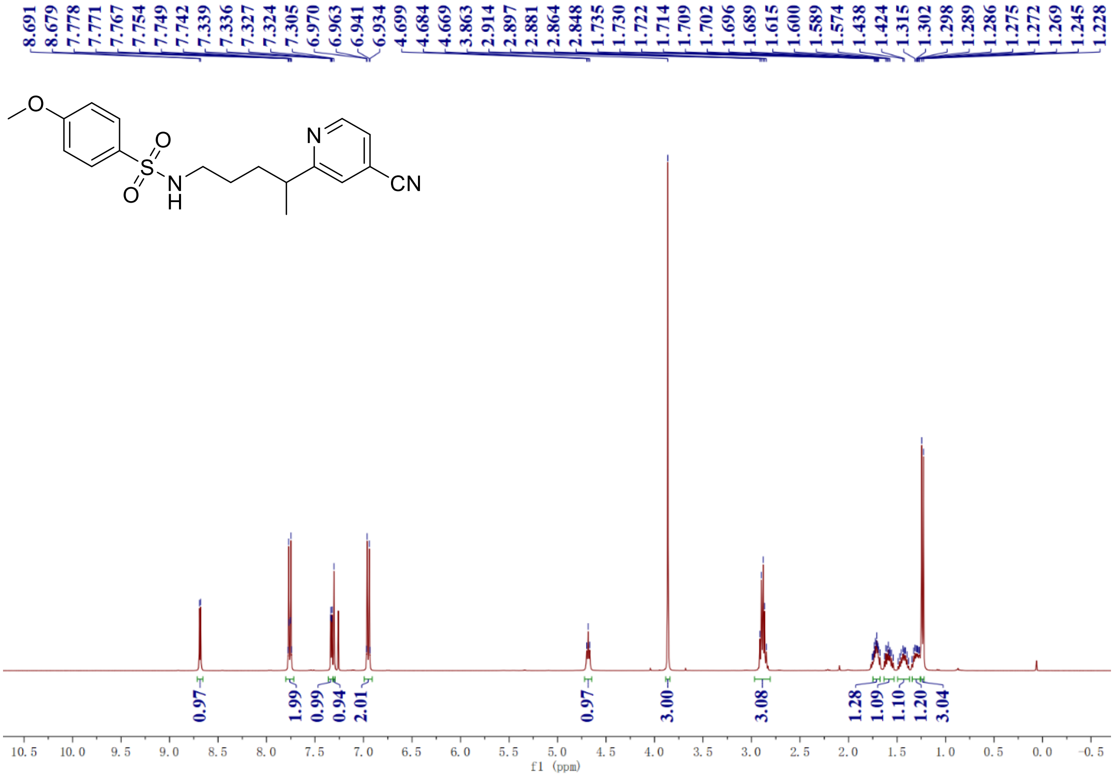

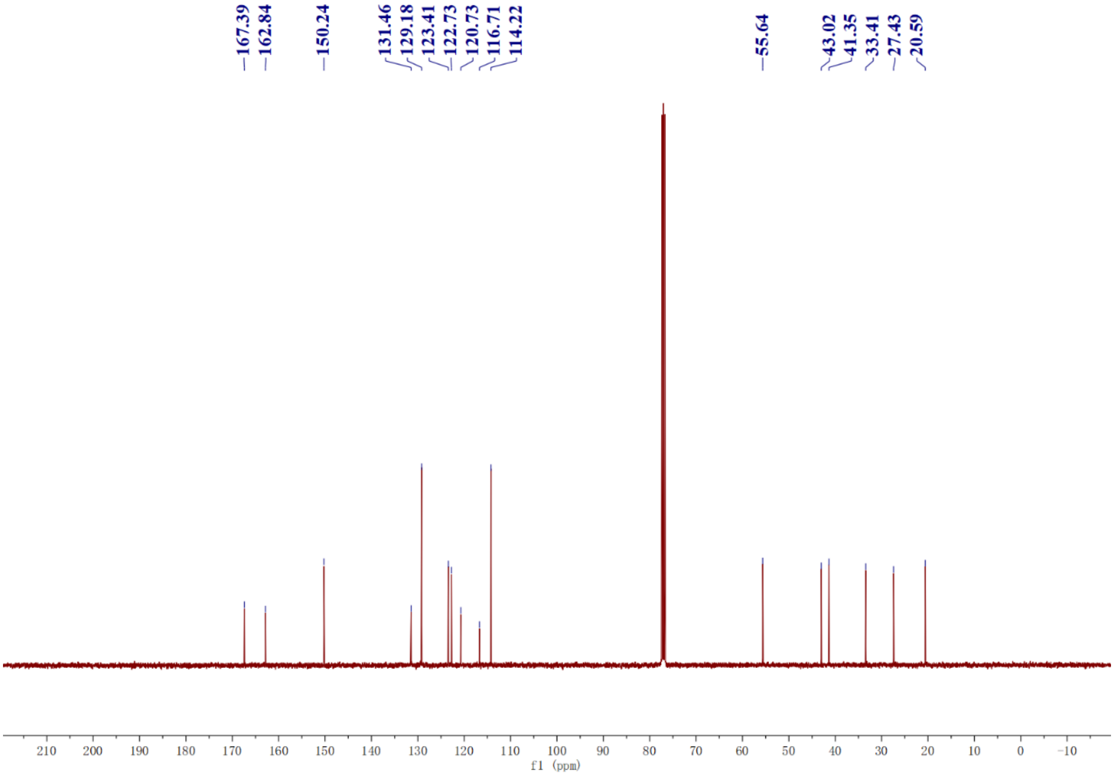

ethyl 2-(5-((4-methoxyphenyl)sulfonamido)pentan-2-yl)-4-methylthiazole-5-carboxylate (30)

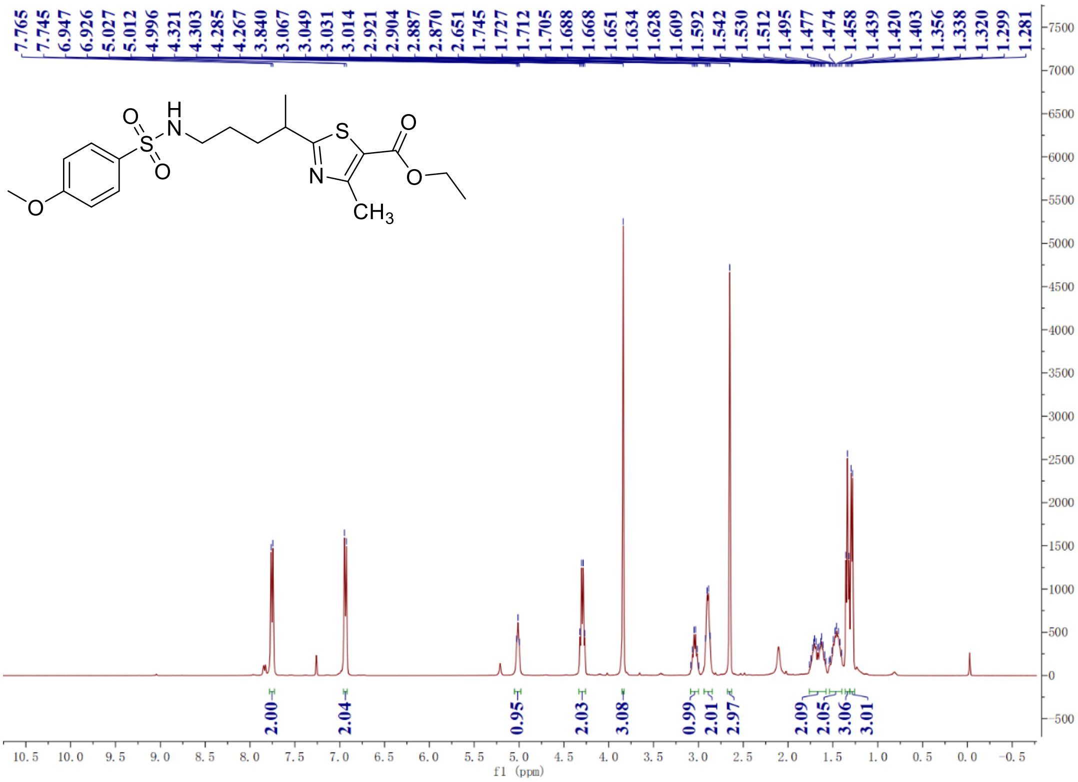

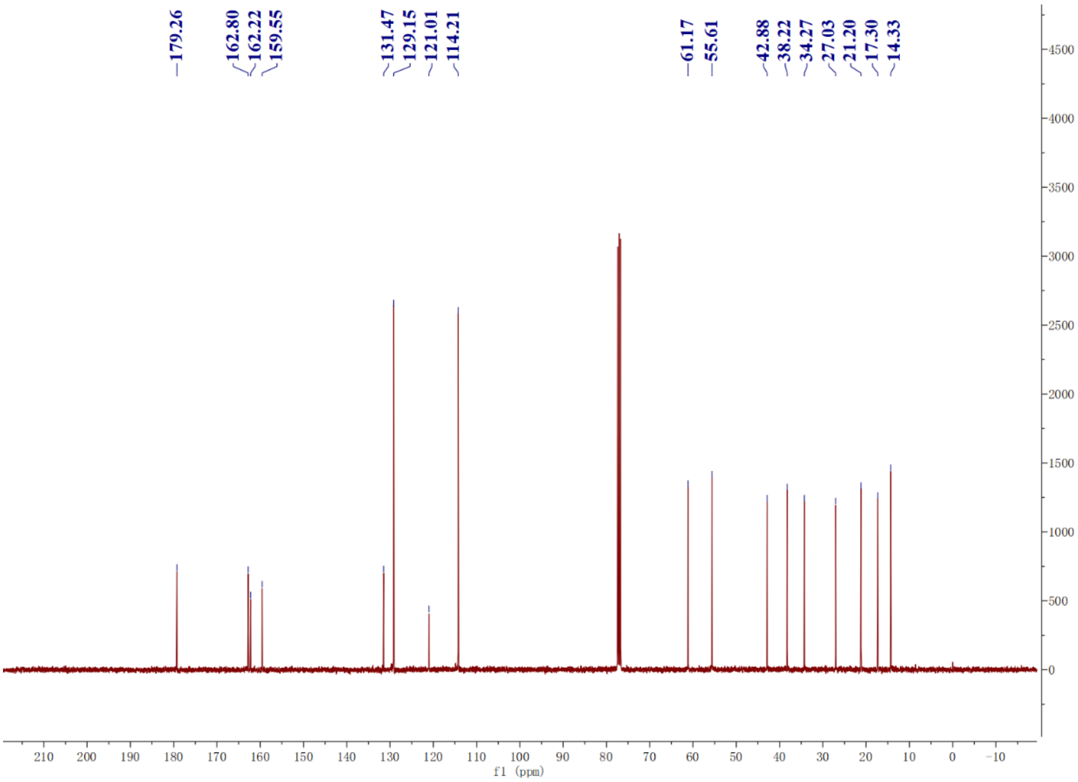

***N*-(4-(5-formylthiazol-2-yl)pentyl)-4-methoxybenzenesulfonamide (31)**

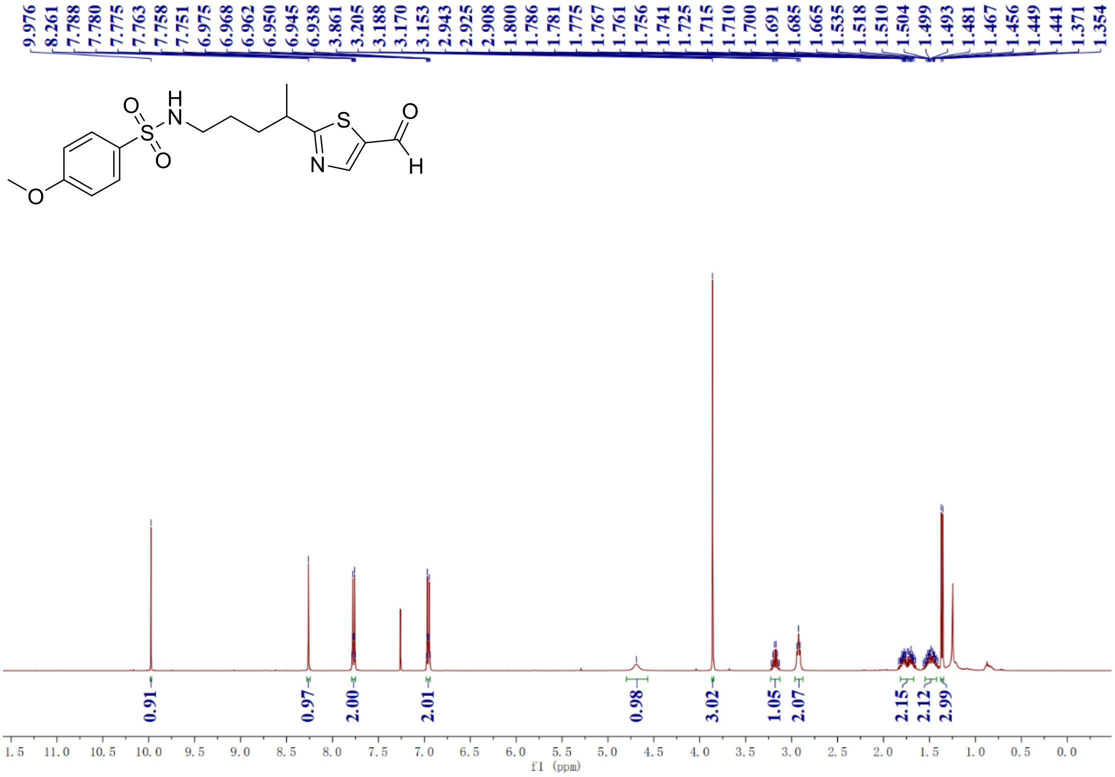

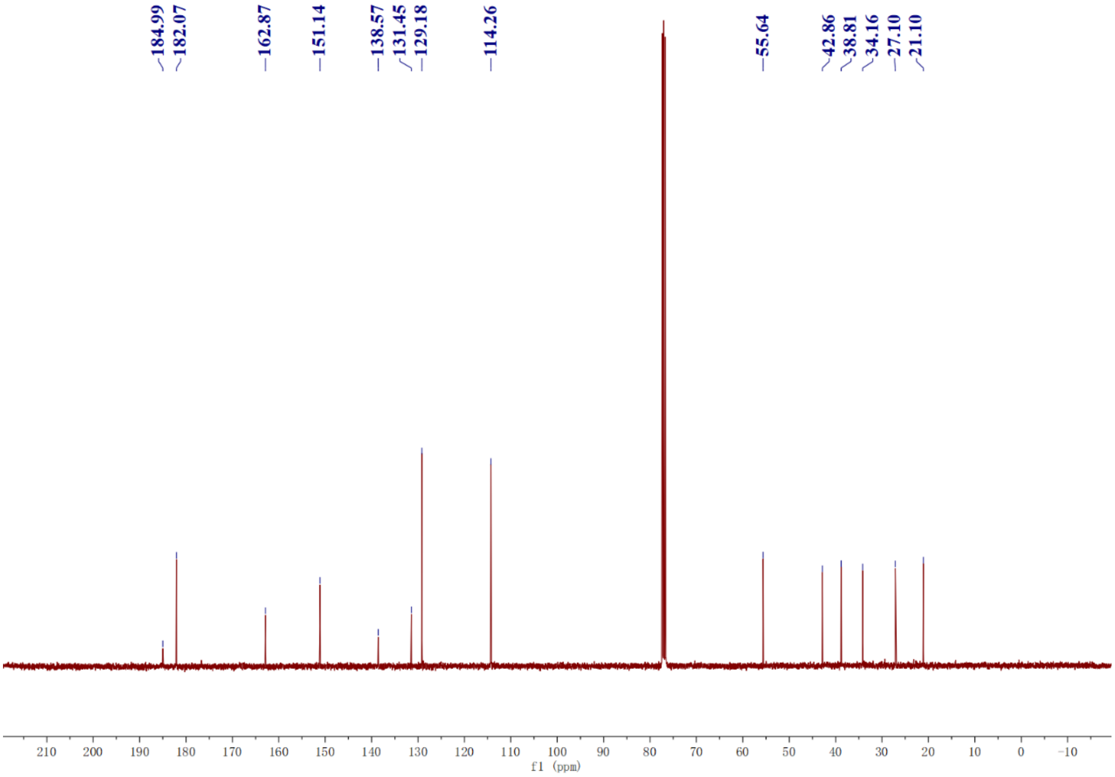

4-methoxy-*N*-(4-(3,5,6-trimethylpyrazin-2-yl)pentyl)benzenesulfonamide (32)

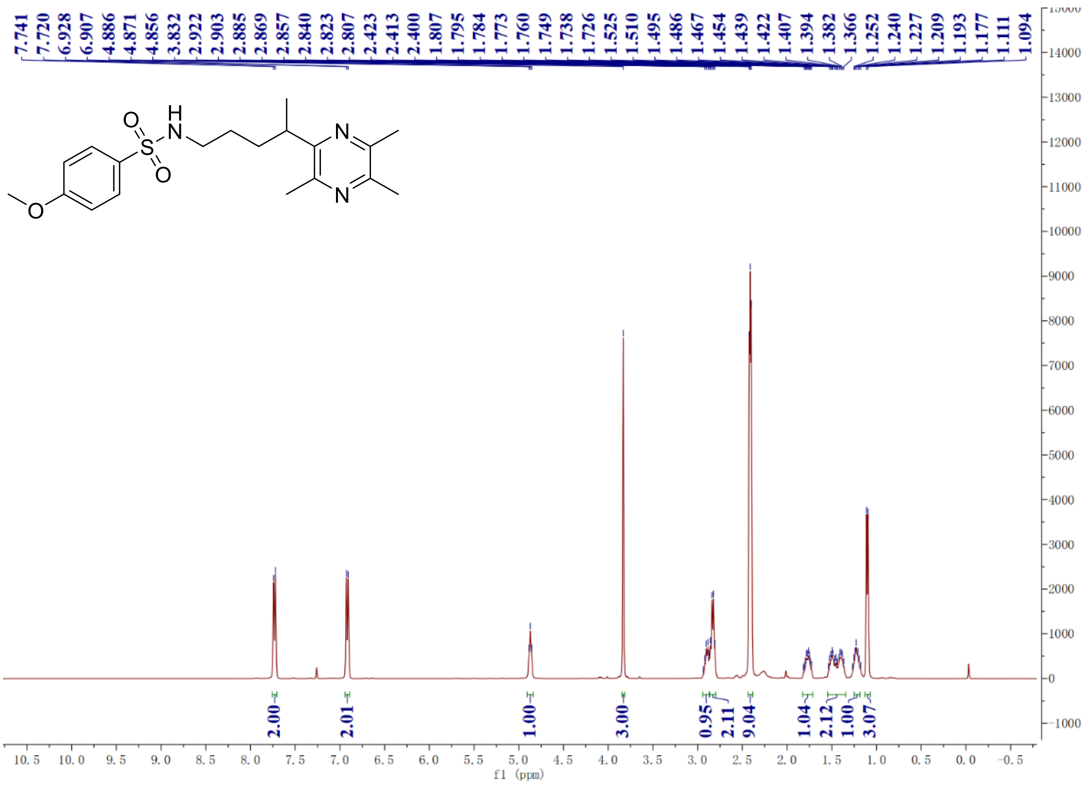

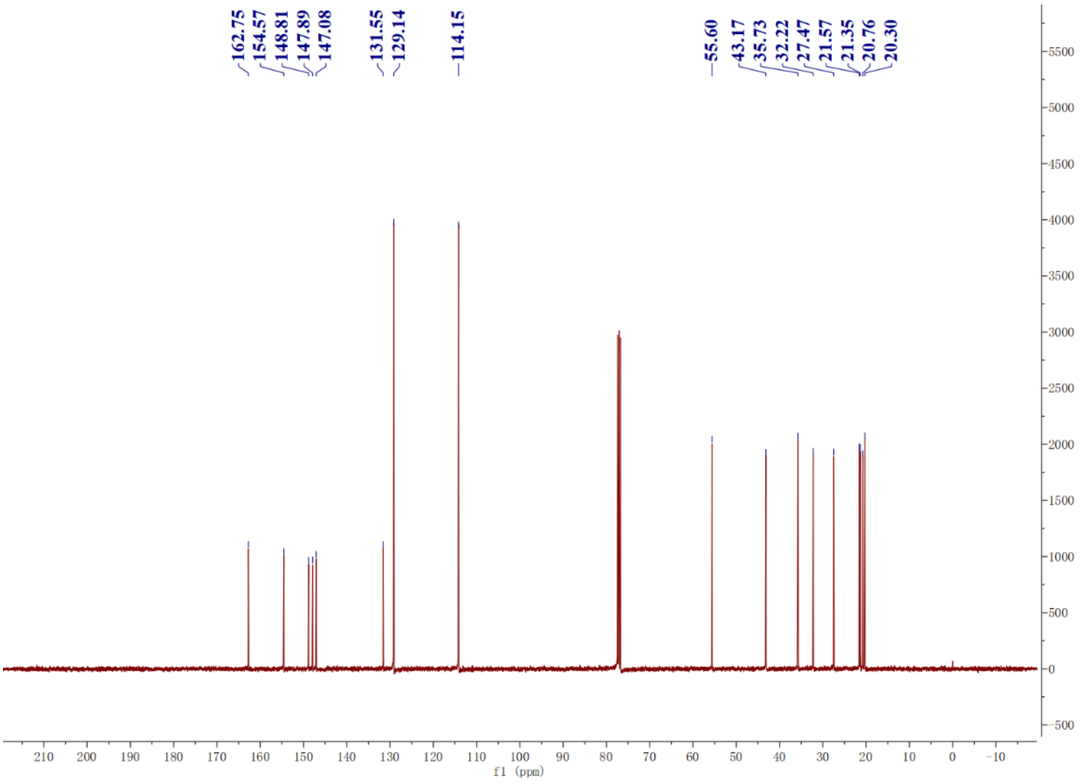

N-(4-(5-acetyl-6-methylpyrazin-2-yl)pentyl)-4-methoxybenzenesulfonamide (33)

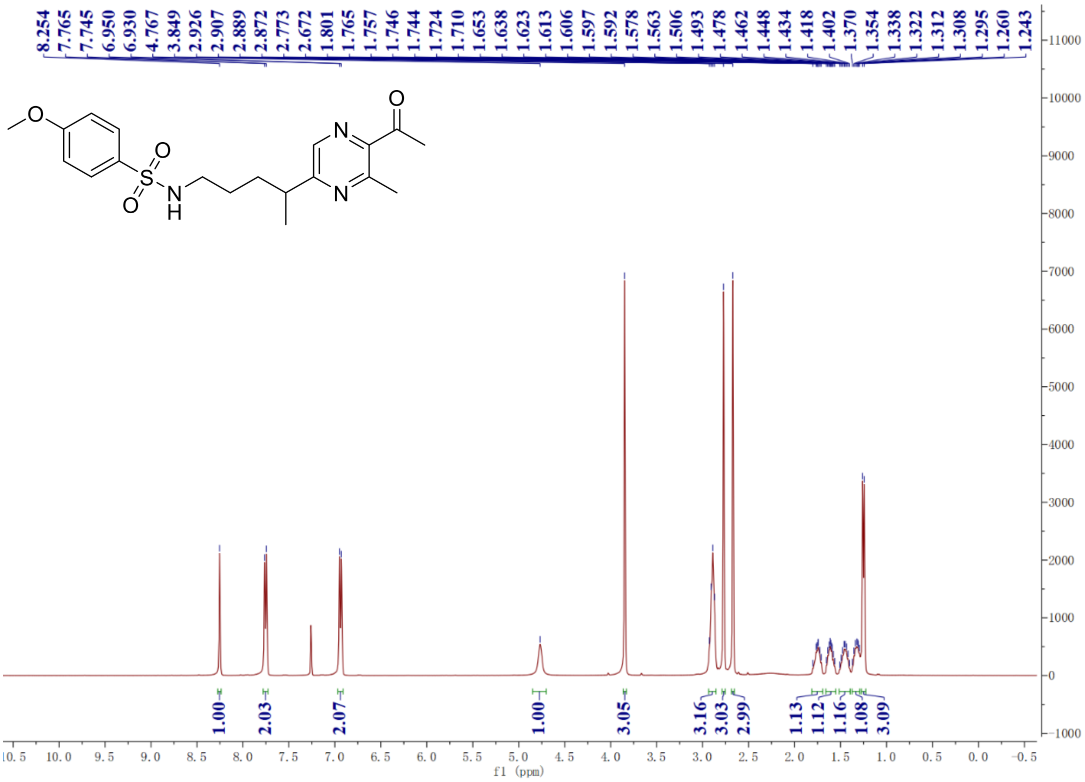

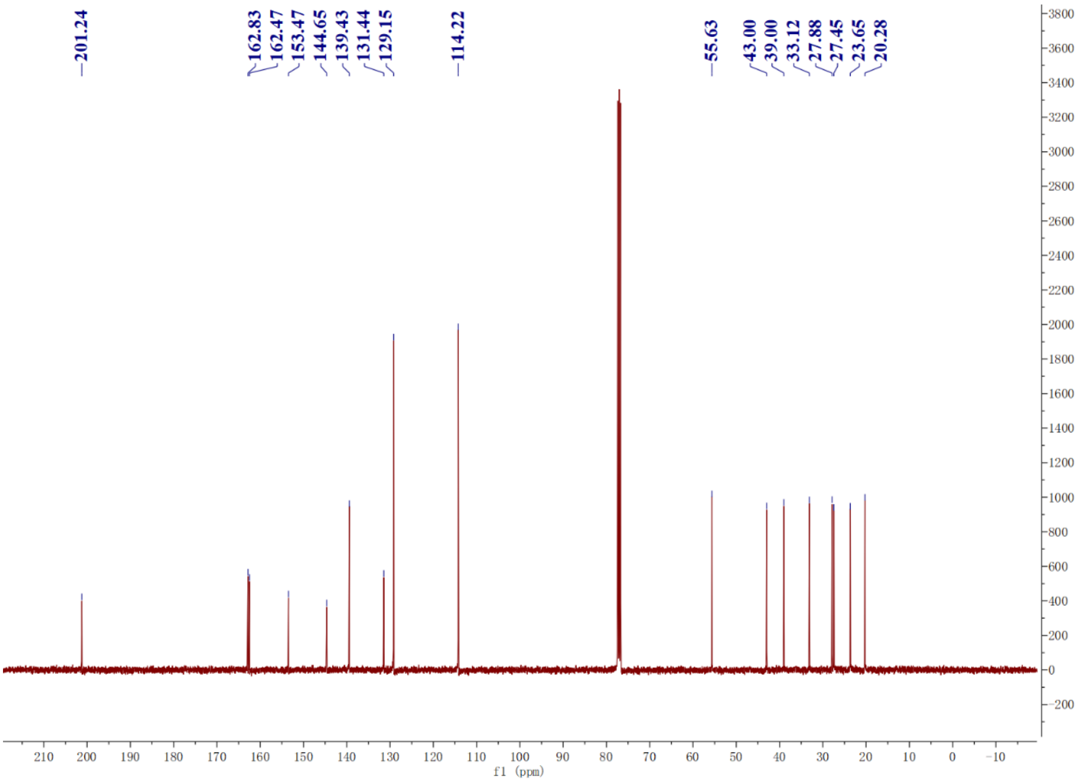

N-(4-(4,6-dimethylpyrimidin-2-yl)pentyl)-4-methoxybenzenesulfonamide (34)

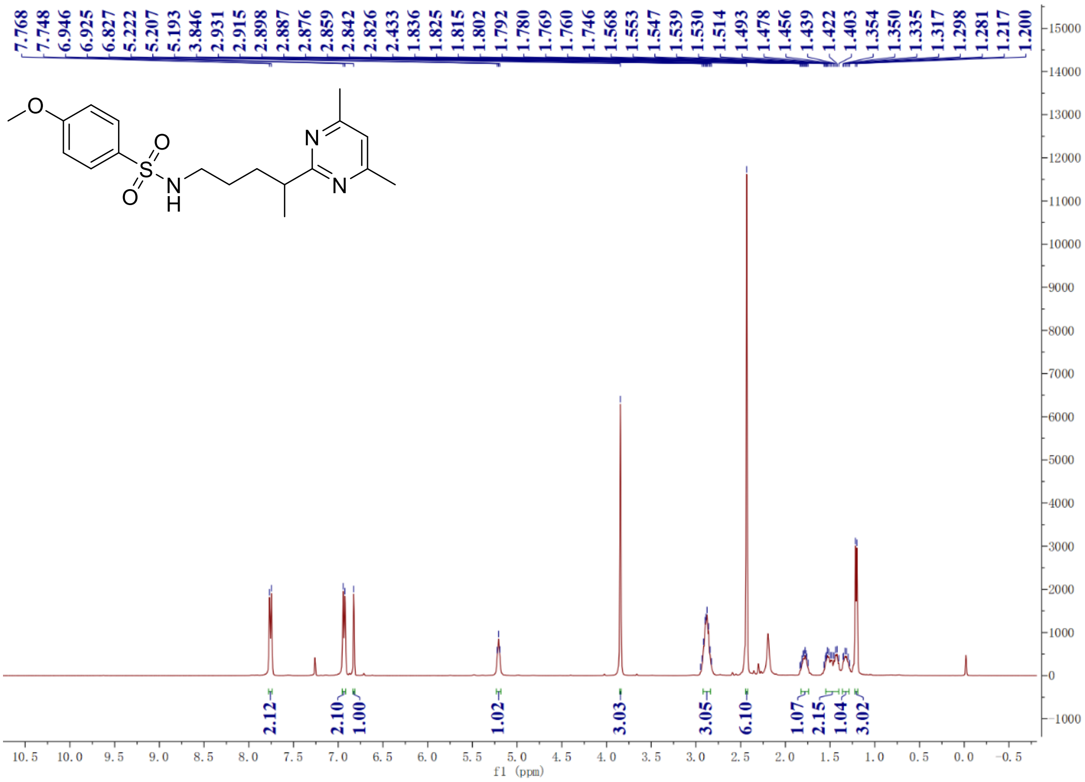

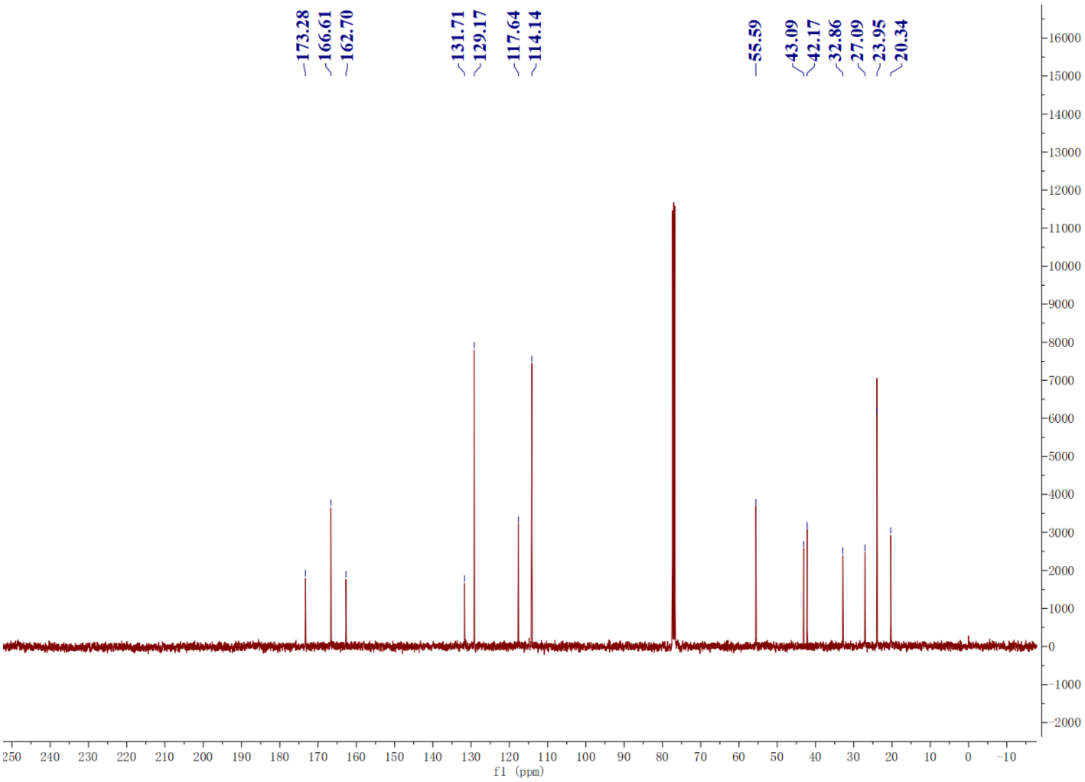

4-methoxy-*N*-(4-(pyridazin-3-yl)pentyl)benzenesulfonamide (35)

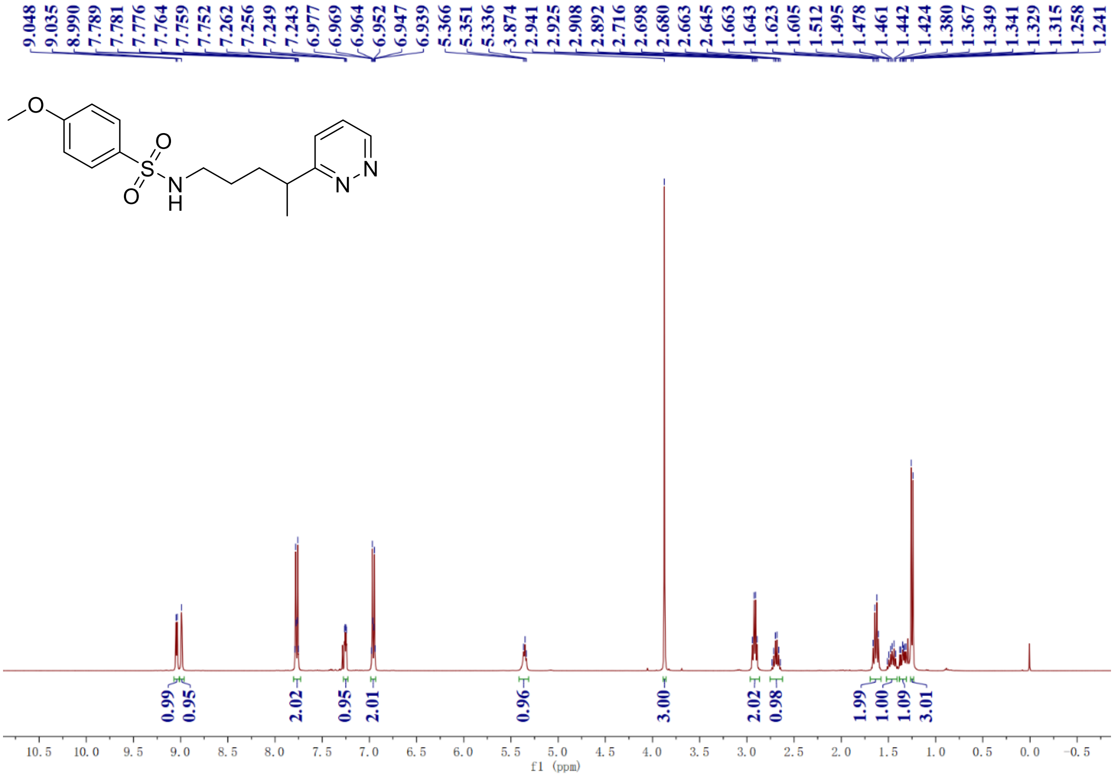

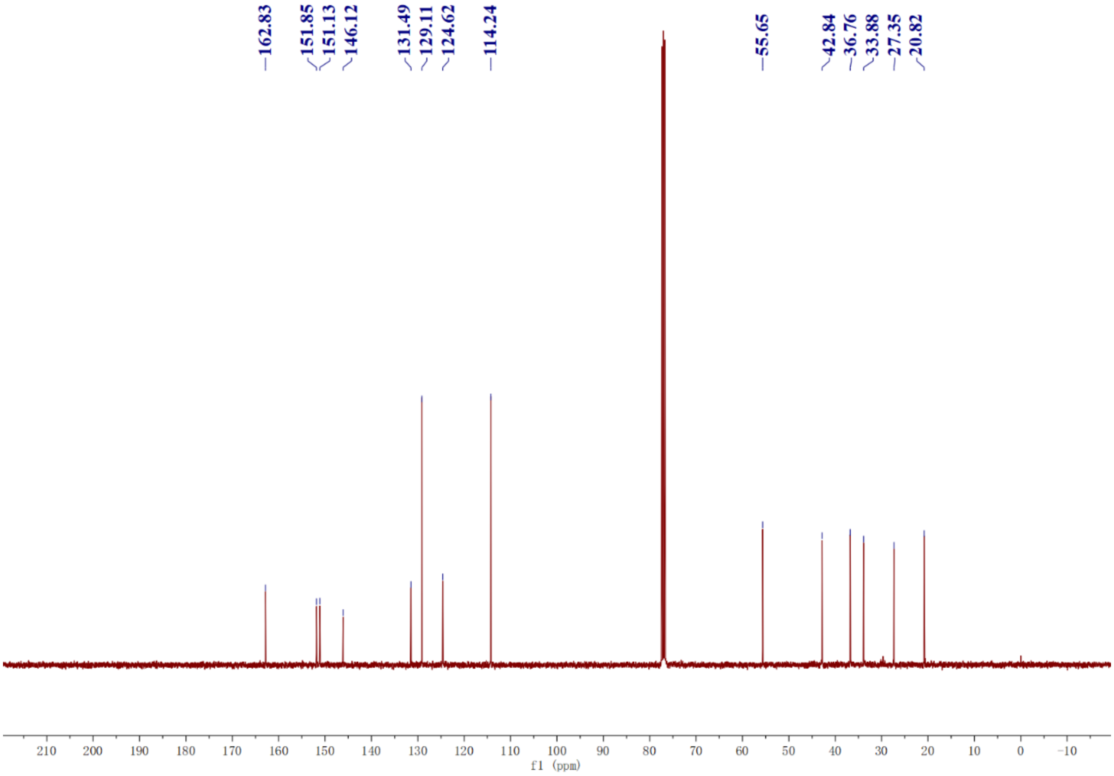

***N*-(4-(acridin-9-yl)pentyl)-4-methoxybenzenesulfonamide (36)**

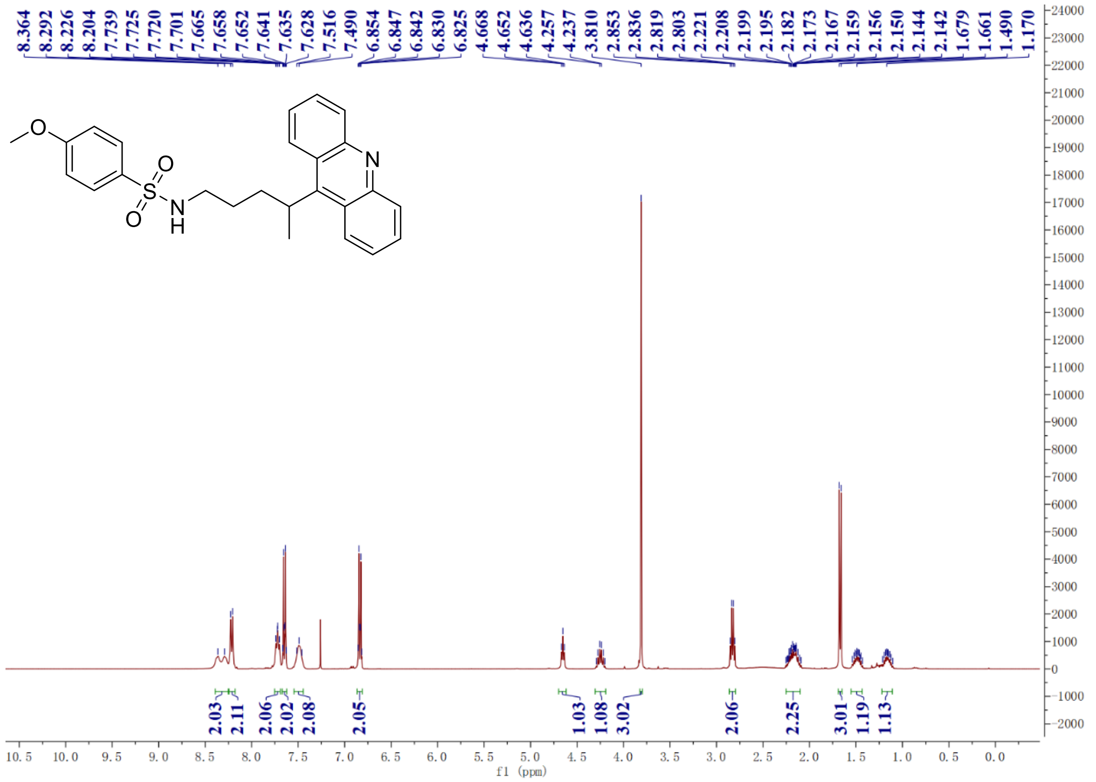

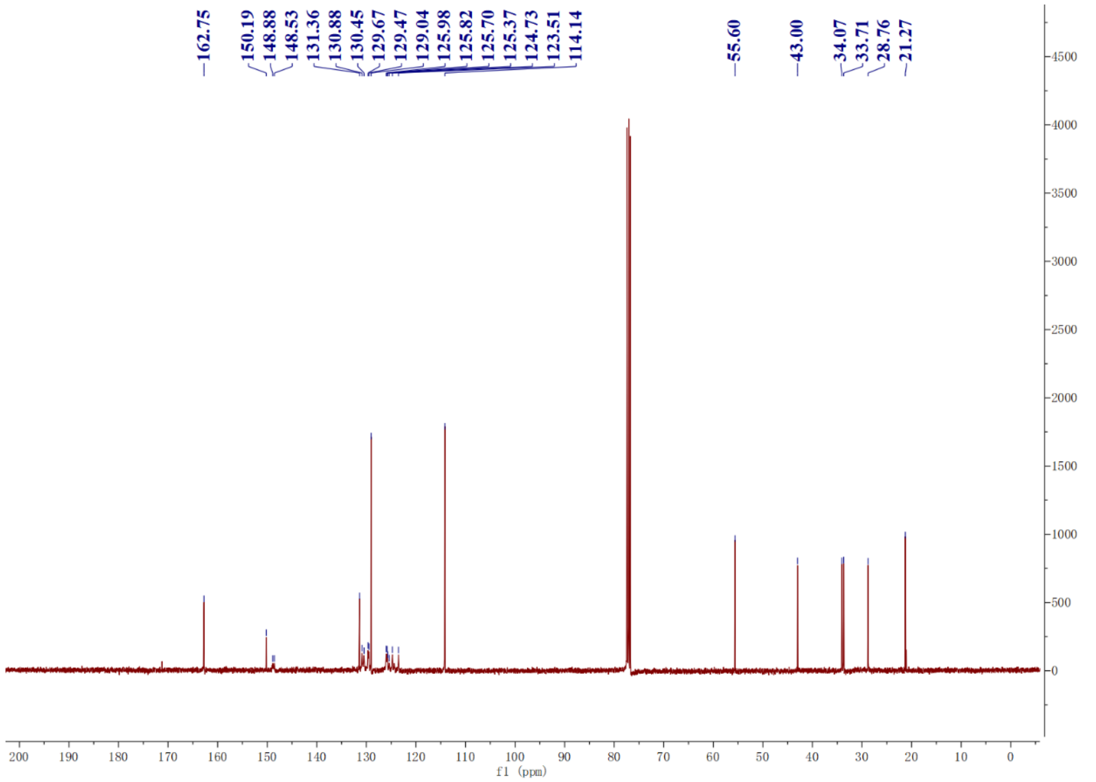

***N*-(4-(3-chloroquinoxalin-2-yl)pentyl)-4-methoxybenzenesulfonamide (37)**

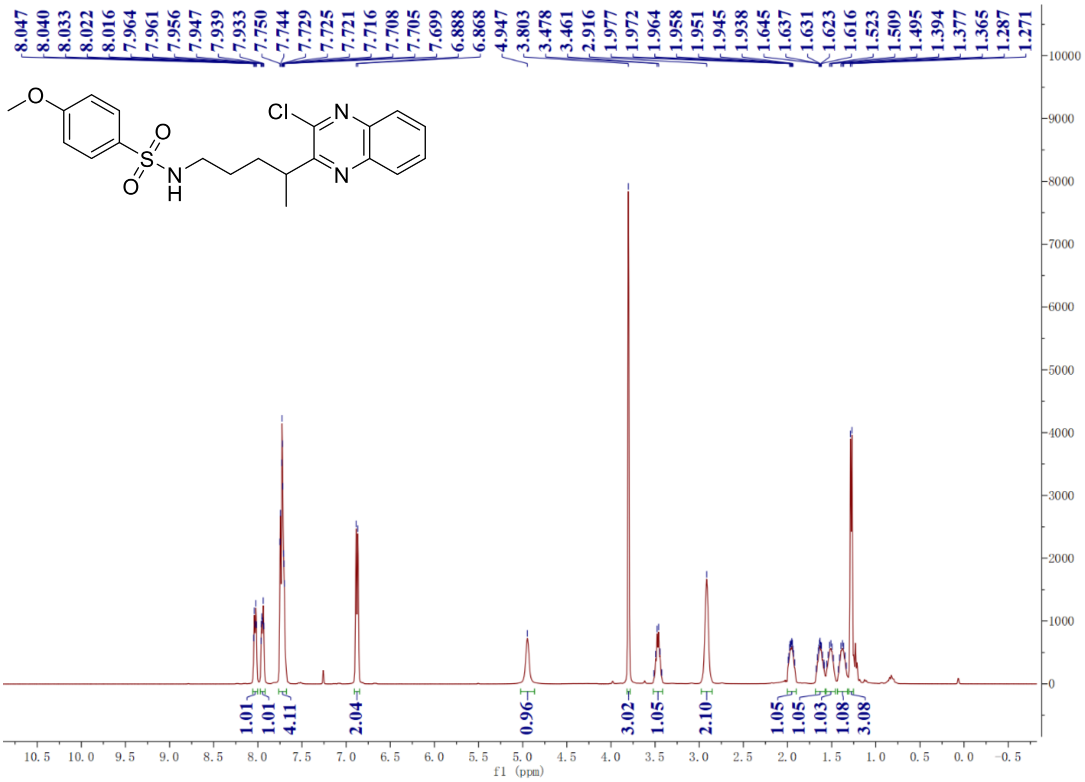

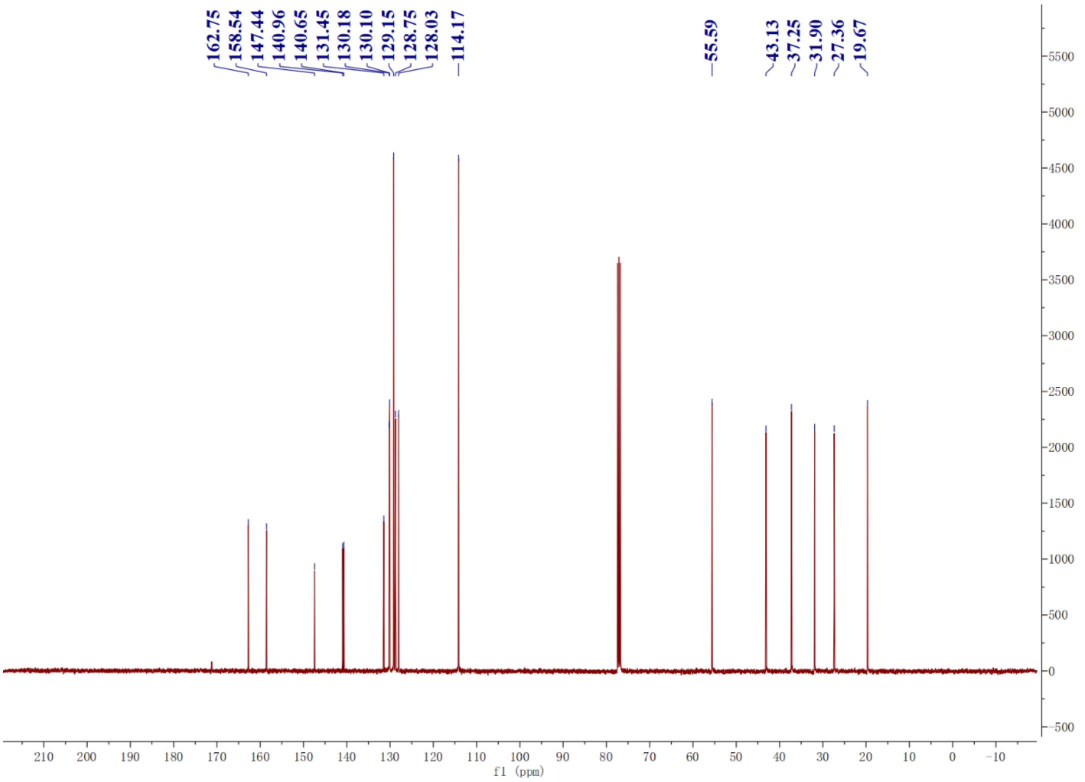

4-methoxy-*N*-(4-(4-oxochroman-2-yl)pentyl)benzenesulfonamide (38)

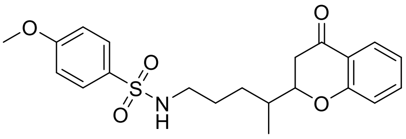

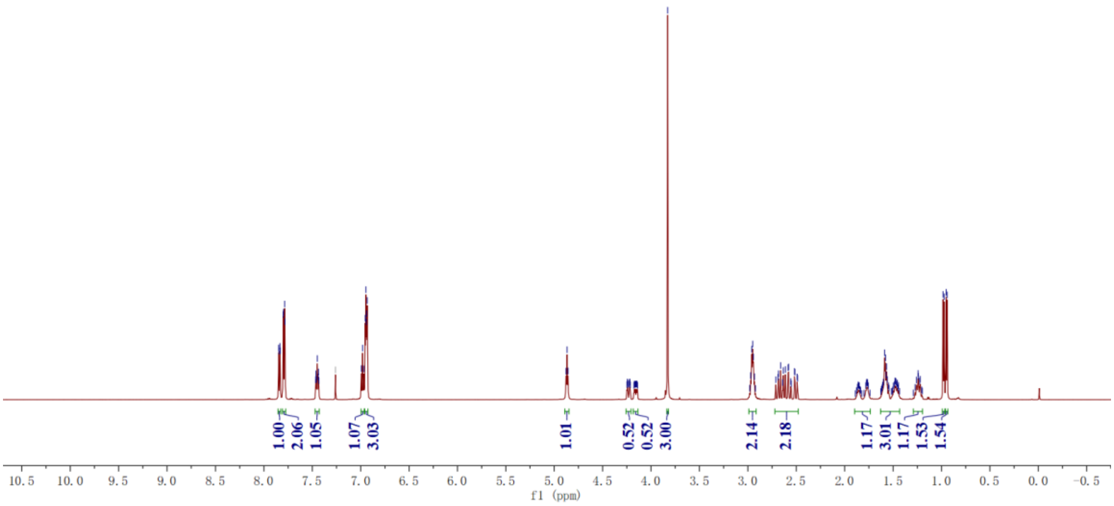

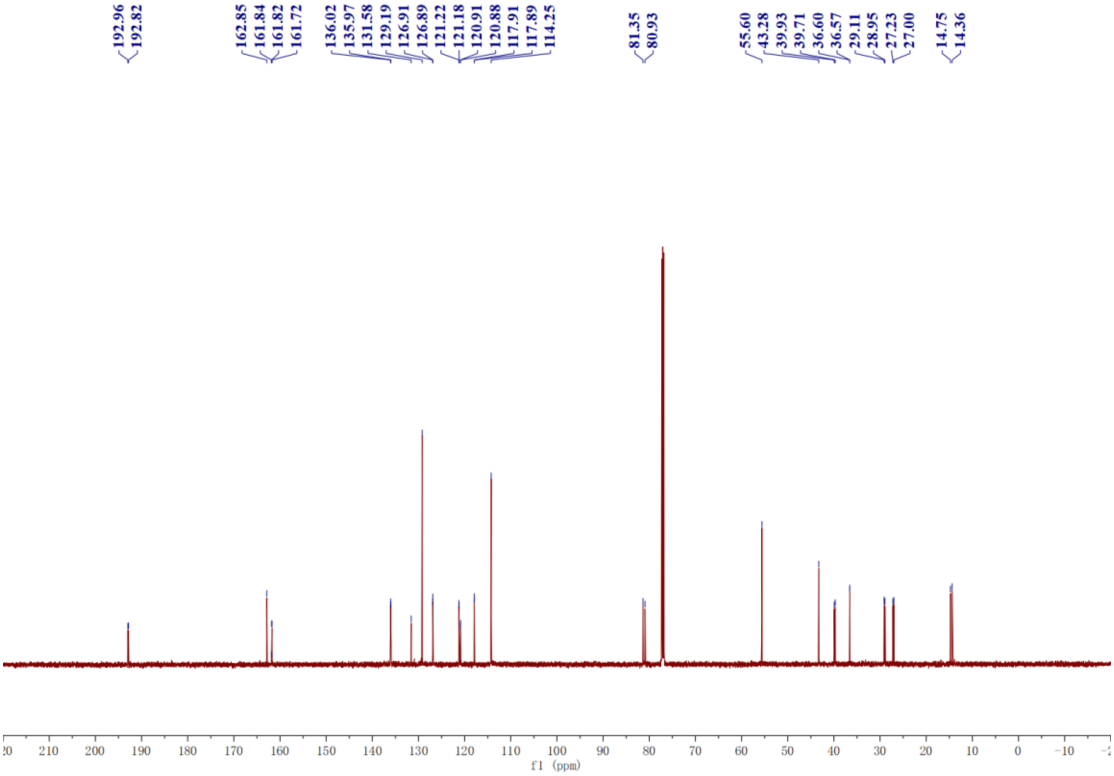

***N*-(4-(6-chlorobenzo[*d*]thiazol-2-yl)hexyl)-4-methoxybenzenesulfonamide (39)**

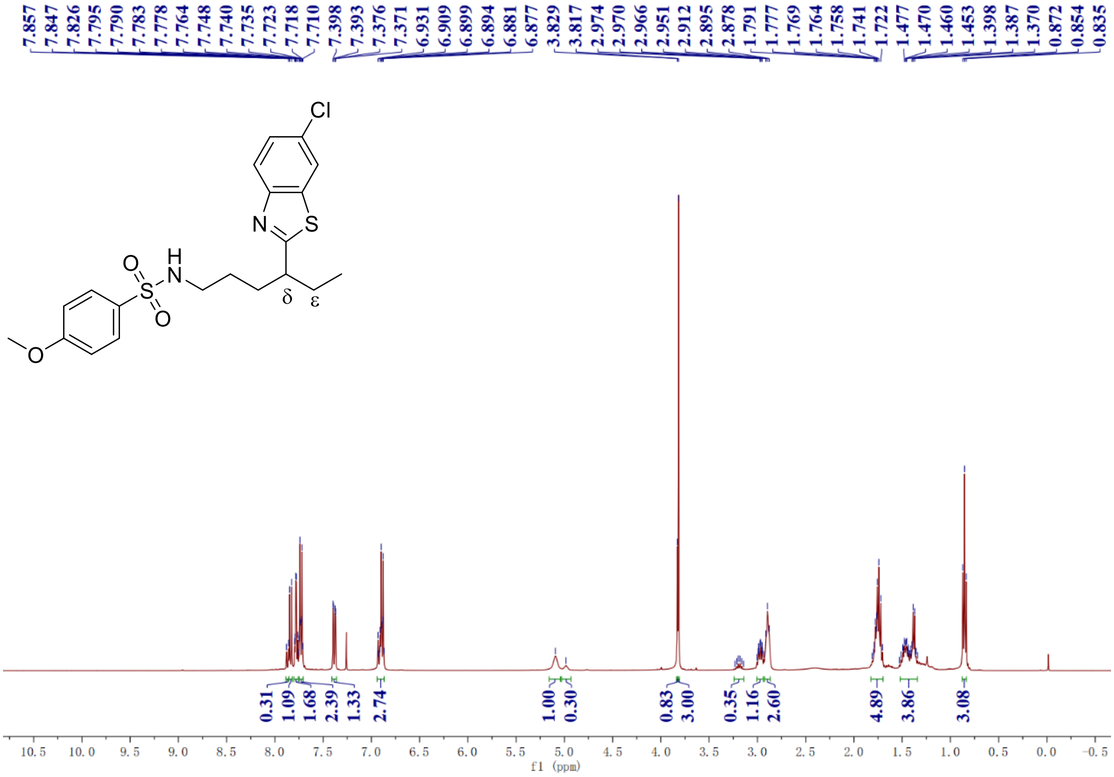

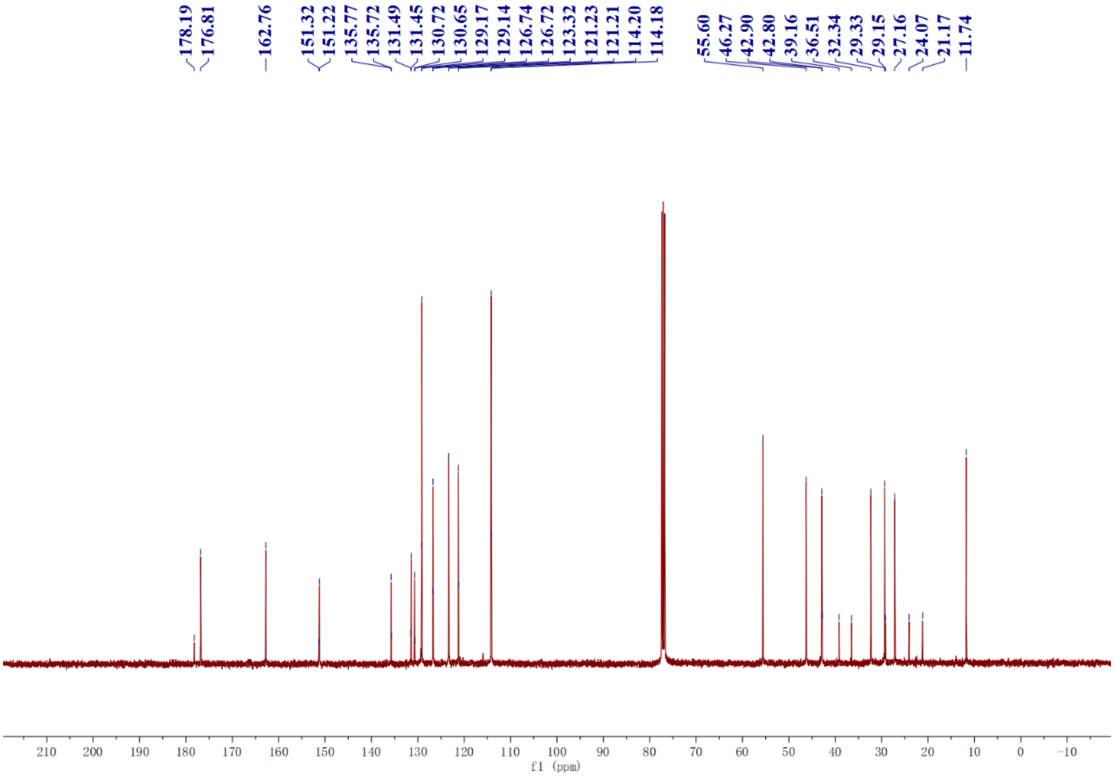

N-(4-(6-chlorobenzo[d]thiazol-2-yl)heptyl)-4-methoxybenzenesulfonamide (40)

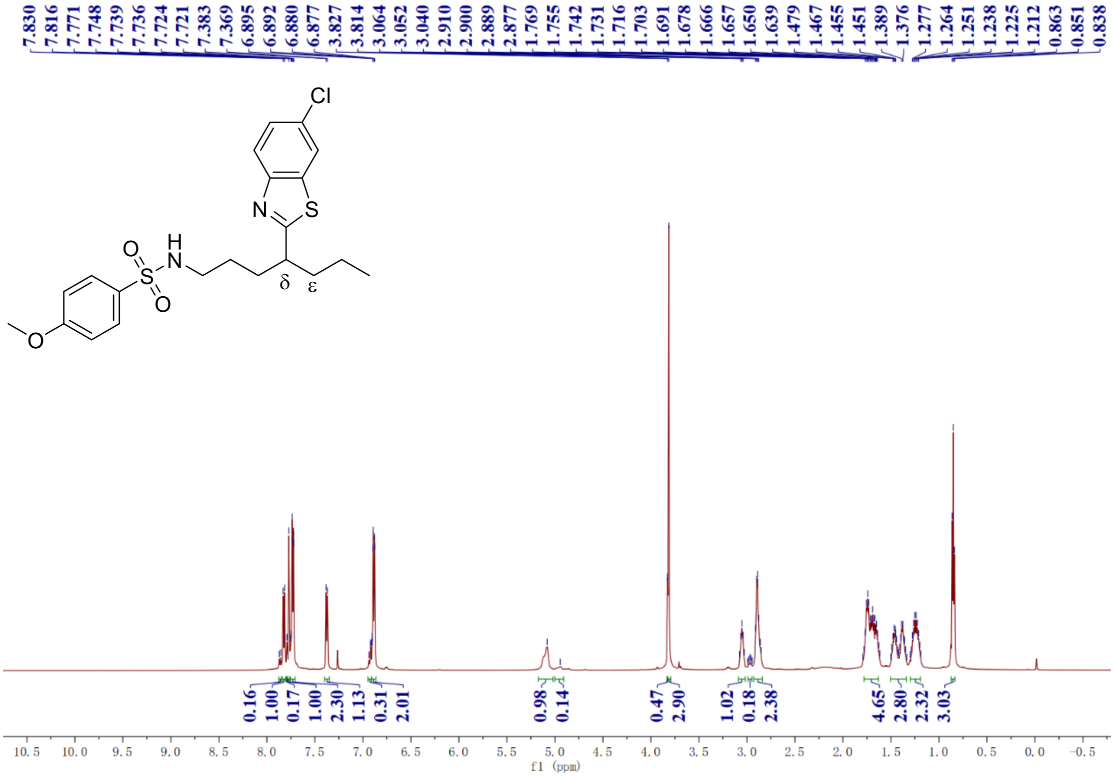

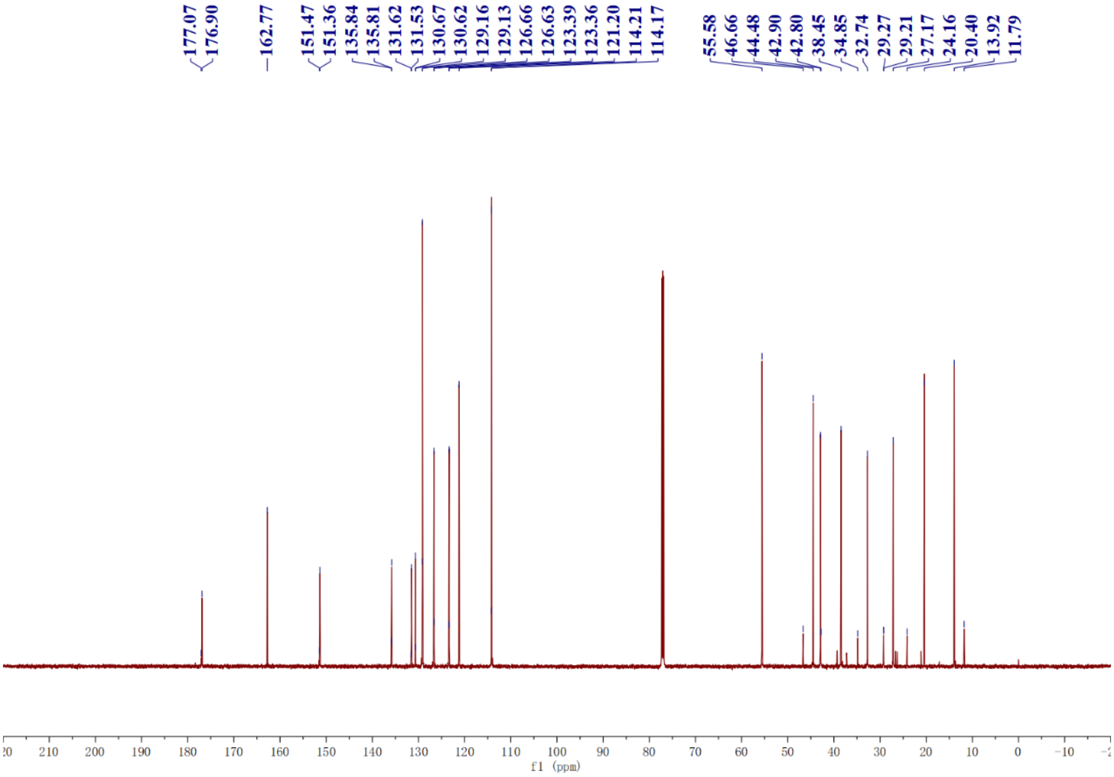

N-(4-(6-chlorobenzo[d]thiazol-2-yl)octyl)-4-methoxybenzenesulfonamide (41)

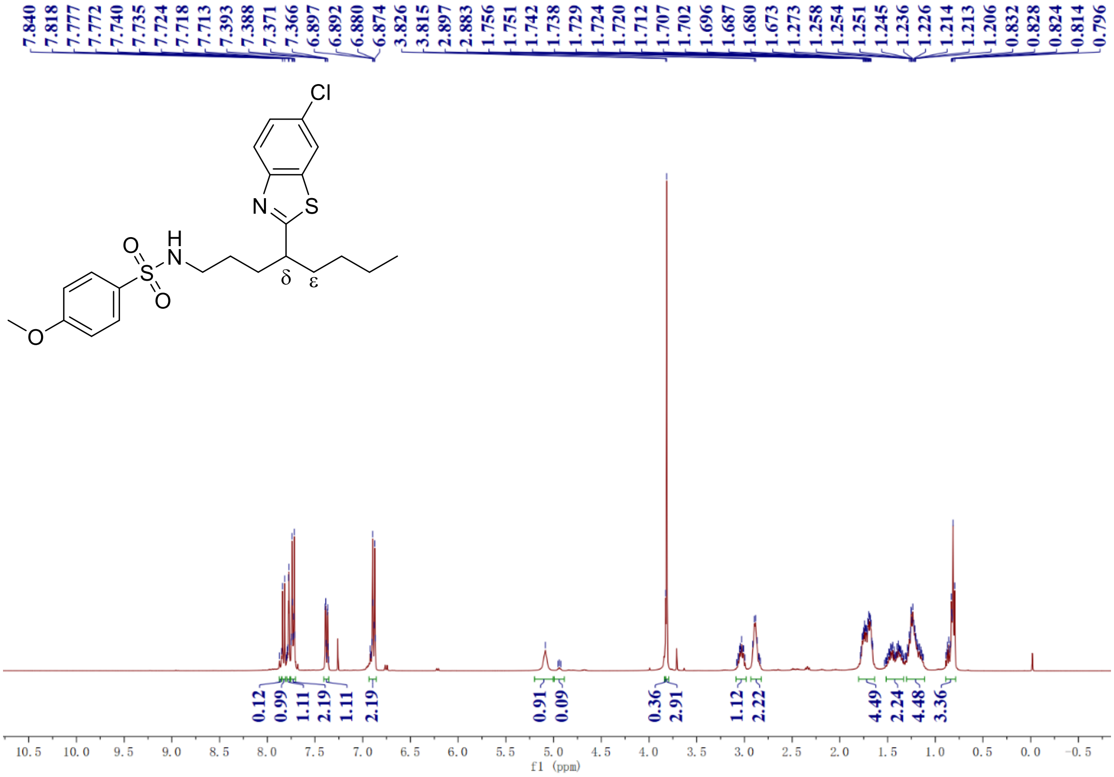

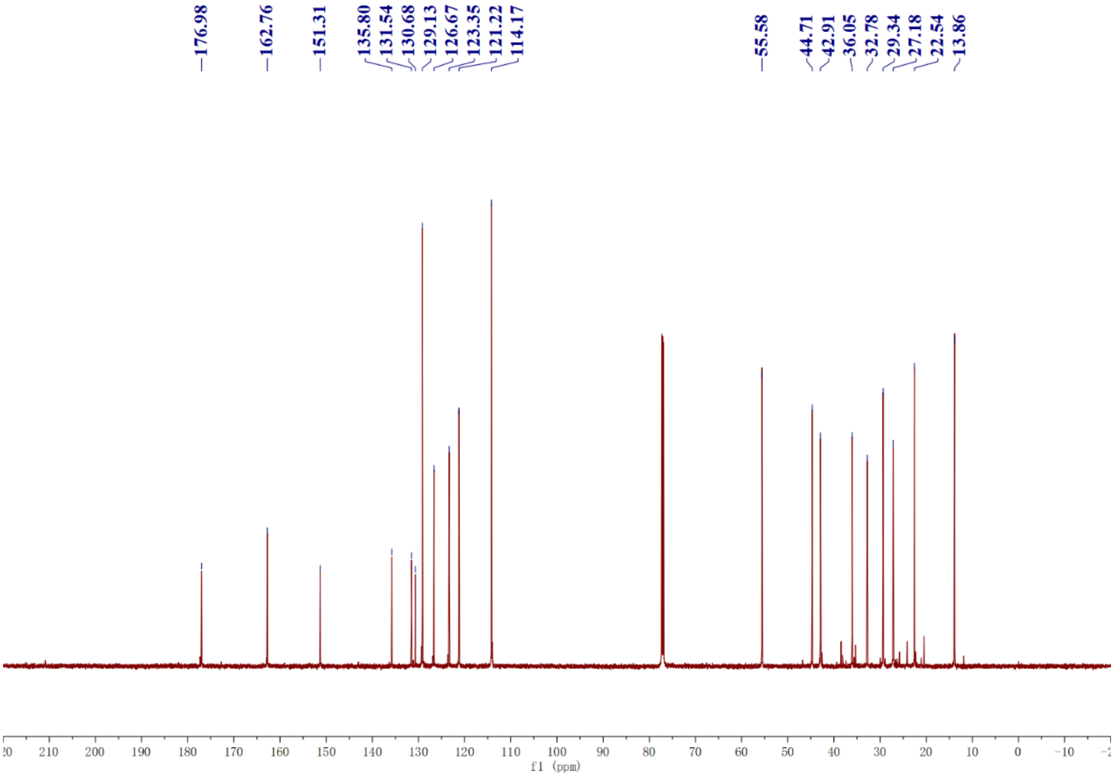

_N_-(2,2-dimethyl-4-(2-phenylquinolin-4-yl)hexyl)-4-methoxybenzenesulfonamide (42)

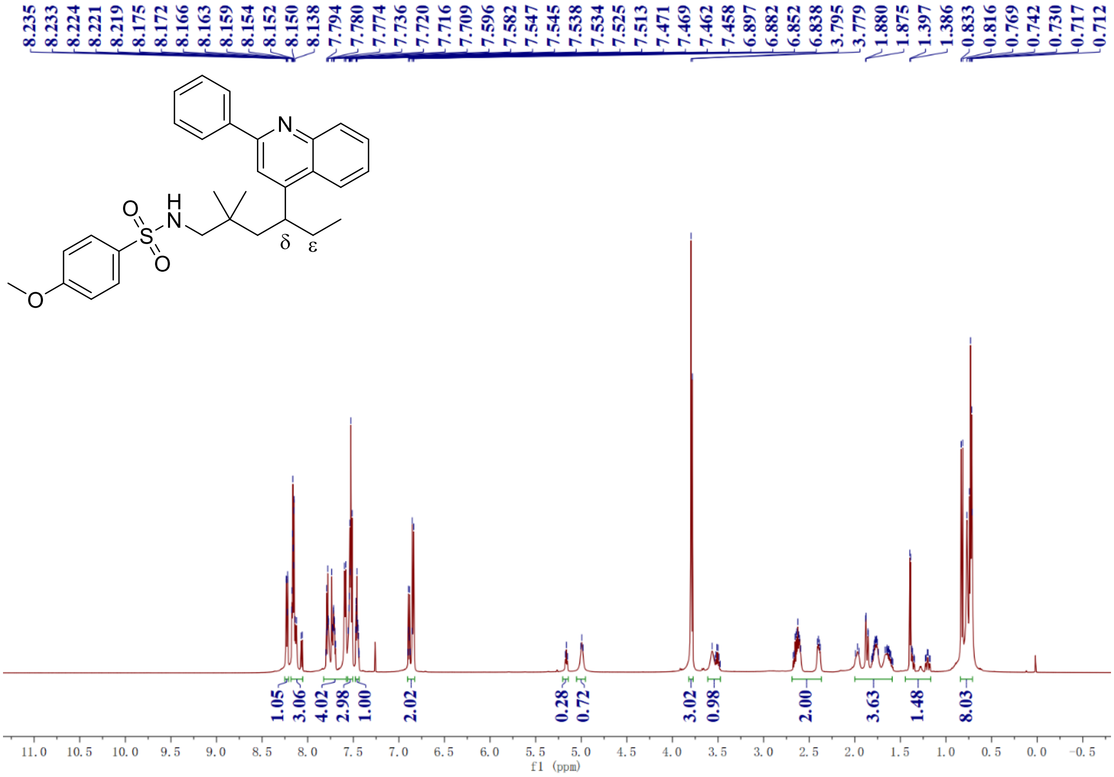

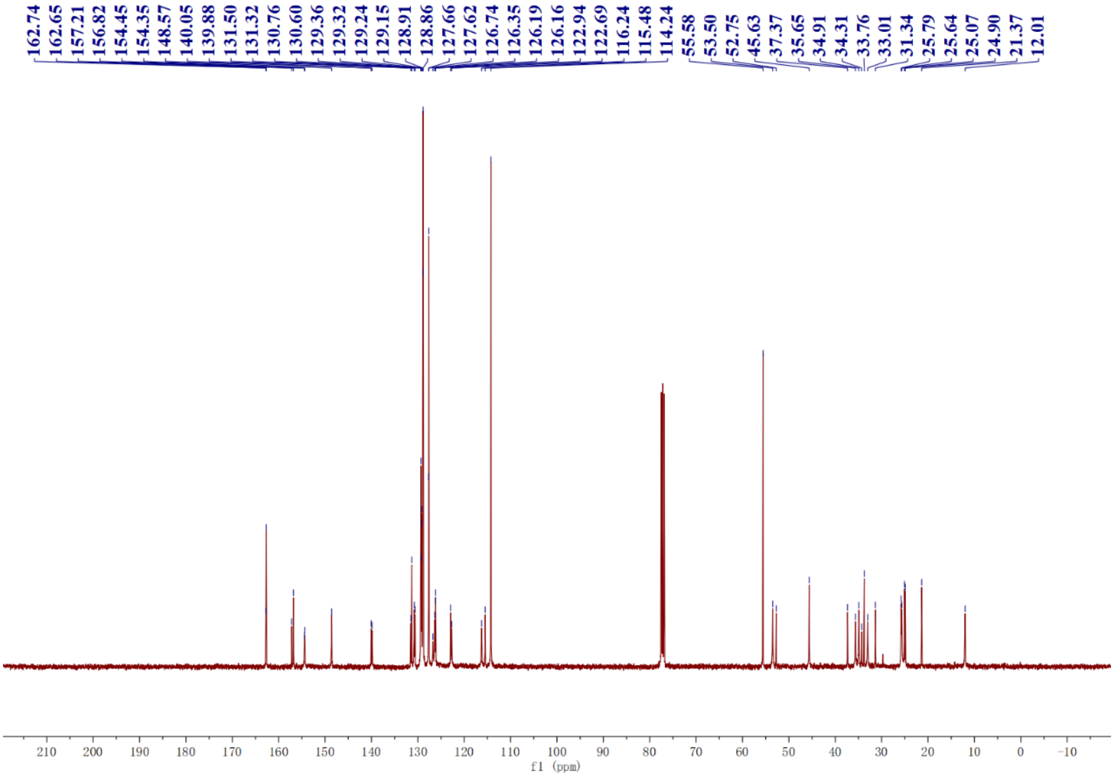

_N_-(4-(6-chlorobenzo[_d_]thiazol-2-yl)-2-ethylhexyl)-4-methoxybenzenesulfonamide (43)

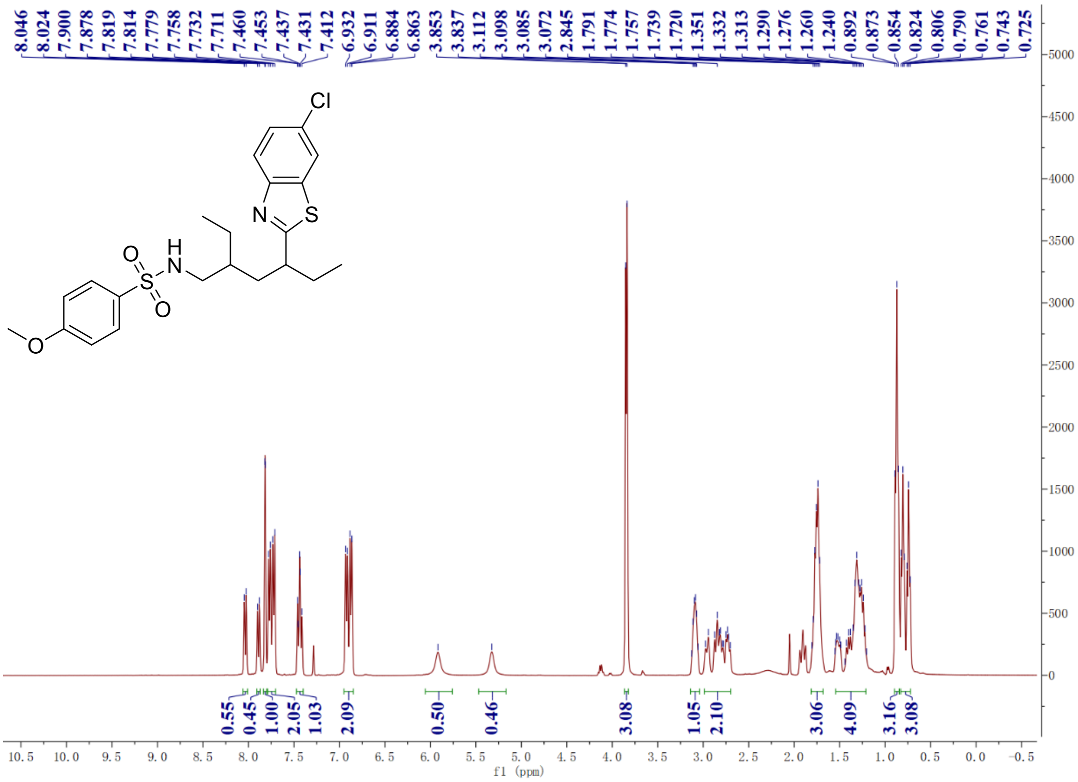

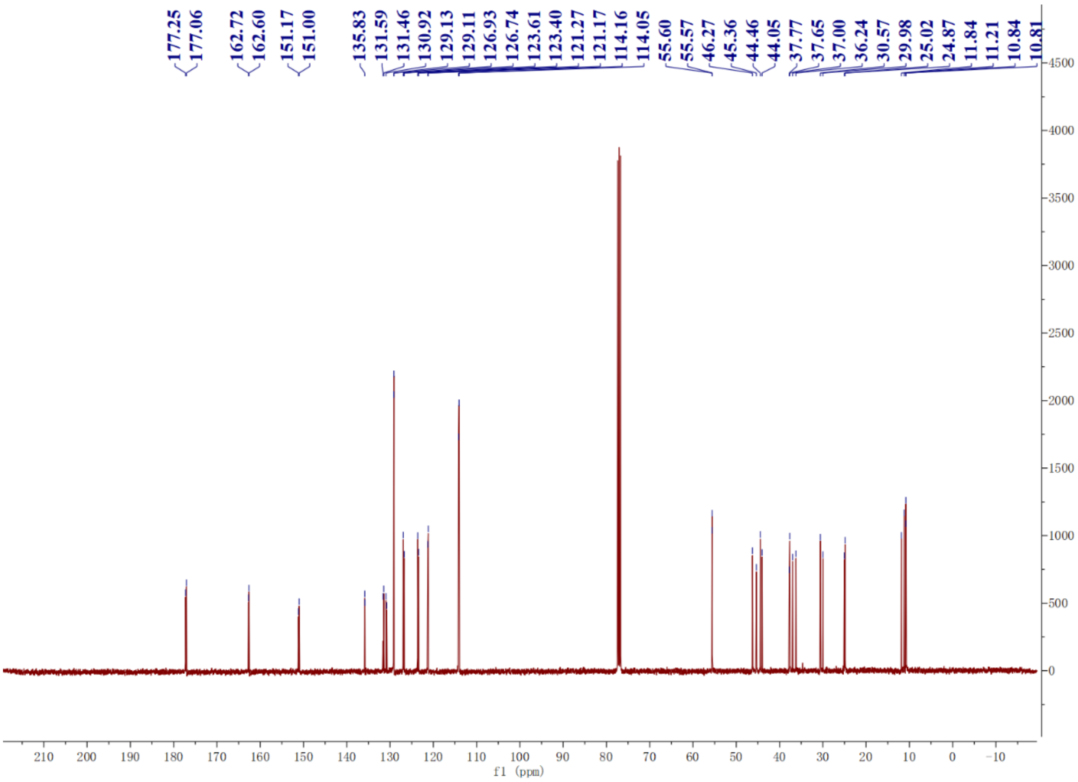

4-methoxy-*N*-(5-methyl-4-(2-phenylquinolin-4-yl)hexyl)benzenesulfonamide (44)

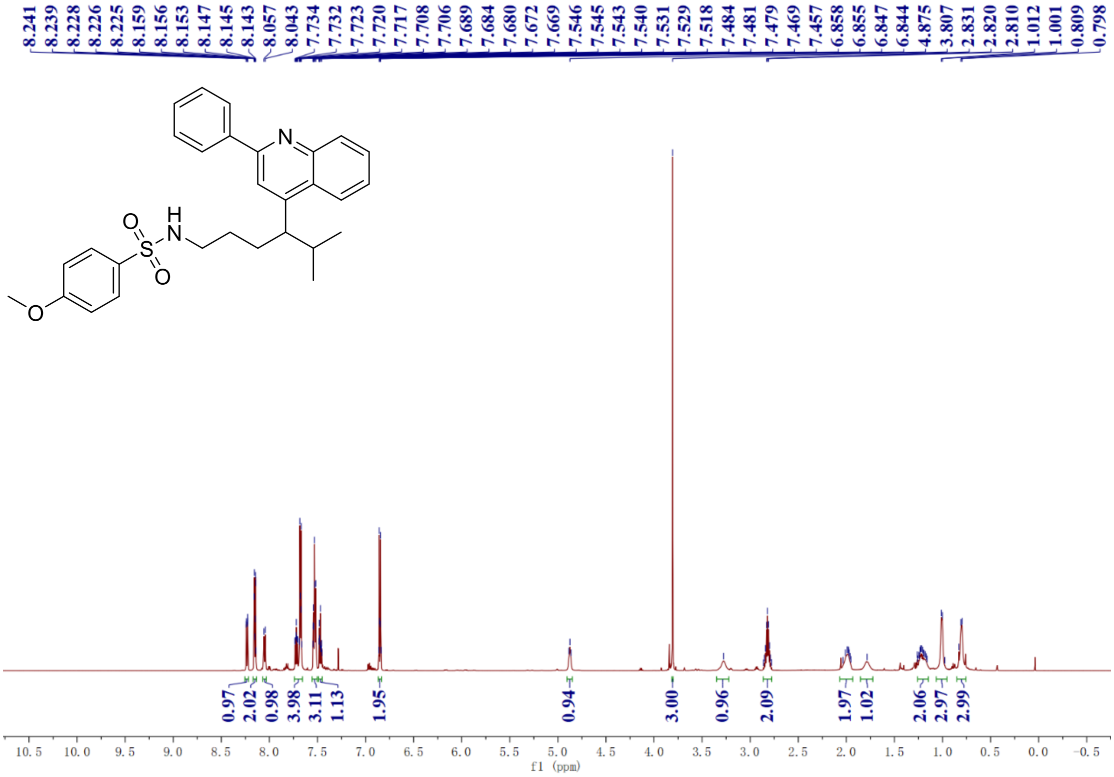

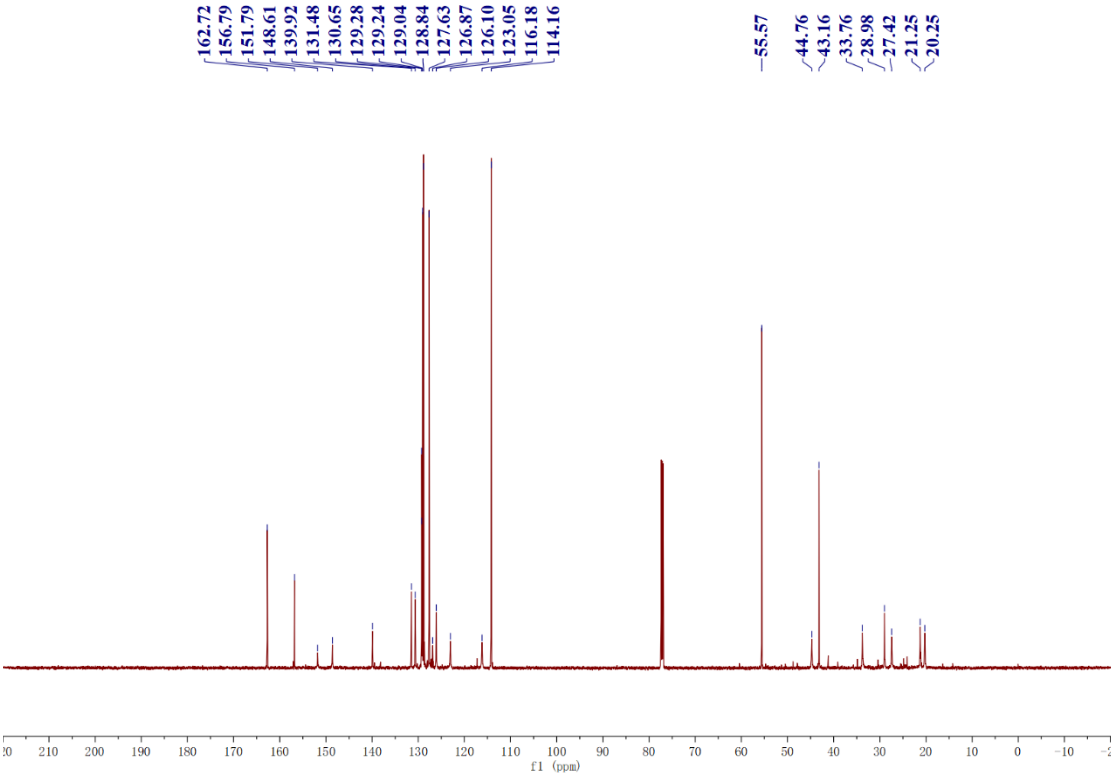

N-(4-(6-chlorobenzo[d]thiazol-2-yl)-1-phenylpentyl)-4-methoxybenzenesulfonamide (45)

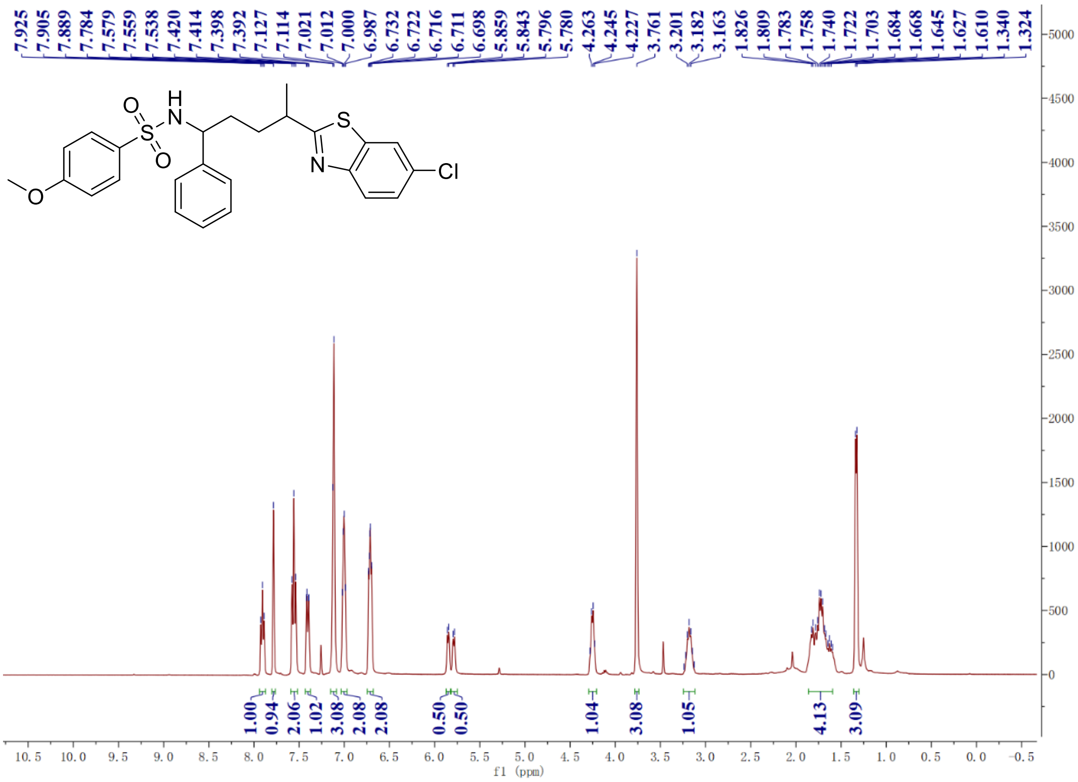

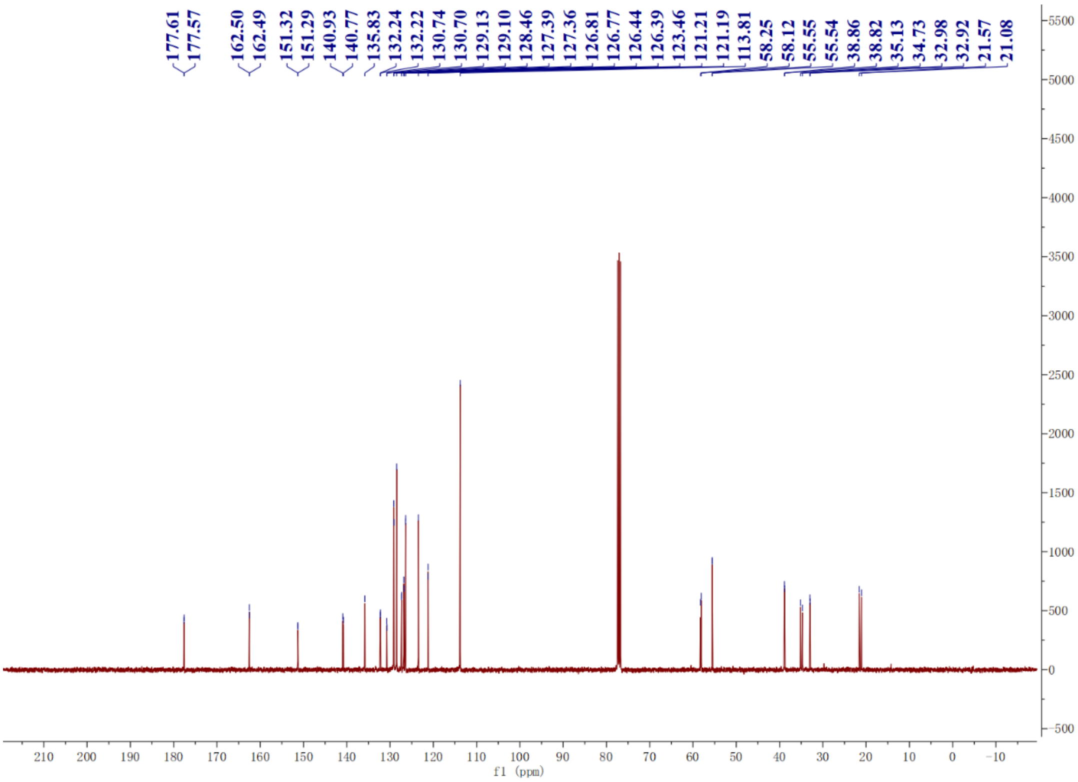

methyl (2*R*)-2-((4-methoxyphenyl)sulfonamido)-5-(2-phenylquinolin-4-yl)hexanoate (46)

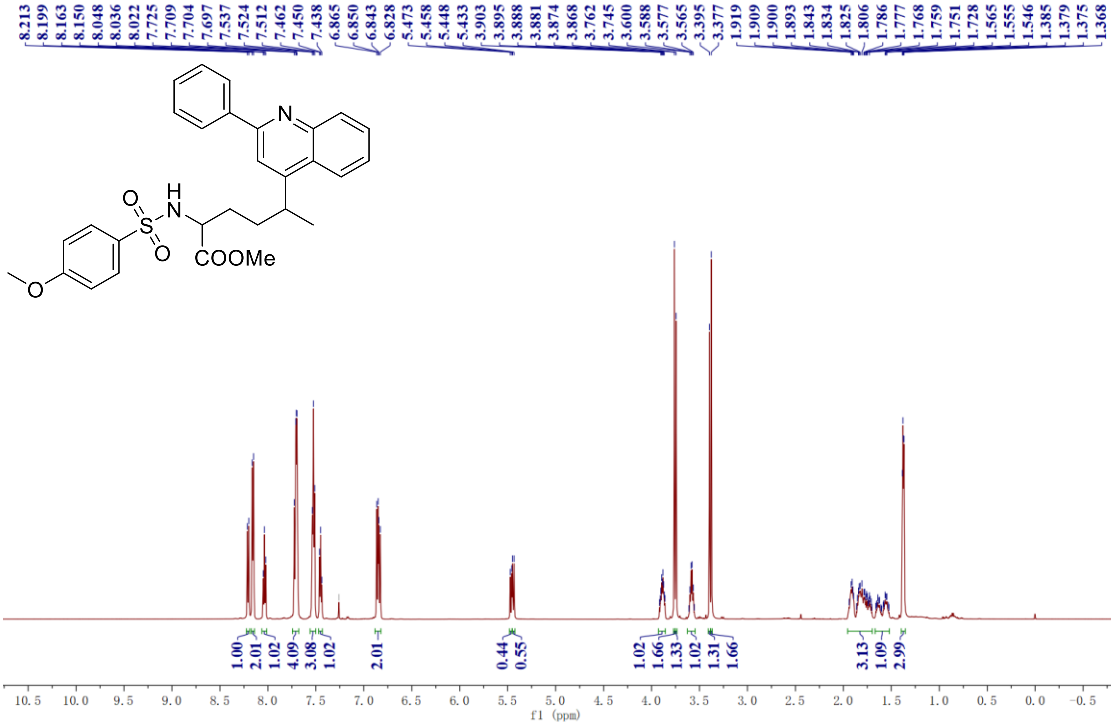

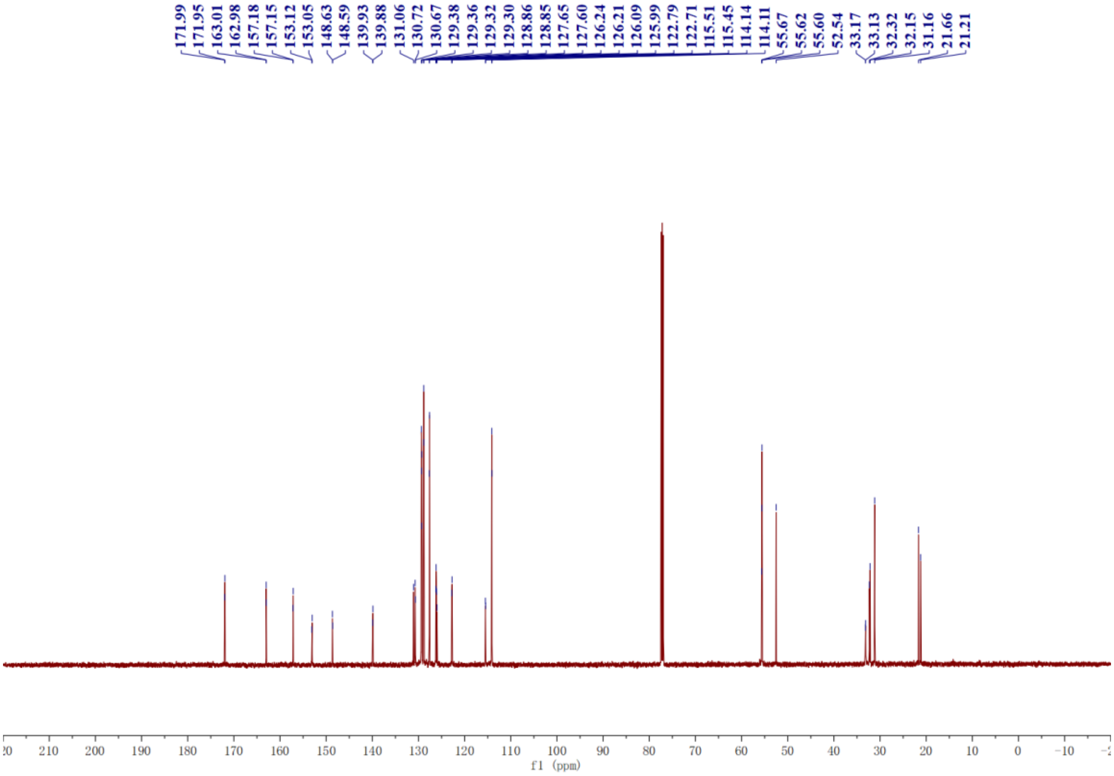

4-methoxy-*N*-(2-methyl-4-(2-phenylquinolin-4-yl)pentyl)benzenesulfonamide (47)

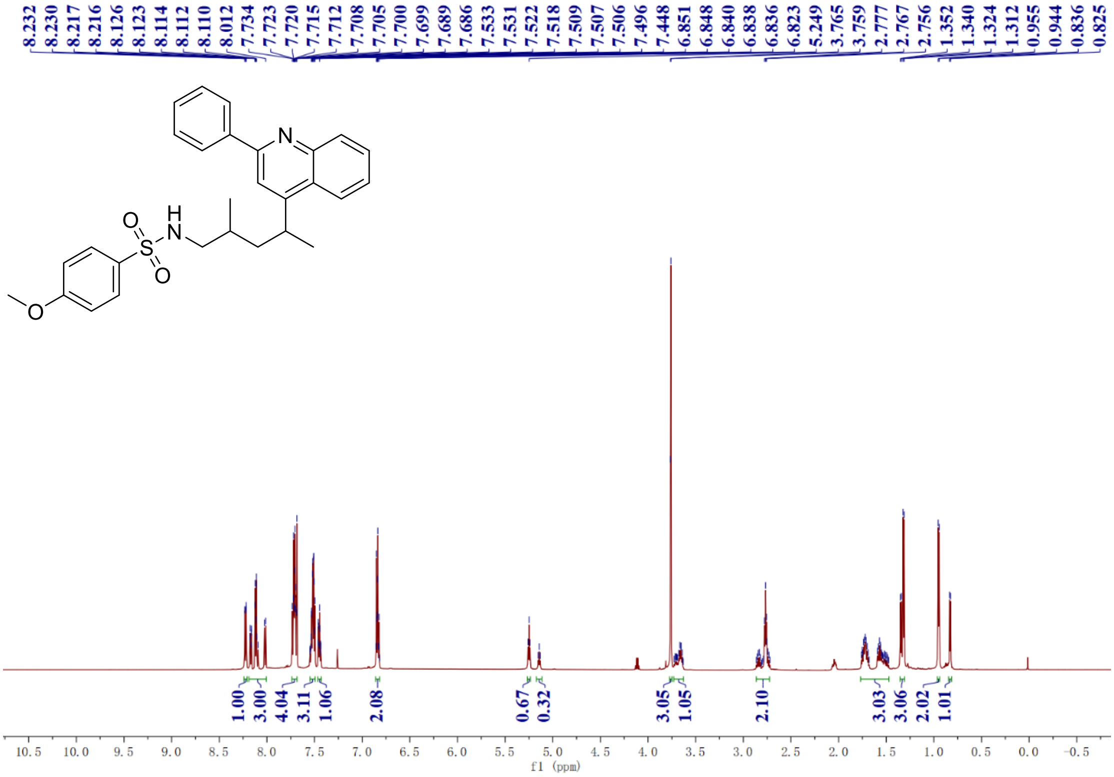

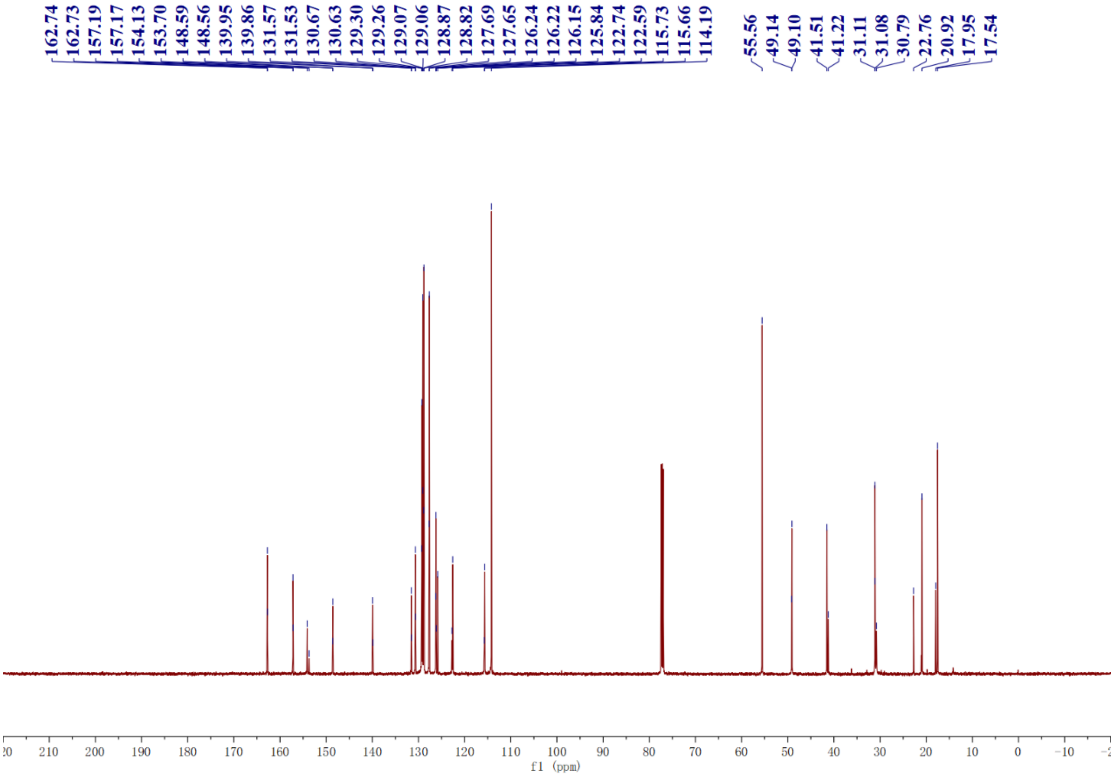

4-methoxy-*N*-(3-methyl-4-(2-phenylquinolin-4-yl)pentyl)benzenesulfonamide (48)

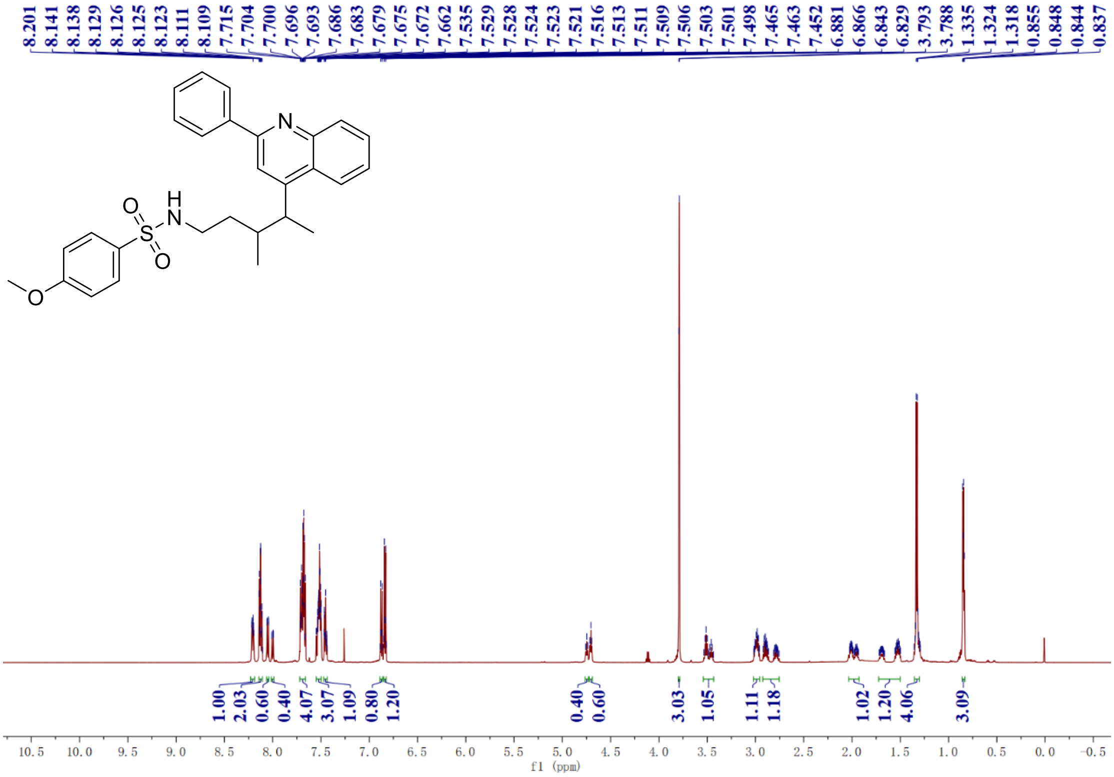

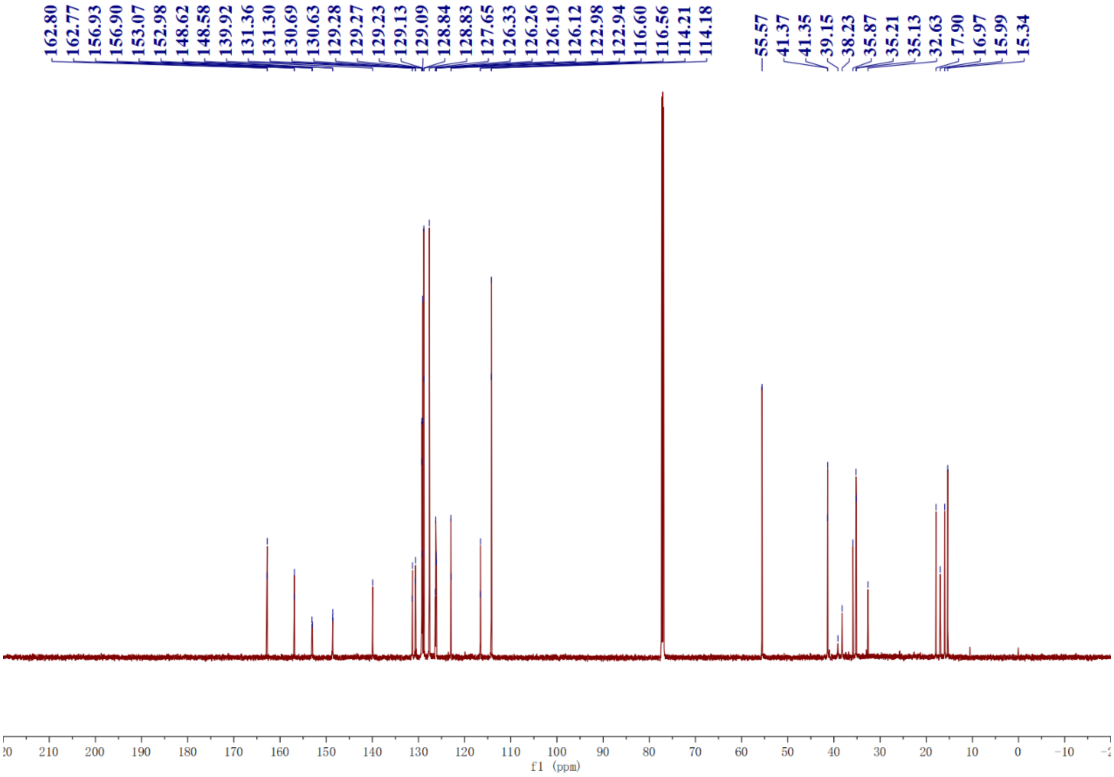

N-(2-(1-(6-chlorobenzo[d]thiazol-2-yl)ethoxy)ethyl)-4-methoxybenzenesulfonamide (49)

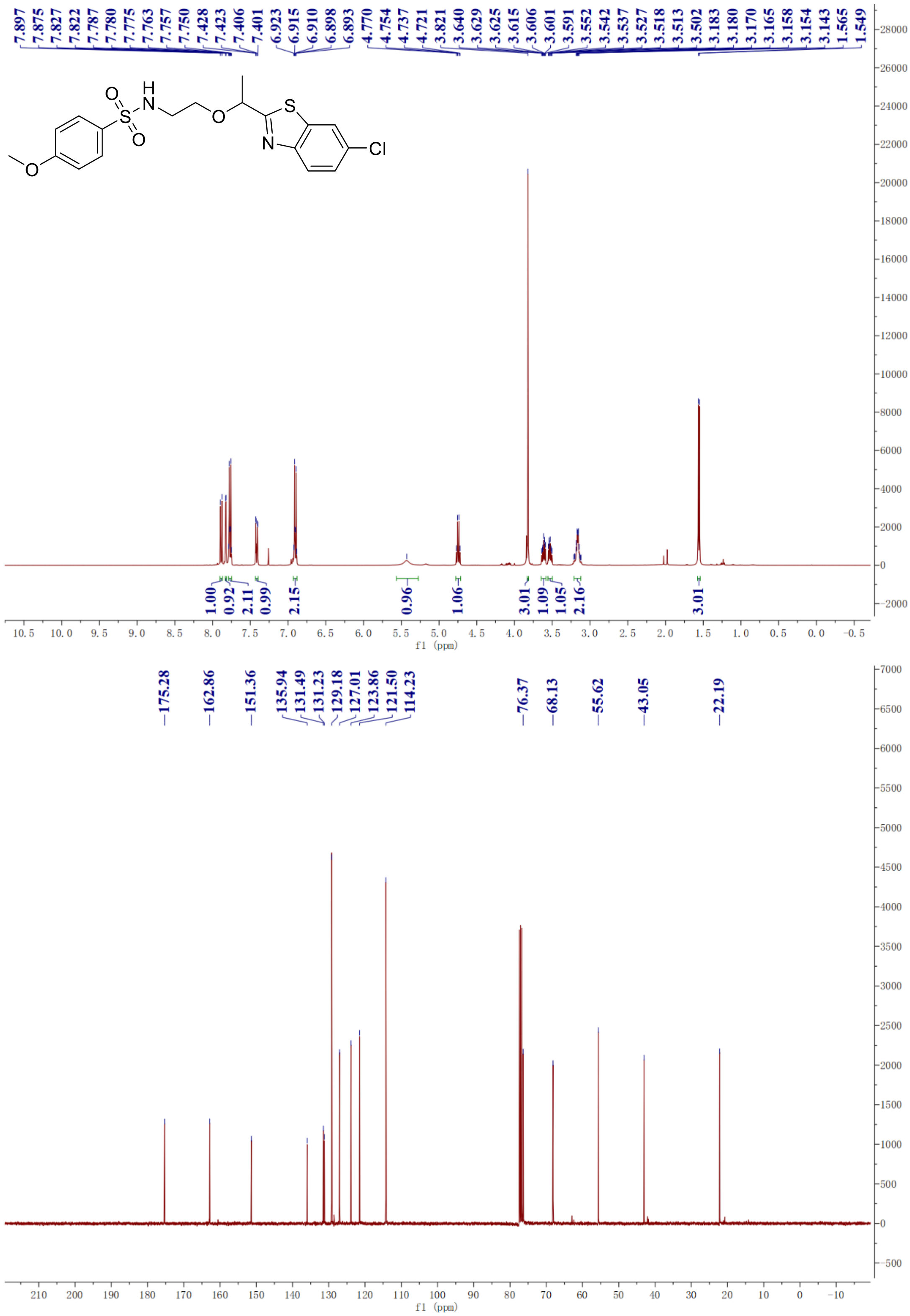

***N*-(6-chloro-4-(6-chlorobenzo[*d*]thiazol-2-yl)hexyl)-4-methoxybenzenesulfonamide (50)**

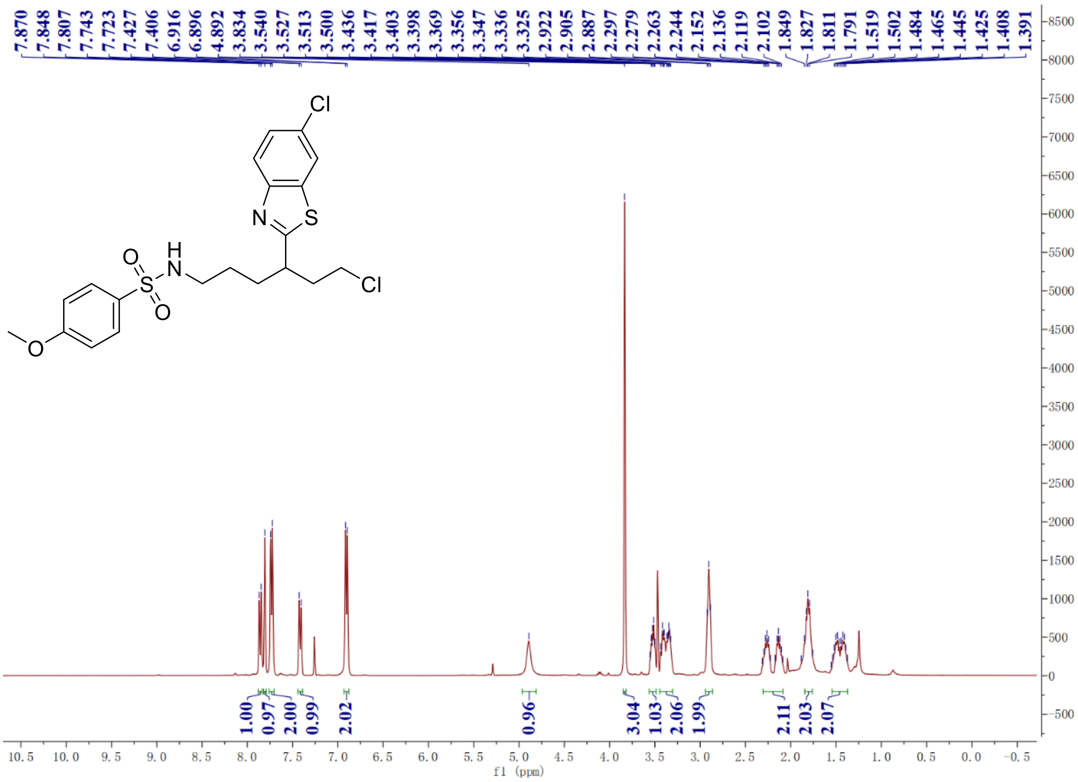

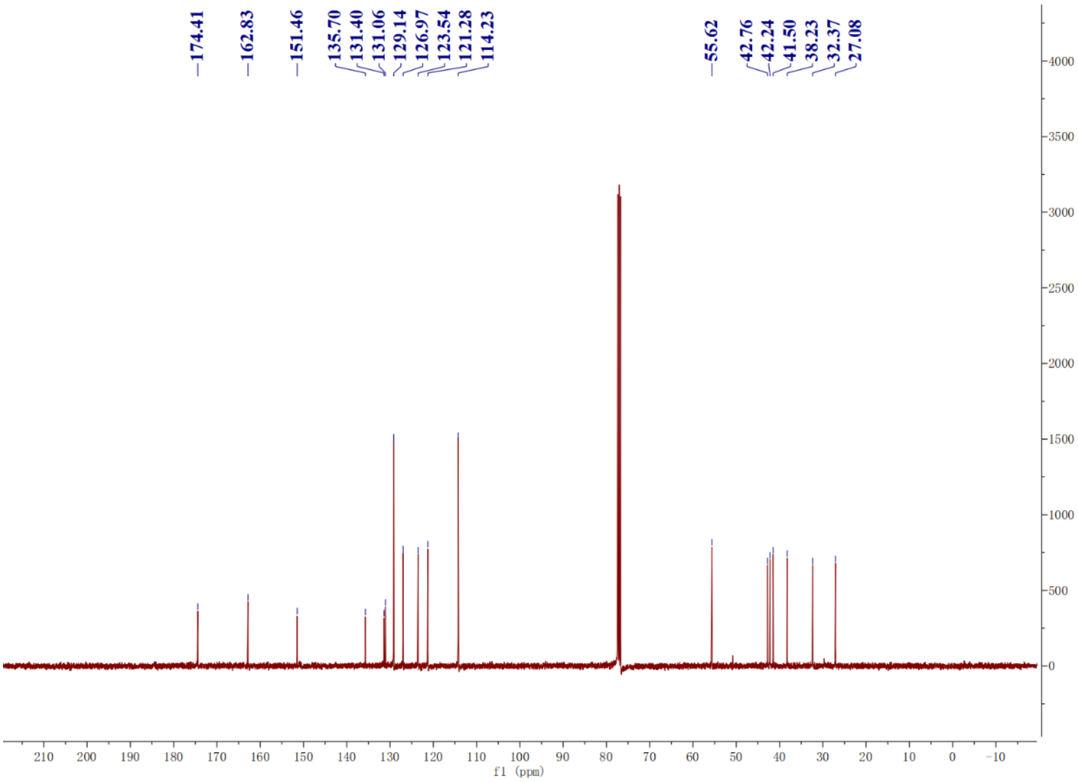

ethyl 5-(6-chlorobenzo[*d*]thiazol-2-yl)-8-((4-methoxyphenyl)sulfonamido)octanoate (51)

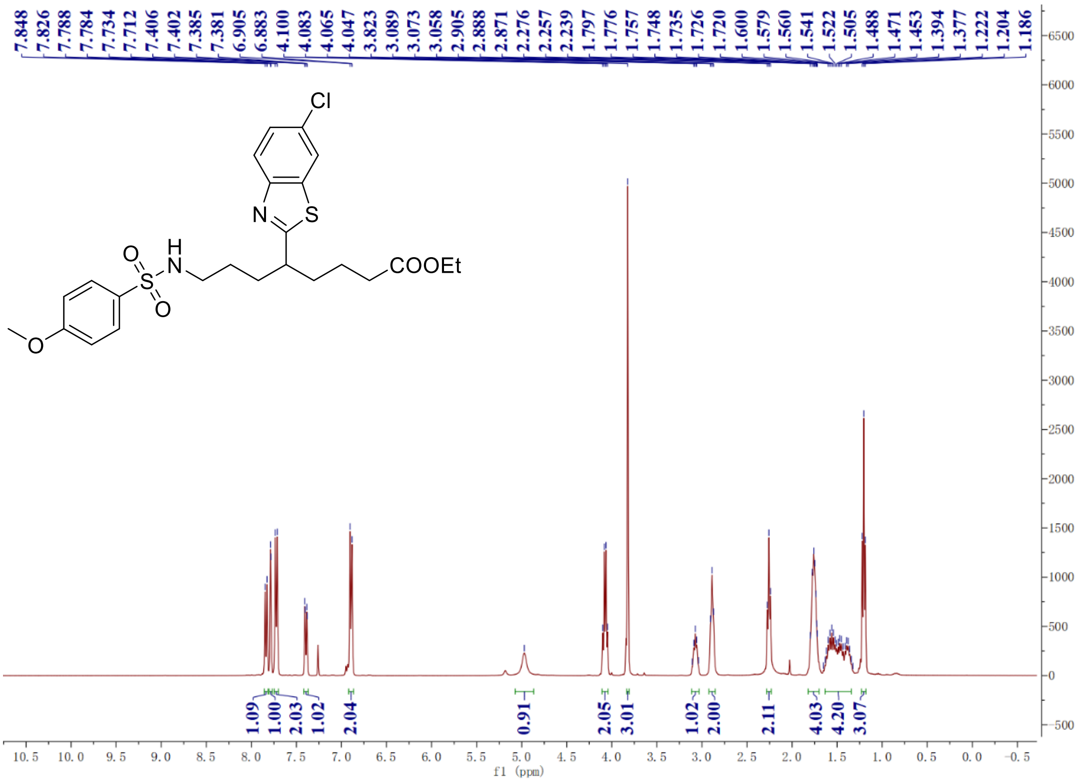

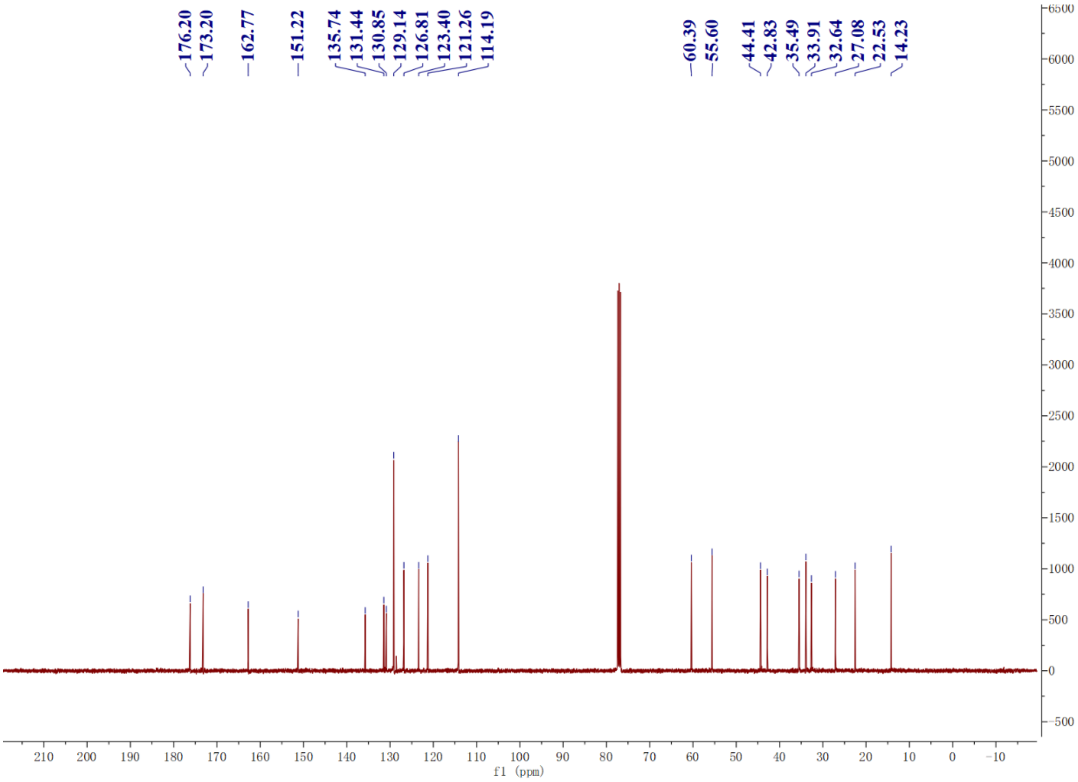

6-((4-methoxyphenyl)sulfonamido)-3-(2-phenylquinolin-4-yl)hexyl 4-methylbenzoate (52)

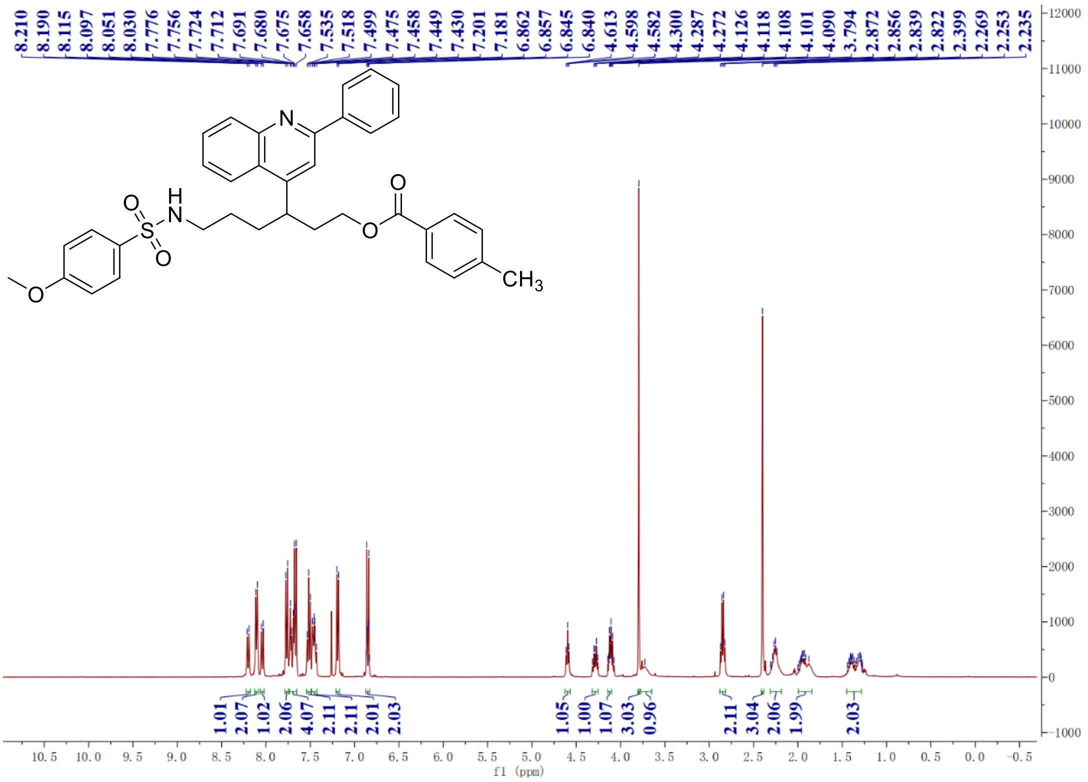

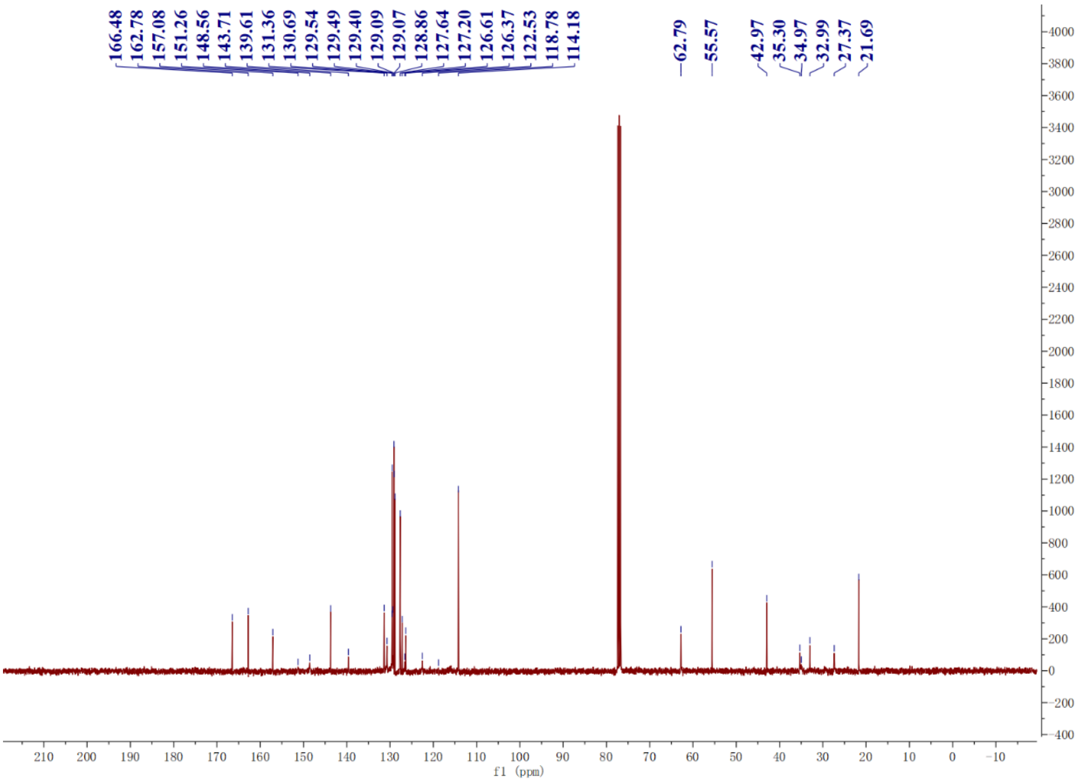

_N_-((3-(6-chlorobenzo[_d_]thiazol-2-yl)cyclohexyl)methyl)-4-methoxybenzenesulfonamide (53)

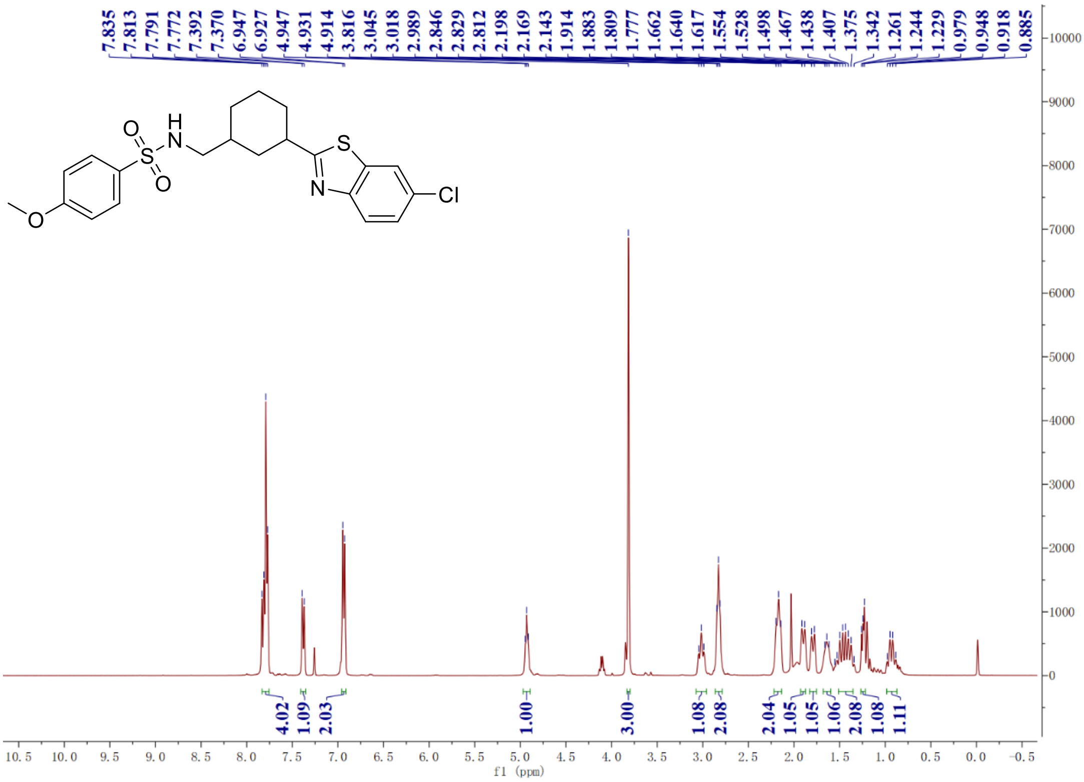

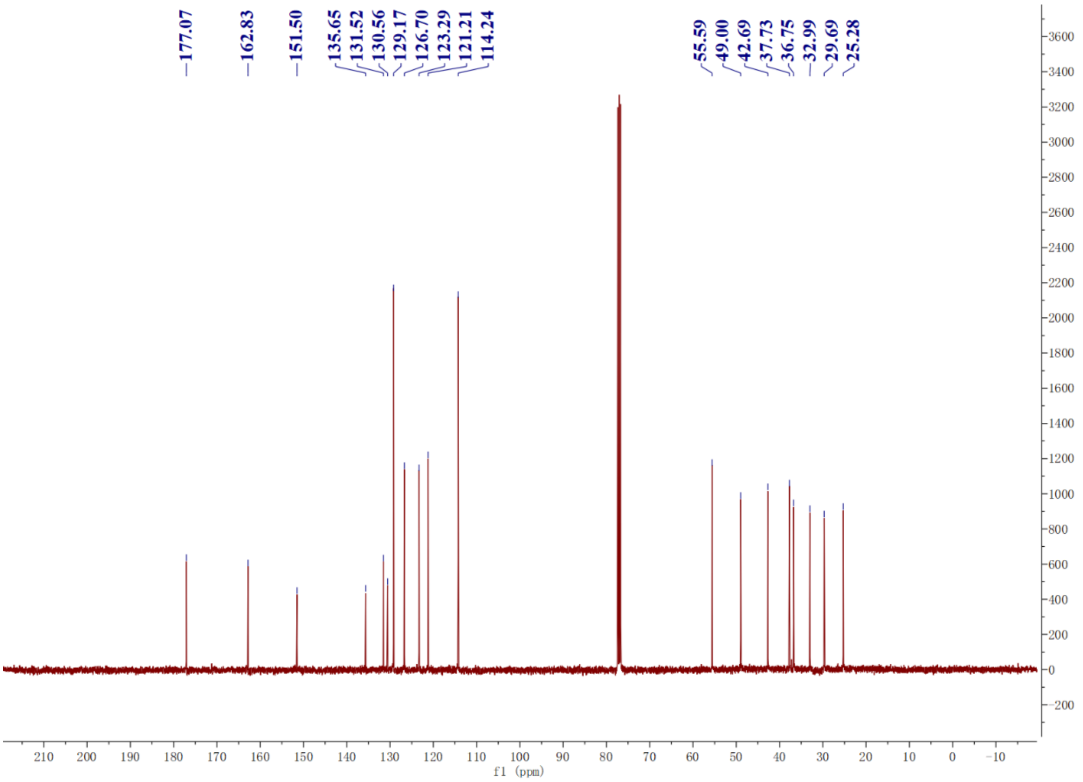

_N_-(2-(2-(6-chlorobenzo[_d_]thiazol-2-yl)cyclopentyl)ethyl)-4-methoxybenzenesulfonamide (54)

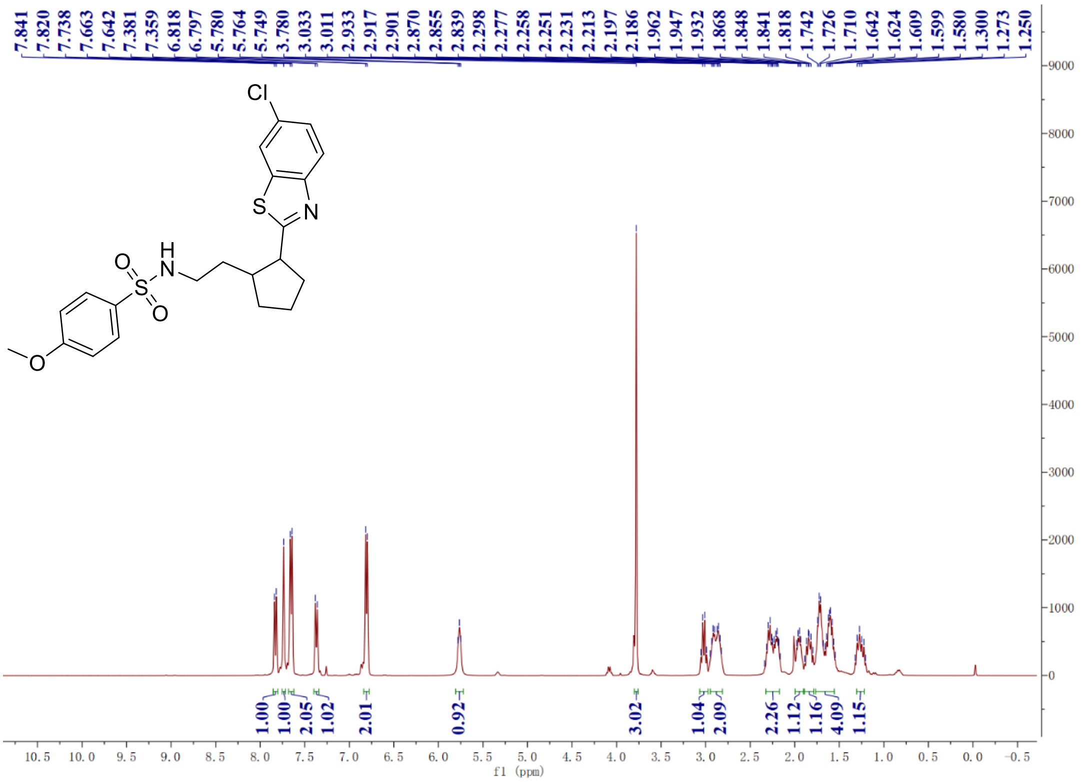

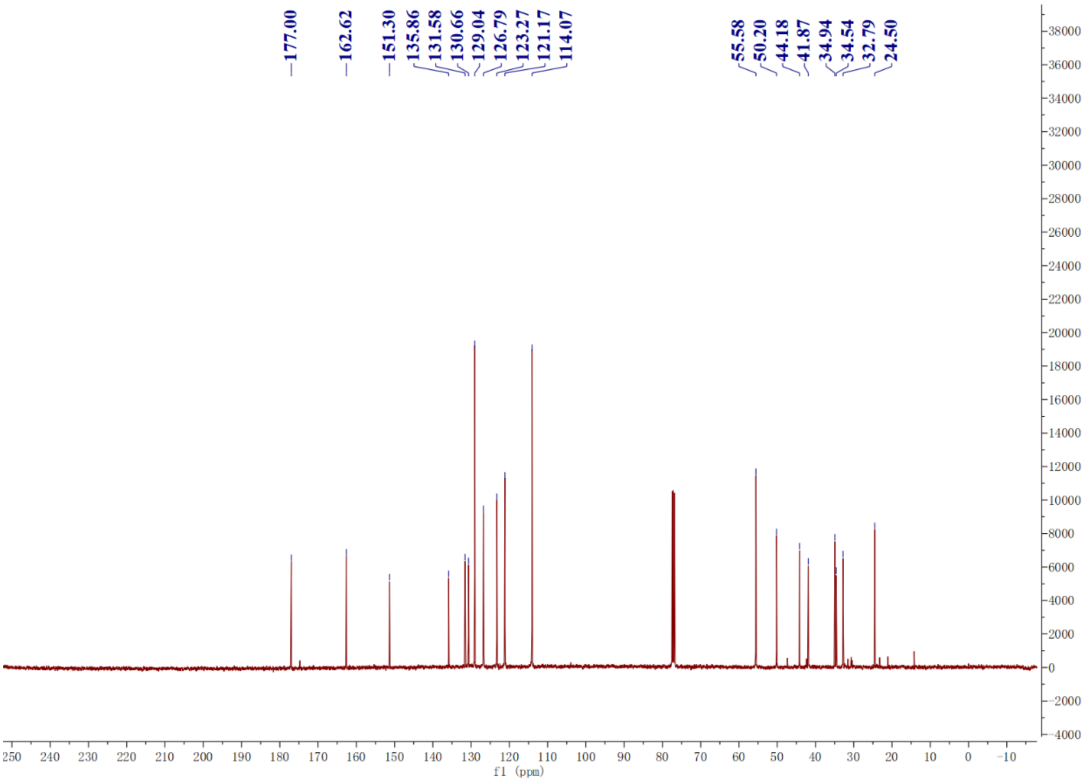

_N_-(2-(2-(6-chlorobenzo[_d_]thiazol-2-yl)cyclohexyl)ethyl)-4-methoxybenzenesulfonamide (55)

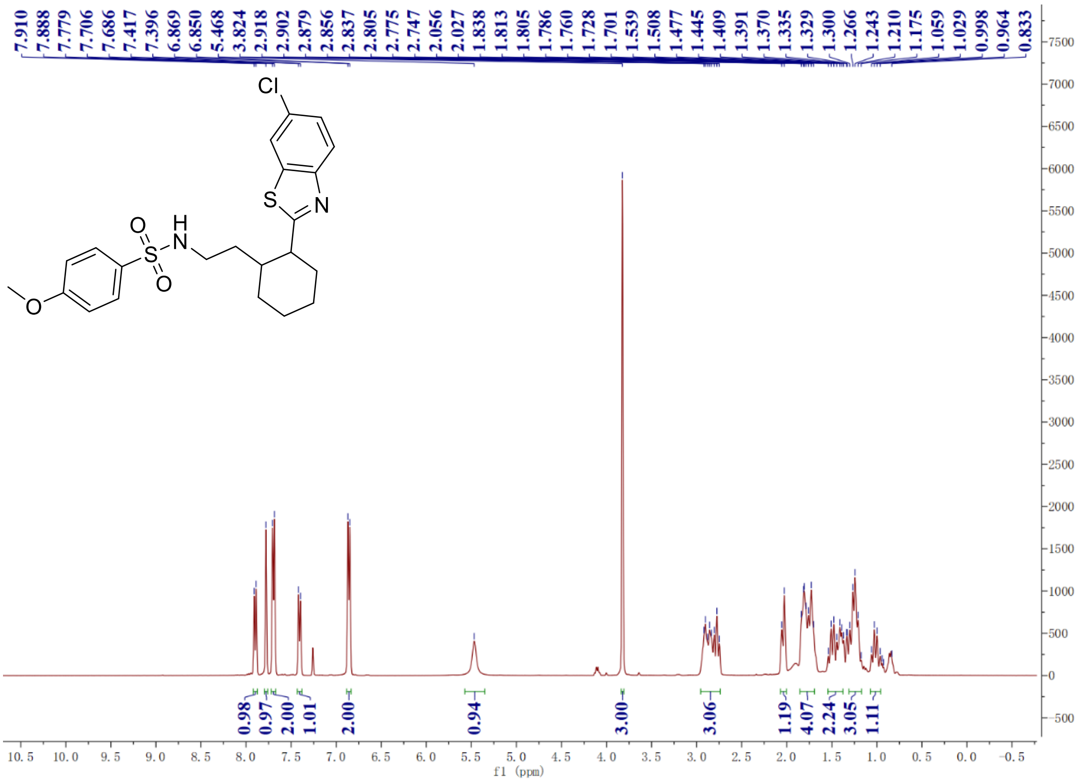

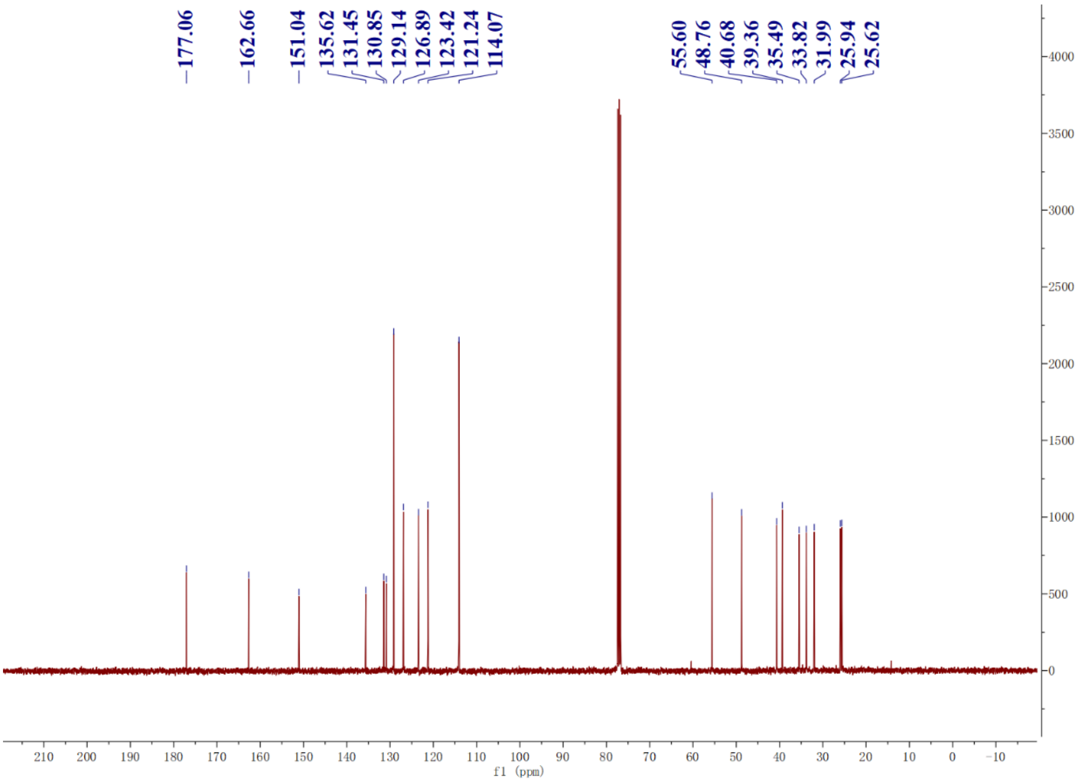

N-(3-(1-(6-chlorobenzo[d]thiazol-2-yl)cyclopentyl)propyl)-4-methoxybenzenesulfonamide (56)

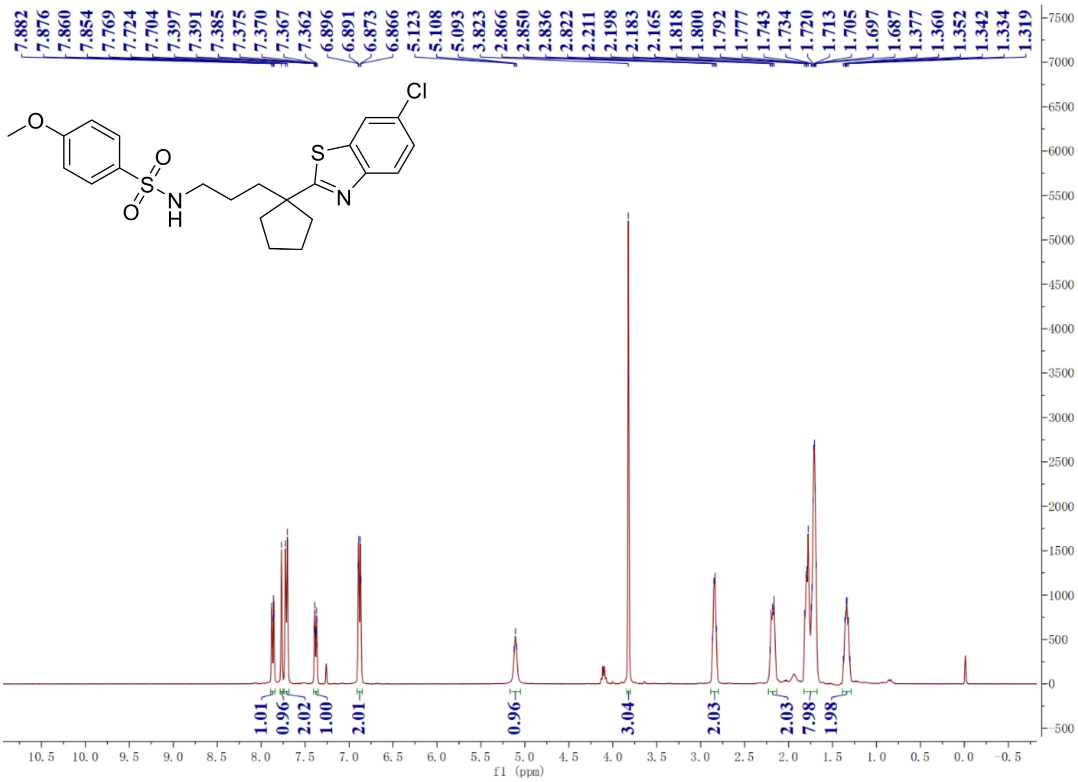

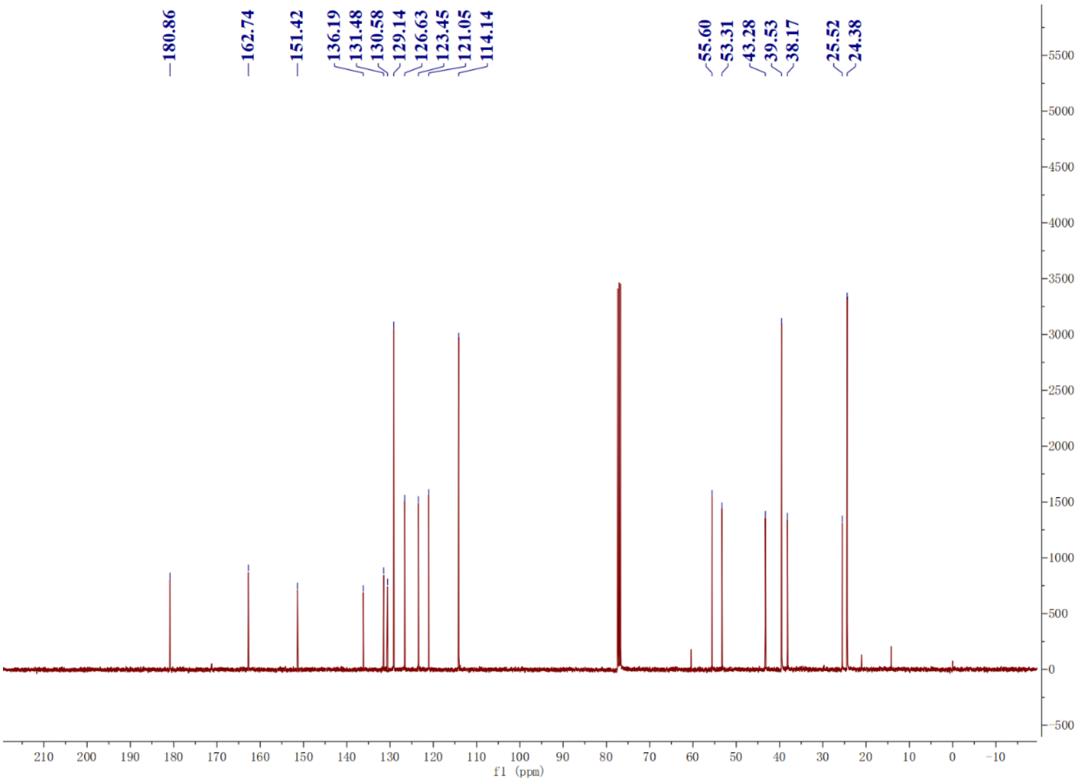

***N*-(3-(1-(6-chlorobenzo[*d*]thiazol-2-yl)cyclohexyl)propyl)-4-methoxybenzenesulfonamide (57)**

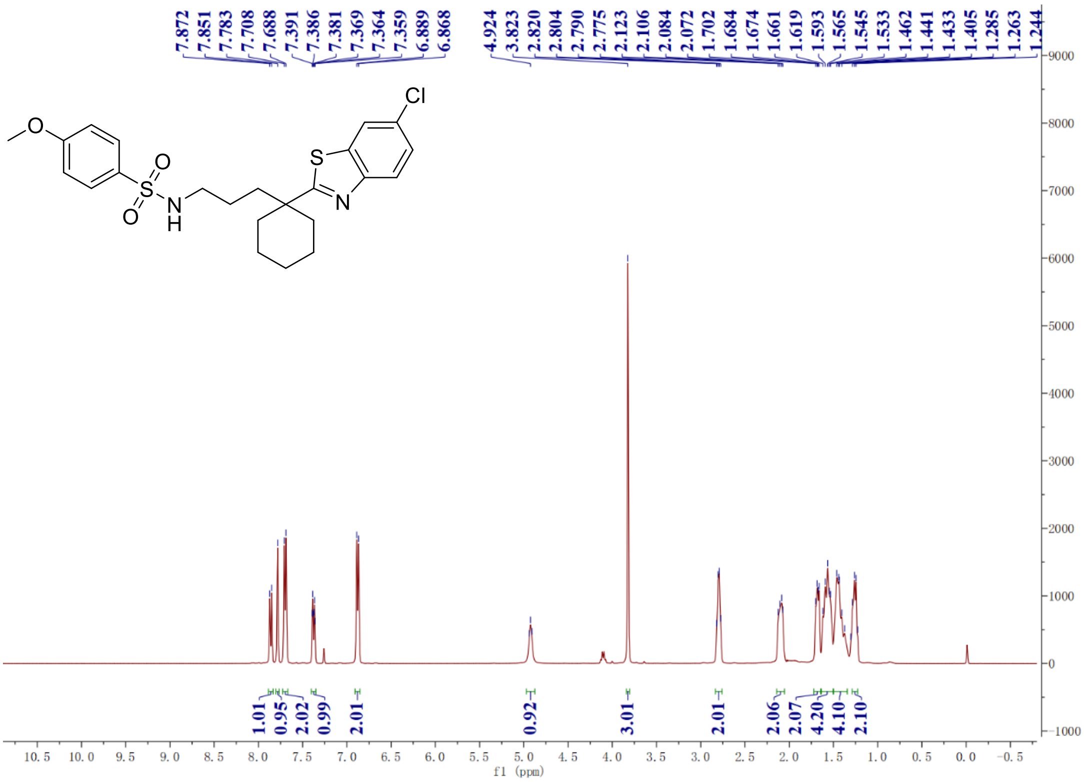

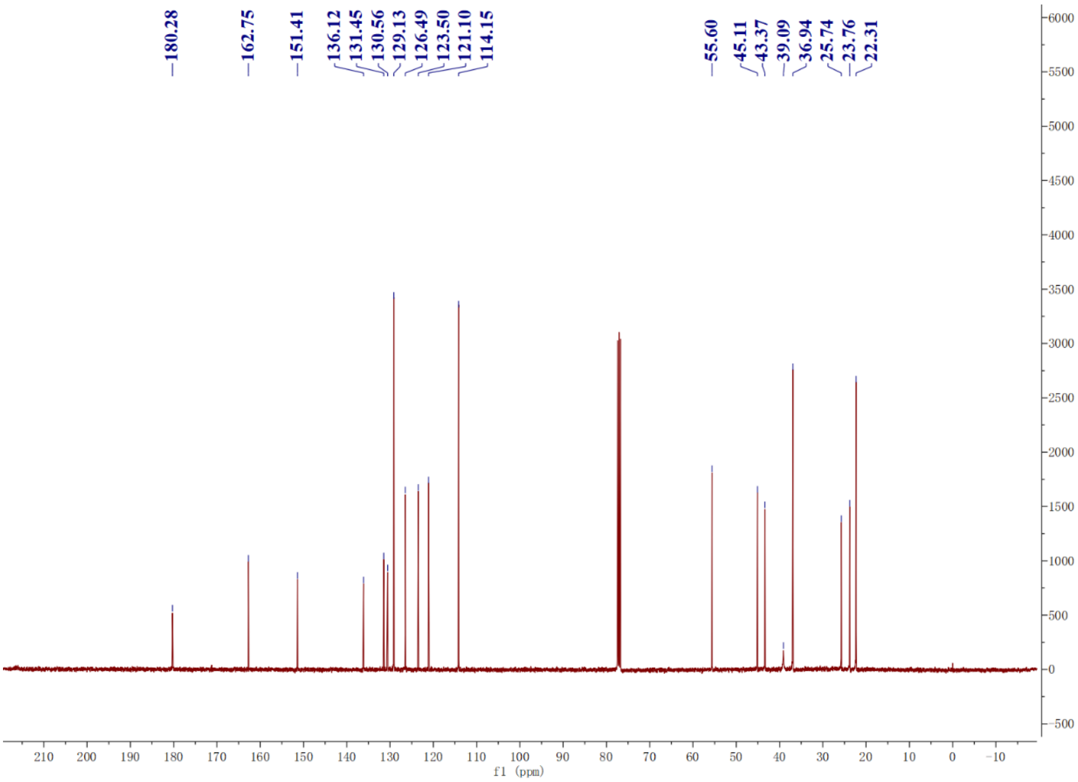

***N*-(4-(6-chlorobenzo[*d*]thiazol-2-yl)-4-methylpentyl)-4-methoxybenzenesulfonamide (58)**

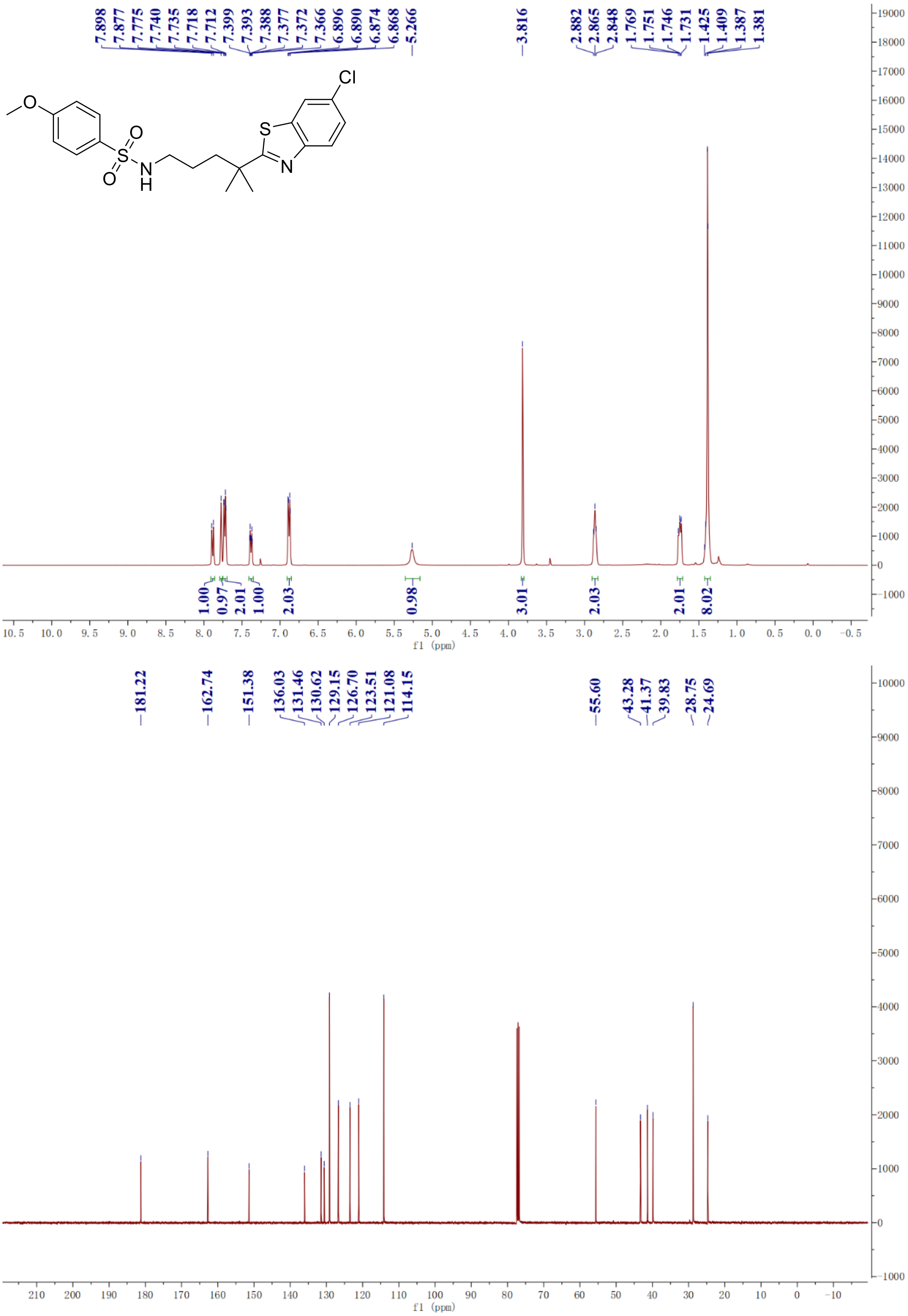

N-(4-(6-chlorobenzo[d]thiazol-2-yl)butyl)-4-methoxybenzenesulfonamide (59)

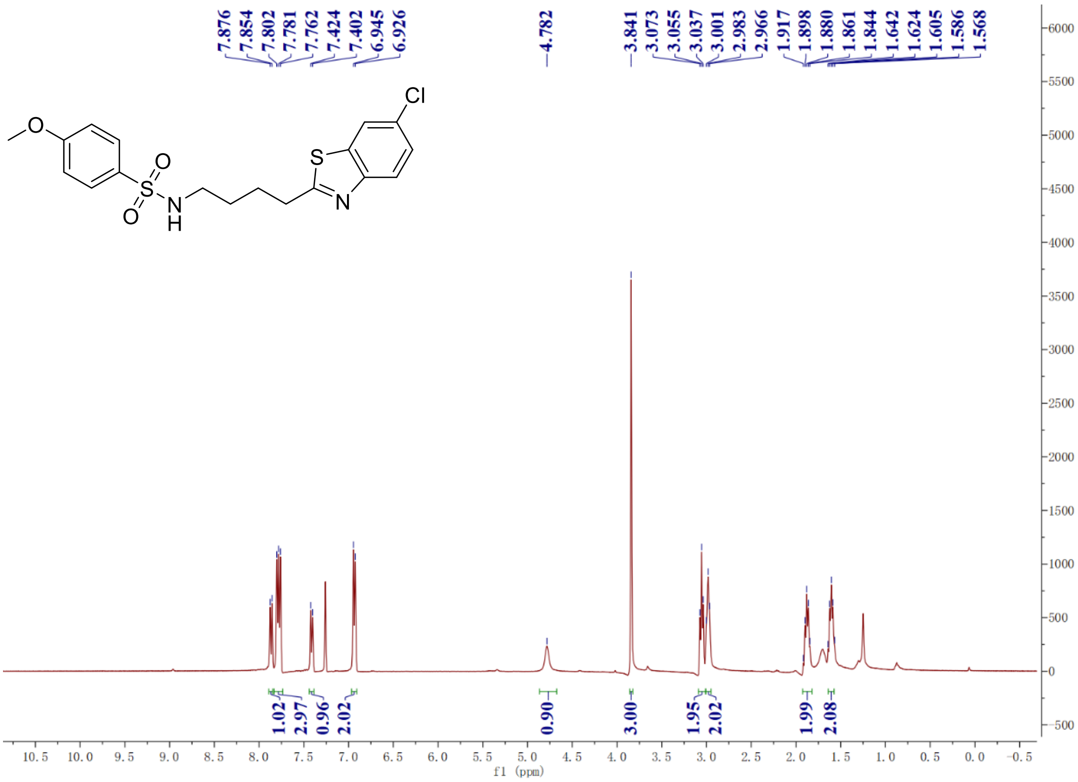

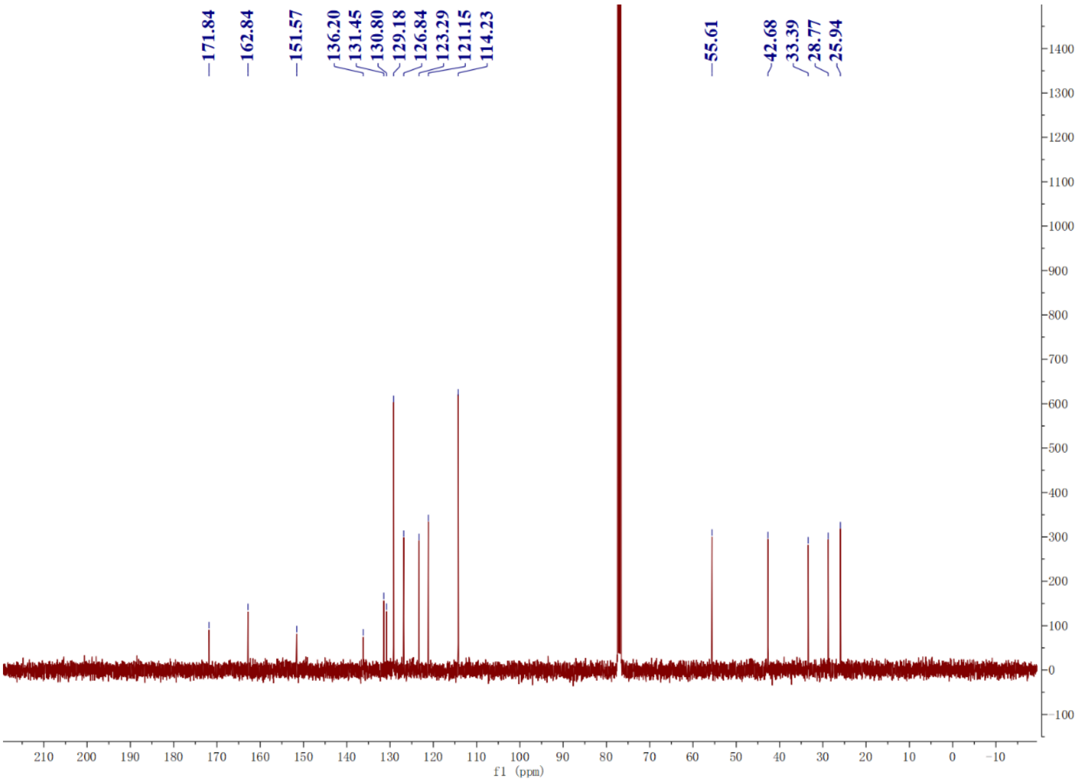

4-methoxy-*N*-(3-methyl-4-(2-phenylquinolin-4-yl)butyl)benzenesulfonamide (60)

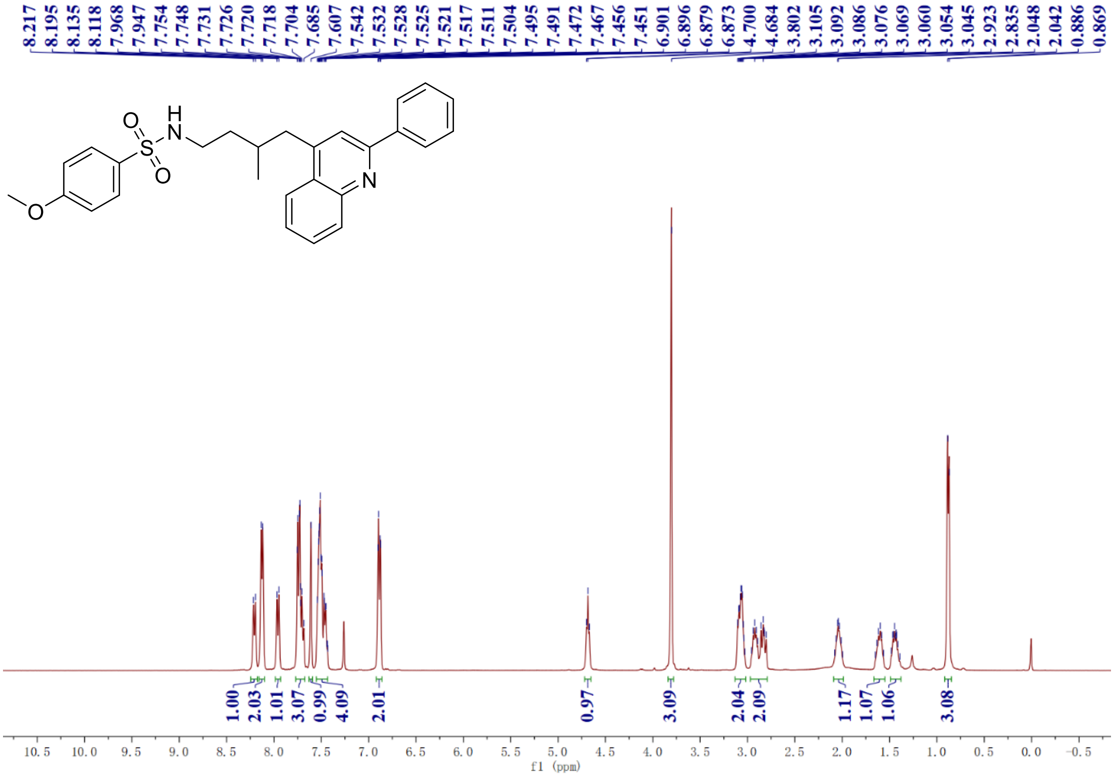

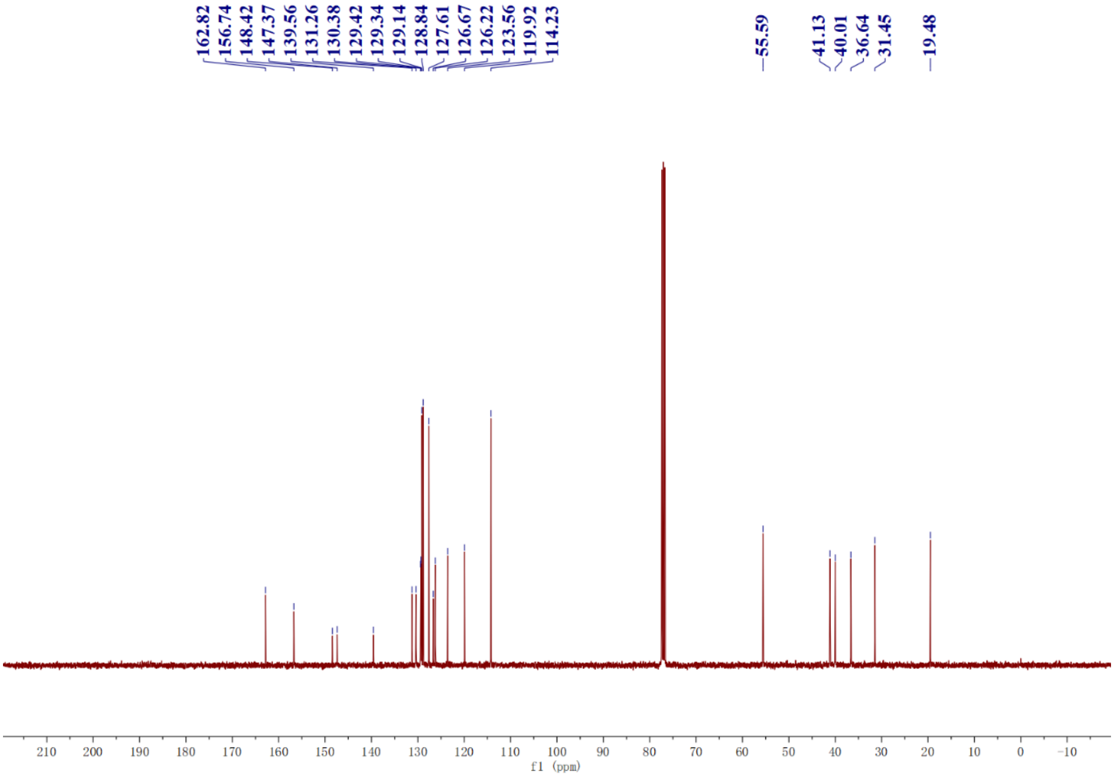

3-(cyclopropylmethoxy)-*N*-(3,5-dichloro-2-(5-((4-methoxyphenyl)sulfonamido)pentan-2-yl)pyridin-4-yl)-4-(difluoromethoxy)benzamide (61)

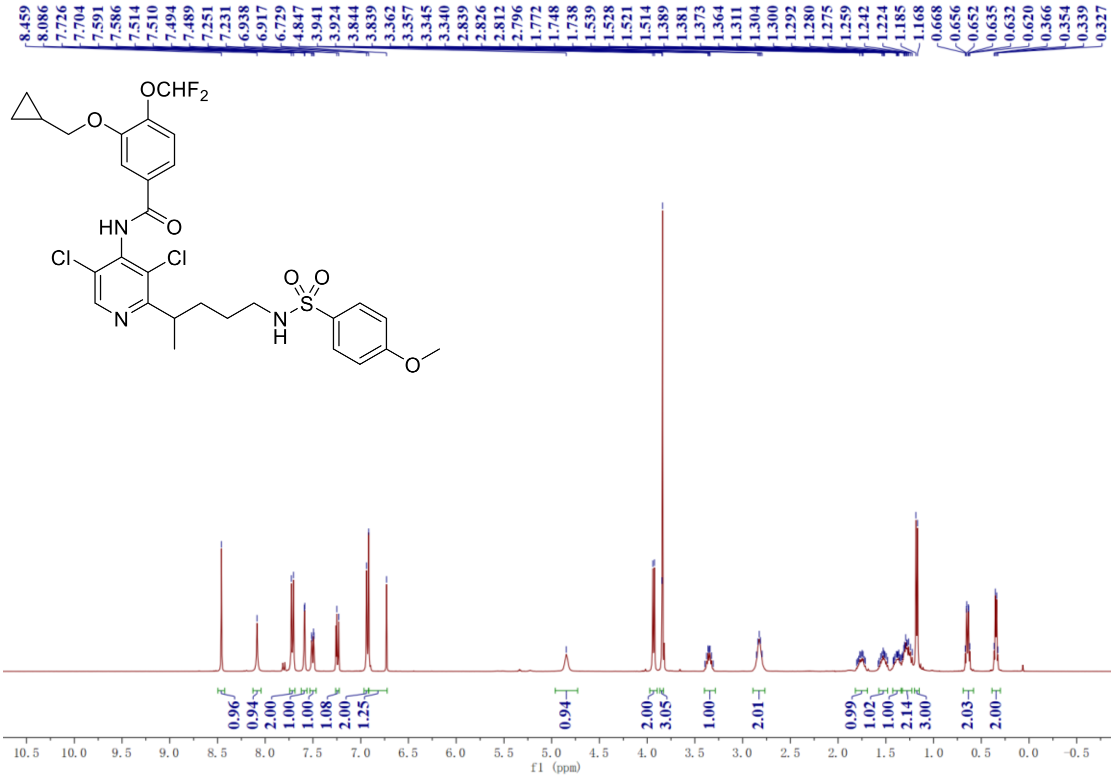

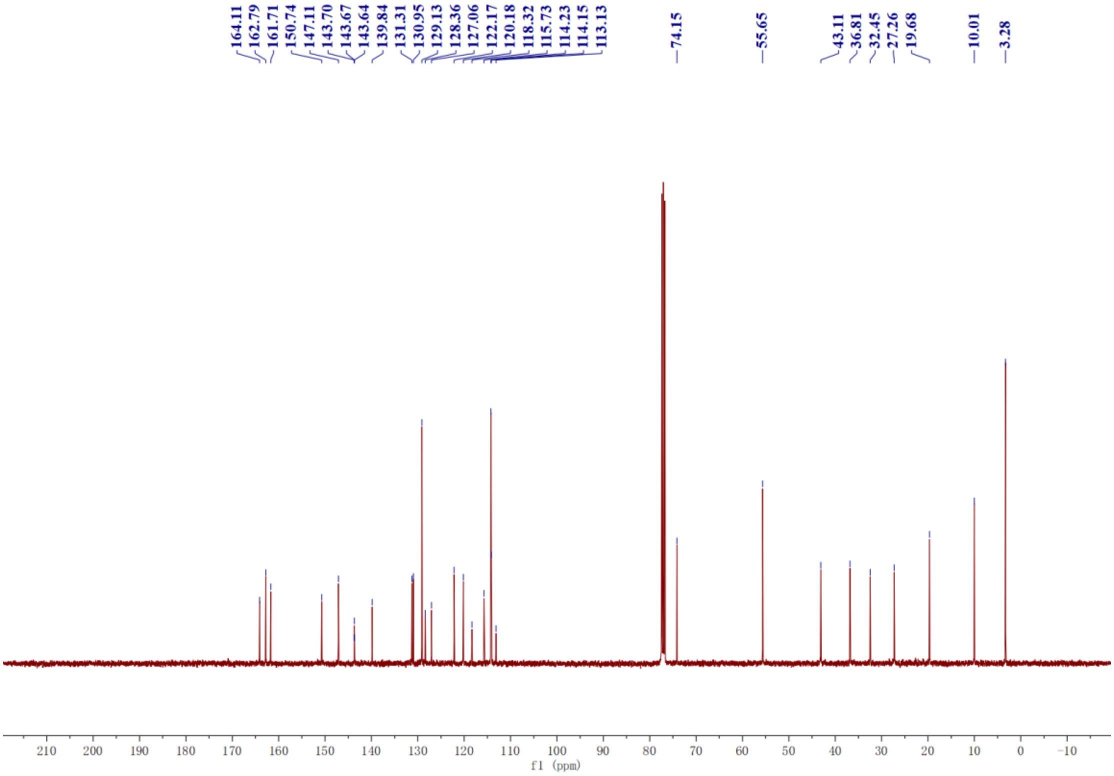

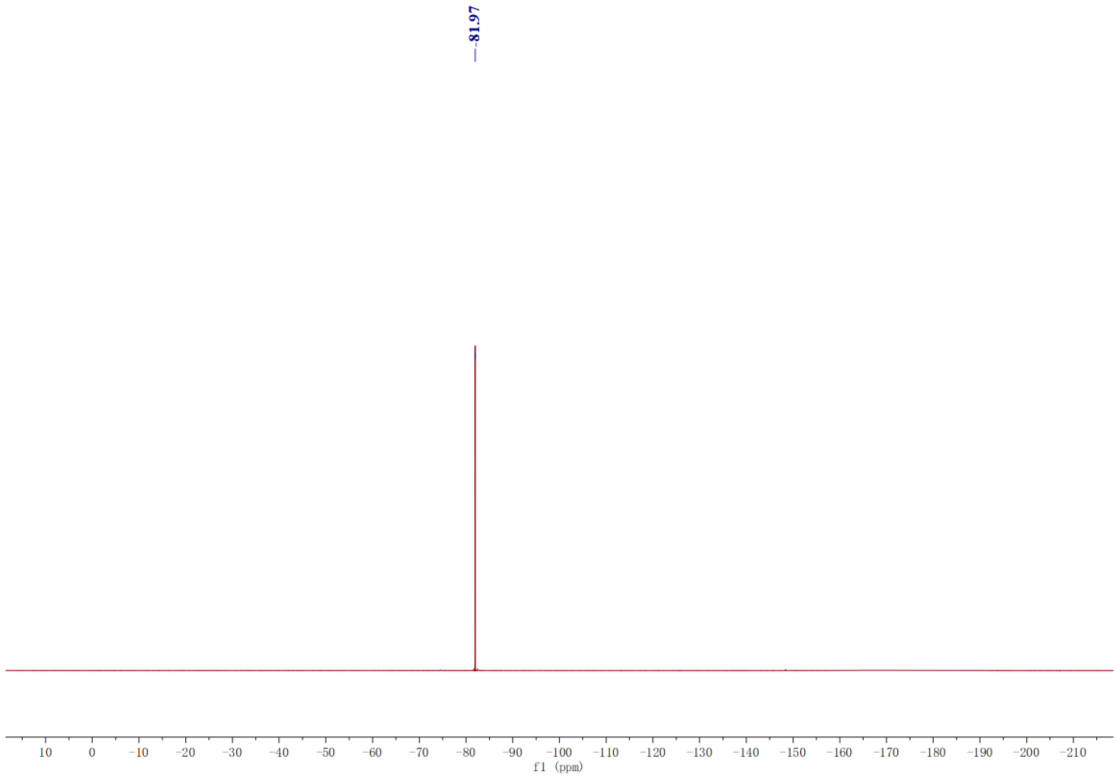
-81.97
10 0 -10 -20 -30 -40 -50 -60 -70 -80 -90 -100 -110 -120 -130 -140 -150 -160 -170 -180 -190 -200 -210
f1 (ppm)

N-(4-(4-((2*S*,3*R*)-3-(2,4-difluorophenyl)-3-hydroxy-4-(1*H*-1,2,4-triazol-1-yl)butan-2-yl)-5-fluoropyrimidin-2-yl)pentyl)-4-methoxybenzenesulfonamide (62)

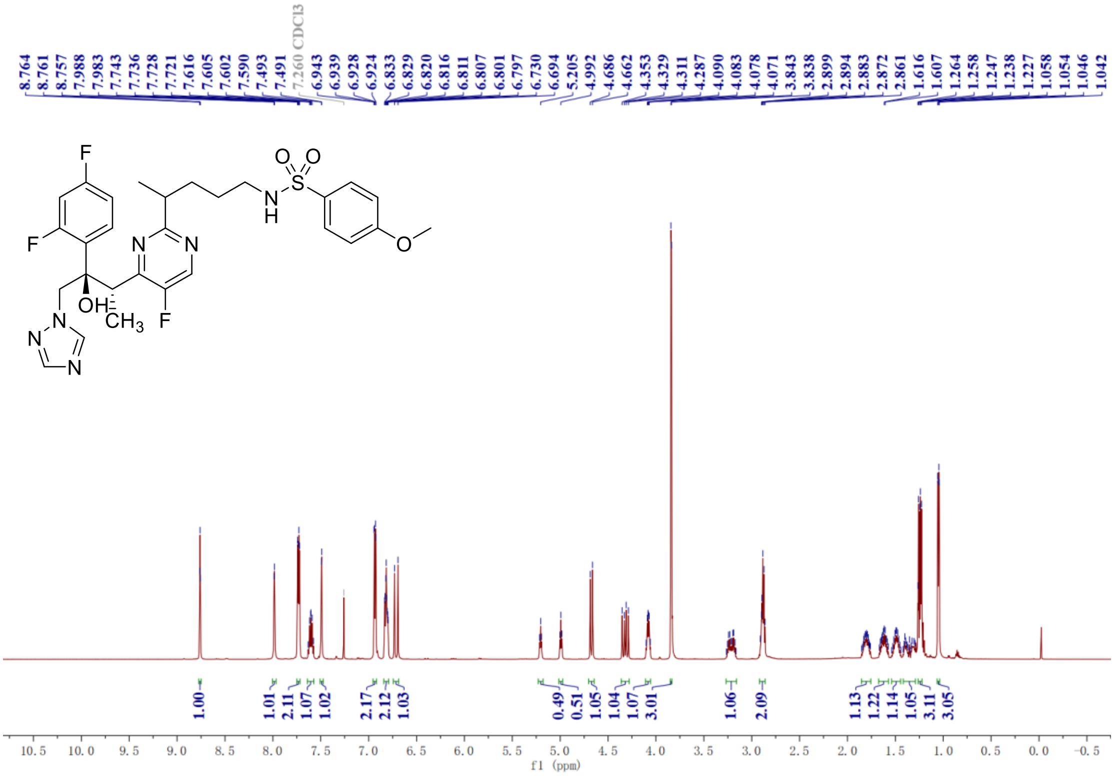

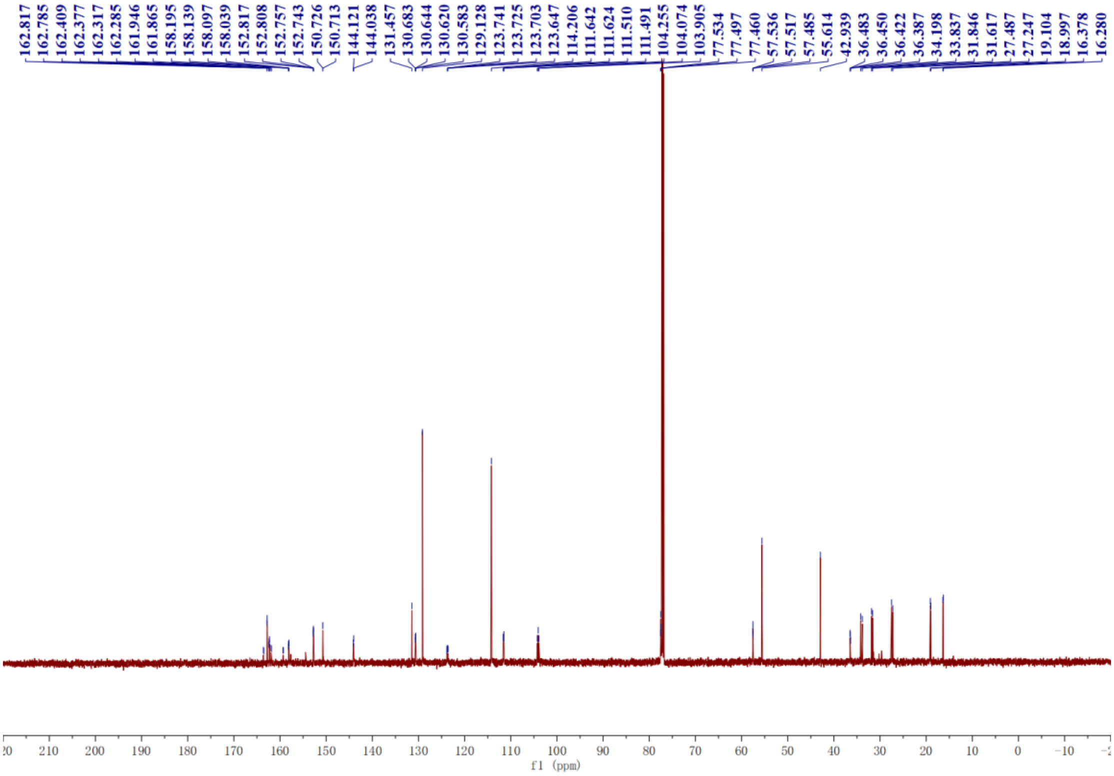

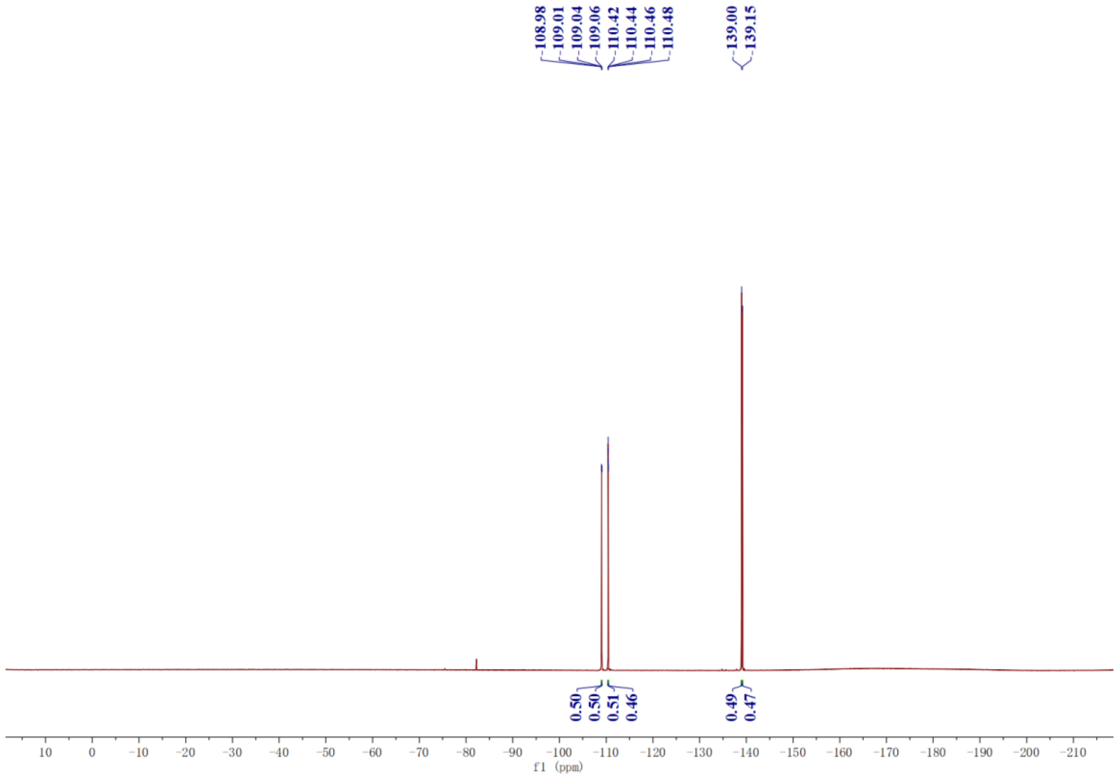
-108.98
-109.01
-109.04
-109.06
-110.42
-110.44
-110.46
-110.48
-139.00
-139.15
0.50
0.50
0.51
0.46
0.49
0.47
10 0 −10 −20 −30 −40 −50 −60 −70 −80 −90 −100 −110 −120 −130 −140 −150 −160 −170 −180 −190 −200 −210
f1 (ppm)

2-((2-(4-chlorophenoxy)-2-methylpropanoyl)oxy)ethyl 6-(5-((4-methoxyphenyl)sulfonamido)pentan-2-yl)nicotinate (63)

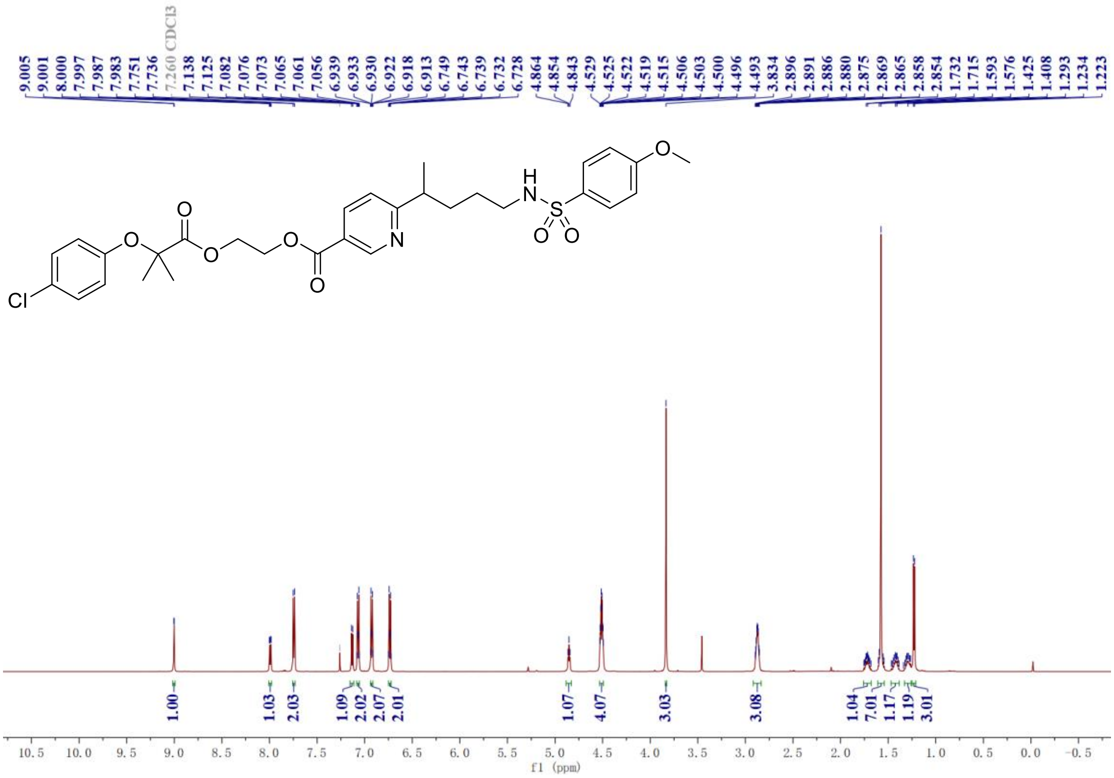

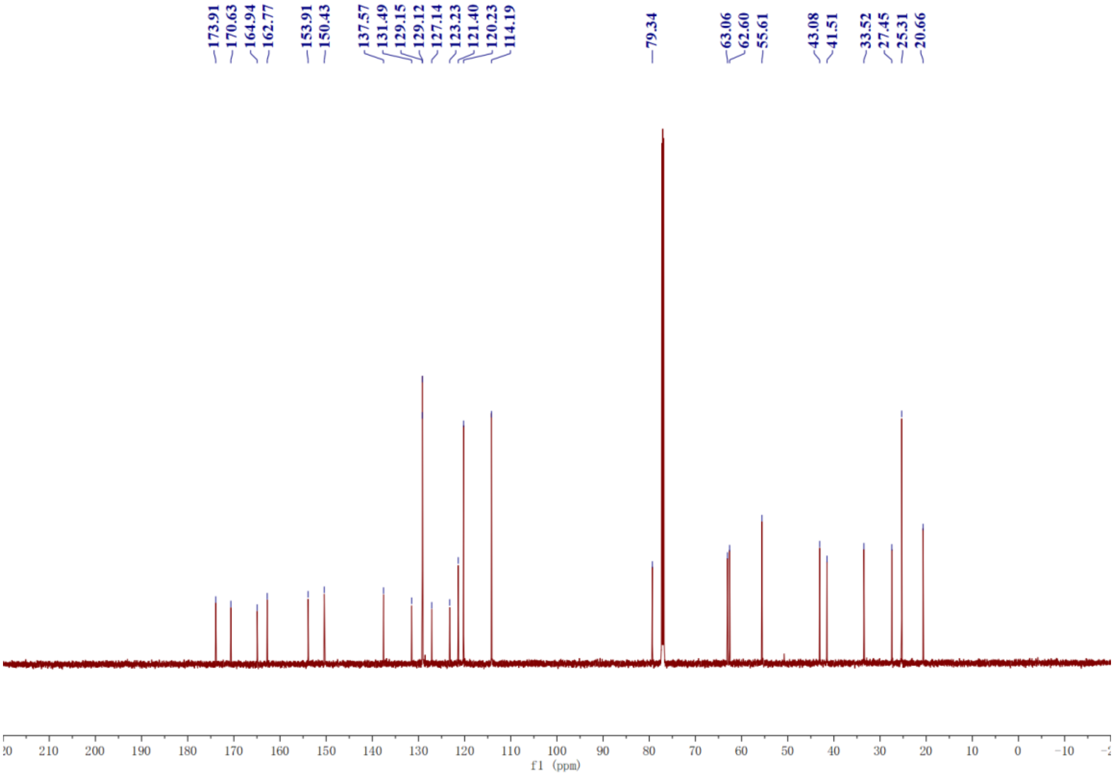

(2*R*)-1,7,7-trimethylbicyclo[2.2.1]heptan-2-yl 7-((4-methoxyphenyl)sulfonamido)-4-(2-phenylquinolin-4-yl)heptanoate (64)

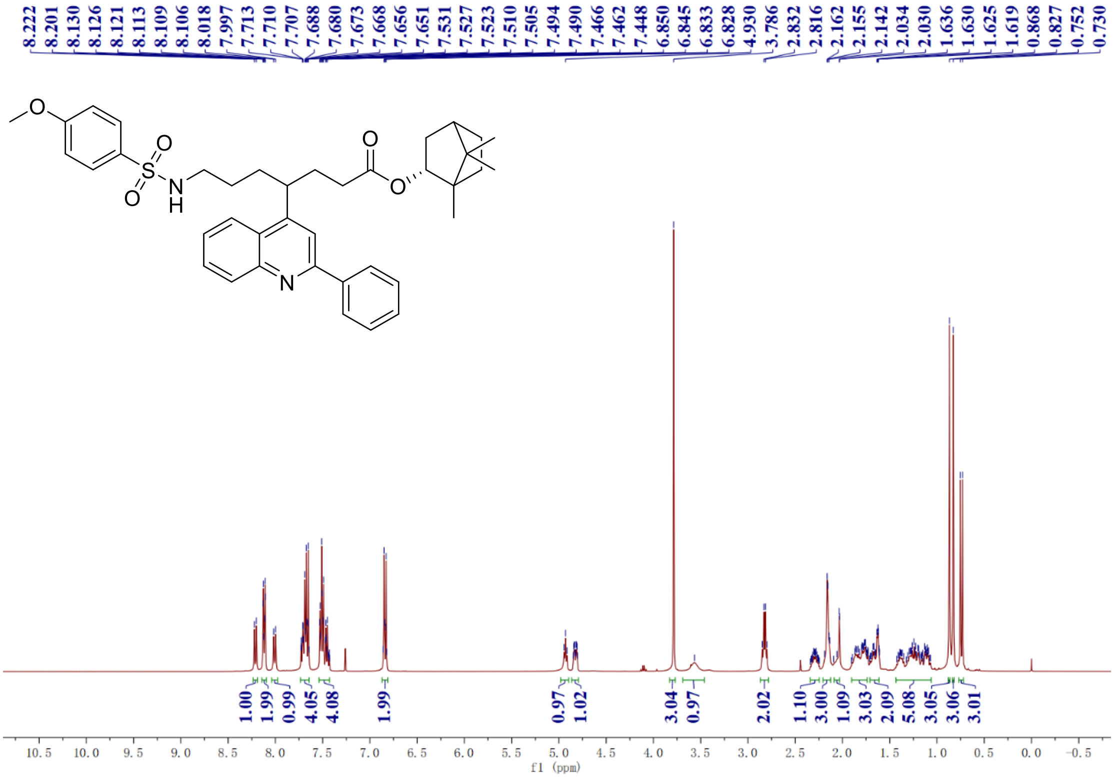

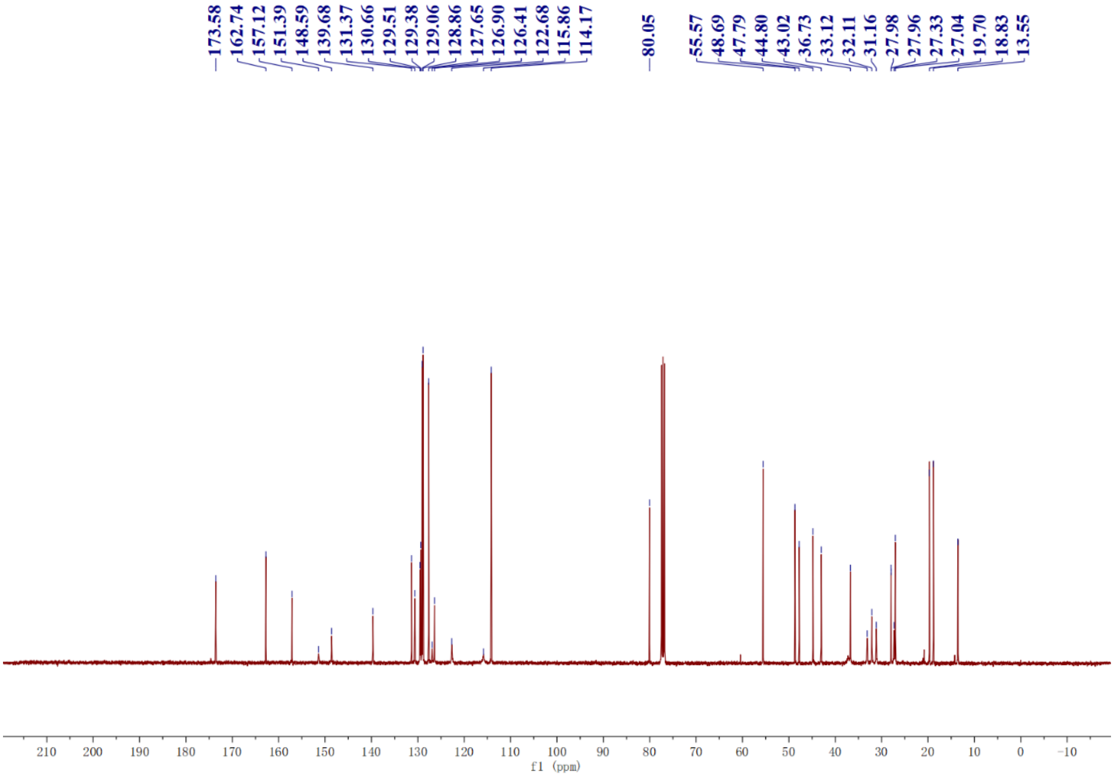

(2*S*,5*R*)-2-isopropyl-5-methylcyclohexyl 4-(6-chlorobenzo[*d*]thiazol-2-yl)-7-((4-methoxyphenyl)sulfonamido)heptanoate (65)

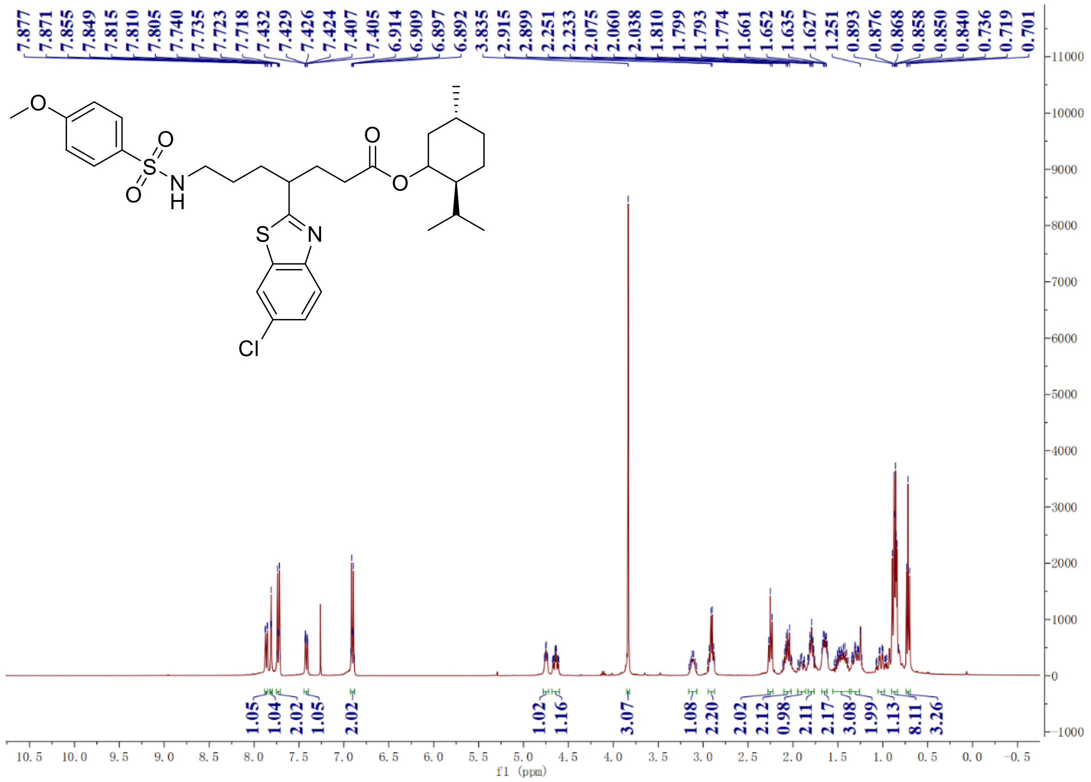

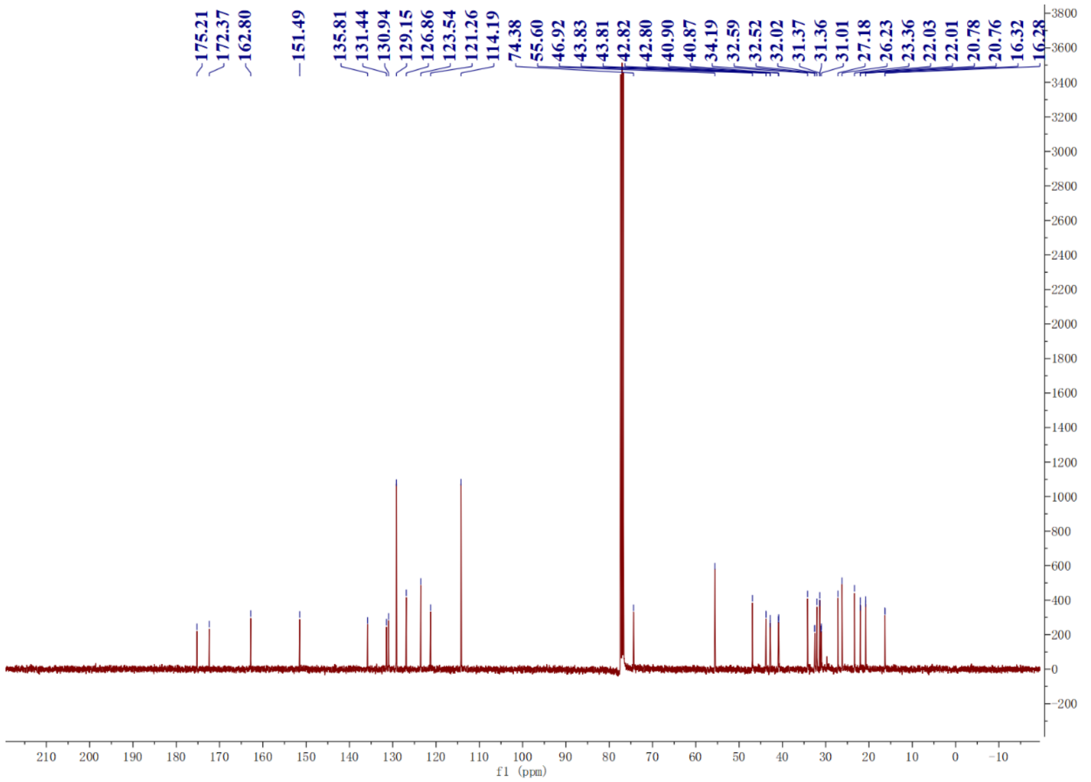

((3a*S*,5a*R*,8a*R*,8b*S*)-2,2,7,7-tetramethyltetrahydro-3a*H*-bis([1,3]dioxolo)[4,5-*b*:4',5'-*d*]pyran-3a-yl)methyl 7-((4-methoxyphenyl)sulfonamido)-4-(2-phenylquinolin-4-yl)heptanoate (66)

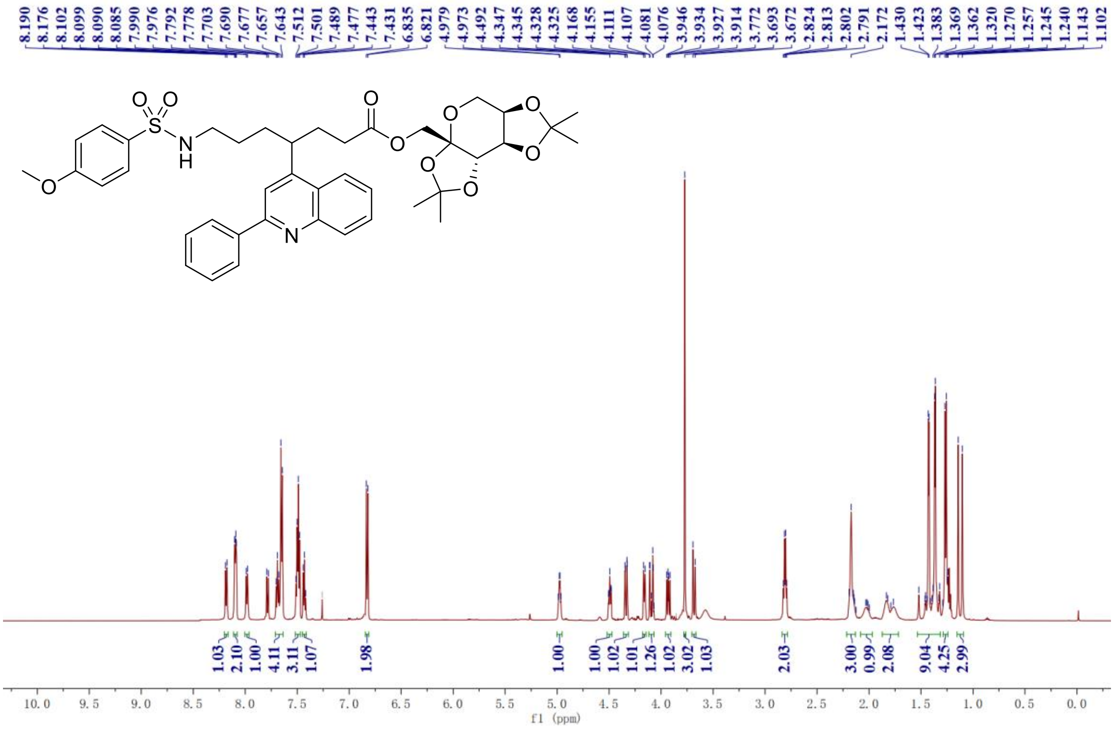

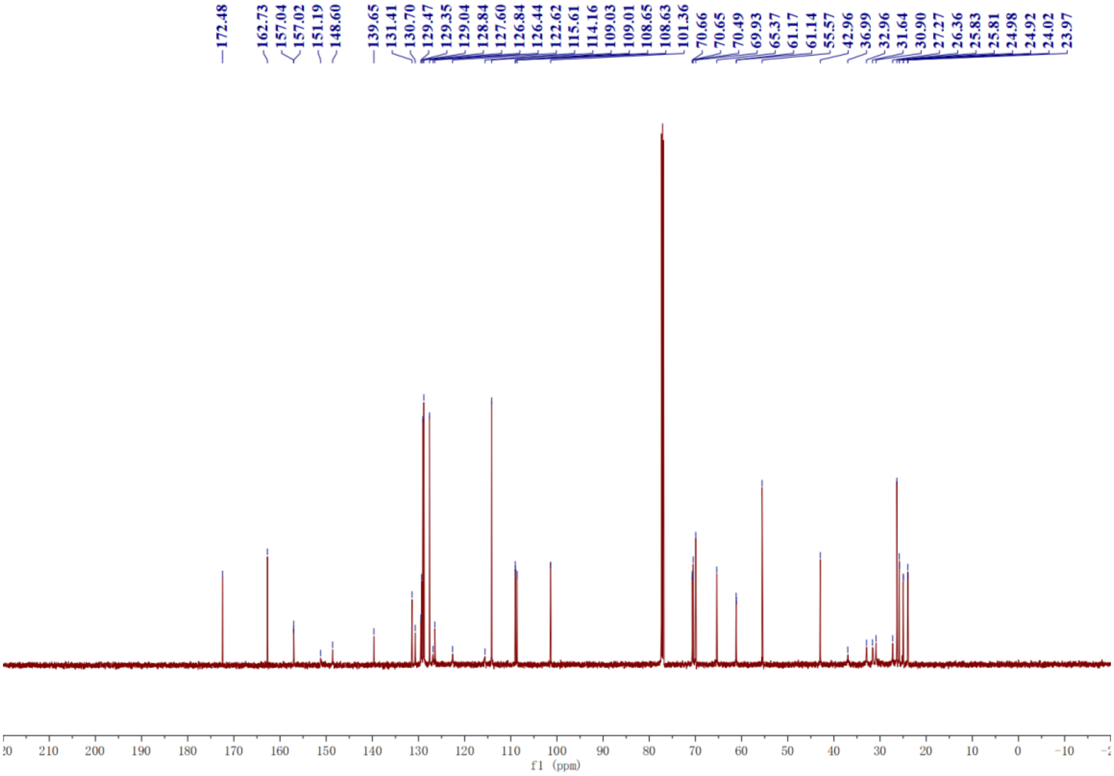

N-(6-(1,1-dioxido-3-oxobenzo[d]isothiazol-2(3H)-yl)-4-(2-phenylquinolin-4-yl)hexyl)-4-methoxybenzenesulfonamide (67)

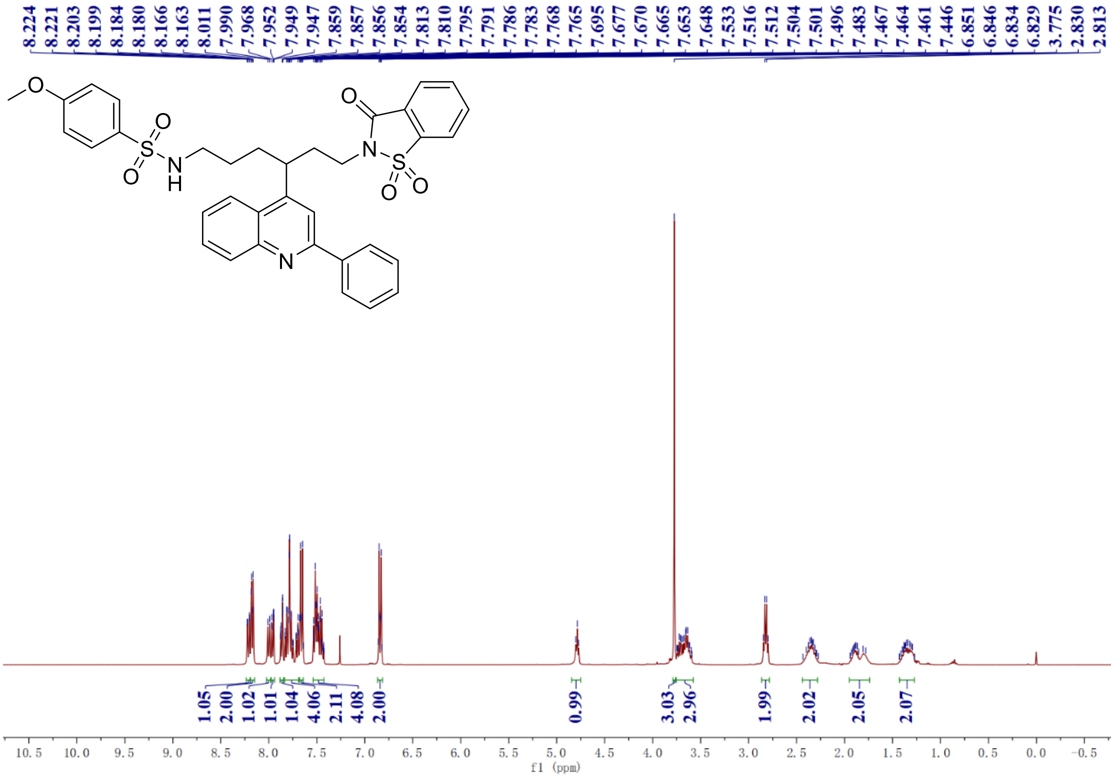

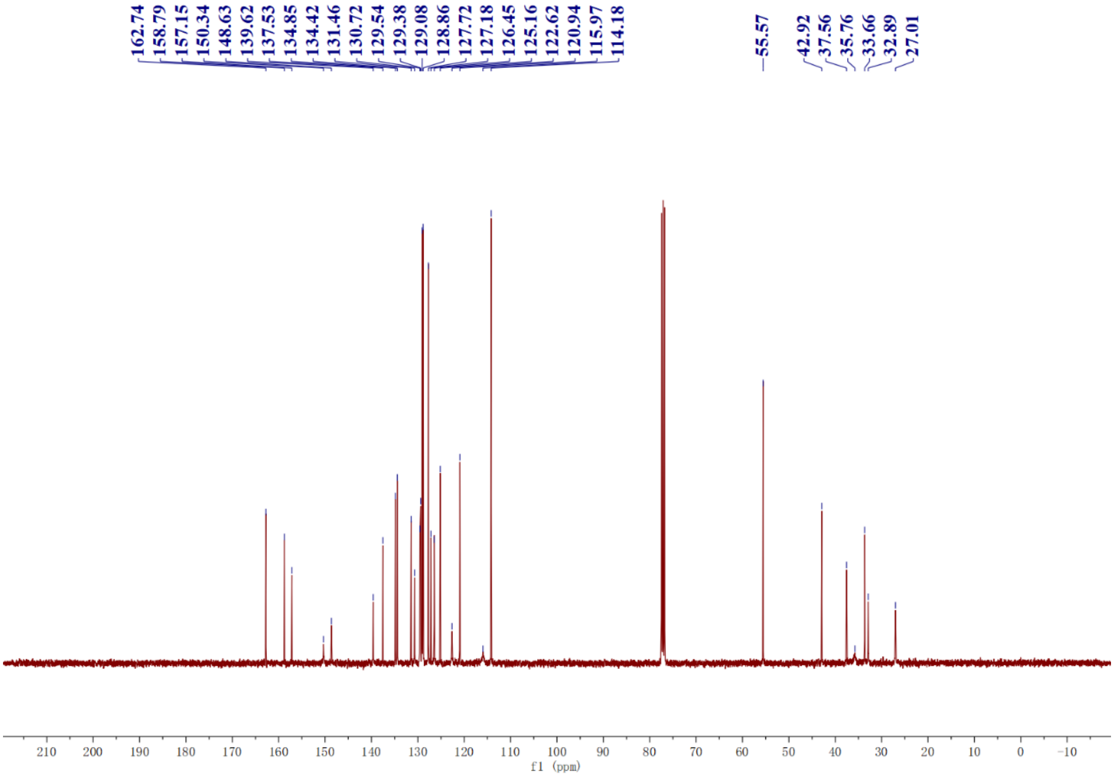

6-((4-methoxyphenyl)sulfonamido)-3-(2-phenylquinolin-4-yl)hexyl 2-(4-isobutylphenyl)propanoate (68)

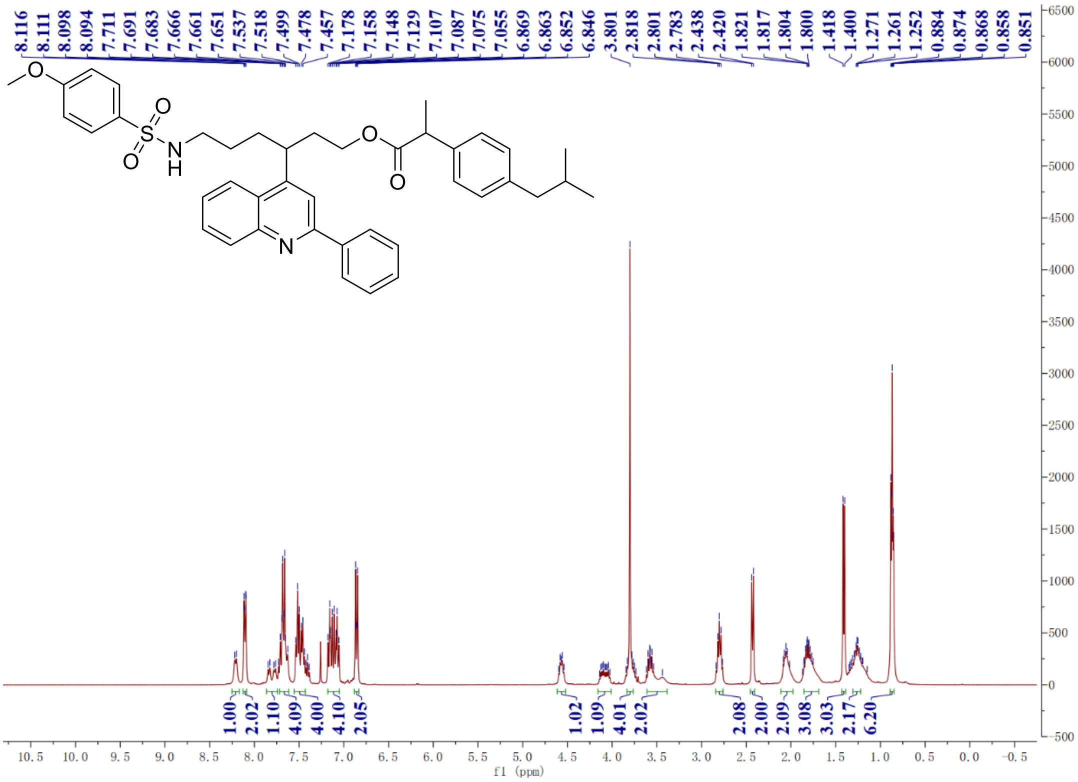

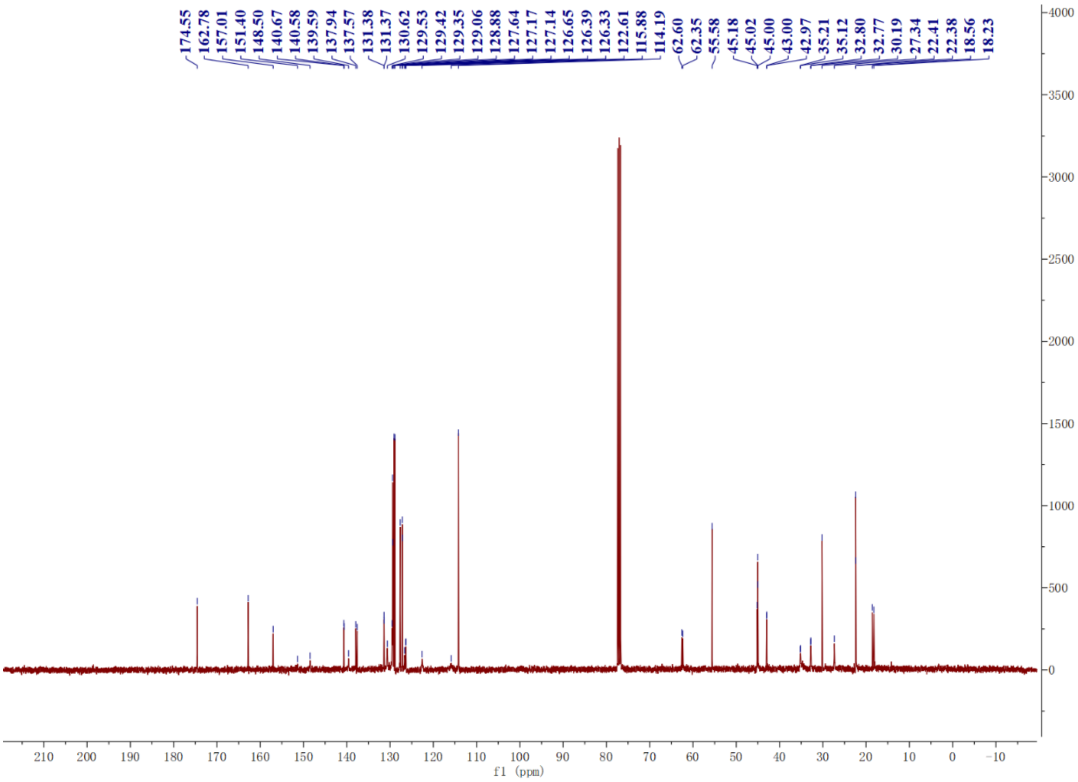

6-((4-methoxyphenyl)sulfonamido)-3-(2-phenylquinolin-4-yl)hexyl 4-([1,1'-biphenyl]-4-yl)-4-oxobutanoate (69)

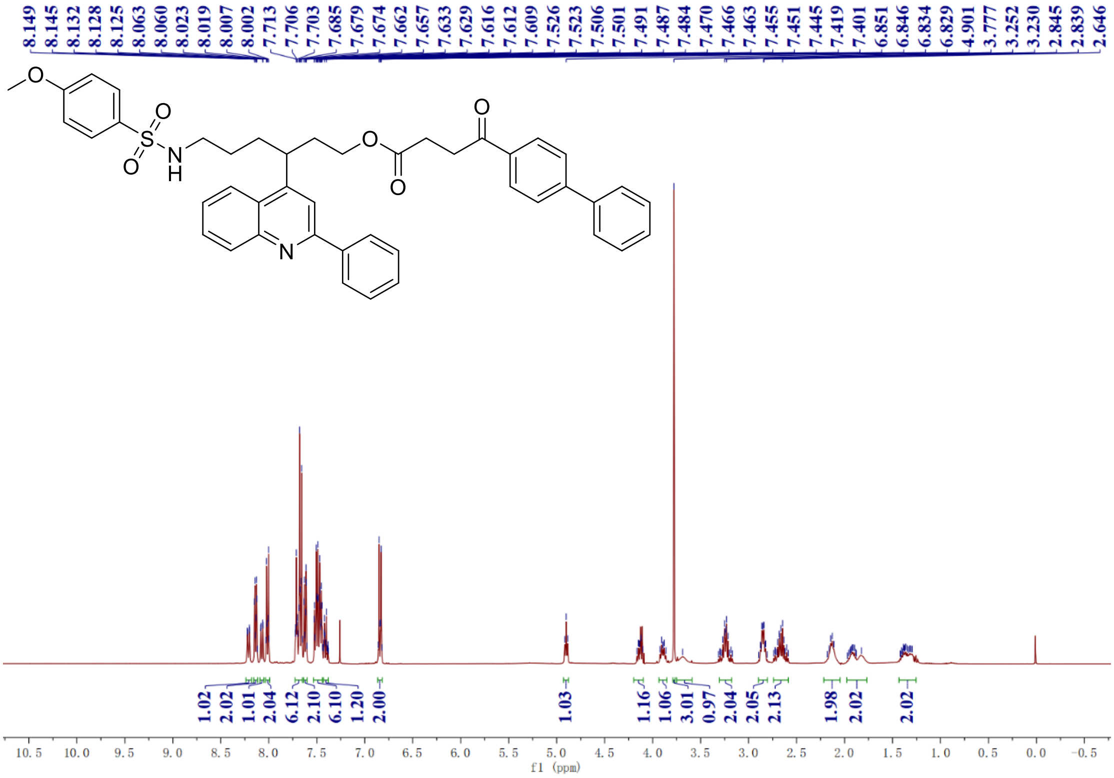

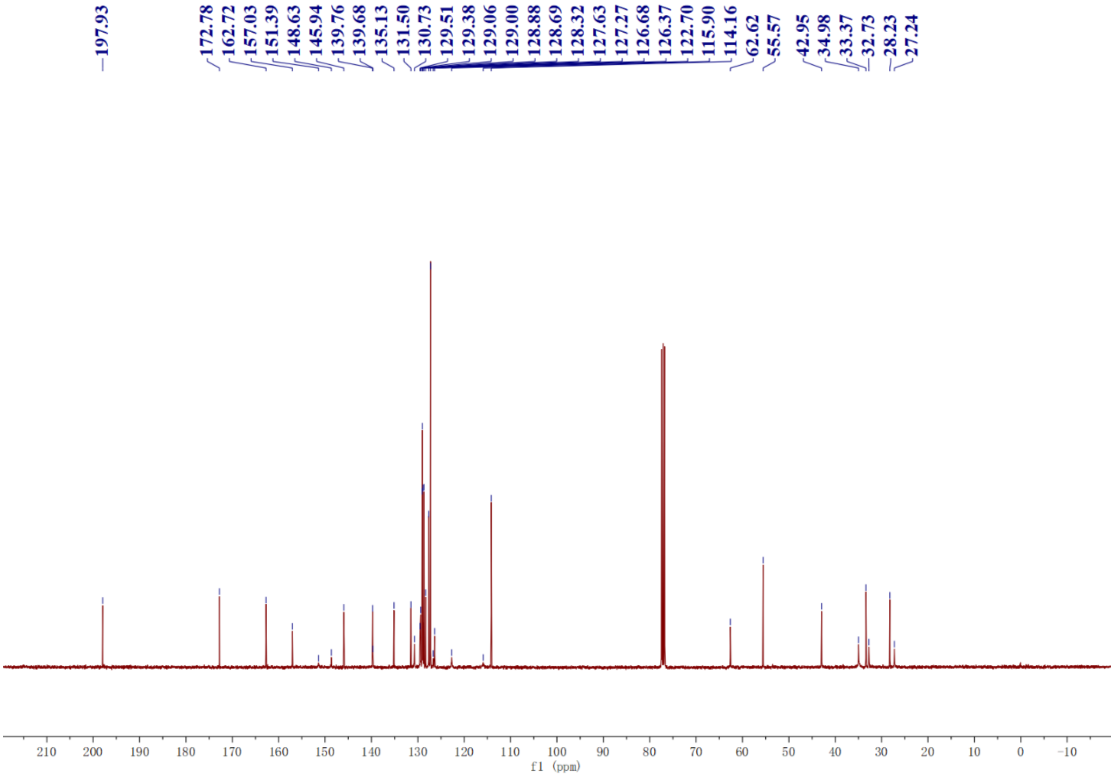

2-oxo-1-phenyl-2-((3,3,5-trimethylcyclohexyl)oxy)ethyl 7-((4-methoxyphenyl)sulfonamido)-4-(2-phenylquinolin-4-yl)heptanoate (70)

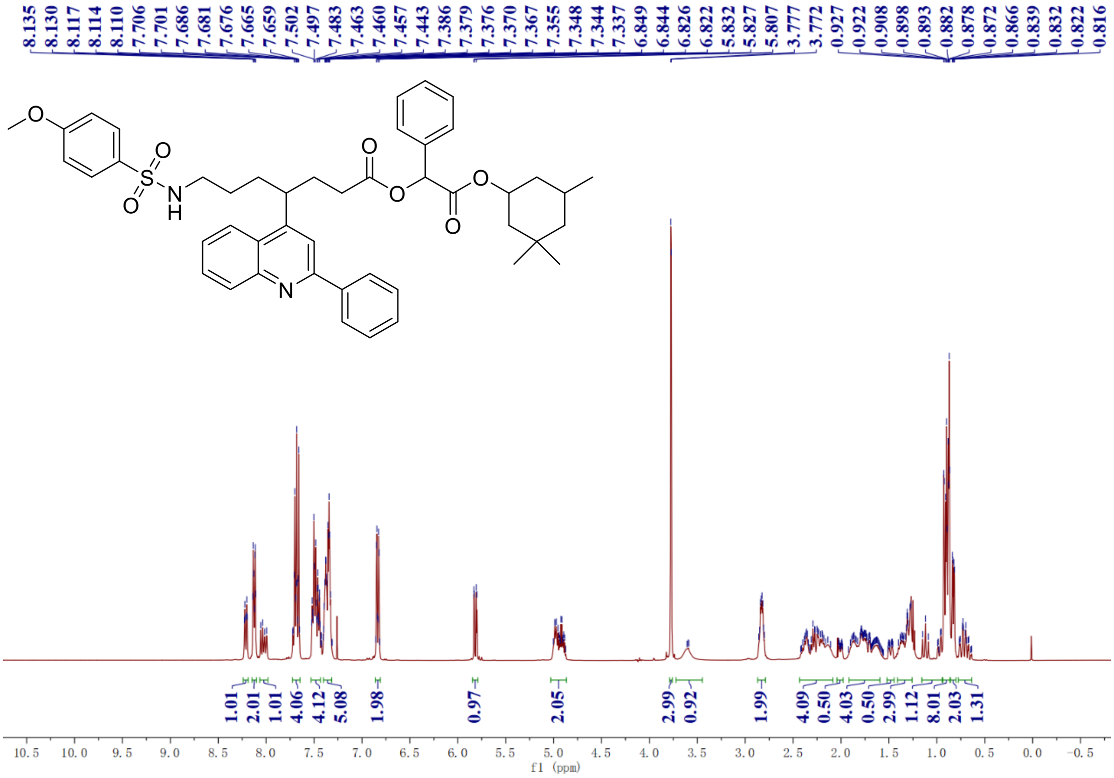

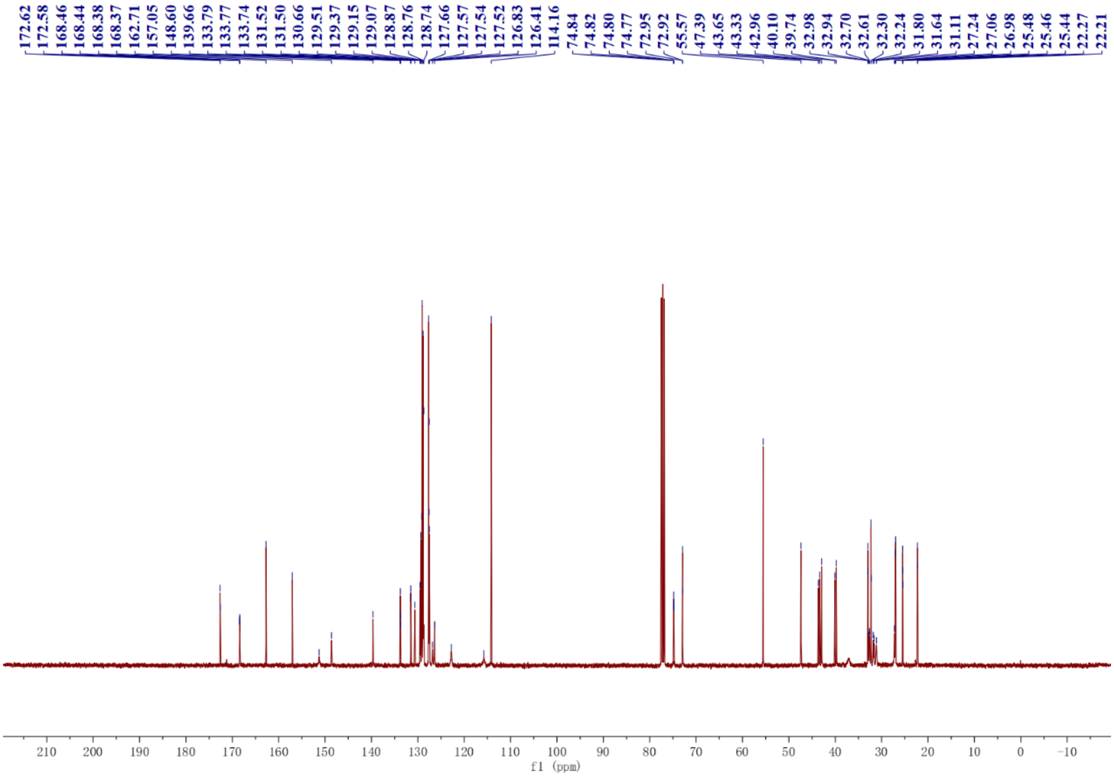

6-((4-methoxyphenyl)sulfonamido)-3-(2-phenylquinolin-4-yl)hexyl 2-(4-(4-chlorobenzoyl)phenoxy)-2-methylpropanoate (71)

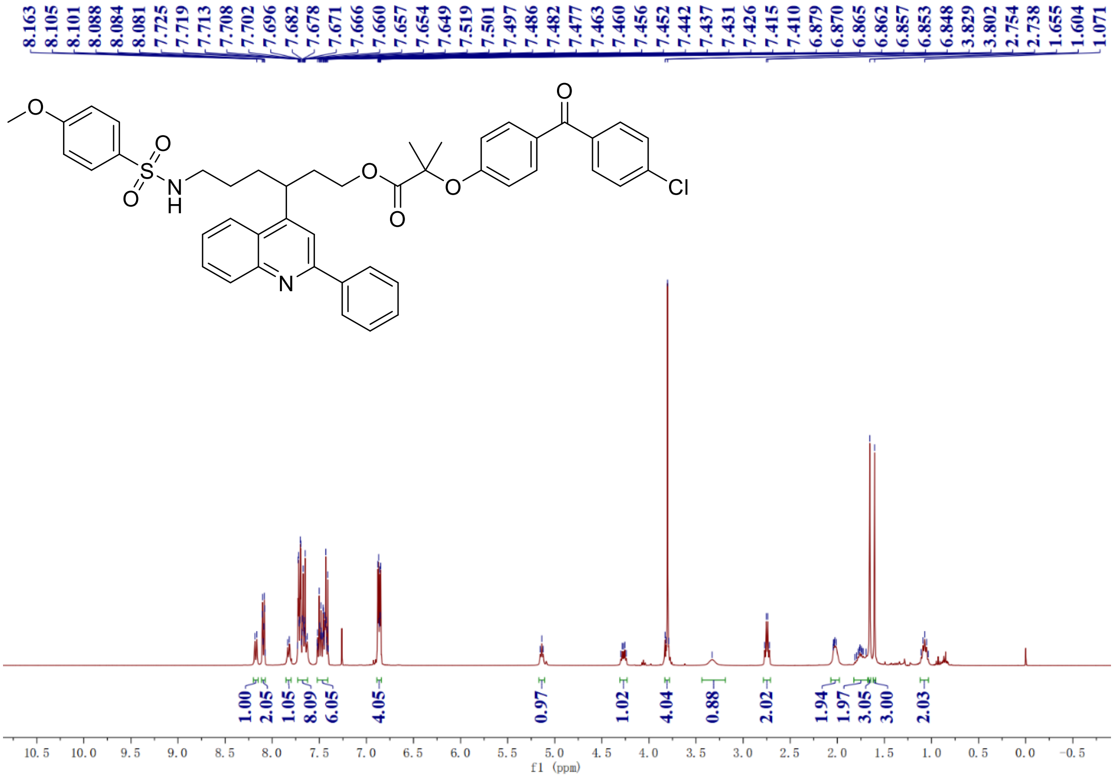

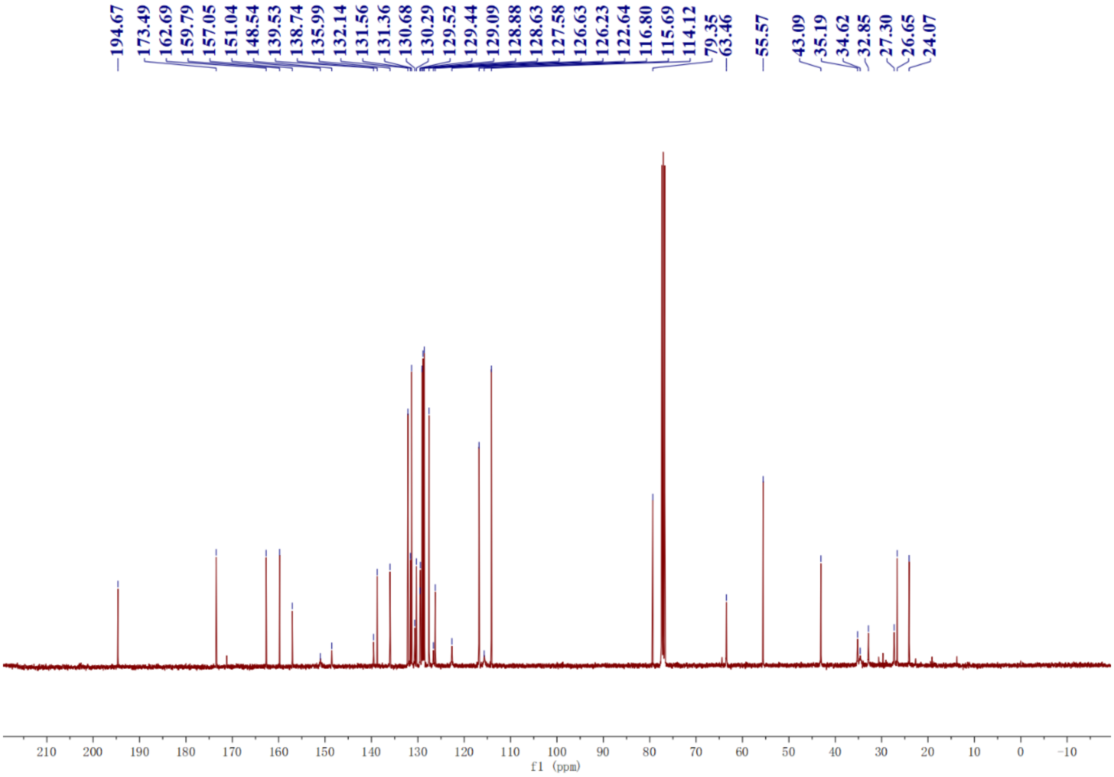

N-(6-((4-methoxyphenyl)sulfonamido)-3-(2-phenylquinolin-4-yl)hexyl)-N-methyl-4-(5-(p-tolyl)-3-(trifluoromethyl)-1H-pyrazol-1-yl)benzenesulfonamide (72)

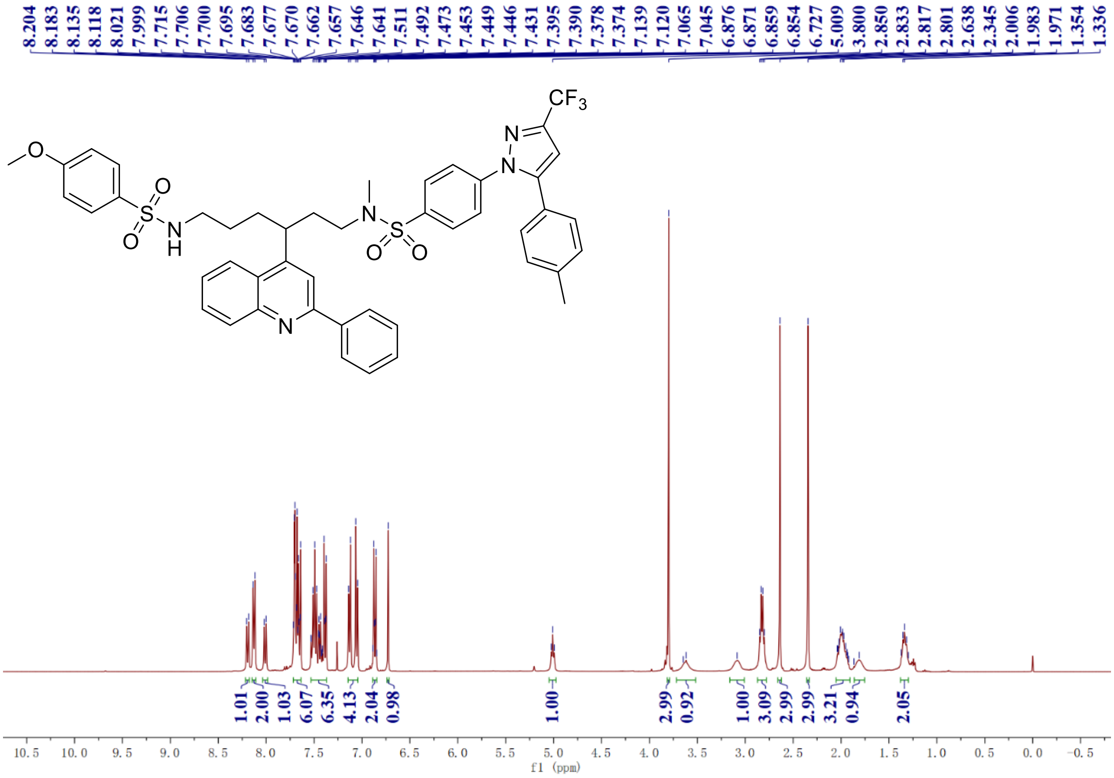

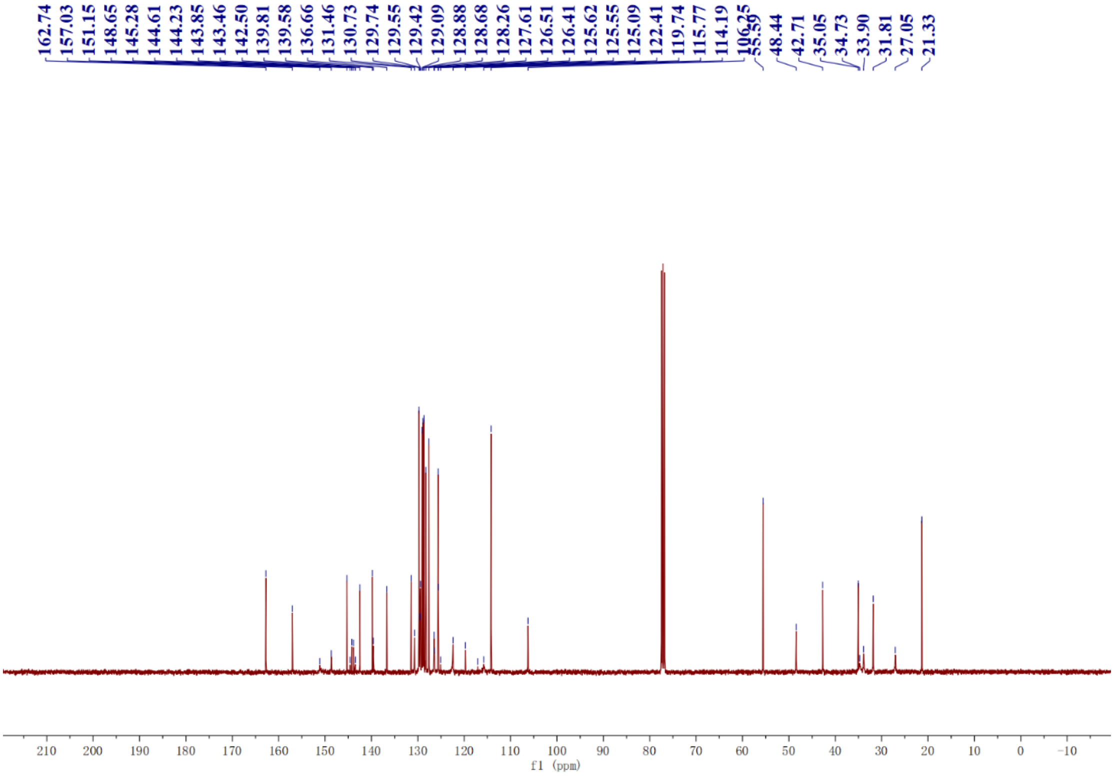

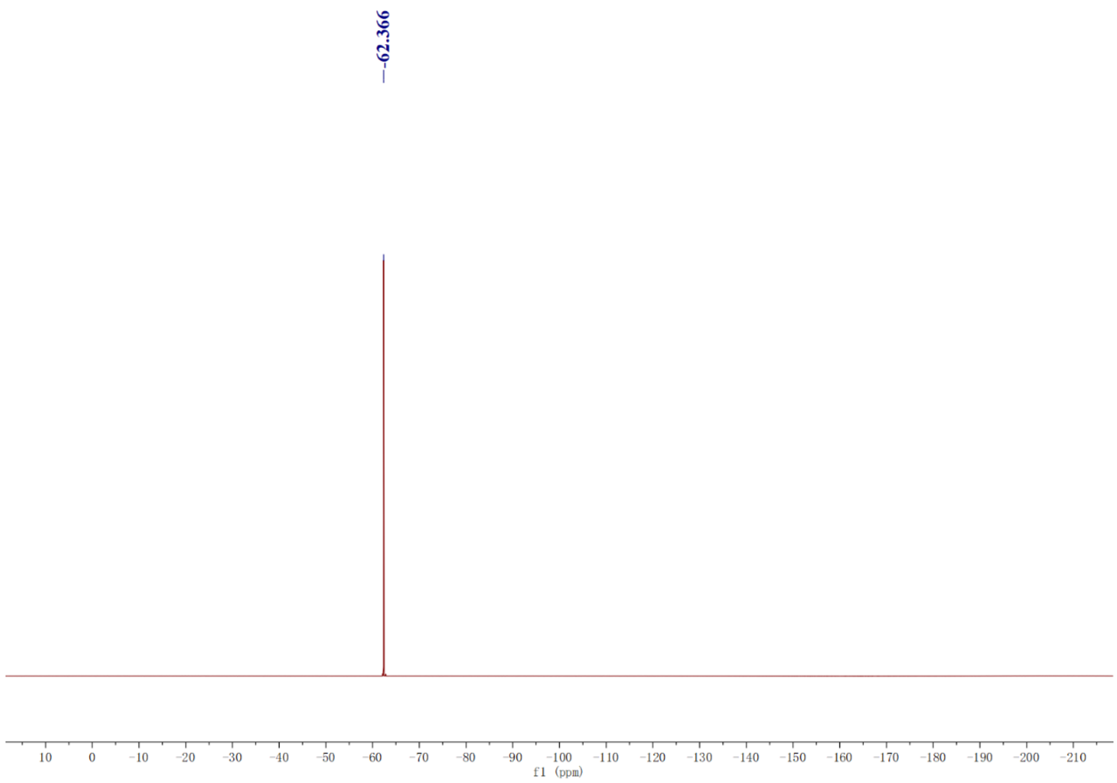
-62.366
10 0 -10 -20 -30 -40 -50 -60 -70 -80 -90 -100 -110 -120 -130 -140 -150 -160 -170 -180 -190 -200 -210
f1 (ppm)

6-chloro-2-cyclopentylbenzo[*d*]thiazole (73)

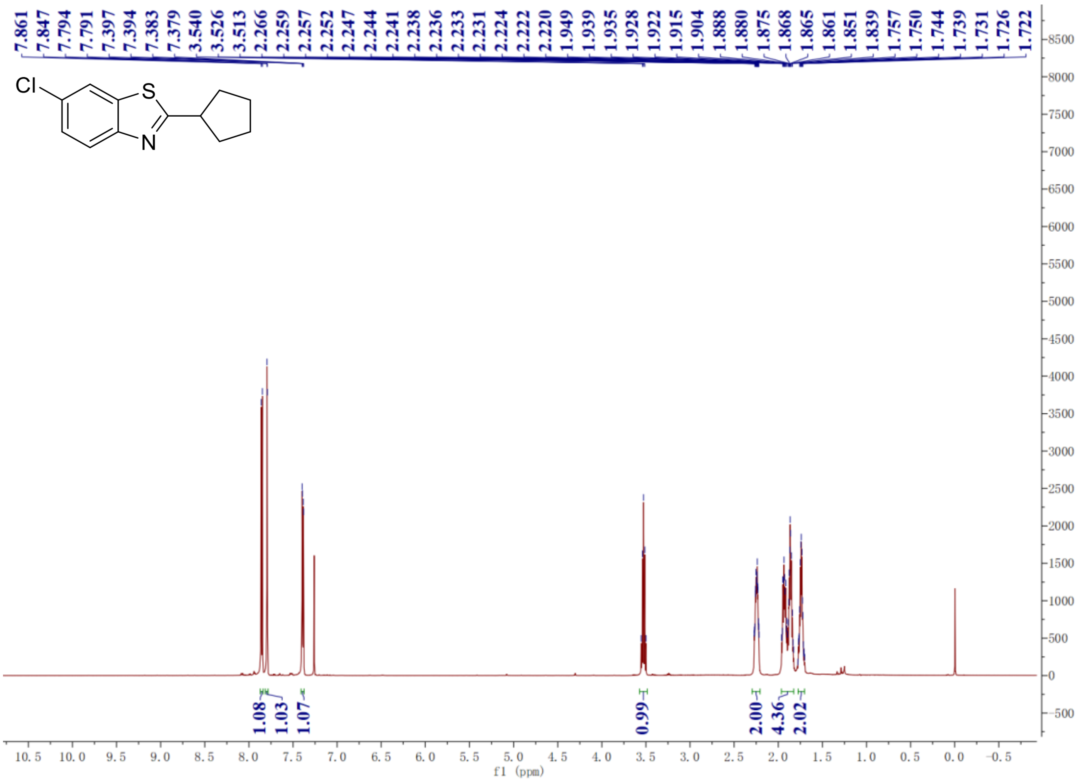

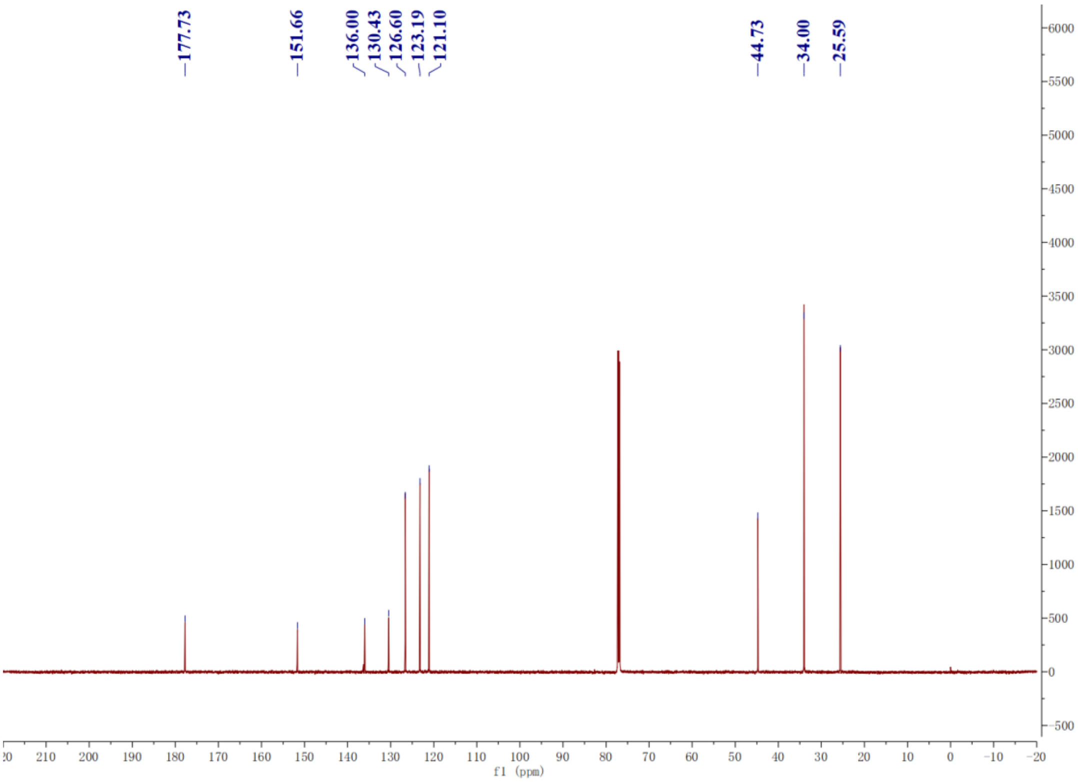

6-chloro-2-cyclooctylbenzo[*d*]thiazole (74)

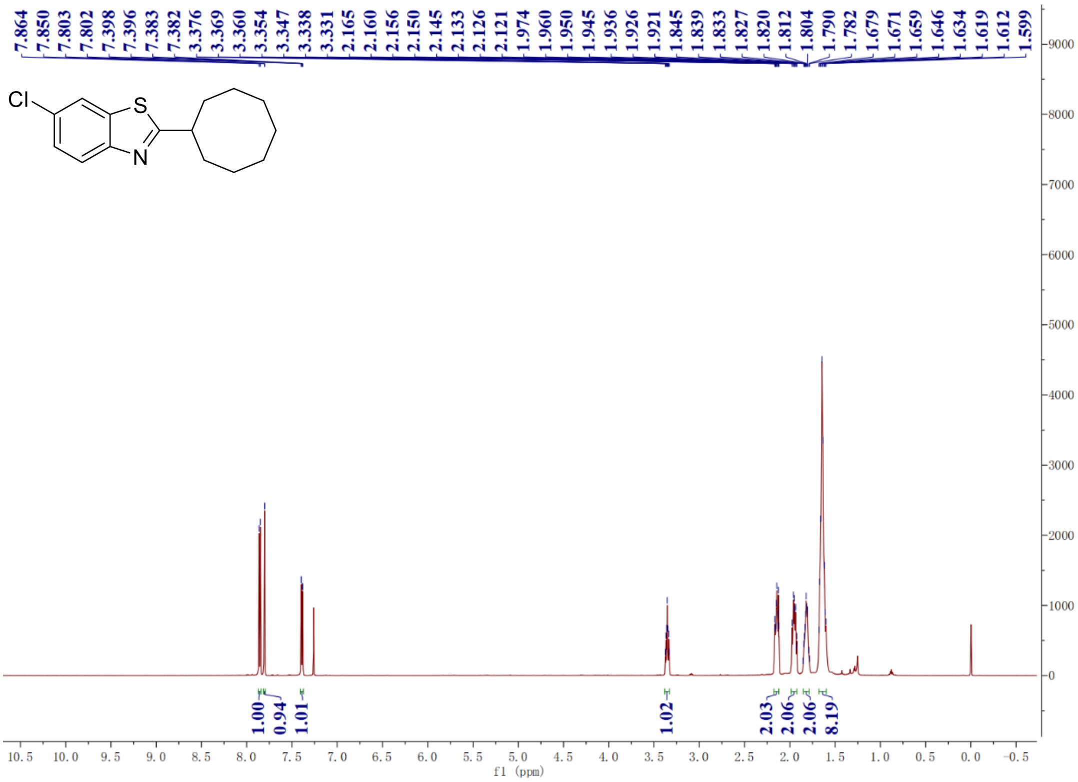

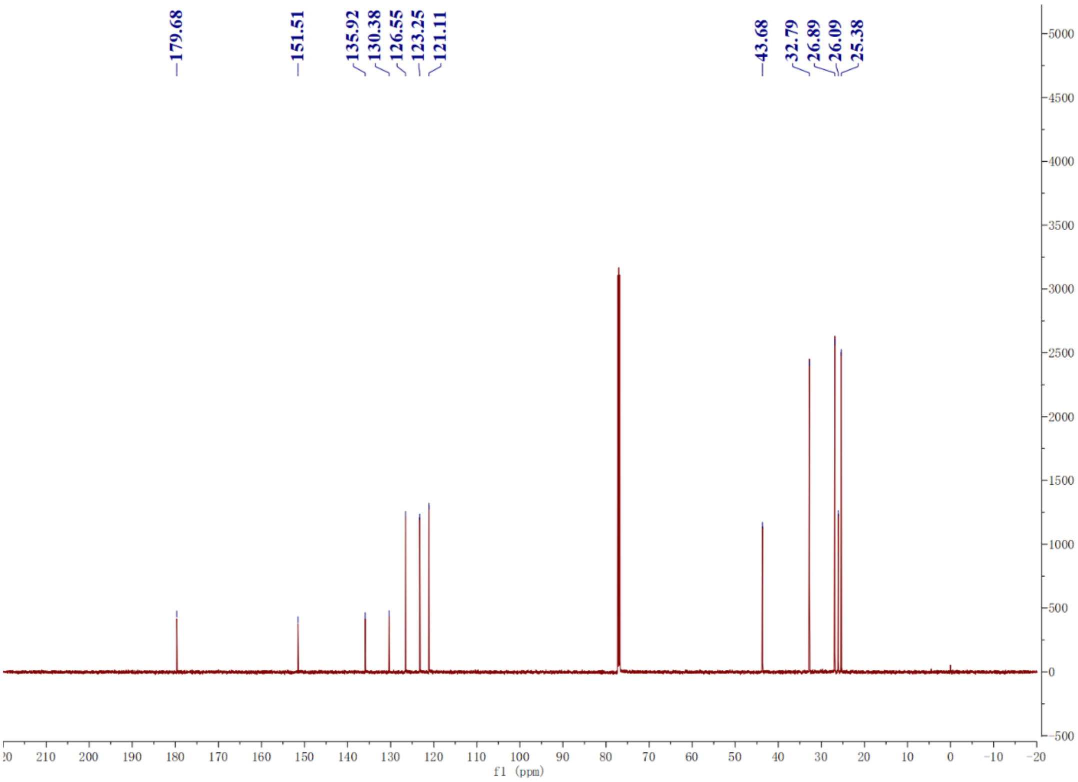

6-chloro-2-cyclododecylbenzo[*d*]thiazole (75)

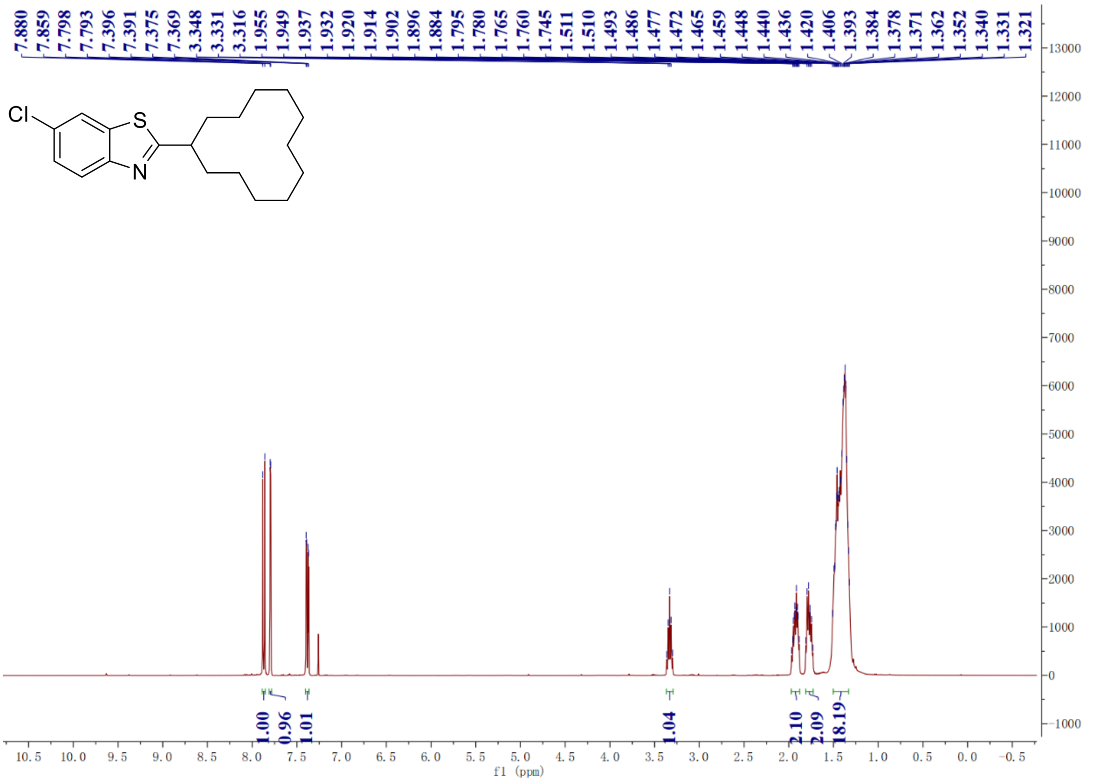

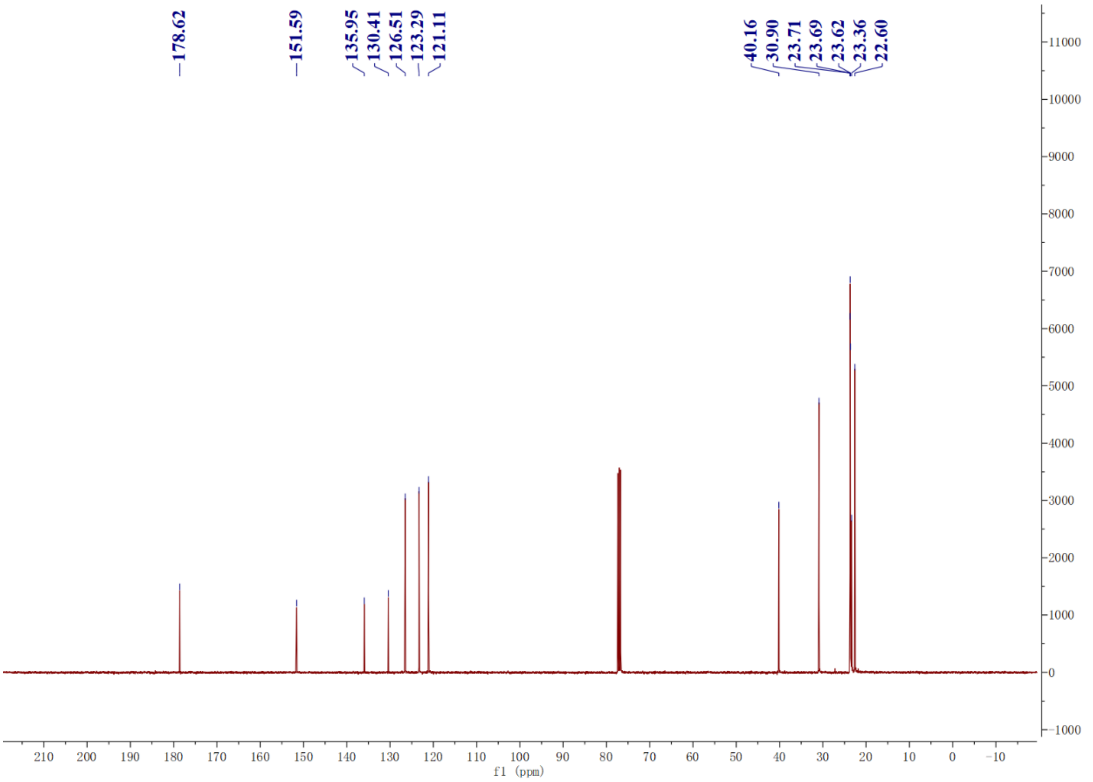

2-cyclohexyl-4-methylquinoline (76)

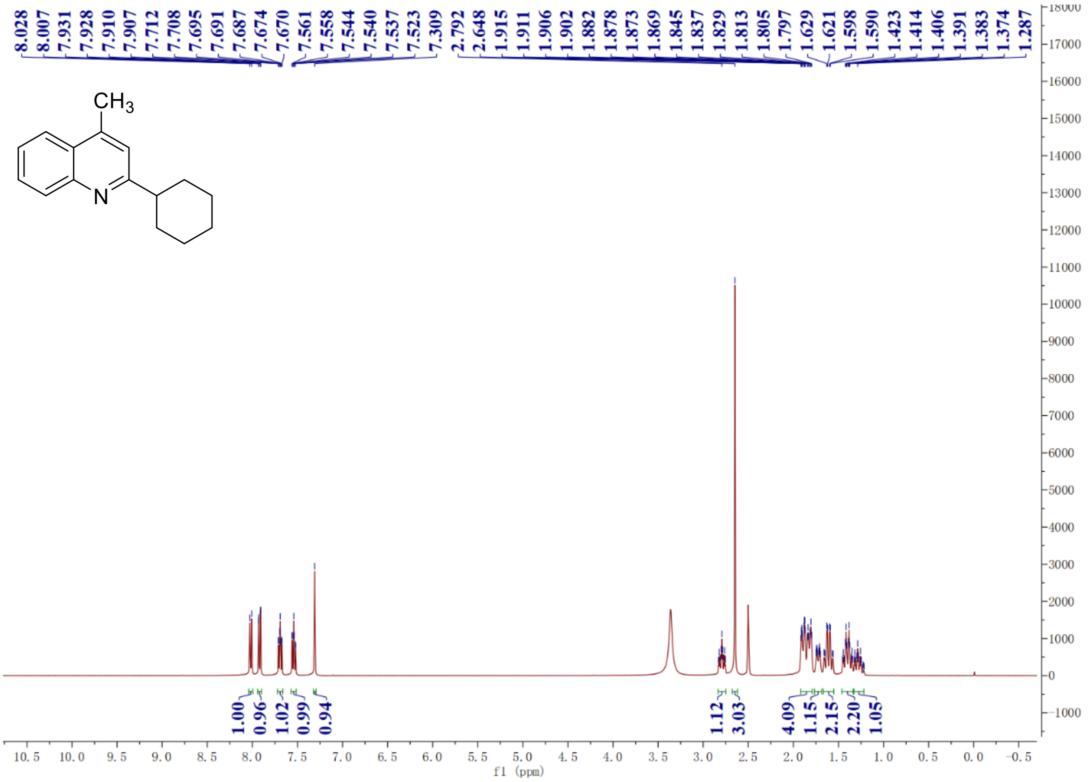

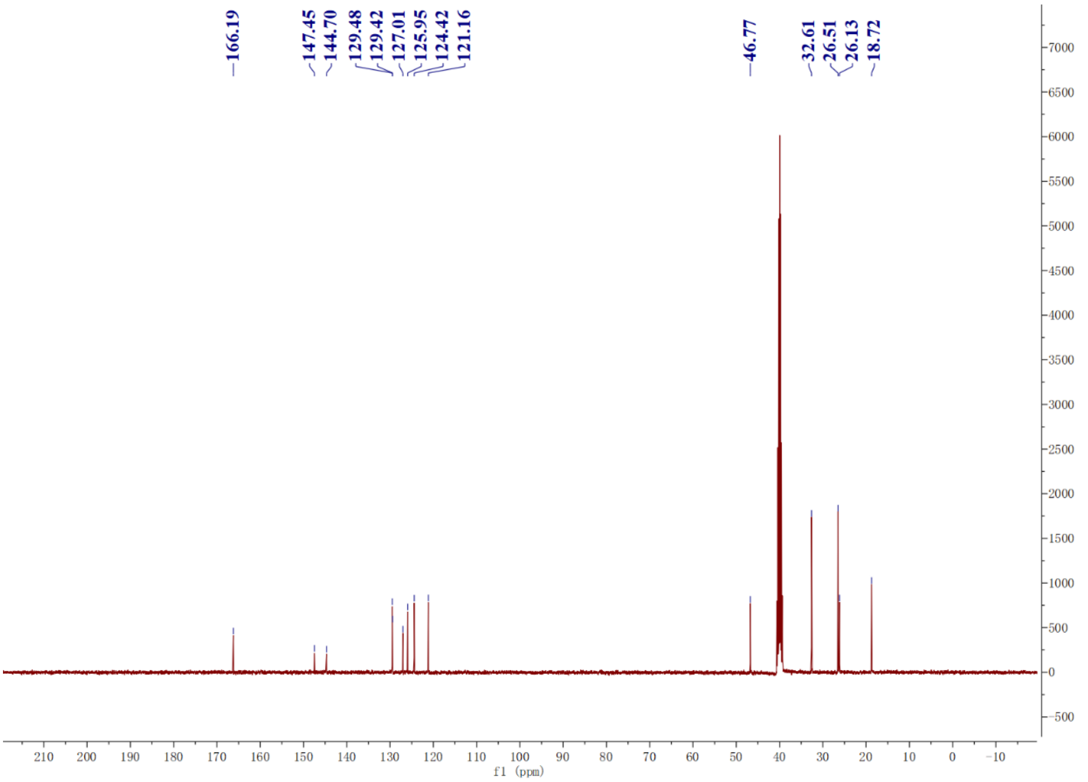

4-(1,2-dimethoxyethyl)-2-phenylquinoline (77)

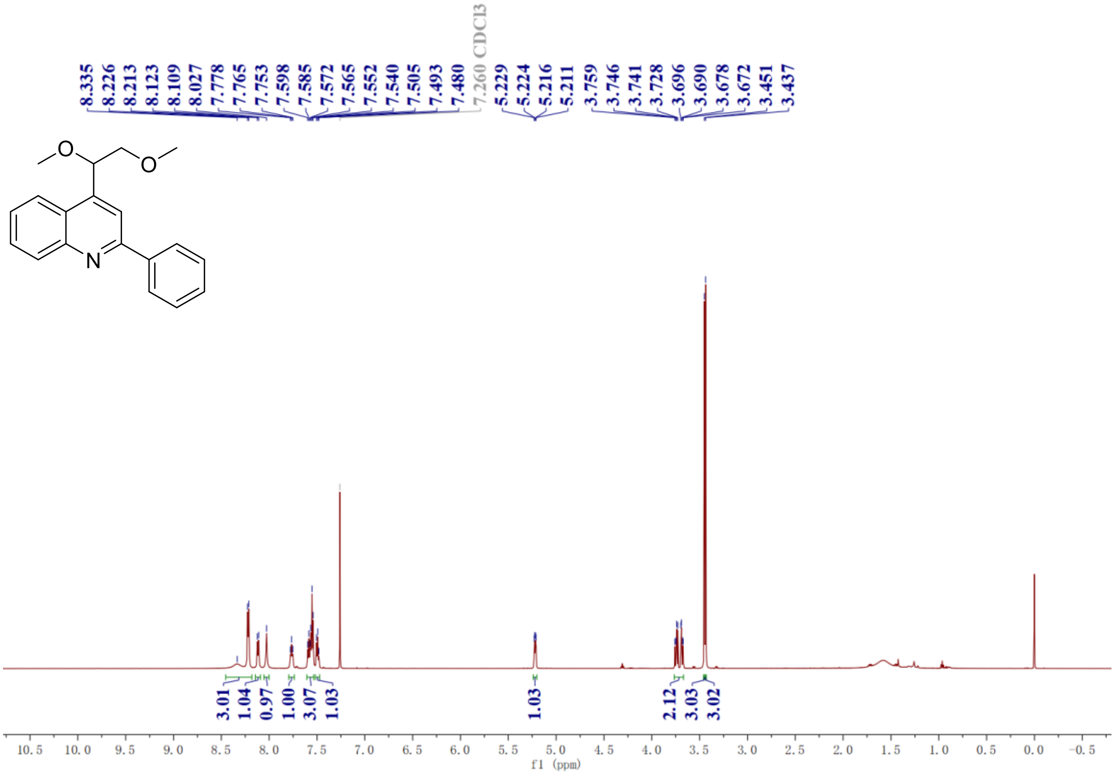

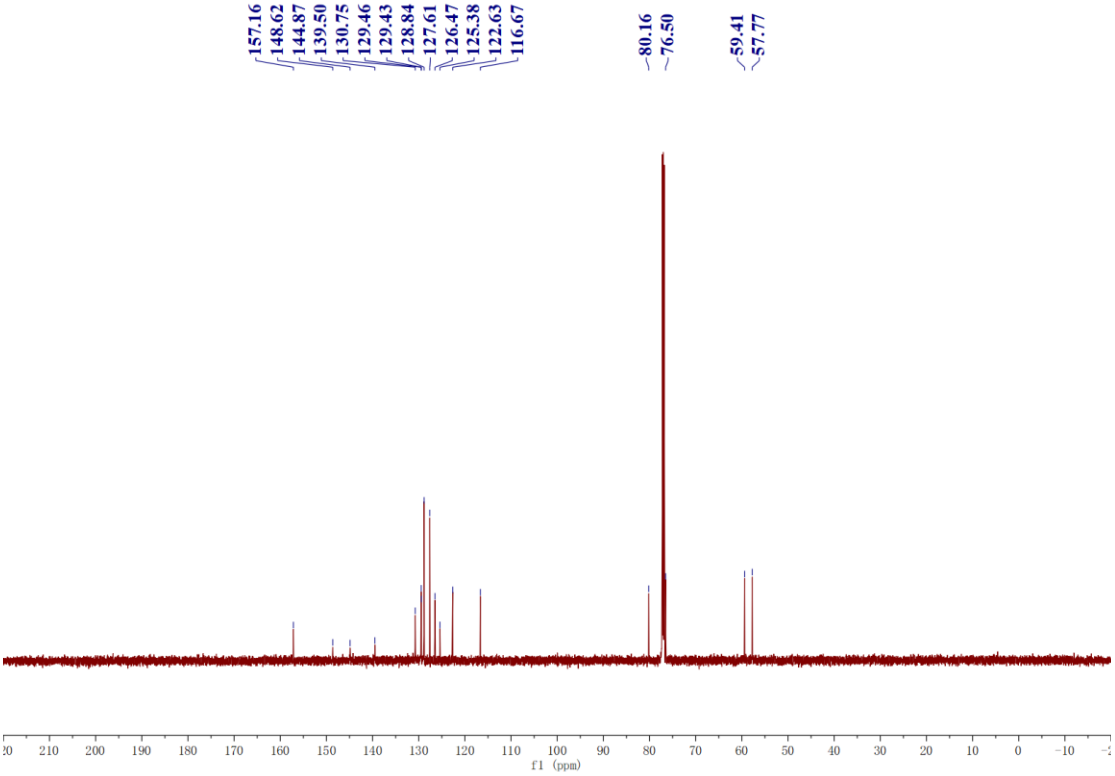

4-((2-methoxyethoxy)methyl)-2-phenylquinoline (78)

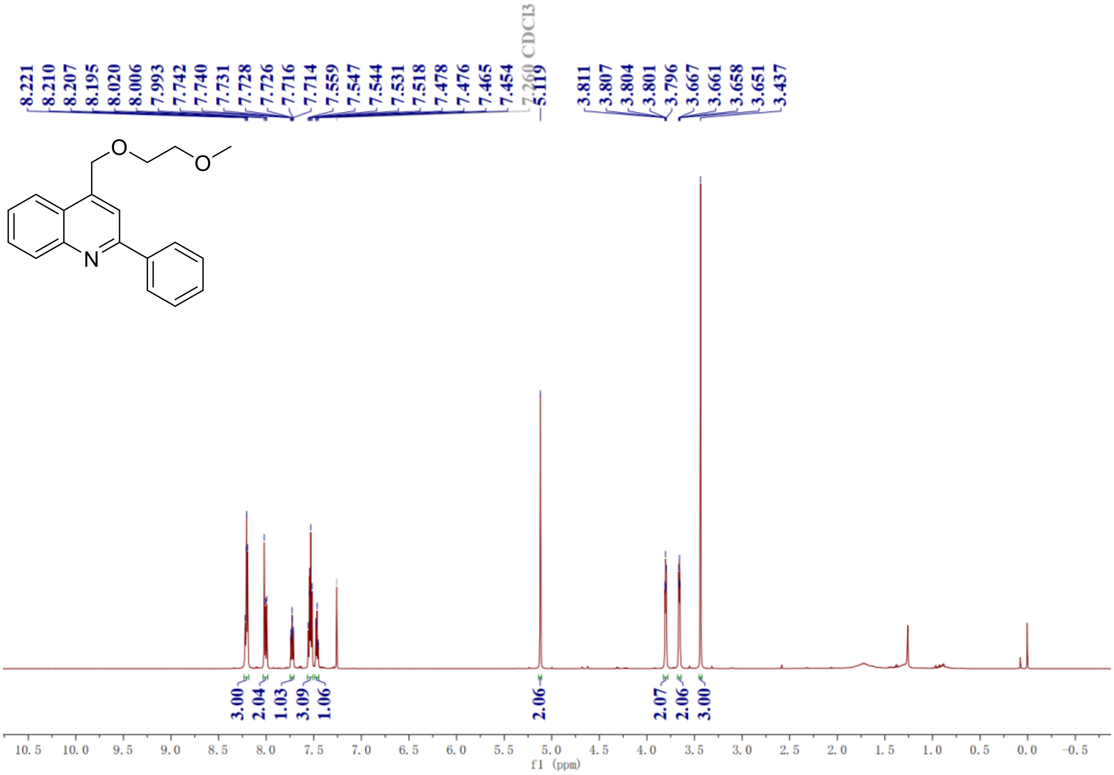

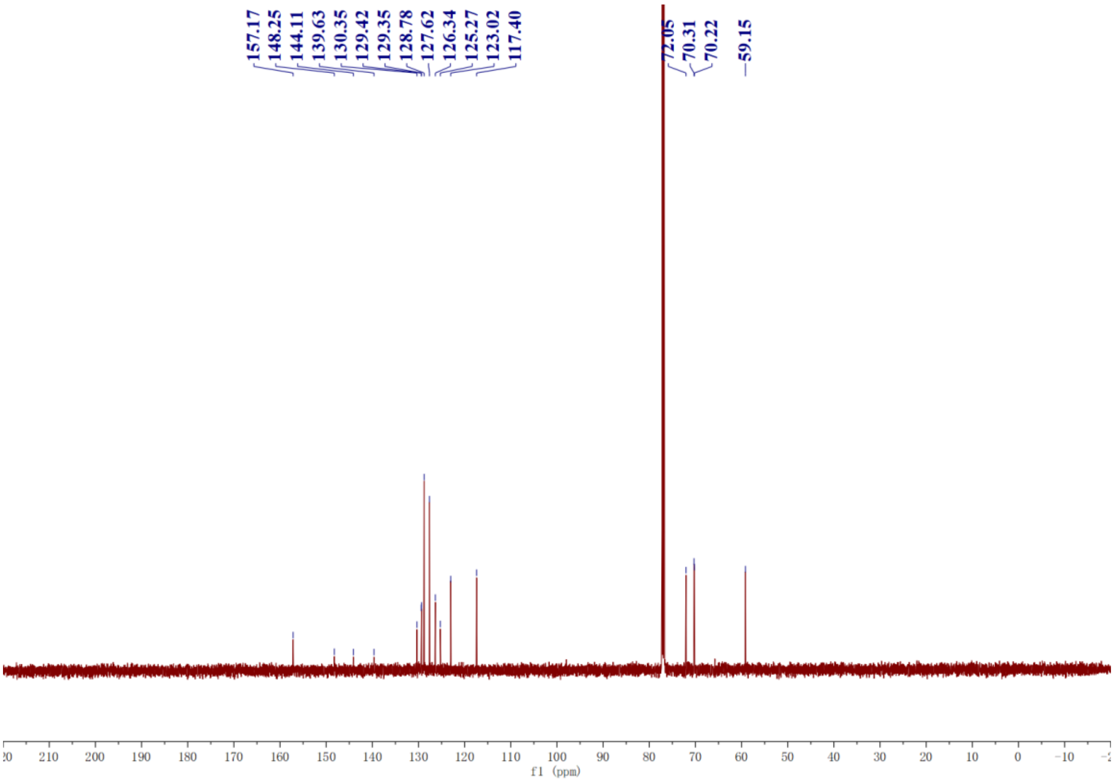

4-(*tert*-butoxymethyl)-2-phenylquinoline (79)

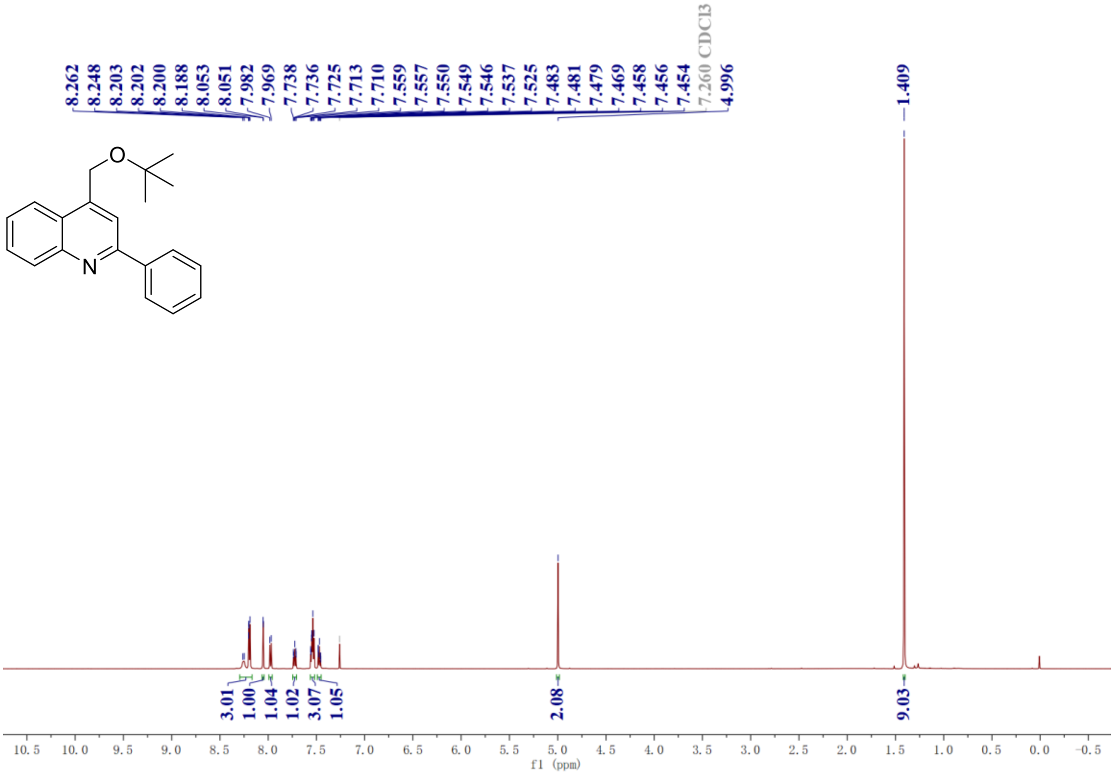

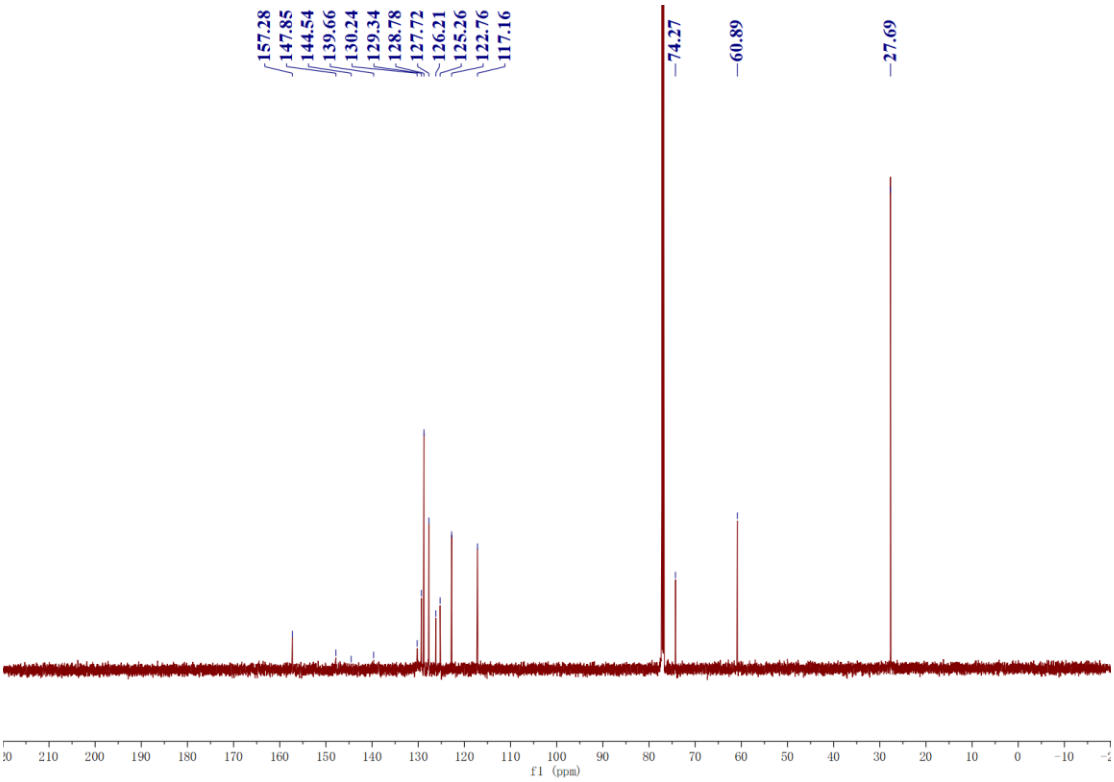

4-(1,4-dioxan-2-yl)-2-phenylquinoline (80)

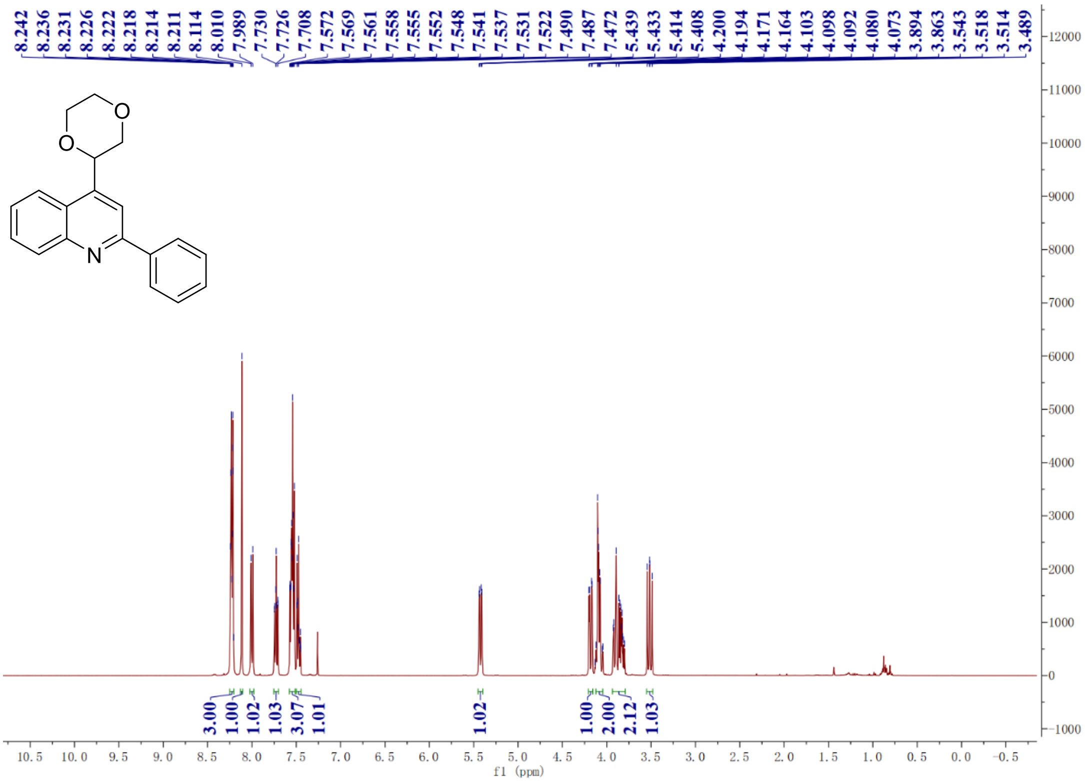

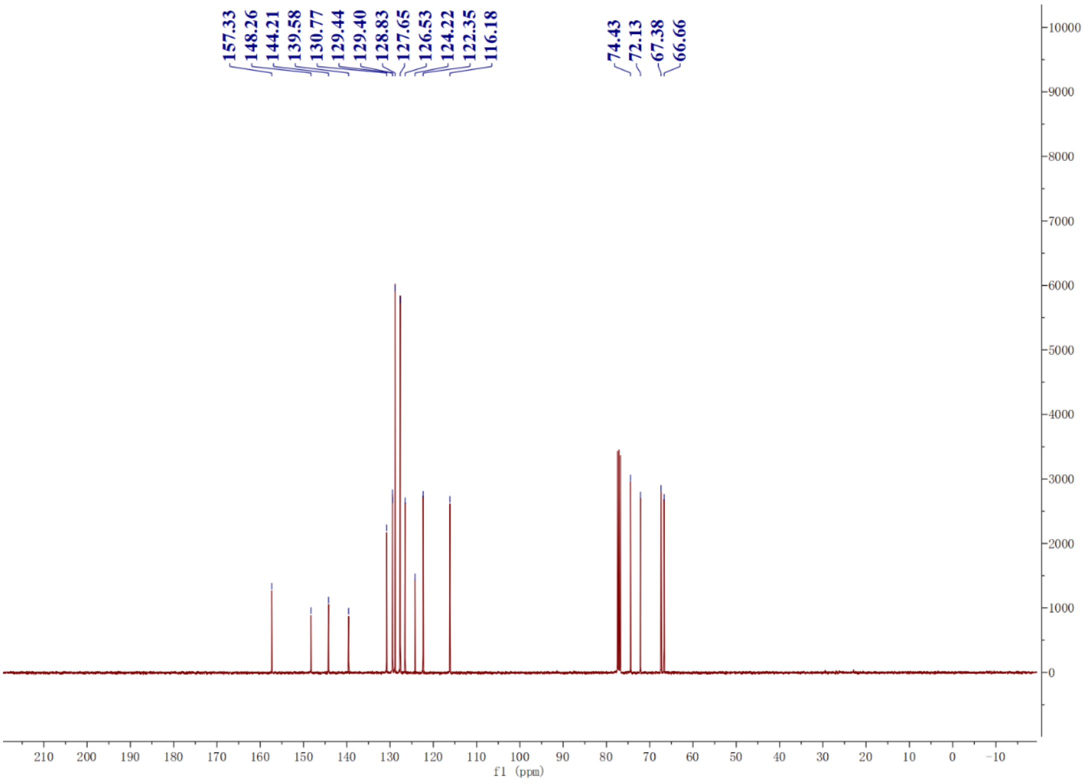

methyl 2-(2-phenylquinolin-4-yl)tetrahydro-2*H*-pyran-4-carboxylate (81)

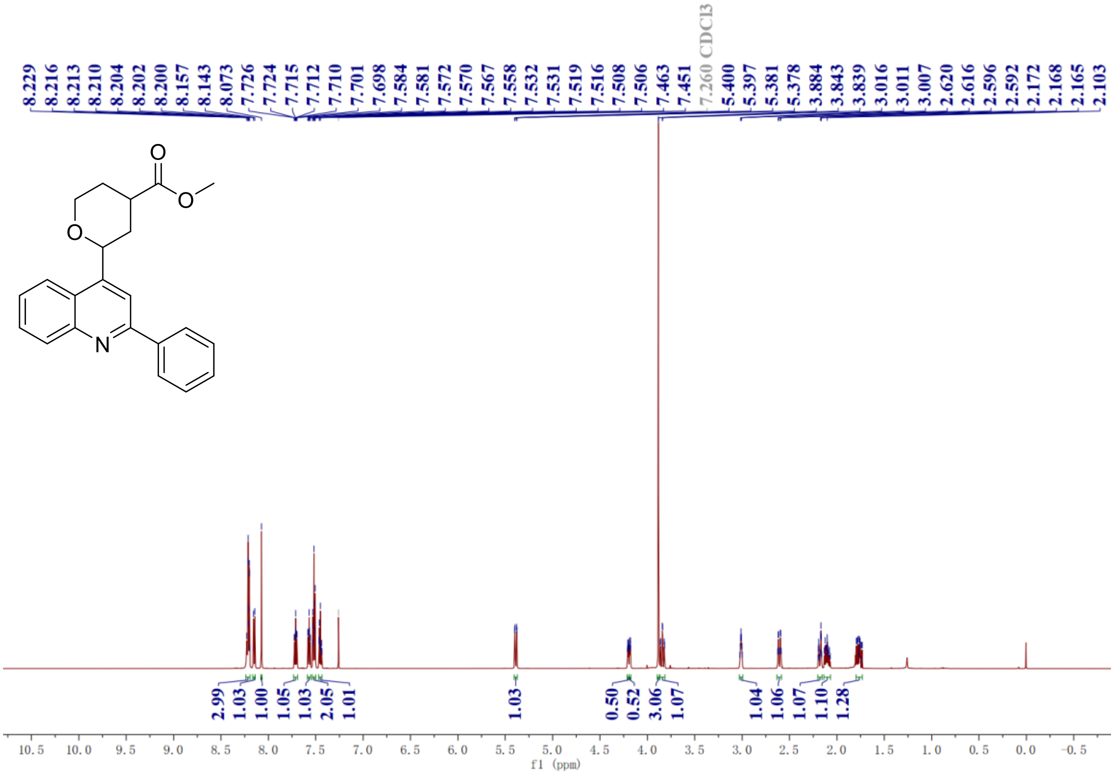

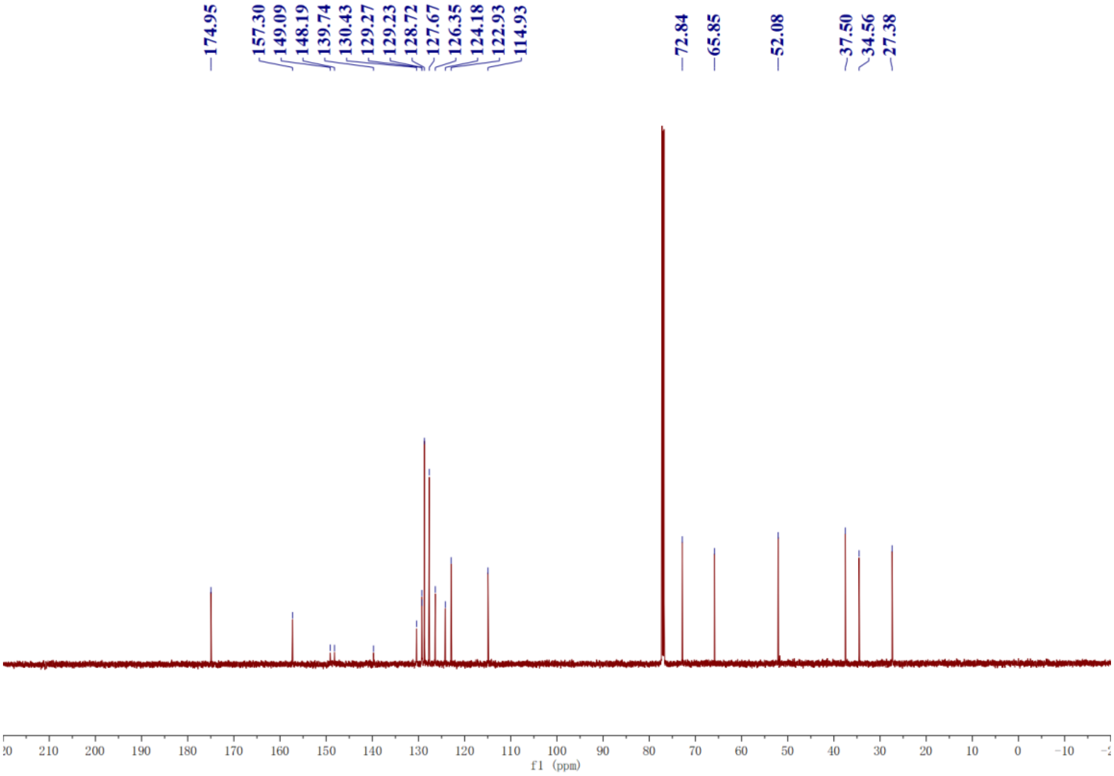

2-(2-phenylquinolin-4-yl)ethan-1-ol (82)

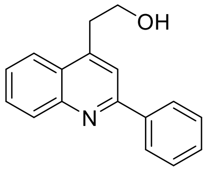

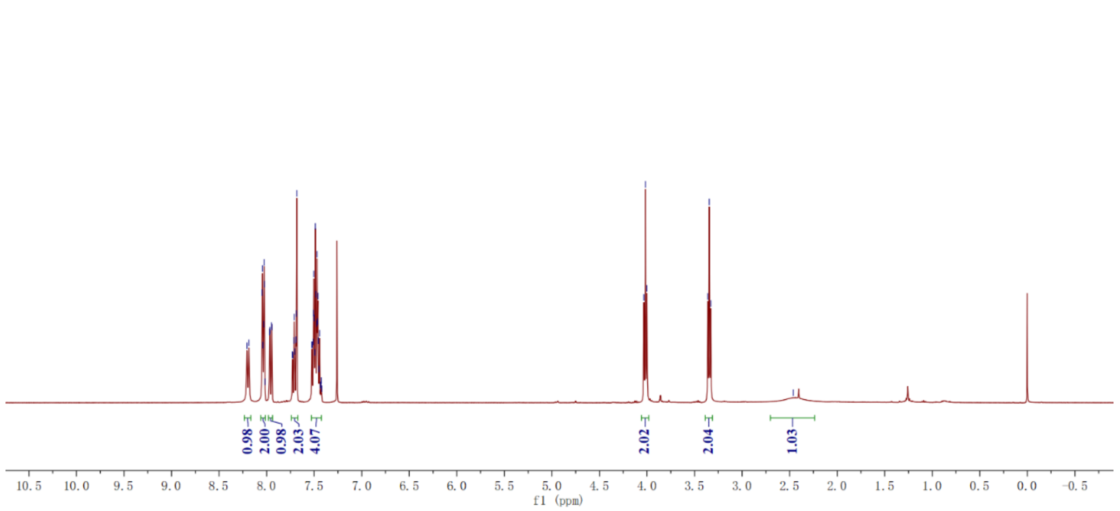

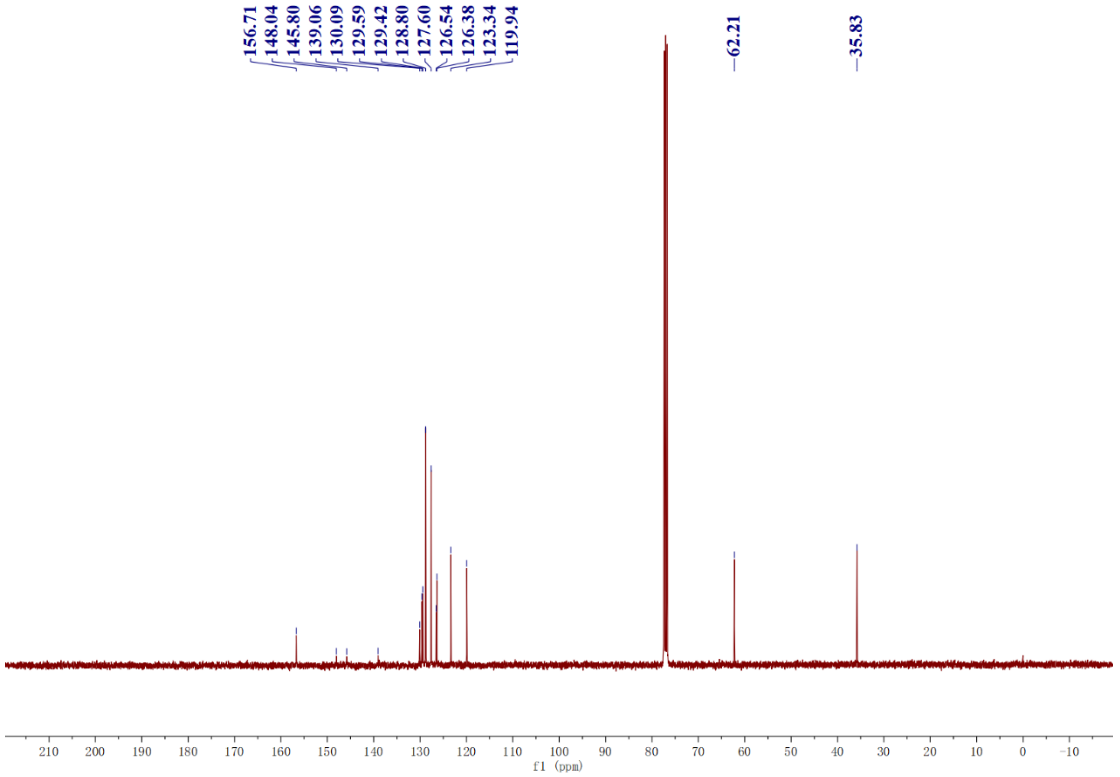

4-(2-phenylquinolin-4-yl)butan-1-ol (83)

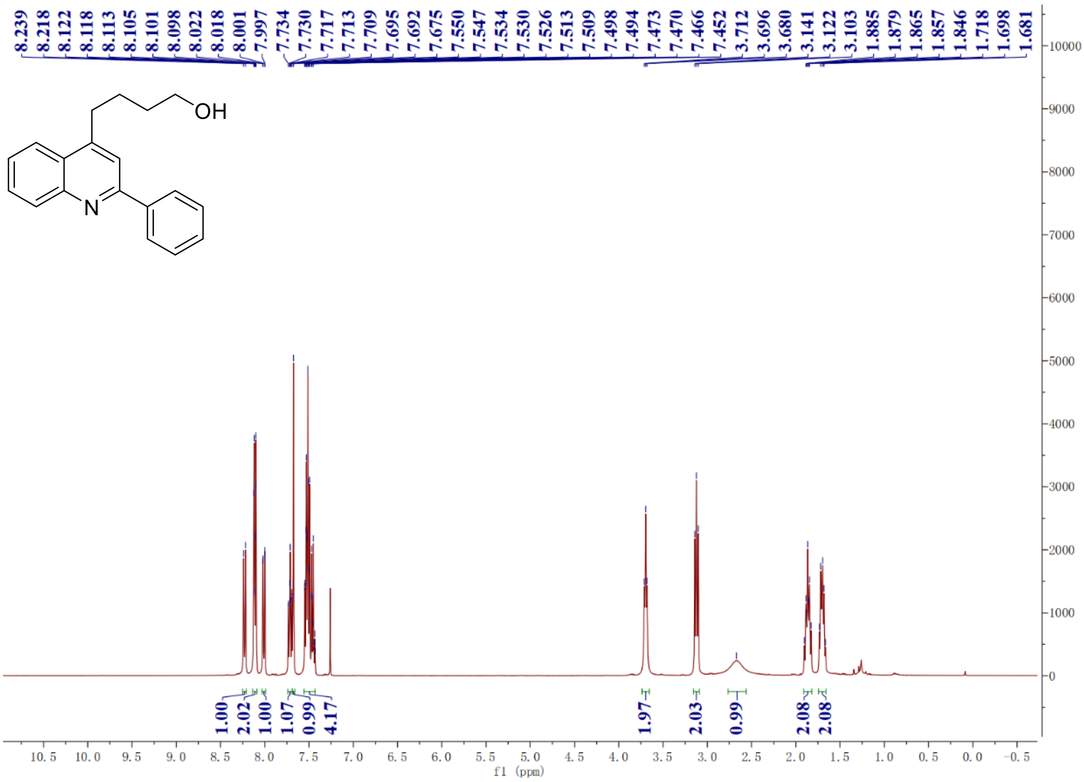

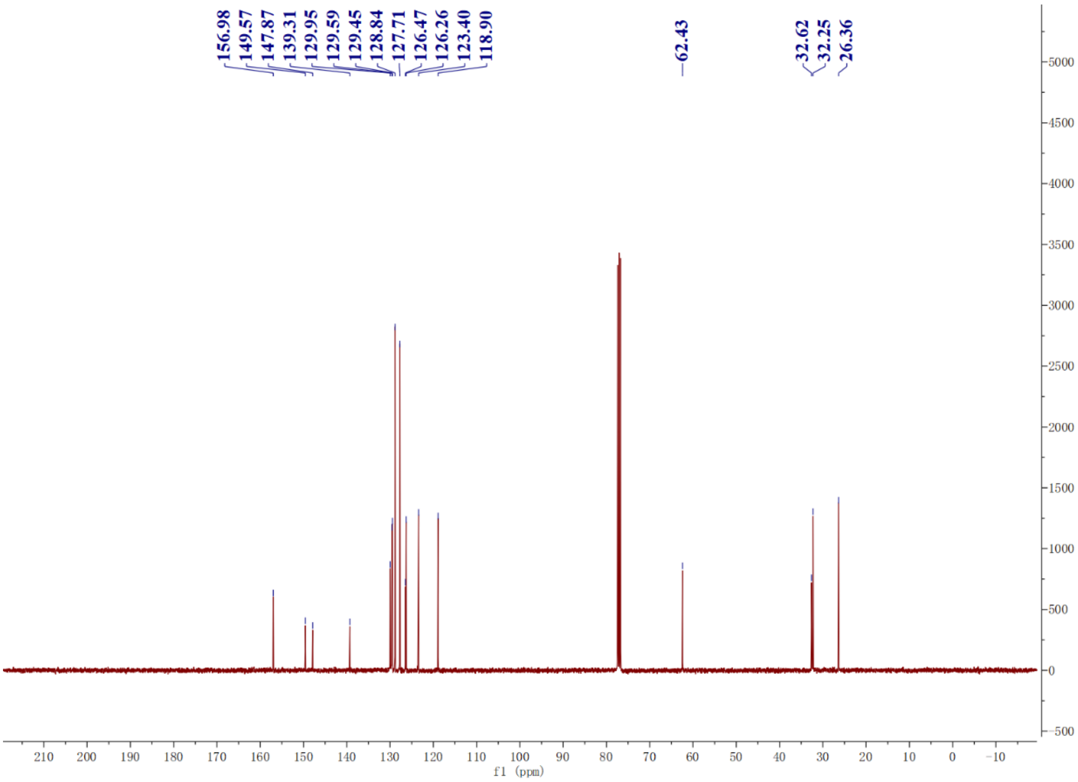

5-(2-phenylquinolin-4-yl)pentan-2-ol (84)

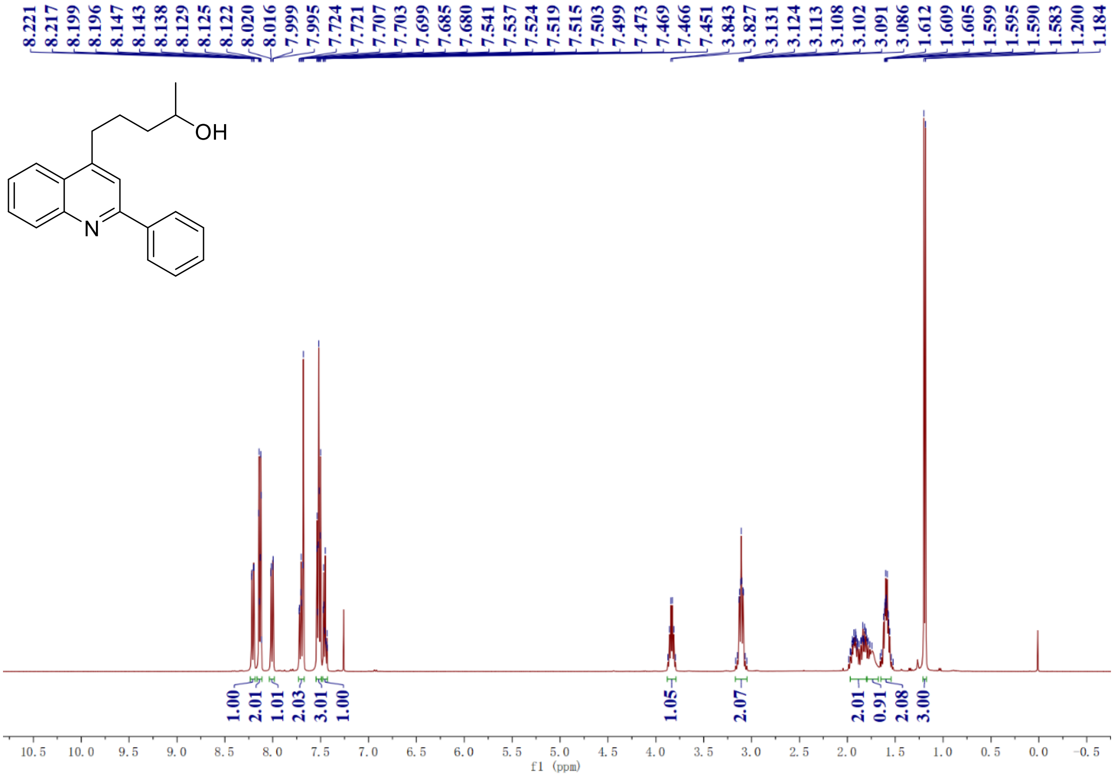

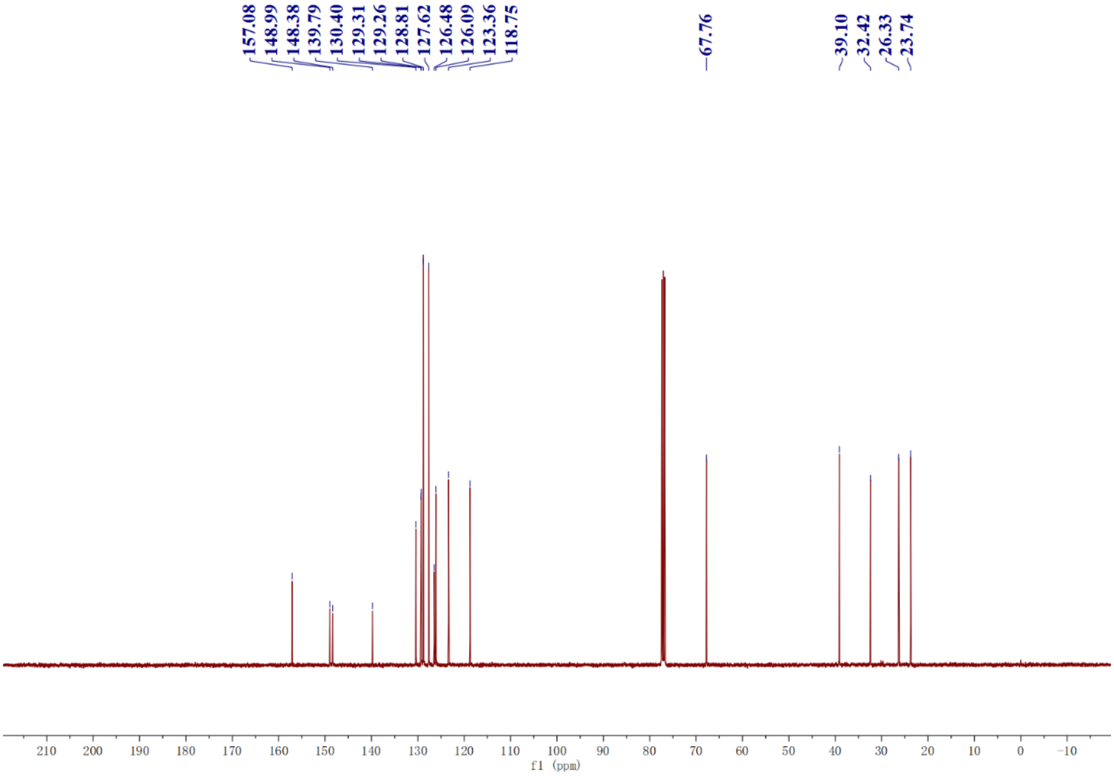

5-(2-phenylquinolin-4-yl)pentan-1-ol (85)

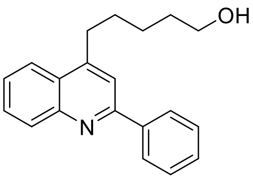

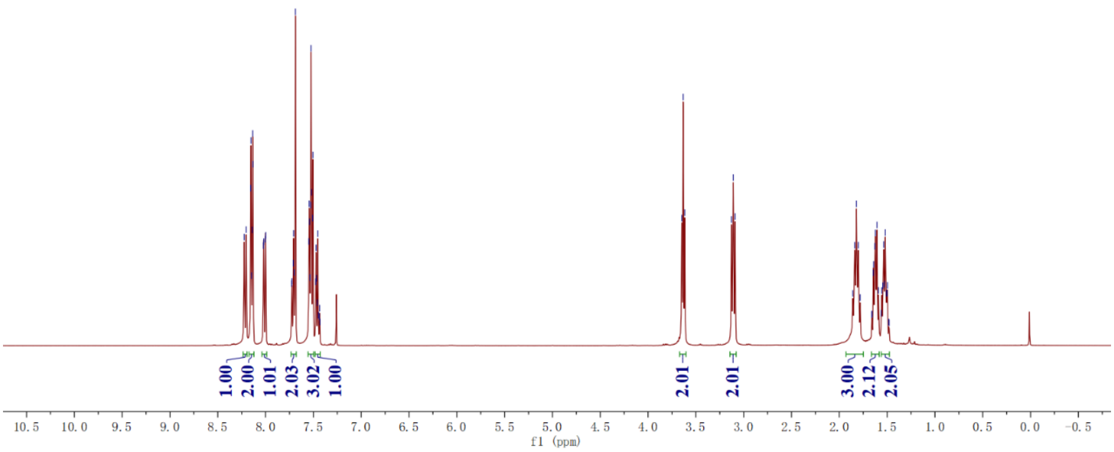

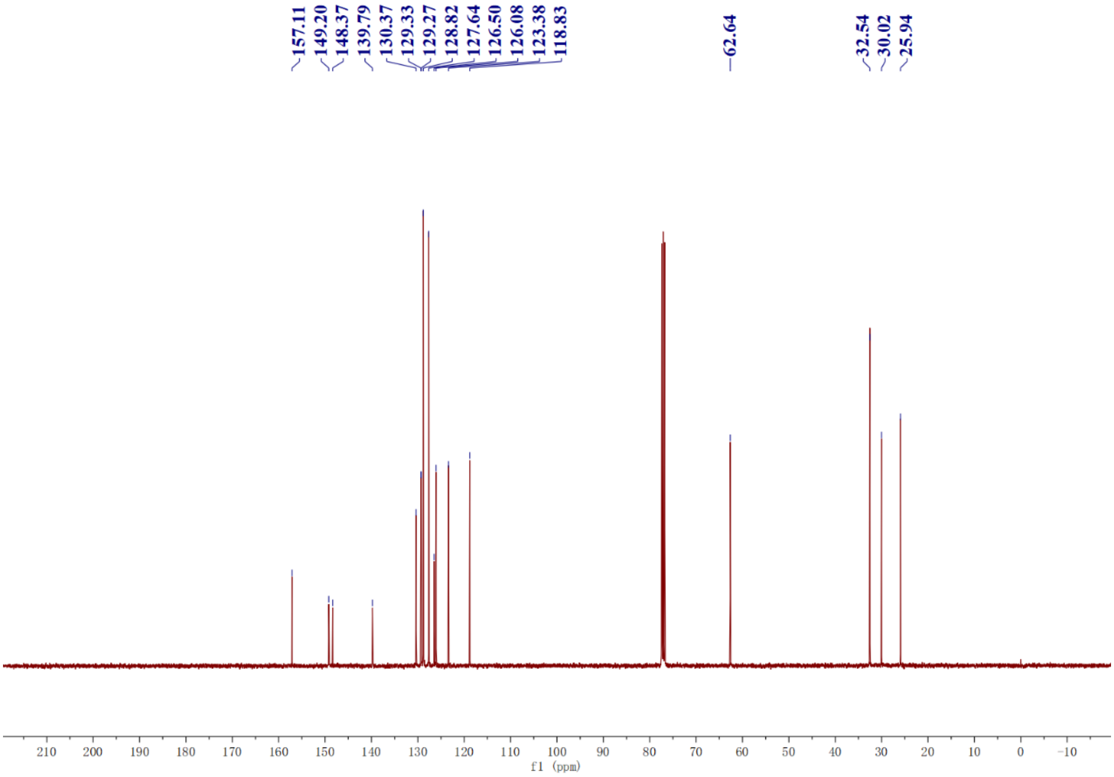

1-(2-phenylquinolin-4-yl)ethane-1,2-diol (86)

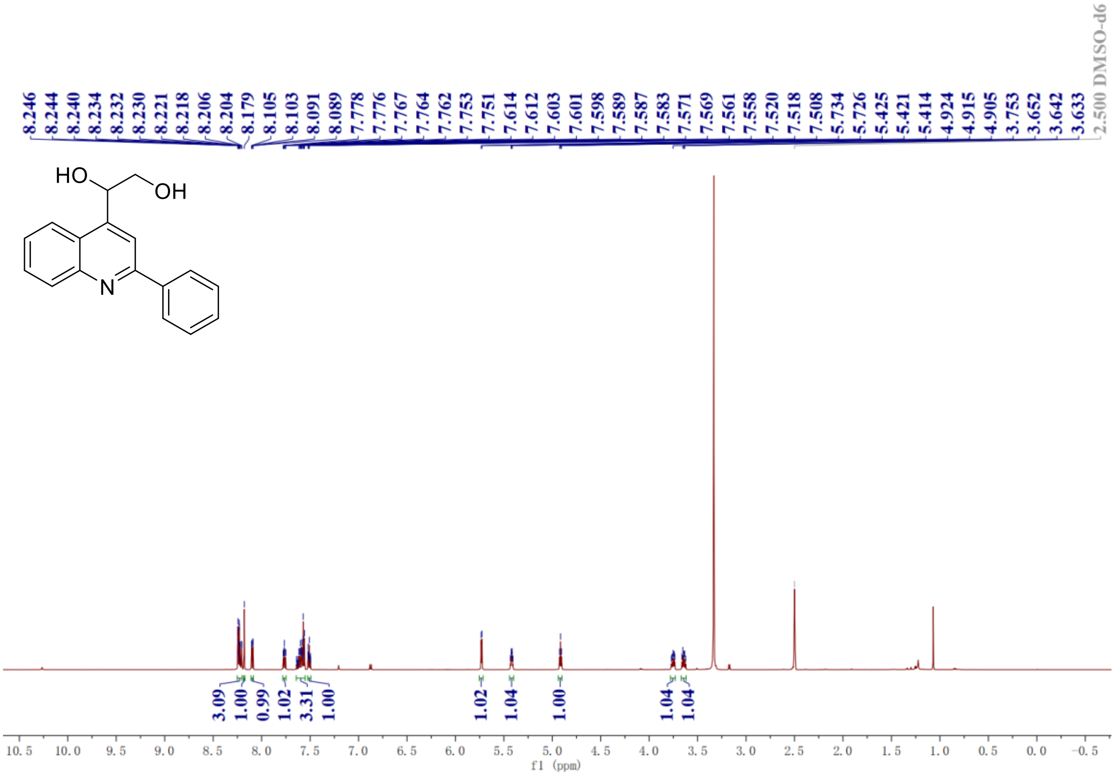

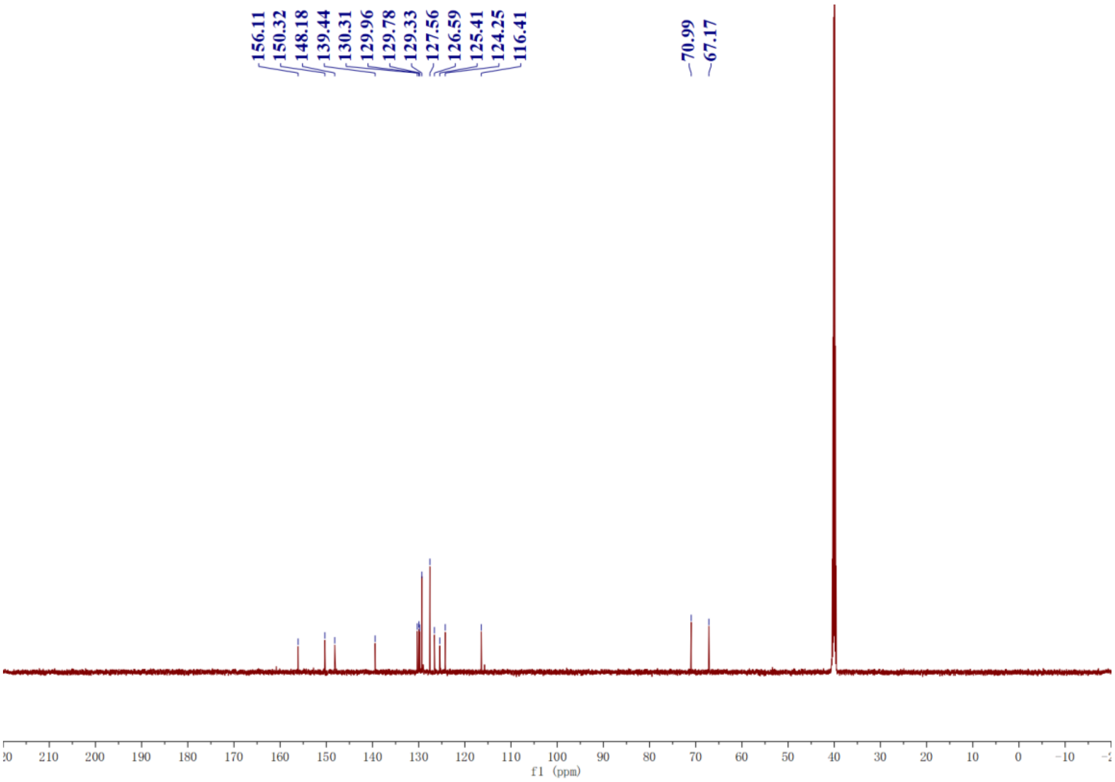

N-methyl-N-((2-phenylquinolin-4-yl)methyl)formamide (87)

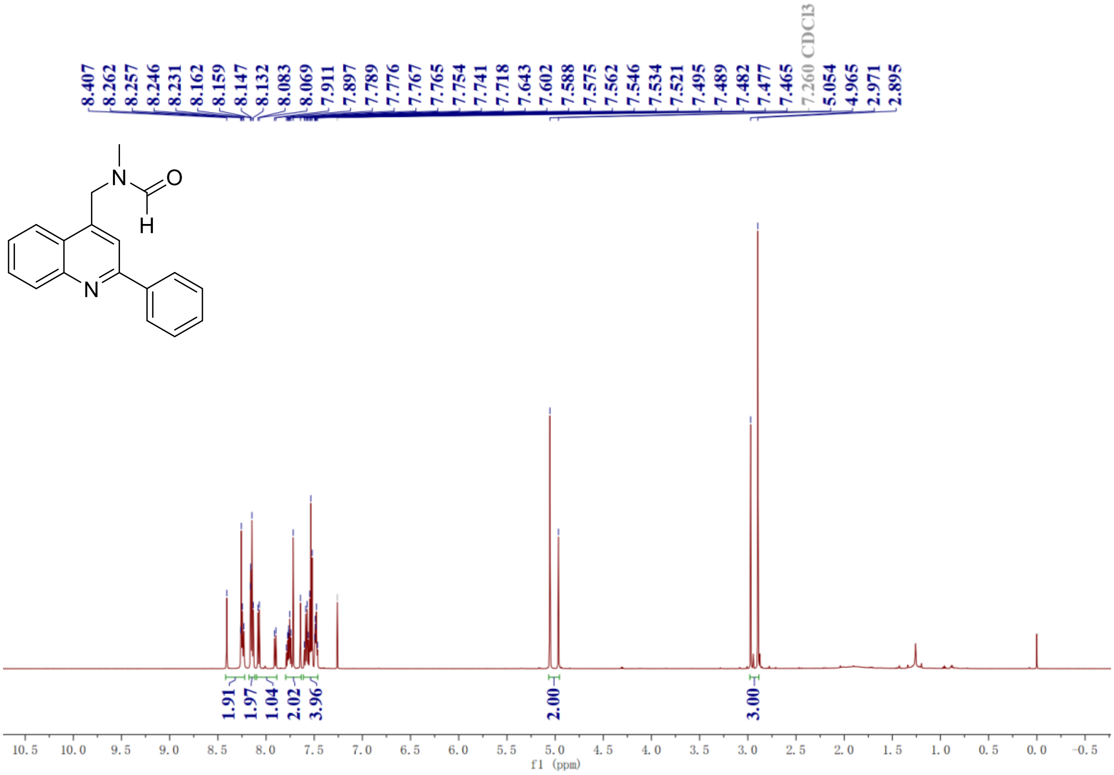

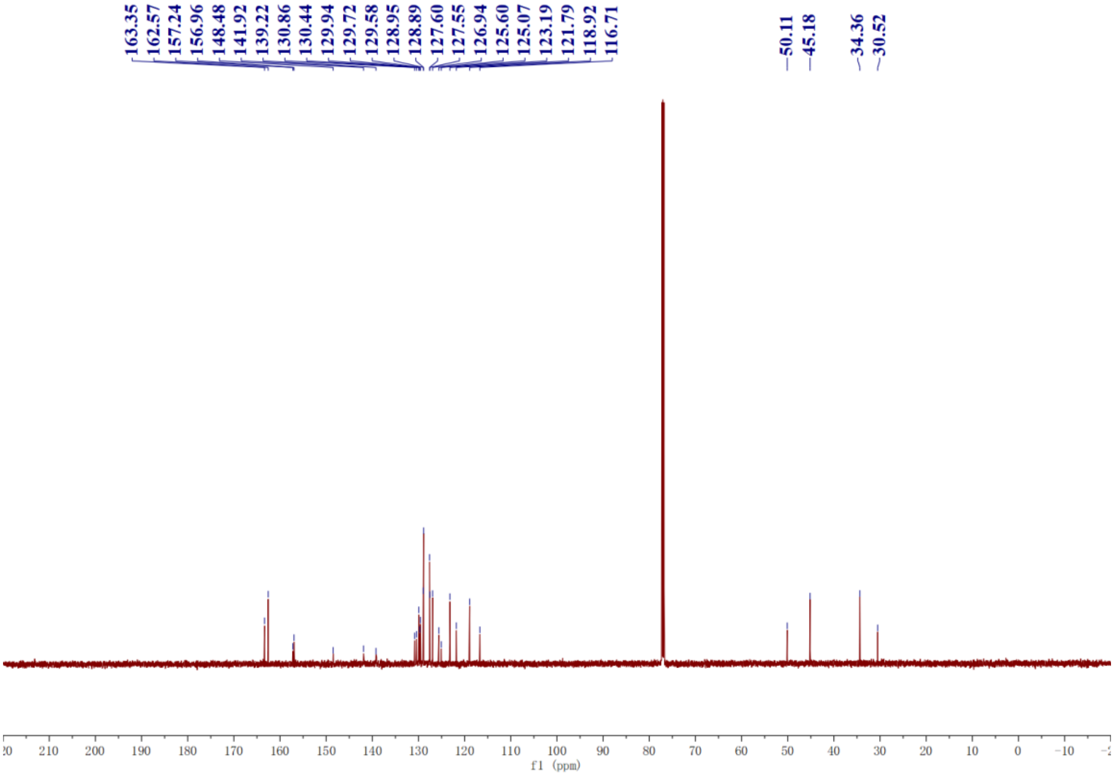

3-(2-phenylquinolin-4-yl)cyclohexan-1-one (88)

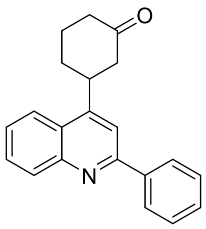

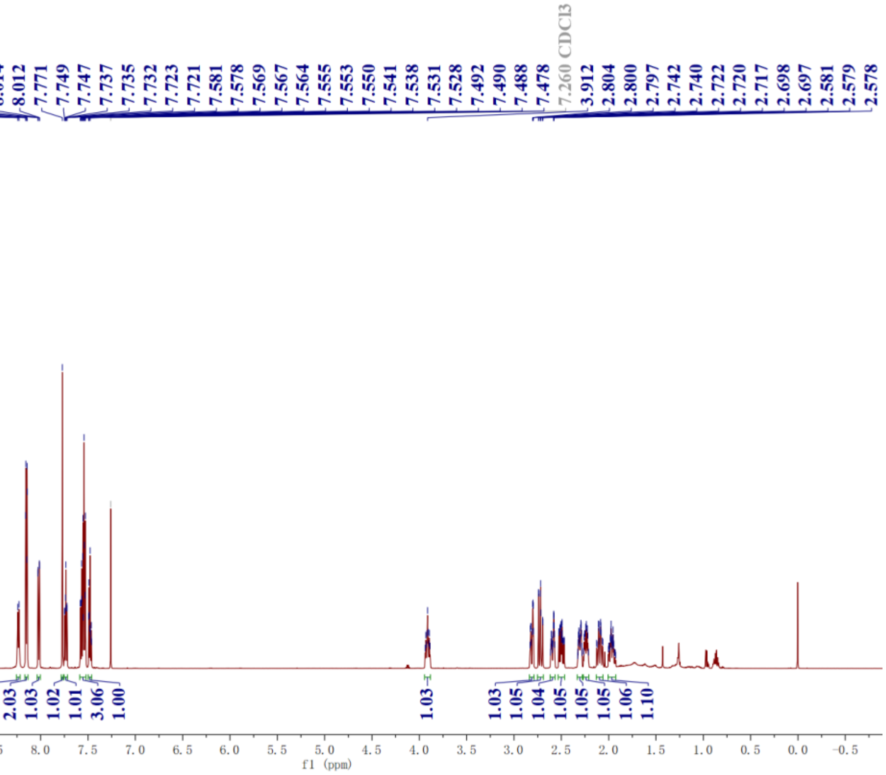

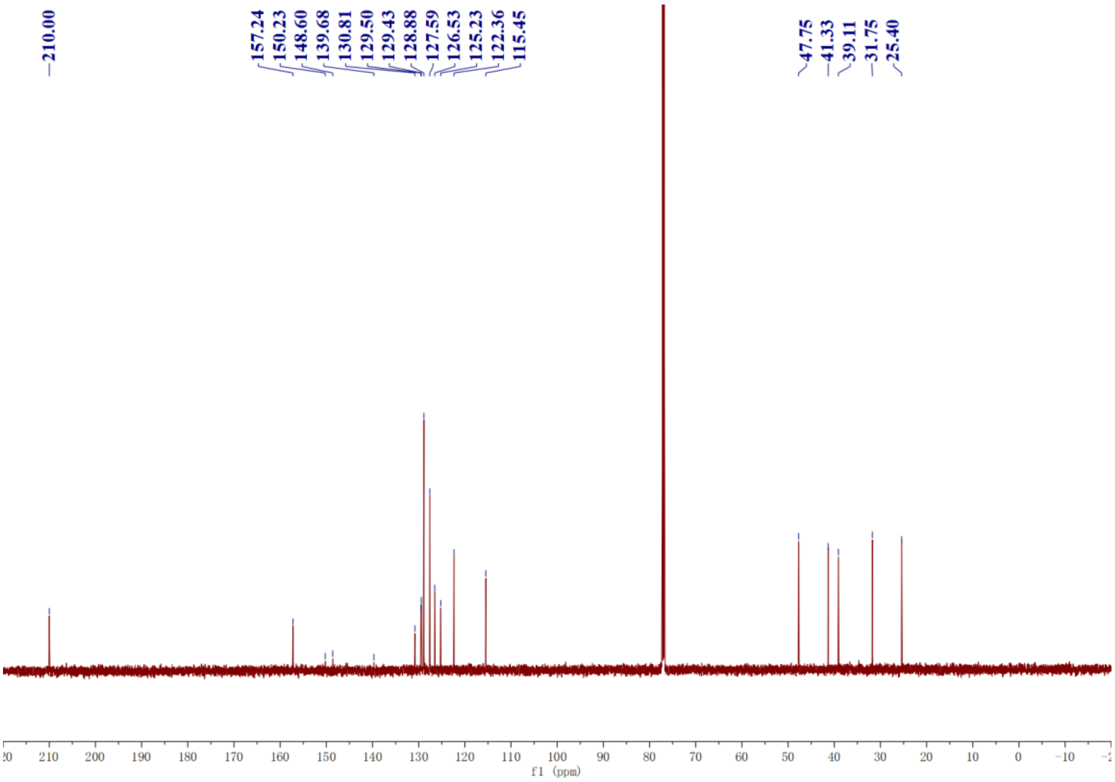

N-(6,6-dicyano-4-methyl-5-phenylhexyl)-4-methoxybenzenesulfonamide (89)

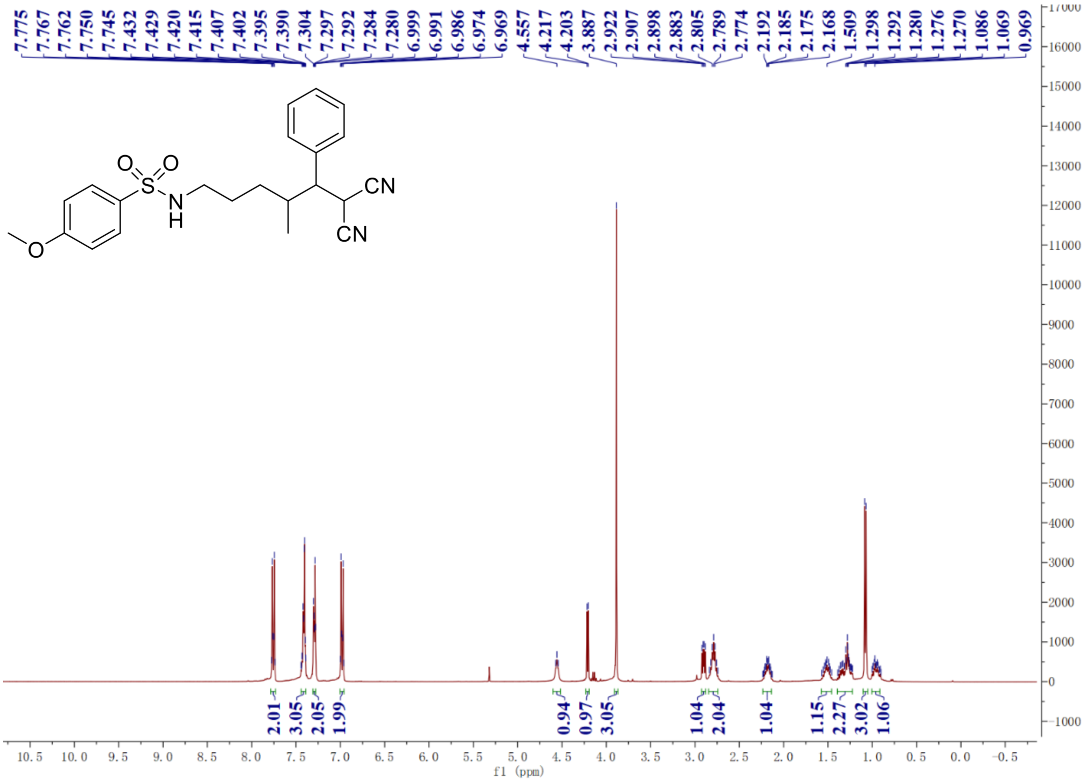

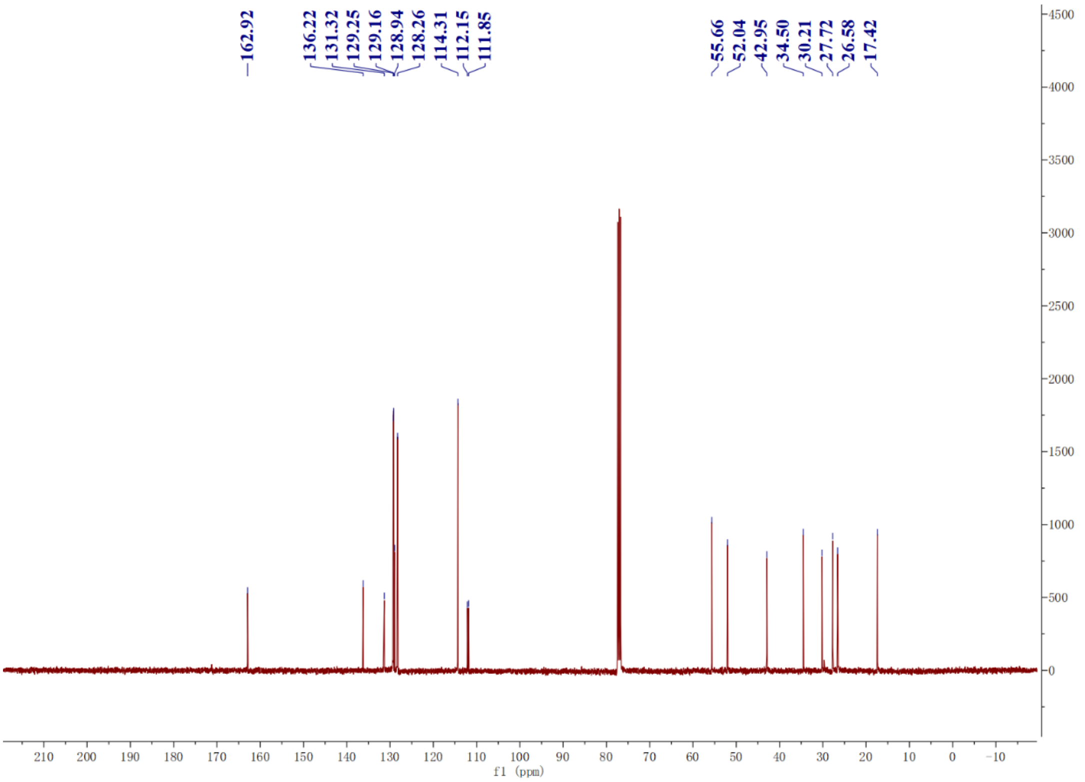

ethyl 2-cyano-7-((4-methoxyphenyl)sulfonamido)-4-methyl-3-phenylheptanoate (90)

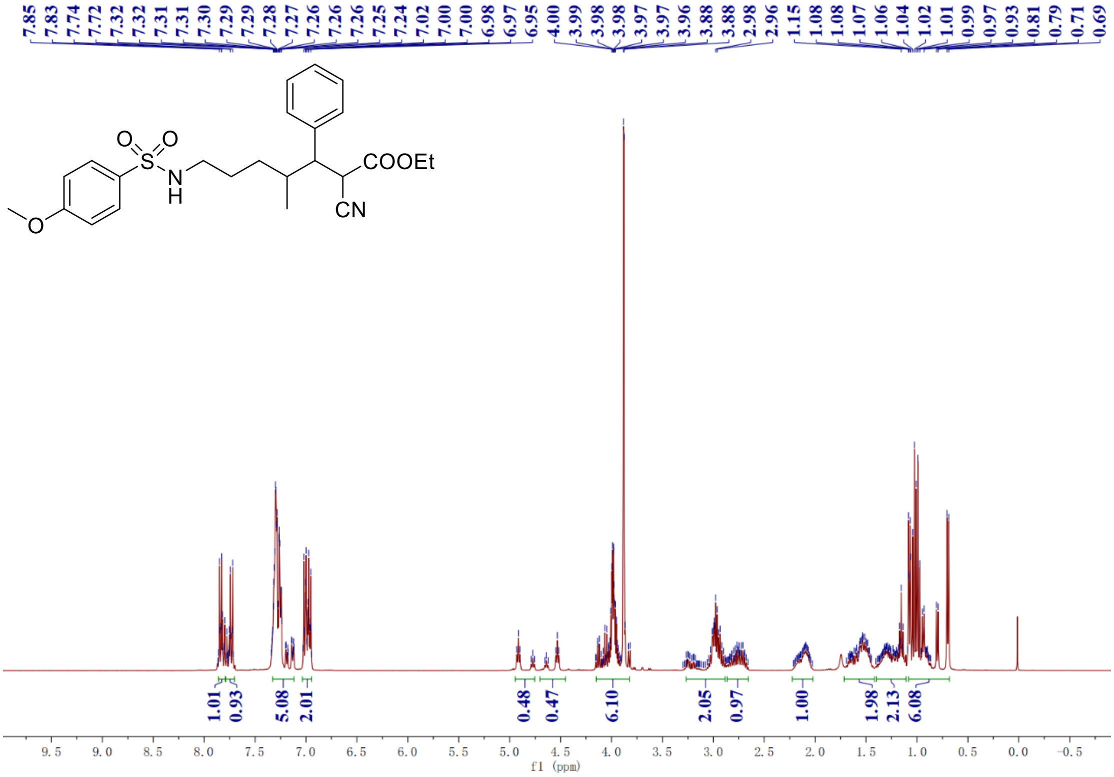

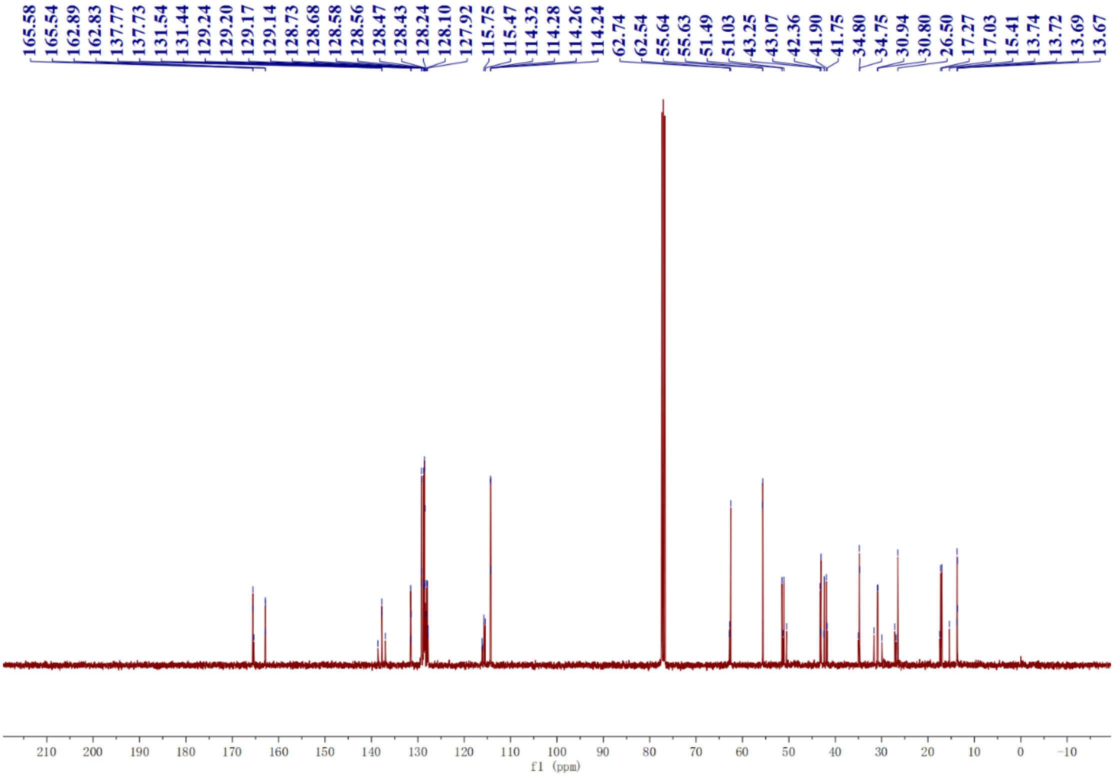

diethyl 2-(6-((4-methoxyphenyl)sulfonamido)-3-methylhexan-2-yl)malonate (91)

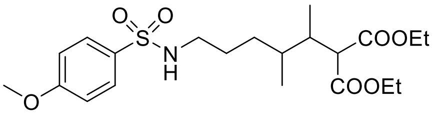

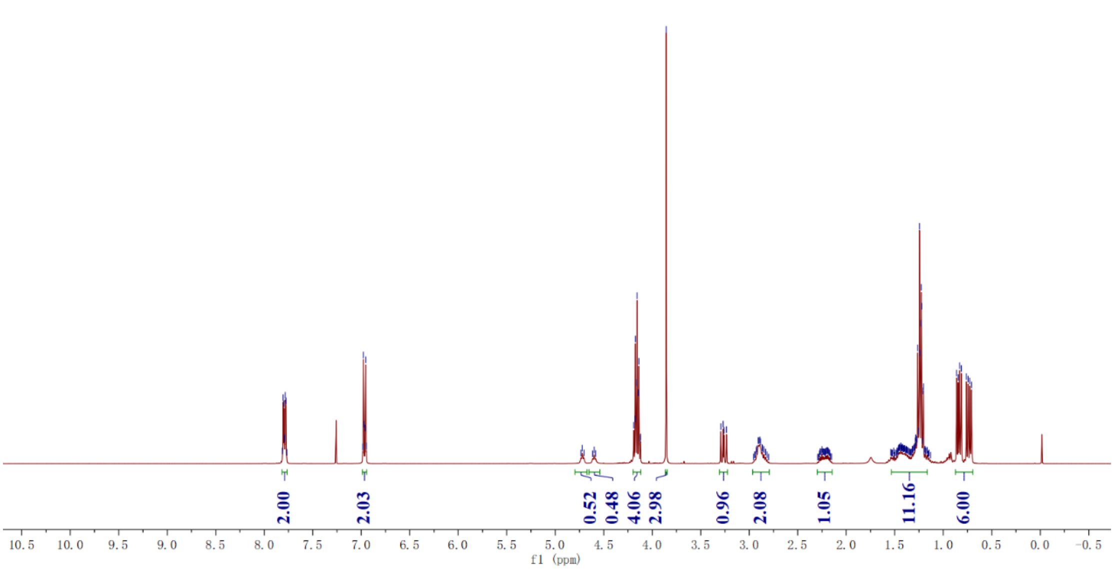

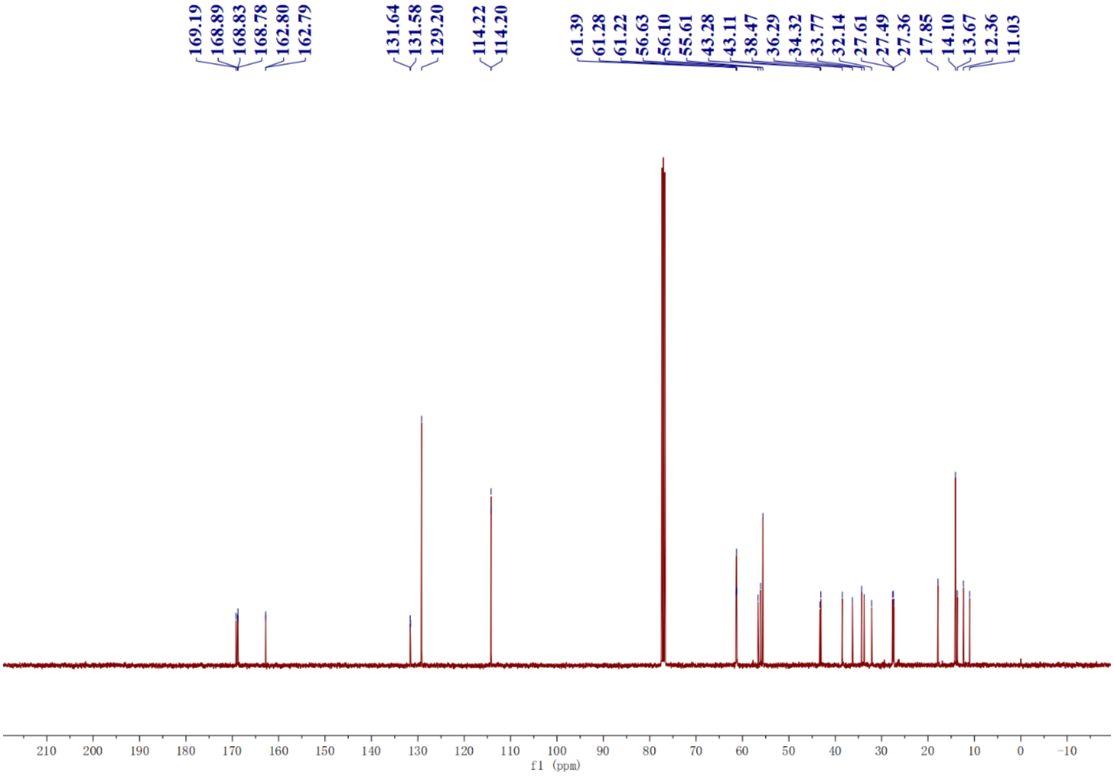

di-*tert*-butyl 1-(5-((4-methoxyphenyl)sulfonamido)pentan-2-yl)hydrazine-1,2-dicarboxylate (92)

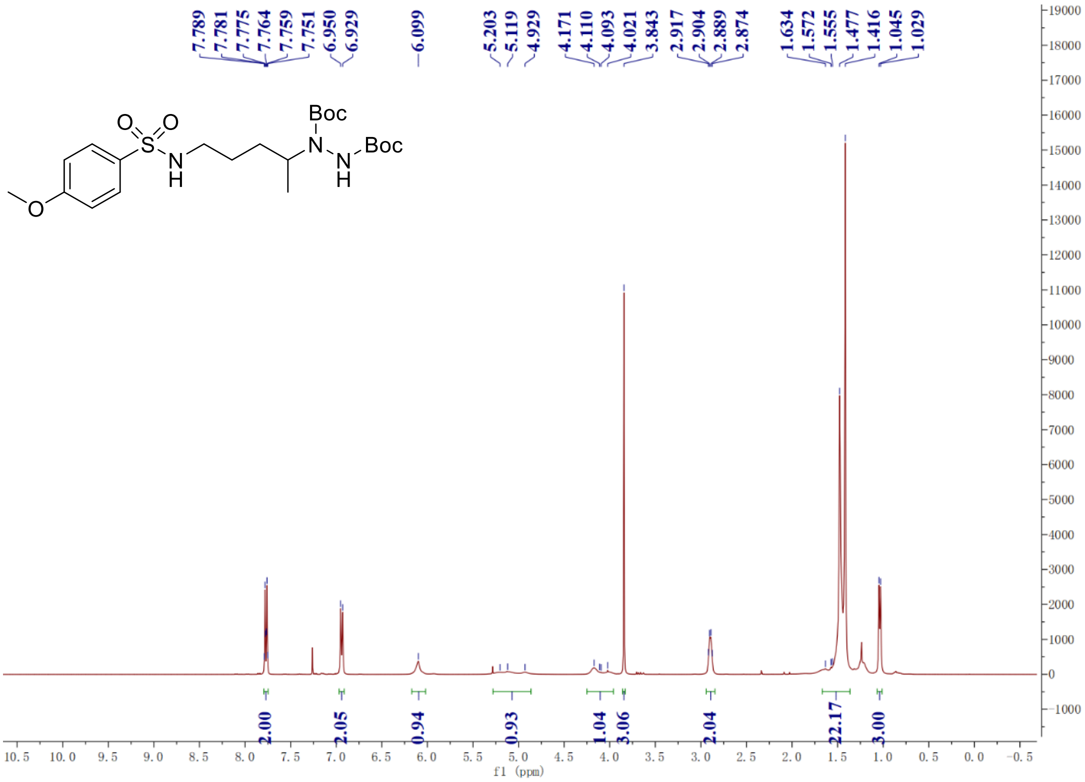

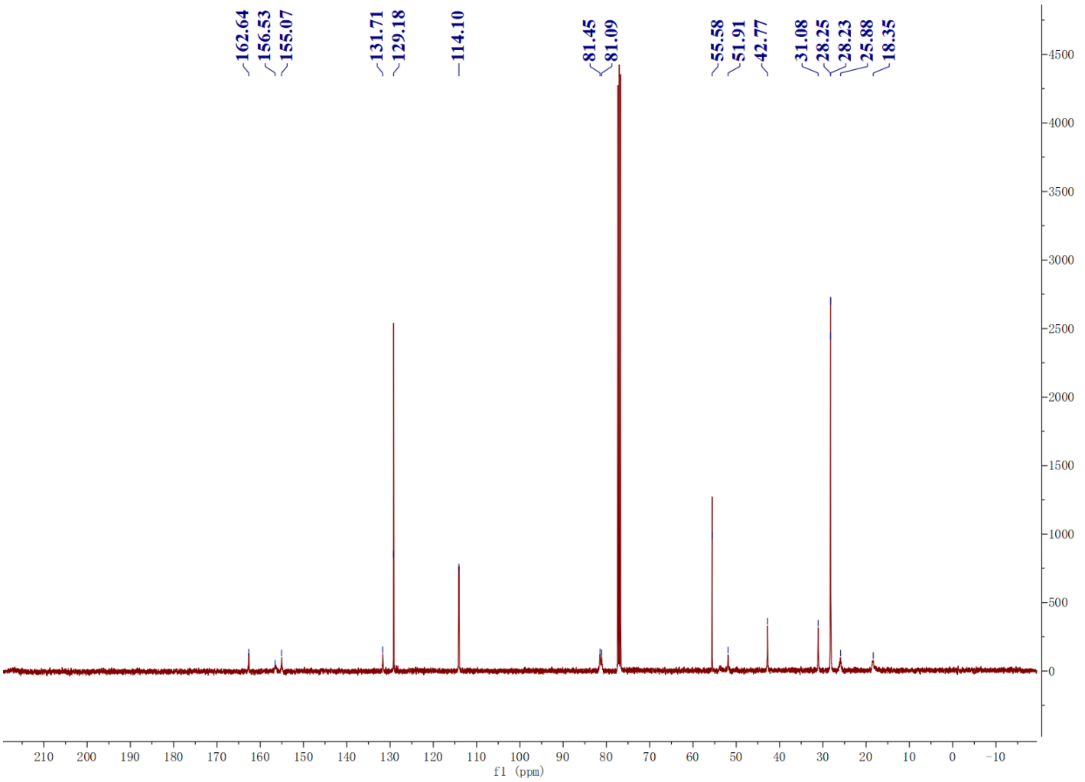

_N_-(4-azidopentyl)-4-methoxybenzenesulfonamide (93)

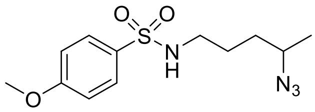

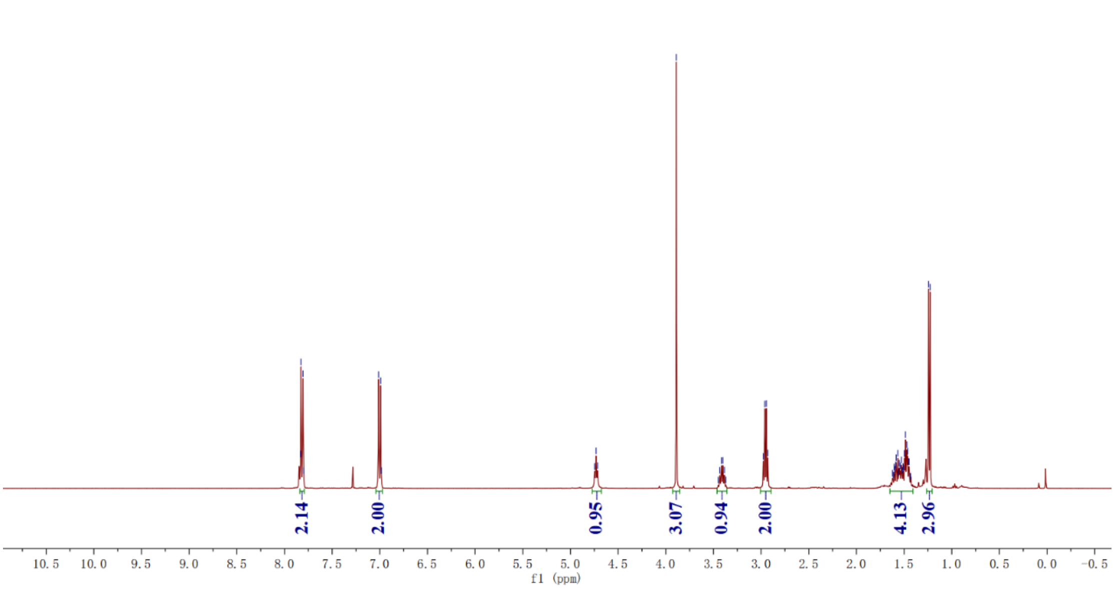

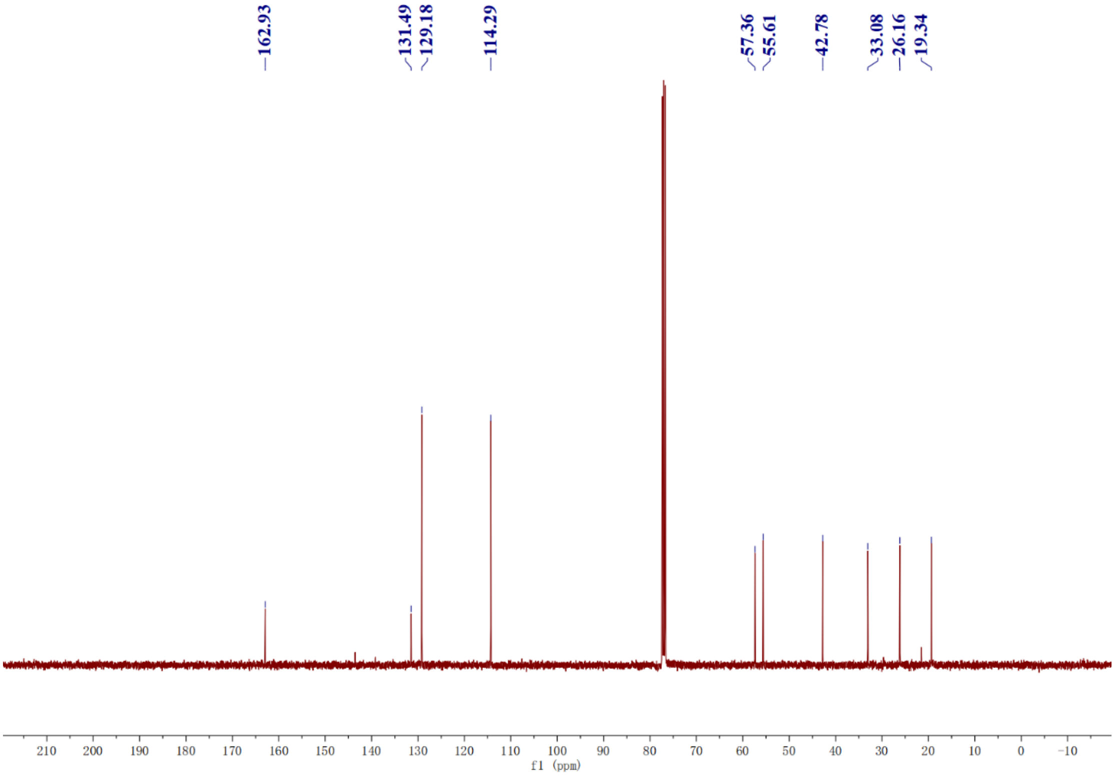

***N*-(4-cyanopentyl)-4-methoxybenzenesulfonamide (94)**

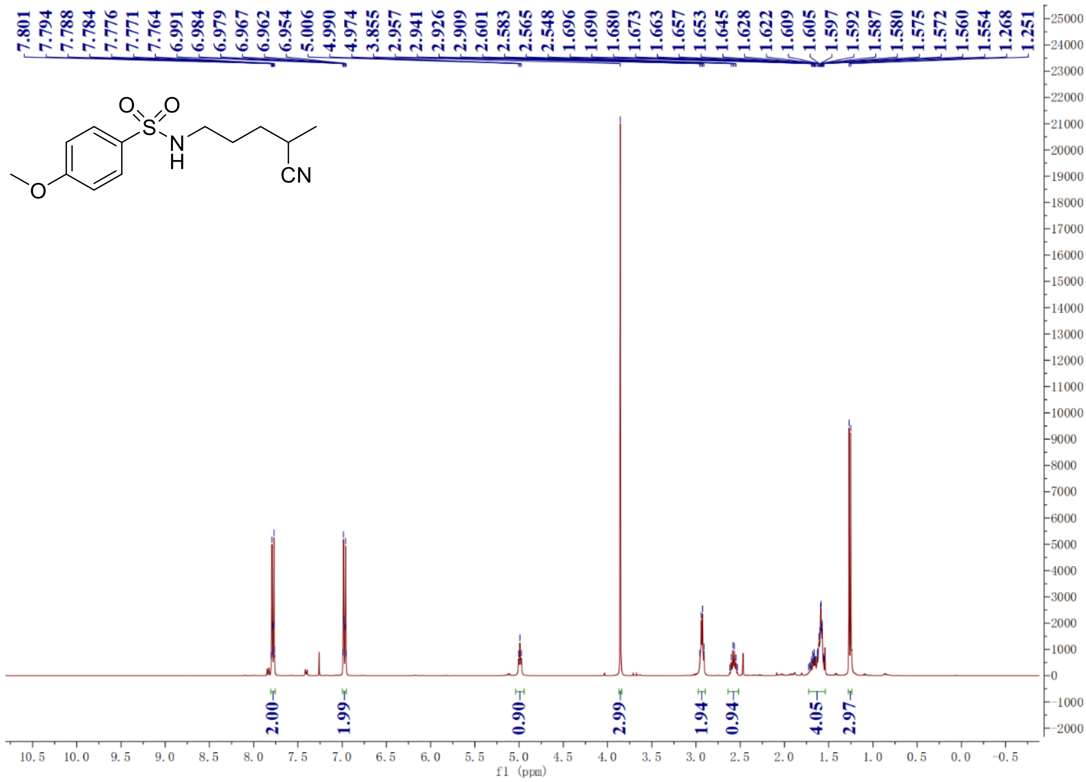

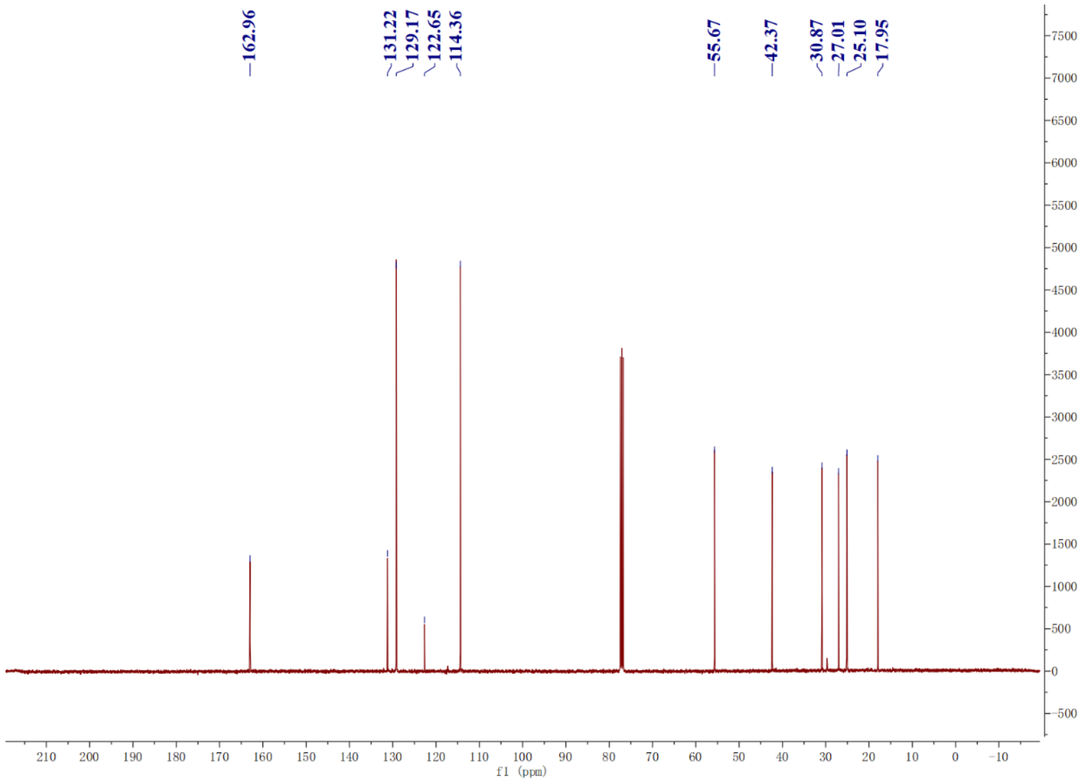

***N*-(4-bromopentyl)-4-methoxybenzenesulfonamide (95)**

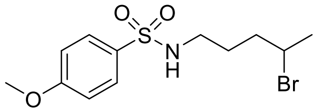

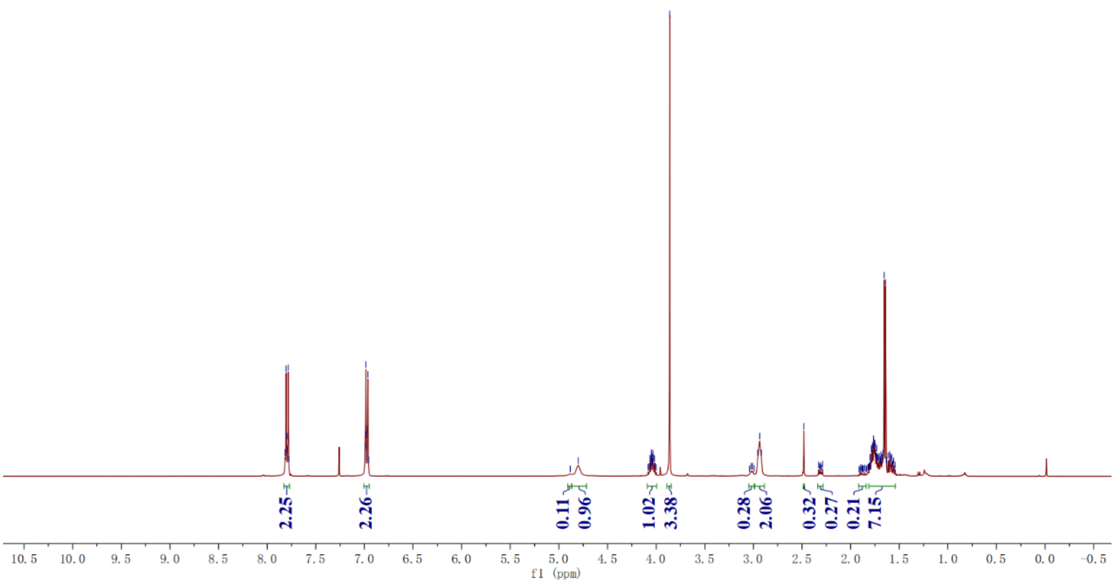

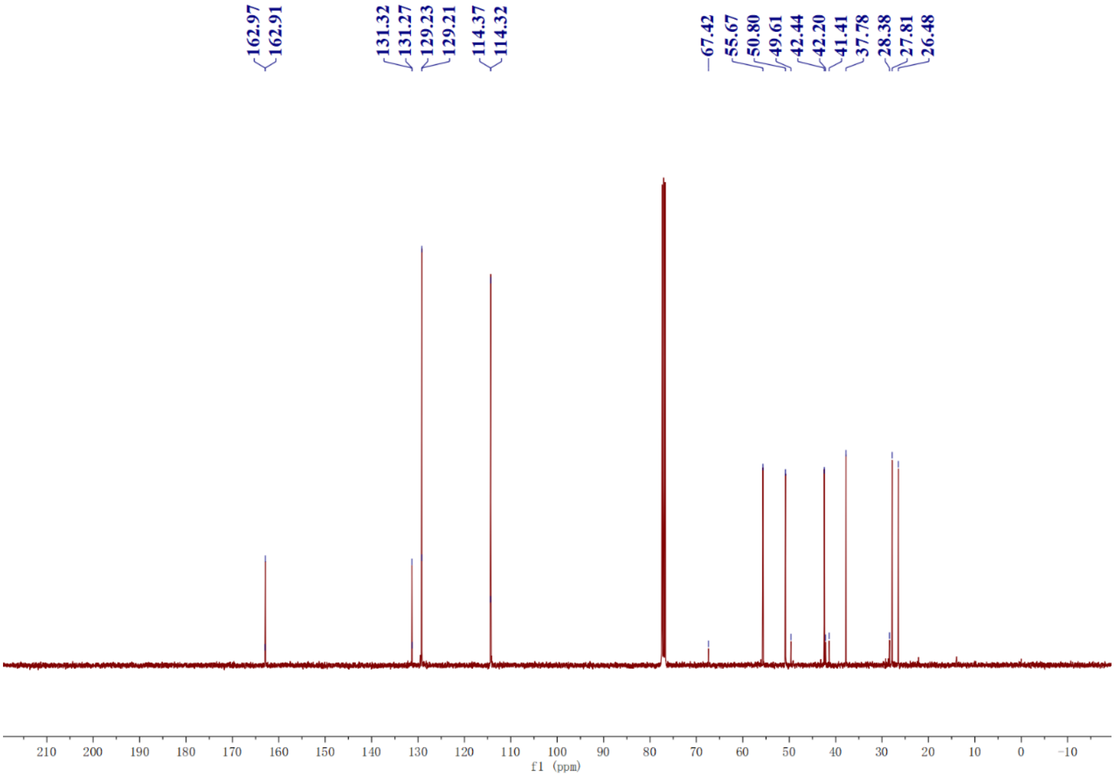

***N*-(4-fluoropentyl)-4-methoxybenzenesulfonamide (96)**

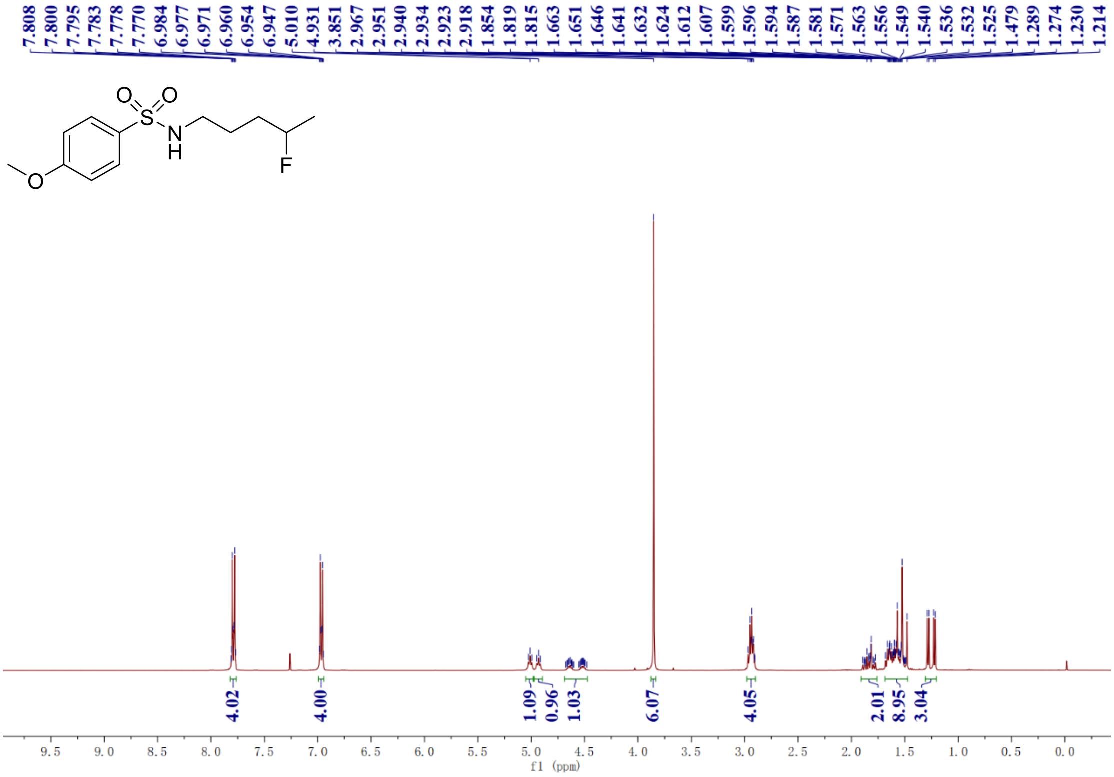

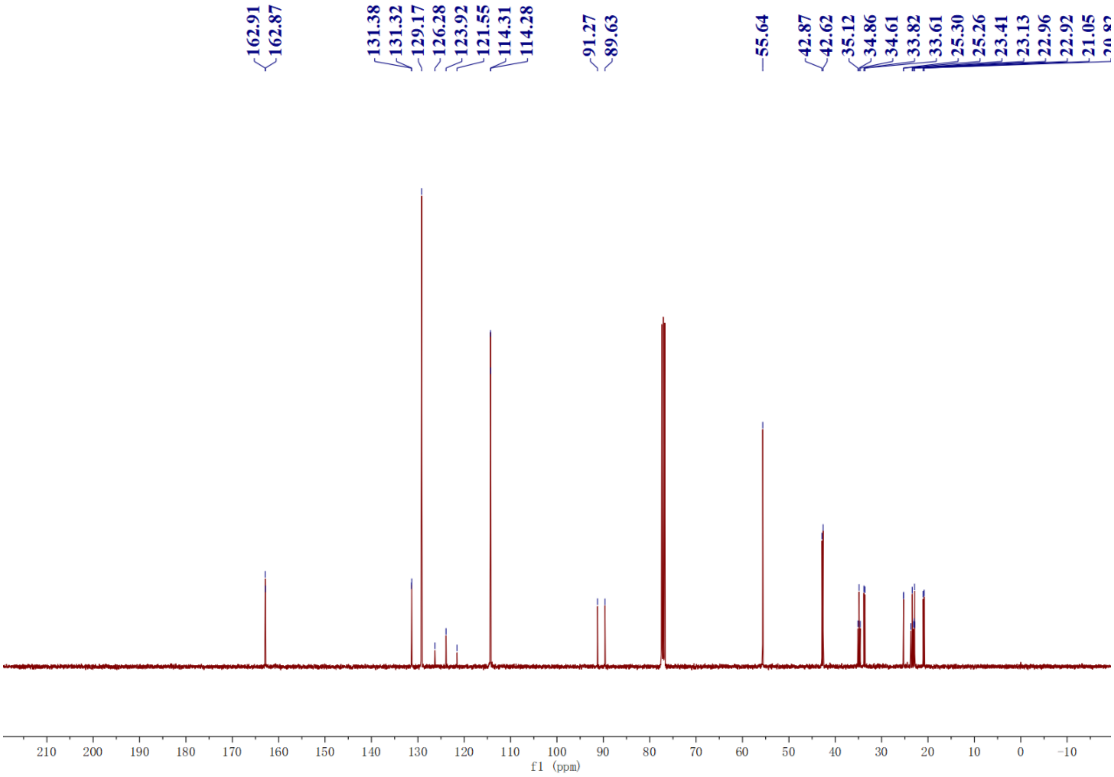

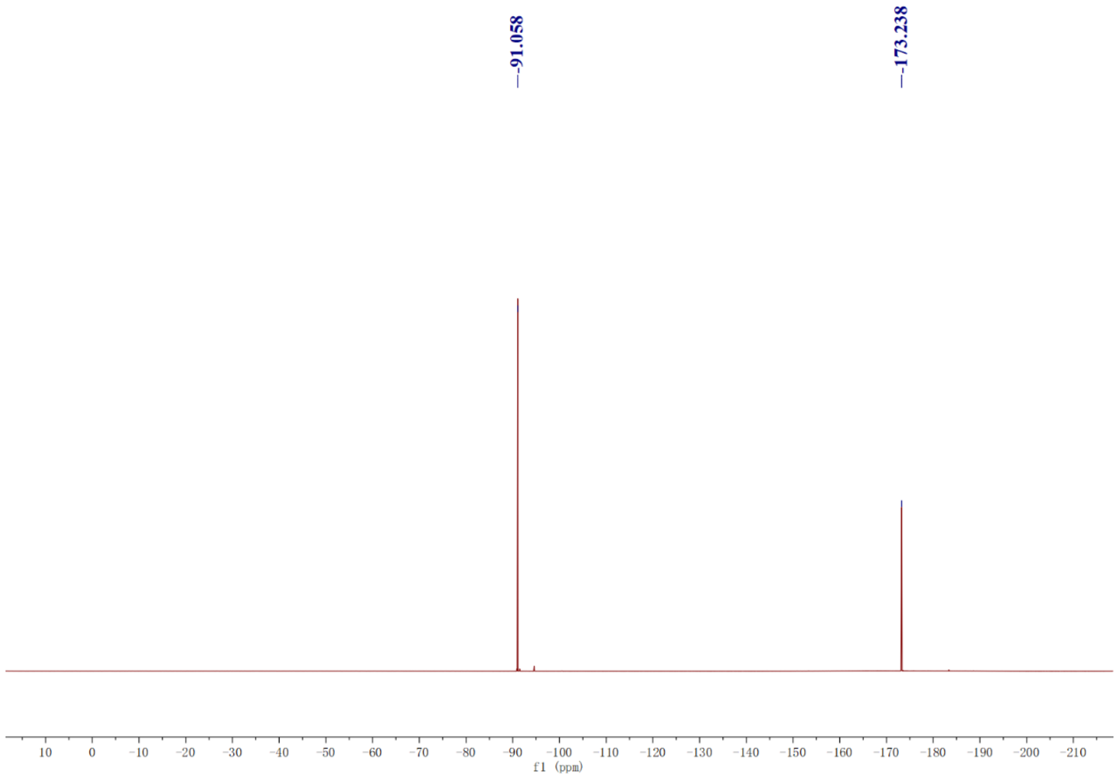
-91.058
-173.238
10 0 -10 -20 -30 -40 -50 -60 -70 -80 -90 -100 -110 -120 -130 -140 -150 -160 -170 -180 -190 -200 -210
f1 (ppm)

4-methoxy-*N*-(4-((trifluoromethyl)thio)pentyl)benzenesulfonamide (97)

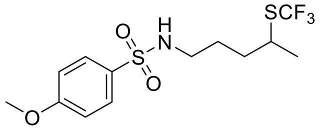

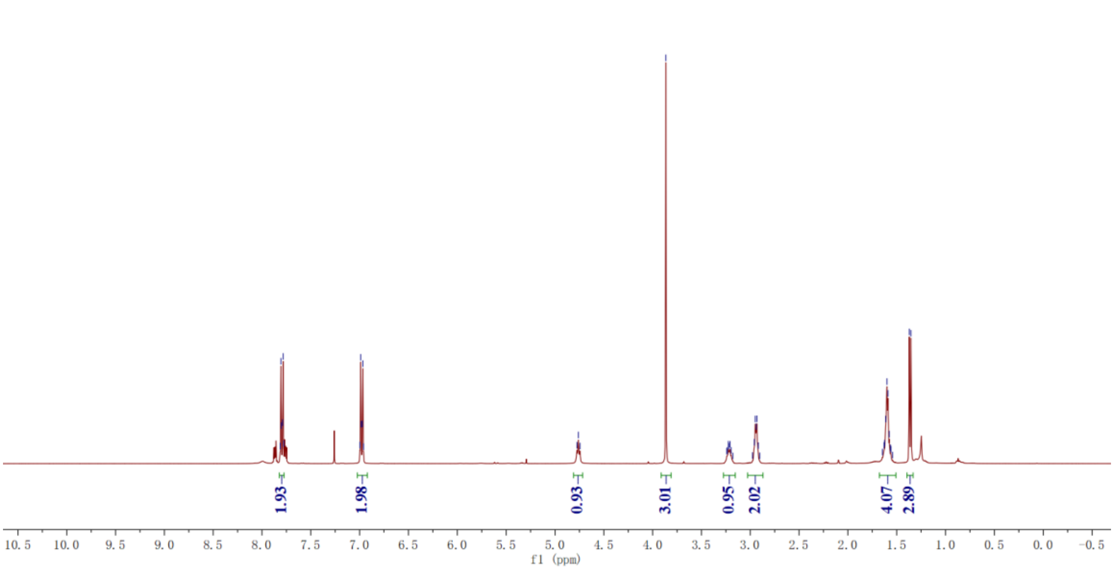

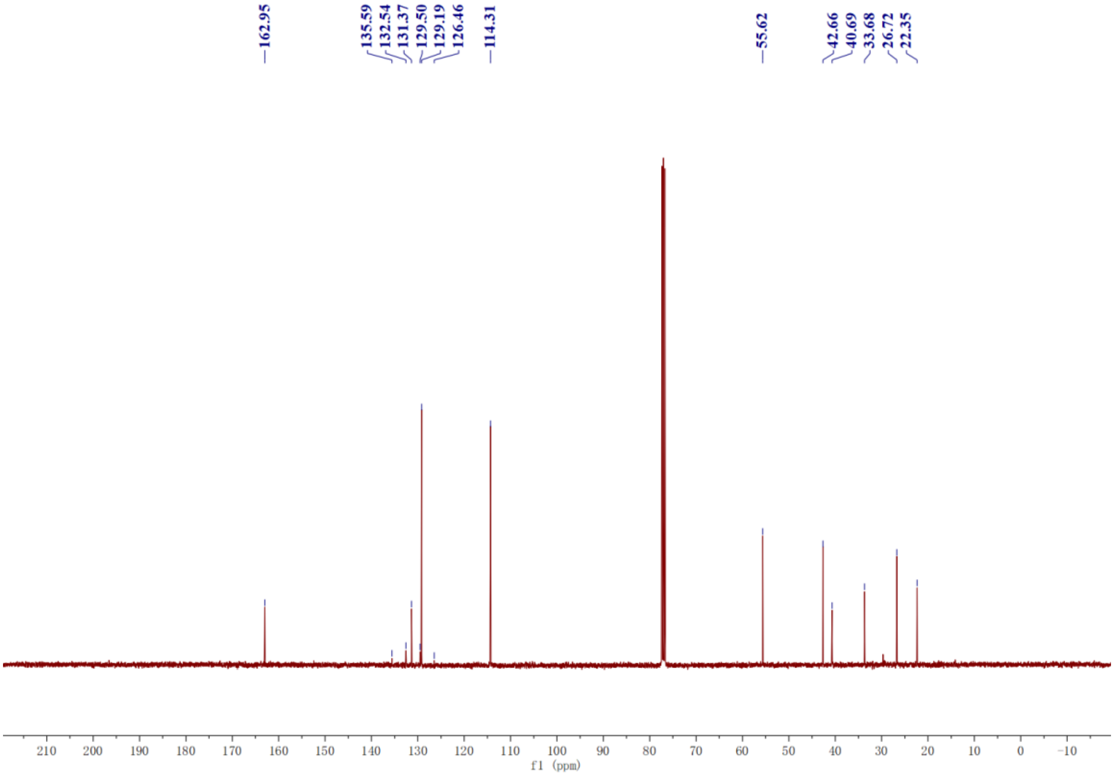

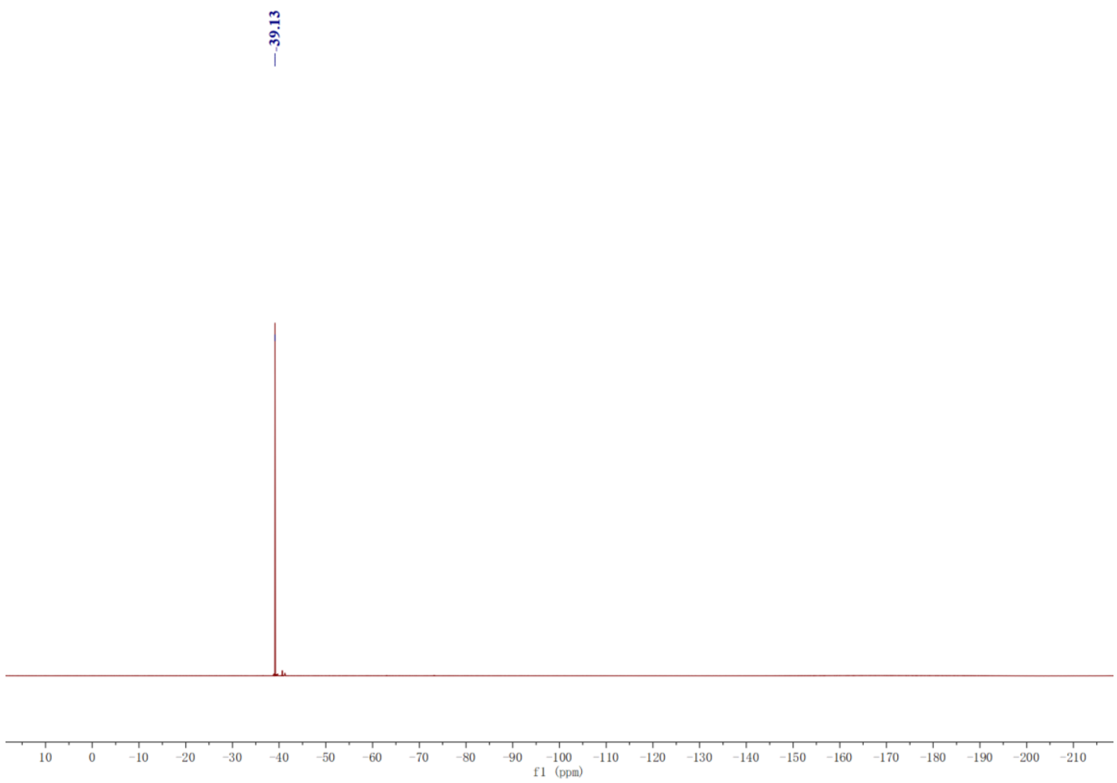
—39.13
10 0 −10 −20 −30 −40 −50 −60 −70 −80 −90 −100 −110 −120 −130 −140 −150 −160 −170 −180 −190 −200 −210
f1 (ppm)

4-methoxy-*N*-(pentyl-4-*d*)benzenesulfonamide (98)

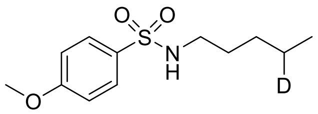

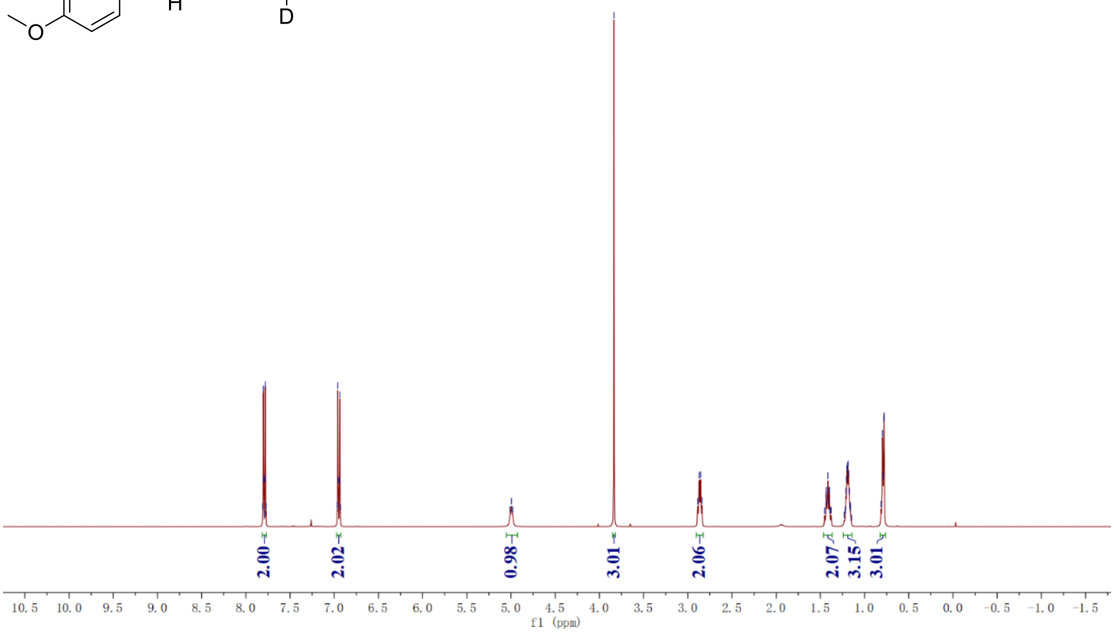

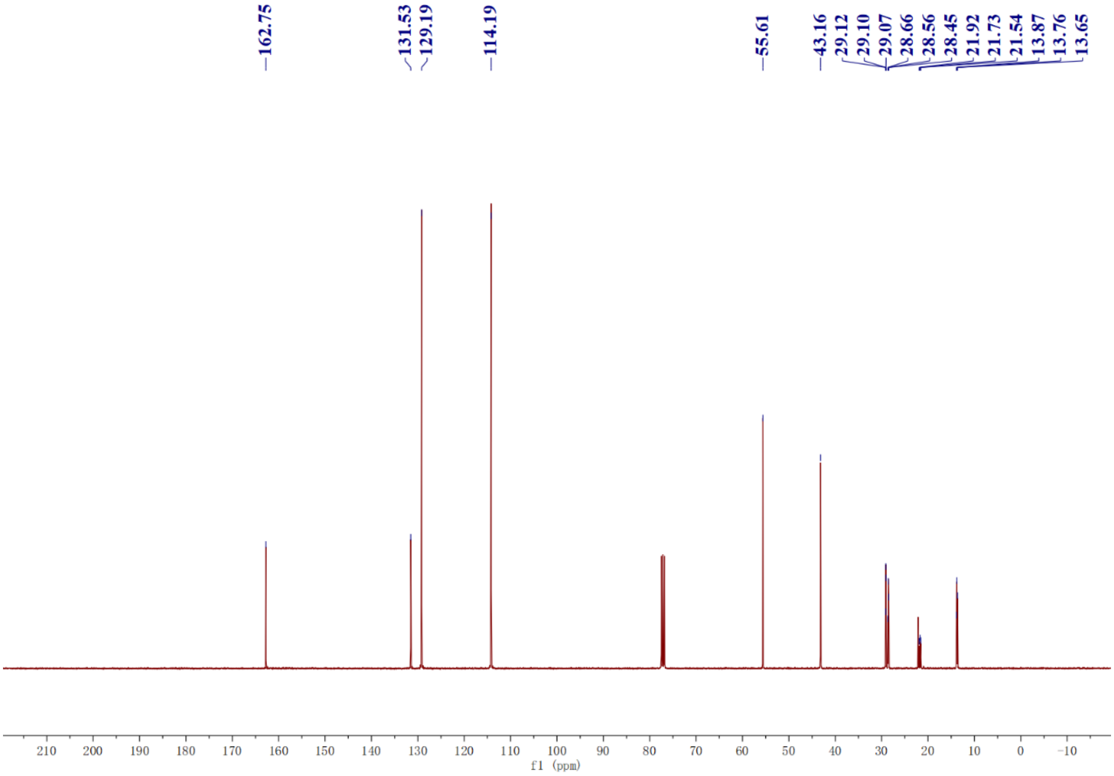

4-(1-((4-methoxyphenyl)sulfonyl)-2-methylpyrrolidin-2-yl)-2-phenylquinoline (99)

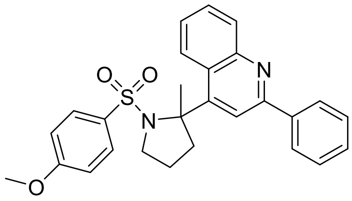

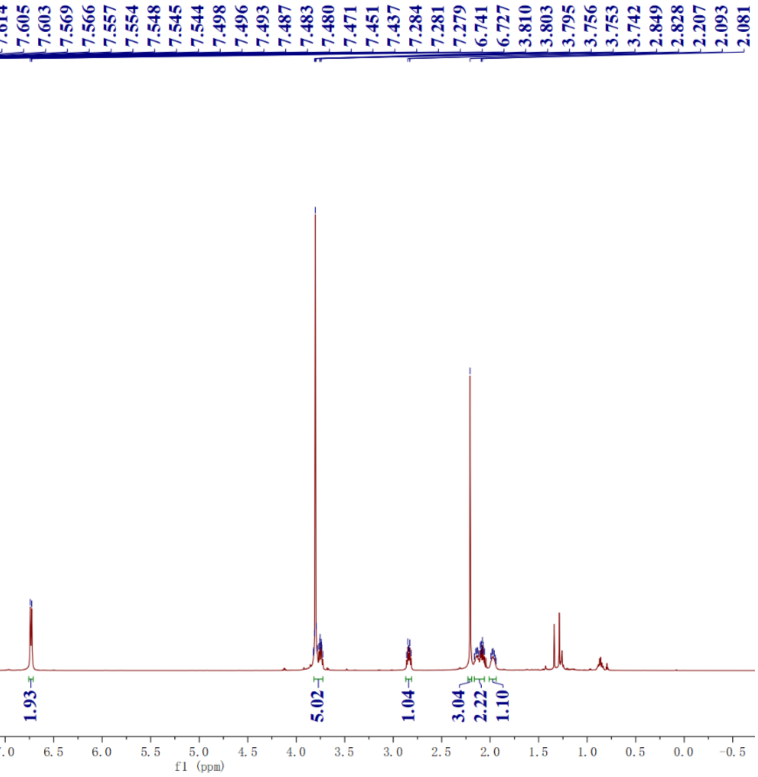

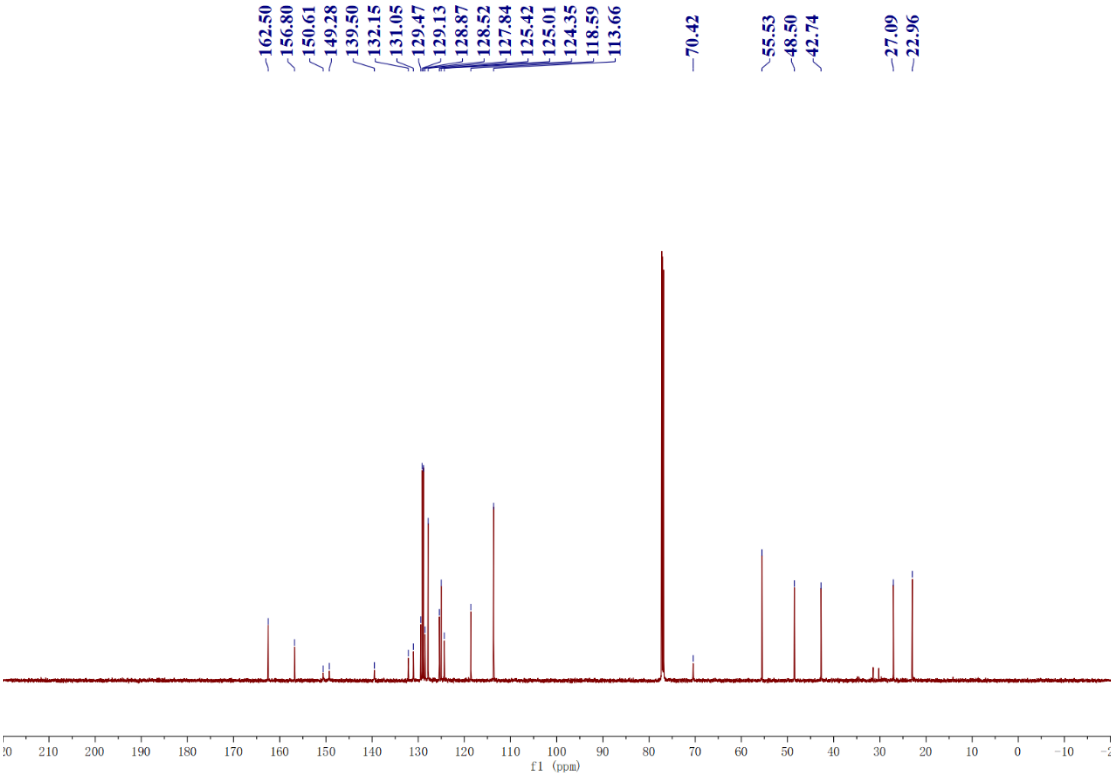

***N*-allyl-4-methoxy-*N*-(4-(2-phenylquinolin-4-yl)pentyl)benzenesulfonamide (100)**

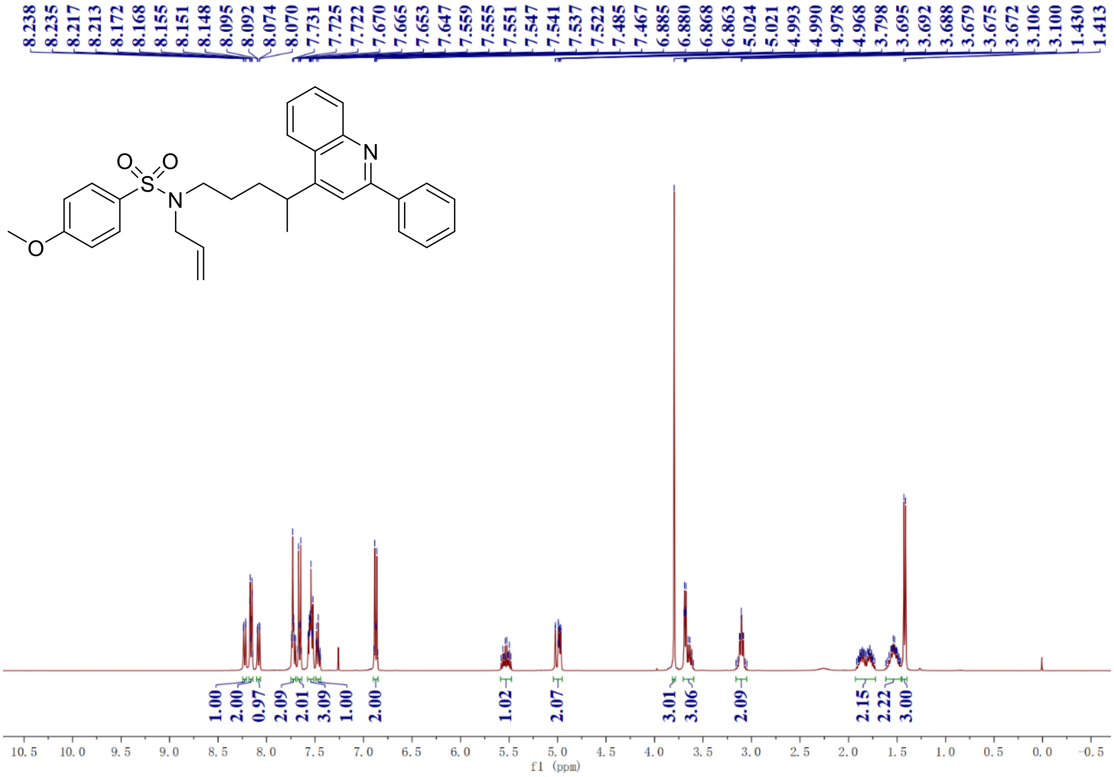

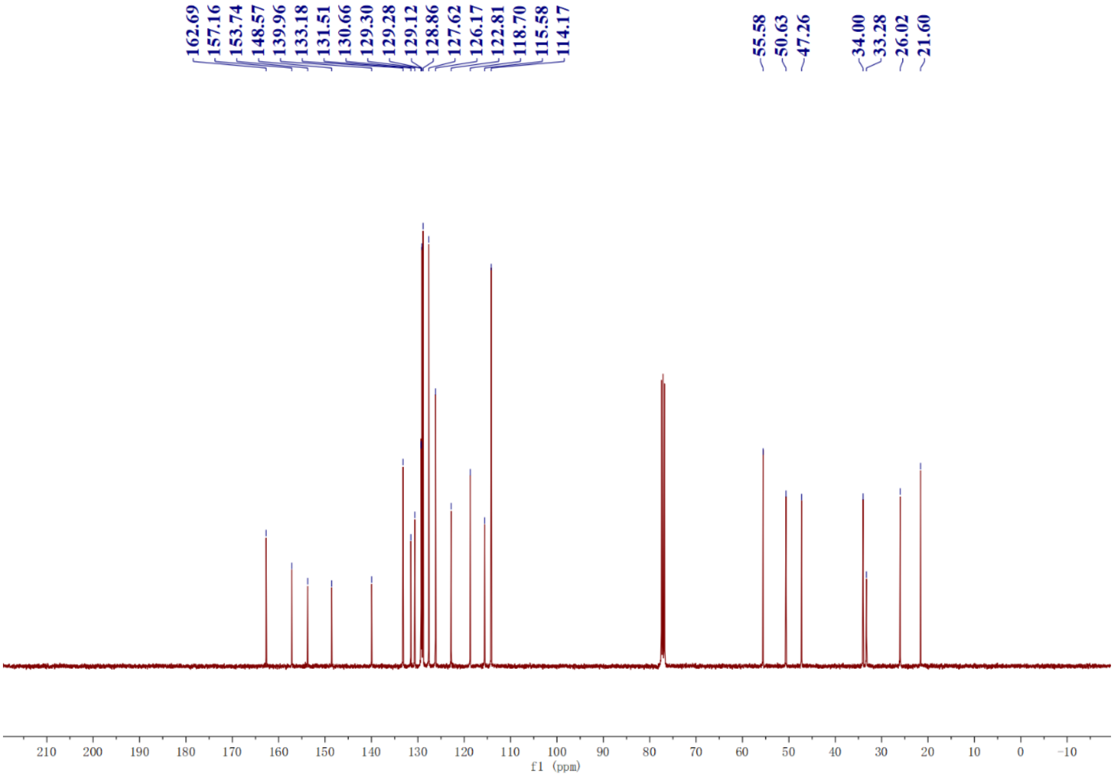

4-methoxy-*N*-pentyl-*N*-((2-phenylquinolin-4-yl)methyl)benzenesulfonamide (103)

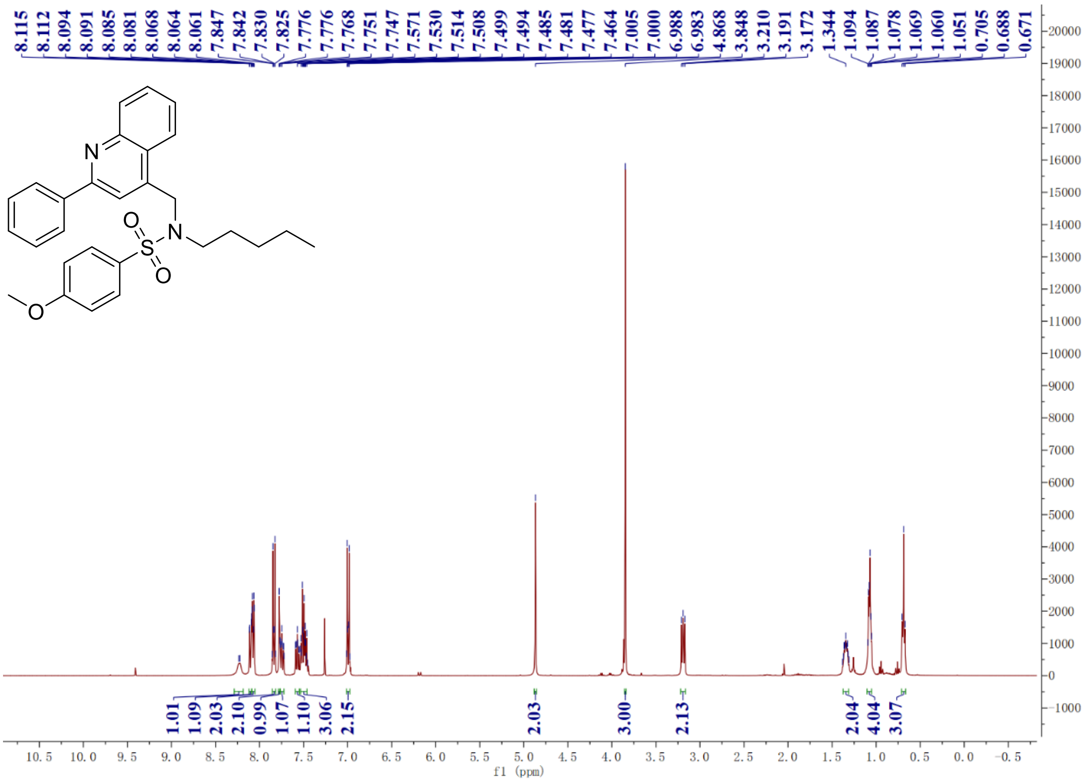

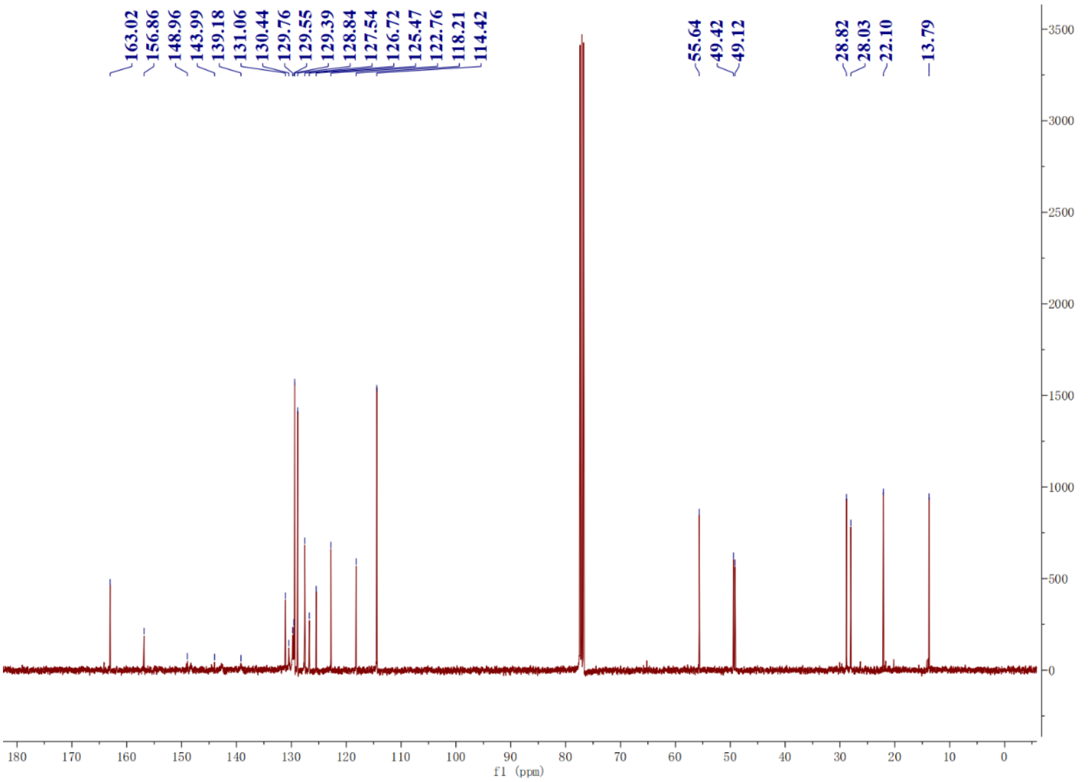

9. Supplementary References

[1] Frisch, M. J.; Trucks, G. W.; Schlegel, H. B.; Scuseria, G. E.; Robb, M. A.; Cheeseman, J. R.; Scalmani, G.; Barone, V.; Petersson, G. A.; Nakatsuji, H.; Li, X.; Caricato, M.; Marenich, A. V.; Bloino, J.; Janesko, B. G.; Gomperts, R.; Mennucci, B.; Hratchian, H. P.; Ortiz, J. V.; Izmaylov, A. F.; Sonnenberg, J. L.; Williams; Ding, F.; Lipparini, F.; Egidi, F.; Goings, J.; Peng, B.; Petrone, A.; Henderson, T.; Ranasinghe, D.; Zakrzewski, V. G.; Gao, J.; Rega, N.; Zheng, G.; Liang, W.; Hada, M.; Ehara, M.; Toyota, K.; Fukuda, R.; Hasegawa, J.; Ishida, M.; Nakajima, T.; Honda, Y.; Kitao, O.; Nakai, H.; Vreven, T.; Throssell, K.; Montgomery Jr., J. A.; Peralta, J. E.; Ogliaro, F.; Bearpark, M. J.; Heyd, J. J.; Brothers, E. N.; Kudin, K. N.; Staroverov, V. N.; Keith, T. A.; Kobayashi, R.; Normand, J.; Raghavachari, K.; Rendell, A. P.; Burant, J. C.; Iyengar, S. S.; Tomasi, J.; Cossi, M.; Millam, J. M.; Klene, M.; Adamo, C.; Cammi, R.; Ochterski, J. W.; Martin, R. L.; Morokuma, K.; Farkas, O.; Foresman, J. B.; Fox, D. J. *Gaussian 16 Rev. A.01*, Wallingford, CT, 2016.

[2] Adamo, C. & Barone, V. Toward reliable density functional methods without adjustable parameters: The PBE0 model. *J. Chem. Phys.* **110**, 6158-6170, (1999).

[3] Grimme, S., Ehrlich, S. & Goerigk, L. Effect of the damping function in dispersion corrected density functional theory. *J. Comput. Chem.* **32**, 1456-1465, (2011).

[4] Weigend, F. & Ahlrichs, R. Balanced basis sets of split valence, triple zeta valence and quadruple zeta valence quality for H to Rn: Design and assessment of accuracy. *Phys. Chem. Chem. Phys.* **7**, 3297-3305, (2005).

[5] Miertuš, S., Scrocco, E. & Tomasi, J. Electrostatic interaction of a solute with a continuum. A direct utilizaion of AB initio molecular potentials for the prevision of solvent effects. *Chem. Phys.* **55**, 117-129, (1981).

[6] Marenich, A. V., Cramer, C. J. & Truhlar, D. G. Universal Solvation Model Based on Solute Electron Density and on a Continuum Model of the Solvent Defined by the Bulk Dielectric Constant and Atomic Surface Tensions. *J. Phys. Chem. B* **113**, 6378-6396, (2009).

[7] Chen, Z. et al. Photoinduced regioselective difluorination of secondary inert C(sp^3)–H bonds in sulfonamides via 1,5-hydrogen-atom transfer. *Org. Chem. Front.* **10**, 4709-4717, (2023).

[8] Modak, A., Pinter, E. N. & Cook, S. P. Copper-Catalyzed, N-Directed Csp^3–H Trifluoromethylthiolation (−SCF_3) and Trifluoromethylselenation (−$SeCF_3$). *J. Am. Chem. Soc.* **141**, 18405-18410, (2019).

[9] Deng, Z., Zhao, Z., He, G. & Chen, G. Photoredox-Mediated Mono- and Difluorination of Remote Unactivated Methylene C(sp^3)–H Bonds of N-Alkyl Sulfonamides. *Org. Lett.* **23**, 3631-3635, (2021).
